# Modular Assembly of Chiral Cp* Clones Unlocks Performant Catalysts for Enantioselective C–H Functionalizations

**DOI:** 10.1021/jacs.6c05307

**Published:** 2026-04-22

**Authors:** Bram Van Den Bossche, Nicolai Cramer

**Affiliations:** Laboratory of Asymmetric Catalysis and Synthesis (LCSA), Institute of Chemical Sciences and Engineering, 27218 Ecole Polytechnique Fédérale de Lausanne (EPFL), 1015 Lausanne, Switzerland

## Abstract

Chiral cyclopentadienyl (Cp^x^) ligands have widespread applications in asymmetric transition-metal catalysis. Yet, further ligand development is crucial to unlock different reactivity outcomes and break through current selectivity boundaries. Past ligand design efforts have primarily focused on incorporating new chiral backbones into disubstituted Cp^x^ entities. In contrast, modification of the substitution degree of the Cp^x^ ring and diversification of the nature of these substituents remain largely underexplored. In this respect, a concise synthetic approach toward highly substituted Cp^x^ ligands with profound substituent flexibility is most desirable. Herein, we report a modular strategy for the rapid assembly of structurally diverse pentasubstituted Cp^x^ ligands (Cp^V^). Readily accessible 1,2,3-trifunctionalized cyclopentadiene building blocks are leveraged in a robust one-step dialkylation procedure, integrating a wide range of chiral *bis*-electrophiles. This combinatorial approach reduces the synthetic upfront investment of catalyst screenings and enables fast ligand diversification with extensive steric and electronic substituent tunability. Subsequent complexation with group 9 metals (Co, Rh, Ir) was amply demonstrated (50+ examples), and electronic parametrization of the ligands via their respective Cp^V^Rh phosphite species was performed. In selected exemplary asymmetric C–H functionalizations, the pentasubstituted Cp^V^ cobalt and rhodium catalysts acted as true chiral Cp* clones, delivering excellent reactivity under identical conditions. Easily introduced modifications of the Cp^V^ substituents prompted strong responses in stereoselectivity, including several instances of enantio-inversion. For each catalytic benchmark assessment, multiple Cp^V^ ligands directly outperformed their di- or trisubstituted Cp^x^ counterparts with simultaneously improved yields, diastereo-, and enantioselectivities. As such, the Cp^V^ platform addresses catalyst performance issues in challenging transformations, as well as substantially expands the reactivity and selectivity optimization options for future methodology development.

## Graphical abstract

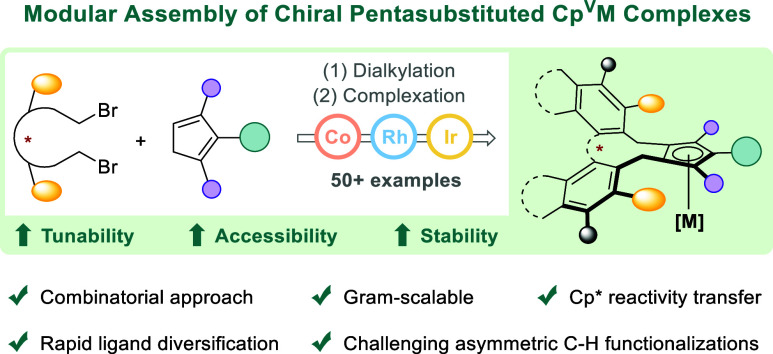

## Introduction

Chiral cyclopentadienyl (Cp^x^) ligands play a pivotal role in asymmetric transition-metal catalysis.^1^ In particular, their group 9 metal complexes (Co, Rh, Ir) have proven to be powerful C–H functionalization catalysts, enabling a broad range of diverse and valuable enantioselective transformations.^1a,2^ Yet, to push forward the current boundaries in reactivity, selectivity, and stability of these privileged complexes, new Cp^x^ ligand development remains crucial. Past efforts have predominantly focused on the incorporation of different chiral backbones into the ligand architecture, resulting in a broad portfolio of disubstituted Cp^x^ ligand classes **A**–**G** (Figure 1a, left),^3^ among others.^4^ The *C*_2_ symmetry of their Cp^x^H preligands, with both faces of the Cp^x^ ring being equivalent (except for **G**), is highly practical as it results in a single enantiomeric complex upon metalation. In addition, the synthesis typically relies on a dialkylation reaction with NaCp, enabling direct ligand access from well-established *bis*-electrophile precursors **L**. However, their in-class tuning is usually restricted to altering the so-called sidewall (SW) of the ligand. Much less attention has been paid to modifying the Cp^x^ ring, in terms of increasing its substitution degree and altering the nature of the installed substituents. For achiral Cps, careful modulation of the stereoelectronic nature, pattern, and number of substituents has been shown to drastically alter the overall catalytic performance of their metal complexes.^5–7^ In contrast, chiral highly substituted Cp^x^ ligands (bearing 3, 4, or 5 substituents) and synthetic strategies to access them remain underdeveloped.^8–10^ However, the handful of reported examples point toward a strong potential and underscore the importance of such developments (Figure 1a, right). For instance, trisubstituted Cp^x^ ligands **H** bearing a bulky 4-alkyl substituent (the so-called frontarm) enabled higher yields and enantioselectivities in multiple rhodium-,^11^ scandium-,^12^ and, most notably, cobalt-catalyzed^13^ C–H functionalizations. The synthesis of **H** involves two additional steps from its disubstituted analog **A**,^8a^ which has proven transferable to other backbone architectures.^8f,11a^ Yet, substituent choice is limited to alkyl groups, and the introduction of other functionalities on the Cp^x^ ring has only been sparsely explored (TMS^8b,8c^ and Ph^10a^) while coming at a synthetic cost (2–5 steps from **L**). The connection of a second chiral backbone unit to **A** yields tetrasubstituted derivatives such as *bis*(binaphthyl)-based ligands **I.** ^9a^ Finally, pentasubstituted ligands **J** and **K** also outperformed classical Cp^x^ architectures in their respective catalytic applications.^10a,10b^ However, the ligand architecture is so far highly restricted to trimethyl-bearing Cp^x^ rings, and their synthesis is step-intensive. Notably, the parallel development of prochiral Cp ligands **P1–5** en route to planar-chiral-only Cp^x^ metal complexes has broadened substituent diversity.^14^ However, recurring limitations include the reliance on resolution techniques or recrystallizations to procure enantiopure metal complexes, as well as challenging ligand design and concomitant selectivity understanding.

**Figure 1:**
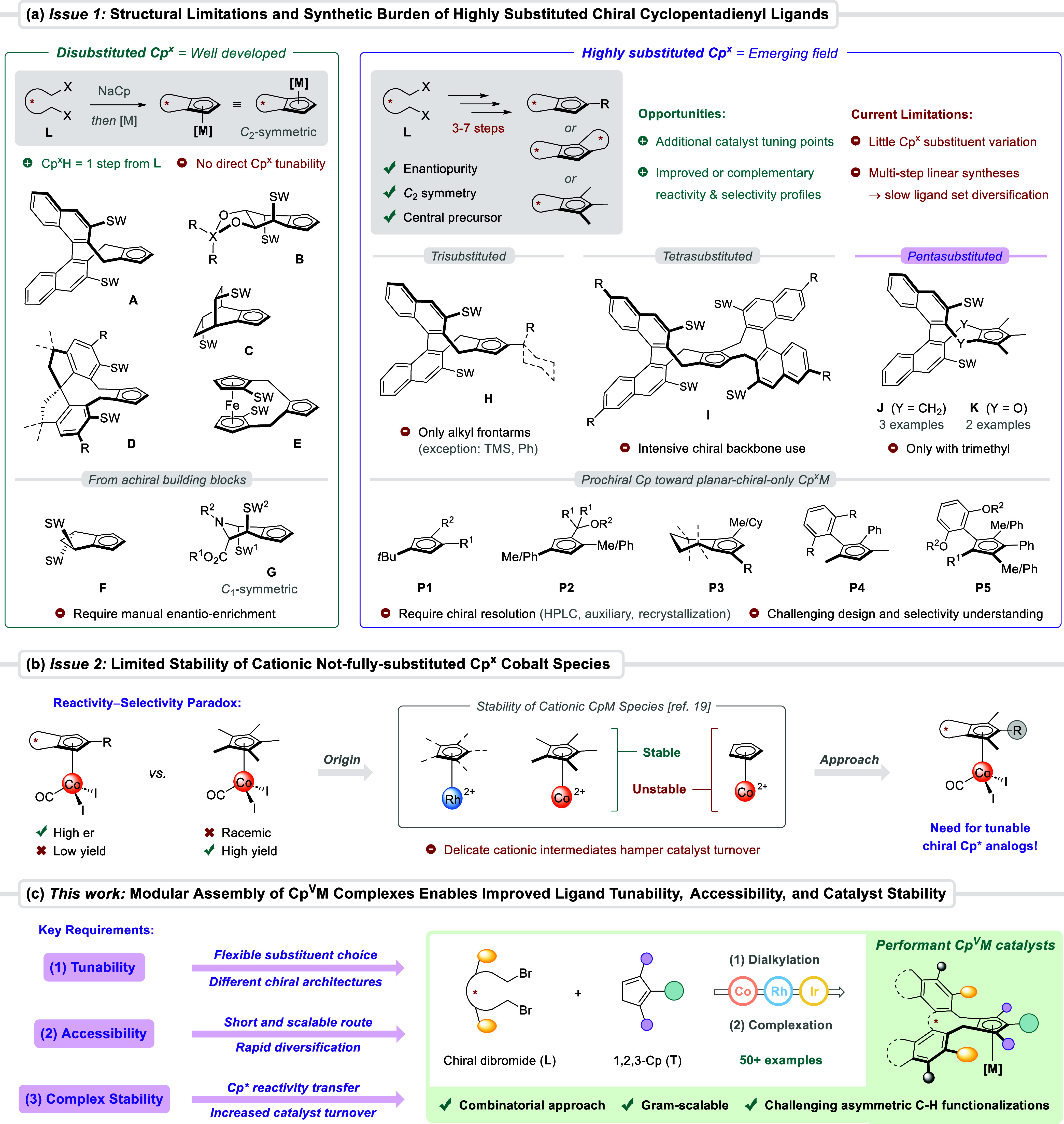
(a) Despite their promising catalytic advances, highly substituted Cp^x^ ligands remain underdeveloped compared to their disubstituted analogs, suffer from structural limitations, and require lengthy linear syntheses. (b) The limited lifetime of cationic partially substituted Cp^x^Co species hampers the development of new asymmetric C–H functionalizations. (c) This work: modular combinatorial synthesis of structurally diverse and catalytically performant pentasubstituted Cp^V^M complexes, offering improved ligand tunability, accessibility, and catalyst stability.

Overall, highly substituted Cp^x^ ligands **H**–**K** have allowed for an improved or complementary catalyst performance compared to their disubstituted analogs. Despite their promising catalytic advancements, they suffer from synthesis related drawbacks concerning accessibility, particular tunability, and an immature selectivity understanding. Their preparation poses a considerable synthetic burden in terms of time and resources, requiring 3–7 additional steps from central chiral precursors **L**, and allows for only few structural variations on the Cp^x^ ring. This gap in ligand availability and lack of diversity considerably hamper the development of novel enantioselective transformations. In addition, it is noteworthy that pentasubstituted Cp^x^ ligands remain vastly underexplored (**J**, **K**, **P5**) despite their strong structural resemblance to the omnipresent achiral Cp* ligand (C_5_Me_5_) and the potential benefits regarding clone-like catalytic behavior this could induce. *Therefore, a rapid modular and combinatorial synthetic strategy to expediently access pentasubstituted Cp^x^ligands with profound substituent flexibility is most desirable*.

So far, a large majority of Cp^x^-enabled asymmetric C–H functionalizations rely on rhodium.^1a,2a^ Yet, the global call for sustainability implores us to trade the use of precious noble metals for earth-abundant 3d metals, such as cobalt.^15^ Progress toward enantioselective Cp^x^Co­(III)-catalyzed processes remains rather slow and faces difficulties of catalyst efficiency (Figure 1b). In fact, almost all reported transformations rely on our trisubstituted Cp^x^ ligand design **H** ^13^ (except one,^16^ using **A**). The alternative approach is to pair achiral CpCo catalysts with a chiral acid additive,^2e^ ^,^ ^17^ but this strategy is restricted to transformations where the acid is involved in the enantiodetermining step. During the (un)­reported development of various reactions with Cp^x^Co catalysts, we noticed a trend between their reactivity and the degree of Cp substitution. Frequently, the observed yields and TONs are highest for the ubiquitous Cp* ligand. Behind are chiral trisubstituted Cp^x^ ligands **H**, in turn greatly outperforming their disubstituted analogs **A**. Such behavior is uncommon for rhodium, where disubstituted Cp^x^Rh catalysts generally show excellent reactivity.^2a^ We reasoned the inferior stability of non-noble cobalt complexes, and more specifically the attenuated Cp–Co bond strength of the cationic catalytic intermediates, to be a main contributor.^18^ Indeed, Maitlis reported that unsubstituted CpCo­(III) complexes readily disproportionate in coordinating solvents, whereas both the CpRh­(III) and pentasubstituted Cp*Co­(III) analogs are stable.^19^ As the remaining coordination sphere of cobalt likewise influences the stability of a CpCo species,^18–20^ the established Cp^x^Co-catalyzed methodologies require a favorable combination of substrates, bases, and solvents, together able to sufficiently stabilize delicate catalytic intermediates. As such, a dedicated optimization of reaction parameters has helped tremendously in the past to push the available Cp^x^Co complexes to their current limits. However, this approach started giving diminishing returns, and a paradigm shift is needed. As a general solution, we conclude that cobalt complexes with pentasubstituted Cp^x^ligands as chiral Cp* analogs would increase catalyst stability and allow for higher turnover.

Herein, we report the design and synthesis of structurally diverse pentasubstituted chiral cyclopentadienyl ligands (designated as Cp^V^) to address the discussed issues of ligand tunability, accessibility, and catalyst stability (Figure 1c). Our approach consists of a convergent and modular synthetic strategy that relies on the dialkylation of readily accessible 1,2,3-trisubstituted cyclopentadiene (1,2,3-Cp) building blocks **T** with various chiral *bis*-electrophiles **L**. The expedient combinatorial approach substantially reduces the required synthetic effort over the linear pathways of highly substituted Cp^x^ ligands **H–K**, is scalable, and allows for wide substituent flexibility. Complexation to group 9 metals is demonstrated, and Cp^V^Rh­(III) phosphite species enable facile electronic ligand parametrization. The Cp^V^ ligands act as full chiral Cp* clones since: (a) they tolerate simpler, faster, and high-yielding complexation procedures compared to established disubstituted Cp^x^ ligands; (b) their cobalt complexes display a substantially increased stability, allowing for the isolation of bench-stable cationic species; and (c) in catalysis, they exhibit similar efficiency as Cp* under identical reaction conditions, thus drastically reducing the required optimization efforts. We benchmark the Cp^V^ cobalt and rhodium complexes in exemplary selected asymmetric C–H functionalizations, where they outperform established Cp^x^ ligands regarding both yield and enantioselectivity, thus indicating a strong future potential.

## Results and Discussion

### Direct Cp^V^ Ligand Access by Dialkylation of 1,2,3-Cps

To reach our target of more accessible chiral Cp^V^ ligands, we selected a convergent synthetic approach. We recently disclosed a unified and diversity-oriented synthesis platform for the rapid preparation of 1,2,3-Cps **T**.^5^ These simple building blocks were designed to be directly used in a dialkylation strategy with chiral *bis*-electrophiles **L** granting expedited access to structurally diverse pentasubstituted Cp^V^ ligands in a single step.^10c^ This mix-and-match approach provides full control of the desired Cp^V^ substituents as well as the chiral backbone architecture at the last stage before complexation, thus largely improving the time and resource efficiency of ligand tuning. Of equal importance is the accessibility of precursors **T** and **L** on a multigram scale from inexpensive precursors through reliable and operationally straightforward procedures.^5,21^

In the pilot transformation between isopropyl-bearing **T1** and chiral dibromide **L1** (Scheme 1a), we found that potassium hydride smoothly enabled a serial deprotonation–alkylation of the 1,2,3-Cp substrate providing in 96% yield a mixture of **Cp** ^**V**^ **1** and spirodiene **S1** in a 56:44 ratio. Due to the [1,5]-H sigmatropic shifts, prevalent in many cyclopentadienes,^22^ pentasubstituted **Cp** ^**V**^ **1** was obtained as a complex mixture of double bond isomers, contrasting partially substituted Cps which usually have one dominant thermodynamically preferred isomer.^5,8a^ While complicating spectroscopic analysis and characterization, such isomerism is completely inconsequential for η^5^-coordinations as all isomers converge to a single anionic Cp^V^ ligand. The substrate-dependent **Cp** ^**V**^/**S** ratio was determined via qNMR of the spirodiene’s characteristic olefinic proton signal, and separation of the **Cp** ^**V**^ **1**/**S1** mixture was feasible, but not necessary for the subsequent complexation. As such, exposure of **Cp** ^**V**^ **1**/**S1** to dicobalt octacarbonyl and oxidation with iodine delivered Cp^V^Co­(III) complex **Co1a** in 65% yield (Scheme 1b). Its solid-state structure was unambiguously determined through X-ray crystallography. To verify that no enantio-erosion had occurred along the dialkylation–complexation sequence, we converted **Co1a** with trimethylphosphite to Cp^V^CoI_2_P­(OMe)_3_ adduct **Co1b**. This complex has a substantially increased stability on silica gel and, contrary to **Co1a**, was amenable to chiral HPLC analysis which confirmed full preservation of its enantiopurity.

**Scheme 1:**
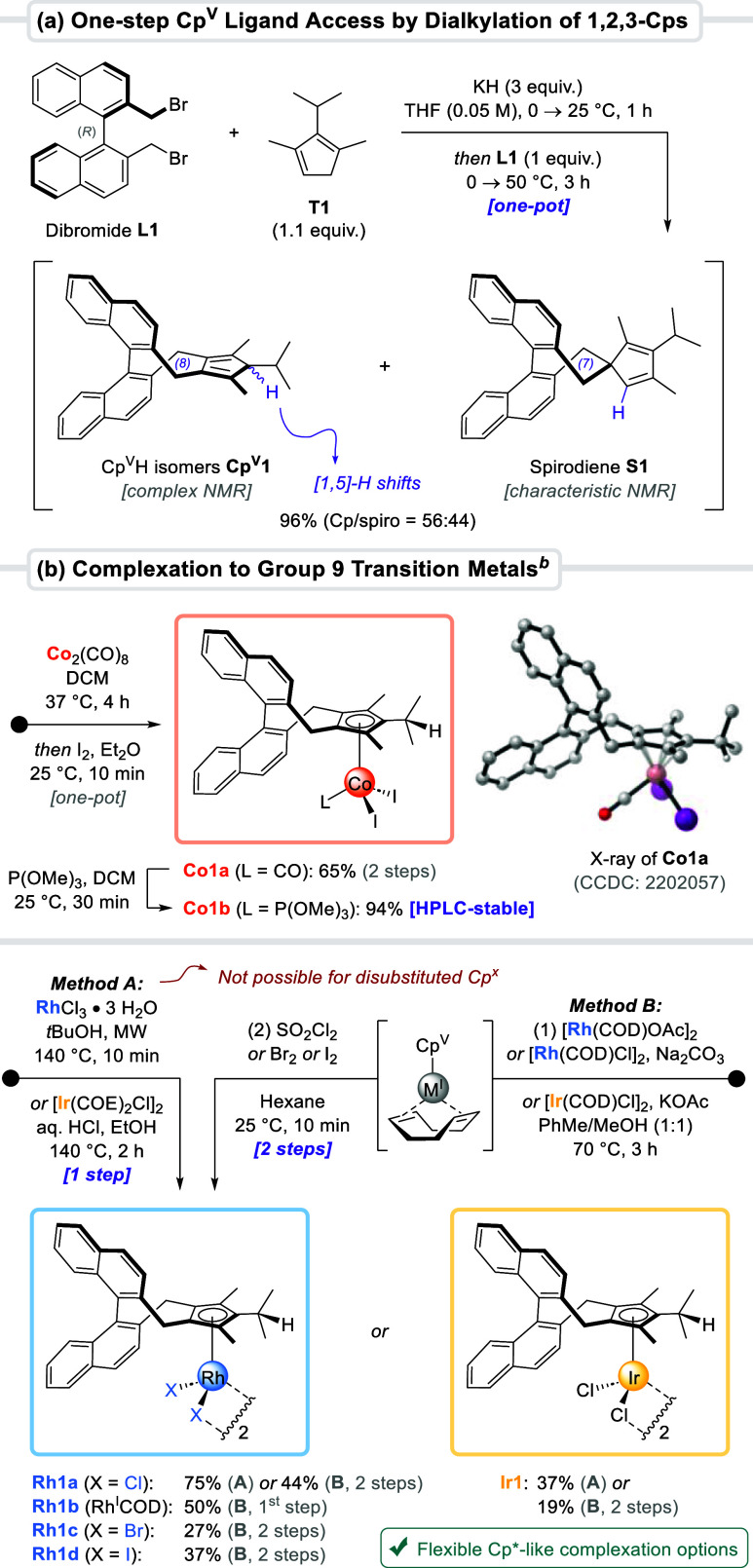
Modular Dialkylation Strategy Enables One-Step Cp^V^ Ligand Assembly from 1,2,3-Cp Precursors^a^

a Isolated yields.
b Stoichiometry based on the amount of **Cp** ^**V**^ **1** as determined by qNMR using 1,3,5-trimethoxybenzene or ethylene carbonate as an internal standard. The solid-state X-ray structure of (*R*_a_)-**Co1a** shows 50% probability thermal ellipsoids; hydrogen atoms (except for on the central *i*Pr carbon) are omitted for clarity. COE = cyclooctene.

Next, we investigated the complexation of **Cp** ^**V**^ **1** with rhodium and iridium. Typically, disubstituted Cp^x^ ligands are complexed by a mild two-step M­(I)-complexation/oxidation sequence (M = Rh, Ir) to access catalytically active Cp^x^M­(III) complexes.^3i,8b,11a,11b,23^ A direct M­(III)-metalation procedure, employing RhCl_3_·3H_2_O for example, is operationally simpler.^10a^ ^,^ ^24^ However, only highly substituted Cp^x^ ligands tolerate the rather harsh reaction conditions. Fulfilling this requirement, pentasubstituted **Cp** ^**V**^ **1** was exposed for 10 min to rhodium trichloride in *t*BuOH at 140 °C, yielding Cp^V^Rh­(III) complex **Rh1a** in 75% yield (Scheme 1b, bottom). The two-step protocol using [Rh­(COD)­OAc]_2_ followed by oxidation with sulfuryl chloride provided **Rh1a** in 44% yield. Yet, this sequential approach allowed preparation of the more delicate bromo (**Rh1c**) and iodo analogs (**Rh1d**) as well as isolation of rhodium­(I) complex **Rh1b**. Regarding iridium, the complexation efficiency followed a similar trend. Compared to the two-step synthesis of **Ir1** in 19% yield, a direct metalation protocol using [Ir­(COE)_2_Cl]_2_ paired with aqueous HCl provided 37% yield.^25^ In sum, the prepared Cp^V^ ligands mimic the robustness and flexibility of achiral Cp* to different complexation methods, thus broadening possibilities for their future coordination to other catalytically relevant transition metals.

### Expedient Assembly of a Structurally Diverse Cp^V^ Metal Catalyst Library

With the feasibility of a combinatorial Cp^V^ ligand synthesis demonstrated, we then sought to establish the scope of the dialkylation–complexation process (Scheme 2). Leveraging our modular strategy, we built a structurally diverse catalyst library by connecting a set of symmetrical 1,2,3-Cps **T1–15** to both binaphthyl- (**L1–5**) and spirobiindanyl-based (**L6–10**) chiral dibromide precursors. Ligands **Cp** ^**V**^ **1–5** containing primary, secondary, and tertiary alkyl frontarms were prepared in good to excellent yields (73–99%). The Cp^V^H/spirodiene ratio decreased with increasing size of the frontarm, ranging from 94:6 for methyl (**Cp** ^**V**^ **3**) to 51:49 for *tert*-butyl (**Cp** ^**V**^ **5**). Notably, trimethyl-bearing ligand **Cp** ^**V**^ **3** has previously proven effective for the Rh­(III)-catalyzed C–H annulation toward enantio-enriched isoindolinones,^10a^ but required a lengthy 7-step sequence from dibromide **L1** with 27% overall yield. In stark contrast, the present methodology allows for a significant improvement in time and resource efficiency by directly accessing **Cp** ^**V**^ **3** from **L1** in a single step with 90% yield of the desired Cp^V^H isomers. Here, this ligand was complexed to cobalt­(III) to yield **Co2** in 42% yield. For the first time, *C*_2_-symmetric Cp^x^ ligands that bear functionalized arenes could be accessed, enabling the incorporation of bulky 3,5-*t*Bu- (**Cp** ^**V**^ **6**), electron-poor 3,5-CF_3_- (**Cp** ^**V**^ **7**), or electron-rich 4-OMe-substituted (**Cp** ^**V**^ **8**) phenyl frontarms. Subsequent η^5^-coordination to cobalt and rhodium provided complexes **Co4–5** and **Rh4–6**. Sterically hindered **Cp** ^**V**^ **9** with its *ortho*-difunctionalized frontarm was equally accessible. However, Co_2_(CO)_8_ could not succeed in its metalation, and unreacted **Cp** ^**V**^ **9** was partially recovered. On the other hand, treatment with RhCl_3_·3H_2_O delivered **Rh7** in an excellent 93% yield. A similar observation was made for *t*Bu-substituted ligand **Cp** ^**V**^ **5**. This difference in complexation ability likely stems from cobalt’s higher sensitivity to steric repulsions,^18^ which is in agreement with the smaller atomic radius and shorter Cp-metal bond lengths compared to its 4d-congener.^26^

**Scheme 2:**
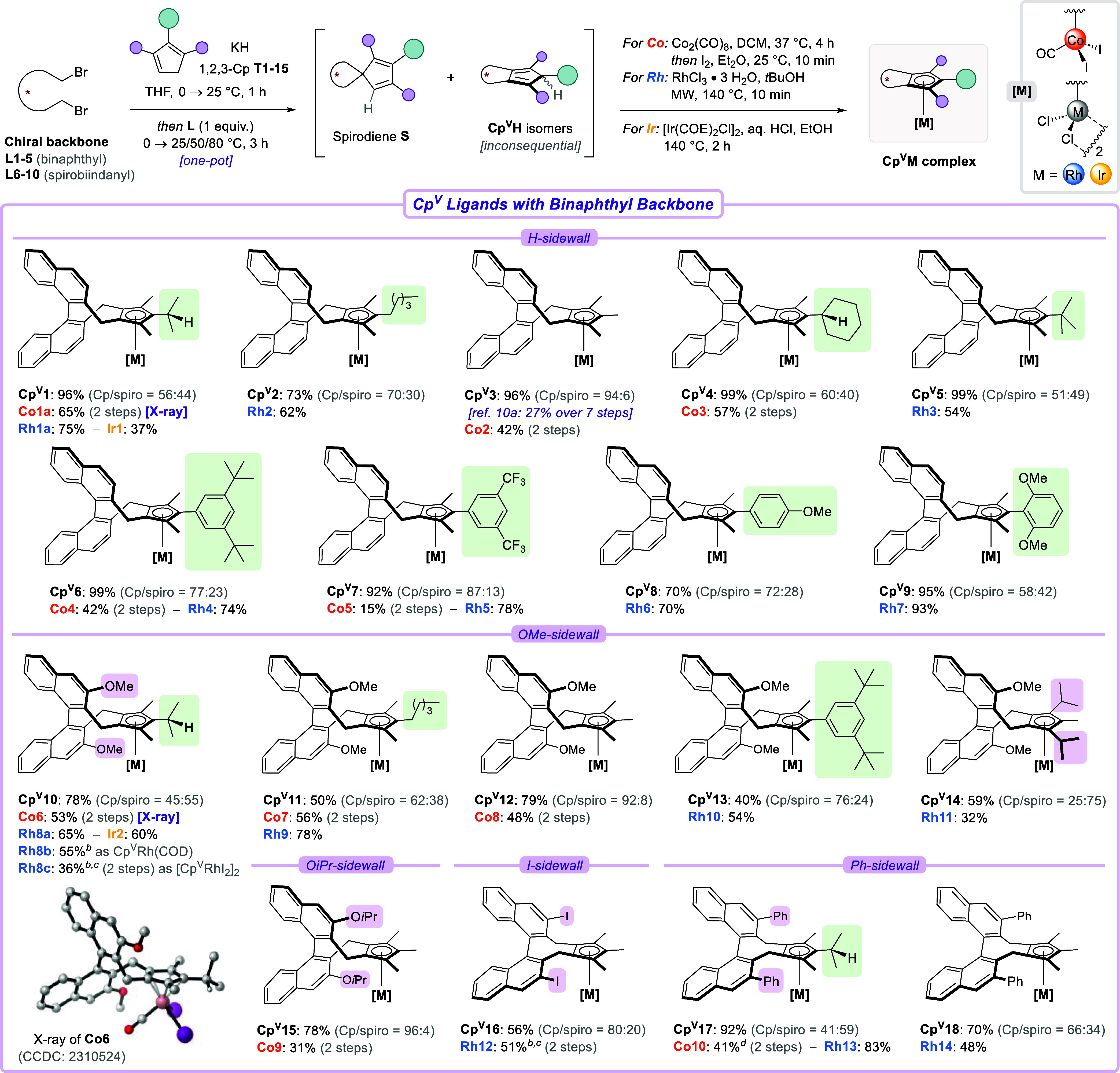
Scope of Cp^V^ Ligands and their Metal Complexes Containing a Chiral Binaphthyl Backbone^a^

a Isolated yields. The solid-state X-ray structure of (*R*_a_)-**Co6** shows 50% probability thermal ellipsoids; hydrogen atoms (except for on the central *i*Pr carbon) and eventual disordering are omitted for clarity.
b Complexation with [Rh­(COD)­OAc]_2_.
c Oxidation with I_2_ (**Rh8c**) or SO_2_Cl_2_ (**Rh12**).
d As (*R*_a_)-**Co10** enantiomer.

Next, additional binaphthyl-derived backbones were investigated (Scheme 2, bottom). In the case of OMe-sidewalls, the dialkylation of **T1** delivered ligand **Cp** ^**V**^ **10** in 78% yield. Subsequent metalation to cobalt (**Co6**, 53%), rhodium (**Rh8a**, 65%), and iridium (**Ir2**, 60%) succeeded uneventfully. Applying the two-step complexation protocol let us isolate rhodium­(I) species **Rh8b** in 55% yield, which was then oxidized with iodine to form **Rh8c**. Other 1,2,3-Cps bearing alkyl or aryl frontarms could be installed as well (**Cp** ^**V**^ **11–13**). Furthermore, we were pleased to isolate diisopropyl-substituted ligand **Cp** ^**V**^ **14** en route to sterically unique complex **Rh11**. The presence of O*i*Pr- (**Cp** ^**V**^ **15**), iodo- (**Cp** ^**V**^ **16**), and Ph-sidewalls (**Cp** ^**V**^ **17–18**) on the chiral dibromide was tolerated, thereafter affording complexes **Co9–10** and **Rh12–14**.

To further illustrate the flexibility of the Cp^V^ synthesis, we then examined chiral *bis*-electrophiles having a spirocyclic architecture (Scheme 3). Dialkylations smoothly provided ligands **Cp** ^**V**^ **19–24**. Their subsequent complexations were successful, thus again underlining the facile access to a broad panel of alkyl and aryl frontarms, including preparation of trimethoxybenzene-substituted **Rh19** in 93% yield. After the chlorides from **Rh20a** were scavenged with AgBF_4_ in acetonitrile, a single crystal of the resulting cationic complex **Rh20b** allowed unambiguous X-ray-mediated confirmation of its ligand architecture. The chiral pocket could be visualized by a steric heatmap, of which the high buried volume (*V*_bur_ = 57.0%) exemplifies the bulk of **Cp** ^**V**^ **24**. The ligand’s OMe/Me-sidewalls occupy the northwestern quadrant, whereas the prominent steric influence of the biphenyl frontarm is well visible in the east. Next, we evaluated other structural variations of the spirocyclic backbone and were pleased to find that OMe/H- (**Cp** ^**V**^ **25**), O*i*Pr/H- (**Cp** ^**V**^ **26**), and bulky OMe/*t*Bu-sidewalls (**Cp** ^**V**^ **27**) were all tolerated and provided the corresponding rhodium complexes **Rh21–23** in good yields (81–85%). Lastly, a simplified backbone architecture without sidewalls (**L10**) was connected to a range of 1,2,3-Cps to afford ligands **Cp** ^**V**^ **28–35** in high yields (76–99%). In turn, they were complexed with cobalt (**Co12–13a**), rhodium (**Rh24–30**), and iridium (**Ir3**). Note that all Co­(III), Rh­(III), and Ir­(III) complexes obtained in this campaign (50+ examples) were stable under ambient conditions and could be conveniently isolated by simple purification through a short plug of silica.

**Scheme 3:**
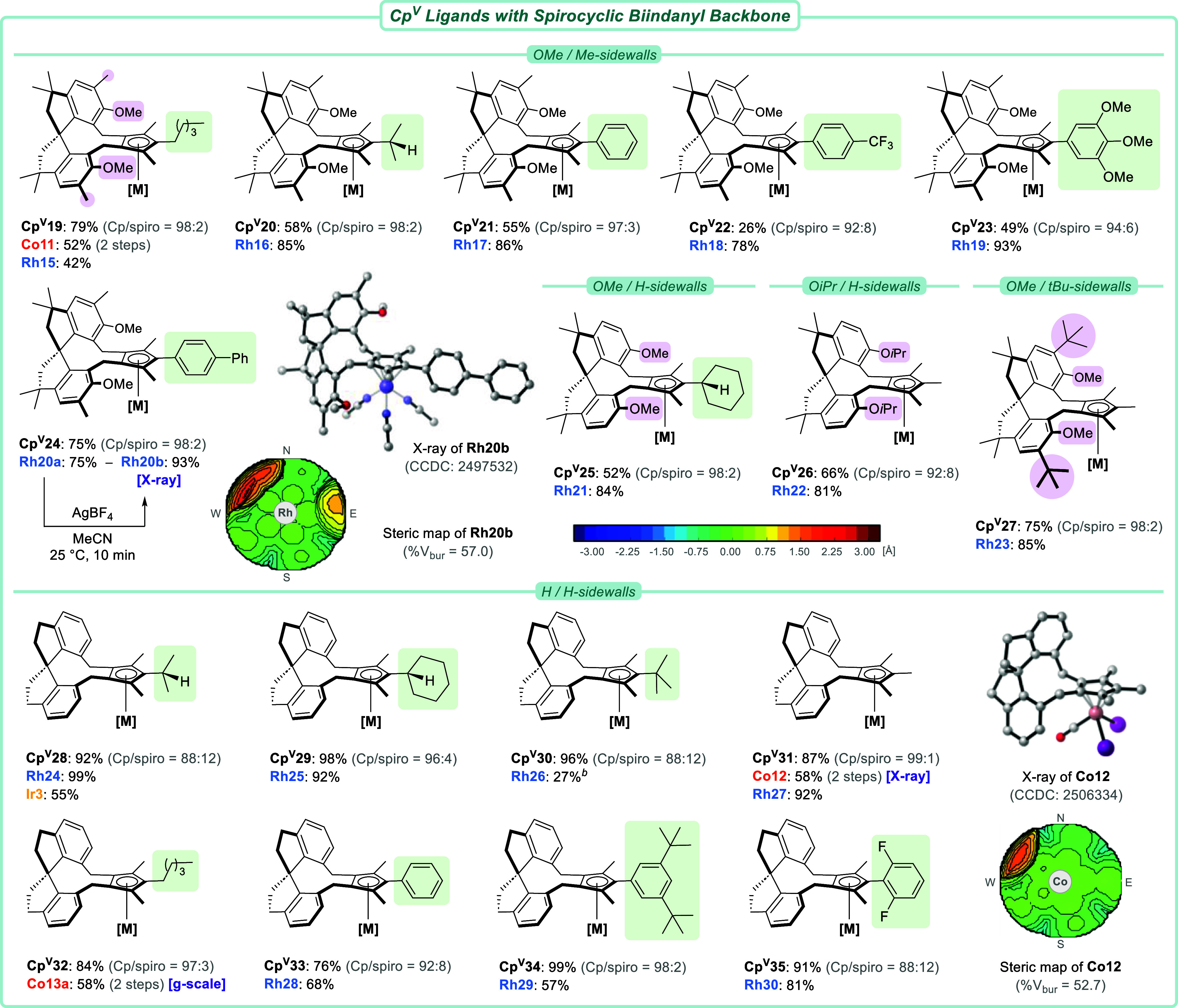
Scope of Cp^V^ Ligands and Their Metal Complexes Containing a Chiral Spirobiindanyl Backbone^a^

a Isolated yields. For reaction conditions, see Scheme 2. The solid-state X-ray structures of (*R*)-**Rh20b** and (*R*)-**Co12** show 50% probability thermal ellipsoids; hydrogen atoms and counterions are omitted for clarity. Steric maps around the metal center were generated using the SambVca 2.1 tool^27^ (with 3.5 Å sphere radius, 0.1 Å mesh spacing, and Bondi radii scaled by 1.17).
b At 80 °C.

During chiral backbone installation by the dialkylation procedure, the formation of the fused 9-membered ring in spirobiindanyl-based ligands **Cp** ^**V**^ **19–35** turned out to be massively favored over their respective 8-ring spirodiene homologs (avg. 95:5 ratio). In contrast, dialkylations with the binaphthyl-based systems showed a less pronounced preference for 8-ring cyclopentadienes **Cp** ^**V**^ **1–18** over 7-ring spirodienes **S1–18** (avg. 67:33 ratio). Initially, we anticipated that these spirodienes were innocent under all the employed complexation conditions and that solely their Cp^V^H structural isomers would react. However, upon exposure of a mixture of **Cp** ^**V**^ **1**/**S1** (56:44 ratio) to an excess of RhCl_3_·3H_2_O (with respect to the content of **Cp** ^**V**^ **1**), rhodium­(III) complex **Rh1a** was obtained in a seemingly impossible 134% yield (89% based on Rh, Scheme S20). To investigate the behavior of spirodiene **S1** under the applied conditions, isolated **S1** was separately subjected to Rh­(III) complexation conditions (RhCl_3_·3H_2_O, *t*BuOH, 140 °C, 10 min). Intriguingly, **Rh1a** was formed and could be isolated in 60% yield (Scheme 4a). *This suggests the occurrence of a unique rhodium­(III)-mediated tandem [1,5]-alkyl shift-complexation*. Such cascade reactivity is very rare and has, to the best of our knowledge, only been reported for smaller [4.4] spirocyclic systems using low-valent metal(0) carbonyl complexes (Cr, W, Fe, Co) in 2–21% yield.^28^

**Scheme 4:**
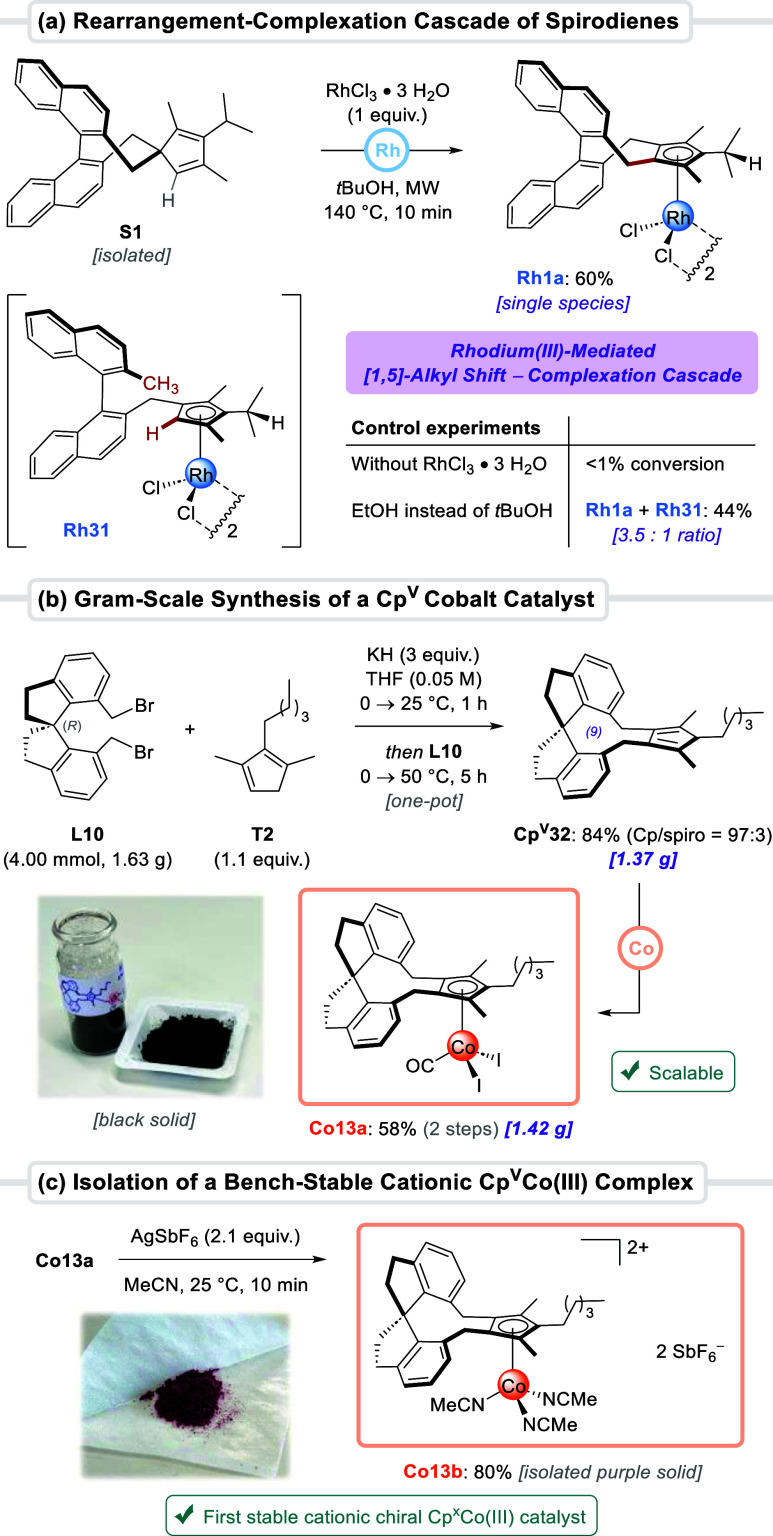
Rh-Mediated [1,5]-Alkyl Shift-Complexation Cascade, Gram-Scale Synthesis of a Cp^V^ Cobalt Catalyst, and a Bench-Stable Cationic Cp^V^Co­(III) Complex^a^

a Isolated yields. For complexation to cobalt: Co_2_(CO)_8_, then I_2_.

Control experiments confirmed that spirodiene **S1** is completely inert in the absence of rhodium trichloride and does not rearrange at 140 °C to form cyclopentadiene **Cp** ^**V**^ **1**. Furthermore, when ethanol solvent was used instead of *tert*-butanol, an inseparable mixture of two Rh­(III) complexes **Rh1a**/**Rh31** (3.5:1 ratio) was obtained. The minor species was identified as tetrasubstituted **Rh31**. Based on these results, we propose that the mechanism of the rearrangement–complexation cascade involves Rh­(III) first behaving as a Lewis acid and coordinating to the spirodiene moiety, which then enables a retro-Friedel–Crafts reaction generating a benzylic carbocation that is stabilized by the polar solvent. This cation could undergo forward Friedel–Crafts alkylation toward the pentasubstituted cyclopentadiene, which is directly followed by complexation. Speculatively, the formation of tetrasubstituted **Rh31** in ethanol is the result of the benzylic cation intermediate getting reduced by an in situ formed rhodium hydride species. Oxidation of ethanol to acetaldehyde through β-hydride elimination is indeed reported to generate such species,^7a^ ^,^ ^29^ explaining as well the absence of **Rh31** when conducting the reaction in *tert*-butanol. Besides its mechanistic interest, the rearrangement–complexation cascade aids the efficiency of Cp^V^Rh catalyst synthesis, with both dialkylation products converting to a single complex.

To exemplify the robustness of our convergent strategy, we completed a gram-scale dialkylation reaction (Scheme 4b). Spirobiindanyl-based dibromide (*R*)-**L10** (4.00 mmol) was exposed to 1,2,3-Cp **T2** in the presence of KH to deliver pentasubstituted **Cp** ^**V**^ **32** in 84% yield (1.37 g). Next, complexation using Co_2_(CO)_8_ and then I_2_ delivered 1.42 g (58% yield) of catalyst **Co13a**. Notably, complexes **Co11–13** represent, to the best of our knowledge, the first examples of Cp^x^Co­(III) catalysts containing a spirocyclic chiral backbone.^13a,13c^ Upon treatment with AgSbF_6_ in acetonitrile, **Co13a** was transformed into dicationic cobalt­(III) complex **Co13b**, isolated as a bench-stable purple solid in 80% yield (Scheme 4c). In contrast, attempting a similar reaction with cobalt complexes bearing di- or trisubstituted Cp^x^ ligands (types **A** and **H**) caused rapid decomposition. These findings support our initial hypothesis regarding the importance of highly substituted Cps for an increased Cp–Co bond strength in catalytically relevant cationic species. To the best of our knowledge, **Co13b** constitutes the first report of a stable dicationic chiral Cp^x^Co­(III) precatalyst. Such isolatable complexes are significant for transformations not amenable to in situ ionization with silver salts.

### Electronic Parametrization of Cp^V^ Rhodium­(III) Complexes by ^31^P NMR

Evaluation of the stereoelectronic environment of the newly obtained Cp^V^ ligands was subsequently performed (Figure 2). Such ligand parametrization aids in developing predictive models for catalyst optimization.^6,14e,30^ Moreover, it provides us a better and quantitative understanding of the different electronic effects regarding sidewall, backwall, and frontarm tuning. In that respect, dimeric [Cp^V^RhCl_2_]_2_ complexes were converted to their respective Cp^V^RhCl_2_P­(OEt)_3_ adducts **Rh′**. Subsequently, the chemical shift of the phosphorus nucleus (δ_P_) as well as its coupling constant with ^103^Rh (*J*_Rh–P_) was collected by ^31^P NMR. We previously established the latter to be the more reliable proxy for estimating Cp electronics and observed that more electron-deficient Cps result in lower *J*_Rh–P_ values.^5^ As such, decreasing the degree of Cp substitution was also reflected by this coupling constant, for instance when going from penta- (Cp*Rh′, 215.4 Hz) over tetra- (**Cp** ^**Me4**^ **Rh′**, 212.3 Hz) to trisubstituted achiral CpRh complexes (**Cp** ^**Me3**^ **Rh′**, 208.8 Hz). Likewise, the novel pentasubstituted Cp^V^ ligand of **Rh1a′** (216.2 Hz) rendered the chiral complex noticeably more electron-rich compared to its established trisubstituted analog **Rh** ^**tri**^ **1′** (211.8 Hz). Altering the ligand sidewalls, for instance, to phenyl groups (**Rh13′**, 213.1 Hz), also had an influence on the Cp^V^ electronics. Replacing the binaphthyl backbone of **Rh1a′** for a spirobiindanyl structure resulted in a more electron-rich complex (**Rh24′**, 218.8 Hz). Importantly, the strongest electronic influence was observed by modifications of the ligand frontarm. For different functionalized arene frontarms (**Rh4–7′**), coupling constants ranged from 211.7 Hz in the case of electron-withdrawing 3,5-CF_3_ groups (**Rh5′**) to 218.4 Hz for 2,6-OMe substitution (**Rh7′**). Interestingly, this suggests that pentasubstituted **Rh5′** is similar to trisubstituted chiral complexes **Rh** ^**tri**^ **1–2′** on an electronic base, whereas **Rh7′** outcompetes Cp*Rh′ in electron-richness. Overall, these results illustrate the broad electronic tunability that the combinatorial synthesis of Cp^V^ ligands offers on top of their evident steric differences, thus providing another layer of rational catalyst tuning and optimization.

**Figure 2:**
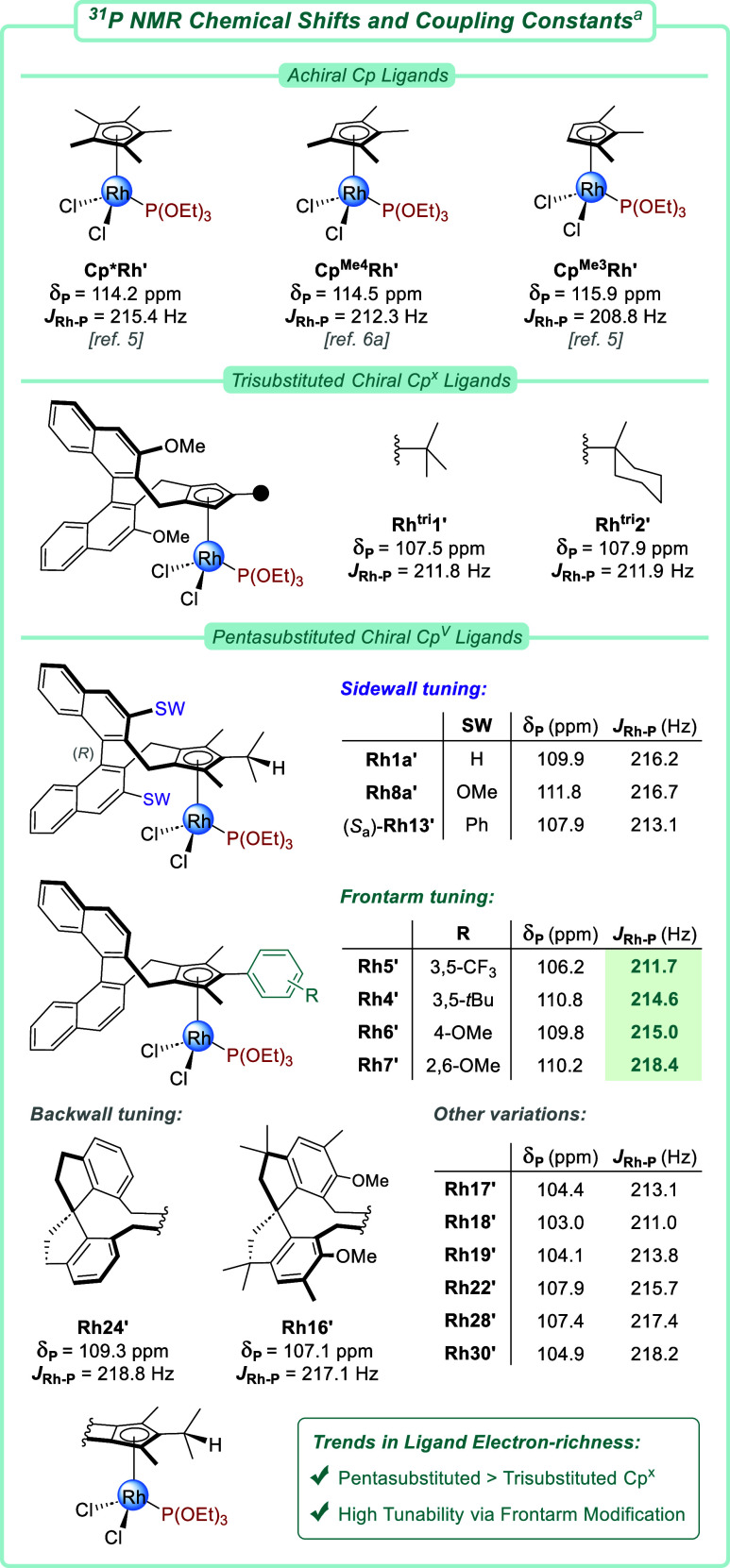
Evaluation of the stereoelectronic environment of the Rh­(III) center in chiral Cp^x^Rh phosphite adducts by ^31^P NMR. ^*a*^ Measured in CD_2_Cl_2_ at 162 MHz.

### Selected Catalytic Performance of Cp^V^M Complexes in Challenging Asymmetric C–H Functionalizations

Gauging the catalytic competence of newly accessible pentasubstituted Cp^V^ metal complexes in challenging enantioselective C–H functionalizations was performed next. The primal objective was to showcase a strong response in reaction outcome (i.e., yield and selectivity levels) to a change in the Cp^V^ substituents, which are fast to accommodate and easy to implement via the outlined modular approach. Particularly, we refrained from extensive optimization rounds of specific transformations and aimed to illustrate the bright future potential of Cp^V^-based asymmetric transition-metal catalysis.

First, we sought to assess whether the catalytic abilities of pentasubstituted Cp^V^ ligands are competitive with classical disubstituted Cp^x^ (e.g., type **A**). For this purpose, we chose as a benchmark transformation the recent Rh­(III)-catalyzed allylic C–H amination protocol by Wang,^31^ providing valuable enantio-enriched allylic amines from unactivated alkenes (Scheme 5). The reaction of allylbenzene **1a** with sulfonamide **2a** was reported to deliver branched amine (−)-**3a** in 82% yield and a 94.5:5.5 enantiomeric ratio using disubstituted catalyst **Rh** ^**di**^ **1** with Ph-sidewalls. Under the reported conditions, we screened a subset of the prepared Cp^V^Rh­(III) complexes. Interestingly, trimethyl-bearing catalyst (*S*_a_)-**Rh14**, which is a pentasubstituted analog of (*R*_a_)-**Rh** ^**di**^ **1**, resulted in an improved yield but diminished selectivity (94%, 25:75 er). Increasing the size of the frontarm to isopropyl (**Rh13**) had a significant but detrimental impact, as it delivered a nearly racemic product. The powerful response of the stereoselectivity to frontarm tuning became especially apparent for Cp^V^ ligands having no binaphthyl sidewall. A full enantio-inversion was observed when replacing the 4-methoxyphenyl substituent in **Rh6** (23:77 er) by a 2,6-dimethoxyphenyl group (**Rh7**, 80:20 er).

**Scheme 5:**
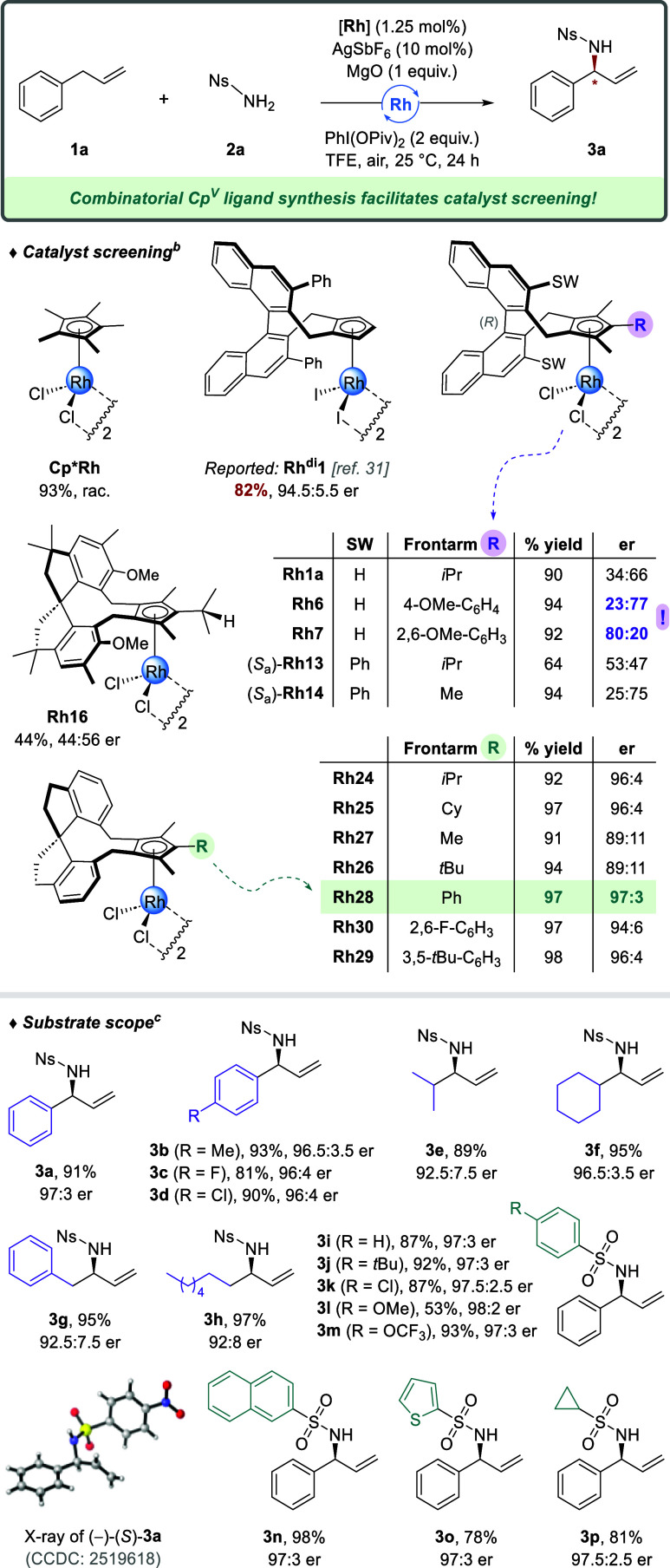
Benchmark Performance of Cp^V^ Ligands in the Allylic C–H Amination of Unactivated Alkenes^a^

a Branched amination products were obtained exclusively. Ns = 4-nitrobenzenesulfonyl.
b Yields determined by qNMR with 1,3,5-trimethoxybenzene as an internal standard.
c Isolated yields, using **Rh28**. The solid-state X-ray structure of (*S*)-**3a** shows 50% probability thermal ellipsoids.

We found spirobiindanyl-based Cp^V^ ligands to be favored for this asymmetric C–H amination, and **Rh24** with a simple unsubstituted backbone and *i*Pr-frontarm outperformed the literature, delivering allylic amine **3a** in 92% yield and 96:4 er. Here, our combinatorial ligand synthesis allowed a rapid screening of different Cp^V^ substituents. In that respect, installing a methyl (**Rh27**) or *tert*-butyl frontarm (**Rh26**) gave no improvement, but catalyst **Rh28** bearing a phenyl group provided an excellent 97% yield and 97:3 er. Permutations of the aromatic ring with 3,5-*t*Bu (**Rh29**) or 2,6-difluoro substituents (**Rh30**) did not enhance the catalyst performance. Notably, the presence of OMe/Me-sidewalls on the ligand (**Rh16**) was detrimental for both the yield and the enantioselectivity. A single crystal of **3a** allowed X-ray-mediated determination of its absolute configuration as (*S*.). Note that the reported disubstituted catalyst **Rh** ^**di**^ **1** provided the same (−)-(*S*)-**3a** enantiomer despite displaying an inversed ligand orientation in the chiral pocket compared to **Rh28**. This observation indicates that chiral induction by pentasubstituted Cp^V^ ligands is the result of a steerable interplay between the backwall’s innate chirality and the Cp^V^ ring’s planar chirality.

With **Rh28** as the best performing catalyst, we briefly investigated its generality for other substrates (Scheme 5, bottom). Substituted allylbenzenes were aminated in good yields and selectivities (**3a**–**d**, 81–93%, ≥96:4 er). Switching the phenyl group in **1a** for isopropyl (**3e**), cyclohexyl (**3f**), benzyl (**3g**), or heptyl (**3h**) was also tolerated. Furthermore, the presence of an electron-poor *N*-nosyl substituent on the amination reagent proved not essential since a diverse set of aryl (**3i**–**n**), heteroaryl (**3o**), and alkyl (**3p**) sulfonamides were well tolerated while maintaining the high enantioselectivity (≥97:3 er).

In most cases, the development of new enantioselective C–H functionalizations requires a two-step process: (1) the identification of a hit catalyst via ligand screening, and (2) the optimization of yield and er values by a more or less tedious fine-tuning of the reaction conditions. The required time and, consequently, resources to complete both processes can vary widely per case. In this respect, our Cp^V^ platform aims to fundamentally aid the efficiency of both steps. The mix-and-match Cp^V^ synthesis not only facilitates the ligand screening step (as established in Scheme 5) but also has the capability to greatly simplify the optimization process. Designed as true chiral Cp* analogs, we hypothesized pentasubstituted Cp^V^-based catalysts to exhibit a clone-like reactivity to their omnipresent achiral counterpart in transformations where classical disubstituted Cp^x^ ligands struggle.

To support this notion, we investigated the dearomative (3 + 2) C–H spiroannulation of 2-alkenylphenol **4** with alkyne **5** (Scheme 6).^32^ You’s development of an enantioselective version using disubstituted **Rh** ^**di**^ **2** was challenged by the poor reactivity of this catalyst compared to achiral Cp*Rh.^33^ Even after careful optimization of the reaction conditions (including solvent mixtures, inert atmosphere, temperature, and time), spirocycle (+)-**6** was obtained in moderate yield and enantioselectivity (51%, 69:31 er). In contrast, directly employing our pentasubstituted catalyst **Rh9** under the simpler and identical conditions as those for Cp*Rh (MeCN, air, 40 °C) provided an excellent 96% yield with a similar level of enantio-induction. Such a direct translation of reaction conditions simplifies and accelerates the development of new enantioselective transformations from racemic precedents.

**Scheme 6:**
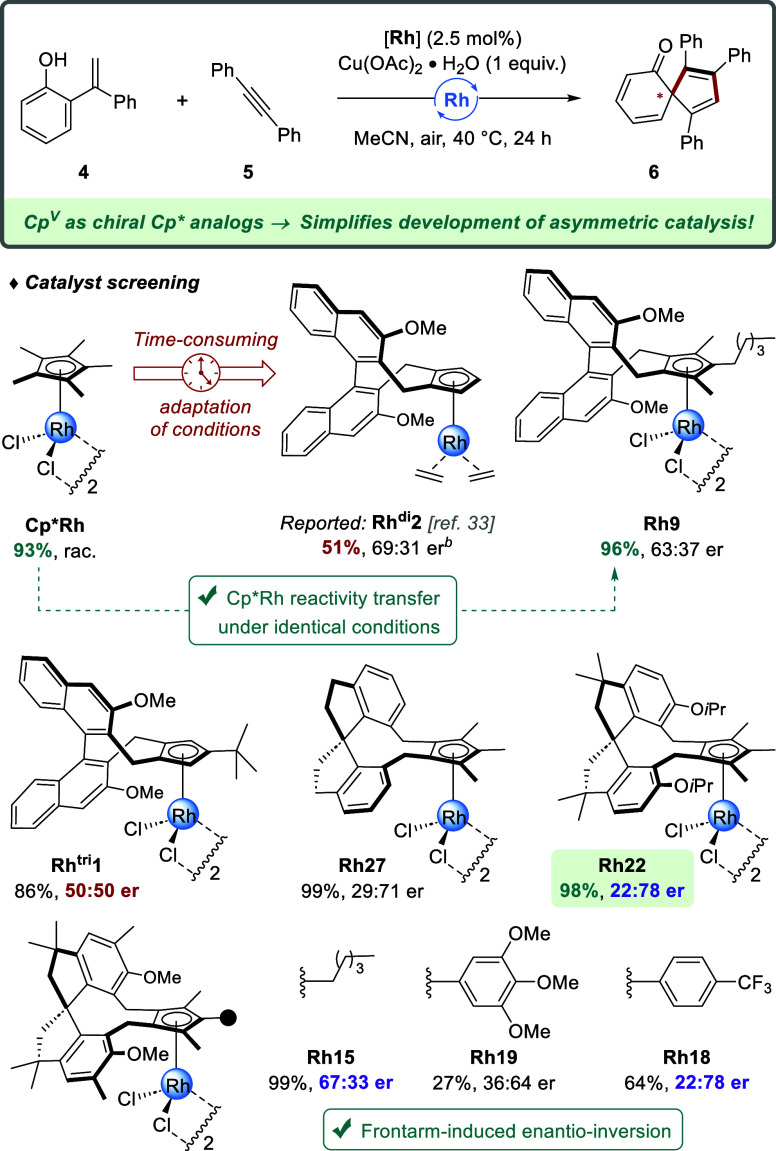
Cp^V^ as Chiral Cp* Analogs: Direct Reactivity Transfer and Strong Selectivity Response to Frontarm Tuning Facilitate Asymmetric Catalysis Development^a^

a Yields determined by qNMR with ethylene carbonate as an internal standard.
b Conditions for **Rh** ^**di**^ **2**: Cu­(OAc)_2_ (2 equiv), DMF/*t*AmylOH (1:1), Ar, 60 °C, 44 h.^33^

We briefly investigated other Cp^V^Rh complexes in this system. Modifying the backwall architecture maintained high reactivity while enhancing enantioselectivity (**Rh27**, 99%, 29:71 er), which was further improved by installing sidewalls (**Rh22**, 98%, 22:78 er). We again observed a large response of both yield and er readouts to subtle Cp^V^ substitution changes. For instance, replacing the central pentyl group of catalyst **Rh15** (99%, 67:33 er) for an arene group resulted in an inversion of the product’s major enantiomer. Tuning the electronic nature of this arene frontarm also strongly influenced catalytic performance as evidenced by comparing electron-rich **Rh19** (27%, 36:64 er) with electron-poor **Rh18** (64%, 22:78 er). Noteworthily, as a comparison, established trisubstituted Cp^x^Rh­(III) catalyst **Rh** ^**tri**^ **1** provided **6** in a completely racemic fashion. Overall, these findings underline the potential of Cp^V^ ligands to leverage their analogy to Cp* for tackling performance issues in challenging asymmetric C–H functionalizations. Catalyst **Rh22** outperformed the literature regarding both yield and enantio-induction, while the preliminary ligand screening provides a clear direction for further improved catalyst design.

With respect to Cp^x^ cobalt catalysis, the critical-to-achieve high reactivity has especially been a difficult and longstanding challenge. The existing enantioselective transformations almost exclusively rely on trisubstituted ligands **H** with the disubstituted variant **A** usually providing vastly inferior yields.^13^ Moreover, when developing new Cp^x^Co­(III)-catalyzed reactions, we often find that ligands **H** are unable to match the efficiency and reactivity of achiral Cp*Co­(CO)­I_2_. A careful and time-consuming optimization of reaction conditions helped to improve their performance on reported transformations but failed on others. We hypothesized that the attenuated stability of the cationic catalytic intermediates bearing partially substituted Cp ligands is responsible (Figure 1b).^18,19^ Chiral pentasubstituted Cp^V^ ligands mimic the degree of substitution of Cp*. As such, we expect that they will provide a more general solution for the Cp^x^Co stability issues, which in turn will translate into an increased catalyst turnover and thus higher yields. The isolation of bench-stable cationic **Co13b** clearly supports the first part of this hypothesis.

To assess the catalytic turnover of Cp^V^Co­(III) complexes, we selected the (3 + 2) C–H spiroannulation of *N*-sulfonyl ketimine **7** toward benzosultam **8** (Scheme 7).^34^ This transformation has not yet been achieved by asymmetric cobalt catalysis. Screening the established di- (**Co** ^**di**^ **1**) and trisubstituted (**Co** ^**tri**^ **1**) chiral complexes confirmed their poor reactivity (6–25% yield) in contrast to the excellent performance of achiral Cp*Co (97% yield). As such, we were pleased to find that pentasubstituted analog **Co8** directly displayed a substantially higher reactivity, furnishing spirocycle (+)-**8** in 94% yield and with an improved enantioselectivity of 90:10 er. Such a level of stereocontrol at 120 °C is noteworthy since enantioselective CpCo-catalyzed processes usually require mild temperatures,^2e^ and examples above 50 °C are relatively rare.^13d,16,35^ Spirobiindanyl-based catalyst **Co12** with its simple unsubstituted backbone also provided a high yield and good level of enantio-induction (93%, 15:85 er).

**Scheme 7:**
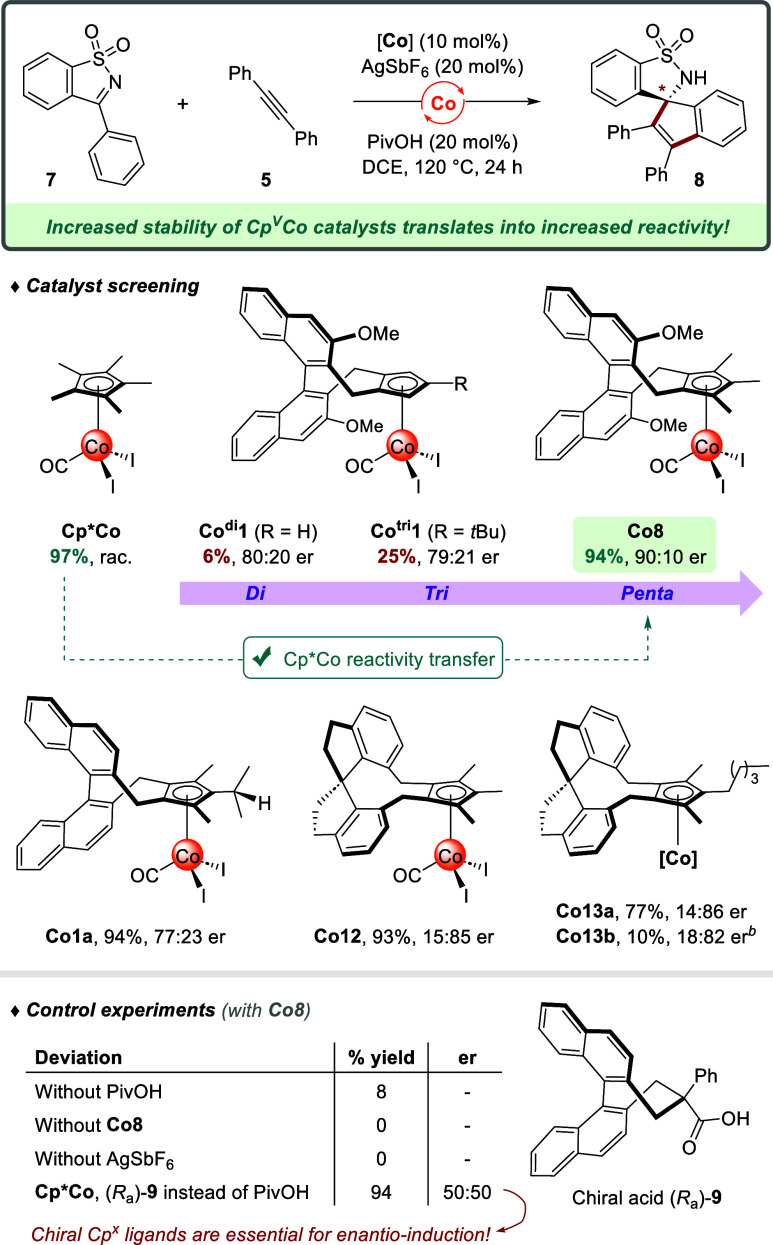
Enhanced Stability of Pentasubstituted Cp^V^ Cobalt Complexes Increases Their Catalytic Reactivity^a^

a Yields determined by qNMR with 1,3,5-trimethoxybenzene as an internal standard.
b Without AgSbF_6_.

Control experiments illustrated that both AgSbF_6_ and PivOH additives are essential, as little to no reaction occurred in the absence of either component. Surprisingly, this remained the case for cationic precatalyst **Co13b**, which resulted in a strongly diminished yield and enantioselectivity (10%, 18:82 er) compared to the combination of complex **Co13a** with AgSbF_6_ (77%, 14:86 er). This result indicates that the role of silver in this transformation goes beyond simply acting as a halide scavenger. When achiral catalyst Cp*Co was paired with Matsunaga’s chiral carboxylic acid (*R*_a_)-**9** in lieu of pivalic acid,^36^ no enantio-induction was achieved. This outcome underscores the absence of the carboxylic acid in the enantiodetermining step,^34,37^ emphasizing the general need for chiral Cp^V^Co catalysts to cater the many transformations where a chiral acid strategy^17^ cannot be employed. Overall, these trailblazing experiments point toward a bright future potential for pentasubstituted Cp^V^ cobalt­(III) complexes as stable and performant catalytic agents, which could be applied as chiral Cp*Co equivalents in its broad catalytic scope of C–H functionalizations.^38^

The precise and simultaneous control of a third reaction parameter on top of high levels of reactivity and enantio-induction, for instance introducing diastereoselectivity, poses a formidable catalytic challenge. In this regard, we selected the (4 + 2) C–H annulation of benzhydroxamate **10** with cyclopropene **11** as exemplary benchmarking transformation (Scheme 8). Besides Cp*,^39^ two different classes of Cp^x^ ligands (types **G** and **H**) have been reported for this transformation.^13e^ ^,^ ^40^ However, neither of them performed on all three parameters at a satisfactory level. Achiral Cp*Rh delivered annulated product **12** in good yield and 15:1 dr, though evidently as a racemate. Disubstituted chiral catalyst **Rh** ^**di**^ **3** provided 91:9 er, but failed in controlling the diastereoselectivity (1:1.1 dr). In contrast, trisubstituted binaphthyl-based **Rh** ^**tri**^ **2** resulted in an excellent >20:1 dr, but fell short regarding yield and enantiocontrol (22%, 64.5:35.5 er). Under identical conditions, we screened a selection of pentasubstituted Cp^V^Rh­(III) catalysts. Isopropyl-bearing **Rh24** with H-sidewalls was able to deliver on all reaction parameters (61%, >20:1 dr, 91:9 er), thereby combining the best aspects of all the previously reported catalysts. Investigation of the central ligand frontarm revealed a cyclohexyl group to be ideal (**Rh25**, 65%, >20:1 dr, 92:8 er), whereas the introduction of OMe/Me-sidewalls (**Rh16**) gave inferior results. As another testament to the strong influence of Cp^V^ substituent modifications, replacing the biphenyl moiety in **Rh20a** for a 3,4,5-trimethoxybenzene group (**Rh19**) resulted in considerable improvements regarding all three reaction parameters. Notably, this contrasts with the underperformance of **Rh19** for the synthesis of spirocycle **6**, thus highlighting the complementarity within our diverse Cp^V^ ligand set.

**Scheme 8:**
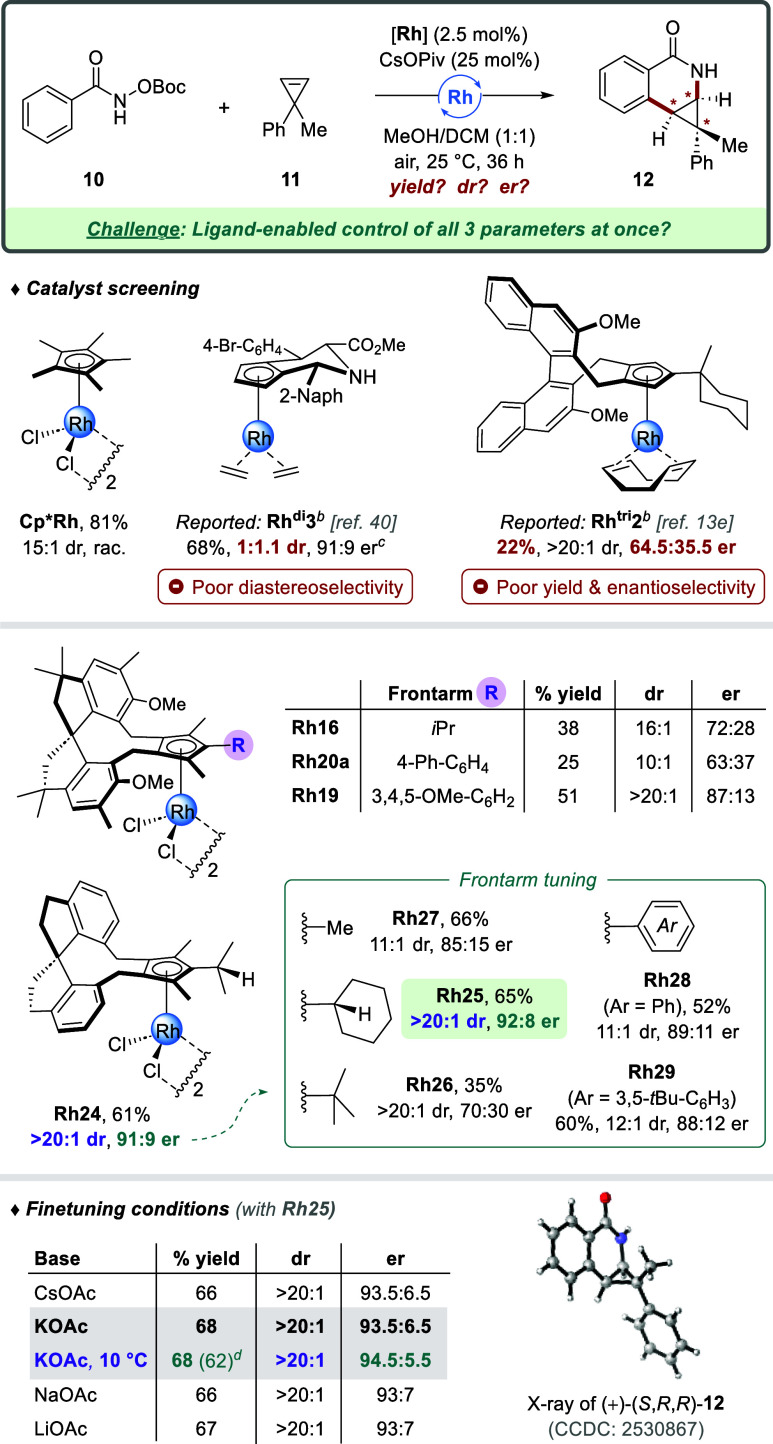
Ligand-Enabled Control of Three Catalytic Parameters in a C–H Annulation: Simultaneously Steering Reactivity, Diastereoselectivity, and Enantioselectivity^a^

a Yields and dr determined by qNMR with 1,3,5-trimethoxybenzene as an internal standard. The solid-state X-ray structure of (*S*,*R*,*R*)-**12** shows 50% probability thermal ellipsoids.
b With (BzO)_2_ instead of CsOPiv.
c Er of the other (*S*,*S*,*S*)-diastereomer.
d Isolated yield at a 0.20 mmol scale.

To fine-tune the reaction outcome for best-performing catalyst **Rh25**, various bases were screened (Scheme 8, bottom). When cesium pivalate was switched for its acetate analog, both yield and enantioselectivity improved. The counterion proved less influential, but potassium was the best, providing **12** in 68% yield and 93.5:6.5 er while maintaining the excellent >20:1 diastereoselectivity. Decreasing the temperature to 10 °C preserved the reactivity and at the same time delivered a higher level of stereocontrol (68%, >20:1 dr, 94.5:5.5 er). The absolute configuration of (+)-**12** was unambiguously determined as (*S*,*R*,*R*) through X-ray crystallography. In sum, pentasubstituted rhodium catalyst **Rh25** was able to simultaneously provide, for the first time, an excellent performance regarding all three reaction parameters (yield, dr, er) in this 3-stereogenic-center-generating C–H annulation.

## Conclusion

In conclusion, we have developed a modular strategy for the rapid assembly of structurally diverse pentasubstituted chiral Cp^V^ ligands. Our synthesis leverages readily accessible 1,2,3-trifunctionalized cyclopentadiene building blocks in a robust one-step dialkylation procedure, incorporating a wide range of chiral binaphthyl- and spirobiindanyl-based *bis*-electrophiles. The combinatorial approach is scalable and features extensive steric and electronic tunability of the Cp^V^ substituents. The Cp^V^ assembly platform enables short syntheses and fast diversification of the ligand set, thus lowering the synthetic burden of catalyst screenings and increasing time and resource economy. The obtained Cp^V^ ligands were complexed to cobalt, rhodium, and iridium (50+ examples), and their electronic nature was parametrized via the corresponding Cp^V^Rh­(III) phosphite species. A rhodium-mediated [1,5]-alkyl shift-complexation cascade of spirodienes was also discovered.

The catalytic properties of Cp^V^ rhodium and cobalt complexes were tested in four exemplary benchmark asymmetric C–H functionalizations. In each case, several members outperformed their established di- and trisubstituted Cp^x^ analogs with simultaneously improved yields, diastereo-, and enantioselectivities. In most instances, the chiral Cp^V^ ligands were found to behave reactivity-wise as Cp* analogs, entailing several benefits: (a) faster, simpler, and high-yielding complexation protocols compared to classical disubstituted Cp^x^ ligands; (b) strongly increased stability of the cobalt complexes, resulting in the first isolation of a bench-stable dicationic Cp^x^Co­(III) species; and (c) similar catalytic behavior as Cp* under identical reaction conditions, thus reducing optimization and adaptation efforts. The very strong responses in reaction selectivity outcome toward easily introduced modifications of the Cp^V^ substituents, including several instances of frontarm-induced enantio-inversion, provide a wealth of screening options on the catalyst tuning level besides the typical reaction optimization parameters.

Overall, the presented work vastly enlarges the existing chiral Cp^x^ landscape and addresses key challenges in asymmetric C–H functionalizations with respect to ligand tunability, accessibility, and catalyst stability. As our combinatorial strategy enables many more different Cp^V^ substitutions, we believe the Cp^V^ platform holds a bright future potential for asymmetric methodology development.

## Supplementary Material



## Notes

Experimental procedures, characterization data for all new compounds, crystallographic details, steric maps, and copies of the chiral HPLC and NMR spectra (PDF)
