# Supplementary material for: Modular Assembly of Chiral Cp* Clones Unlocks Performant Catalysts for Enantioselective C–H Functionalizations
Source: J Am Chem Soc. 2026 Apr 22;148(17):18468–81. doi: 10.1021/jacs.6c05307 (PMC13154215; doi:10.1021/jacs.6c05307)
Supplement: Supplementary file 1 [file ja6c05307_si_001.pdf]

Supporting Information

Modular Assembly of Chiral Cp* Clones Unlocks Performant Catalysts for Enantioselective C—H Functionalizations

Bram Van Den Bossche,[a] *and Nicolai Cramer*[a]*

[a] Laboratory of Asymmetric Catalysis and Synthesis (LCSA),
Institute of Chemical Sciences and Engineering,
École Polytechnique Fédérale de Lausanne (EPFL),
BCH 4305, Avenue F.-A. Forel 2, 1015 Lausanne, Switzerland.

* Corresponding author. E-mail: nicolai.cramer@epfl.ch
Homepage: https://www.epfl.ch/labs/lcsa/

Table of Contents

1. **General Methods** **S3**

2. **Synthesis of 1,2,3-Cp Building Blocks** **S6**

3. **Synthesis of Chiral Backwall Precursors** **S7**

4. **Synthesis of Chiral Cyclopentadienes** **S27**

   4.1 Classical Synthesis of Di- and Trisubstituted Cp^xH S27

   4.2 Convergent Modular Synthesis of Pentasubstituted Cp^VH S32

5. **Synthesis of Chiral Cp Metal Complexes** **S49**

   5.1 Di- and Trisubstituted Cp^x Metal Complexes S49

   5.2 Pentasubstituted Cp^V Cobalt Complexes S53

   5.3 Pentasubstituted Cp^V Rhodium Complexes S61

   5.4 Pentasubstituted Cp^V Iridium Complexes S82

   5.5 Rh(III)-Mediated [1,5]-Alkyl Shift-Complexation Cascade S85

   5.6 Cationic Cp^V Cobalt(III) and Rhodium(III) Complexes S88

   5.7 Cp^V Cobalt(III) and Rhodium(III) Phosphite Adducts S91

   5.8 Challenging Dialkylation-Complexation Sequences S98

6. **Asymmetric Catalytic Transformations** **S101**

   6.1 Rh-catalyzed Allylic C-H Amination of Unactivated Alkenes S101

   6.2 Rh-catalyzed Dearomative (3 + 2) C-H Spiroannulation of 2-Alkenylphenols S120

   6.3 Co-catalyzed (3 + 2) C-H Spiroannulation towards Chiral Benzosultams S123

   6.4 Rh-catalyzed (4 + 2) C-H Annulation for the Benzamidation of Cyclopropenes S126

7. **X-ray Crystallographic Data** **S130**

8. **Topographical Steric Maps** **S137**

9. **References** **S142**

10. **NMR spectra** **S144**

1. General Methods

Experimental procedures, solvents, and reagents

All reactions were carried out in flame-dried or oven-dried glassware with magnetic stirring and under an atmosphere of nitrogen (using Schlenk techniques or inside a MBraun nitrogen-filled glovebox), unless otherwise indicated. Microwave-promoted reactions were performed in a Biotage Initiator+ microwave system. Reactions at sub-room temperatures (other than 0 °C or -78 °C) were achieved using a Huber TC45E immersion cooler plunged in a Dewar bowl filled with isopropanol. The following solvents were purified by an Innovative Technology Solvent Delivery System: anhydrous dichloromethane (DCM), diethyl ether (Et_2O), acetonitrile (MeCN), toluene (PhMe), and tetrahydrofuran (THF). Solvents inside a nitrogen-filled glovebox (used e.g. for cobalt complexations) were additionally degassed by freeze-pump-thaw technique (3 x) before being stored under 4 Å molecular sieves. Chemicals, and other solvents than the ones mentioned *in supra*, were used as obtained from the suppliers (Sigma, Acros, Alfa Aesar, TCI, abcr, Combi-Blocks, Fluorochem), unless otherwise indicated.

Thin-Layer Chromatography (TLC)

Analytical thin-layer chromatography was performed with commercial glass plates coated with 0.25 mm silica gel (E. Merck, Kieselgel 60 F_{254}). Compounds were either visualized under UV-light at 254 nm, or by dipping the plates in an aqueous potassium permanganate solution, an ethanolic phosphomolybdic acid (PMA) stain, an ethanolic vanillin stain, or an aqueous ceric ammonium molybdate (CAM) stain; afterwards all these stains were developed by heating. Retention factors (R_f) were determined using a 5 x 2 cm plate and are reported with indication of the solvent mixture used. Preparative thin-layer chromatography (Prep. TLC) for purification of samples up to 20 mg per plate (20 x 20 cm) was performed using the exact set-up as for analytical TLC. Compounds were visualized under UV-light at 254 nm.

Flash Column Chromatography (FCC)

Flash column chromatography was performed with SiliCycle *SiliaFlash P60* silica gel (40-63 µm) or neutral alumina (aluminum oxide, activated, neutral, Brockmann I activity), and using pressurized air during elution. The crude products were loaded onto the column either as coated on silica (dry loading, by evaporating *in vacuo* a DCM solution with suspended silica gel) or as solution in a minimal amount of eluent (wet loading). Short pads of silica gel (used for filtrations) were always first packed with eluent.

Nuclear Magnetic Resonance Spectroscopy (NMR)

Proton nuclear magnetic resonance (^1H NMR) data were acquired on a Bruker *AVANCEIII-400* (400 MHz), Bruker *AVANCENEO-500* (500 MHz), Bruker *AVANCEIIIHD-600* (600 MHz), or Bruker *AVANCEII-800* (800 MHz) spectrometer at 298 K, unless otherwise indicated. Chemical shifts (δ) are reported in parts per million (ppm) relative to incompletely deuterated $CDCl_3$ (s, 7.26 ppm), C_6D_6 (s, 7.16 ppm), or CD_2Cl_2 (t, 5.32 ppm, J = 1.1 Hz). Splitting patterns are designated as s, singlet; d, doublet; t, triplet; q, quartet; p, pentet; h, hextet; hept, heptet; dd, doublet of doublets; m, multiplet; br, broad; app, apparent; or combinations thereof. For quantitative ^1H NMR (qNMR) experiments, the spectra were recorded with a relaxation delay (d_1) of 10 seconds. TraceCERT® internal standards (1,3,5-trimethoxybenzene, ethylene carbonate) were purchased from Sigma-Aldrich and used as obtained.

Proton-decoupled carbon-13 nuclear magnetic resonance ($^{13}C\{^1H\}$ NMR) data were acquired on a Bruker *AVANCEIII-400* (101 MHz), Bruker *AVANCENEO-500* (126 MHz), Bruker *AVANCEIIIHD-600* (151 MHz), or Bruker *AVANCEII-800* (201 MHz) spectrometer at 298 K, unless otherwise indicated. Chemical shifts are reported in ppm relative to $CDCl_3$ (77.16 ppm), C_6D_6 (128.06 ppm), or CD_2Cl_2 (53.84 ppm). If symmetric carbons are present, they are listed as one signal. If an apparent multiplicity is observed due to coupling with other nuclei (e.g. ^{19}F, ^{31}P, ^{103}Rh), the splitting pattern and coupling constant are provided. Proton-decoupled fluorine-19 nuclear magnetic resonance ($^{19}F\{^1H\}$ NMR) data were acquired on a Bruker *AVANCEIII-400* (376 MHz) spectrometer at 298 K, unless otherwise indicated. Proton-decoupled phosphorus-31 nuclear magnetic resonance ($^{31}P\{^1H\}$ NMR) data were acquired on a Bruker *AVANCEIII-400* (162 MHz) spectrometer at 298 K, unless otherwise indicated.

Infrared Spectroscopy (IR)

Infrared (IR) data were recorded on a Bruker *Alpha-P* FT-IR spectrometer. Absorbance frequencies are reported in reciprocal centimeters (cm^{-1}). Compounds were applied as a solution and, after evaporation of the volatile solvent (usually DCM or $CHCl_3$), measured as a thin film. Relative signal intensities are designated as s, strong; m, medium; w, weak.

High Resolution Mass Spectrometry (HRMS)

HRMS measurements were performed on an Agilent LC-MS TOF (multimode: ESI + APCI), Waters Xevo G2-S QTOF (ESI, APPI, or APCI), Exploris 240 (Sicrit plasma), or Thermo Orbitrap Elite (nanochip-ESI) mass spectrometer. High resolution mass values of the indicated ion are given in *m/z*. For the ion is provided both a molecular formula, as an indication of the added/removed ions or molecules compared to the parent molecule M.

Melting point determination (M.p.)

Melting points were measured on a Büchi *B-540* apparatus and are uncorrected. They are represented as a range, indicating the temperatures at which the melting process started and ended. Melting points were determined for solids only (not for foams or metal complexes).

Optical rotation ($[\alpha]_D^T$)

Optical rotations were measured on a Polartronic M polarimeter, using a 10.0 cm cell with a Na 589 nm filter. The temperature T, at which the optical rotation was recorded, is indicated in superscript (usually 23 °C). The solvent, in which the measurement took place (usually $CHCl_3$), and the applied concentration *c* (in g/100 mL) are provided as well. Noteworthily, for many colored metal complexes (e.g. $[Cp^VRhCl_2]_2$ or $Cp^VCo(CO)I_2$ type species), the optical rotation value fluctuated with time and an averaged value is therefore provided.

Chiral High-Performance Liquid Chromatography (Chiral HPLC)

Analytical separation of enantiomers to determine enantiomeric ratio (er) values was performed on a Shimadzu Prominence UFLC XR system (at 35 °C), using CHIRALPAK®-type chiral stationary phase columns (4.6 x 150 mm, 3 µm particle size) and a mixture of hexane/isopropanol as eluent.

X-ray Crystallographic Analysis (XRD)

Single crystal X-ray diffraction analysis was performed by Dr. F. Fadaei-Tirani at the EPFL in Lausanne. The employed apparatus and applied experimental methodology are described for each crystal at the end of the Supporting Information, together with the obtained crystal data. For each structure, the crystallographic data has been deposited at the Cambridge Crystallographic Data Center (CCDC). Using the CCDC reference numbers provided, copies of the data can be obtained online and free of charge.

Author Contributions

BVDB designed and performed all the experiments and analyzed the results. Both authors conceived and conceptualized the project, interpreted the results, and performed the writing and revision of the manuscript. NC provided funding and resources.

No generative AI tools (ChatGPT, Grammarly, etc.) were used at any stage during the preparation or revision of the main manuscript or the Supporting Information.

2. Synthesis of 1,2,3-Cp Building Blocks

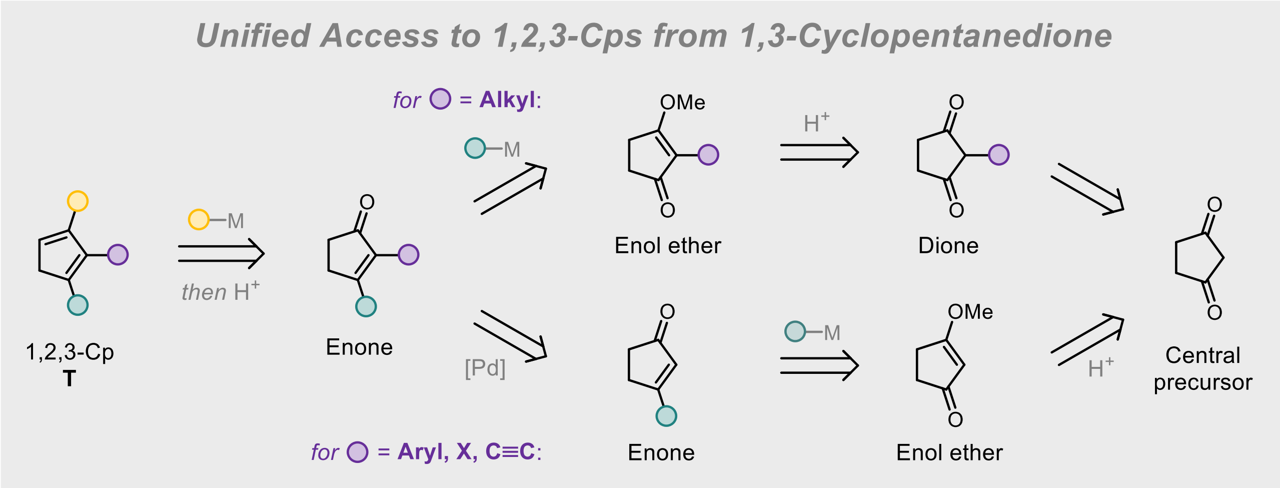

Scheme S1. General overview of the unified synthetic access to diverse 1,2,3-trisubstituted cyclopentadienes **T**.

All 1,2,3-trisubstituted cyclopentadiene (1,2,3-Cp) building blocks **T** were conveniently accessed in a unified way from 1,3-cyclopentanedione as inexpensive central precursor through a concise and scalable route (**Scheme S1**), which was recently reported by our group.[1] They could be stored for many months in a freezer without decomposition. The 1,2,3-Cps **T1-17** used in this work are outlined in **Figure S1**.

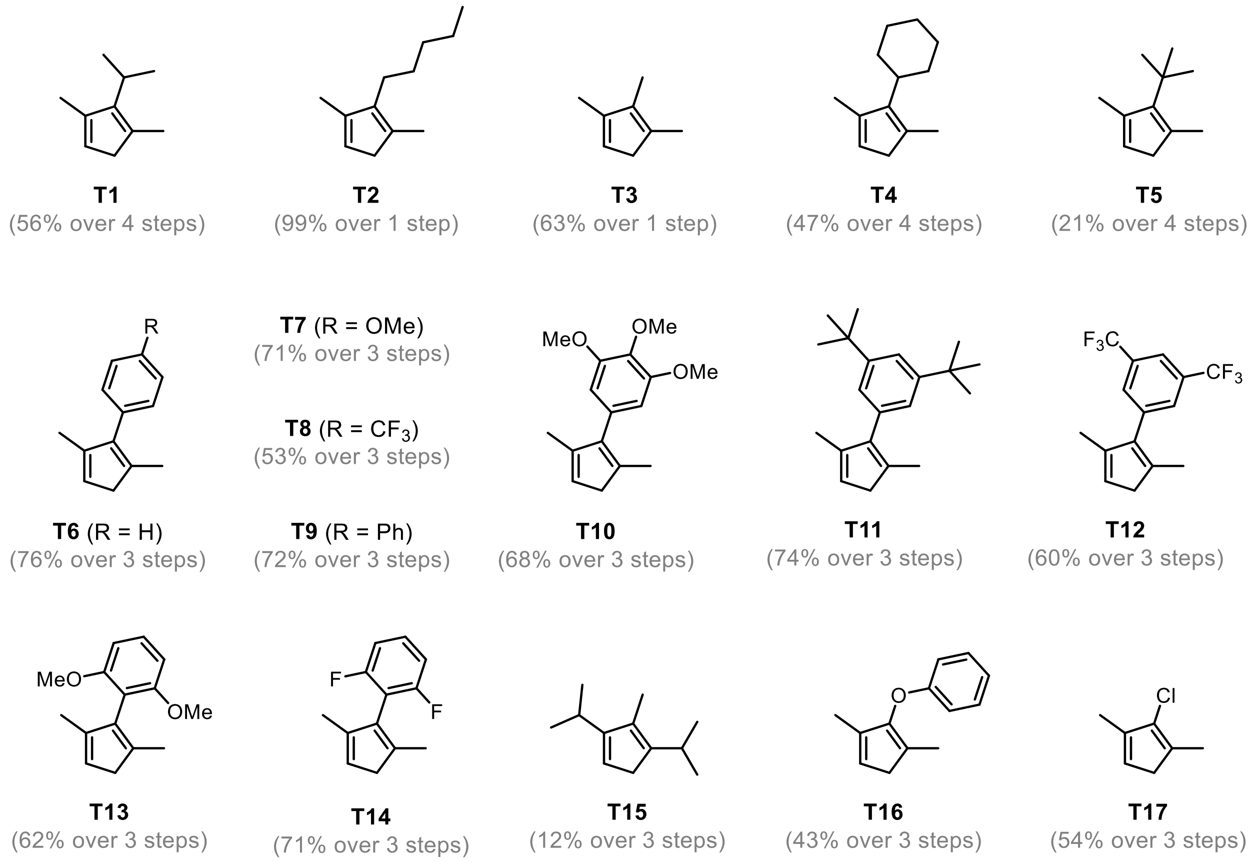

Figure S1. Overview of the 1,2,3-trisubstituted cyclopentadienes **T** used in this work.

3. Synthesis of Chiral Backwall Precursors

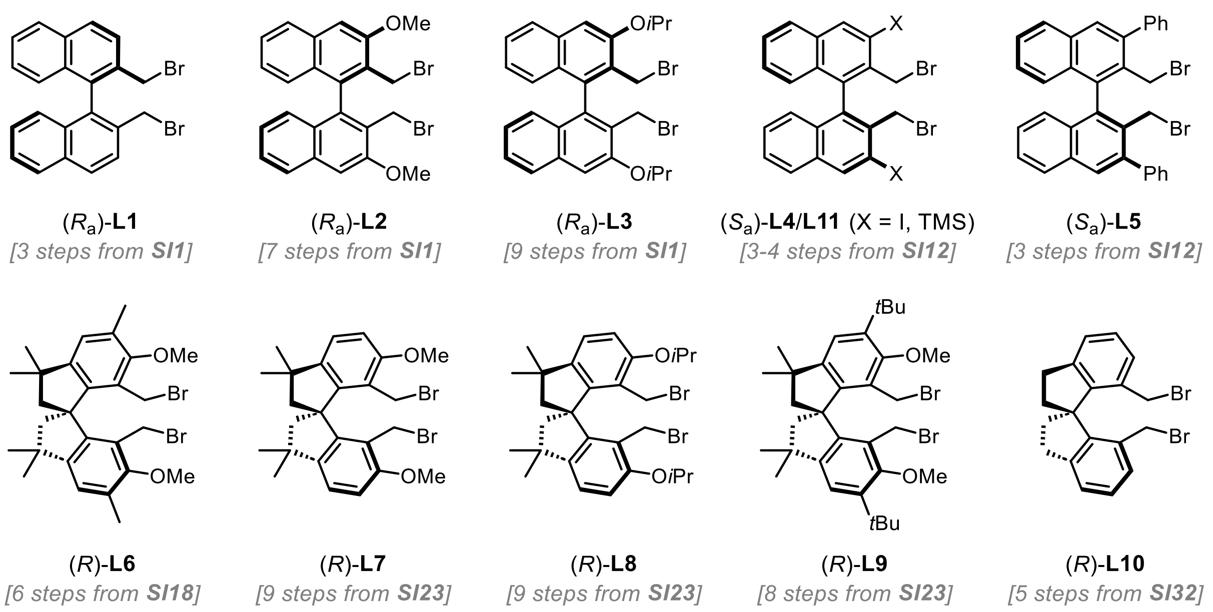

Figure S2. Overview of the chiral backbone precursors **L** used in this work.

All chiral backwall precursors **L1-11** used in this work (**Figure S2**) could be prepared on multi-gram scale according to the reported procedures, or adaptations thereof, as described *in infra*.

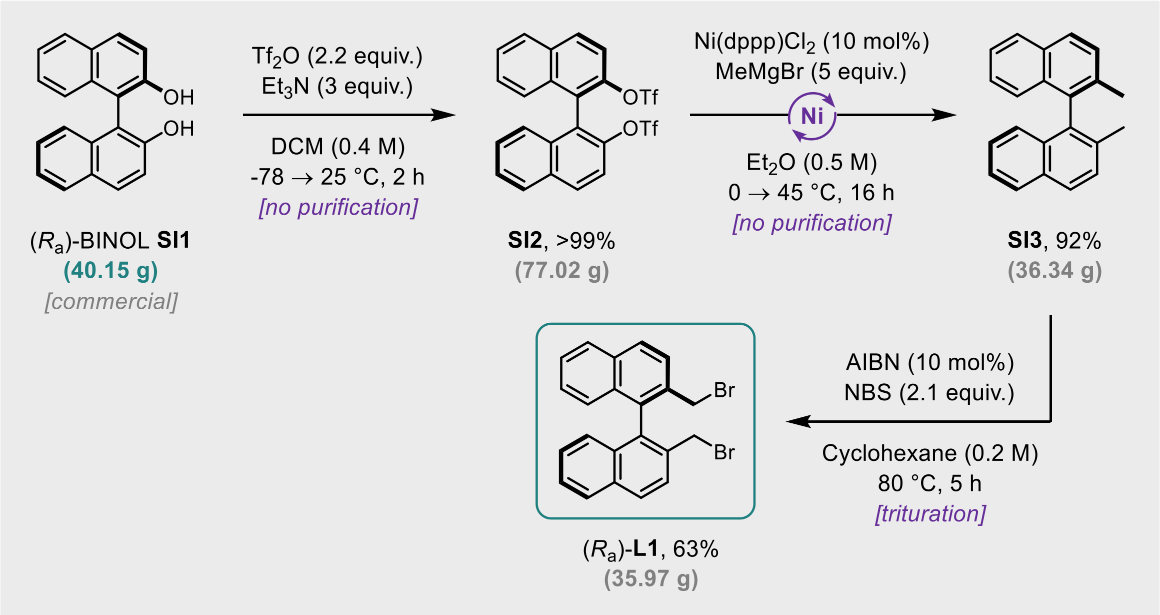

Scheme S2. Preparation of chiral dibromide (R_a)-**L1** in 3 steps from (R_a)-BINOL **SI1**.

[1] Synthesis of Chiral Dibromide (Ra)-L1

Chiral dibromide (R_a)-**L1** was prepared in 3 steps from commercial (R_a)-BINOL **SI1** (CAS 18531-94-7) according to the following adaptations (**Scheme S2**) of reported procedures.[2,3] The protocol was done

on 40.15 g (140 mmol) scale, ultimately affording 35.97 g (82 mmol) of (R_a)-**L1** in a single run without requiring column chromatography. The sole purification was performed by trituration after the last step.

Step 1 – Triflation. In a flame-dried round-bottom flask was made a solution of (R_a)-BINOL **SI1** (40.15 g, 140.2 mmol) in anhydrous DCM (2.5 mL/mmol) under an atmosphere of nitrogen, after which triethylamine (59 mL, 420.7 mmol, 3 equiv.) was added. At -78 °C (acetone/dry ice bath), trifluoromethanesulfonic anhydride (53 mL, 308.5 mmol, 2.2 equiv.) was added dropwise to the colourless solution *via* an addition funnel. The cold bath was removed, and the resulting black reaction mixture was stirred while warming up to room temperature (25 °C) for 2 hours. Next, the reaction was quenched by addition of water (2.5 mL/mmol) and poured into ice-cold aq. HCl (1 M, 1.5 mL/mmol), after which it was extracted with DCM (2 x). The combined organic layers were sequentially washed with aq. HCl (1 M, 2 x), water, sat. aq. $NaHCO_3$ solution, and brine. The black solution was dried over $MgSO_4$ and then filtered through a pad of silica gel (10 cm height) with DCM as eluent, giving a yellow solution. All volatiles were removed *in vacuo*, affording *bis*-triflate **SI2** (77.02 g, 139.9 mmol, quantitative yield) as a light-orange solid with the characterization data matching those previously reported,[3] and in sufficient purity to be used in the next step without further purification.

^{1}H NMR (400 MHz, $CDCl_3$) δ = 8.15 (d, J = 9.0 Hz, 2H), 8.01 (d, J = 8.4 Hz, 2H), 7.63 (d, J = 9.1 Hz, 2H), 7.61 – 7.57 (m, 2H), 7.44 – 7.39 (m, 2H), 7.28 – 7.24 (m, 2H) ppm; **R_f** (pentane/EtOAc, 9:1) = 0.43.

Step 2 – Kumada coupling. In a flame-dried three-necked round-bottom flask was made a solution of *bis*-triflate **SI2** (77.02 g, 139.9 mmol) in anhydrous Et_2O (2 mL/mmol) under an atmosphere of nitrogen. [1,3-Bis(diphenyl-phosphino)propane]dichloronickel(II) powder (7.74 g, 14.0 mmol, 10 mol%) was added in one portion and under a counterflow of nitrogen. At 0 °C (ice bath), a methylmagnesium bromide solution (3.0 M in Et_2O, 233 mL, 699.4 mmol, 5 equiv.) was added dropwise *via* an addition funnel. The cold bath was removed, and the reaction mixture was refluxed (oil bath at 45 °C) for 16 hours. After cooling to room temperature (25 °C), the reaction was carefully quenched by dropwise addition of sat. aq. NH_4Cl solution (0.5 mL/mmol) and then filtered through a pad of celite (3 cm) with Et_2O as eluent. After extraction with Et_2O (2 x), the combined organic layers were washed with brine. The orange solution was dried over $MgSO_4$ and then filtered through a pad of silica gel (10 cm height) with DCM as eluent, giving a light-yellow solution. All volatiles were removed *in vacuo*, affording product **SI3** (36.34 g, 128.7 mmol, 92% yield) as a light-yellow solid with the characterization data matching those previously reported,[2] and in sufficient purity to be used in the next step without further purification.

^{1}H NMR (400 MHz, $CDCl_3$) δ = 7.91 – 7.86 (m, 4H), 7.51 (d, J = 8.4 Hz, 2H), 7.42 – 7.37 (m, 2H), 7.23 – 7.18 (m, 2H), 7.07 – 7.02 (m, 2H), 2.04 (s, 6H) ppm; **R_f** (pentane/EtOAc, 9:1) = 0.70.

Step 3 – Radical bromination. In a flame-dried round-bottom flask was made a solution of **SI3** (36.34 g, 128.7 mmol), azobisisobutyronitrile (AIBN, 2.16 g, 12.9 mmol, 10 mol%), and recrystallized *N*-bromo-succinimide (NBS, 48.10 g, 270.3 mmol, 2.1 equiv.) in cyclohexane (5 mL/mmol) under an atmosphere of nitrogen. The reaction mixture was stirred at reflux (oil bath at 100 °C) for 5 hours. After cooling to room temperature (25 °C), the reaction was diluted with ethyl acetate, which redissolved all precipitated white solids, and then washed with sequentially water (3 x) and brine. The orange solution was dried

over $MgSO_4$ and then filtered through a pad of silica gel (10 cm height) with DCM as eluent, giving a yellow solution. Removal of all volatiles *in vacuo* provided a light-brown solid, to which methanol (3 mL/mmol) was added, and the resulting suspension was refluxed (oil bath at 90 °C) for 2 hours under vigorous stirring. Next, heating was turned off and the suspension was cooled down slowly to room temperature (25 °C) under continued stirring. After filtration on a glass sinter funnel, the obtained solids were washed with methanol, then dried *in vacuo*, affording chiral dibromide **L1** (35.97 g, 81.7 mmol, 63% yield) as a white powder with the characterization data matching those previously reported.[2]

^{1}H NMR (400 MHz, $CDCl_3$) δ = 8.02 (d, *J* = 8.5 Hz, 2H), 7.93 (d, *J* = 8.3 Hz, 2H), 7.75 (d, *J* = 8.6 Hz, 2H), 7.52 – 7.47 (m, 2H), 7.31 – 7.25 (m, 2H), 7.10 – 7.05 (m, 2H), 4.26 (s, 4H) ppm; **R_f** (pentane/EtOAc, 9:1) = 0.55.

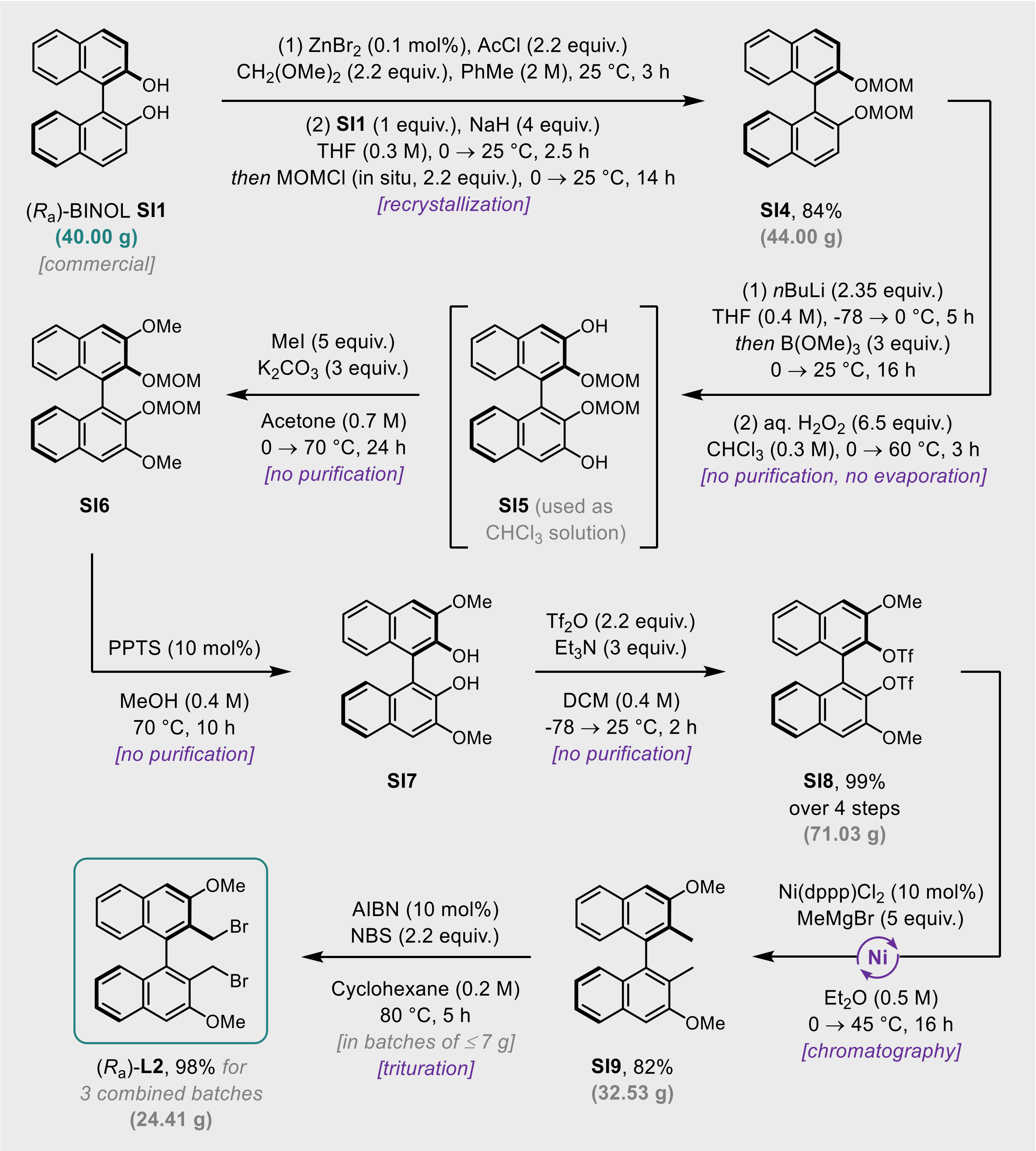

Scheme S3. Preparation of chiral dibromide (R_a)-**L2** in 7 steps from (R_a)-BINOL **SI1**.

[2] Synthesis of Chiral Dibromide (R_a)-L2

Chiral dibromide (R_a)-**L2** was prepared in 7 steps from commercial (R_a)-BINOL **SI1** (CAS 18531-94-7) according to the following adaptations (**Scheme S3**) of reported procedures.[2,4,5] Purifications were only done after step 1 (recrystallization), step 6 (column chromatography), and step 7 (trituration).

Step 1 – MOM-protection. First, the MOMCl reagent was prepared in situ.[6] In a flame-dried 250 mL two-necked round-bottom flask was made a solution of dimethoxymethane (27.8 mL, 307.3 mmol, 2.2 equiv.) and zinc(II) bromide (31 mg, 139 µmol, 0.1 mol%) in anhydrous toluene (0.5 mL/mmol, i.e. 70 mL) under an atmosphere of nitrogen. At room temperature (25 °C), acetyl chloride (22.1 mL, 307.3 mmol, 2.2 equiv.) was added dropwise over *ca.* 20 min *via* an addition funnel, after which the reaction mixture was stirred for another 3 hours. Notably, the addition process was exothermic. In the meantime, in a flame-dried 2 L three-necked round-bottom flask was made a suspension of sodium hydride (60% in mineral oil, 22.35 g, 558.8 mmol, 4 equiv.) in anhydrous THF (1 mL/mmol) under an atmosphere of nitrogen. At 0 °C (ice bath), a solution of (R_a)-BINOL **SI1** (40.00 g, 139.7 mmol) in anhydrous THF (2 mL/mmol) was added dropwise over *ca.* 2 hours *via* an addition funnel, after which the reaction mixture was stirred for another 30 min. Notably, this deprotonation should be performed carefully inside a well-ventilated fume hood due to the large amount of concomitant H_2 gas evolution. Next, at 0 °C (ice bath), the in situ prepared MOMCl solution (*ca.* 2.2 equiv.) was added dropwise *via* an addition funnel to the deprotonated BINOL solution. The reaction mixture was allowed to slowly warm up in the cold bath to room temperature (25 °C) under stirring for 14 hours. Next, the reaction was quenched by dropwise addition of water (20 mL), after which it was further diluted with water (2 mL/mmol) and extracted with Et_2O (2 x). The combined organic layers were washed with brine, dried over $MgSO_4$, and filtered. All volatiles were removed *in vacuo*, affording a yellow solid (mixed with mineral oil originating from the commercial NaH dispersion). Methanol (200 mL) was added, and the resulting suspension was refluxed (oil bath at 80 °C) for 1 hour under vigorous stirring. Next, heating and stirring were turned off, and the homogeneous solution was cooled down slowly to room temperature (25 °C) to induce crystallization. After filtration on a glass sinter funnel, the obtained crystals were washed with pentane, then dried *in vacuo*, affording product **SI4** (44.00 g, 117.5 mmol, 84% yield) as a white crystalline solid with the characterization data matching those previously reported.[7]

^{1}H NMR (400 MHz, $CDCl_3$) δ = 7.95 (d, *J* = 9.0 Hz, 2H), 7.87 (d, *J* = 8.2 Hz, 2H), 7.58 (d, *J* = 9.0 Hz, 2H), 7.37 – 7.32 (m, 2H), 7.25 – 7.20 (m, 2H), 7.16 (d, *J* = 8.5 Hz, 2H), 5.08 (d, *J* = 6.8 Hz, 2H), 4.98 (d, *J* = 6.7 Hz, 2H), 3.15 (s, 6H) ppm; **R_f** (pentane/EtOAc, 4:1) = 0.42.

Step 2 – One-pot Lithiation-Boronation-Oxidation. In a flame-dried 1 L three-necked round-bottom flask was made a solution of **SI4** (44.00 g, 117.5 mmol) in anhydrous THF (2.5 mL/mmol) under an atmosphere of nitrogen. At -78 °C (acetone/dry ice bath), a *n*-butyllithium solution (1.6 M in hexane, 173 mL, 276.2 mmol, 2.35 equiv.) was added dropwise to the colorless solution *via* an addition funnel. The resulting deep red mixture was stirred for 4 hours while warming up slowly to 0 °C, at which point it was put into an ice bath and stirred for 1 more hour at 0 °C, turning into a thick light-brown mixture. Next, at 0 °C (ice bath), trimethyl borate (40.4 mL, 352.5 mmol, 3 equiv.) was added dropwise *via* an addition funnel and under vigorous stirring. The resulting thick light-grey slurry was gently shaken *via* a shaking

plate for 16 hours at room temperature (25 °C), gradually turning into a light-green solution. All volatiles were removed *in vacuo*, affording a grey-green gum, which was dissolved in chloroform (3 mL/mmol). Next, at 0 °C (ice bath), hydrogen peroxide (30% in H_2O, 78.0 mL, 763.8 mmol, 6.5 equiv.) was added dropwise *via* an addition funnel under vigorous stirring. Notably, this addition process was exothermic. The reaction mixture was refluxed (oil bath at 80 °C) for 3 hours, providing a bright orange solution that contains a grey precipitate. Next, at 0 °C (ice bath), the reaction was quenched and diluted with water (4 mL/mmol). After extraction with chloroform (3 x 1 mL/mmol), the combined organic layers were washed with brine and then filtered through a pad of Na_2SO_4 (10 cm height), giving an orange solution. Importantly, no evaporation was performed, and the chloroform solution of crude **SI5** was directly used as such in the next step. Analysis of a small aliquot by ^{1}H NMR did confirm the successful formation of **SI5** in sufficient purity, and with the characterization data matching those previously reported.[2]

^{1}H NMR (400 MHz, $CDCl_3$) δ = 7.77 (d, *J* = 8.3 Hz, 2H), 7.50 (s, 2H), 7.41 (s, 2H), 7.40 – 7.35 (m, 2H), 7.15 – 7.09 (m, 2H), 7.06 – 7.02 (m, 2H), 4.71 (d, *J* = 6.3 Hz, 2H), 4.63 (d, *J* = 6.3 Hz, 2H), 3.39 (s, 6H) ppm; **R_f** (pentane/EtOAc, 4:1) = 0.19.

Step 3 – Methylation. At 0 °C (ice bath), under air, a solution of potassium carbonate (ground *via* mortar, 49.21 g, 352.5 mmol, 3 equiv.) in acetone (1.5 mL/mmol) was directly added to a chloroform solution of crude **SI5** (assumed 117.5 mmol, 1 equiv.) that was obtained in the previous step. Then, at 0 °C, iodomethane (36.9 mL, 587.6 mmol, 5 equiv.) was added dropwise *via* an addition funnel. The ice bath was removed, and the light-yellow reaction mixture was stirred at reflux (oil bath at 70 °C) for 24 hours. If the conversion was incomplete, more iodomethane (1 equiv.) was added before refluxing for another 6 hours. After cooling to room temperature (25 °C), the reaction mixture was directly filtered through a pad of silica gel (10 cm height) with DCM as eluent, giving an orange solution. All volatiles were removed *in vacuo*, affording crude product **SI6** with the characterization data matching those previously reported,[2] and in sufficient purity to be used in the next step without further purification.

^{1}H NMR (400 MHz, $CDCl_3$) δ = 7.76 (d, *J* = 8.2 Hz, 2H), 7.38 – 7.33 (m, 2H), 7.29 (s, 2H), 7.18 – 7.09 (m, 4H), 4.96 (d, *J* = 5.6 Hz, 2H), 4.83 (d, *J* = 5.6 Hz, 2H), 4.02 (s, 6H), 2.58 (s, 6H) ppm; **R_f** (pentane/EtOAc, 2:1) = 0.42.

Step 4 – MOM-deprotection. In a round-bottom flask was made a suspension of crude **SI6** (assumed 117.5 mmol, 1 equiv.) in methanol (2.5 mL/mmol) under air. Pyridin-1-ium 4-methylbenzenesulfonate (PPTS, 3.01 g, 11.8 mmol, 10 mol%) was added, and the reaction mixture was stirred at 70 °C for 10 hours. Next, after evaporation of all volatiles *in vacuo*, the residue was redissolved in DCM and filtered through a pad of silica gel (10 cm height) with DCM as eluent. All volatiles were removed *in vacuo*, affording crude product **SI7** with the characterization data matching those previously reported,[2] and in sufficient purity to be used in the next step without further purification.

^{1}H NMR (400 MHz, $CDCl_3$) δ = 7.79 (d, *J* = 8.1 Hz, 2H), 7.36 – 7.29 (m, 4H), 7.18 – 7.14 (m, 4H), 5.91 (s, 2H), 4.09 (s, 6H) ppm; **R_f** (pentane/EtOAc, 2:1) = 0.22.

Step 5 – Triflation. In a flame-dried round-bottom flask was made a solution of crude **SI7** (assumed 117.5 mmol, 1 equiv.) in anhydrous DCM (2.5 mL/mmol) under an atmosphere of nitrogen, after which triethylamine (49.6 mL, 352.5 mmol, 3 equiv.) was added. At -78 °C (acetone/dry ice bath), trifluoromethanesulfonic anhydride 44.4 mL, 258.5 mmol, 2.2 equiv.) was added dropwise to the orange solution *via* an addition funnel. The cold bath was removed, and the resulting black reaction mixture was stirred while warming up to room temperature (25 °C) for 3 hours. Next, the reaction was quenched by addition of water (2.5 mL/mmol) and poured into ice-cold aq. HCl (1 M, 1.5 mL/mmol), after which it was extracted with DCM (2 x). The combined organic layers were sequentially washed with aq. HCl (1 M, 2 x), water, sat. aq. $NaHCO_3$ solution, and brine. The black solution was dried over $MgSO_4$ and then filtered through a pad of silica gel (10 cm height) with DCM as eluent. All volatiles were removed *in vacuo*, affording *bis*-triflate **SI8** (71.03 g, 116.3 mmol, 99% yield over 4 steps) as a light-orange solid with the characterization data matching those previously reported,[2] and in sufficient purity to be used in the next step without further purification.

^{1}H NMR (400 MHz, $CDCl_3$) δ = 7.89 – 7.85 (m, 2H), 7.54 – 7.50 (m, 2H), 7.49 (s, 2H), 7.26 – 7.21 (m, 2H), 7.16 – 7.12 (m, 2H), 4.11 (s, 6H) ppm; **R_f** (pentane/EtOAc, 4:1) = 0.36.

Step 6 – Kumada coupling. In a flame-dried 1L three-necked round-bottom flask was made a solution of *bis*-triflate **SI8** (70.95 g, 116.2 mmol) in anhydrous Et_2O (2 mL/mmol) under an atmosphere of nitrogen. [1,3-Bis(diphenylphosphino)propane]dichloronickel(II) powder (6.43 g, 11.6 mmol, 10 mol%) was added in one portion and under a counterflow of nitrogen. At 0 °C (ice bath), a methylmagnesium bromide solution (3.0 M in Et_2O, 194 mL, 581.1 mmol, 5 equiv.) was added dropwise *via* an addition funnel. The cold bath was removed, and the reaction mixture was refluxed (oil bath at 45 °C) for 16 hours. After cooling to room temperature (25 °C), the reaction was carefully quenched by dropwise addition of sat. aq. NH_4Cl solution (0.5 mL/mmol) and then filtered through a pad of celite (3 cm) with Et_2O as eluent, removing the brown precipitate. After extraction with Et_2O (2 x), the combined organic layers were washed with brine, dried over $MgSO_4$, and then filtered through a pad of silica gel (10 cm height) with DCM as eluent. Removal of all volatiles *in vacuo* provided a brown solid, which was purified by flash column chromatography on silica gel (dry loading, 20 cm column height, gradient: pentane/EtOAc = 50:1 → 10:1), affording product **SI9** (32.53 g, 95.0 mmol, 83% yield) as a white crystalline solid with the characterization data matching those previously reported.[2]

^{1}H NMR (400 MHz, $CDCl_3$) δ = 7.79 (d, *J* = 8.2 Hz, 2H), 7.38 – 7.33 (m, 2H), 7.23 (s, 2H), 7.09 – 7.03 (m, 2H), 6.96 (d, *J* = 8.4 Hz, 2H), 4.03 (s, 6H), 1.92 (s, 6H) ppm; **R_f** (pentane/EtOAc, 9:1) = 0.50.

Step 7 – Radical bromination. Notably, this step was done in multiple batches of up to 7 g scale each. To exclusively obtain the desired benzylic bromination product **L2**, and avoid any parasitic aromatic *ortho*-bromination,[5] it is essential to use recrystallized NBS reagent (procedure *in infra*), and to ensure that chromatographically purified **SI9** does not contain solvent traces. As such, in a flame-dried round-bottom flask was made a solution of **SI9** (6.23 g, 18.2 mmol), azobisisobutyronitrile (AIBN, 305 mg, 1.8 mmol, 10 mol%), and recrystallized *N*-bromosuccinimide (NBS, 7.12 g, 40.0 mmol, 2.2 equiv.) in cyclohexane (5 mL/mmol) under an atmosphere of nitrogen. The reaction mixture was stirred at reflux (oil bath at 100 °C) for 5 hours. After cooling to room temperature (25 °C), the resulting bright yellow

mixture was diluted with ethyl acetate, which redissolved all precipitated solids. At this point, multiple batches could be combined to allow for a single work-up and purification. The combined organic layers were sequentially washed with water (3 x) and brine, dried over $MgSO_4$, and then filtered through a pad of silica gel (10 cm height) with DCM as eluent. Removal of all volatiles *in vacuo* provided chiral dibromide **L2** (24.41 g, 48.8 mmol, 98% yield for 3 combined batches) as a light-beige solid with the characterization data matching those previously reported,[4] and usually in excellent purity as assessed by ^{1}H NMR. Further purification, if required, could be achieved by trituration with a minimal amount of hot methanol to provide an off-white powder.

^{1}H NMR (400 MHz, $CDCl_3$) δ = 7.81 (d, *J* = 8.2 Hz, 2H), 7.46 – 7.41 (m, 2H), 7.33 (s, 2H), 7.13 – 7.08 (m, 2H), 7.01 – 6.97 (m, 2H), 4.34 (d, *J* = 9.5 Hz, 2H), 4.28 (d, *J* = 9.5 Hz, 2H), 4.11 (s, 6H) ppm; **R_f** (pentane/EtOAc, 9:1) = 0.29.

Recrystallizing NBS. Inside a well-ventilated fume hood, at 95 °C (oil bath), commercial *N*-bromo-succinimide (100 g, light-yellow powder) was added to a 2 L round-bottom flask containing pre-heated water (*ca*. 1 L) under vigorous stirring. Caution should be exercised because of the orange Br_2 fumes evolving during this process. If necessary, more water was added until a homogeneous orange solution was obtained. Next, heating and stirring were turned off, and the solution was cooled down slowly to room temperature (25 °C) over several hours to induce crystallization. The resulting white flaky crystals were filtered on a glass sinter funnel through a bed of ice, washed with water, and then dried *in vacuo*. These crystals could be stored in a freezer at -20 °C for at least 2 years without the need to repurify.

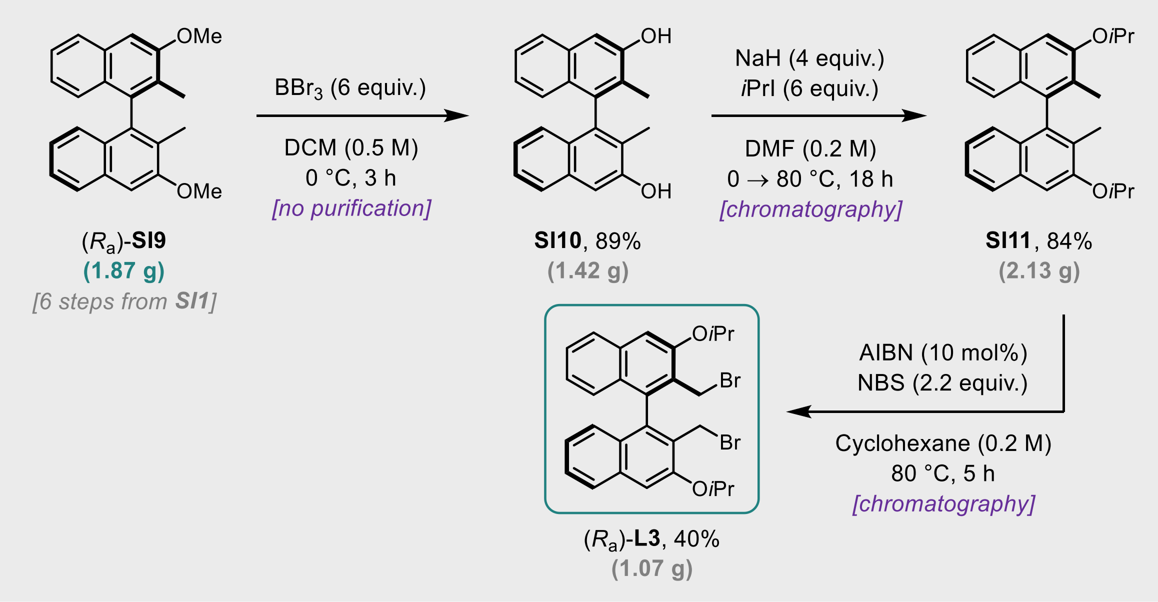

Scheme S4. Preparation of chiral dibromide (R_a)-**L3** in 9 steps from (R_a)-BINOL **SI1**.

[3] Synthesis of Chiral Dibromide (R_a)-L3

Chiral dibromide (R_a)-**L3** was prepared in 3 additional steps from compound (R_a)-**SI9** according to the following procedure (**Scheme S4**).

Step 1 – Ether cleavage. Adapted from a reported procedure.[8] In a flame-dried round-bottom flask was made a solution of **SI9** (1872 mg, 5.09 mmol) in anhydrous DCM (2 mL/mmol) under an atmosphere of nitrogen. At 0 °C (ice bath), a boron tribromide solution (1.0 M in DCM, 3.41 mL, 34.13 mmol, 6 equiv.) was added dropwise. The reaction mixture was stirred for 3 hours at 0 °C. Next, still at 0 °C, it was carefully quenched with water, after which sat. aq. $NaHCO_3$ solution (50 mL/mmol) was added. After adjustment of the pH value to *ca.* 5 by addition of acetic acid, it was extracted with DCM (3 x), dried over $MgSO_4$, filtered, and concentrated under reduced pressure, affording product **SI10** (1416 mg, 4.51 mmol, 89% yield) as a light-yellow solid with the characterization data matching those previously reported,[8] and in sufficient purity to be used in the next step without further purification.

^{1}H NMR (400 MHz, $CDCl_3$) δ = 7.73 (d, *J* = 8.2 Hz, 2H), 7.38 – 7.32 (m, 2H), 7.27 (s, 2H), 7.09 – 7.04 (m, 2H), 6.95 (d, *J* = 8.4 Hz, 2H), 5.08 (s, 2H), 1.97 (s, 6H) ppm.

Step 2 – Alkylation. At 0 °C (ice bath) and under an atmosphere of nitrogen, sodium hydride (deoiled, 607 mg, 25.28 mmol, 4 equiv.) was added portion-wise to a solution of **SI10** (2136 mg, 6.32 mmol) in anhydrous dimethylformamide (5 mL/mmol) under a counterflow of nitrogen. After stirring for 15 min at 0 °C, isopropyl iodide (3.49 mL, 37.91 mmol, 6 equiv.) was added. The dark orange reaction mixture was stirred at 80 °C for 18 hours. If required, extra isopropyl iodide (2 equiv.) was added to ensure full conversion. After cooling to room temperature (25 °C), the reaction was quenched with sat. aq. NH_4Cl and extracted with ethyl acetate (3 x). The combined organic layers were consecutively washed with water (5 x) and brine, dried over $MgSO_4$, filtered, and concentrated under reduced pressure. The residue was purified by flash column chromatography on silica gel (dry loading, 18 cm column height, isocratic: pentane/EtOAc = 50:1), affording product **SI11** (2128 mg, 5.34 mmol, 84% yield) as a white solid.

(R_a)-3,3'-Diisopropoxy-2,2'-dimethyl-1,1'-binaphthalene (**SI11**)

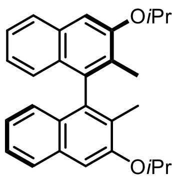

^{1}H NMR (400 MHz, $CDCl_3$) δ = 7.77 – 7.73 (m, 2H), 7.36 – 7.30 (m, 2H), 7.22 (s, 2H), 7.06 – 7.01 (m, 2H), 6.97 – 6.93 (m, 2H), 4.80 (hept, *J* = 6.0 Hz, 2H), 1.90 (s, 6H), 1.48 (d, *J* = 6.0 Hz, 12H) ppm; **^{13}C{^{1}H} NMR** (101 MHz, $CDCl_3$) δ = 154.9, 137.0, 133.3, 128.6, 128.1, 126.6, 126.0, 125.4, 123.6, 106.7, 70.2, 22.4, 13.8 ppm; **IR** (ATR) ṽ = 3060 (w), 2976 (w), 2925 (w), 1619 (w), 1596 (w), 1572 (w), 1500 (w), 1433 (m), 1384 (w), 1374 (w), 1323 (w), 1286 (w), 1262 (w), 1231 (m), 1167 (w), 1111 (s), 1026 (w), 952 (w), 862 (w), 831 (w), 780 (w), 746 (w), 696 (w) cm^{-1}; **HRMS** (ESI/QTOF) *m/z* = calcd. for $[C_{28}H_{31}O_2]^+$, $[M+H]^+$: 399.2319, found: 399.2313; **R_f** (pentane/EtOAc, 50:1) = 0.29; **M.p.** = 86-87 °C; $[\alpha]_D^{23}$ = +30.1 (*c* = 1.0, $CHCl_3$).

Step 3 – Radical bromination. According to the procedure described for **L2**, but starting from **SI11** (2078 mg, 4.85 mmol), chiral dibromide **L3** was obtained as a white sticky foam. Purification was performed by flash column chromatography on silica gel (dry loading, 20 cm column height, gradient: pentane/DCM = 9:1 → 5:1). Suspending the obtained foam in methanol, then evaporating it, afforded **L3** (1074 mg, 1.93 mmol, 40% yield) as a free-flowing white solid. Notably, the use of chromatographically purified **SI11** (free of solvent traces) and recrystallized NBS was again important to avoid undesired aromatic *ortho*-bromination.

(*R*a)-2,2'-Bis(bromomethyl)-3,3'-diisopropoxy-1,1'-binaphthalene (**L3**)

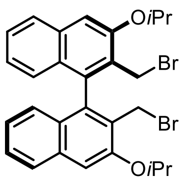

^{1}H NMR (400 MHz, $CDCl_3$) δ = 7.79 – 7.74 (m, 2H), 7.44 – 7.38 (m, 2H), 7.30 (s, 2H), 7.11 – 7.05 (m, 2H), 7.02 – 6.97 (m, 2H), 4.90 (hept, *J* = 6.1 Hz, 2H), 4.36 – 4.25 (m, 4H), 1.54 (d, *J* = 6.0 Hz, 12H) ppm; **^{13}C{^{1}H} NMR** (101 MHz, $CDCl_3$) δ = 153.9, 136.7, 134.8, 127.54, 127.52, 127.4, 127.2, 126.6, 124.0, 107.9, 70.6, 28.3, 22.2, 22.1 ppm; **IR** (ATR) ṽ = 2977 (w), 1619 (m), 1595 (s), 1569 (w), 1433 (m), 1385 (w), 1373 (w), 1326 (m), 1293 (w), 1237 (w), 1214 (m), 1179 (s), 1149 (w), 1131 (w), 1113 (s), 956 (m), 866 (w), 834 (w), 746 (m), 675 (w), 469 (w) cm^{-1}; **HRMS** (APPI/LTQ-Orbitrap) *m/z* = calcd. for $[C_{28}H_{28}Br_2O_2]^+$, $[M]^+$: 554.0451, found: 554.0467; **R$_f$** (pentane/DCM, 9:1) = 0.25; **M.p.** = 129-130 °C; $[\alpha]_D^{26}$ = +139.4 (*c* = 0.3, $CHCl_3$).

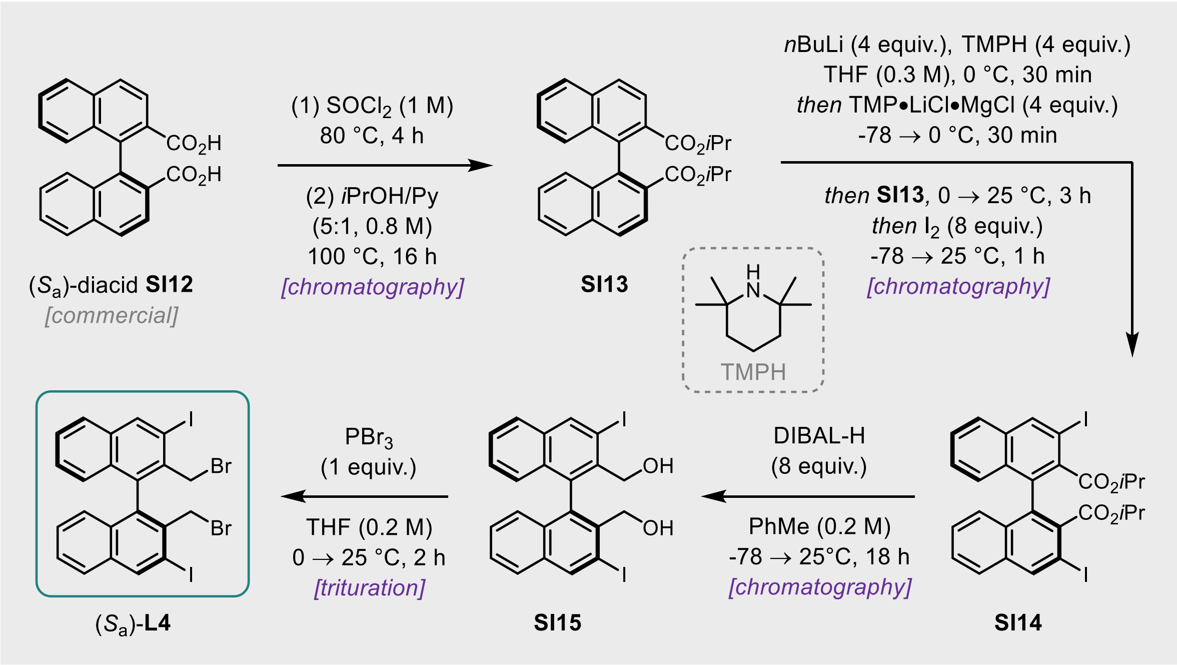

Scheme S5. Preparation of chiral dibromide (*S*a)-**L4** in 4 steps from (*S*a)-diacid **SI12**.

[4] Synthesis of Chiral Dibromide (S$_a$)-L4

Chiral dibromide (*S*a)-**L4** was prepared in 4 steps from commercial diacid (*S*a)-**SI12** (CAS 18531-96-9) according to the reported procedures (**Scheme S5**) with matching characterization data.[9,10]

For (*S*a)-**SI13**:
^{1}H NMR (400 MHz, $CDCl_3$) δ = 8.17 (d, *J* = 8.7 Hz, 2H), 8.01 (d, *J* = 8.7 Hz, 2H), 7.92 (d, *J* = 8.2 Hz, 2H), 7.53 – 7.47 (m, 2H), 7.26 – 7.21 (m, 2H), 7.13 (d, *J* = 8.6 Hz, 2H), 4.76 (hept, *J* = 6.3 Hz, 2H), 0.76 (d, *J* = 6.2 Hz, 6H), 0.44 (d, *J* = 6.3 Hz, 6H) ppm.

For (*S*a)-**SI14**:
^{1}H NMR (400 MHz, $CDCl_3$) δ = 8.52 (s, 2H), 7.78 (d, *J* = 8.3 Hz, 2H), 7.54 – 7.47 (m, 2H), 7.37 – 7.30 (m, 2H), 7.20 – 7.15 (m, 2H), 4.76 (hept, *J* = 6.3 Hz, 2H), 0.76 (d, *J* = 6.3 Hz, 6H), 0.69 (d, *J* = 6.3 Hz, 6H) ppm.

For (S_a)-**SI15**:

^{1}H NMR (400 MHz, $CDCl_3$) δ = 8.61 (s, 2H), 7.84 – 7.79 (m, 2H), 7.52 – 7.46 (m, 2H), 7.30 – 7.25 (m, 2H), 6.92 – 6.88 (m, 2H), 4.61 (d, *J* = 12.3 Hz, 2H), 4.16 (d, *J* = 12.3 Hz, 2H) ppm.

For (S_a)-**L4**:

^{1}H NMR (400 MHz, $CDCl_3$) δ = 8.65 (s, 2H), 7.82 (d, *J* = 8.3 Hz, 2H), 7.55 – 7.48 (m, 2H), 7.32 – 7.26 (m, 3H), 7.00 – 6.96 (m, 2H), 4.41 (d, *J* = 10.4 Hz, 2H), 4.32 (d, *J* = 10.5 Hz, 2H) ppm.

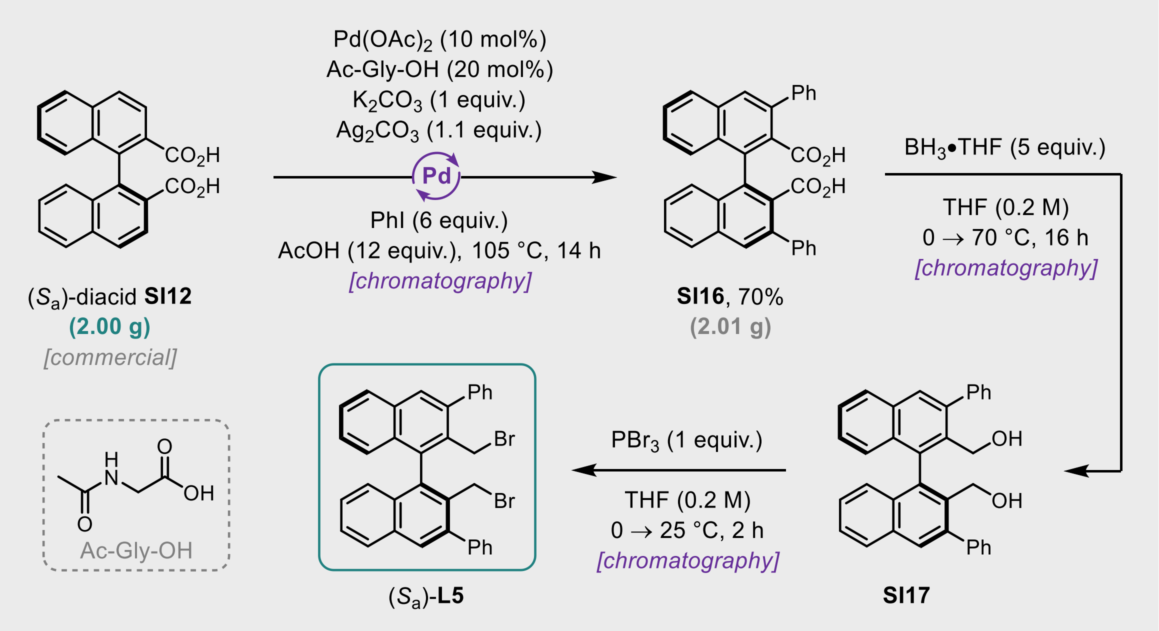

Scheme S6. Preparation of chiral dibromide (S_a)-**L5** in 3 steps from (S_a)-diacid **SI12**.

[5] Synthesis of Chiral Dibromide (S_a)-L5

Chiral dibromide (S_a)-**L5** was prepared in 3 steps from commercial diacid (S_a)-**SI12** (CAS 18531-96-9) according to the reported procedure (**Scheme S6**) with matching characterization data.[11] Notably, diacid **SI12** itself can also be prepared from BINOL **SI1** in 3 steps *via* triflation toward **SI2**, Pd-catalyzed carbonylation, and ester hydrolysis according to a reported procedure.[12]

For (S_a)-**SI16**:

Prepared according to the following scaled-up adaptation of a reported procedure.[11] An oven-dried 20 mL microwave vial was charged with diacid **SI12** (2.00 g, 5.84 mmol), palladium(II) acetate (131 mg, 0.58 mmol, 10 mol%), *N*-acetylglycine (137 mg, 1.17 mmol, 20 mol%), potassium carbonate (807 mg, 5.84 mmol, 1 equiv.), and silver(I) carbonate (1.77 g, 6.43 mmol, 1.1 equiv.). The vial was capped and placed under an atmosphere of nitrogen by Schlenk technique. Next, iodobenzene (3.9 mL, 35.05 mmol, 6 equiv.) and acetic acid (4.0 mL, 70.10 mmol, 12 equiv.) were added, and the resulting reaction mixture was stirred in a heating block at 105 °C for 14 hours. After cooling to room temperature (25 °C) and carefully releasing the built-up pressure, the reaction was quenched with aq. HCl (1 M) and then filtered through a pad of celite (4 cm) with ethyl acetate as eluent. After extraction with ethyl acetate (3 x), the dark orange combined organic layers were dried over $MgSO_4$ and concentrated under reduced pressure. The residue was purified by flash column chromatography on silica gel (dry loading, 18 cm column

height, gradient: pentane/EtOAc = 3:1 → 1:2 with 1% AcOH), affording product **SI16** (2.01 g, 4.06 mmol, 70% yield) as an orange solid with the characterization data matching those previously reported.[13]

^{1}H NMR (400 MHz, $CDCl_3$) δ = 8.04 (s, 2H), 7.95 (d, *J* = 8.2 Hz, 2H), 7.59 (d, *J* = 7.3 Hz, 4H), 7.54 (t, *J* = 7.5 Hz, 2H), 7.50 – 7.39 (m, 6H), 7.32 (t, *J* = 7.7 Hz, 2H), 7.14 (d, *J* = 8.5 Hz, 2H) ppm; **R_f** (pentane/EtOAc, 2:1 with 1% AcOH) = 0.11.

For (S_a)-**SI17**:
^{1}H NMR (400 MHz, $CDCl_3$) δ = 7.96 (s, 2H), 7.93 (d, *J* = 8.2 Hz, 2H), 7.74 – 7.70 (m, 4H), 7.53 – 7.40 (m, 8H), 7.29 – 7.23 (m, 2H), 7.05 (d, *J* = 8.5 Hz, 2H), 4.43 (d, *J* = 11.4 Hz, 2H), 4.17 (d, *J* = 11.4 Hz, 2H) ppm.

For (S_a)-**L5**:
^{1}H NMR (400 MHz, $CDCl_3$) δ = 7.94 – 7.90 (m, 4H), 7.64 – 7.60 (m, 4H), 7.54 – 7.42 (m, 8H), 7.32 – 7.27 (m, 2H), 7.19 – 7.15 (m, 2H), 4.32 – 4.25 (m, 4H) ppm.

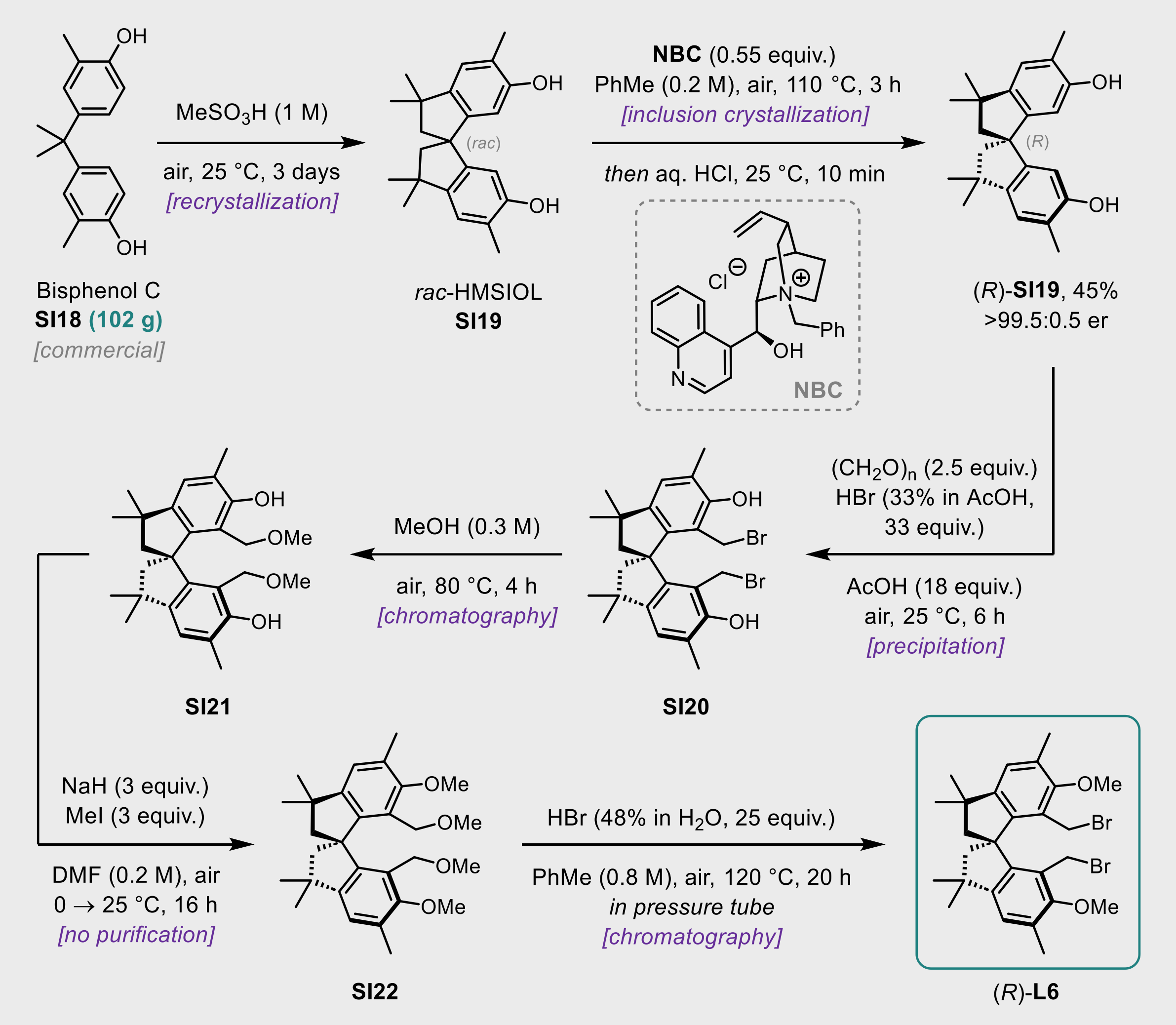

Scheme S7. Preparation of chiral dibromide (*R*)-**L6** in 6 steps from Bisphenol C **SI18**.

[6] Synthesis of Chiral Dibromide (R)-L6

Chiral dibromide (*R*)-**L6** was prepared in 6 steps (all under air) from commercial Bisphenol C **SI18** (CAS 79-97-0) according to the reported procedure (**Scheme S7**) with matching characterization data.[14] Resolution of racemic hexamethylated spirobiindane-6,6'-diol (HMSIOL) **SI19** was conveniently done by inclusion crystallization using (8*S*,9*R*)-*N*-benzylcinchonidinium chloride **NBC** (CAS 69257-04-1).[15]

For (*R*)-**SI19**:

^{1}H NMR (400 MHz, $CDCl_3$) δ = 6.90 (s, 2H), 6.12 (s, 2H), 4.27 (s, 2H), 2.29 (d, *J* = 13.1 Hz, 2H), 2.23 (s, 6H), 2.18 (d, *J* = 13.0 Hz, 2H), 1.36 (s, 6H), 1.30 (s, 6H) ppm; **Chiral HPLC** (Chiralpak IG, 4.6 x 150 mm, 3 µm, Hexane/*i*PrOH 95:5, 1.0 mL/min, 35 °C, 210 nm) t_r (major) = 5.95 min, t_r (minor) = 7.32 min, >99.5:0.5 er.

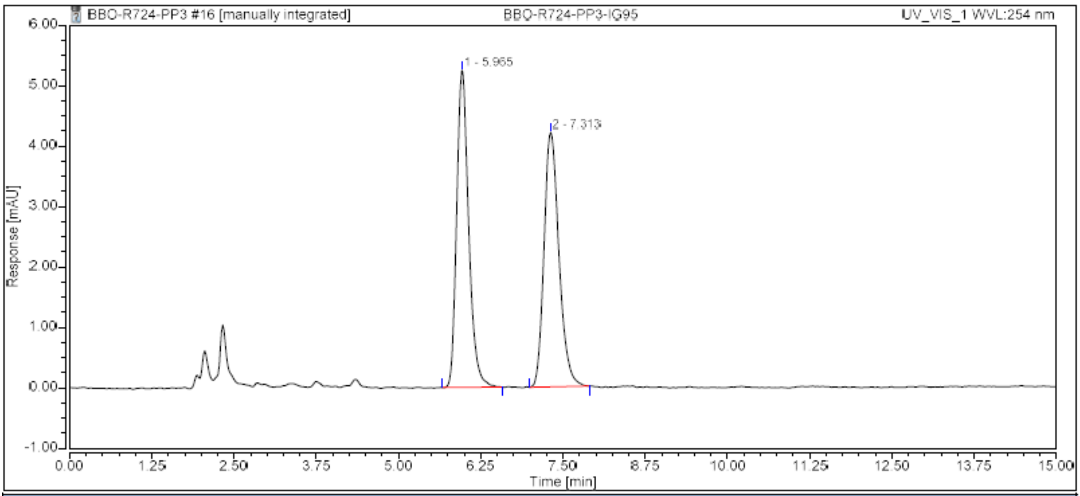

Integration Results

No.	Peak Name	Retention Time min	Area mAU*min	Height mAU	Relative Area %	Relative Height %	Amount n.a.
1		5.965	1.106	5.247	49.96	55.46	n.a.
2		7.313	1.108	4.213	50.04	44.54	n.a.

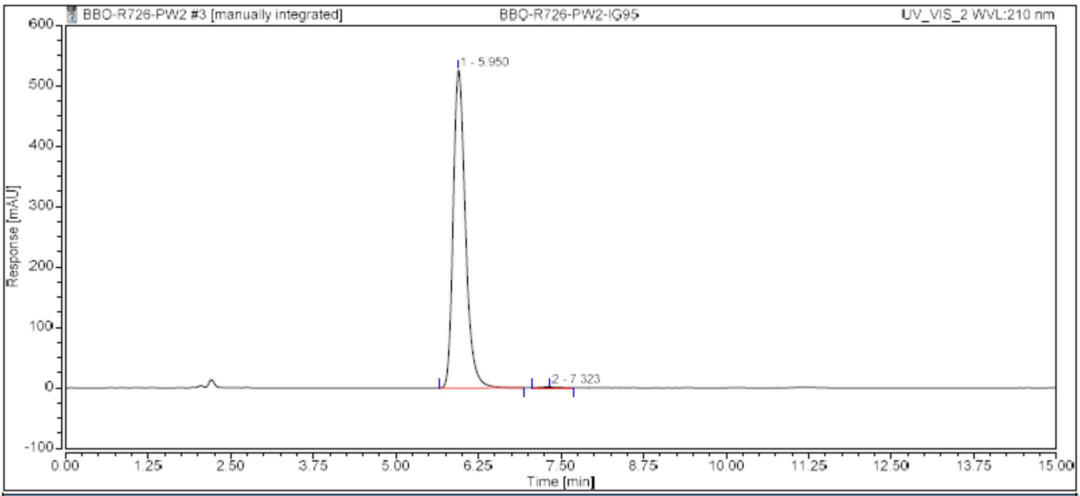

Integration Results

No.	Peak Name	Retention Time min	Area mAU*min	Height mAU	Relative Area %	Relative Height %	Amount n.a.
1		5.950	112.635	526.358	99.69	99.75	n.a.
2		7.323	0.355	1.323	0.31	0.25	n.a.

For (*R*)-**SI20**:

^{1}H NMR (400 MHz, $CDCl_3$) δ = 6.98 (s, 2H), 5.05 (s, 2H), 4.19 (d, *J* = 10.3 Hz, 2H), 4.02 (d, *J* = 10.3 Hz, 2H), 2.47 (d, *J* = 13.5 Hz, 2H), 2.34 (d, *J* = 13.4 Hz, 2H), 2.28 (s, 6H), 1.42 (s, 6H), 1.32 (s, 6H) ppm.

For (*R*)-**SI21**:
^{1}H NMR (400 MHz, $CDCl_3$) δ = 7.82 (s, 2H), 6.89 (s, 2H), 4.15 (d, *J* = 12.5 Hz, 2H), 4.04 (d, *J* = 12.5 Hz, 2H), 3.13 (s, 6H), 2.34 (d, *J* = 13.3 Hz, 2H), 2.25 (s, 6H), 2.13 (d, *J* = 13.4 Hz, 2H), 1.36 (s, 6H), 1.30 (s, 6H) ppm.

For (*R*)-**SI22**:
^{1}H NMR (400 MHz, $CDCl_3$) δ = 6.95 (s, 2H), 3.90 (d, *J* = 9.1 Hz, 2H), 3.77 – 3.73 (m, 8H), 2.96 (s, 6H), 2.48 (d, *J* = 13.1 Hz, 2H), 2.34 – 2.29 (m, 8H), 1.36 (s, 6H), 1.30 (s, 6H) ppm.

For (*R*)-**L6**:
^{1}H NMR (400 MHz, $CDCl_3$) δ = 7.00 (s, 2H), 4.23 (d, *J* = 9.5 Hz, 2H), 3.92 (d, *J* = 9.5 Hz, 2H), 3.86 (s, 6H), 2.63 (d, *J* = 13.4 Hz, 2H), 2.33 (s, 6H), 2.30 (d, *J* = 13.4 Hz, 2H), 1.43 (s, 6H), 1.33 (s, 6H) ppm.

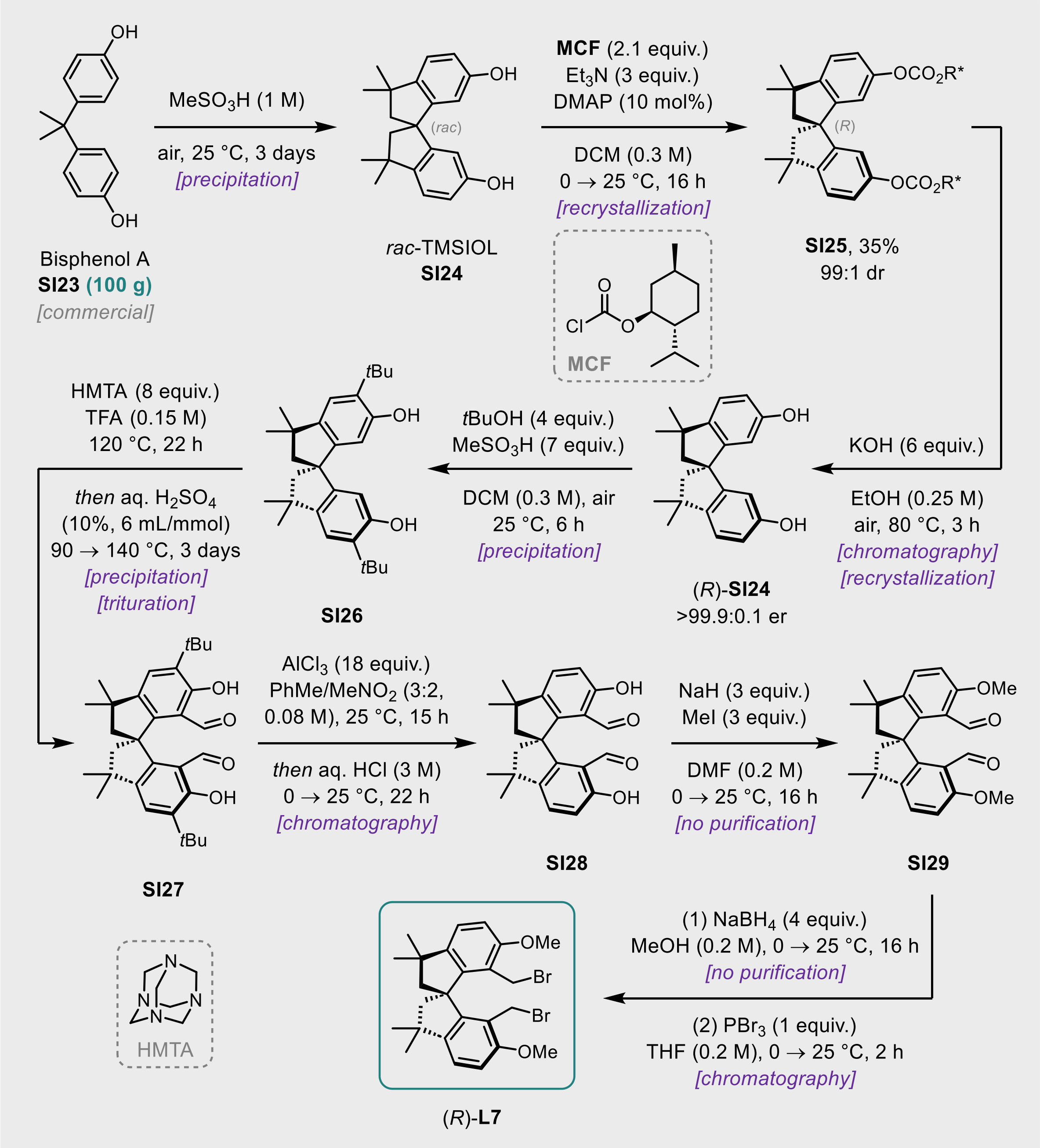

Scheme S8. Preparation of chiral dibromide (*R*)-**L7** in 9 steps from Bisphenol A **SI23**.

[7] Synthesis of Chiral Dibromide (R)-L7

Chiral dibromide (*R*)-**L7** was prepared in 9 steps from commercial Bisphenol A **SI23** (CAS 80-05-7) according to the reported procedures (**Scheme S8**) with matching characterization data.[14,16] Resolution of racemic tetramethylated spirobiindane-6,6'-diol (TMSIOL) **SI24** was performed by diastereomeric separation using (−)-menthyl chloroformate **MCF** (CAS 14602-86-9) and further recrystallization.[17,18]

For (*R*)-**SI24**:

^{1}H NMR (400 MHz, $CDCl_3$) δ = 7.02 (d, *J* = 8.2 Hz, 2H), 6.70 (dd, *J* = 8.2, 2.5 Hz, 2H), 6.25 (d, *J* = 2.4 Hz, 2H), 4.59 (s, 2H), 2.33 (d, *J* = 13.1 Hz, 2H), 2.22 (d, *J* = 13.1 Hz, 2H), 1.36 (s, 6H), 1.31 (s, 6H) ppm; **Chiral HPLC** (Chiralpak IA, 4.6 x 150 mm, 3 µm, Hexane/*i*PrOH 90:10, 1.0 mL/min, 35 °C, 210 nm) t_r (minor) = 6.65 min, t_r (major) = 10.41 min, >99.9:0.1 er.

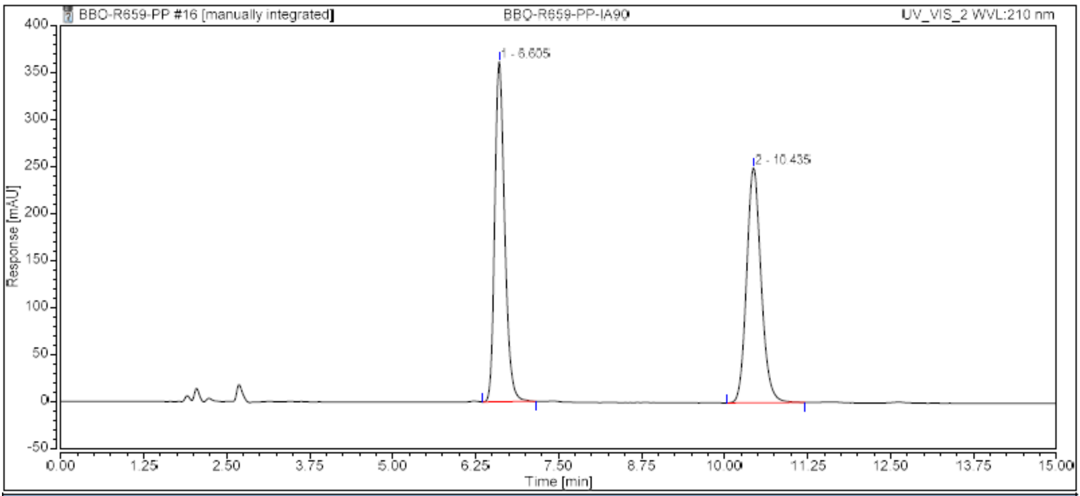

Integration Results

No.	Peak Name	Retention Time min	Area mAU*min	Height mAU	Relative Area %	Relative Height %	Amount n.a.
1		6.605	62.433	361.778	49.55	59.13	n.a.
2		10.435	63.560	250.060	50.45	40.87	n.a.

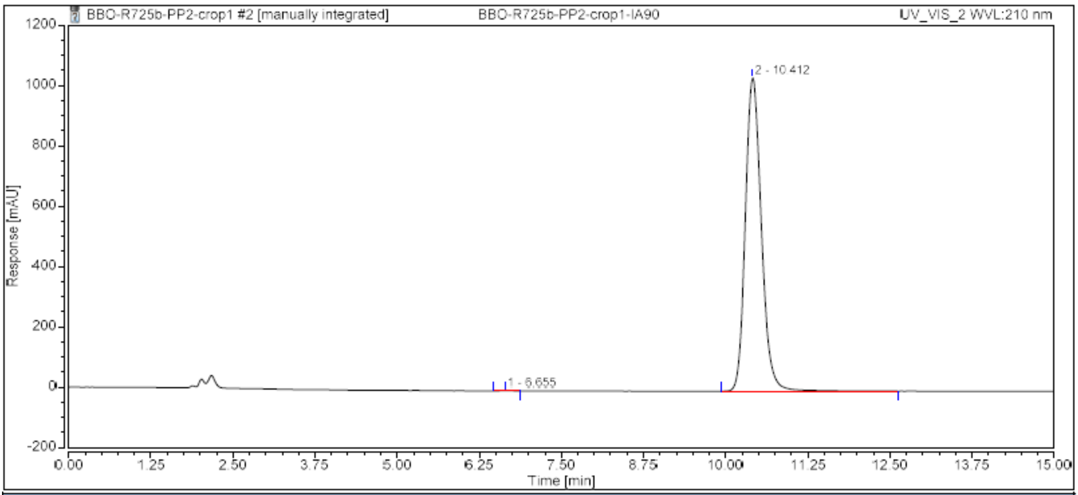

Integration Results

No.	Peak Name	Retention Time min	Area mAU*min	Height mAU	Relative Area %	Relative Height %	Amount n.a.
1		6.655	0.209	1.198	0.07	0.12	n.a.
2		10.412	293.771	1038.490	99.93	99.88	n.a.

For (*R*)-**SI25**:

^{1}H NMR (400 MHz, $CDCl_3$) δ = 7.15 (d, *J* = 8.3 Hz, 2H), 7.03 (dd, *J* = 8.3, 2.3 Hz, 2H), 6.61 (d, *J* = 2.3 Hz, 2H), 4.56 (td, *J* = 10.9, 4.4 Hz, 2H), 2.37 (d, *J* = 13.2 Hz, 2H), 2.26 (d, *J* = 13.1 Hz, 2H), 2.17 – 2.09 (m, 2H),

2.04 – 1.94 (m, 2H), 1.73 – 1.64 (m, 4H), 1.51 – 1.39 (m, 4H), 1.38 (s, 6H), 1.33 (s, 6H), 1.14 – 1.00 (m, 4H), 0.94 – 0.84 (m, 14H), 0.79 (d, *J* = 7.0 Hz, 6H) ppm; **Chiral HPLC** (Chiralpak IB, 4.6 x 150 mm, 3 µm, Hexane/*i*PrOH 99.8:0.2, 1.0 mL/min, 35 °C, 254 nm) t_r (major) = 3.31 min, t_r (minor) = 4.60 min, 99:1 dr.

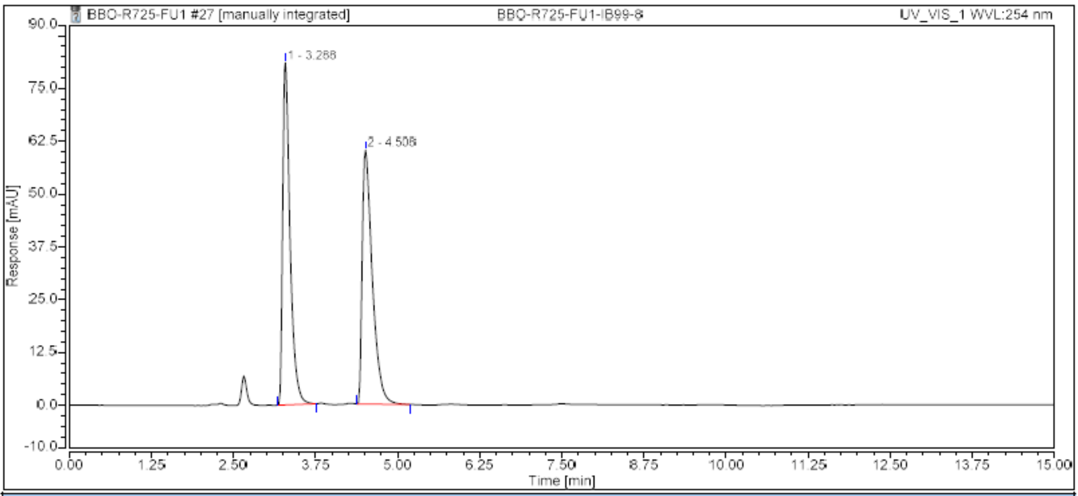

Integration Results

No.	Peak Name	Retention Time min	Area mAU*min	Height mAU	Relative Area %	Relative Height %	Amount n.a.
1		3.288	10.562	80.995	49.74	57.39	n.a.
2		4.508	10.674	60.135	50.26	42.61	n.a.

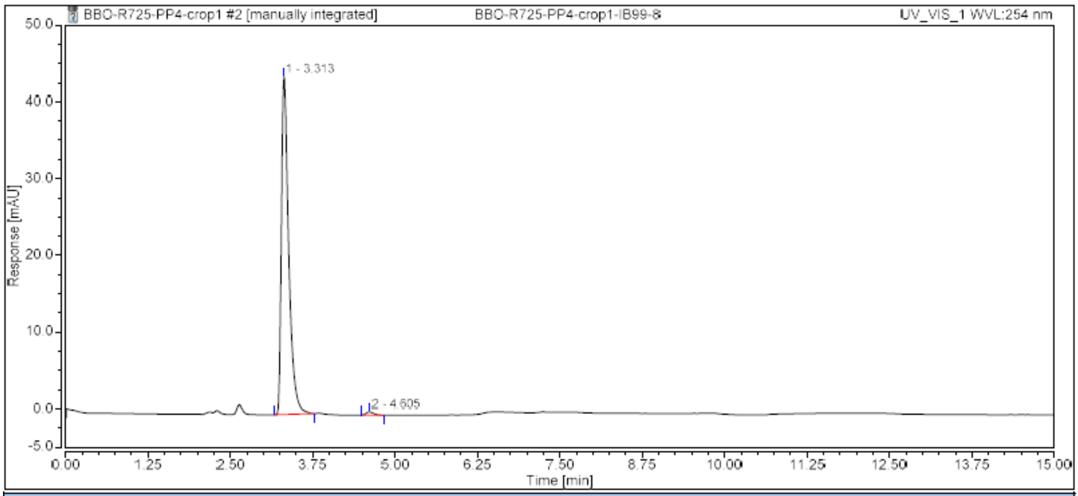

Integration Results

No.	Peak Name	Retention Time min	Area mAU*min	Height mAU	Relative Area %	Relative Height %	Amount n.a.
1		3.313	5.636	44.027	99.06	99.07	n.a.
2		4.605	0.053	0.414	0.94	0.93	n.a.

For (*R*)-**SI26**:

^{1}H NMR (400 MHz, $CDCl_3$) δ = 7.00 (s, 2H), 6.11 (s, 2H), 2.28 (d, *J* = 13.0 Hz, 2H), 2.18 (d, *J* = 13.0 Hz, 2H), 1.41 (s, 18H), 1.35 (s, 6H), 1.30 (s, 6H) ppm.

For (*R*)-**SI27**:

^{1}H NMR (400 MHz, $CDCl_3$) δ = 12.55 (s, 2H), 9.60 (s, 2H), 7.30 (s, 2H), 2.56 (d, *J* = 13.5 Hz, 2H), 2.38 (d, *J* = 13.5 Hz, 2H), 1.42 (s, 18H), 1.37 (s, 6H), 1.35 (s, 6H) ppm.

For (*R*)-**SI28**:

^{1}H NMR (400 MHz, $CDCl_3$) δ = 11.72 (s, 2H), 9.57 (s, 2H), 7.35 (d, *J* = 8.6 Hz, 2H), 6.94 (d, *J* = 8.6 Hz, 2H), 2.62 (d, *J* = 13.6 Hz, 2H), 2.43 (d, *J* = 13.6 Hz, 2H), 1.39 (s, 6H), 1.37 (s, 6H) ppm.

For (*R*)-**SI29**:
^{1}H NMR (400 MHz, $CDCl_3$) δ = 10.06 (s, 2H), 7.36 (d, *J* = 8.5 Hz, 2H), 6.88 (d, *J* = 8.5 Hz, 2H), 3.84 (s, 6H), 2.48 (d, *J* = 12.6 Hz, 2H), 2.33 (d, *J* = 12.6 Hz, 2H), 1.46 (s, 6H), 1.37 (s, 6H) ppm.

For (*R*)-**L7**:
^{1}H NMR (400 MHz, $CDCl_3$) δ = 7.15 (d, *J* = 8.4 Hz, 2H), 6.84 (d, *J* = 8.4 Hz, 2H), 4.21 (d, *J* = 9.5 Hz, 2H), 3.91 – 3.85 (m, 8H), 2.66 (d, *J* = 13.6 Hz, 2H), 2.34 (d, *J* = 13.5 Hz, 2H), 1.45 (s, 6H), 1.34 (s, 6H) ppm.

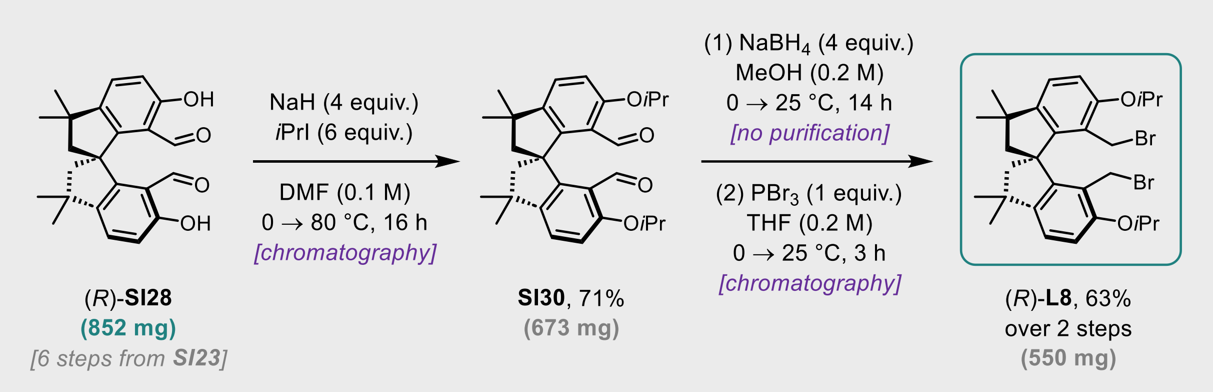

Scheme S9. Preparation of chiral dibromide (*R*)-**L8** in 9 steps from Bisphenol A **SI23**.

[8] Synthesis of Chiral Dibromide (R)-L8

Chiral dibromide (*R*)-**L8** was prepared in 3 additional steps from compound (*R*)-**SI28** according to the following procedure (**Scheme S9**).

Step 1 – Alkylation. At 0 °C (ice bath) and under an atmosphere of nitrogen, sodium hydride (deoiled, 202 mg, 8.41 mmol, 4 equiv.) was added portion-wise to a solution of **SI28** (852 mg, 2.10 mmol) in anhydrous dimethylformamide (10 mL/mmol) under a counterflow of nitrogen. After stirring for 30 min at 0 °C, isopropyl iodide (1.27 mL, 12.62 mmol, 6 equiv.) was added. The dark orange reaction mixture was stirred at 80 °C for 16 hours. After cooling to room temperature (25 °C), the reaction was quenched with sat. aq. NH_4Cl and extracted with ethyl acetate (3 x). The combined organic layers were consecutively washed with water (5 x) and brine, dried over $MgSO_4$, filtered, and concentrated under reduced pressure. The residue was purified by flash column chromatography on silica gel (dry loading, 18 cm column height, gradient: pentane/EtOAc = 20:1 → 15:1), affording product **SI30** (673 mg, 1.50 mmol, 71% yield) as a yellow solid.

<u>(*R*)-6,6'-Diisopropoxy-3,3,3',3'-tetramethyl-2,2',3,3'-tetrahydro-1,1'-spirobi[indene]-7,7'-dicarbaldehyde</u> (**SI30**)

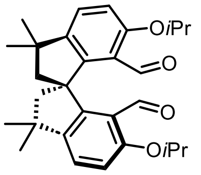

^{1}H NMR (400 MHz, $CDCl_3$) δ = 10.12 (s, 2H), 7.32 (d, *J* = 8.4 Hz, 2H), 6.87 (d, *J* = 8.5 Hz, 2H), 4.55 (hept, *J* = 6.0 Hz, 2H), 2.48 (d, *J* = 12.5 Hz, 2H), 2.28 (d, *J* = 12.5 Hz, 2H), 1.46 (s, 6H), 1.37 (s, 6H), 1.34 – 1.30 (m, 12H) ppm; **^{13}C{^{1}H} NMR** (101 MHz, $CDCl_3$) δ = 190.8, 160.7, 151.4, 146.5, 128.3, 121.1, 113.3, 71.8, 59.5, 57.3, 42.5, 32.8, 29.4, 22.3, 22.2 ppm; **IR** (ATR) ṽ = 2973 (w), 2939 (w), 2863 (w), 1690 (s), 1585 (m), 1469 (w), 1458 (w), 1422 (w), 1405 (w), 1384 (w), 1360 (w), 1299 (m), 1255 (w), 1191 (w), 1168 (w), 1136 (w), 1112 (m), 942

(w), 815 (w) cm^{-1}; **HRMS** (ESI/QTOF) *m/z* = calcd. for $[C_{29}H_{36}NaO_4]^+$, $[M+Na]^+$: 471.2506, found: 471.2508; **R$_f$** (pentane/EtOAc, 15:1) = 0.24; $[\alpha]_D^{21}$ = -414.7 (*c* = 0.5, $CHCl_3$).

Step 2 – Reduction. At 0 °C (ice bath) and under an atmosphere of nitrogen, sodium borohydride (227 mg, 6.00 mmol, 4 equiv.) was added portion-wise to a solution of **SI30** (673 mg, 1.50 mmol) in anhydrous methanol (5 mL/mmol) under a counterflow of nitrogen. After stirring for 10 min at 0 °C, the ice bath was removed, and the light-yellow reaction mixture was stirred at room temperature (25 °C) for 14 hours. Next, the reaction was quenched with sat. aq. NH_4Cl, diluted with water, and extracted with ethyl acetate (3 x). The combined organic layers were consecutively washed with water (2 x) and brine, dried over $MgSO_4$, filtered, and concentrated under reduced pressure. The resulting crude intermediate diol, obtained as a light-yellow foam, was immediately used in the next step without further purification.

Step 3 – Bromination. At 0 °C (ice bath) and under an atmosphere of nitrogen, phosphorus tribromide (141 µL, 1.50 mmol, 1 equiv.) was added dropwise to a solution of the crude chiral diol (assumed 1.50 mmol, 1 equiv.) in anhydrous THF (5 mL/mmol). After stirring for 10 min at 0 °C, the ice bath was removed, and the reaction mixture was stirred at room temperature (25 °C) for 3 hours. After cooling to 0 °C, the reaction was quenched with water and then extracted with ethyl acetate (3 x). The combined organic layers were sequentially washed with sat. aq. $NaHCO_3$ solution and brine, dried over $MgSO_4$, filtered, and concentrated under reduced pressure. The residue was purified by flash column chromatography on silica gel (dry loading, 18 cm column height, isocratic: pentane/EtOAc = 40:1), affording chiral dibromide **L8** (550 mg, 0.95 mmol, 63% yield over 2 steps) as a white foam.

(*R*)-7,7'-Bis(bromomethyl)-6,6'-diisopropoxy-3,3,3',3'-tetramethyl-2,2',3,3'-tetrahydro-1,1'-spirobi[indene] (**L8**)

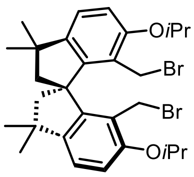

^{1}H NMR (400 MHz, $CDCl_3$) δ = 7.10 (d, *J* = 8.4 Hz, 2H), 6.81 (d, *J* = 8.5 Hz, 2H), 4.60 (hept, *J* = 6.1 Hz, 2H), 4.22 (d, *J* = 9.4 Hz, 2H), 3.87 (d, *J* = 9.3 Hz, 2H), 2.66 (d, *J* = 13.4 Hz, 2H), 2.33 (d, *J* = 13.4 Hz, 2H), 1.45 (s, 6H), 1.38 (d, *J* = 6.1 Hz, 6H), 1.36 – 1.32 (m, 12H) ppm; **^{13}C{^{1}H} NMR** (101 MHz, $CDCl_3$) δ = 156.8, 149.0, 144.0, 123.5, 122.7, 112.4, 70.4, 58.6, 56.6, 42.9, 32.9, 30.2, 26.6, 22.4, 22.3 ppm; **IR** (ATR) ṽ = 2954 (w), 2864 (w), 1588 (w), 1473 (m), 1383 (w), 1372 (w), 1362 (w), 1307 (w), 1262 (s), 1213 (w), 1196 (w), 1174 (w), 1139 (w), 1114 (m), 1009 (w), 950 (w), 909 (w), 813 (w), 734 (w), 548 (w) cm^{-1}; **HRMS** (APPI/LTQ-Orbitrap) *m/z* = calcd. for $[C_{29}H_{38}Br_2O_2]^+$, $[M]^+$: 576.1233, found: 576.1240; **R$_f$** (pentane/EtOAc, 40:1) = 0.32; $[\alpha]_D^{21}$ = +51.1 (*c* = 0.3, $CHCl_3$).

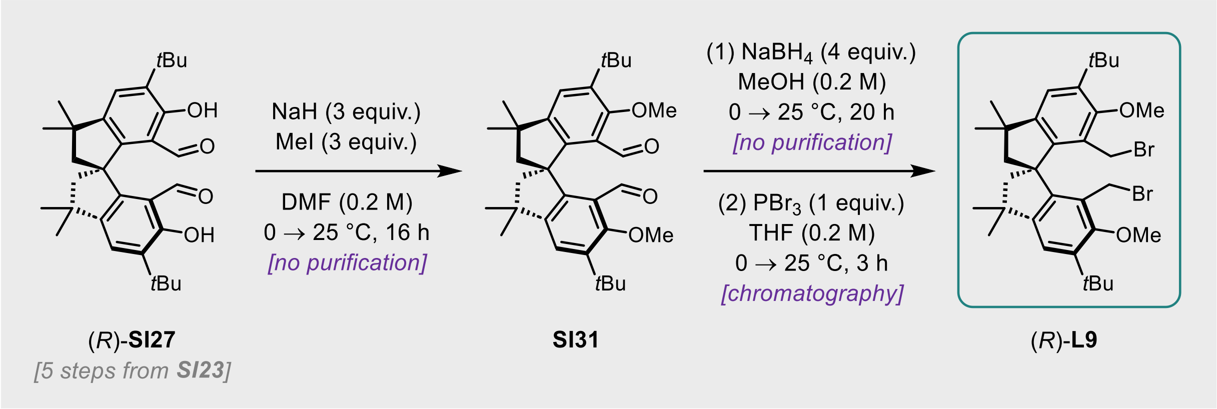

Scheme S10. Preparation of chiral dibromide (*R*)-**L9** in 8 steps from Bisphenol A **SI23**.

[9] Synthesis of Chiral Dibromide (R)-L9

Chiral dibromide (*R*)-**L9** was prepared in 3 additional steps from compound (*R*)-**SI27** according to the reported procedure (**Scheme S10**) with matching characterization data.[14]

For (*R*)-**SI31**:

^{1}H NMR (400 MHz, $CDCl_3$) δ = 9.88 (s, 2H), 7.33 (s, 2H), 3.70 (s, 6H), 2.41 (d, *J* = 2.7 Hz, 4H), 1.43 (s, 6H), 1.42 (s, 18H), 1.38 (s, 6H) ppm.

For (*R*)-**L9**:

^{1}H NMR (400 MHz, $CDCl_3$) δ = 7.15 (s, 2H), 4.26 (d, *J* = 10.3 Hz, 2H), 4.01 (d, *J* = 10.3 Hz, 2H), 3.87 (s, 6H), 2.61 (d, *J* = 13.5 Hz, 2H), 2.31 (d, *J* = 13.5 Hz, 2H), 1.45 – 1.40 (m, 24H), 1.34 (s, 6H) ppm.

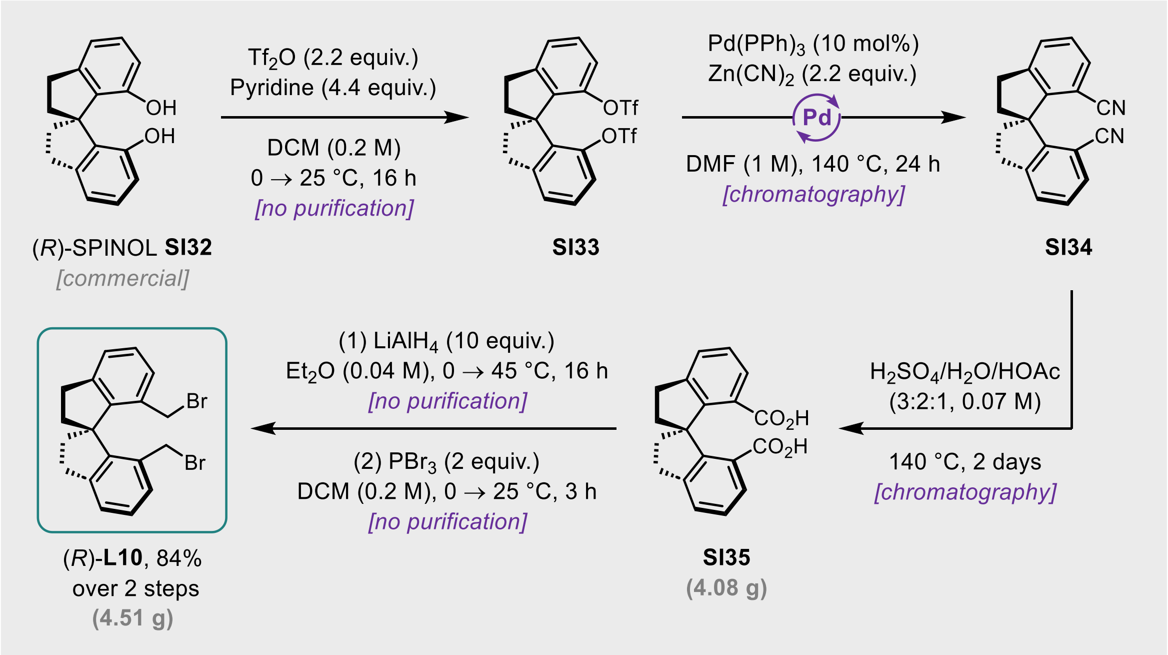

Scheme S11. Preparation of chiral dibromide (*R*)-**L10** in 5 steps from (*R*)-SPINOL **SI32**.

[10] Synthesis of Chiral Dibromide (R)-L10

Chiral dibromide (*R*)-**L10** was prepared in 2 additional steps from (*R*)-**SI35** according to the following procedure (**Scheme S11**). Diacid **SI35** itself was prepared in 3 steps from commercial (*R*)-SPINOL **SI32** (CAS 223259-62-9) according to the reported procedures.[19,20]

Step 1 – Reduction. Adapted from a reported procedure.[20] In a flame-dried 1 L two-necked round-bottom flask was made a solution of lithium aluminum hydride (4.0 M in Et_2O, 33.1 mL, 132.3 mmol, 10 equiv.) in anhydrous Et_2O (5 mL/mmol) under an atmosphere of nitrogen. At 0 °C (ice bath), a solution of chiral diacid **SI35** (4.08 g, 13.23 mmol) in anhydrous Et_2O (20 mL/mmol) was added dropwise over *ca.* 30 min *via* an addition funnel. The cold bath was removed, and the reaction mixture was refluxed (oil bath at 45 °C) for 16 hours. After cooling to 0 °C (ice bath), the reaction was quenched by dropwise addition of water (20 mL) with concomitant H_2 evolution, after which it was further diluted with water (15 mL/mmol) and then aq. HCl (6 M, 5 mL/mmol). After extraction with Et_2O (3 x), the combined organic layers were washed with brine, dried over $MgSO_4$, and filtered through a pad of silica gel (10 cm height) with Et_2O as eluent. All volatiles were removed *in vacuo*, affording the crude intermediate diol as a white foam with the characterization data matching those previously reported,[20] and in sufficient purity to be used in the next step without further purification.

^{1}H NMR (400 MHz, $CDCl_3$) δ = 7.33 – 7.21 (m, 6H), 4.26 (d, *J* = 11.9 Hz, 2H), 4.19 (d, *J* = 11.9 Hz, 2H), 3.06 – 2.99 (m, 4H), 2.38 (br s, 2H), 2.37 – 2.29 (m, 2H), 2.08 – 1.97 (m, 2H) ppm.

Step 2 – Bromination. In a flame-dried 250 mL round-bottom flask was made a solution of the crude chiral diol (assumed 13.23 mmol, 1 equiv.) in anhydrous DCM (5 mL/mmol) under an atmosphere of nitrogen. At 0 °C (ice bath), phosphorus tribromide (2.51 mL, 26.47 mmol, 2 equiv.) was added dropwise. After stirring for 1 hour at 0 °C, the cold bath was removed and the reaction mixture was stirred at room temperature (25 °C) for 2 hours. After cooling to 0 °C, the reaction was quenched with water and then extracted with DCM (3 x). The combined organic layers were sequentially washed with sat. aq. $NaHCO_3$ solution and brine. The colorless homogeneous solution was dried over $MgSO_4$ and then filtered through a pad of silica gel (10 cm height) with DCM as eluent. All volatiles were removed *in vacuo*, affording chiral dibromide **L10** (4.51 g, 11.1 mmol, 84% yield over 2 steps) as a white crystalline solid in excellent purity and with the characterization data matching those previously reported.[21]

^{1}H NMR (400 MHz, $CDCl_3$) δ = 7.29 – 7.21 (m, 6H), 4.13 (d, *J* = 10.3 Hz, 2H), 4.05 (d, *J* = 10.3 Hz, 2H), 3.09 – 3.02 (m, 4H), 2.42 – 2.28 (m, 4H) ppm.

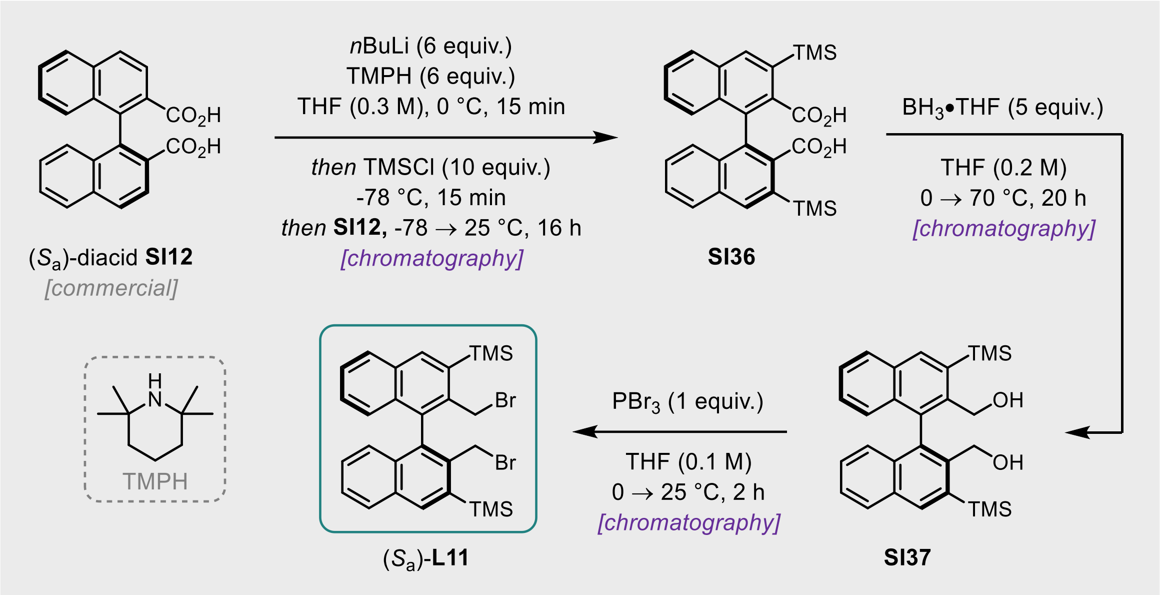

Scheme S12. Preparation of chiral dibromide (S_a)-**L11** in 3 steps from (S_a)-diacid **SI12**.

[11] Synthesis of Chiral Dibromide (S_a)-L11

Chiral dibromide (S_a)-**L11** was prepared in 3 steps from commercial diacid (S_a)-**SI12** (CAS 18531-96-9) according to the reported procedure (**Scheme S12**) with matching characterization data.[9]

For (S_a)-**SI36**:
^{1}H NMR (400 MHz, $CDCl_3$) δ = 8.21 (s, 2H), 7.90 (d, *J* = 8.2 Hz, 2H), 7.52 – 7.46 (m, 2H), 7.31 – 7.25 (m, 2H), 7.03 – 7.00 (m, 2H), 0.41 (s, 18H) ppm.

For (S_a)-**SI37**:
^{1}H NMR (400 MHz, $CDCl_3$) δ = 8.22 (s, 2H), 7.92 (d, *J* = 8.1 Hz, 2H), 7.48 – 7.42 (m, 2H), 7.25 – 7.21 (m, 2H), 6.96 – 6.92 (m, 2H), 4.57 (d, *J* = 11.6 Hz, 2H), 4.20 (d, *J* = 11.6 Hz, 2H), 0.49 (s, 18H) ppm.

For (S_a)-**L11**:
^{1}H NMR (400 MHz, $CDCl_3$) δ = 8.24 (s, 2H), 7.91 (d, *J* = 8.2 Hz, 2H), 7.50 – 7.45 (m, 2H), 7.26 – 7.20 (m, 2H), 7.01 (d, *J* = 8.5 Hz, 2H), 4.44 (d, *J* = 10.3 Hz, 2H), 4.34 (d, *J* = 10.2 Hz, 2H), 0.55 (s, 18H) ppm.

4. Synthesis of Chiral Cyclopentadienes

4.1 Classical Synthesis of Di- and Trisubstituted $Cp^{x}H$

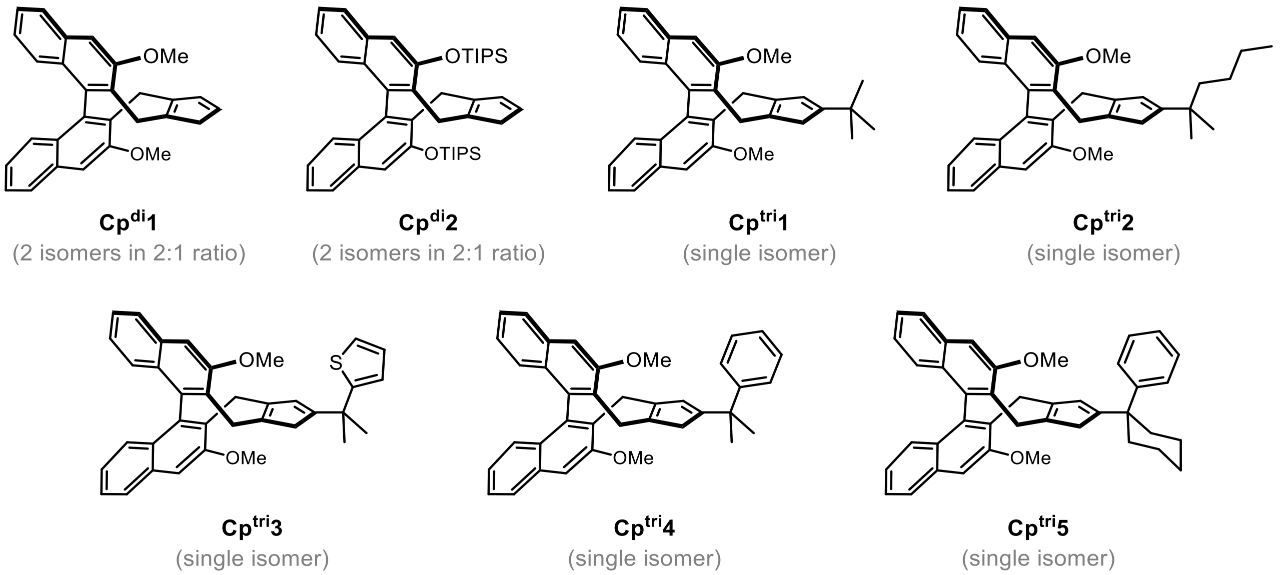

Figure S3. Overview of the di- and trisubstituted chiral cyclopentadienes ($Cp^{x}H$) used in this work.

All di- and trisubstituted chiral cyclopentadienes **Cp^{di}1-2** and **Cp^{tri}1-5** used in this work (**Figure S3**) were prepared according to the reported procedures, or adaptations thereof, as described *in infra*.

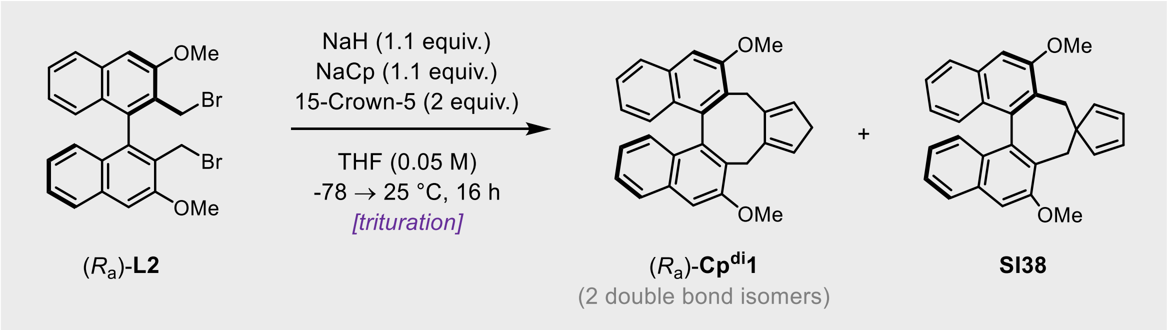

Scheme S13. Preparation of disubstituted chiral cyclopentadiene (R_a)-**Cp^{di}1** in 1 step from dibromide (R_a)-**L2**.

Disubstituted chiral cyclopentadiene (R_a)-**Cp^{di}1** was prepared in 1 step from dibromide (R_a)-**L2** according to a slight adaptation (**Scheme S13**) of the reported procedure,[4] including the use of 15-crown-5 ether as was reported for similar disubstituted $Cp^{x}H$ ligands,[9] and with matching characterization data. Notably, **Cp^{di}1** was conveniently obtained from the crude brown residue by trituration with cold ethyl acetate, which precipitates pure **Cp^{di}1** as a beige solid. Subsequent column chromatography of the mother liquor enabled separation of remaining **Cp^{di}1** from spirodiene **SI38**.

Disubstituted chiral cyclopentadiene (R_a)-**Cp^{di}2** was prepared in 3 additional steps from spirodiene **SI38** according to the reported procedure.[4]

For (R_a)-**Cp^{di}1**:
Obtained as an inseparable mixture of two double bond isomers (2:1 ratio). For ^{1}H NMR, the integration is calibrated with respect to the major isomer (and thus 0.5H integration per proton for the minor).
^{1}H NMR (400 MHz, C_6D_6) δ = 7.77 – 7.71 (m, 3H), 7.52 (d, *J* = 8.4 Hz, 1H), 7.46 (d, *J* = 8.4 Hz, 2H), 7.32 – 7.25 (m, 3H), 7.08 (s, 0.5H), 7.06 (s, 2H), 7.02 – 6.94 (m, 3.5H), 6.52 (d, *J* = 5.3 Hz, 0.5H), 6.18 – 6.12 (m, 2.5H), 4.38 (d, *J* = 13.9 Hz, 2H), 4.20 (d, *J* = 14.4 Hz, 0.5H), 4.06 (d, *J* = 14.5 Hz, 0.5H), 3.44 (s, 6H), 3.41 (s, 1.5H), 3.38 (s, 1.5H), 3.29 – 3.22 (m, 2H), 3.15 – 3.07 (m, 0.5H), 3.07 – 2.99 (m, 1H), 2.87 – 2.78 (m, 0.5H), 2.69 – 2.62 (m, 2H) ppm.

For (R_a)-**SI38**:
^{1}H NMR (400 MHz, C_6D_6) δ = 7.77 (d, *J* = 8.2 Hz, 2H), 7.61 (d, *J* = 8.4 Hz, 2H), 7.33 – 7.28 (m, 2H), 7.10 (s, 2H), 7.01 – 6.96 (m, 2H), 6.32 – 6.29 (m, 2H), 6.25 – 6.22 (m, 2H), 3.47 (s, 6H), 3.11 (d, *J* = 12.9 Hz, 2H), 2.61 (d, *J* = 12.9 Hz, 2H) ppm.

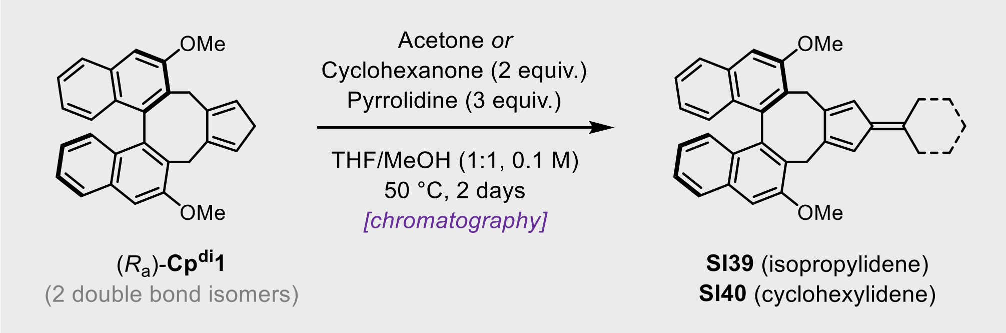

Scheme S14. Preparation of chiral fulvenes (R_a)-**SI39-40** from cyclopentadiene (R_a)-**Cp^{di}1**.

The chiral fulvenes **SI39-40** required for the synthesis of trisubstituted cyclopentadienes **Cp^{tri}1-5** were prepared in 1 step from (R_a)-**Cp^{di}1** according to a slight adaptation (**Scheme S14**) of the reported procedure with matching characterization data.[22]

For (R_a)-**SI39**:
^{1}H NMR (400 MHz, C_6D_6) δ = 7.75 (d, *J* = 8.2 Hz, 2H), 7.48 (d, *J* = 8.4 Hz, 2H), 7.32 – 7.27 (m, 2H), 7.07 (s, 2H), 7.01 – 6.96 (m, 2H), 6.38 (d, *J* = 1.6 Hz, 2H), 4.39 (d, *J* = 13.9 Hz, 2H), 3.44 (s, 6H), 3.38 (d, *J* = 14.0 Hz, 2H), 1.73 (s, 6H) ppm.

For (R_a)-**SI40**:
^{1}H NMR (400 MHz, C_6D_6) δ = 7.75 (d, *J* = 8.1 Hz, 2H), 7.49 (d, *J* = 8.5 Hz, 2H), 7.32 – 7.27 (m, 2H), 7.07 (s, 2H), 7.01 – 6.96 (m, 2H), 6.45 (d, *J* = 1.6 Hz, 2H), 4.42 (d, *J* = 14.0 Hz, 2H), 3.44 – 3.38 (m, 8H), 2.33 – 2.26 (m, 4H), 1.43 – 1.36 (m, 4H), 1.34 – 1.27 (m, 2H) ppm.

Subsequent nucleophilic addition of organolithium reagents onto these fulvenes (*General Procedure 1*, **Scheme S15**) delivered (R_a)-**Cp^{tri}1-5** as sole double bond isomers with the characterization data of **Cp^{tri}1** and **Cp^{tri}4** matching those previously reported.[22] Notably, **Cp^{tri}2-3** and **Cp^{tri}5** have not yet been reported, and their characterization data is provided *in infra*.

***General Procedure 1** – Addition onto Fulvenes toward Trisubstituted Cp^x Ligands.*

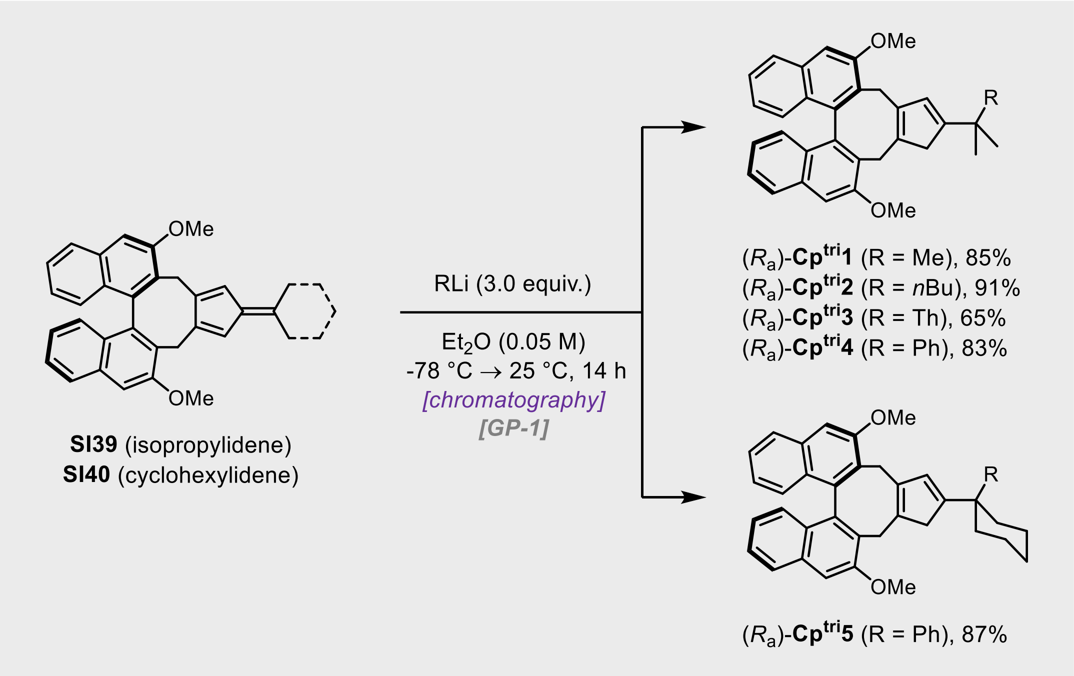

Scheme S15. Preparation of trisubstituted chiral cyclopentadienes (R_a)-**Cp^{tri}1-5** from fulvenes **SI39-40**.

In a flame-dried round-bottom flask was made a solution of fulvene **SI39** or **SI40** (1 equiv.) in anhydrous Et_2O (20 mL/mmol) under an atmosphere of nitrogen. At -78 °C (acetone/dry ice bath), the corresponding commercial organolithium reagent (3 equiv.) was added dropwise. The reaction mixture was allowed to slowly warm up in the cold bath to room temperature (25 °C) under stirring for 14 hours. Next, at 0 °C (ice bath), the reaction was quenched with sat. aq. NH_4Cl solution, diluted with water, and extracted with ethyl acetate (3 x). The combined organic layers were washed with brine, dried over $MgSO_4$, filtered, and concentrated under reduced pressure. The residue was purified by flash column chromatography on silica gel, affording the corresponding trisubstituted Cp^{x}H ligands **Cp^{tri}1-5**.

For (R_a)-**Cp^{tri}1**:
^{1}H NMR (400 MHz, C_6D_6) δ = 7.75 (d, *J* = 8.2 Hz, 2H), 7.52 (d, *J* = 8.4 Hz, 2H), 7.32 – 7.27 (m, 2H), 7.06 (d, *J* = 7.0 Hz, 2H), 7.03 – 6.97 (m, 2H), 6.15 (s, 1H), 4.21 (d, *J* = 13.9 Hz, 1H), 4.06 (d, *J* = 14.3 Hz, 1H), 3.39 (s, 3H), 3.38 (s, 3H), 3.14 – 3.03 (m, 3H), 2.86 – 2.77 (m, 1H), 1.08 (s, 9H) ppm.

For (R_a)-**Cp^{tri}4**:
^{1}H NMR (400 MHz, C_6D_6) δ = 7.76 – 7.71 (m, 2H), 7.52 – 7.47 (m, 2H), 7.31 – 7.26 (m, 2H), 7.26 – 7.21 (m, 2H), 7.15 – 7.12 (m, 2H), 7.08 (s, 1H), 7.08 – 7.03 (m, 1H), 7.02 (s, 1H), 7.01 – 6.95 (m, 2H), 6.25 (s, 1H), 4.22 (d, *J* = 14.2 Hz, 1H), 3.91 (d, *J* = 14.2 Hz, 1H), 3.43 (s, 3H), 3.34 (s, 3H), 3.09 – 2.98 (m, 2H), 2.98 – 2.90 (m, 1H), 2.77 – 2.67 (m, 1H), 1.44 (s, 3H), 1.41 (s, 3H) ppm.

(R_a)-5,16-Dimethoxy-2-(2-methylhexan-2-yl)-4,17-dihydro-1*H*-cyclopenta[6,7]cycloocta[2,1-*a*:3,4-*a*']dinaphthalene (**Cp^{tri}2**)

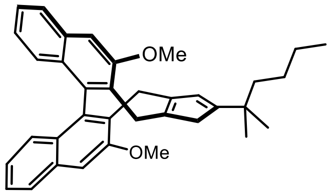

According to *General Procedure 1*, starting from fulvene **SI39** (135 µmol) with a *n*-butyllithium solution (1.6 M in hexanes), ligand (R_a)-**Cp^{tri}2** (62 mg, 123 µmol, 91% yield) was obtained as a white foam. Purification was performed by flash column chromatography on silica gel (dry loading, 15 cm column height, isocratic: pentane/EtOAc = 40:1). This trisubstituted cyclopentadiene exists as a sole double bond isomer.

^{1}H NMR (600 MHz, CD_2Cl_2) δ = 7.82 (dd, *J* = 8.2, 3.6 Hz, 2H), 7.40 – 7.35 (m, 2H), 7.31 (s, 1H), 7.29 (s, 1H), 7.11 – 7.07 (m, 4H), 6.00 (s, 1H), 3.99 (s, 3H), 3.98 (s, 3H), 3.94 (d, *J* = 14.6 Hz, 1H), 3.86 (d, *J* = 14.5 Hz, 1H), 3.03 – 2.94 (m, 2H), 2.73 (dd, *J* = 14.5, 3.7 Hz, 1H), 2.65 (dq, *J* = 14.6, 3.5 Hz, 1H), 1.47 – 1.37 (m, 2H), 1.27 (dp, *J* = 14.6, 7.0 Hz, 2H), 1.17 – 1.09 (m, 8H), 0.88 (t, *J* = 7.3 Hz, 3H) ppm; **^{13}C{^{1}H} NMR** (151 MHz, CD_2Cl_2) δ = 155.8, 155.7, 154.6, 137.6, 137.2, 136.7, 133.5, 133.4, 130.5, 128.5, 128.3, 127.63, 127.57, 127.2, 127.14, 127.06, 127.0, 125.9, 125.8, 123.9, 106.0, 105.9, 56.0, 55.8, 45.5, 43.7, 36.3, 28.8, 28.5, 27.4, 26.6, 26.5, 23.8, 14.3 ppm; **IR** (ATR) ṽ = 2955 (w), 2928 (w), 1595 (w), 1449 (w), 1421 (w), 1327 (w), 1293 (w), 1234 (w), 1218 (w), 1197 (m), 1162 (m), 1149 (w), 1110 (s), 1020 (m), 952 (m), 862 (w), 829 (m), 745 (s), 711 (s), 678 (w), 637 (w) cm^{-1}; **HRMS** (ESI-APCI/TOF) *m/z* = calcd. for $[C_{36}H_{39}O_2]^+$, $[M+H]^+$: 503.2945, found: 503.2948; **R$_f$** (pentane/EtOAc, 20:1) = 0.48; $[\alpha]_D^{23}$ = +239.9 (*c* = 1.3, CH_2Cl_2).

(R_a)-2-(2-(5,16-Dimethoxy-4,17-dihydro-1*H*-cyclopenta[6,7]cycloocta[2,1-*a*:3,4-*a*']dinaphthalen-2-yl)propan-2-yl)thiophene (**Cp^{tri}3**)

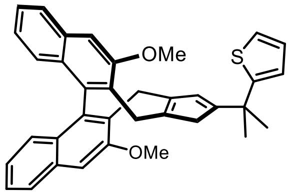

According to *General Procedure 1*, starting from fulvene **SI39** (540 µmol) with a 2-thienyllithium solution (1.0 M in THF/hexanes), ligand (R_a)-**Cp^{tri}3** (186 mg, 352 µmol, 65% yield) was obtained as a light-yellow foam. Purification was performed by flash column chromatography on silica gel (dry loading, 15 cm column height, isocratic: pentane/EtOAc = 20:1). This trisubstituted cyclopentadiene exists as a sole double bond isomer.

^{1}H NMR (600 MHz, CD_2Cl_2) δ = 7.81 (dd, *J* = 8.2, 3.4 Hz, 2H), 7.38 – 7.35 (m, 2H), 7.30 (s, 1H), 7.28 (s, 1H), 7.12 (dd, *J* = 5.1, 1.2 Hz, 1H), 7.10 – 7.05 (m, 4H), 6.89 (dd, *J* = 5.1, 3.5 Hz, 1H), 6.80 (dd, *J* = 3.5, 1.2 Hz, 1H), 6.11 (s, 1H), 4.00 (s, 3H), 3.95 (s, 3H), 3.92 (d, *J* = 14.6 Hz, 1H), 3.81 (d, *J* = 14.5 Hz, 1H), 3.00 (dd, *J* = 23.1, 3.0 Hz, 1H), 2.94 (dd, *J* = 23.3, 3.1 Hz, 1H), 2.70 (dd, *J* = 14.5, 3.8 Hz, 1H), 2.64 (dq, *J* = 14.6, 3.4 Hz, 1H), 1.61 (s, 6H) ppm; **^{13}C{^{1}H} NMR** (151 MHz, CD_2Cl_2) δ = 156.6, 155.8, 155.7, 153.4, 138.0, 137.4, 137.2, 136.8, 133.53, 133.49, 131.3, 128.3, 128.2, 127.62, 127.58, 127.2, 127.15, 127.06, 127.0, 126.7, 125.92, 125.87, 123.9, 123.2, 122.7, 106.1, 106.0, 56.0, 55.8, 45.9, 39.7, 31.3, 26.6, 26.5 ppm; **IR** (ATR) ṽ = 2961 (w), 1619 (w), 1595 (w), 1449 (w), 1421 (w), 1409 (w), 1391 (w), 1339 (w), 1327 (w), 1294 (w), 1234 (w), 1218 (w), 1197 (m), 1162 (m), 1150 (w), 1110 (s), 1020 (m), 952 (m), 863 (w), 830 (m), 746 (m), 711 (s), 678 (w), 637 (w), 622 (w) cm^{-1}; **HRMS** (ESI-APCI/TOF) *m/z* = calcd. for $[C_{36}H_{33}O_2S]^+$, $[M+H]^+$: 529.2196, found: 529.2197; **R$_f$** (pentane/EtOAc, 20:1) = 0.33; $[\alpha]_D^{23}$ = +279.3 (*c* = 0.7, CH_2Cl_2).

(*R*$_a$)-5,16-Dimethoxy-2-(1-phenylcyclohexyl)-4,17-dihydro-1*H*-cyclopenta[6,7]cycloocta[2,1-*a*:3,4-*a*']dinaphthalene (**Cp^{tri}5**)

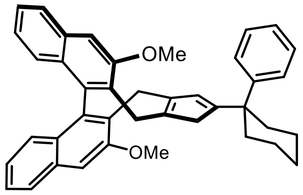

According to *General Procedure 1*, starting from fulvene **SI40** (127 µmol) with a phenyllithium solution (1.9 M in *n*Bu_2O), ligand **L11** (63 mg, 111 µmol, 87% yield) was obtained as a white foam. Purification was performed by flash column chromatography on silica gel (dry loading, 15 cm column height, isocratic: pentane/EtOAc = 40:1). This trisubstituted cyclopentadiene exists as a sole double bond isomer.

^{1}H NMR (600 MHz, CD_2Cl_2) δ = 7.82 – 7.79 (m, 2H), 7.38 – 7.34 (m, 2H), 7.33 – 7.30 (m, 2H), 7.30 – 7.26 (m, 4H), 7.15 – 7.12 (m, 1H), 7.10 – 7.05 (m, 4H), 6.09 (s, 1H), 3.99 (s, 3H), 3.94 – 3.90 (m, 4H), 3.76 (d, *J* = 14.6 Hz, 1H), 2.83 (dd, *J* = 23.1, 3.1 Hz, 1H), 2.73 (dd, *J* = 23.2, 2.9 Hz, 1H), 2.67 – 2.62 (m, 2H), 2.20 – 2.09 (m, 2H), 2.06 – 1.97 (m, 2H), 1.61 – 1.53 (m, 2H), 1.52 – 1.41 (m, 4H) ppm; **^{13}C{^{1}H} NMR** (151 MHz, CD_2Cl_2) δ = 155.80, 155.78, 155.7, 137.6, 137.1, 136.8, 133.5, 133.44, 133.42, 132.1, 130.65, 130.61, 128.5, 128.33, 128.27, 127.95, 127.93, 127.58, 127.55, 127.13, 127.05, 127.03, 127.01, 126.3, 126.1, 125.9, 125.8, 125.6, 123.91, 123.89, 106.0, 105.9, 56.0, 55.8, 45.6, 44.8, 37.0, 36.9, 26.8, 26.59, 26.55, 23.29, 23.25 ppm; **IR** (ATR) ṽ = 2932 (w), 2854 (w), 1620 (w), 1595 (w), 1449 (m), 1421 (w), 1410 (w), 1338 (w), 1327 (w), 1234 (w), 1219 (w), 1197 (w), 1163 (m), 1150 (w), 1110 (s), 862 (w), 830 (w), 761 (w), 746 (m), 709 (m) cm^{-1}; **HRMS** (ESI-APCI/TOF) *m/z* = calcd. for $[C_{41}H_{39}O_2]^+$, $[M+H]^+$: 563.2945, found: 563.2946; **R$_f$** (pentane/EtOAc, 20:1) = 0.24; $[\alpha]_D^{23}$ = +202.6 (*c* = 0.6, CH_2Cl_2).

4.2 Convergent Modular Synthesis of Pentasubstituted Cp^{V}H

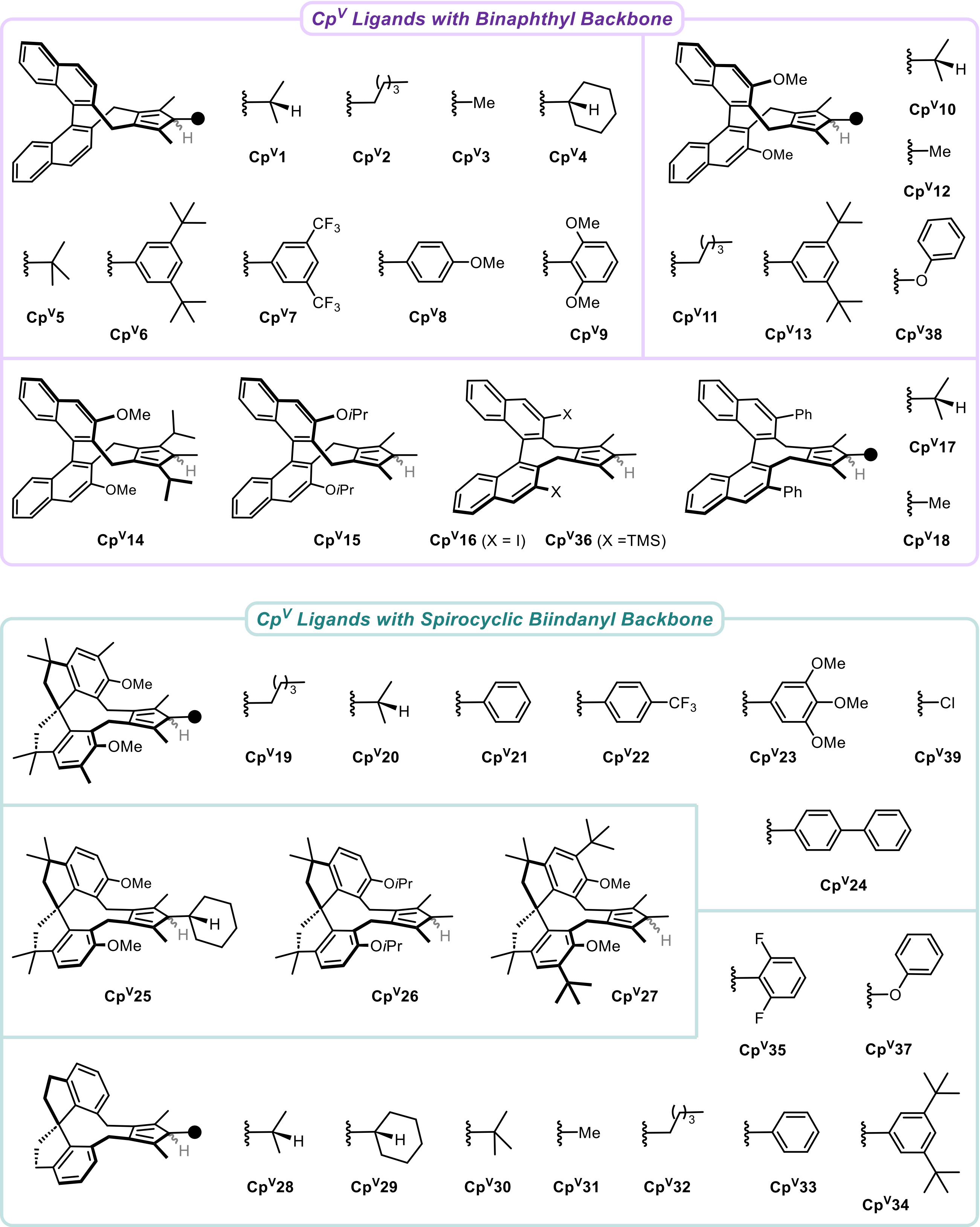

Figure S4. Overview of the pentasubstituted chiral cyclopentadiene isomers (Cp^{V}H) prepared in this work.

***General Procedure 2* – Dialkylation of 1,2,3-Cps toward Pentasubstituted Cp^V Ligands.**

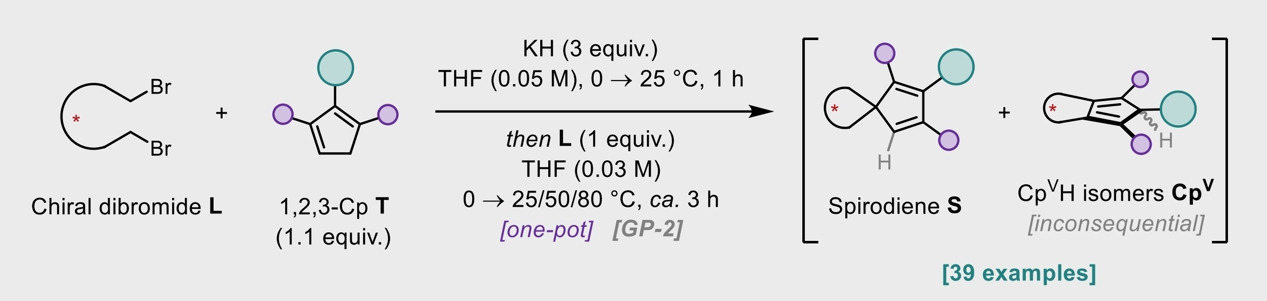

Scheme S16. One-pot deprotonation-dialkylation of 1,2,3-Cps toward pentasubstituted chiral cyclopentadienes **Cp^V**.

In a nitrogen-filled glovebox, an oven-dried microwave vial was charged with potassium hydride (deoiled, 3 equiv.) and capped. Outside of the glovebox, the vial was connected to a Schlenk line and anhydrous THF (10 mL/mmol) was added. At 0 °C (ice bath), a solution of 1,2,3-trisubstituted cyclopentadiene **T** (1.1 equiv.) in anhydrous THF (10 mL/mmol) was added. The ice bath was removed, and the suspension was allowed to warm up to room temperature (25 °C) under vigorous stirring for 1 hour, slowly turning darker with concomitant H_2 evolution. Next, the resulting potassium cyclopentadienide solution was cooled back to 0 °C (ice bath), and a solution of chiral dibromide **L** (1 equiv.) in anhydrous THF (10 mL/mmol) was added. The ice bath was removed, and the reaction mixture was stirred for ≥3 hours in a heating block at 50 °C. Alternatively, it was stirred overnight at 25 °C, or for ≥1.5 hours at 80 °C. After cooling to room temperature (25 °C), the brown reaction mixture was carefully quenched with sat. aq. NH_4Cl solution, then diluted with water, after which it was extracted with Et_2O (3 x). The combined organic layers were washed with brine, dried over $MgSO_4$, filtered, and concentrated under reduced pressure. The residue was purified by flash column chromatography on silica gel, affording spirodiene **S** and pentasubstituted chiral cyclopentadiene **Cp^V** as a complex mixture of isomers. The **Cp^V/S** ratio was determined by qNMR analysis in $CDCl_3$ *via* the spirodiene's characteristic olefinic proton, using 1,3,5-trimethoxybenzene or ethylene carbonate as an internal standard. Afterwards, the **Cp^V/S** mixture was directly engaged in the subsequent complexation step without further purification.

Note 1: Regarding the choice of internal standard, it is suggested to use 1,3,5-trimethoxybenzene before rhodium or iridium complexations, and ethylene carbonate before cobalt complexation, which then facilitates its removal (polarity-wise) during chromatographic purification of the complexes.

Note 2: Most **Cp^V** products could easily be visualized during TLC analysis with a vanillin stain and subsequent heating, generating a strong dark blue spot. Alternatively, a PMA stain also worked well, especially for spirobiindanyl-based systems.

Note 3: Formation of spirobiindanyl-based cyclopentadienes **Cp^{V}19-35** (containing a fused 9-membered ring) was strongly favored over their respective 8-ring spirodiene homologues **S19-35** (*avg.* 95:5 ratio). In contrast, dialkylation towards binaphthyl-based cyclopentadienes **Cp^{V}1-18** (containing a fused 8-membered ring) generally showed a less pronounced preference over their 7-ring spirodiene homologues **S1-18** (*avg.* 67:33 ratio).

Note 4: While the often-complex Cp^VH isomeric mixtures complicate spectroscopic analysis and characterization, such isomerism is completely inconsequential for the subsequent complexation step, since η^5-coordination to a metal makes all Cp^VH isomers converge to a single anionic Cp^V ligand. Full characterizations were therefore performed at the metal (Co, Rh, Ir) complex stage.

Note 5: Separation of the spirodiene **S** from the $\mathbf{Cp^V}$ isomers was feasible (as illustrated for **S8**), but this was not necessary for the subsequent complexation step (see **Sections 5.2**, **5.3** and **5.4**). When using the mild cobalt, rhodium(I), or iridium(I) complexation procedures, the spirodienes were innocent and solely their Cp^VH structural isomers reacted, hereby allowing their recovery afterwards if desired (as illustrated for **S1**). On the other hand, when using the direct rhodium(III) complexation protocol (with $RhCl_3 \cdot 3\ H_2O$ at 140 °C), the spirodienes did react through a Rh-mediated rearrangement-complexation cascade toward the desired Cp^VRh complexes, hereby aiding the efficiency of Cp^VRh synthesis (see **Section 5.5**).

Pentasubstituted cyclopentadiene isomers (R_a)-$\mathbf{Cp^V1}$ and Spirodiene (R_a)-**S1**

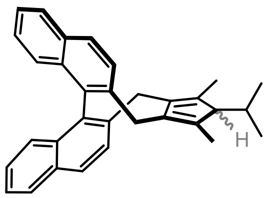

$\mathbf{Cp^V1}$ (isomers)

+

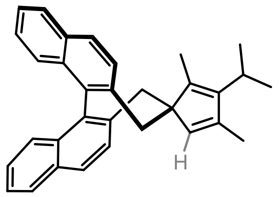

S1 (Cp/spiro = 56:44)

According to *General Procedure 2*, starting from chiral dibromide (R_a)-**L1** (0.65 mmol) and 1,2,3-Cp **T1** (1.1 equiv.) at 50 °C for 3 hours, a complex mixture of Cp^VH isomers $\mathbf{Cp^V1}$ and spirodiene **S1** (259 mg, 0.62 mmol, 96% yield, 56:44 ratio) was obtained as a light-yellow solid. Purification was performed by flash column chromatography on silica gel (dry loading, 15 cm column height, gradient: pentane/EtOAc = 100:0 → 70:1).

The characteristic ^1H NMR signal (400 MHz, $CDCl_3$) of spirodiene **S1** is located at δ = 5.67 (q, *J* = 1.6 Hz, 1H) ppm. **HRMS** (ESI/QTOF) *m/z* = calcd. for $[C_{32}H_{31}]^+$, $[M+H]^+$: 415.2420, found: 415.2422; $\mathbf{R_f}$ (pentane/EtOAc, 100:1) = 0.26.

Notably, spirodiene (−)-(R_a)-**S1** could be isolated as a white foam through Rh(I) complexation of the $\mathbf{Cp^V1}$ isomers toward **Rh1b** according to the first step of *General Procedure 4b* (see **Section 5.3**) and subsequent separation by Prep. TLC on silica (pentane/EtOAc = 50:1).

For spirodiene (R_a)-**S1**:
$\mathbf{^1H}$ **NMR** (600 MHz, CD_2Cl_2) δ = 7.96 – 7.91 (m, 3H), 7.89 (d, *J* = 8.3 Hz, 1H), 7.55 – 7.52 (m, 2H), 7.44 – 7.39 (m, 2H), 7.33 – 7.30 (m, 1H), 7.25 – 7.17 (m, 3H), 5.65 (q, *J* = 1.6 Hz, 1H), 2.89 (d, *J* = 12.6 Hz, 1H), 2.87 – 2.78 (m, 2H), 2.19 (d, *J* = 13.6 Hz, 1H), 2.01 (d, *J* = 12.5 Hz, 1H), 1.96 (d, *J* = 1.6 Hz, 3H), 1.61 (s, 3H), 1.20 – 1.17 (m, 6H) ppm; $\mathbf{^{13}C\{^1H\}}$ **NMR** (151 MHz, CD_2Cl_2) δ = 143.8, 142.7, 140.0, 138.6, 138.0, 137.6, 134.5, 133.8, 133.2, 133.1, 132.5, 132.3, 129.7, 128.61, 128.57, 128.5, 128.3, 127.5, 127.4, 127.3, 125.8, 125.2, 125.1, 66.2, 39.2, 38.0, 26.7, 21.8, 21.7, 16.1, 12.0 ppm; **IR** (ATR) ṽ = 2958 (w), 2929 (w), 1508 (w), 1462 (w), 1441 (w), 1361 (w), 1215 (m), 1027 (w), 836 (w), 815 (w), 790 (m), 747 (w), 667 (s), 625 (m), 573 (w), 520 (w), 416 (w) cm^{-1}; **HRMS** (ESI/QTOF) *m/z* = calcd. for $[C_{32}H_{31}]^+$, $[M+H]^+$: 415.2420, found: 415.2416; $\mathbf{R_f}$ (pentane/EtOAc, 100:1) = 0.26; $[\alpha]_D^{22}$ = -163.3 (*c* = 0.5, $CHCl_3$).

Pentasubstituted cyclopentadiene isomers (R_a)-**Cp^{V}2** and Spirodiene (R_a)-**S2**

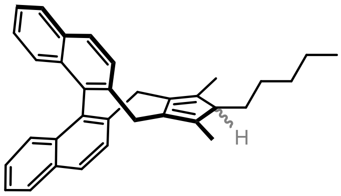

Cp^{V}2 (isomers)

+

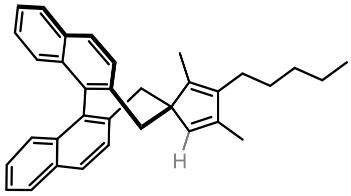

S2 (Cp/spiro = 70:30)

According to *General Procedure 2*, starting from chiral dibromide (R_a)-**L1** (1.27 mmol) and 1,2,3-Cp **T2** (1.1 equiv.) at 50 °C for 3 hours, a complex mixture of Cp^{V}H isomers **Cp^{V}2** and spirodiene **S2** (411 mg, 0.93 mmol, 73% yield, 70:30 ratio) was obtained as a light-yellow foam. Purification was performed by flash column chromatography on silica gel (wet loading with pentane/DCM = 20:1, 10 cm column height, gradient: pentane/EtOAc = 100:0 → 30:1).

The characteristic ^{1}H NMR signal (400 MHz, $CDCl_3$) of spirodiene **S2** is located at δ = 5.70 (q, *J* = 1.8 Hz, 1H) ppm. **HRMS** (Sicrit plasma/LTQ-Orbitrap) *m/z* = calcd. for $[C_{34}H_{35}]^+$, $[M+H]^+$: 443.2733, found: 443.2729; **R$_f$** (pentane/EtOAc, 40:1) = 0.32.

Pentasubstituted cyclopentadiene isomers (R_a)-**Cp^{V}3** and Spirodiene (R_a)-**S3**

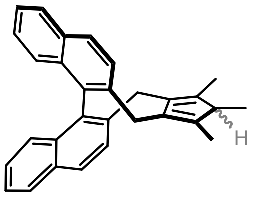

Cp^{V}3 (isomers)

+

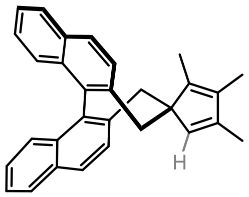

S3 (Cp/spiro = 94:6)

According to *General Procedure 2*, starting from chiral dibromide (R_a)-**L1** (1.08 mmol) and 1,2,3-Cp **T3** (1.1 equiv.) at 50 °C for 3 hours, a complex mixture of Cp^{V}H isomers **Cp^{V}3** and spirodiene **S3** (404 mg, 1.05 mmol, 96% yield, 94:6 ratio) was obtained as a light-yellow foam. Purification was performed by flash column chromatography on silica gel (wet loading with pentane, 15 cm column height, gradient: pentane/EtOAc = 100:0 → 50:1). Notably, this dialkylation allows an expedient synthesis of **Cp^{V}3** (1 step from **L1**, 90% yield of Cp^{V}H isomers) compared to the literature protocol (7 steps from **L1**, 27% overall yield).[23,24]

The characteristic ^{1}H NMR signal (400 MHz, $CDCl_3$) of spirodiene **S3** is located at δ = 5.71 (q, *J* = 1.8 Hz, 1H) ppm. **HRMS** (ESI/QTOF) *m/z* = calcd. for $[C_{30}H_{27}]^+$, $[M+H]^+$: 387.2107, found: 387.2104; **R$_f$** (pentane/EtOAc, 50:1) = 0.25.

Pentasubstituted cyclopentadiene isomers (R_a)-**Cp^{V}4** and Spirodiene (R_a)-**S4**

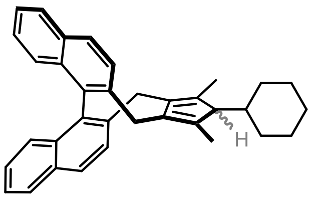

Cp^{V}4 (isomers)

+

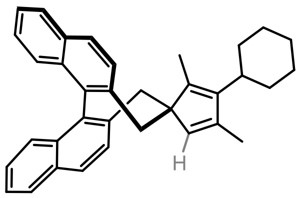

S4 (Cp/spiro = 60:40)

According to *General Procedure 2*, starting from chiral dibromide (R_a)-**L1** (1.36 mmol) and 1,2,3-Cp **T4** (1.1 equiv.) at 50 °C for 3 hours, a complex mixture of Cp^{V}H isomers **Cp^{V}4** and spirodiene **S4** (613 mg, 1.35 mmol, 99% yield, 60:40 ratio) was obtained as a light-yellow solid. Purification was performed by flash column chromatography on silica gel (dry loading, 15 cm column height, gradient: pentane/EtOAc = 100:0 → 80:1).

The characteristic ^{1}H NMR signal (400 MHz, $CDCl_3$) of spirodiene **S4** is located at δ = 5.67 (q, *J* = 1.8 Hz, 1H) ppm. **HRMS** (Sicrit plasma/LTQ-Orbitrap) *m/z* = calcd. for $[C_{35}H_{35}]^+$, $[M+H]^+$: 455.2733, found: 455.2733; **R$_f$** (pentane/EtOAc, 100:1) = 0.34.

Pentasubstituted cyclopentadiene isomers (R_a)-**Cp^{V}5** and Spirodiene (R_a)-**S5**

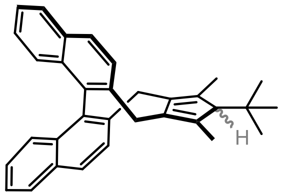

Cp^{V}5 (isomers)

+

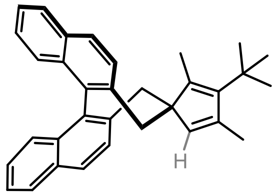

S5 (Cp/spiro = 51:49)

According to *General Procedure 2*, starting from chiral dibromide (R_a)-**L1** (451 µmol) and 1,2,3-Cp **T5** (1.1 equiv.) at 50 °C for 3 hours, a complex mixture of Cp^{V}H isomers **Cp^{V}5** and spirodiene **S5** (192 mg, 447 µmol, 99% yield, 51:49 ratio) was obtained as a light-yellow foam. Purification was performed by flash column chromatography on silica gel (wet loading with pentane, 10 cm column height, gradient: pentane/EtOAc = 100:0 → 50:1).

The characteristic ^{1}H NMR signal (400 MHz, $CDCl_3$) of spirodiene **S5** is located at δ = 5.67 (q, *J* = 1.6 Hz, 1H) ppm. **HRMS** (Sicrit plasma/LTQ-Orbitrap) *m/z* = calcd. for $[C_{33}H_{33}]^+$, $[M+H]^+$: 429.2577, found: 429.2577; **R$_f$** (pentane/EtOAc, 100:1) = 0.34.

Pentasubstituted cyclopentadiene isomers (R_a)-**Cp^{V}6** and Spirodiene (R_a)-**S6**

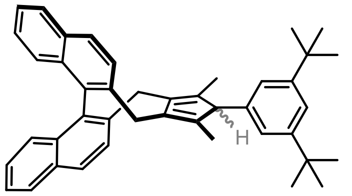

Cp^{V}6 (isomers)

+

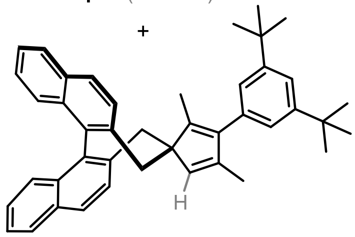

S6 (Cp/spiro = 77:23)

According to *General Procedure 2*, starting from chiral dibromide (R_a)-**L1** (0.77 mmol) and 1,2,3-Cp **T11** (1.1 equiv.) at 50 °C for 3 hours, a complex mixture of Cp^{V}H isomers **Cp^{V}6** and spirodiene **S6** (424 mg, 0.76 mmol, 99% yield, 77:23 ratio) was obtained as a light-yellow foam. Purification was performed by flash column chromatography on silica gel (dry loading, 18 cm column height, gradient: pentane/EtOAc = 100:0 → 50:1).

The characteristic ^{1}H NMR signal (400 MHz, $CDCl_3$) of spirodiene **S6** is located at δ = 5.84 (q, *J* = 1.8 Hz, 1H) ppm. **HRMS** (nanochip-ESI/LTQ-Orbitrap) *m/z* = calcd. for $[C_{43}H_{45}]^+$, $[M+H]^+$: 561.3516, found: 561.3515; **R$_f$** (pentane/EtOAc, 100:1) = 0.31.

Pentasubstituted cyclopentadiene isomers (R_a)-**Cp^{V}7** and Spirodiene (R_a)-**S7**

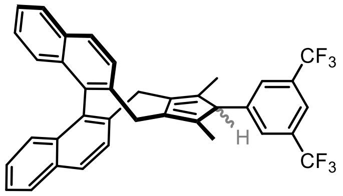

Cp^{V}7 (isomers)

+

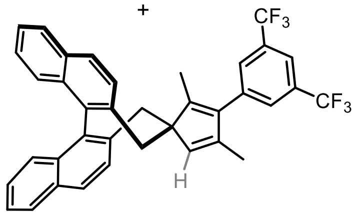

S7 (Cp/spiro = 87:13)

According to *General Procedure 2*, starting from chiral dibromide (R_a)-**L1** (338 µmol) and 1,2,3-Cp **T12** (1.1 equiv.) at 50 °C for 3 hours, a complex mixture of Cp^{V}H isomers **Cp^{V}7** and spirodiene **S8** (182 mg, 311 µmol, 92% yield, 87:13 ratio) was obtained as a light-yellow foam. Purification was performed by flash column chromatography on silica gel (dry loading, 15 cm column height, gradient: pentane/EtOAc = 100:0 → 50:1).

The characteristic ^{1}H NMR signal (400 MHz, $CDCl_3$) of spirodiene **S7** is located at δ = 5.88 (q, *J* = 1.8 Hz, 1H) ppm. **HRMS** (ESI/QTOF) *m/z* = calcd. for $[C_{37}H_{27}F_6]^+$, $[M+H]^+$: 585.2011, found: 585.2010; **R$_f$** (pentane/EtOAc, 100:1) = 0.34.

Pentasubstituted cyclopentadiene isomers (R_a)-**Cp^{V}8** and Spirodiene (R_a)-**S8**

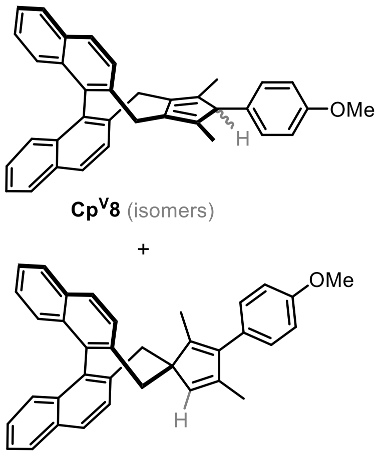

Cp^{V}8 (isomers)

S8 (Cp/spiro = 72:28)

According to *General Procedure 2*, starting from chiral dibromide (R_a)-**L1** (260 µmol) and 1,2,3-Cp **T7** (1.1 equiv.) at 50 °C for 3 hours, a complex mixture of Cp^{V}H isomers **Cp^{V}8** and spirodiene **S8** (87 mg, 182 µmol, 70% yield, 72:28 ratio) was obtained as a light-yellow foam. Purification was performed by flash column chromatography on silica gel (dry loading, 15 cm column height, gradient: pentane/EtOAc = 100:0 → 30:1).

The characteristic ^{1}H NMR signal (400 MHz, $CDCl_3$) of spirodiene **S8** is located at δ = 5.79 (q, *J* = 1.8 Hz, 1H) ppm. **HRMS** (nanochip-ESI/LTQ-Orbitrap) *m/z* = calcd. for $[C_{36}H_{31}O]^+$, $[M+H]^+$: 479.2369, found: 479.2367; **R$_f$** (pentane/EtOAc, 50:1) = 0.33.

Notably, spirodiene (−)-(R_a)-**S8** could be separated from (+)-(R_a)-**Cp^{V}8** by two consecutive reversed-phase flash column chromatographies on C_{18}-coated silica gel (dry loading, gradient: H_2O/MeCN = 20:80 → 0:100), and was obtained as a light-yellow foam.

For spirodiene (R_a)-**S8**:
^{1}H NMR (400 MHz, CD_2Cl_2) δ = 8.00 – 7.93 (m, 3H), 7.90 (d, *J* = 8.3 Hz, 1H), 7.58 (dd, *J* = 8.3, 4.7 Hz, 2H), 7.46 – 7.40 (m, 2H), 7.36 – 7.32 (m, 1H), 7.27 – 7.20 (m, 3H), 7.20 – 7.13 (m, 2H), 6.96 – 6.91 (m, 2H), 5.82 – 5.77 (m, 1H), 3.82 (s, 3H), 2.97 (d, *J* = 12.4 Hz, 1H), 2.91 (d, *J* = 13.6 Hz, 1H), 2.35 (d, *J* = 13.6 Hz, 1H), 2.18 (d, *J* = 12.4 Hz, 1H), 1.81 (d, *J* = 1.6 Hz, 3H), 1.62 (s, 3H) ppm; **^{13}C{^{1}H} NMR** (101 MHz, CD_2Cl_2) δ = 158.8, 147.1, 139.6, 139.5, 138.3, 137.6, 136.7, 134.6, 133.9, 133.23, 133.16, 132.5, 132.3, 130.8, 129.6, 129.1, 128.6, 128.55, 128.51, 128.4, 127.6, 127.5, 127.3, 125.9, 125.3, 125.2, 113.8, 67.0, 55.6, 39.3, 37.8, 15.1, 12.7 ppm; **IR** (ATR) ṽ = 3048 (w), 2925 (w), 2852 (w), 1607 (w), 1509 (s), 1441 (w), 1286 (w), 1245 (s), 1174 (w), 1107 (w), 1034 (w), 975 (w), 865 (w), 834 (m), 816 (m), 749 (m), 738 (w), 669 (w), 625 (w), 553 (w) cm^{-1}; **HRMS** (ESI/APCI) *m/z* = calcd. for $[C_{36}H_{31}O]^+$, $[M+H]^+$: 479.2369, found: 479.2370; **R$_f$** (pentane/EtOAc, 50:1) = 0.33; $[\alpha]_D^{23}$ = -73.3 (*c* = 0.6, CH_2Cl_2).

Pentasubstituted cyclopentadiene isomers (*R*a)-**Cp^V9** and Spirodiene (*R*a)-**S9**

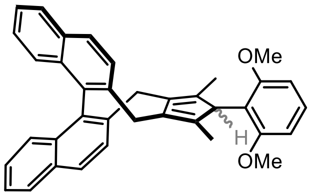

Cp^V9 (isomers)

\+

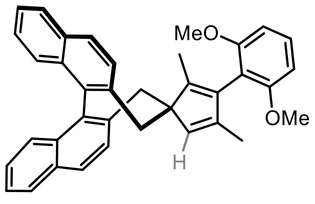

S9 (Cp/spiro = 58:42)

According to *General Procedure 2*, starting from chiral dibromide (*R*a)-**L1** (1.18 mmol) and 1,2,3-Cp **T13** (1.1 equiv.) at 50 °C for 3 hours, a complex mixture of Cp^VH isomers **Cp^V9** and spirodiene **S9** (571 mg, 1.12 mmol, 95% yield, 58:42 ratio) was obtained as a white foam. Purification was performed by flash column chromatography on silica gel (dry loading, 18 cm column height, gradient: pentane/EtOAc = 30:1 → 15:1).

The characteristic ^{1}H NMR signal (400 MHz, $CDCl_3$) of spirodiene **S9** is located at δ = 5.79 (q, *J* = 1.8 Hz, 1H) ppm. **HRMS** (Sicrit plasma/LTQ-Orbitrap) *m/z* = calcd. for $[C_{37}H_{33}O_2]^+$, $[M+H]^+$: 509.2475, found: 509.2474; **R$_f$** (pentane/EtOAc, 20:1) = 0.25.

Pentasubstituted cyclopentadiene isomers (*R*a)-**Cp^V10** and Spirodiene (*R*a)-**S10**

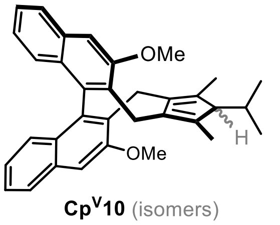

Cp^V10 (isomers)

\+

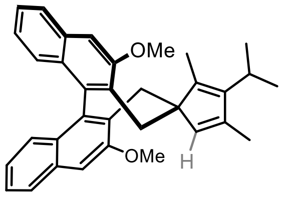

S10 (Cp/spiro = 45:55)

According to *General Procedure 2*, starting from chiral dibromide (*R*a)-**L2** (0.82 mmol) and 1,2,3-Cp **T1** (1.1 equiv.) at 25 °C for 15 hours, a complex mixture of Cp^VH isomers **Cp^V10** and spirodiene **S10** (303 mg, 0.64 mmol, 78% yield, 45:55 ratio) was obtained as a light-yellow solid. Purification was performed by flash column chromatography on silica gel (dry loading, 15 cm column height, gradient: pentane/EtOAc = 100:0 → 30:1).

The characteristic ^{1}H NMR signal (400 MHz, $CDCl_3$) of spirodiene **S10** is located at δ = 5.57 (q, *J* = 1.8 Hz, 1H) ppm. **HRMS** (Sicrit plasma/LTQ-Orbitrap) *m/z* = calcd. for $[C_{34}H_{35}O_2]^+$, $[M+H]^+$: 475.2632, found: 475.2630; **R$_f$** (pentane/EtOAc, 50:1) = 0.24.

Pentasubstituted cyclopentadiene isomers (*R*a)-**Cp^V11** and Spirodiene (*R*a)-**S11**

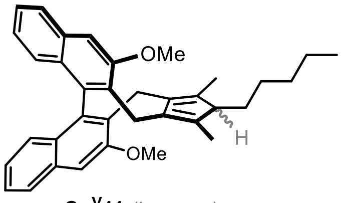

Cp^V11 (isomers)

\+

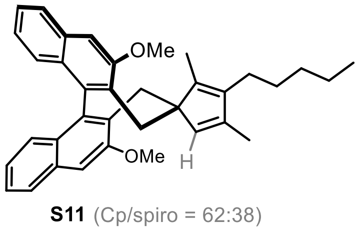

S11 (Cp/spiro = 62:38)

According to *General Procedure 2*, starting from chiral dibromide (*R*a)-**L2** (563 µmol) and 1,2,3-Cp **T2** (1.1 equiv.) at 25 °C for 24 hours, a complex mixture of Cp^VH isomers **Cp^V11** and spirodiene **S11** (141 mg, 280 µmol, 50% yield, 62:38 ratio) was obtained as a light-yellow foam. Purification was performed by flash column chromatography on silica gel (dry loading, 15 cm column height, gradient: pentane/EtOAc = 100:0 → 30:1).

The characteristic ^{1}H NMR signal (400 MHz, $CDCl_3$) of spirodiene **S11** is located at δ = 5.59 (q, *J* = 1.8 Hz, 1H) ppm. **HRMS** (Sicrit plasma/LTQ-Orbitrap) *m/z* = calcd. for $[C_{36}H_{39}O_2]^+$, $[M+H]^+$: 503.2945, found: 503.2945; **R$_f$** (pentane/EtOAc, 50:1) = 0.24.

Pentasubstituted cyclopentadiene isomers (R_a)-**Cp^V12** and Spirodiene (R_a)-**S12**

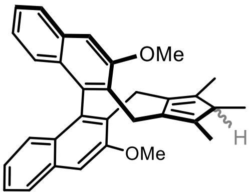

Cp^V12 (isomers)

+

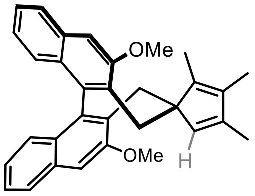

S12 (Cp/spiro = 92:8)

According to *General Procedure 2*, starting from chiral dibromide (R_a)-**L2** (0.89 mmol) and 1,2,3-Cp **T3** (1.1 equiv.) at 50 °C for 3 hours, a complex mixture of Cp^{V}H isomers **Cp^{V}12** and spirodiene **S12** (313 mg, 0.70 mmol, 79% yield, 92:8 ratio) was obtained as a light-yellow foam. Purification was performed by flash column chromatography on silica gel (dry loading, 15 cm column height, gradient: pentane/EtOAc = 100:0 → 30:1). Notably, **Cp^{V}12** was previously reported.[23,25]

The characteristic ^{1}H NMR signal (400 MHz, $CDCl_3$) of spirodiene **S12** is located at δ = 5.60 (q, *J* = 1.8 Hz, 1H) ppm. **HRMS** (ESI/QTOF) *m/z* = calcd. for $[C_{32}H_{31}O_2]^+$, $[M+H]^+$: 447.2319, found: 447.2311; **R$_f$** (pentane/EtOAc, 30:1) = 0.45.

Pentasubstituted cyclopentadiene isomers (R_a)-**Cp^V13** and Spirodiene (R_a)-**S13**

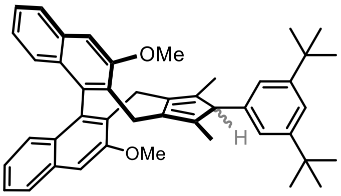

Cp^V13 (isomers)

+

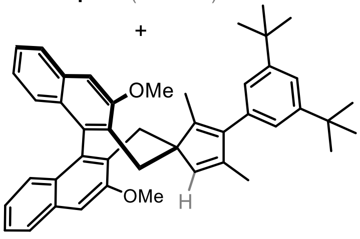

S13 (Cp/spiro = 76:24)

According to *General Procedure 2*, starting from chiral dibromide (R_a)-**L2** (601 µmol) and 1,2,3-Cp **T11** (1.1 equiv.) at 50 °C for 3 hours, a complex mixture of Cp^{V}H isomers **Cp^{V}13** and spirodiene **S13** (148 mg, 239 µmol, 40% yield, 76:24 ratio) was obtained as a light-yellow solid. Purification was performed by flash column chromatography on silica gel (dry loading, 15 cm column height, gradient: pentane/EtOAc = 100:0 → 50:1).

The characteristic ^{1}H NMR signal (400 MHz, $CDCl_3$) of spirodiene **S13** is located at δ = 5.73 (q, *J* = 1.7 Hz, 1H) ppm. **HRMS** (APCI/QTOF) *m/z* = calcd. for $[C_{45}H_{48}NaO_2]^+$, $[M+Na]^+$: 643.3547, found: 643.3541; **R$_f$** (pentane/EtOAc, 30:1) = 0.35.

Pentasubstituted cyclopentadiene isomers (R_a)-**Cp^V14** and Spirodiene (R_a)-**S14**

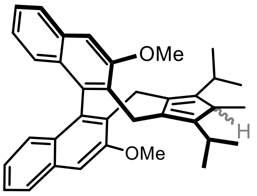

Cp^V14 (isomers)

+

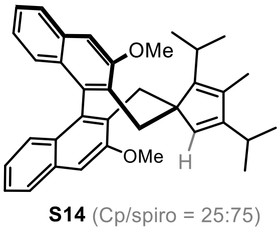

S14 (Cp/spiro = 25:75)

According to *General Procedure 2*, starting from chiral dibromide (R_a)-**L2** (238 µmol) and 1,2,3-Cp **T15** (1.1 equiv.) at 50 °C for 3 hours, a complex mixture of Cp^{V}H isomers **Cp^{V}14** and spirodiene **S14** (71 mg, 141 µmol, 59% yield, 25:75 ratio) was obtained as a light-yellow foam. Purification was performed by flash column chromatography on silica gel (dry loading, 15 cm column height, gradient: pentane/EtOAc = 100:0 → 30:1).

The characteristic ^{1}H NMR signal (400 MHz, $CDCl_3$) of spirodiene **S14** is located at δ = 5.38 (q, *J* = 1.6 Hz, 1H) ppm. **HRMS** (nanochip-ESI/LTQ-Orbitrap) *m/z* = calcd. for $[C_{36}H_{39}O_2]^+$, $[M+H]^+$: 503.2945, found: 503.2948; **R$_f$** (pentane/EtOAc, 30:1) = 0.43.

Pentasubstituted cyclopentadiene isomers (R_a)-**Cp^{V}15** and Spirodiene (R_a)-**S15**

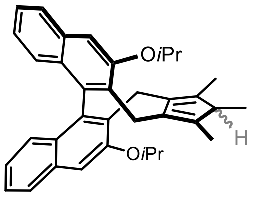

Cp^{V}15 (isomers)

+

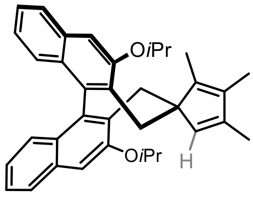

S15 (Cp/spiro = 96:4)

According to *General Procedure 2*, starting from chiral dibromide (R_a)-**L3** (0.77 mmol) and 1,2,3-Cp **T3** (1.1 equiv.) at 50 °C for 3 hours, a complex mixture of Cp^{V}H isomers **Cp^{V}15** and spirodiene **S15** (301 mg, 0.60 mmol, 78% yield, 96:4 ratio) was obtained as a light-yellow foam. Purification was performed by flash column chromatography on silica gel (dry loading, 15 cm column height, gradient: pentane/EtOAc = 100:0 → 30:1).

The characteristic ^{1}H NMR signal (400 MHz, CDCl$_3$) of spirodiene **S15** is located at δ = 5.62 (q, *J* = 1.8 Hz, 1H) ppm. **HRMS** (ESI/QTOF) *m/z* = calcd. for [$C_{36}H_{39}O_2$]$^+$, [M+H]$^+$: 503.2945, found: 503.2940; **R$_f$** (pentane/EtOAc, 40:1) = 0.28.

Pentasubstituted cyclopentadiene isomers (S_a)-**Cp^{V}16** and Spirodiene (S_a)-**S16**

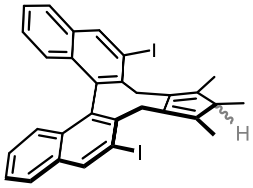

Cp^{V}16 (isomers)

+

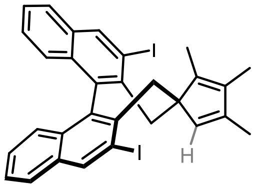

S16 (Cp/spiro = 80:20)

According to *General Procedure 2*, starting from chiral dibromide (S_a)-**L4** (160 µmol) and 1,2,3-Cp **T3** (1.1 equiv.) at 25 °C for 15 hours, a complex mixture of Cp^{V}H isomers **Cp^{V}16** and spirodiene **S16** (57 mg, 90 µmol, 56% yield, 80:20 ratio) was obtained as a light-yellow foam. Purification was performed by flash column chromatography on silica gel (dry loading, 15 cm column height, gradient: pentane/EtOAc = 100:0 → 50:1).

The characteristic ^{1}H NMR signal (400 MHz, CDCl$_3$) of spirodiene **S16** is located at δ = 5.67 (q, *J* = 1.8 Hz, 1H) ppm. **HRMS** (nanochip-ESI/LTQ-Orbitrap) *m/z* = calcd. for [$C_{30}H_{23}I_2O$]$^+$, [M+H]$^+$: 652.9833, found: 652.9838; **R$_f$** (pentane/EtOAc, 50:1) = 0.51.

Pentasubstituted cyclopentadiene isomers (S_a)-**Cp^{V}17** and Spirodiene (S_a)-**S17**

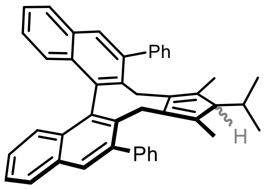

Cp^{V}17 (isomers)

+

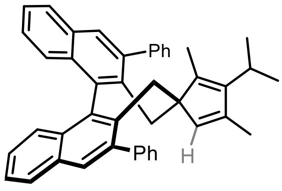

S17 (Cp/spiro = 41:59)

According to *General Procedure 2*, starting from chiral dibromide (S_a)-**L5** (303 µmol) and 1,2,3-Cp **T1** (1.1 equiv.) at 50 °C for 3 hours, a complex mixture of Cp^{V}H isomers **Cp^{V}17** and spirodiene **S17** (157 mg, 277 µmol, 92% yield, 41:59 ratio) was obtained as a light-yellow solid. Purification was performed by flash column chromatography on silica gel (dry loading, 15 cm column height, gradient: pentane/EtOAc = 100:0 → 30:1). Notably, a batch of the (R_a)-enantiomer was prepared as well.

The characteristic ^{1}H NMR signal (400 MHz, CDCl$_3$) of spirodiene **S17** is located at δ = 5.35 (q, *J* = 1.8 Hz, 1H) ppm. **HRMS** (ESI/QTOF) *m/z* = calcd. for [$C_{44}H_{39}$]$^+$, [M+H]$^+$: 567.3046, found: 567.3048; **R$_f$** (pentane/EtOAc, 50:1) = 0.25.

<u>Pentasubstituted cyclopentadiene isomers (S_a)-**Cp**</u>

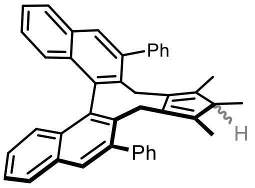

Cp^{V}18 (isomers)

+

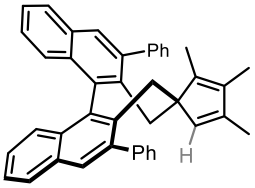

S18 (Cp/spiro = 66:34)

According to *General Procedure 2*, starting from chiral dibromide (S_a)-**L5** (281 µmol) and 1,2,3-Cp **T3** (1.1 equiv.) at 50 °C for 3 hours, a complex mixture of Cp^{V}H isomers **Cp^{V}18** and spirodiene **S18** (106 mg, 196 µmol, 70% yield, 66:34 ratio) was obtained as a white foam. Purification was performed by flash column chromatography on silica gel (dry loading, 15 cm column height, gradient: pentane/EtOAc = 100:0 → 30:1). Notably, (R_a)-**Cp^{V}18** was previously reported.[23]

The characteristic ^{1}H NMR signal (400 MHz, CDCl$_3$) of spirodiene **S18** is located at δ = 5.35 (q, *J* = 1.8 Hz, 1H) ppm. **HRMS** (ESI/QTOF) *m/z* = calcd. for $[C_{42}H_{35}]^+$, $[M+H]^+$: 539.2733, found: 539.2737; **R$_f$** (pentane/EtOAc, 50:1) = 0.33.

Pentasubstituted cyclopentadiene isomers (*R*)-**Cp^{V}19** and Spirodiene (*R*)-**S19**

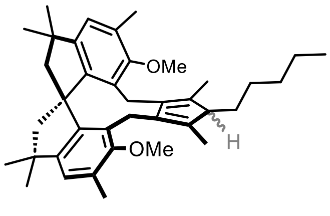

Cp^{V}19 (isomers)

+

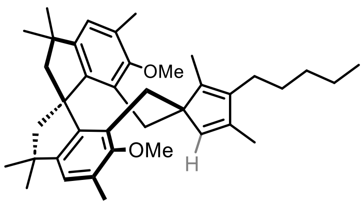

S19 (Cp/spiro = 98:2)

According to *General Procedure 2*, starting from chiral dibromide (*R*)-**L6** (571 µmol) and 1,2,3-Cp **T2** (1.1 equiv.) at 25 °C for 18 hours, a complex mixture of Cp^{V}H isomers **Cp^{V}19** and spirodiene **S19** (249 mg, 450 µmol, 79% yield, 98:2 ratio) was obtained as a light-yellow solid. Purification was performed by flash column chromatography on silica gel (dry loading, 15 cm column height, gradient: pentane/EtOAc = 100:0 → 30:1).

The characteristic ^{1}H NMR signal (400 MHz, CDCl$_3$) of spirodiene **S19** is located at δ = 5.17 (q, *J* = 1.6 Hz, 1H) ppm. **HRMS** (ESI/QTOF) *m/z* = calcd. for $[C_{39}H_{53}O_2]^+$, $[M+H]^+$: 553.4040, found: 553.4056; **R$_f$** (pentane/EtOAc, 30:1) = 0.41.

Pentasubstituted cyclopentadiene isomers (*R*)-**Cp^{V}20** and Spirodiene (*R*)-**S20**

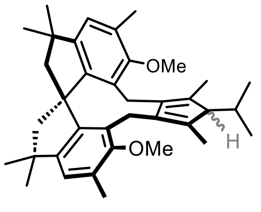

Cp^{V}20 (isomers)

+

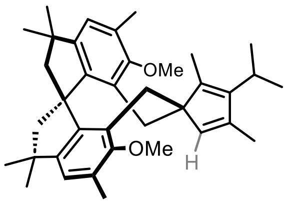

S20 (Cp/spiro = 98:2)

According to *General Procedure 2*, starting from chiral dibromide (*R*)-**L6** (192 µmol) and 1,2,3-Cp **T1** (1.1 equiv.) at 80 °C for 1.5 hours, a complex mixture of Cp^{V}H isomers **Cp^{V}20** and spirodiene **S20** (59 mg, 112 µmol, 58% yield, 98:2 ratio) was obtained as a light-yellow solid. Purification was performed by flash column chromatography on silica gel (dry loading, 15 cm column height, gradient: pentane/EtOAc = 100:0 → 30:1).

The characteristic ^{1}H NMR signal (400 MHz, CDCl$_3$) of spirodiene **S20** is located at δ = 5.18 (q, *J* = 1.6 Hz, 1H) ppm. **HRMS** (ESI/QTOF) *m/z* = calcd. for $[C_{37}H_{49}O_2]^+$, $[M+H]^+$: 525.3727, found: 525.3734; **R$_f$** (pentane/EtOAc, 30:1) = 0.44.

Pentasubstituted cyclopentadiene isomers (*R*)-**Cp^{V}21** and Spirodiene (*R*)-**S21**

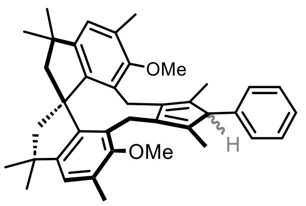

Cp^{V}21 (isomers)

+

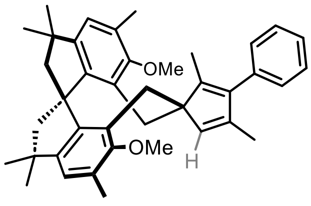

S21 (Cp/spiro = 97:3)

According to *General Procedure 2*, starting from chiral dibromide (*R*)-**L6** (179 µmol) and 1,2,3-Cp **T6** (1.1 equiv.) at 25 °C for 36 hours, a complex mixture of Cp^{V}H isomers **Cp^{V}21** and spirodiene **S21** (55 mg, 99 µmol, 55% yield, 97:3 ratio) was obtained as a light-yellow foam. Purification was performed by flash column chromatography on silica gel (dry loading, 15 cm column height, gradient: pentane/EtOAc = 100:0 → 30:1).

The characteristic ^{1}H NMR signal (400 MHz, $CDCl_3$) of spirodiene **S21** is located at δ = 5.33 (q, *J* = 1.6 Hz, 1H) ppm. **HRMS** (ESI/QTOF) *m/z* = calcd. for $[C_{40}H_{46}NaO_2]^+$, $[M+Na]^+$: 581.3390, found: 581.3391; **R$_f$** (pentane/EtOAc, 30:1) = 0.29.

Pentasubstituted cyclopentadiene isomers (*R*)-**Cp^{V}22** and Spirodiene (*R*)-**S22**

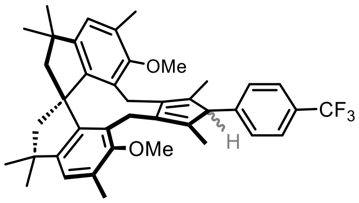

Cp^{V}22 (isomers)

+

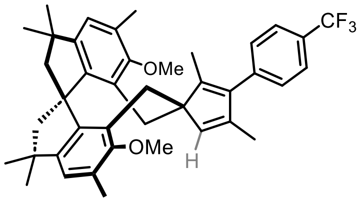

S22 (Cp/spiro = 92:8)

According to *General Procedure 2*, starting from chiral dibromide (*R*)-**L6** (171 µmol) and 1,2,3-Cp **T8** (1.1 equiv.) at 80 °C for 3 hours, a complex mixture of Cp^{V}H isomers **Cp^{V}22** and spirodiene **S22** (28 mg, 44 µmol, 26% yield, 92:8 ratio) was obtained as a light-yellow foam. Purification was performed by flash column chromatography on silica gel (dry loading, 15 cm column height, gradient: pentane/EtOAc = 100:0 → 40:1).

The characteristic ^{1}H NMR signal (400 MHz, $CDCl_3$) of spirodiene **S22** is located at δ = 5.36 (q, *J* = 1.6 Hz, 1H) ppm. **HRMS** (ESI/QTOF) *m/z* = calcd. for $[C_{41}H_{46}F_3O_2]^+$, $[M+H]^+$: 627.3444, found: 627.3460; **R$_f$** (pentane/EtOAc, 50:1) = 0.27.

Pentasubstituted cyclopentadiene isomers (*R*)-**Cp^{V}23** and Spirodiene (*R*)-**S23**

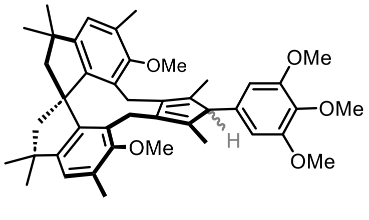

Cp^{V}23 (isomers)

+

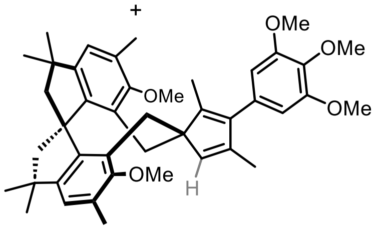

S23 (Cp/spiro = 94:6)

According to *General Procedure 2*, starting from chiral dibromide (*R*)-**L6** (154 µmol) and 1,2,3-Cp **T10** (1.1 equiv.) at 80 °C for 2 hours, a complex mixture of Cp^{V}H isomers **Cp^{V}23** and spirodiene **S23** (49 mg, 76 µmol, 49% yield, 94:6 ratio) was obtained as a light-yellow foam. Purification was performed by flash column chromatography on silica gel (dry loading, 15 cm column height, gradient: pentane/EtOAc = 20:1 → 4:1).

The characteristic ^{1}H NMR signal (400 MHz, $CDCl_3$) of spirodiene **S23** is located at δ = 5.36 (q, *J* = 1.7 Hz, 1H) ppm. **HRMS** (nanochip-ESI/LTQ-Orbitrap) *m/z* = calcd. for $[C_{43}H_{52}NaO_5]^+$, $[M+Na]^+$: 671.3707, found: 671.3727; **R$_f$** (pentane/EtOAc, 9:1) = 0.21.

Pentasubstituted cyclopentadiene isomers (*R*)-**Cp^{V}24** and Spirodiene (*R*)-**S24**

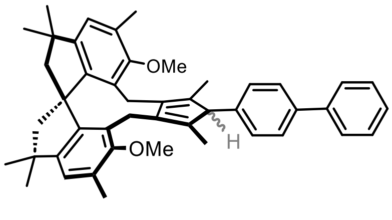

Cp^{V}24 (isomers)

+

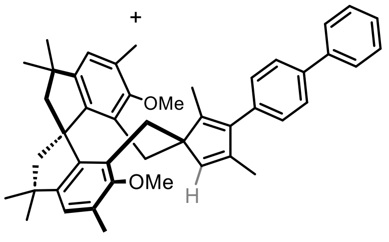

S24 (Cp/spiro = 98:2)

According to *General Procedure 2*, starting from chiral dibromide (*R*)-**L6** (190 µmol) and 1,2,3-Cp **T9** (1.1 equiv.) at 50 °C for 5 hours, a complex mixture of Cp^{V}H isomers **Cp^{V}24** and spirodiene **S24** (90 mg, 142 µmol, 75% yield, 98:2 ratio) was obtained as a light-yellow foam. Purification was performed by flash column chromatography on silica gel (dry loading, 15 cm column height, gradient: pentane/EtOAc = 100:0 → 30:1).

The characteristic ^{1}H NMR signal (400 MHz, $CDCl_3$) of spirodiene **S24** is located at δ = 5.35 (app s, 1H) ppm. **HRMS** (ESI/QTOF) *m/z* = calcd. for $[C_{46}H_{51}O_2]^+$, $[M+H]^+$: 635.3884, found: 635.3873; **R$_f$** (pentane/EtOAc, 30:1) = 0.32.

Pentasubstituted cyclopentadiene isomers (*R*)-**Cp^{V}25** and Spirodiene (*R*)-**S25**

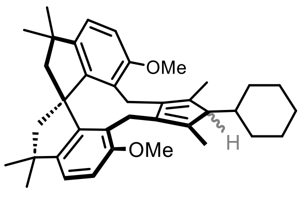

Cp^{V}25 (isomers)

+

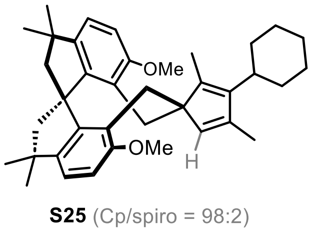

S25 (Cp/spiro = 98:2)

According to *General Procedure 2*, starting from chiral dibromide (*R*)-**L7** (351 µmol) and 1,2,3-Cp **T4** (1.1 equiv.) at 80 °C for 1.5 hours, a complex mixture of Cp^{V}H isomers **Cp^{V}25** and spirodiene **S25** (98 mg, 183 µmol, 52% yield, 98:2 ratio) was obtained as a light-yellow foam. Purification was performed by flash column chromatography on silica gel (wet loading with pentane, 15 cm column height, gradient: pentane/EtOAc = 100:0 → 30:1).

The characteristic ^{1}H NMR signal (400 MHz, $CDCl_3$) of spirodiene **S25** is located at δ = 5.65 (app s, 1H) ppm. **HRMS** (ESI/QTOF) *m/z* = calcd. for $[C_{38}H_{49}O_2]^+$, $[M+H]^+$: 537.3727, found: 537.3727; **R$_f$** (pentane/EtOAc, 40:1) = 0.27.

Pentasubstituted cyclopentadiene isomers (*R*)-**Cp^{V}26** and Spirodiene (*R*)-**S26**

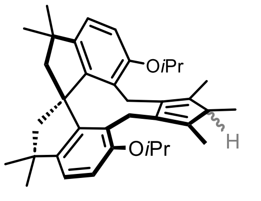

Cp^{V}26 (isomers)

\+

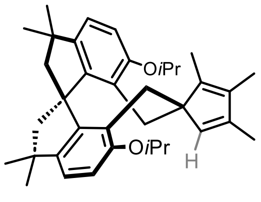

S26 (Cp/spiro = 92:8)

According to *General Procedure 2*, starting from chiral dibromide (*R*)-**L8** (310 µmol) and 1,2,3-Cp **T3** (1.1 equiv.) at 80 °C for 1.5 hours, a complex mixture of Cp^{V}H isomers **Cp^{V}26** and spirodiene **S26** (107 mg, 205 µmol, 66% yield, 92:8 ratio) was obtained as a light-yellow foam. Purification was performed by flash column chromatography on silica gel (wet loading with pentane, 15 cm column height, gradient: pentane/EtOAc = 100:0 → 30:1).

The characteristic ^{1}H NMR signal (400 MHz, $CDCl_3$) of spirodiene **S26** is located at δ = 5.62 (app s, 1H) ppm. **HRMS** (ESI/QTOF) *m/z* = calcd. for $[C_{37}H_{49}O_2]^+$, $[M+H]^+$: 525.3727, found: 525.3733; **R$_f$** (pentane/EtOAc, 50:1) = 0.22.

Pentasubstituted cyclopentadiene isomers (*R*)-**Cp^{V}27** and Spirodiene (*R*)-**S27**

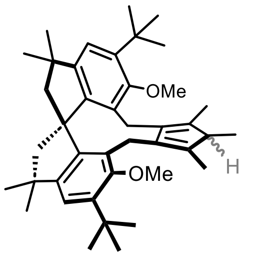

Cp^{V}27 (isomers)

\+

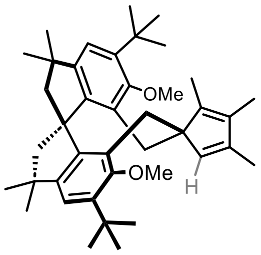

S27 (Cp/spiro = 98:2)

According to *General Procedure 2*, starting from chiral dibromide (*R*)-**L9** (255 µmol) and 1,2,3-Cp **T3** (1.1 equiv.) at 80 °C for 1.5 hours, a complex mixture of Cp^{V}H isomers **Cp^{V}27** and spirodiene **S27** (111 mg, 191 µmol, 75% yield, 98:2 ratio) was obtained as a light-yellow foam. Purification was performed by flash column chromatography on silica gel (wet loading with pentane, 15 cm column height, gradient: pentane/EtOAc = 100:0 → 30:1).

The characteristic ^{1}H NMR signal (400 MHz, $CDCl_3$) of spirodiene **S27** is located at δ = 5.51 (q, *J* = 1.6 Hz, 1H) ppm. **HRMS** (ESI/QTOF) *m/z* = calcd. for $[C_{41}H_{56}NaO_2]^+$, $[M+Na]^+$: 603.4173, found: 603.4168; **R$_f$** (pentane/EtOAc, 50:1) = 0.24.

Pentasubstituted cyclopentadiene isomers (*R*)-**Cp^{V}28** and Spirodiene (*R*)-**S28**

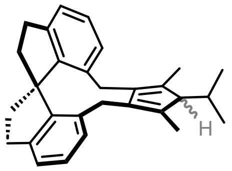

Cp^{V}28 (isomers)

+

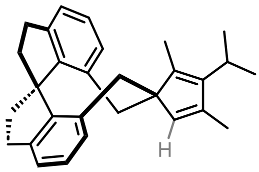

S28 (Cp/spiro = 88:12)

According to *General Procedure 2*, starting from chiral dibromide (*R*)-**L10** (451 µmol) and 1,2,3-Cp **T1** (1.1 equiv.) at 80 °C for 2 hours, a complex mixture of Cp^{V}H isomers **Cp^{V}28** and spirodiene **S28** (157 mg, 413 µmol, 92% yield, 88:12 ratio) was obtained as a light-yellow foam. Purification was performed by flash column chromatography on silica gel (wet loading with pentane, 15 cm column height, gradient: pentane/EtOAc = 100:0 → 100:1).

The characteristic ^{1}H NMR signal (400 MHz, $CDCl_3$) of spirodiene **S28** is located at δ = 5.39 (q, *J* = 1.7 Hz, 1H) ppm. **HRMS** (APCI/QTOF) *m/z* = calcd. for $[C_{29}H_{33}]^+$, $[M+H]^+$: 381.2577, found: 381.2569; **R$_f$** (pentane/EtOAc, 100:1) = 0.44.

Pentasubstituted cyclopentadiene isomers (*R*)-**Cp^{V}29** and Spirodiene (*R*)-**S29**

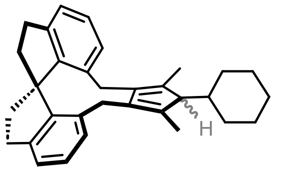

Cp^{V}29 (isomers)

+

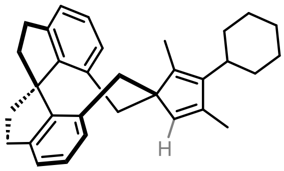

S29 (Cp/spiro = 96:4)

According to *General Procedure 2*, starting from chiral dibromide (*R*)-**L10** (325 µmol) and 1,2,3-Cp **T4** (1.1 equiv.) at 80 °C for 2 hours, a complex mixture of Cp^{V}H isomers **Cp^{V}29** and spirodiene **S29** (133 mg, 317 µmol, 98% yield, 96:4 ratio) was obtained as a white foam. Purification was performed by flash column chromatography on silica gel (wet loading with pentane, 10 cm column height, gradient: pentane/EtOAc = 100:0 → 30:1).

The characteristic ^{1}H NMR signal (400 MHz, $CDCl_3$) of spirodiene **S29** is located at δ = 5.38 (q, *J* = 1.7 Hz, 1H) ppm. **HRMS** (ESI/QTOF) *m/z* = calcd. for $[C_{32}H_{37}]^+$, $[M+H]^+$: 421.2890, found: 421.2888; **R$_f$** (pentane/EtOAc, 50:1) = 0.27.

Pentasubstituted cyclopentadiene isomers (*R*)-**Cp^{V}30** and Spirodiene (*R*)-**S30**

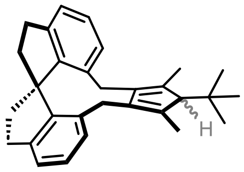

Cp^{V}30 (isomers)

+

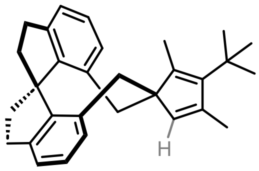

S30 (Cp/spiro = 88:12)

According to *General Procedure 2*, starting from chiral dibromide (*R*)-**L10** (527 µmol) and 1,2,3-Cp **T5** (1.1 equiv.) at 80 °C for 3 hours, a complex mixture of Cp^{V}H isomers **Cp^{V}30** and spirodiene **S30** (199 mg, 505 µmol, 96% yield, 88:12 ratio) was obtained as a white foam. Purification was performed by flash column chromatography on silica gel (wet loading with pentane, 15 cm column height, gradient: pentane/EtOAc = 100:0 → 50:1).

The characteristic ^{1}H NMR signal (400 MHz, $CDCl_3$) of spirodiene **S30** is located at δ = 5.40 (q, *J* = 1.6 Hz, 1H) ppm. **HRMS** (APCI/QTOF) *m/z* = calcd. for $[C_{30}H_{35}]^+$, $[M+H]^+$: 395.2733, found: 395.2721; **R$_f$** (pentane/EtOAc, 100:1) = 0.31.

Pentasubstituted cyclopentadiene isomers (*R*)-**Cp^{V}31** and Spirodiene (*R*)-**S31**

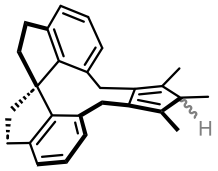

Cp^{V}31 (isomers)

+

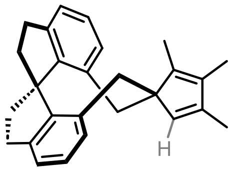

S31 (Cp/spiro = 99:1)

According to *General Procedure 2*, starting from chiral dibromide (*R*)-**L10** (863 µmol) and 1,2,3-Cp **T3** (1.1 equiv.) at 80 °C for 2 hours, a complex mixture of Cp^{V}H isomers **Cp^{V}31** and spirodiene **S31** (265 mg, 752 µmol, 87% yield, 99:1 ratio) was obtained as a white foam. Purification was performed by flash column chromatography on silica gel (wet loading with pentane, 15 cm column height, gradient: pentane/EtOAc = 100:0 → 50:1).

The characteristic ^{1}H NMR signal (400 MHz, $CDCl_3$) of spirodiene **S31** is located at δ = 5.42 (app s, 1H) ppm. **HRMS** (ESI/QTOF) *m/z* = calcd. for $[C_{27}H_{29}]^+$, $[M+H]^+$: 353.2264, found: 353.2258; **R$_f$** (pentane/EtOAc, 50:1) = 0.47.

Pentasubstituted cyclopentadiene isomers (*R*)-**Cp^{V}32** and Spirodiene (*R*)-**S32**

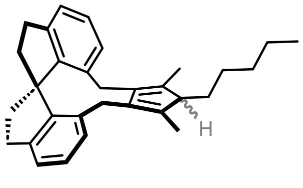

Cp^{V}32 (isomers)

+

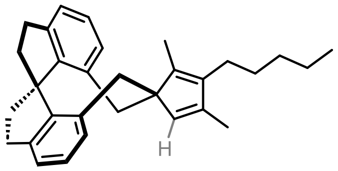

S32 (Cp/spiro = 97:3)

According to a gram-scale version of *General Procedure 2*, in a 250 mL round-bottom flask with deoiled potassium hydride powder (482 mg, 3.0 equiv.), starting from chiral dibromide (*R*)-**L10** (1.628 g, 4.00 mmol) and 1,2,3-Cp **T2** (724 mg, 1.1 equiv.) in 120 mL anhydrous THF at 50 °C for 5 hours, a complex mixture of Cp^{V}H isomers **Cp^{V}32** and spirodiene **S32** (1.369 g, 3.35 mmol, 84% yield, 97:3 ratio) was obtained as a light-yellow foam. Notably, this gram-scale dialkylation proceeded with similar efficiency as for smaller scales and did not exhibit practical constraints. Purification was performed by flash column chromatography on silica gel (wet loading with pentane, 15 cm column height, gradient: pentane/EtOAc = 100:0 → 50:1).

The characteristic ^{1}H NMR signal (400 MHz, $CDCl_3$) of spirodiene **S32** is located at δ = 5.42 (q, *J* = 1.8 Hz, 1H) ppm. **HRMS** (ESI/QTOF) *m/z* = calcd. for $[C_{31}H_{37}]^+$, $[M+H]^+$: 409.2890, found: 409.2888; **R$_f$** (pentane) = 0.24.

Pentasubstituted cyclopentadiene isomers (*R*)-**Cp^{V}33** and Spirodiene (*R*)-**S33**

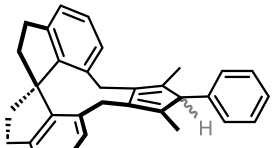

Cp^{V}33 (isomers)

+

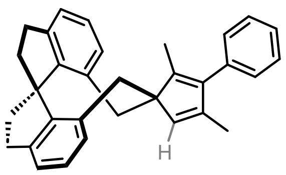

S33 (Cp/spiro = 92:8)

According to *General Procedure 2*, starting from chiral dibromide (*R*)-**L10** (352 µmol) and 1,2,3-Cp **T6** (1.1 equiv.) at 80 °C for 2 hours, a complex mixture of Cp^{V}H isomers **Cp^{V}33** and spirodiene **S33** (111 mg, 269 µmol, 76% yield, 92:8 ratio) was obtained as a white foam. Purification was performed by flash column chromatography on silica gel (wet loading with pentane, 10 cm column height, gradient: pentane/EtOAc = 100:0 → 30:1).

The characteristic ^{1}H NMR signal (400 MHz, $CDCl_3$) of spirodiene **S33** is located at δ = 5.53 (q, *J* = 1.7 Hz, 1H) ppm. **HRMS** (ESI/QTOF) *m/z* = calcd. for $[C_{32}H_{31}]^+$, $[M+H]^+$: 415.2420, found: 415.2417; **R$_f$** (pentane/EtOAc, 50:1) = 0.22.

Pentasubstituted cyclopentadiene isomers (*R*)-**Cp^{V}34** and Spirodiene (*R*)-**S34**

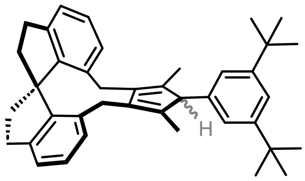

Cp^{V}34 (isomers)

+

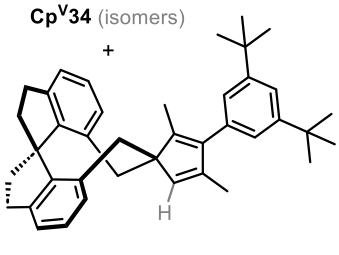

S34 (Cp/spiro = 98:2)

According to *General Procedure 2*, starting from chiral dibromide (*R*)-**L10** (328 µmol) and 1,2,3-Cp **T11** (1.1 equiv.) at 80 °C for 2 hours, a complex mixture of Cp^{V}H isomers **Cp^{V}34** and spirodiene **S34** (172 mg, 327 µmol, 99% yield, 98:2 ratio) was obtained as a light-yellow foam. Purification was performed by flash column chromatography on silica gel (wet loading with pentane, 10 cm column height, gradient: pentane/EtOAc = 100:0 → 30:1).

The characteristic ^{1}H NMR signal (400 MHz, $CDCl_3$) of spirodiene **S34** is located at δ = 5.54 (q, *J* = 1.7 Hz, 1H) ppm. **HRMS** (APCI/QTOF) *m/z* = calcd. for $[C_{40}H_{47}]^+$, $[M+H]^+$: 527.3672, found: 527.3666; **R$_f$** (pentane/EtOAc, 50:1) = 0.20.

Pentasubstituted cyclopentadiene isomers (*R*)-**Cp^{V}35** and Spirodiene (*R*)-**S35**

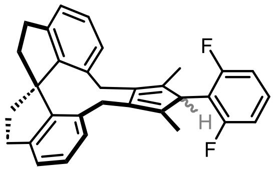

Cp^{V}35 (isomers)

+

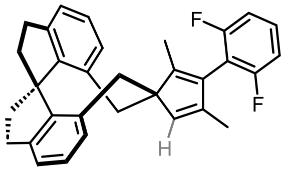

S35 (Cp/spiro = 88:12)

According to *General Procedure 2*, starting from chiral dibromide (*R*)-**L10** (345 µmol) and 1,2,3-Cp **T14** (1.1 equiv.) at 80 °C for 2 hours, a complex mixture of Cp^{V}H isomers **Cp^{V}35** and spirodiene **S35** (142 mg, 315 µmol, 91% yield, 88:12 ratio) was obtained as a white foam. Purification was performed by flash column chromatography on silica gel (wet loading with pentane, 10 cm column height, gradient: pentane/EtOAc = 100:0 → 50:1).

The characteristic ^{1}H NMR signal (400 MHz, $CDCl_3$) of spirodiene **S35** is located at δ = 5.55 (q, *J* = 1.7 Hz, 1H) ppm. **HRMS** (APCI/QTOF) *m/z* = calcd. for $[C_{32}H_{29}F_2]^+$, $[M+H]^+$: 451.2232, found: 451.2227; **R$_f$** (pentane/EtOAc, 50:1) = 0.32.

Pentasubstituted cyclopentadiene isomers (*S*$_a$)-**Cp^{V}36** and Spirodiene (*S*$_a$)-**S36**

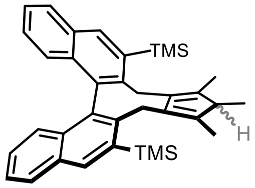

Cp^{V}36 (isomers)

+

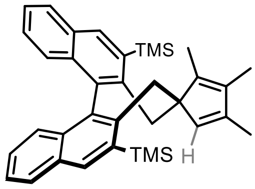

S36 (Cp/spiro = 63:37)

According to *General Procedure 2*, starting from chiral dibromide (*S*$_a$)-**L11** (366 µmol) and 1,2,3-Cp **T3** (1.1 equiv.) at 25 °C for 6 hours, a complex mixture of Cp^{V}H isomers **Cp^{V}36** and spirodiene **S36** (171 mg, 323 µmol, 88% yield, 63:37 ratio) was obtained as a white foam. Purification was performed by flash column chromatography on silica gel (wet loading with pentane, 15 cm column height, gradient: pentane/EtOAc = 100:0 → 50:1).

The characteristic ^{1}H NMR signal (400 MHz, $CDCl_3$) of spirodiene **S36** is located at δ = 5.57 (q, *J* = 1.8 Hz, 1H) ppm. **HRMS** (ESI/QTOF) *m/z* = calcd. for $[C_{36}H_{43}Si_2]^+$, $[M+H]^+$: 531.2898, found: 531.2896; **R$_f$** (pentane/EtOAc, 100:1) = 0.36.

Pentasubstituted cyclopentadiene isomers (*R*)-**Cp^{V}37** and Spirodiene (*R*)-**S37**

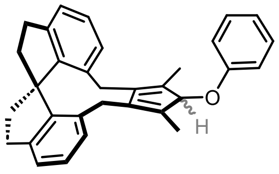

Cp^{V}37 (isomers)

+

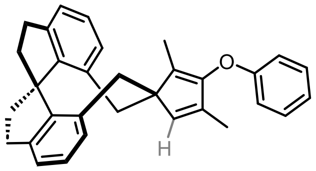

S37 (Cp/spiro = 54:46)

According to *General Procedure 2*, starting from chiral dibromide (*R*)-**L10** (423 µmol) and 1,2,3-Cp **T16** (1.0 equiv.) at 80 °C for 3 hours, a complex mixture of Cp^{V}H isomers **Cp^{V}37** and spirodiene **S37** (157 mg, 364 µmol, 86% yield, 54:46 ratio) was obtained as a white foam. Purification was performed by flash column chromatography on silica gel (wet loading with pentane/DCM = 20:1, 12 cm column height, gradient: pentane/EtOAc = 100:0 → 50:1).

The characteristic ^{1}H NMR signal (400 MHz, $CDCl_3$) of spirodiene **S37** is located at δ = 5.58 (q, *J* = 1.7 Hz, 1H) ppm. **HRMS** (APCI/QTOF) *m/z* = calcd. for $[C_{32}H_{31}O]^+$, $[M+H]^+$: 431.2369, found: 431.2389; **R$_f$** (pentane/EtOAc, 30:1) = 0.38.

Pentasubstituted cyclopentadiene isomers (*R*$_a$)-**Cp^{V}38** and Spirodiene (*R*$_a$)-**S38**

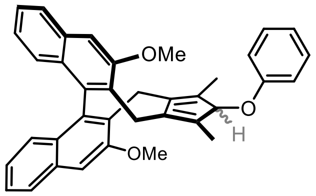

Cp^{V}38 (isomers)

+

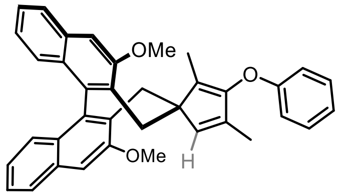

S38 (Cp/spiro = 40:60)

According to *General Procedure 2*, starting from chiral dibromide (*R*$_a$)-**L2** (801 µmol) and 1,2,3-Cp **T16** (1.0 equiv.) at 25 °C for 5 hours, a complex mixture of Cp^{V}H isomers **Cp^{V}38** and spirodiene **S38** (323 mg, 615 µmol, 77% yield, 40:60 ratio) was obtained as a light-yellow foam. Purification was performed by flash column chromatography on silica gel (dry loading, 15 cm column height, gradient: pentane/EtOAc = 100:0 → 20:1).

The characteristic ^{1}H NMR signal (400 MHz, $CDCl_3$) of spirodiene **S38** is located at δ = 5.75 (q, *J* = 1.8 Hz, 1H) ppm. **HRMS** (nanochip-ESI/LTQ-Orbitrap) *m/z* = calcd. for $[C_{37}H_{33}O_3]^+$, $[M+H]^+$: 525.2424, found: 525.2423; **R$_f$** (pentane/EtOAc, 30:1) = 0.32.

Pentasubstituted cyclopentadiene isomers (*R*)-**Cp^{V}39** and Spirodiene (*R*)-**S39**

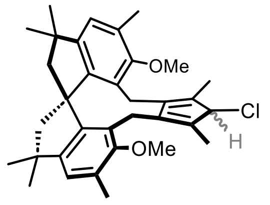

Cp^{V}39 (isomers)

+

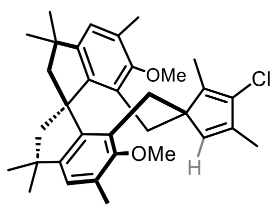

S39 (Cp/spiro = 99:1)

According to *General Procedure 2*, starting from chiral dibromide (*R*)-**L6** (311 µmol) and 1,2,3-Cp **T17** (1.1 equiv.) at 50 °C for 3 hours, a complex mixture of Cp^{V}H isomers **Cp^{V}39** and spirodiene **S39** (121 mg, 234 µmol, 75% yield, 99:1 ratio) was obtained as a light-yellow foam. Purification was performed by flash column chromatography on silica gel (dry loading, 15 cm column height, gradient: pentane/EtOAc = 100:0 → 30:1).

The characteristic ^{1}H NMR signal (400 MHz, $CDCl_3$) of spirodiene **S39** is located at δ = 5.34 (app s, 1H) ppm. **HRMS** (ESI/QTOF) *m/z* = calcd. for $[C_{34}H_{42}ClO_2]^+$, $[M+H]^+$: 517.2868, found: 517.2875; **R$_f$** (pentane/EtOAc, 30:1) = 0.36.

5. Synthesis of Chiral Cp Metal Complexes

5.1 Di- and Trisubstituted Cp^x Metal Complexes

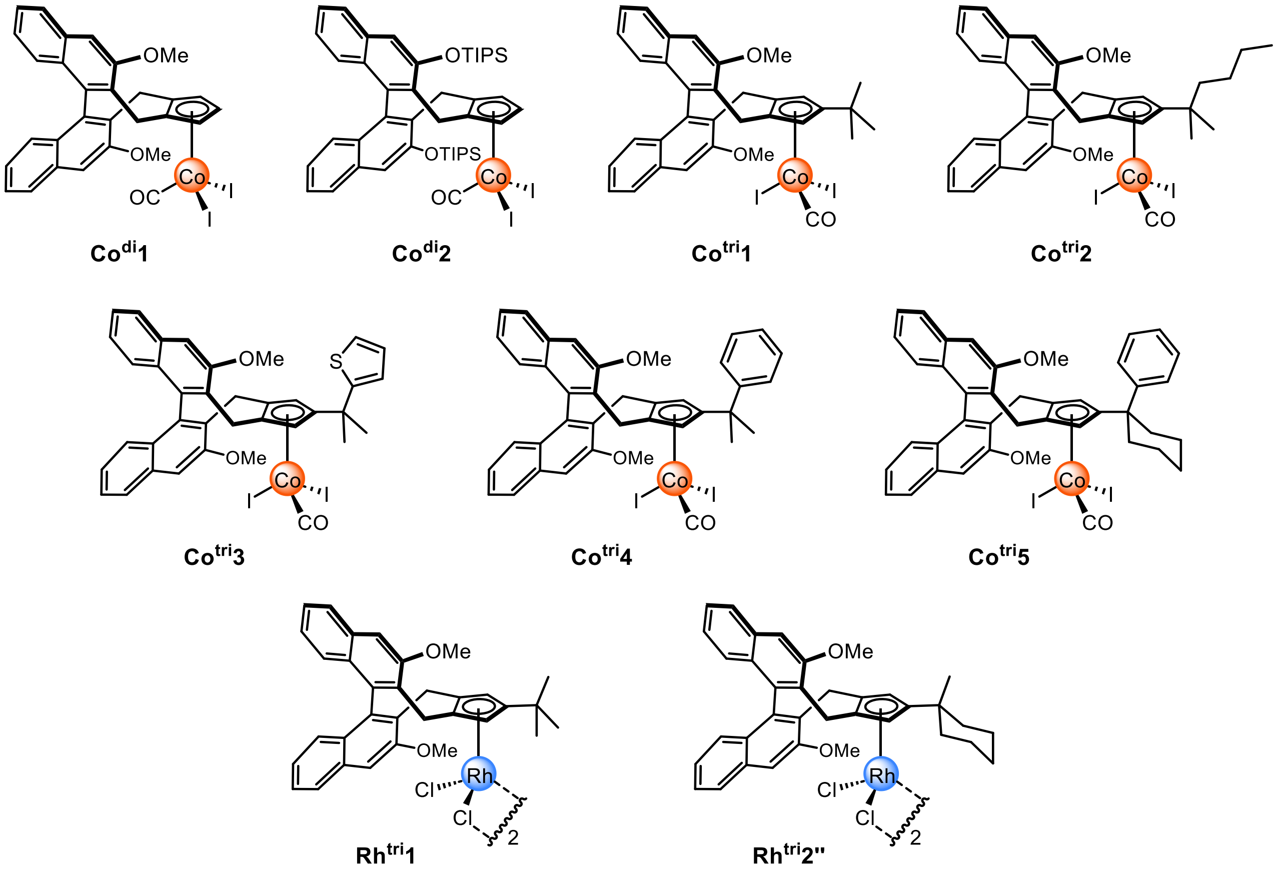

Figure S5. Overview of the di- and trisubstituted Cp^{x}Co(III) and Cp^{x}Rh(III) complexes used in this work.

Di- and trisubstituted chiral cyclopentadienyl cobalt(III) and rhodium(III) complexes (R_a)-**Co^{di}1**, (R_a)-**Co^{tri}1**, (R_a)-**Rh^{tri}1**, and (R_a)-**Rh^{tri}2''** were prepared from the corresponding chiral cyclopentadienes according to the reported procedures with matching characterization data.[26–28] Chiral cobalt complexes (R_a)-**Co^{di}2** and (R_a)-**Co^{tri}2-5** have not yet been reported, and the characterization data is provided *in infra*.

For (R_a)-**Co^{di}1**:
^{1}H NMR (400 MHz, $CDCl_3$) δ = 7.87 (d, *J* = 8.2 Hz, 1H), 7.80 (d, *J* = 8.2 Hz, 1H), 7.47 – 7.40 (m, 2H), 7.40 (s, 1H), 7.23 (s, 1H), 7.15 – 7.06 (m, 3H), 7.00 (d, *J* = 8.5 Hz, 1H), 5.82 (t, *J* = 2.2 Hz, 1H), 5.75 (t, *J* = 2.6 Hz, 1H), 5.46 (t, *J* = 2.2 Hz, 1H), 4.12 (s, 3H), 4.07 (d, *J* = 16.1 Hz, 1H), 3.97 (s, 3H), 3.90 (d, *J* = 14.0 Hz, 1H), 3.59 (d, *J* = 14.0 Hz, 1H), 2.77 (d, *J* = 16.1 Hz, 1H) ppm.

For (R_a)-**Co^{tri}1**:
^{1}H NMR (400 MHz, $CDCl_3$) δ = 7.88 (d, *J* = 8.1 Hz, 1H), 7.80 (d, *J* = 8.4 Hz, 1H), 7.47 – 7.40 (m, 2H), 7.39 (s, 1H), 7.23 (s, 1H), 7.14 – 7.08 (m, 3H), 7.00 (d, *J* = 8.9 Hz, 1H), 5.56 (d, *J* = 2.1 Hz, 1H), 5.44 (d, *J* = 2.1 Hz, 1H), 4.18 (d, *J* = 16.3 Hz, 1H), 4.10 (s, 3H), 3.96 (s, 3H), 3.72 (d, *J* = 13.7 Hz, 1H), 3.49 (d, *J* = 13.9 Hz, 1H), 2.80 (d, *J* = 16.2 Hz, 1H), 1.54 (s, 9H) ppm.

For (R_a)-**Rh^{tri}1**:

^{1}H NMR (400 MHz, d_6-DMSO) δ = 7.93 – 7.88 (m, 2H), 7.52 (s, 1H), 7.46 – 7.38 (m, 3H), 7.14 – 7.04 (m, 2H), 6.82 – 6.76 (m, 2H), 6.01 (d, *J* = 1.6 Hz, 1H), 5.99 (d, *J* = 1.6 Hz, 1H), 4.08 (d, *J* = 16.6 Hz, 1H), 4.01 (s, 3H), 3.93 (s, 3H), 3.57 (d, *J* = 13.0 Hz, 1H), 3.11 (d, *J* = 12.9 Hz, 1H), 2.28 (d, *J* = 16.4 Hz, 1H), 1.22 (s, 9H) ppm.

For (R_a)-**Rh^{tri}2''**:

^{1}H NMR (400 MHz, d_6-DMSO) δ = 7.91 (dd, *J* = 8.2, 4.3 Hz, 2H), 7.52 (s, 1H), 7.46 – 7.38 (m, 3H), 7.14 – 7.05 (m, 2H), 6.83 – 6.78 (m, 2H), 6.00 (s, 2H), 4.07 (d, *J* = 16.4 Hz, 1H), 4.00 (s, 3H), 3.93 (s, 3H), 3.58 (d, *J* = 13.0 Hz, 1H), 3.12 (d, *J* = 12.9 Hz, 1H), 2.26 (d, *J* = 16.4 Hz, 1H), 1.72 – 1.62 (m, 1H), 1.60 – 1.32 (m, 8H), 1.25 (s, 3H), 1.23 – 1.12 (m, 1H) ppm.

Cobalt complex (R_a)-**Co^{di}2**

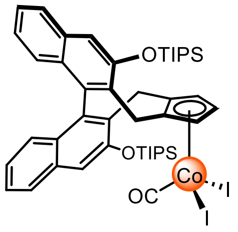

According to *General Procedure 3* (see **Section 5.2**), starting from **Cp^{di}2** (94 µmol), chiral Co(III)-complex **Co^{di}2** (42 mg, 41 µmol, 44% yield) was obtained as a black solid. The cobalt complex exists as a single 18-electron monomeric species.

^{1}H NMR (600 MHz, $CDCl_3$) δ = 7.82 (d, *J* = 8.2 Hz, 1H), 7.71 (d, *J* = 8.2 Hz, 1H), 7.54 (s, 1H), 7.46 – 7.43 (m, 1H), 7.40 – 7.36 (m, 1H), 7.28 (s, 1H), 7.15 – 7.11 (m, 1H), 7.10 – 7.07 (m, 1H), 6.95 (d, *J* = 8.5 Hz, 1H), 6.85 (d, *J* = 8.5 Hz, 1H), 6.05 (t, *J* = 2.3 Hz, 1H), 5.86 (t, *J* = 2.7 Hz, 1H), 5.12 (t, *J* = 2.2 Hz, 1H), 4.23 (d, *J* = 16.0 Hz, 1H), 3.98 (d, *J* = 14.2 Hz, 1H), 3.41 (d, *J* = 14.2 Hz, 1H), 2.82 (d, *J* = 15.9 Hz, 1H), 1.56 – 1.42 (m, 6H), 1.26 (d, *J* = 7.5 Hz, 9H), 1.20 (d, *J* = 7.5 Hz, 9H), 1.19 – 1.16 (m, 18H) ppm; **^{13}C{^{1}H} NMR** (151 MHz, $CDCl_3$) δ = 195.6, 152.3, 151.2, 138.6, 137.2, 134.1, 133.9, 128.7, 127.8, 127.6, 127.1, 126.9, 126.8, 126.7, 126.54, 126.48, 126.4, 124.74, 124.66, 115.5, 113.9, 108.0, 99.2, 96.3, 90.3, 85.5, 27.1, 25.4, 18.54, 18.47, 18.4, 13.3, 13.2 ppm; **IR** (ATR) ṽ = 2944 (m), 2891 (w), 2866 (m), 2069 (s), 1593 (w), 1460 (w), 1436 (m), 1332 (w), 1242 (w), 1224 (w), 1173 (w), 1115 (w), 925 (m), 909 (w), 882 (w), 841 (w), 812 (m), 747 (w), 731 (w), 688 (w), 649 (w), 469 (w) cm^{-1}; **HRMS** (ESI/QTOF) *m*/*z* = calcd. for $[C_{45}H_{59}CoIO_2Si_2]^+$, $[M\text{-}CO\text{-}I^-]^+$: 873.2425, found: 873.2433; $[\alpha]_D^{23}$ = -194.4 (*c* = 0.3, $CHCl_3$).

Cobalt complex (R_a)-**Co^{tri}2**

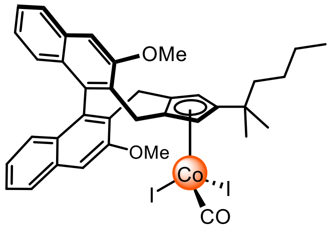

According to *General Procedure 3* (see **Section 5.2**), starting from **Cp^{tri}2** (123 µmol), chiral Co(III)-complex **Co^{tri}2** (67 mg, 80 µmol, 65% yield over 2 steps) was obtained as a black solid. The cobalt complex exists as a single 18-electron monomeric species.

^{1}H NMR (400 MHz, $CDCl_3$) δ = 7.88 (d, *J* = 8.2 Hz, 1H), 7.81 (d, *J* = 8.2 Hz, 1H), 7.48 – 7.40 (m, 2H), 7.39 (s, 1H), 7.23 (s, 1H), 7.17 – 7.08 (m, 3H), 7.02 (d, *J* = 8.4 Hz, 1H), 5.53 (d, *J* = 2.1 Hz, 1H), 5.37 (d, *J* = 2.1 Hz, 1H), 4.20 (d, *J* = 16.3 Hz, 1H), 4.10 (s, 3H), 3.95 (s, 3H), 3.71 (d, *J* = 13.7 Hz, 1H), 3.54 (d, *J* = 13.7 Hz, 1H), 2.83 (d, *J* = 16.3 Hz, 1H), 1.65 (s, 3H), 1.61 (s, 3H), 1.61 – 1.53 (m, 1H), 1.51 – 1.42 (m, 1H), 1.27 – 1.17 (m, 2H), 1.15 – 1.04 (m, 1H), 1.00 – 0.88 (m, 1H), 0.83 (t, *J* = 7.3 Hz, 3H) ppm; **^{13}C{^{1}H} NMR** (101 MHz, $CDCl_3$) δ = 198.1, 156.2, 154.7, 138.1, 137.1, 134.4, 134.0, 127.4, 127.2, 127.1, 127.0, 126.83, 126.79, 126.76, 124.8, 124.6, 124.2, 113.3, 106.3, 106.1, 99.9, 98.8, 94.6, 91.4, 55.6, 55.4, 46.3, 35.3, 29.0, 27.8, 26.6, 25.9, 24.3, 23.1, 14.2 ppm; **IR** (ATR) ṽ = 2957 (w), 2930 (w), 2054 (s), 1596

(w), 1450 (m), 1423 (w), 1329 (w), 1236 (w), 1226 (w), 1196 (w), 1167 (w), 1114 (w), 1020 (w), 907 (w), 833 (w), 730 (m), 466 (w) cm^{-1}; **HRMS** (ESI-APCI/TOF) *m/z* = calcd. for $[C_{36}H_{37}CoIO_2]^+$, $[M\text{-}CO\text{-}I^-]^+$: 687.1170, found: 687.1176; $[\alpha]_D^{23}$ = -35.2 (*c* = 0.6, CH_2Cl_2).

Cobalt complex (R_a)-**Co^{tri}3**

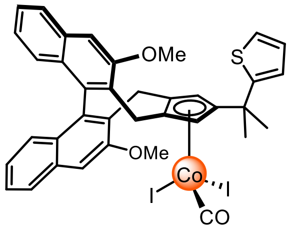

According to *General Procedure 3* (see **Section 5.2**), starting from **Cp^{tri}3** (352 µmol), chiral Co(III)-complex **Co^{tri}3** (110 mg, 127 µmol, 36% yield over 2 steps) was obtained as a black solid. The cobalt complex exists as a single 18-electron monomeric species. Notably, the thiophene moiety does not coordinate to cobalt by replacing the CO ligand (as confirmed by both ^{13}C NMR and IR).

^{1}H NMR (400 MHz, CD_2Cl_2) δ = 7.90 (d, *J* = 8.3 Hz, 1H), 7.81 (d, *J* = 8.1 Hz, 1H), 7.49 – 7.38 (m, 3H), 7.25 (s, 1H), 7.19 – 7.16 (m, 1H), 7.14 – 7.08 (m, 2H), 7.06 – 7.01 (m, 2H), 6.93 – 6.86 (m, 1H), 6.83 (d, *J* = 3.6 Hz, 1H), 5.61 (d, *J* = 2.1 Hz, 1H), 5.53 (d, *J* = 2.1 Hz, 1H), 4.14 (d, *J* = 16.3 Hz, 1H), 4.08 (s, 3H), 3.89 (s, 3H), 3.69 (d, *J* = 13.6 Hz, 1H), 3.47 (d, *J* = 13.7 Hz, 1H), 2.74 (d, *J* = 16.4 Hz, 1H), 2.17 (s, 3H), 2.09 (s, 3H) ppm; **^{13}C{^{1}H} NMR** (151 MHz, CD_2Cl_2) δ = 198.6, 156.6, 155.0, 154.3, 138.5, 137.0, 134.7, 134.4, 127.5, 127.42, 127.36, 127.3, 127.09, 127.07, 127.01, 126.98, 125.0, 124.59, 124.56, 124.3, 123.5, 111.8, 106.5, 106.4, 100.7, 99.1, 95.6, 92.5, 55.9, 55.6, 38.3, 31.7, 30.8, 26.0, 24.5 ppm; **IR** (ATR) ṽ = 2967 (w), 2059 (s), 1619 (w), 1597 (w), 1478 (w), 1450 (m), 1423 (w), 1411 (w), 1387 (w), 1329 (w), 1294 (w), 1239 (w), 1226 (w), 1197 (w), 1167 (w), 1149 (w), 1114 (w), 1079 (w), 1020 (w), 907 (w), 833 (w), 747 (w), 732 (w), 700 (w), 466 (w) cm^{-1}; **HRMS** (ESI-APCI/TOF) *m/z* = calcd. for $[C_{36}H_{31}CoIO_2S]^+$, $[M\text{-}CO\text{-}I^-]^+$: 713.0421, found: 713.0422; $[\alpha]_D^{23}$ = -63.8 (*c* = 0.4, CH_2Cl_2).

Cobalt complex (R_a)-**Co^{tri}4**

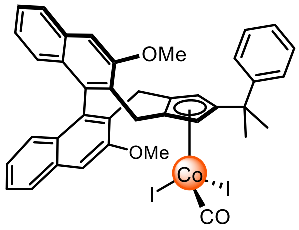

According to *General Procedure 3* (see **Section 5.2**), starting from **Cp^{tri}4** (189 µmol), chiral Co(III)-complex **Co^{tri}4** (65 mg, 75 µmol, 40% yield over 2 steps) was obtained as a black solid. The cobalt complex exists as a single 18-electron monomeric species.

^{1}H NMR (400 MHz, CD_2Cl_2) δ = 7.90 (d, *J* = 8.3 Hz, 1H), 7.82 (d, *J* = 8.2 Hz, 1H), 7.48 – 7.38 (m, 3H), 7.30 – 7.16 (m, 6H), 7.14 – 7.08 (m, 2H), 7.06 – 7.00 (m, 2H), 5.55 (d, *J* = 2.2 Hz, 1H), 5.44 (d, *J* = 2.1 Hz, 1H), 4.13 (d, *J* = 16.4 Hz, 1H), 4.08 (s, 3H), 3.85 (s, 3H), 3.67 (d, *J* = 13.6 Hz, 1H), 3.49 (d, *J* = 13.6 Hz, 1H), 2.74 (d, *J* = 16.5 Hz, 1H), 2.11 (s, 3H), 2.03 (s, 3H) ppm; **^{13}C{^{1}H} NMR** (101 MHz, CD_2Cl_2) δ = 198.8, 156.7, 155.1, 148.6, 138.5, 137.0, 134.7, 134.4, 128.9, 127.6, 127.53, 127.45, 127.38, 127.36, 127.1, 127.00, 126.96, 126.1, 125.1, 124.6, 124.3, 113.3, 106.5, 106.4, 100.5, 99.5, 95.8, 92.4, 55.9, 55.6, 39.5, 30.5, 29.7, 26.1, 24.5 ppm; **IR** (ATR) ṽ = 2963 (w), 2933 (w), 2055 (s), 1618 (w), 1596 (w), 1449 (m), 1423 (w), 1411 (w), 1328 (w), 1295 (w), 1238 (w), 1226 (w), 1197 (w), 1167 (w), 1114 (w), 1020 (w), 952 (w), 833 (w), 748 (w), 710 (w), 701 (w), 466 (w) cm^{-1}; **HRMS** (ESI/QTOF) *m/z* = calcd. for $[C_{38}H_{33}CoIO_2]^+$,$[M\text{-}CO\text{-}I^-]^+$: 707.0852, found: 707.0833; $[\alpha]_D^{23}$ = -30.4 (*c* = 0.7, CH_2Cl_2).

Cobalt complex (R_a)-**Co**^{tri}**5**

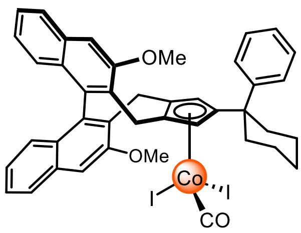

According to *General Procedure 3* (see **Section 5.2**), starting from **Cp**^{tri}**5** (111 µmol), chiral Co(III)-complex **Co**^{tri}**5** (47 mg, 51 µmol, 46% yield over 2 steps) was obtained as a black solid. The cobalt complex exists as a single 18-electron monomeric species.

^{1}**H NMR** (400 MHz, CD_2Cl_2) δ = 7.88 (d, *J* = 8.2 Hz, 1H), 7.79 (d, *J* = 8.2 Hz, 1H), 7.49 – 7.30 (m, 7H), 7.27 – 7.21 (m, 1H), 7.19 (s, 1H), 7.13 – 7.06 (m, 2H), 7.04 – 6.96 (m, 2H), 5.51 (d, *J* = 2.1 Hz, 1H), 5.23 (d, *J* = 2.1 Hz, 1H), 4.13 (d, *J* = 16.3 Hz, 1H), 4.06 (s, 3H), 3.79 (s, 3H), 3.56 (d, *J* = 13.6 Hz, 1H), 3.46 (d, *J* = 13.6 Hz, 1H), 2.97 (d, *J* = 14.0 Hz, 1H), 2.77 (d, *J* = 13.9 Hz, 1H), 2.72 – 2.60 (m, 2H), 2.55 – 2.45 (m, 1H), 1.77 – 1.60 (m, 3H), 1.50 – 1.30 (m, 3H) ppm; ^{13}**C{**^{1}**H} NMR** (101 MHz, CD_2Cl_2) δ = 198.8, 156.8, 155.1, 143.3, 138.4, 137.0, 134.7, 134.3, 129.4, 127.9, 127.7, 127.5, 127.4, 127.35, 127.28, 127.1, 127.0, 126.9, 125.2, 124.6, 124.3, 114.4, 106.44, 106.39, 100.3, 99.3, 95.1, 91.1, 55.9, 55.5, 42.8, 37.2, 37.1, 26.4, 26.1, 24.5, 22.83, 22.78 ppm; **IR** (ATR) ṽ = 2934 (w), 2056 (s), 1618 (w), 1597 (w), 1449 (m), 1423 (w), 1411 (w), 1329 (w), 1295 (w), 1240 (w), 1226 (w), 1196 (w), 1166 (w), 1114 (w), 1020 (w), 952 (w), 865 (w), 833 (w), 747 (w), 702 (w), 466 (w) cm^{-1}; **HRMS** (ESI-APCI/TOF) *m/z* = calcd. for $[C_{41}H_{37}CoIO_2]^+$, $[M\text{-}CO\text{-}I^-]^+$: 747.1170, found: 747.1170; $[\alpha]_D^{23}$ = -47.3 (*c* = 0.4, CH_2Cl_2).

5.2 Pentasubstituted Cp^V Cobalt Complexes

***General Procedure 3* – Cobalt(III) Complexation of Chiral Cyclopentadienes.**

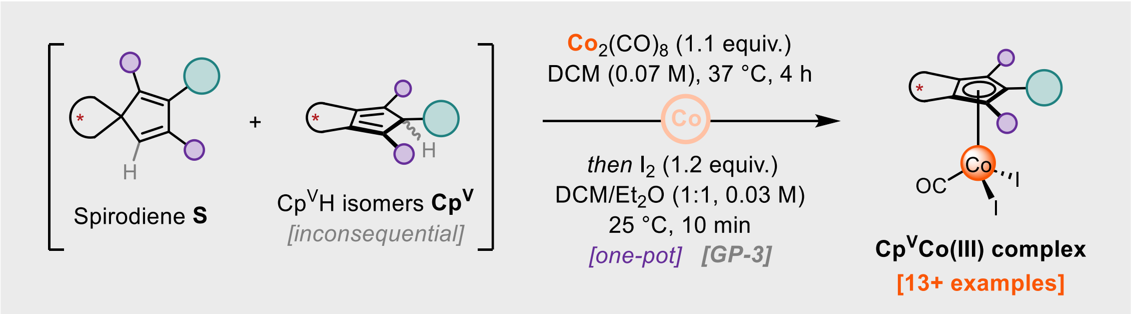

Scheme S17. Cobalt(III) complexation of chiral cyclopentadienes toward (usually) Cp^{V}Co(CO)I_2-type monomers.

Step 1. In a nitrogen-filled glovebox, an oven-dried microwave vial was charged with dicobalt octacarbonyl (1.1 equiv.). A mixture of pentasubstituted chiral cyclopentadiene isomers **Cp^V** and spirodiene **S** (1 equiv. of Cp^{V}H isomers, as determined by qNMR) was dissolved in anhydrous degassed DCM (15 mL/mmol), and added. The vial was capped, brought outside of the glovebox, and connected to a Schlenk line. The black reaction mixture was stirred in a heating block at 37 °C for 4 hours.

Step 2. After cooling to room temperature (25 °C), a solution of iodine (1.2 equiv.) in anhydrous degassed Et_2O (15 mL/mmol) was added to the reaction mixture, and it was stirred for >10 min. The vial was kept connected to a Schlenk line considering the instantaneous evolution of carbon monoxide gas. After careful purging of the head space with nitrogen inside a well-ventilated fume hood, the vial was opened, and all volatiles were removed *in vacuo*. The black residue was purified by flash column chromatography on silica gel (dry loading, 5 cm column height, gradient: pentane/DCM = 100:0 *then* 80:20 *then* 50:50), which was performed rapidly to minimize complex decomposition on the silica (i.e. turning blue). Notably, the DCM gradient was only applied as soon as a dark brown band had completely eluted with pentane. At 80:20 ratio (pentane/DCM), a yellow band quickly eluted, after which a 50:50 eluent ratio provided the desired dark purple fractions. These were collected, combined, and all volatiles were removed *in vacuo*, affording the corresponding chiral Cp^V cobalt(III) complex as a black solid. It typically exists as a single Cp^{V}Co(CO)I_2-type monomeric species. Usually, the complex was sufficiently pure for most purposes but, if required, it could be further purified by trituration with *i*Pr_2O.

Note: Investigation of the reaction solvent showed similar yields when the DCM was first evaporated before addition of the ethereal iodine solution, or when the iodine was dissolved in DCM instead of Et_2O. Moreover, the reported use of a CO atmosphere did not improve the yield.[26] Therefore, above protocol (without solvent evaporation and under N_2 atmosphere) is preferred due to its operational simplicity.

Cobalt complex (R_a)-**Co1a**

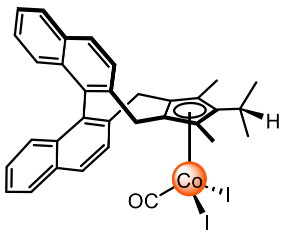

According to *General Procedure 3*, starting from an isolated mixture of Cp^VH isomers **Cp^V1** and spirodiene **S1** (56:44 ratio, 748 µmol of Cp^VH), chiral Co(III)-complex **Co1a** (366 mg, 485 µmol, 65% yield over 2 steps) was obtained as a black solid. The cobalt complex exists as a single 18-electron monomeric species. A suitable crystal for X-ray analysis (**Figure S6**) was obtained by slow evaporation of a concentrated solution in chloroform.

^{1}H NMR (400 MHz, CD_2Cl_2) δ = 8.11 (s, 2H), 8.00 (d, *J* = 8.3 Hz, 1H), 7.97 – 7.92 (m, 2H), 7.53 – 7.45 (m, 2H), 7.35 – 7.18 (m, 4H), 7.06 (d, *J* = 8.5 Hz, 1H), 3.65 (d, *J* = 16.2 Hz, 1H), 3.54 (d, *J* = 14.5 Hz, 1H), 3.48 (d, *J* = 14.5 Hz, 1H), 3.18 (hept, *J* = 7.2 Hz, 1H), 3.01 (d, *J* = 16.2 Hz, 1H), 2.66 (s, 3H), 2.32 (s, 3H), 1.59 (d, *J* = 7.0 Hz, 3H), 1.20 (d, *J* = 7.2 Hz, 3H) ppm; **^{13}C{^{1}H} NMR** (101 MHz, CD_2Cl_2) δ = 200.1, 136.6, 135.9, 134.6, 133.5, 133.21, 133.16, 132.5, 132.2, 130.3, 129.8, 129.6, 128.7, 128.5, 127.2, 126.83, 126.81, 126.64, 126.58, 126.4, 110.0, 109.3, 101.6, 100.3, 97.0, 30.3, 30.2, 27.1, 22.1, 22.0, 14.2, 12.1 ppm; **IR** (ATR) ṽ = 3051 (w), 2961 (w), 2923 (m), 2852 (w), 2049 (s), 1508 (w), 1460 (w), 1368 (w), 1261 (w), 1248 (w), 1058 (w), 1024 (w), 814 (m), 775 (w), 753 (m), 737 (w), 704 (w), 501 (w), 466 (w) cm^{-1}; **HRMS** (ESI-APCI/TOF) *m/z* = calcd. for $[C_{32}H_{29}CoI]^+$, $[M\text{-}CO\text{-}I^-]^+$: 599.0640, found: 599.0642; $[\boldsymbol{\alpha}]_D^{26}$ = -476.3 (*c* = 0.05, $CHCl_3$); **XRD** (Cu$K\alpha$, R_1 = 1.66%) CCDC: 2202057.

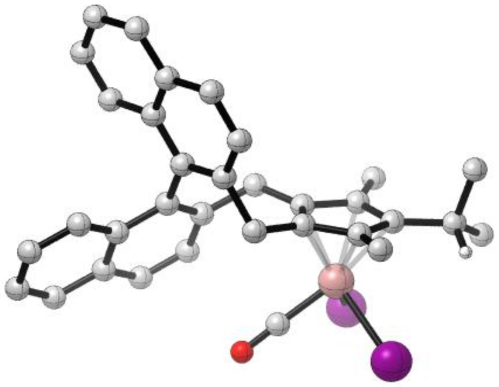

Figure S6. Solid-state X-ray structure of (R_a)-**Co1a** (CCDC: 2202057) showing 50% probability thermal ellipsoids. Hydrogen atoms (except for on the central *i*Pr carbon) are omitted for clarity.

Cobalt complex (R_a)-**Co2**

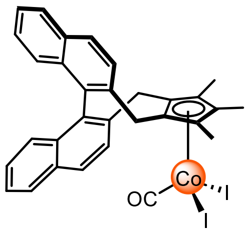

According to *General Procedure 3*, starting from an isolated mixture of Cp^VH isomers **Cp^V3** and spirodiene **S3** (94:6 ratio, 978 µmol of Cp^VH), chiral Co(III)-complex **Co2** (298 mg, 410 µmol, 42% yield over 2 steps) was obtained as a black solid. The cobalt complex exists as a single 18-electron monomeric species.

^{1}H NMR (600 MHz, CD_2Cl_2) δ = 8.11 (s, 2H), 8.02 – 7.99 (m, 1H), 7.96 – 7.92 (m, 2H), 7.52 – 7.46 (m, 2H), 7.33 – 7.24 (m, 3H), 7.22 – 7.18 (m, 1H), 7.05 (d, *J* = 8.5 Hz, 1H), 3.71 (d, *J* = 16.4 Hz, 1H), 3.54 (d, *J* = 14.6 Hz, 1H), 3.47 (d, *J* = 14.6 Hz, 1H), 3.07 (d, *J* = 16.3 Hz, 1H), 2.49 (s, 3H), 2.39 (s, 3H), 2.32 (s, 3H) ppm; **^{13}C{^{1}H} NMR** (151 MHz, CD_2Cl_2) δ = 200.7, 136.6, 135.8, 134.8, 133.5, 133.2, 132.9,

132.5, 132.2, 130.2, 129.7, 129.6, 128.7, 128.5, 127.21, 127.19, 126.9, 126.8, 126.6, 126.5, 126.4, 108.2, 103.7, 101.8, 99.9, 97.3, 30.6, 30.4, 13.1, 12.1, 12.0 ppm; **IR** (ATR) ṽ = 3053 (w), 2048 (s), 1508 (w), 1460 (w), 1422 (w), 1374 (w), 1024 (w), 907 (m), 825 (w), 813 (w), 752 (w), 730 (m), 708 (w), 647 (w), 500 (w), 485 (w), 466 (w) cm^{-1}; **HRMS** (ESI/QTOF) *m/z* = calcd. for $[C_{30}H_{25}CoI]^+$, $[M-CO-I^-]^+$: 571.0327, found: 571.0333; $[\alpha]_D^{22}$ = -230.8 (*c* = 0.10, $CHCl_3$).

Cobalt complex (R_a)-**Co3**

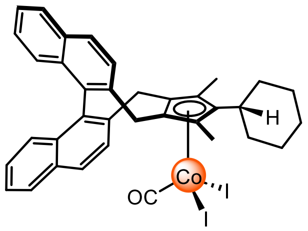

According to *General Procedure 3*, starting from an isolated mixture of Cp^VH isomers **Cp^{V}4** and spirodiene **S4** (60:40 ratio, 803 µmol of Cp^VH), chiral Co(III)-complex **Co3** (363 mg, 457 µmol, 57% yield over 2 steps) was obtained as a black solid. The cobalt complex exists as a single 18-electron monomeric species.

^{1}H NMR (400 MHz, CD_2Cl_2) δ = 8.13 – 8.07 (m, 2H), 8.00 (d, *J* = 8.2 Hz, 1H), 7.97 – 7.92 (m, 2H), 7.52 – 7.46 (m, 2H), 7.33 – 7.19 (m, 4H), 7.06 (d, *J* = 8.4 Hz, 1H), 3.65 (d, *J* = 16.2 Hz, 1H), 3.55 (d, *J* = 14.5 Hz, 1H), 3.48 (d, *J* = 14.5 Hz, 1H), 3.01 (d, *J* = 16.2 Hz, 1H), 2.79 – 2.70 (m, 1H), 2.66 (s, 3H), 2.51 – 2.43 (m, 1H), 2.33 (s, 3H), 1.91 – 1.65 (m, 5H), 1.50 – 1.18 (m, 4H) ppm; **^{13}C{^{1}H} NMR** (101 MHz, CD_2Cl_2) δ = 200.1, 136.7, 135.9, 134.7, 133.5, 133.21, 133.20, 132.5, 132.2, 130.3, 129.8, 129.6, 128.7, 128.5, 127.2, 126.84, 126.81, 126.7, 126.6, 126.4, 110.1, 107.8, 101.6, 100.3, 97.0, 38.0, 33.0, 31.9, 30.4, 30.3, 27.3, 27.2, 26.5, 14.6, 12.2 ppm; **IR** (ATR) ṽ = 3051 (w), 2926 (m), 2852 (w), 2049 (s), 1507 (w), 1447 (m), 1370 (w), 1264 (w), 1248 (w), 1142 (w), 1025 (w), 815 (m), 775 (w), 756 (m), 737 (w), 501 (w), 466 (w) cm^{-1}; **HRMS** (APCI/QTOF) *m/z* = calcd. for $[C_{35}H_{33}CoI]^+$, $[M-CO-I^-]^+$: 639.0953, found: 639.0978; $[\alpha]_D^{22}$ = -406.7 (*c* = 0.06, $CHCl_3$).

Cobalt complex (R_a)-**Co4**

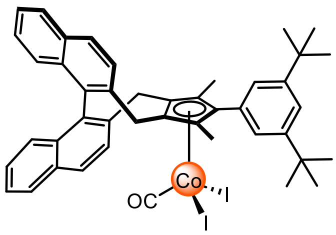

According to *General Procedure 3*, starting from an isolated mixture of Cp^VH isomers **Cp^{V}6** and spirodiene **S6** (77:23 ratio, 444 µmol of Cp^VH), chiral Co(III)-complex **Co4** (167 mg, 185 µmol, 42% yield over 2 steps) was obtained as a black solid. The cobalt complex exists as a single 18-electron monomeric species.

^{1}H NMR (400 MHz, CD_2Cl_2) δ = 8.16 – 8.11 (m, 2H), 8.04 – 7.99 (m, 2H), 7.96 (d, *J* = 8.2 Hz, 1H), 7.54 – 7.47 (m, 5H), 7.45 (d, *J* = 8.4 Hz, 1H), 7.34 – 7.25 (m, 2H), 7.25 – 7.21 (m, 1H), 7.11 – 7.07 (m, 1H), 3.85 (d, *J* = 14.6 Hz, 1H), 3.77 (d, *J* = 16.2 Hz, 1H), 3.57 (d, *J* = 14.5 Hz, 1H), 3.15 (d, *J* = 16.2 Hz, 1H), 2.59 (s, 3H), 2.41 (s, 3H), 1.37 (s, 18H) ppm; **^{13}C{^{1}H} NMR** (126 MHz, CD_2Cl_2) δ = 200.1, 151.1, 136.6, 136.1, 134.9, 133.5, 133.3, 133.1, 132.5, 132.2, 130.5, 129.7, 129.6, 128.7, 128.5, 127.7, 127.23, 127.20, 127.0, 126.9, 126.80, 126.77, 126.6, 126.4, 123.6, 110.8, 105.2, 102.9, 100.2, 96.7, 35.4, 31.5, 30.9, 30.7, 13.9, 13.5 ppm; **IR** (ATR) ṽ = 2960 (s), 2924 (m), 2865 (w), 2055 (m), 1593 (w), 1475 (w), 1447 (w), 1394 (w), 1363 (w), 1248 (w), 1025 (w), 881 (w), 814 (m), 754 (m), 738 (w), 716 (w), 472 (w), 452 (w), 430 (w), 418 (w) cm^{-1}; **HRMS** (ESI-APCI/TOF) *m/z* = calcd. for $[C_{43}H_{43}CoI]^+$, $[M-CO-I^-]^+$: 745.1736, found: 745.1737; $[\alpha]_D^{22}$ = -425.3 (*c* = 0.05, $CHCl_3$).

Cobalt complex (*R*$_a$)-**Co5**

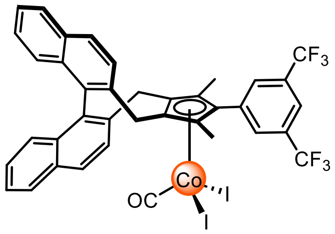

According to *General Procedure 3*, starting from an isolated mixture of Cp^{V}H isomers **Cp^{V}7** and spirodiene **S7** (87:13 ratio, 171 µmol of Cp^{V}H), chiral Co(III)-complex **Co5** (25 mg, 26 µmol, 15% yield over 2 steps) was obtained as a black solid. The cobalt complex exists as a single 18-electron monomeric species.

^{1}H NMR (400 MHz, CD_2Cl_2) δ = 8.19 – 8.14 (m, 3H), 8.09 – 7.95 (m, 5H), 7.56 – 7.48 (m, 2H), 7.43 (d, *J* = 8.4 Hz, 1H), 7.35 – 7.27 (m, 2H), 7.22 (d, *J* = 8.3 Hz, 1H), 7.11 (d, *J* = 8.1 Hz, 1H), 3.66 – 3.60 (m, 2H), 3.54 (d, *J* = 14.5 Hz, 1H), 3.09 (d, *J* = 16.1 Hz, 1H), 2.70 (s, 3H), 2.50 (s, 3H) ppm; **^{13}C{^{1}H} NMR** (126 MHz, CD_2Cl_2) δ = 199.6, 136.7, 136.1, 133.9, 133.6, 133.4, 133.0, 132.9 – 132.8 (m), 132.5, 132.3, 132.1, 132.0 (q, *J* = 33.6 Hz), 130.1, 129.9, 129.8, 128.8, 128.6, 127.4, 127.2, 127.1, 126.9, 126.75, 126.69, 126.6, 123.6 (q, *J* = 273.0 Hz), 123.6 – 123.5 (m), 112.8, 102.5, 101.2, 100.0, 96.9, 30.3, 30.0, 14.0, 13.9 ppm; **^{19}F{^{1}H} NMR** (376 MHz, CD_2Cl_2) δ = -63.25 ppm; **IR** (ATR) ṽ = 2061 (m), 1346 (m), 1278 (s), 1172 (m), 1136 (s), 1109 (w), 902 (w), 815 (w), 756 (w), 734 (w), 709 (w), 468 (w) cm^{-1}; **HRMS** (nanochip-ESI/LTQ-Orbitrap) *m/z* = calcd. for $[C_{37}H_{25}CoF_6I]^+$, $[M\text{-}CO\text{-}I^-]^+$: 769.0232, found: 769.0228; $[\alpha]_D^{26}$ = -334.4 (*c* = 0.06, $CHCl_3$).

Cobalt complex (*R*$_a$)-**Co6**

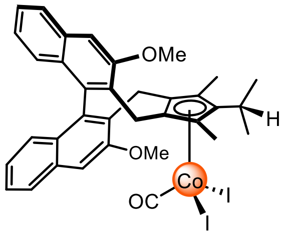

According to *General Procedure 3*, starting from an isolated mixture of Cp^{V}H isomers **Cp^{V}10** and spirodiene **S10** (45:55 ratio, 170 µmol of Cp^{V}H), chiral Co(III)-complex **Co6** (73 mg, 90 µmol, 53% yield over 2 steps) was obtained as a black solid. Notably, the complexation was repeated successfully on a larger scale (881 µmol), affording 358 mg (440 µmol, 50% yield over 2 steps) of **Co6**. The cobalt complex exists as a mixture of two catalytically equivalent species (i.e. with and without CO ligand, in 3:1 ratio). For ^{1}H NMR, the integration is calibrated with respect to the major species (and thus 0.33H integration per proton for the minor species). A suitable crystal for X-ray analysis (**Figure S7**) was obtained by slow evaporation of a concentrated solution in DCM.

^{1}H NMR (400 MHz, CD_2Cl_2) δ = 7.90 – 7.86 (m, 1.33H), 7.85 – 7.81 (m, 1.33H), 7.46 – 7.38 (m, 4H), 7.29 (s, 1.33H), 7.14 – 7.07 (m, 2.66H), 7.07 – 7.03 (m, 1.33H), 7.00 – 6.95 (m, 1.33H), 4.24 (d, *J* = 15.8 Hz, 0.33H), 4.13 – 4.06 (m, 5H), 4.01 – 3.95 (m, 4.33H), 3.87 – 3.79 (m, 1.33H), 3.54 (d, *J* = 14.0 Hz, 1H), 3.15 (hept, *J* = 7.1 Hz, 1H), 2.90 (hept, *J* = 7.2 Hz, 0.33H), 2.65 – 2.60 (m, 3.33H), 2.57 (d, *J* = 16.0 Hz, 1H), 2.53 (s, 1H), 2.52 (s, 1H), 2.40 (s, 3H), 1.47 (d, *J* = 7.1 Hz, 3H), 1.37 (d, *J* = 7.1 Hz, 1H), 1.28 (d, *J* = 7.2 Hz, 3H), 1.20 (d, *J* = 7.1 Hz, 1H) ppm; **^{13}C{^{1}H} NMR** (101 MHz, CD_2Cl_2) δ = 200.1, 156.6, 156.5, 155.5, 155.4, 138.8, 138.7, 137.5, 137.4, 134.6, 134.4, 134.3, 134.2, 128.5, 127.8, 127.5, 127.42, 127.37, 127.24, 127.18, 127.1, 126.99, 126.96, 126.9, 126.82, 126.77, 124.7, 124.6, 124.4, 124.2, 123.9, 108.1, 107.2, 106.3, 106.25, 106.20, 105.4, 105.2, 104.2, 102.0, 100.8, 97.0, 96.6, 55.8, 55.69, 55.65, 55.5, 27.2, 26.8, 26.1, 24.5, 23.2, 22.9, 22.4, 22.2, 21.9, 21.7, 14.4, 13.3, 12.9, 11.4 ppm; **IR** (ATR) ṽ = 2962 (w), 2929 (w), 2050 (s), 1619 (w), 1597 (w), 1451 (m), 1425 (w), 1387 (w), 1365 (w), 1329 (w), 1295 (w), 1237 (w), 1222 (w), 1197 (w), 1162 (w), 1150 (w), 1109 (m), 1020 (w), 865 (w), 832 (w), 747 (w), 471 (w) cm^{-1}; **HRMS**

(ESI/QTOF) m/z = calcd. for $[C_{34}H_{33}CoIO_2]^+$, $[M-CO-I^-]^+$: 659.0852, found: 659.0862; $[\alpha]_D^{26}$ = -591.7 (c = 0.04, $CHCl_3$); **XRD** (Cu$K\alpha$, R_1 = 4.84%) CCDC: 2310524.

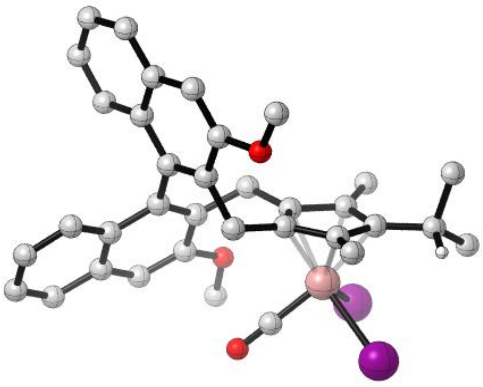

Figure S7. Solid-state X-ray structure of (R_a)-**Co6** (CCDC: 2310524) showing 50% probability thermal ellipsoids. Hydrogen atoms (except for on the central *i*Pr carbon) and disorder are omitted for clarity.

Cobalt complex (R_a)-**Co7**

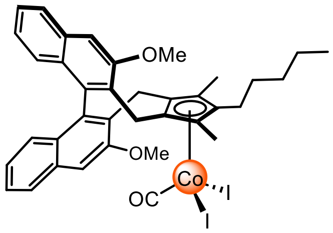

According to *General Procedure 3*, starting from an isolated mixture of Cp^{V}H isomers **Cp^{V}11** and spirodiene **S11** (62:38 ratio, 943 µmol of Cp^{V}H), chiral Co(III)-complex **Co7** (442 mg, 524 µmol, 56% yield over 2 steps) was obtained as a black solid. The cobalt complex exists as a single 18-electron monomeric species.

^{1}H NMR (600 MHz, $CDCl_3$) δ = 7.85 (d, J = 8.2 Hz, 1H), 7.81 (d, J = 8.2 Hz, 1H), 7.45 – 7.40 (m, 2H), 7.35 (s, 1H), 7.25 (s, 1H), 7.16 – 7.07 (m, 3H), 7.00 (d, J = 8.4 Hz, 1H), 4.12 (s, 3H), 4.08 (d, J = 16.1 Hz, 1H), 3.98 (s, 3H), 3.81 (d, J = 14.2 Hz, 1H), 3.75 (d, J = 14.3 Hz, 1H), 2.82 – 2.75 (m, 1H), 2.62 (d, J = 16.1 Hz, 1H), 2.59 (s, 3H), 2.53 – 2.47 (m, 1H), 2.21 (s, 3H), 1.43 – 1.21 (m, 6H), 0.88 (t, J = 7.0 Hz, 3H) ppm; **^{13}C{^{1}H} NMR** (151 MHz, $CDCl_3$) δ = 199.2, 156.0, 155.0, 138.3, 137.5, 134.2, 133.7, 127.9, 127.3, 127.25, 127.21, 127.1, 126.9, 126.83, 126.76, 126.6, 124.5, 124.1, 123.8, 106.2, 105.9, 105.3, 103.4, 103.1, 102.0, 95.4, 55.45, 55.42, 31.9, 29.9, 27.0, 25.0, 22.8, 22.6, 14.1, 12.2, 11.9 ppm; **IR** (ATR) ṽ = 2954 (w), 2925 (w), 2855 (w), 2048 (s), 1618 (w), 1596 (w), 1450 (m), 1423 (w), 1411 (w), 1388 (w), 1367 (w), 1330 (w), 1294 (w), 1237 (w), 1222 (w), 1197 (w), 1162 (w), 1150 (w), 1107 (m), 1018 (m), 909 (w), 865 (w), 832 (w), 731 (m), 468 (w) cm^{-1}; **HRMS** (ESI/QTOF) m/z = calcd. for $[C_{36}H_{37}CoIO_2]^+$, $[M-CO-I^-]^+$: 687.1165, found: 687.1174; $[\alpha]_D^{22}$ = -286.1 (c = 0.09, $CHCl_3$).

Cobalt complex (R_a)-**Co8**

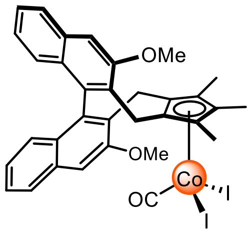

According to *General Procedure 3*, starting from an isolated mixture of Cp^{V}H isomers **Cp^{V}12** and spirodiene **S12** (92:8 ratio, 638 µmol of Cp^{V}H), chiral Co(III)-complex **Co8** (241 mg, 306 µmol, 48% yield over 2 steps) was obtained as a black solid with the characterization data matching those previously reported.[25,26] The cobalt complex exists as a single 18-electron monomeric species.

^{1}H NMR (400 MHz, $CDCl_3$) δ = 7.85 (d, *J* = 8.3 Hz, 1H), 7.80 (d, *J* = 8.1 Hz, 1H), 7.45 – 7.39 (m, 2H), 7.36 (s, 1H), 7.24 (s, 1H), 7.15 – 7.06 (m, 3H), 6.97 (d, *J* = 8.6 Hz, 1H), 4.12 (s, 3H), 4.08 (d, *J* = 16.0 Hz, 1H), 3.98 (s, 3H), 3.82 (d, *J* = 14.2 Hz, 1H), 3.67 (d, *J* = 14.2 Hz, 1H), 2.62 – 2.57 (m, 4H), 2.33 (s, 3H), 2.24 (s, 3H) ppm; $[\alpha]_D^{22}$ = -193.9 (*c* = 0.13, $CHCl_3$).

Cobalt complex (R_a)-**Co9**

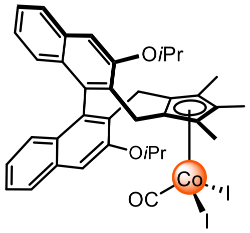

According to *General Procedure 3*, starting from an isolated mixture of Cp^VH isomers **Cp^V15** and spirodiene **S15** (96:4 ratio, 574 µmol of Cp^VH), chiral Co(III)-complex **Co9** (149 mg, 176 µmol, 31% yield over 2 steps) was obtained as a black solid. The cobalt complex exists as a single 18-electron monomeric species.

^{1}H NMR (600 MHz, CD_2Cl_2) δ = 7.87 – 7.84 (m, 1H), 7.78 – 7.76 (m, 1H), 7.44 – 7.35 (m, 3H), 7.28 (s, 1H), 7.09 – 7.03 (m, 2H), 6.92 – 6.87 (m, 2H), 4.91 (hept, *J* = 6.0 Hz, 1H), 4.85 (hept, *J* = 6.1 Hz, 1H), 4.16 (d, *J* = 15.9 Hz, 1H), 3.88 (d, *J* = 14.3 Hz, 1H), 3.47 (d, *J* = 14.3 Hz, 1H), 2.83 (s, 3H), 2.57 (d, *J* = 15.9 Hz, 1H), 2.31 (s, 3H), 2.22 (s, 3H), 1.71 (d, *J* = 6.1 Hz, 3H), 1.56 (d, *J* = 6.0 Hz, 3H), 1.49 – 1.46 (m, 6H) ppm; **^{13}C{^{1}H} NMR** (151 MHz, CD_2Cl_2) δ = 200.1, 154.7, 153.4, 138.9, 137.8, 134.6, 134.3, 128.3, 127.31, 127.27, 127.1, 127.0, 126.9, 126.8, 126.7, 125.2, 124.3, 124.1, 108.2, 108.0, 107.9, 104.6, 100.4, 99.3, 96.6, 71.5, 71.1, 24.6, 23.0, 22.6, 22.4, 22.3, 22.2, 14.2, 12.5, 11.8 ppm; **IR** (ATR) ṽ = 2976 (w), 2928 (w), 2051 (s), 1618 (w), 1594 (m), 1430 (m), 1385 (w), 1374 (w), 1328 (w), 1291 (w), 1235 (w), 1221 (w), 1197 (w), 1163 (w), 1104 (m), 1061 (w), 945 (w), 908 (w), 834 (w), 747 (w), 731 (m), 468 (w) cm^{-1}; **HRMS** (nanochip-ESI/LTQ-Orbitrap) *m*/*z* = calcd. for $[C_{36}H_{37}CoIO_2]^+$, $[M\text{-}CO\text{-}I^-]^+$: 687.1165, found: 687.1168; $[\alpha]_D^{22}$ = -324.5 (*c* = 0.07, $CHCl_3$).

Cobalt complex (R_a)-**Co10**

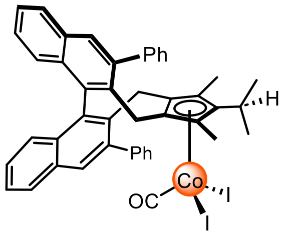

According to *General Procedure 3*, starting from an isolated mixture of Cp^VH isomers (R_a)-**Cp^V17** and spirodiene (R_a)-**S17** (41:59 ratio, 252 µmol of Cp^VH), chiral Co(III)-complex **Co10** (93 mg, 103 µmol, 41% yield over 2 steps) was obtained as a black solid. The cobalt complex exists as a mixture of two catalytically equivalent species (i.e. with and without CO ligand, in 2:1 ratio). For ^{1}H NMR, the integration is calibrated with respect to the major species (and thus 0.5H integration per proton for the minor species).

^{1}H NMR (400 MHz, CD_2Cl_2) δ = 8.02 – 7.96 (m, 2.5H), 7.95 – 7.89 (m, 1.5H), 7.86 (s, 1H), 7.82 (s, 1H), 7.61 – 7.57 (m, 0.5H), 7.56 – 7.38 (m, 13.5H), 7.36 – 7.20 (m, 7.5H), 7.18 – 7.11 (m, 1.5H), 6.99 (d, *J* = 8.5 Hz, 1H), 4.29 (d, *J* = 14.5 Hz, 1H), 4.17 (d, *J* = 16.5 Hz, 1H), 4.04 (d, *J* = 14.8 Hz, 0.5H), 3.96 – 3.87 (m, 1.5H), 3.35 (d, *J* = 17.2 Hz, 0.5H), 3.09 (hept, *J* = 6.8 Hz, 0.5H), 2.99 (d, *J* = 16.5 Hz, 1H), 2.71 (hept, *J* = 7.1 Hz, 1H), 1.86 (s, 3H), 1.50 (d, *J* = 17.3 Hz, 0.5H), 1.35 (s, 3H), 1.31 – 1.26 (m, 4.5H), 1.19 (d, *J* = 7.0 Hz, 3H), 1.09 (d, *J* = 7.1 Hz, 3H), 1.04 (d, *J* = 7.1 Hz, 1.5H), 0.18 (s, 1.5H) ppm; **^{13}C{^{1}H} NMR** (101 MHz, CD_2Cl_2) δ = 199.7, 142.2, 141.8, 141.7, 141.3, 141.1, 140.4, 140.3, 140.0, 138.8, 138.4, 137.8, 137.3, 133.9, 133.0, 132.9, 132.8, 132.2, 131.9, 131.8, 131.6, 131.5, 131.0, 130.7, 130.2, 130.1, 130.0, 129.7, 129.2, 129.1, 128.62, 128.56, 128.4, 128.3, 128.2, 127.6, 127.2, 127.10, 127.06, 127.0, 126.94, 126.86, 126.83, 126.75, 106.7, 105.5, 104.6, 103.4, 103.0, 99.0, 97.5, 96.2, 93.4, 93.2, 29.4, 28.1, 27.1, 26.8, 26.6, 26.5, 22.2,

22.1, 21.6, 20.5, 12.5, 12.3, 10.7, 10.5 ppm; **IR** (ATR) ṽ = 3054 (w), 2961 (w), 2925 (w), 2868 (w), 2049 (s), 1589 (w), 1493 (w), 1447 (w), 1367 (w), 1264 (w), 1074 (w), 1026 (w), 893 (w), 784 (w), 752 (m), 736 (m), 703 (s), 471 (w) cm^{-1}; **HRMS** (ESI/APCI) *m/z* = calcd. for $[C_{44}H_{37}CoI]^+$, $[M\text{-}CO\text{-}I^-]^+$: 751.1266, found: 751.1267.

Cobalt complex (*R*)-**Co11**

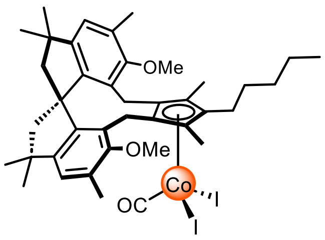

According to *General Procedure 3*, starting from an isolated mixture of Cp^VH isomers **Cp^V19** and spirodiene **S19** (98:2 ratio, 451 µmol of Cp^VH), chiral Co(III)-complex **Co11** (211 mg, 236 µmol, 52% yield over 2 steps) was obtained as a black solid. The cobalt complex exists as a mixture of two catalytically equivalent species (i.e. with and without CO ligand, in 1:1.2 ratio). For ^1H NMR, the integration is calibrated with respect to the minor species (and thus 1.2H integration per proton for the major species).

^{1}H NMR (400 MHz, CD_2Cl_2) δ = 6.94 – 6.92 (m, 3.2H), 6.88 (s, 1.2H), 4.53 (d, *J* = 14.0 Hz, 1H), 3.83 (s, 3H), 3.73 (s, 3H), 3.69 (s, 3.6H), 3.65 (d, *J* = 14.8 Hz, 1.2H), 3.61 (s, 3.6H), 3.56 (d, *J* = 14.7 Hz, 1.2H), 3.49 (d, *J* = 13.9 Hz, 1H), 3.24 (d, *J* = 12.4 Hz, 1H), 3.20 (d, *J* = 13.1 Hz, 1.2H), 3.04 – 2.96 (m, 1.2H), 2.88 (d, *J* = 14.2 Hz, 1H), 2.79 – 2.71 (m, 1H), 2.60 (s, 3H), 2.57 (d, *J* = 13.2 Hz, 1.2H), 2.52 (d, *J* = 12.9 Hz, 1.2H), 2.37 (d, *J* = 13.1 Hz, 2H), 2.32 – 2.26 (m, 4H), 2.22 (s, 3H), 2.19 – 2.09 (m, 8.2H), 2.01 (s, 3.6H), 1.97 (s, 3H), 1.92 – 1.84 (m, 2.2H), 1.79 (s, 3.6H), 1.48 – 1.44 (m, 10.8H), 1.43 (s, 3H), 1.38 (s, 3H), 1.34 (s, 3H), 1.32 (s, 3.6H), 1.31 (s, 3.6H), 1.30 – 1.04 (m, 16.2H), 0.89 (t, *J* = 6.8 Hz, 3H), 0.84 (t, *J* = 7.0 Hz, 3.6H) ppm; **^{13}C{^{1}H} NMR** (101 MHz, CD_2Cl_2) δ = 198.8, 157.6, 157.5, 157.3, 149.1, 147.7, 147.5, 147.1, 147.0, 146.7, 146.1, 146.0, 132.1, 131.5, 131.3, 131.2, 125.5, 125.3, 125.0, 124.9, 124.6, 124.5, 123.9, 123.2, 110.3, 107.2, 105.7, 100.9, 99.0, 97.1, 96.4, 94.0, 91.1, 87.6, 60.6, 60.5, 60.3, 59.6, 59.5, 59.2, 58.8, 58.6, 57.6, 57.2, 42.6, 42.1, 41.9, 41.4, 32.6, 32.1, 32.0, 31.9, 31.7, 31.3, 31.22, 31.17, 31.1, 30.4, 29.0, 28.5, 28.3, 25.7, 24.7, 22.9, 22.8, 20.8, 20.3, 17.8, 17.1, 16.64, 16.62, 14.1, 14.0, 12.7, 12.5, 11.2, 10.7 ppm; **IR** (ATR) ṽ = 2954 (s), 2924 (s), 2859 (m), 2058 (s), 1463 (s), 1405 (w), 1362 (w), 1325 (w), 1313 (w), 1263 (w), 1215 (m), 1164 (w), 1091 (m), 1061 (w), 995 (m), 920 (w), 875 (w), 735 (w), 466 (w) cm^{-1}; **HRMS** (ESI/QTOF) *m/z* = calcd. for $[C_{39}H_{51}CoIO_2]^+$, $[M\text{-}CO\text{-}I^-]^+$: 737.2260, found: 737.2271; $[\alpha]_D^{26}$ = -332.4 (*c* = 0.07, $CHCl_3$).

Cobalt complex (*R*)-**Co12**

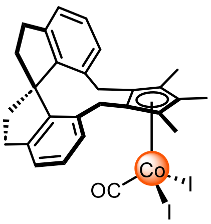

According to *General Procedure 3*, starting from an isolated mixture of Cp^VH isomers **Cp^V31** and spirodiene **S31** (99:1 ratio, 743 µmol of Cp^VH), chiral Co(III)-complex **Co12** (297 mg, 429 µmol, 58% yield over 2 steps) was obtained as a black solid. The cobalt complex exists as a single 18-electron monomeric species. A suitable crystal for X-ray analysis (**Figure S8**) was obtained by slow vapor diffusion (*ca.* two days) of pentane into a concentrated solution in chloroform.

^{1}H NMR (600 MHz, CD_2Cl_2) δ = 7.92 (d, *J* = 7.3 Hz, 1H), 7.34 – 7.28 (m, 2H), 7.18 – 7.15 (m, 1H), 7.12 (t, *J* = 7.5 Hz, 1H), 6.78 (d, *J* = 7.5 Hz, 1H), 3.87 (d, *J* = 13.7 Hz, 1H), 3.31 (d, *J* = 13.7 Hz, 1H), 3.25 (d, *J* = 15.2 Hz, 1H), 3.13 (d, *J* = 15.2 Hz, 1H), 3.06 – 2.94 (m, 3H), 2.91 – 2.85 (m, 1H), 2.68 (s, 3H), 2.46 (s, 3H), 2.32 (s, 3H), 2.31 – 2.26 (m, 2H), 2.01 – 1.86 (m, 2H) ppm; **^{13}C{^{1}H} NMR** (151 MHz, CD_2Cl_2) δ = 199.5, 147.93,

147.87, 144.5, 143.8, 133.0, 132.9, 132.4, 128.6, 128.5, 128.0, 125.2, 124.7, 112.7, 101.2, 100.7, 99.6, 98.4, 61.9, 39.6, 38.4, 30.4, 30.3, 26.00, 25.99, 14.1, 13.4, 11.5 ppm; **IR** (ATR) ṽ = 2945 (w), 2853 (w), 2054 (s), 1450 (w), 1430 (w), 1369 (w), 1014 (w), 909 (w), 786 (w), 758 (w), 730 (m), 495 (w), 463 (w) cm^{-1}; **HRMS** (nanochip-ESI/LTQ-Orbitrap) *m/z* = calcd. for $[C_{27}H_{27}CoI]^+$, $[M\text{-}CO\text{-}I^-]^+$: 537.0484, found: 537.0493; $[\boldsymbol{\alpha}]_{\mathbf{D}}^{22}$ = -570.4 (*c* = 0.04, $CHCl_3$); **XRD** (Mo*Kα*, R_1 = 2.57%) CCDC: 2506334.

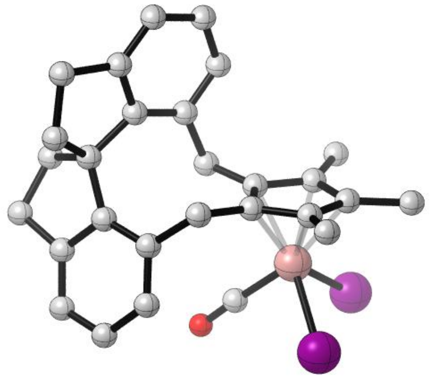

Figure S8. Solid-state X-ray structure of (*R*)-**Co12** (CCDC: 2506334) showing 50% probability thermal ellipsoids. Hydrogen atoms are omitted for clarity.

Cobalt complex (*R*)-**Co13a**

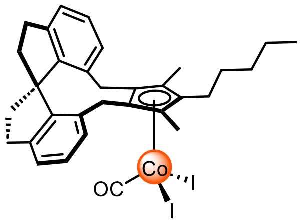

According to *General Procedure 3*, starting from an isolated mixture of Cp^VH isomers **Cp^V32** and spirodiene **S32** (97:3 ratio, 3.24 mmol of Cp^VH), chiral Co(III)-complex **Co13a** (1.415 g, 1.89 mmol, 58% yield over 2 steps) was obtained as a black solid (**Figure S9**). Notably, this gram-scale cobalt complexation proceeded with similar efficiency as for smaller scales and did not exhibit practical constraints (yet the oxidation step with concomitant CO gas evolution should be performed carefully inside a well-ventilated fume hood). The cobalt complex exists as a single 18-electron monomeric species.

^{1}H NMR (600 MHz, CD_2Cl_2) δ = 7.90 (d, *J* = 7.3 Hz, 1H), 7.34 – 7.28 (m, 2H), 7.19 – 7.16 (m, 1H), 7.15 – 7.11 (m, 1H), 6.77 (d, *J* = 7.5 Hz, 1H), 3.90 (d, *J* = 13.7 Hz, 1H), 3.31 (d, *J* = 13.7 Hz, 1H), 3.25 (d, *J* = 15.2 Hz, 1H), 3.15 (d, *J* = 15.2 Hz, 1H), 3.06 – 2.95 (m, 3H), 2.91 – 2.81 (m, 2H), 2.66 (s, 3H), 2.51 – 2.46 (m, 1H), 2.46 (s, 3H), 2.32 – 2.27 (m, 2H), 1.99 – 1.90 (m, 2H), 1.46 – 1.30 (m, 6H), 0.90 (t, *J* = 7.1 Hz, 3H) ppm; **^{13}C{^{1}H} NMR** (151 MHz, CD_2Cl_2) δ = 199.2, 147.94, 147.86, 144.5, 143.8, 132.94, 132.89, 132.6, 128.59, 128.56, 127.9, 125.1, 124.7, 111.7, 103.1, 101.8, 100.2, 99.0, 61.9, 39.5, 38.4, 32.3, 30.4, 30.3, 29.6, 26.3, 26.2, 26.0, 22.9, 14.1, 14.0, 13.4 ppm; **IR** (ATR) ṽ = 2949 (m), 2927 (m), 2855 (w), 2053 (s), 1468 (w), 1450 (w), 1430 (w), 1369 (w), 909 (w), 787 (w), 758 (w), 731 (m), 497 (w), 463 (w) cm^{-1}; **HRMS** (ESI/QTOF) *m/z* = calcd. for $[C_{31}H_{35}CoI]^+$, $[M\text{-}CO\text{-}I^-]^+$: 593.1110, found: 593.1115; $[\boldsymbol{\alpha}]_{\mathbf{D}}^{22}$ = -432.3 (*c* = 0.05, $CHCl_3$).

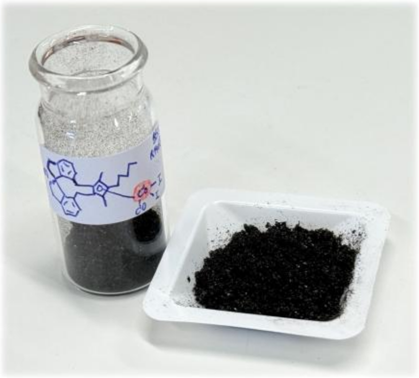

Figure S9. Gram-scale cobalt(III) complexation of **Cp^{V}32** furnishes 1.415 g of (*R*)-**Co13a** as a black solid.

5.3 Pentasubstituted Cp^V Rhodium Complexes

General Procedure 4 – Rhodium(III) Complexation of Chiral Cyclopentadienes.

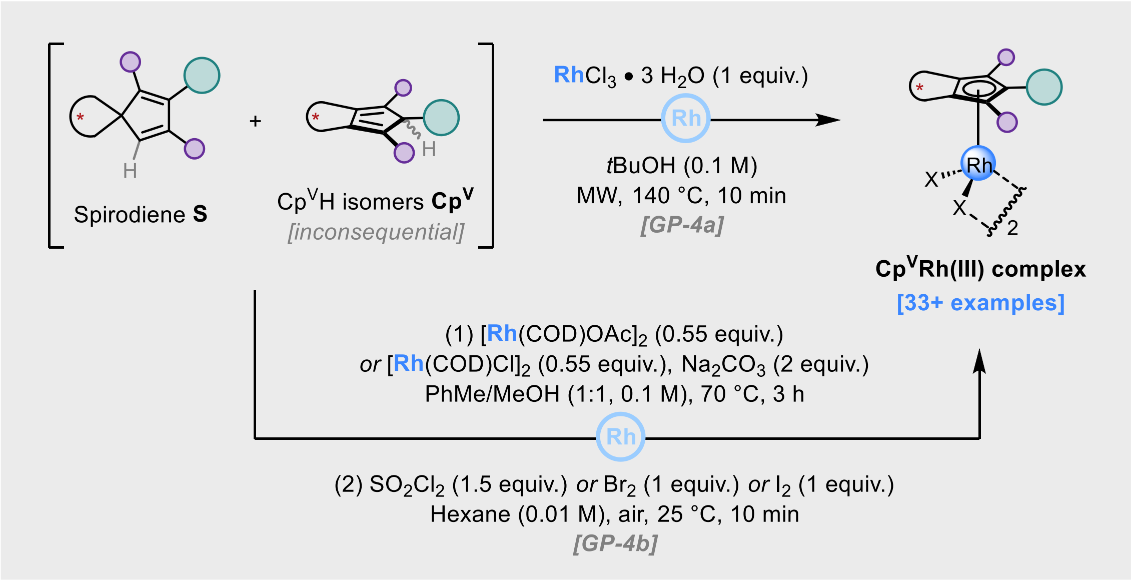

Scheme S18. Rhodium(III) complexation of chiral cyclopentadienes towards [Cp^{V}RhX$_2$]$_2$-type dimers.

General Procedure 4a: With RhCl$_3$·3 H$_2$O.

An oven-dried microwave vial was charged with a mixture of pentasubstituted chiral cyclopentadiene isomers **Cp^V** and spirodiene **S** (1 equiv. of Cp^{V}H isomers, as determined by qNMR) and rhodium trichloride trihydrate (1 equiv.), capped, and placed under an atmosphere of nitrogen by Schlenk technique. The solids were suspended in anhydrous *tert*-butanol (10 mL/mmol), and then shortly treated with a heat gun (*ca.* 10 seconds) to afford a homogeneous dark red solution. Next, the reaction mixture was stirred in a microwave reactor at 140 °C for 10 minutes. After cooling to room temperature (25 °C), the resulting orange-red suspension was first homogenized and transferred by addition of DCM, after which all volatiles were evaporated *in vacuo*. The residue was redissolved in DCM and filtered through a pad of celite (5 cm) with DCM as eluent. An orange-red solution eluted, whereas fine black solids were

retained on top. After evaporation of all volatiles *in vacuo*, the residue was purified by small-scale flash column chromatography on silica (wet loading with DCM, 5 cm column height, 0.5 cm diameter), eluting first with DCM (to remove impurities; usually a yellow-orange fraction) and then with ethyl acetate (to collect the retained complex; a dark red fraction). Removal of all volatiles *in vacuo* afforded the corresponding dimeric chiral [Cp^VRhCl_2]$_2$-type rhodium(III) complex as a dark red solid.

***General Procedure 4b*: With [Rh(COD)X]$_2$ (X = OAc, Cl) and Oxidant (SO_2Cl_2, Br_2, I_2).**

Step 1. An oven-dried microwave vial was charged with a mixture of pentasubstituted chiral cyclopentadiene isomers **Cp^V** and spirodiene **S** (1 equiv. of Cp^VH isomers, as determined by qNMR) and cyclooctadiene rhodium(I) acetate dimer (0.55 equiv.), capped, and placed under an atmosphere of nitrogen by Schlenk technique. Alternatively, cyclooctadiene rhodium(I) chloride dimer (0.55 equiv.), paired with sodium carbonate (2 equiv.), was used. After sequential addition of anhydrous toluene (5 mL/mmol) and anhydrous methanol (5 mL/mmol), the reaction mixture was stirred in a heating block at 70 °C for 3 hours. After cooling to room temperature (25 °C), all volatiles were evaporated *in vacuo*. The residue was redissolved in toluene, and the resulting suspension was passed through a pad of silica gel (3 cm) with toluene as eluent to give a homogeneous light-yellow solution. After removal of all volatiles *in vacuo*, the residue was redissolved in hexane (*or* Et_2O, if required for solubility, 50 mL/mmol) and immediately subjected to the oxidation step. Alternatively, if desired, chromatographic purification on silica allowed isolation of the Cp^VRh(COD)-type rhodium(I) complex as well as unreacted spirodiene **S**.

Step 2. At room temperature (25 °C) and under air, a solution of oxidant, i.e. sulfuryl chloride (1.5 equiv.) *or* bromine (1 equiv.) *or* iodine (1 equiv.), in hexane (50 mL/mmol) was added to the hexane solution of crude Cp^VRh(COD) complex under vigorous stirring, which resulted in an immediate color change and the precipitation of a fine orange-red (with SO_2Cl_2) *or* brown-red (with Br_2) *or* black-purple (with I_2) powder. After 10 minutes, the suspension was filtered on a glass sinter funnel. The filter cake was washed with pentane and dried *in vacuo*, affording the corresponding dimeric chiral [Cp^VRhX_2]$_2$-type rhodium(III) complex (X = Cl, Br, I). Usually, no purification through a pad of silica gel was required.

Note 1: For small-scale complexations, the **Cp^V/S** mixture foam could be transferred to the microwave vial as a THF solution, which was then evaporated *in vacuo* before adding the rhodium precursor.

Note 2: The preferred solvent for the NMR characterization of most [Cp^VRhX_2]$_2$-type complexes was d_6-DMSO. It breaks up the dimers by forming monomeric adducts, resulting in more resolved spectra with substantially sharper signals, contrary to the often broad and unclear signals in e.g. CD_2Cl_2 (**Figure S10**).

Note 3: In the $^{13}C\{^1H\}$ NMR spectra of multiple **Rh** complexes, not all Cp^V-ring carbon signals (usually seen as low-intensity doublets due to coupling with ^{103}Rh) were well resolved, yet their presence could be easily confirmed *via* the HMBC spectrum.

Rhodium complex (R_a)-**Rh1a**

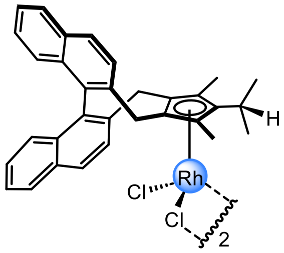

According to *General Procedure 4a*, starting from an isolated mixture of Cp^{V}H isomers **Cp^{V}1** and spirodiene **S1** (56:44 ratio, 195 µmol of Cp^{V}H), chiral Rh(III)-complex **Rh1a** (173 mg, 147 µmol of monomer, 75% yield) was obtained as a dark red solid. When *General Procedure 4b* was followed, using $[Rh(COD)OAc]_2$ as precursor and SO_2Cl_2 as oxidant, a lower yield of **Rh1a** (44% yield over 2 steps) was obtained.

^{1}H NMR (400 MHz, d_6-DMSO) δ = 8.08 – 7.96 (m, 4H), 7.78 (d, *J* = 8.6 Hz, 1H), 7.56 (d, *J* = 8.4 Hz, 1H), 7.52 – 7.43 (m, 2H), 7.33 – 7.28 (m, 1H), 7.26 – 7.21 (m, 1H), 7.00 (d, *J* = 8.5 Hz, 1H), 6.86 (d, *J* = 8.5 Hz, 1H), 3.60 – 3.51 (m, 2H), 3.29 (d, *J* = 16.2 Hz, 1H), 2.67 (hept, *J* = 7.0 Hz, 1H), 2.49 (d, *J* = 15.8 Hz, 1H), 1.90 (s, 3H), 1.82 (s, 3H), 1.29 (d, *J* = 7.0 Hz, 3H), 1.17 (d, *J* = 7.1 Hz, 3H) ppm; **^{13}C{^{1}H} NMR** (101 MHz, d_6-DMSO) δ = 135.1, 134.7, 133.6, 132.4, 132.3, 131.6, 131.2, 131.0, 129.3, 128.3, 128.2, 127.9, 127.2, 126.8, 126.2, 126.0, 125.9, 125.8, 125.4, 105.8 (d, *J* = 6.9 Hz), 105.1 (d, *J* = 7.7 Hz), 103.9 (d, *J* = 7.1 Hz), 95.6 (d, *J* = 7.3 Hz), 92.0 (d, *J* = 8.2 Hz), 29.8, 27.5, 25.1, 20.3, 19.9, 9.4, 8.6 ppm; **IR** (ATR) ṽ = 3049 (w), 2965 (w), 2927 (w), 1594 (w), 1508 (w), 1460 (w), 1365 (w), 1334 (w), 1265 (w), 1059 (w), 1025 (w), 866 (w), 813 (s), 775 (w), 752 (s), 733 (s), 703 (m), 680 (w), 566 (w), 461 (w) cm^{-1}; **HRMS** (ESI/QTOF) *m/z* = calcd. for $[C_{32}H_{29}ClRh]^+$, $[(M/2)-Cl^-]^+$: 551.1007, found: 551.1014; $[\alpha]_D^{26}$ = -33.3 (*c* = 0.06, $CHCl_3$).

Rhodium complex (R_a)-**Rh1b**

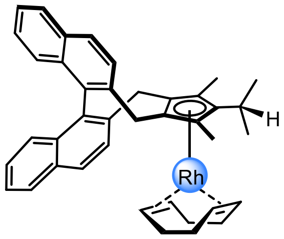

According to the first step of *General Procedure 4b*, starting from an isolated mixture of Cp^{V}H isomers **Cp^{V}1** and spirodiene **S1** (56:44 ratio, 74 µmol of Cp^{V}H) and using $[Rh(COD)Cl]_2$ as precursor, chiral Rh(I)-complex **Rh1b** (23 mg, 37 µmol, 50% yield) was obtained as a light-yellow solid. Purification was performed by Prep. TLC on silica (pentane/EtOAc = 50:1), which allowed isolation of unreacted spirodiene **S1** as well.

^{1}H NMR (400 MHz, CD_2Cl_2) δ = 8.03 (d, *J* = 8.4 Hz, 1H), 7.96 (d, *J* = 8.3 Hz, 1H), 7.91 (d, *J* = 8.3 Hz, 2H), 7.85 (d, *J* = 8.4 Hz, 1H), 7.45 – 7.39 (m, 2H), 7.38 (d, *J* = 8.4 Hz, 1H), 7.28 – 7.19 (m, 3H), 7.10 (d, *J* = 8.4 Hz, 1H), 3.55 (d, *J* = 14.5 Hz, 1H), 3.23 (d, *J* = 13.8 Hz, 1H), 3.19 – 3.12 (m, 2H), 2.99 (d, *J* = 14.6 Hz, 1H), 2.81 (d, *J* = 13.9 Hz, 1H), 2.77 – 2.70 (m, 3H), 2.31 – 2.20 (m, 2H), 2.06 – 1.94 (m, 2H), 1.92 (s, 3H), 1.89 – 1.79 (m, 4H), 1.50 – 1.47 (m, 6H), 1.27 (d, *J* = 7.1 Hz, 3H) ppm; **^{13}C{^{1}H} NMR** (101 MHz, CD_2Cl_2) δ = 137.6, 137.3, 136.2, 135.1, 132.7, 132.6, 132.45, 132.37, 130.0, 128.9, 128.8, 128.42, 128.39, 127.5, 127.2, 127.1, 126.5, 126.2, 125.42, 125.36, 109.8 (d, *J* = 4.4 Hz), 102.3 (d, *J* = 4.7 Hz), 98.1 (d, *J* = 4.0 Hz), 95.4 (d, *J* = 3.9 Hz), 93.1 (d, *J* = 4.4 Hz), 70.0 (d, *J* = 14.1 Hz), 69.2 (d, *J* = 14.1 Hz), 33.2, 32.2, 32.0, 29.2, 26.5, 24.8, 23.8, 10.3, 8.9 ppm; **IR** (ATR) ṽ = 2961 (m), 2926 (m), 2871 (w), 1648 (w), 1619 (w), 1595 (w), 1508 (w), 1445 (w), 1361 (w), 1027 (w), 909 (m), 865 (w), 814 (m), 753 (m), 732 (s) cm^{-1}; **HRMS** (ESI/QTOF) *m/z* = calcd. for $[C_{40}H_{41}Rh]^+$, $[M]^+$: 624.2258, found: 624.2278; **R_f** (pentane/EtOAc, 50:1) = 0.24; $[\alpha]_D^{26}$ = -73.3 (*c* = 0.05, $CHCl_3$).

Rhodium complex (R_a)-**Rh1c**

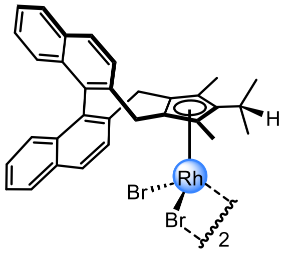

According to *General Procedure 4b*, starting from an isolated mixture of Cp^VH isomers **Cp^V1** and spirodiene **S1** (56:44 ratio, 258 µmol of Cp^VH) and using $[Rh(COD)Cl]_2$ as precursor and Br_2 as oxidant, chiral Rh(III)-complex **Rh1c** (47 mg, 69 µmol of monomer, 27% yield over 2 steps) was obtained as a brown-red solid.

^{1}H NMR (800 MHz, d_6-DMSO) δ = 8.06 (d, *J* = 8.6 Hz, 1H), 8.04 – 8.02 (m, 1H), 8.01 – 7.99 (m, 1H), 7.98 (d, *J* = 8.6 Hz, 1H), 7.80 (d, *J* = 8.5 Hz, 1H), 7.56 (d, *J* = 8.6 Hz, 1H), 7.51 – 7.49 (m, 1H), 7.47 – 7.44 (m, 1H), 7.33 – 7.30 (m, 1H), 7.26 – 7.23 (m, 1H), 7.01 – 6.98 (m, 1H), 6.86 – 6.84 (m, 1H), 3.62 (d, *J* = 16.6 Hz, 1H), 3.58 (d, *J* = 14.1 Hz, 1H), 3.50 (d, *J* = 14.2 Hz, 1H), 2.78 (hept, *J* = 7.1 Hz, 1H), 2.59 (d, *J* = 16.6 Hz, 1H), 2.00 (s, 3H), 1.90 (s, 3H), 1.32 (d, *J* = 7.1 Hz, 3H), 1.15 (d, *J* = 7.2 Hz, 3H) ppm; **^{13}C{^{1}H} NMR** (151 MHz, d_6-DMSO) δ = 135.2, 134.6, 134.1, 132.4, 132.28, 132.25, 131.6, 131.2, 131.1, 129.4, 128.3, 128.2, 127.9, 127.1, 126.8, 126.2, 126.0, 125.9, 125.8, 125.4, 107.3 (d, *J* = 6.7 Hz), 106.3 (d, *J* = 6.5 Hz), 103.8 (d, *J* = 6.6 Hz), 95.1 (d, *J* = 6.8 Hz), 93.6 (d, *J* = 7.6 Hz), 30.0, 28.2, 25.5, 20.5, 20.3, 9.9, 9.0 ppm; **IR** (ATR) ṽ = 3052 (w), 2966 (w), 2929 (w), 2871 (w), 1508 (w), 1460 (w), 1366 (w), 1025 (w), 908 (m), 866 (w), 814 (m), 775 (w), 751 (m), 728 (s), 705 (w), 679 (w), 645 (w) cm^{-1}; **HRMS** (ESI/QTOF) *m/z* = calcd. for $[C_{32}H_{29}BrRh]^+$, $[(M/2)-Br^-]^+$: 595.0502, found: 595.0507; $[\alpha]_D^{22}$ = -73.6 (*c* = 0.05, $CHCl_3$).

Rhodium complex (R_a)-**Rh1d**

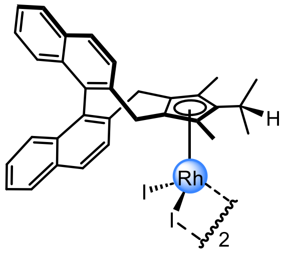

According to *General Procedure 4b*, starting from an isolated mixture of Cp^VH isomers **Cp^V1** and spirodiene **S1** (56:44 ratio, 217 µmol of Cp^VH) and using $[Rh(COD)Cl]_2$ as precursor and I_2 as oxidant, chiral Rh(III)-complex **Rh1d** (61 mg, 80 µmol of monomer, 37% yield over 2 steps) was obtained as a purple-black solid.

^{1}H NMR (600 MHz, d_6-DMSO) δ = 8.06 (d, *J* = 8.5 Hz, 1H), 8.02 (d, *J* = 8.2 Hz, 1H), 8.01 – 7.98 (m, 2H), 7.86 (d, *J* = 8.5 Hz, 1H), 7.55 (d, *J* = 8.5 Hz, 1H), 7.52 – 7.48 (m, 1H), 7.48 – 7.44 (m, 1H), 7.33 – 7.29 (m, 1H), 7.26 – 7.22 (m, 1H), 6.99 (d, *J* = 8.5 Hz, 1H), 6.85 (d, *J* = 8.5 Hz, 1H), 3.84 (d, *J* = 14.0 Hz, 1H), 3.74 (d, *J* = 16.6 Hz, 1H), 3.59 (d, *J* = 13.9 Hz, 1H), 3.02 (hept, *J* = 7.1 Hz, 1H), 2.78 (d, *J* = 16.5 Hz, 1H), 2.18 (s, 3H), 2.04 (s, 3H), 1.34 (d, *J* = 7.0 Hz, 3H), 1.12 (d, *J* = 7.2 Hz, 3H) ppm; **^{13}C{^{1}H} NMR** (151 MHz, d_6-DMSO) δ = 135.4, 135.0, 134.5, 132.4, 132.32, 132.27, 131.6, 131.3, 131.2, 129.5, 128.4, 128.3, 128.2, 128.0, 126.9, 126.8, 126.2, 126.1, 125.9, 125.8, 125.5, 109.7 (d, *J* = 5.4 Hz), 107.2 (d, *J* = 5.3 Hz), 104.1 (d, *J* = 5.1 Hz), 96.4 (d, *J* = 6.5 Hz), 94.7 (d, *J* = 6.4 Hz), 30.3, 29.7, 26.4, 21.4, 21.0, 11.1, 10.1 ppm; **IR** (ATR) ṽ = 3052 (w), 2968 (w), 2928 (w), 2870 (w), 1593 (w), 1507 (w), 1454 (w), 1369 (w), 1336 (w), 1056 (w), 1023 (w), 908 (m), 866 (w), 813 (m), 774 (w), 751 (m), 730 (s), 704 (w) cm^{-1}; **HRMS** (ESI/QTOF) *m/z* = calcd. for $[C_{64}H_{58}I_3Rh_2]^+$, $[M-I^-]^+$: 1412.9778, found: 1412.9799; $[\alpha]_D^{22}$ = -368.3 (*c* = 0.06, $CHCl_3$).

Rhodium complex (R_a)-**Rh2**

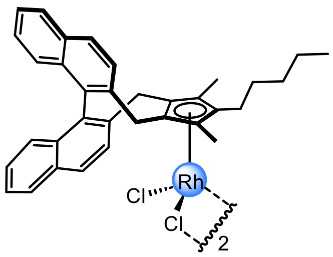

According to *General Procedure 4a*, starting from an isolated mixture of Cp^VH isomers **Cp^{V}2** and spirodiene **S2** (70:30 ratio, 160 µmol of Cp^VH), chiral Rh(III)-complex **Rh2** (61 mg, 99 µmol of monomer, 62% yield) was obtained as a dark red solid.

^{1}H NMR (400 MHz, d_6-DMSO) δ = 8.06 (d, *J* = 8.5 Hz, 1H), 8.04 – 7.97 (m, 3H), 7.88 (d, *J* = 8.5 Hz, 1H), 7.57 (d, *J* = 8.5 Hz, 1H), 7.53 – 7.44 (m, 2H), 7.35 – 7.30 (m, 1H), 7.28 – 7.22 (m, 1H), 7.04 (d, *J* = 8.5 Hz, 1H), 6.87 (d, *J* = 8.5 Hz, 1H), 3.55 – 3.48 (m, 2H), 3.20 (d, *J* = 14.2 Hz, 1H), 2.52 (d, *J* = 15.6 Hz, 1H), 2.30 – 2.20 (m, 1H), 2.16 – 2.06 (m, 1H), 1.80 (s, 3H), 1.73 (s, 3H), 1.41 – 1.34 (m, 2H), 1.33 – 1.23 (m, 4H), 0.85 (t, *J* = 6.9 Hz, 3H) ppm; **^{13}C{^{1}H} NMR** (101 MHz, d_6-DMSO) δ = 135.1, 134.7, 133.4, 132.4, 132.3, 131.5, 131.1, 130.9, 129.3, 128.3, 128.2, 128.0, 127.3, 126.8, 126.2, 126.0, 125.9, 125.8, 125.5, 107.7 (d, *J* = 6.8 Hz), 103.3 (d, *J* = 7.6 Hz), 101.3 (d, *J* = 7.1 Hz), 93.7 (d, *J* = 7.6 Hz), 91.7 (d, *J* = 7.3 Hz), 31.2, 29.8, 27.8, 26.9, 23.4, 21.8, 13.8, 8.23, 8.18 ppm; **IR** (ATR) ṽ = 2955 (w), 2926 (w), 2858 (w), 1508 (w), 1459 (w), 1377 (w), 1025 (w), 908 (m), 867 (w), 825 (w), 811 (m), 749 (m), 728 (s), 645 (w) cm^{-1}; **HRMS** (ESI/QTOF) *m/z* = calcd. for $[C_{34}H_{33}ClRh]^+$, $[(M/2)-Cl^-]^+$: 579.1320, found: 579.1333; $[\alpha]_D^{22}$ = +38.1 (*c* = 0.17, $CHCl_3$).

Rhodium complex (R_a)-**Rh3**

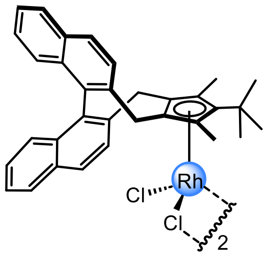

According to *General Procedure 4a*, starting from an isolated mixture of Cp^VH isomers **Cp^{V}5** and spirodiene **S5** (51:49 ratio, 212 µmol of Cp^VH), chiral Rh(III)-complex **Rh3** (69 mg, 115 µmol of monomer, 54% yield) was obtained as a dark red solid.

^{1}H NMR (500 MHz, d_6-DMSO) δ = 8.06 (d, *J* = 8.4 Hz, 1H), 8.04 – 7.96 (m, 3H), 7.79 (d, *J* = 8.5 Hz, 1H), 7.54 – 7.48 (m, 2H), 7.48 – 7.44 (m, 1H), 7.34 – 7.30 (m, 1H), 7.26 – 7.22 (m, 1H), 7.05 – 7.01 (m, 1H), 6.88 – 6.85 (m, 1H), 3.61 (d, *J* = 14.1 Hz, 1H), 3.56 (d, *J* = 16.3 Hz, 1H), 3.30 (d, *J* = 14.0 Hz, 1H), 2.48 (d, *J* = 16.2 Hz, 1H), 2.10 (s, 3H), 1.93 (s, 3H), 1.41 (s, 9H) ppm; **^{13}C{^{1}H} NMR** (126 MHz, d_6-DMSO) δ = 135.1, 134.7, 133.6, 132.4, 132.35, 132.27, 131.5, 131.1, 131.0, 129.3, 128.3, 128.1, 127.8, 127.2, 126.8, 126.3, 126.0, 125.9, 125.8, 125.4, 107.60 (d, *J* = 6.9 Hz), 107.57 (d, *J* = 7.4 Hz), 104.7 (d, *J* = 7.2 Hz), 94.3 (d, *J* = 7.2 Hz), 91.6 (d, *J* = 8.4 Hz), 34.0, 30.3, 30.0, 27.0, 12.6, 11.6 ppm; **IR** (ATR) ṽ = 3052 (w), 2961 (w), 2923 (w), 1508 (w), 1459 (w), 1428 (w), 1388 (w), 1367 (w), 1225 (w), 1025 (w), 907 (m), 867 (w), 814 (m), 752 (m), 726 (s), 699 (w), 685 (w), 645 (w) cm^{-1}; **HRMS** (ESI/QTOF) *m/z* = calcd. for $[C_{33}H_{31}ClRh]^+$, $[(M/2)-Cl^-]^+$: 565.1164, found: 565.1171; $[\alpha]_D^{22}$ = +36.5 (*c* = 0.13, $CHCl_3$).

Rhodium complex (R_a)-**Rh4**

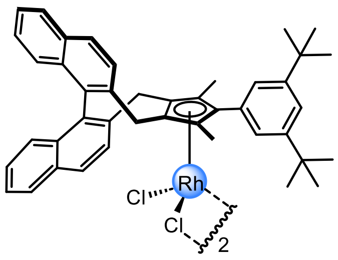

According to *General Procedure 4a*, starting from an isolated mixture of Cp^VH isomers **Cp^V6** and spirodiene **S6** (77:23 ratio, 92 µmol of Cp^VH), chiral Rh(III)-complex **Rh4** (50 mg, 68 µmol of monomer, 74% yield) was obtained as a dark red solid.

^{1}H NMR (600 MHz, d_6-DMSO) δ = 8.11 – 8.09 (m, 1H), 8.06 – 8.00 (m, 3H), 7.76 (d, *J* = 8.6 Hz, 1H), 7.68 (d, *J* = 8.5 Hz, 1H), 7.57 – 7.55 (m, 2H), 7.52 – 7.49 (m, 1H), 7.49 – 7.46 (m, 1H), 7.42 – 7.40 (m, 1H), 7.34 – 7.30 (m, 1H), 7.28 – 7.24 (m, 1H), 7.01 – 6.98 (m, 1H), 6.89 – 6.85 (m, 1H), 3.67 (d, *J* = 16.4 Hz, 1H), 3.63 (d, *J* = 13.9 Hz, 1H), 3.42 (d, *J* = 14.0 Hz, 1H), 2.69 (d, *J* = 16.3 Hz, 1H), 1.90 (s, 3H), 1.75 (s, 3H), 1.29 (s, 18H) ppm; **^{13}C{^{1}H} NMR** (151 MHz, d_6-DMSO) δ = 150.0, 135.1, 134.7, 133.7, 132.4, 132.3, 132.1, 131.7, 131.3, 129.3, 128.3, 128.1, 127.7, 127.6, 127.3, 126.7, 126.2, 126.0, 125.9, 125.4, 125.3, 121.8, 105.8 (d, *J* = 5.9 Hz), 103.6 (d, *J* = 5.0 Hz), 99.7 (d, *J* = 5.0 Hz), 99.2 (d, *J* = 6.5 Hz), 92.3 (d, *J* = 7.2 Hz), 34.8, 31.2, 30.4, 27.7, 9.9, 9.6 ppm; **IR** (ATR) ṽ = 3057 (w), 2962 (s), 2904 (w), 2867 (w), 1595 (w), 1508 (w), 1475 (w), 1441 (w), 1394 (w), 1363 (w), 1248 (w), 1026 (w), 910 (m), 881 (w), 868 (w), 850 (w), 814 (m), 754 (s), 732 (s), 687 (w) cm^{-1}; **HRMS** (nanochip-ESI/LTQ-Orbitrap) *m/z* = calcd. for $[C_{43}H_{43}ClRh]^+$, $[(M/2)-Cl^-]^+$: 697.2103, found: 697.2089; $[\alpha]_D^{22}$ = +86.7 (*c* = 0.05, $CHCl_3$).

Rhodium complex (R_a)-**Rh5**

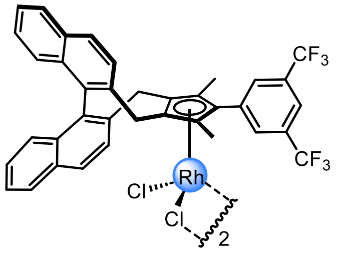

According to *General Procedure 4a*, starting from an isolated mixture of Cp^VH isomers **Cp^V7** and spirodiene **S7** (87:13 ratio, 96 µmol of Cp^VH), chiral Rh(III)-complex **Rh5** (57 mg, 75 µmol of monomer, 78% yield) was obtained as a dark red solid.

^{1}H NMR (600 MHz, d_6-DMSO) δ = 8.47 (s, 2H), 8.21 (s, 1H), 8.12 (d, *J* = 8.5 Hz, 1H), 8.07 – 8.04 (m, 1H), 8.04 – 8.01 (m, 2H), 7.72 (d, *J* = 8.5 Hz, 1H), 7.67 (d, *J* = 8.5 Hz, 1H), 7.53 – 7.46 (m, 2H), 7.34 – 7.30 (m, 1H), 7.29 – 7.25 (m, 1H), 6.99 (d, *J* = 8.5 Hz, 1H), 6.88 (d, *J* = 8.5 Hz, 1H), 3.77 (d, *J* = 16.5 Hz, 1H), 3.68 (d, *J* = 13.9 Hz, 1H), 3.38 (d, *J* = 13.9 Hz, 1H), 2.69 (d, *J* = 16.5 Hz, 1H), 1.90 (s, 3H), 1.75 (s, 3H) ppm; **^{13}C{^{1}H} NMR** (151 MHz, d_6-DMSO) δ = 135.1, 134.6, 133.4, 132.45, 132.38, 131.8, 131.7, 131.6, 131.3, 131.1, 130.2 (q, *J* = 33.1 Hz), 129.3, 128.3, 128.2, 127.8, 127.7, 126.8, 126.13, 126.10, 125.94, 125.89, 125.5, 123.1 (q, *J* = 273.1 Hz), 122.7 – 122.6 (m), 107.6 (d, *J* = 5.6 Hz), 103.8 (d, *J* = 5.8 Hz), 101.6 (d, *J* = 5.0 Hz), 93.4 (d, *J* = 6.6 Hz), 93.3 (d, *J* = 6.9 Hz), 30.3, 27.5, 9.7, 9.1 ppm; **^{19}F{^{1}H} NMR** (376 MHz, d_6-DMSO) δ = -56.45 ppm; **IR** (ATR) ṽ = 1344 (m), 1280 (s), 1183 (m), 1139 (m), 1110 (w), 903 (w), 815 (w), 757 (w), 676 (w) cm^{-1}; **HRMS** (ESI/QTOF) *m/z* = calcd. for $[C_{37}H_{25}ClF_6Rh]^+$, $[(M/2)-Cl^-]^+$: 721.0598, found: 721.0615; $[\alpha]_D^{26}$ = -4.2 (*c* = 0.08, $CHCl_3$).

Rhodium complex (R_a)-**Rh6**

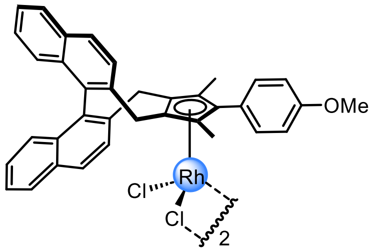

According to *General Procedure 4a*, starting from an isolated mixture of Cp^VH isomers **Cp^V8** and spirodiene **S8** (72:28 ratio, 130 µmol of Cp^VH), chiral Rh(III)-complex **Rh6** (59 mg, 91 µmol of monomer, 70% yield) was obtained as a dark red solid.

^{1}H NMR (600 MHz, d_6-DMSO) δ = 8.11 – 8.07 (m, 1H), 8.06 – 7.99 (m, 3H), 7.75 (d, *J* = 8.5 Hz, 1H), 7.63 (d, *J* = 8.5 Hz, 1H), 7.62 – 7.59 (m, 2H), 7.52 – 7.49 (m, 1H), 7.49 – 7.45 (m, 1H), 7.33 – 7.30 (m, 1H), 7.27 – 7.24 (m, 1H), 7.01 – 6.97 (m, 3H), 6.89 – 6.86 (m, 1H), 3.79 (s, 3H), 3.65 (d, *J* = 16.5 Hz, 1H), 3.62 (d, *J* = 14.0 Hz, 1H), 3.39 (d, *J* = 14.0 Hz, 1H), 2.66 (d, *J* = 16.4 Hz, 1H), 1.89 (s, 3H), 1.73 (s, 3H) ppm; **^{13}C{^{1}H} NMR** (151 MHz, d_6-DMSO) δ = 159.6, 135.1, 134.7, 133.7, 132.4, 132.3, 132.1, 132.0, 131.6, 131.3, 131.2, 129.3, 128.3, 128.2, 127.7, 127.5, 126.7, 126.2, 126.0, 125.89, 125.85, 125.5, 120.0, 113.7, 105.4 (d, *J* = 6.6 Hz), 103.9 (d, *J* = 6.1 Hz), 98.8 (d, *J* = 7.3 Hz), 98.6 (d, *J* = 6.2 Hz), 92.2 (d, *J* = 7.7 Hz), 55.2, 30.4, 27.6, 9.9, 9.5 ppm; **IR** (ATR) ṽ = 3054 (w), 2928 (w), 1608 (m), 1521 (m), 1458 (m), 1441 (w), 1293 (w), 1252 (s), 1178 (m), 1031 (m), 989 (w), 910 (m), 867 (w), 834 (m), 815 (w), 754 (m), 730 (s), 423 (w) cm^{-1}; **HRMS** (nanochip-ESI/LTQ-Orbitrap) *m/z* = calcd. for $[C_{36}H_{29}ClORh]^+$, $[(M/2)-Cl^-]^+$: 615.0956, found: 615.0950; $[\alpha]_D^{22}$ = +72.2 (*c* = 0.06, $CHCl_3$).

Rhodium complex (R_a)-**Rh7**

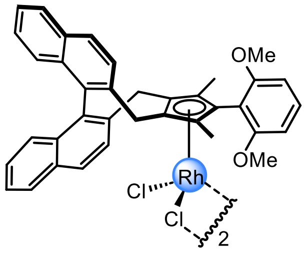

According to *General Procedure 4a*, starting from an isolated mixture of Cp^VH isomers **Cp^V9** and spirodiene **S9** (58:42 ratio, 143 µmol of Cp^VH), chiral Rh(III)-complex **Rh7** (91 mg, 133 µmol of monomer, 93% yield) was obtained as a dark red solid. Notably, one of the Cp-ring's ^{13}C NMR signals was not resolved.

^{1}H NMR (400 MHz, d_6-DMSO) δ = 8.12 (d, *J* = 8.5 Hz, 1H), 8.05 (d, *J* = 8.2 Hz, 1H), 8.03 – 7.97 (m, 2H), 7.74 (d, *J* = 8.5 Hz, 1H), 7.64 (d, *J* = 8.5 Hz, 1H), 7.54 – 7.43 (m, 2H), 7.41 (t, *J* = 8.4 Hz, 1H), 7.36 – 7.29 (m, 1H), 7.29 – 7.21 (m, 1H), 7.02 (d, *J* = 8.5 Hz, 1H), 6.88 (d, *J* = 8.5 Hz, 1H), 6.71 – 6.64 (m, 2H), 3.79 (s, 3H), 3.73 (s, 3H), 3.57 (d, *J* = 13.9 Hz, 1H), 3.50 (d, *J* = 16.2 Hz, 1H), 3.41 (d, *J* = 13.8 Hz, 1H), 2.55 (d, *J* = 16.1 Hz, 1H), 1.68 (s, 3H), 1.46 (s, 3H) ppm; **^{13}C{^{1}H} NMR** (101 MHz, d_6-DMSO) δ = 158.0, 157.4, 134.9, 134.8, 133.8, 132.5, 132.4, 132.3, 131.7, 131.34, 131.27, 131.2, 129.5, 128.3, 128.2, 127.2, 126.8, 126.2, 126.0, 125.9, 125.7, 125.4, 110.1 (d, *J* = 6.1 Hz), 104.8, 104.2, 103.6 (d, *J* = 7.8 Hz), 103.1, 92.3 (d, *J* = 8.1 Hz), 90.0 (d, *J* = 8.4 Hz), 55.8, 55.6, 30.5, 27.6, 10.8, 10.2 ppm; **IR** (ATR) ṽ = 2931 (w), 1587 (w), 1474 (m), 1430 (m), 1378 (w), 1295 (w), 1254 (m), 1111 (s), 1026 (w), 987 (w), 815 (w), 783 (w), 775 (w), 754 (w), 733 (m), 699 (w) cm^{-1}; **HRMS** (APCI/QTOF) *m/z* = calcd. for $[C_{37}H_{31}ClO_2Rh]^+$, $[(M/2)-Cl^-]^+$: 645.1062, found: 645.1047; $[\alpha]_D^{26}$ = -98.8 (*c* = 0.08, $CHCl_3$).

Rhodium complex (R_a)-**Rh8a**

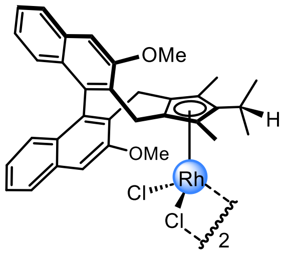

According to *General Procedure 4a*, starting from an isolated mixture of Cp^{V}H isomers **Cp^{V}10** and spirodiene **S10** (45:55 ratio, 35 µmol of Cp^{V}H), chiral Rh(III)-complex **Rh8a** (15 mg, 23 µmol of monomer, 65% yield) was obtained as a dark red solid. When *General Procedure 4b* was followed, using $[Rh(COD)OAc]_2$ as precursor and SO_2Cl_2 as oxidant, a lower yield of **Rh8a** (42% yield over 2 steps) was obtained.

^{1}H NMR (400 MHz, d_6-DMSO) δ = 7.93 – 7.87 (m, 2H), 7.52 (s, 1H), 7.46 – 7.41 (m, 1H), 7.41 – 7.36 (m, 2H), 7.15 – 7.10 (m, 1H), 7.08 – 7.03 (m, 1H), 6.87 (d, *J* = 8.5 Hz, 1H), 6.79 (d, *J* = 8.4 Hz, 1H), 4.01 – 3.96 (m, 4H), 3.92 (s, 3H), 3.64 (d, *J* = 13.4 Hz, 1H), 3.08 (d, *J* = 13.5 Hz, 1H), 2.61 (hept, *J* = 7.0 Hz, 1H), 2.09 (d, *J* = 15.8 Hz, 1H), 1.92 (s, 3H), 1.81 (s, 3H), 1.23 (d, *J* = 6.9 Hz, 3H), 1.20 (d, *J* = 7.1 Hz, 3H) ppm; **^{13}C{^{1}H} NMR** ^{13}C NMR (101 MHz, d_6-DMSO) δ = 157.0, 154.8, 136.6, 136.5, 133.7, 133.5, 127.1, 126.9, 126.6, 126.4, 126.3, 126.2, 125.9, 125.7, 124.4, 124.1, 123.3, 108.2 (d, *J* = 6.8 Hz), 106.2, 105.6, 101.8 (d, *J* = 7.2 Hz), 99.8 (d, *J* = 7.0 Hz), 97.9 (d, *J* = 8.2 Hz), 93.6 (d, *J* = 7.9 Hz), 55.6, 55.5, 24.5, 22.2, 21.3, 20.7, 20.1, 9.7, 9.3 ppm; **IR** (ATR) ṽ = 2962 (w), 2930 (w), 1619 (w), 1596 (m), 1450 (s), 1422 (m), 1387 (w), 1329 (w), 1295 (w), 1236 (m), 1196 (w), 1174 (w), 1163 (w), 1150 (w), 1109 (s), 1020 (m), 864 (w), 831 (w), 746 (m) cm^{-1}; **HRMS** (ESI/QTOF) *m/z* = calcd. for $[C_{34}H_{33}ClO_2Rh]^+$, $[(M/2)-Cl^-]^+$: 611.1219, found: 611.1227; $[\alpha]_D^{26}$ = -66.7 (*c* = 0.04, $CHCl_3$).

Rhodium complex (R_a)-**Rh8b**

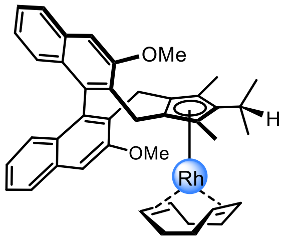

According to the first step of *General Procedure 4b*, starting from an isolated mixture of Cp^{V}H isomers **Cp^{V}10** and spirodiene **S10** (45:55 ratio, 60 µmol of Cp^{V}H) and using $[Rh(COD)OAc]_2$ as precursor, chiral Rh(I)-complex **Rh8b** (23 mg, 33 µmol, 55% yield) was obtained as a light-yellow solid. Purification was performed by Prep. TLC on silica (pentane/EtOAc = 40:1).

^{1}H NMR (400 MHz, CD_2Cl_2) δ = 7.87 (d, *J* = 8.2 Hz, 1H), 7.83 – 7.79 (m, 1H), 7.41 – 7.36 (m, 2H), 7.36 – 7.32 (m, 1H), 7.27 (s, 1H), 7.09 – 7.03 (m, 2H), 6.99 – 6.93 (m, 2H), 4.16 (d, *J* = 14.5 Hz, 1H), 4.10 (s, 3H), 3.98 (s, 3H), 3.65 (d, *J* = 13.5 Hz, 1H), 3.29 – 3.20 (m, 2H), 2.80 – 2.72 (m, 4H), 2.59 (d, *J* = 14.5 Hz, 1H), 2.07 – 2.00 (m, 2H), 1.99 – 1.89 (m, 7H), 1.74 – 1.67 (m, 2H), 1.52 (s, 3H), 1.50 (d, *J* = 7.1 Hz, 3H), 1.28 (d, *J* = 7.1 Hz, 3H) ppm; **^{13}C{^{1}H} NMR** (101 MHz, CD_2Cl_2) δ = 156.3, 155.6, 138.1, 137.0, 133.8, 133.6, 129.6, 129.5, 127.91, 127.88, 127.2, 127.14, 127.08, 127.0, 125.90, 125.88, 123.9, 123.8, 109.2 (d, *J* = 4.5 Hz), 105.61, 105.59, 102.4 (d, *J* = 4.7 Hz), 96.4 (d, *J* = 3.8 Hz), 95.9 (d, *J* = 3.9 Hz), 93.5 (d, *J* = 4.2 Hz), 70.0 (d, *J* = 14.1 Hz), 69.1 (d, *J* = 14.1 Hz), 55.60, 55.55, 34.2, 31.5, 26.7, 24.8, 23.9, 23.2, 22.9, 9.9, 8.9 ppm; **IR** (ATR) ṽ = 2958 (w), 2924 (s), 2868 (m), 2822 (w), 1619 (w), 1596 (m), 1450 (s), 1423 (m), 1328 (w), 1294 (w), 1236 (m), 1220 (m), 1197 (m), 1161 (w), 1150 (w), 1108 (s), 1022 (w), 863 (w), 829 (w), 745 (m) cm^{-1}; **HRMS** (ESI/QTOF) *m/z* = calcd. for $[C_{42}H_{45}O_2Rh]^+$, $[M]^+$: 684.2469, found: 684.2483; **R$_f$** (pentane/EtOAc, 40:1) = 0.31; $[\alpha]_D^{22}$ = -183.3 (*c* = 0.05, $CHCl_3$).

Rhodium complex (R_a)-**Rh8c**

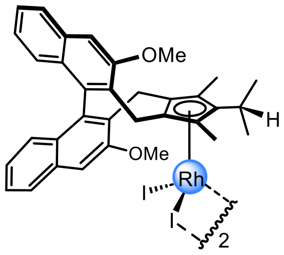

According to the second step of *General Procedure 4b*, starting from isolated Rh(I)-complex **Rh8b** and using I_2 as oxidant, chiral Rh(III)-complex **Rh8c** (18 mg, 22 µmol, 65% yield) was obtained as a dark brown solid. After filtration, the complex was washed with both pentane and Et_2O.

^{1}H NMR (400 MHz, d_6-DMSO) δ = 7.93 – 7.87 (m, 2H), 7.53 (s, 1H), 7.45 – 7.36 (m, 3H), 7.13 – 7.08 (m, 1H), 7.07 – 7.02 (m, 1H), 6.81 – 6.78 (m, 1H), 6.74 (d, *J* = 8.6 Hz, 1H), 4.13 (d, *J* = 16.3 Hz, 1H), 4.00 (s, 3H), 3.94 (s, 3H), 3.72 (d, *J* = 13.4 Hz, 1H), 3.60 (d, *J* = 13.3 Hz, 1H), 2.83 (hept, *J* = 6.8 Hz, 1H), 2.36 (d, *J* = 16.4 Hz, 1H), 2.21 (s, 3H), 2.20 (s, 3H), 1.30 (d, *J* = 7.0 Hz, 3H), 1.11 (d, *J* = 7.1 Hz, 3H) ppm; **^{13}C{^{1}H} NMR** (101 MHz, d_6-DMSO) δ = 156.7, 154.6, 137.1, 136.4, 133.7, 133.4, 127.1, 126.92, 126.91, 126.6, 126.40, 126.36, 126.1, 126.0, 125.8, 107.8 (d, *J* = 6.2 Hz), 106.3, 105.7, 104.4 (d, *J* = 6.9 Hz), 104.0 (d, *J* = 6.2 Hz), 99.8 (d, *J* = 6.8 Hz), 95.6 (d, *J* = 7.0 Hz), 55.4, 55.0, 25.4, 23.6, 22.6, 21.2, 20.9, 12.3, 11.1 ppm; **IR** (ATR) ṽ = 2960 (w), 2925 (m), 1619 (w), 1597 (m), 1452 (s), 1423 (m), 1390 (w), 1330 (w), 1293 (w), 1236 (m), 1197 (,m), 1176 (w), 1163 (w), 1150 (w), 1109 (s), 1020 (w), 910 (w), 864 (w), 831 (w), 746 (w), 731 (s) cm^{-1}; **HRMS** (ESI/QTOF) *m/z* = calcd. for $[C_{34}H_{33}IO_2Rh]^+$, $[(M/2)-I]^+$: 703.0575, found: 703.0583; $[\alpha]_D^{22}$ = -492.7 (*c* = 0.05, $CHCl_3$).

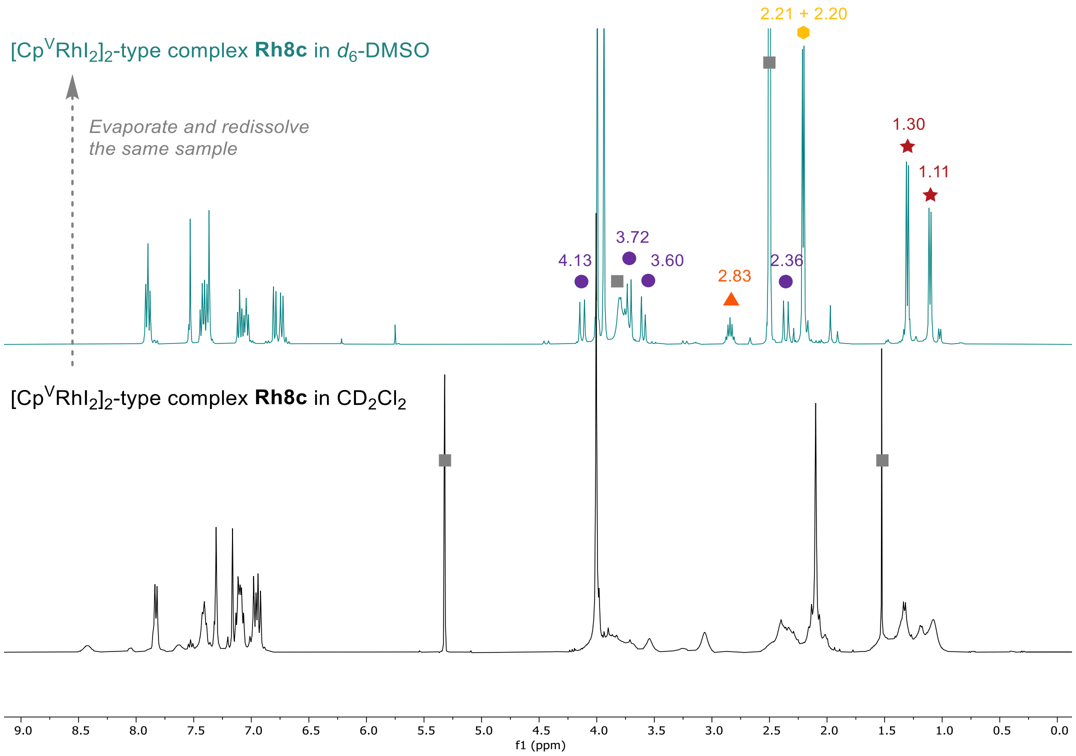

Figure S10. Comparison of the ^{1}H NMR signals (400 MHz) of dimeric $[Cp^VRhI_2]_2$-type complex (R_a)-**Rh8c** in different solvents. In CD_2Cl_2 (bottom spectrum), the signals were broad and unclear. In contrast, d_6-DMSO (top spectrum) provided a clear NMR with well resolved signals. Selected characteristic signals have been marked with their chemical shift value δ_H (in ppm): orange triangle (CH of *i*Pr group), purple circles (diastereotopic CH_2 groups), red stars (CH_3 of *i*Pr group), yellow hexagon (Cp-bound CH_3), and gray squares (residual solvent and water signals).

Rhodium complex (R_a)-**Rh9**

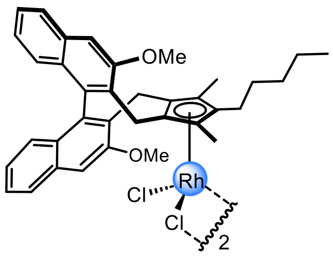

According to *General Procedure 4a*, starting from an isolated mixture of Cp^VH isomers **Cp^V11** and spirodiene **S11** (62:38 ratio, 22 µmol of Cp^VH), chiral Rh(III)-complex **Rh9** (24 mg, 17 µmol of monomer, 78% yield) was obtained as a dark red solid.

^{1}H NMR (400 MHz, d_6-DMSO) δ = 7.94 – 7.88 (m, 2H), 7.53 (s, 1H), 7.46 – 7.37 (m, 3H), 7.16 – 7.11 (m, 1H), 7.09 – 7.04 (m, 1H), 6.89 (d, *J* = 8.1 Hz, 1H), 6.80 (d, *J* = 8.5 Hz, 1H), 3.99 (s, 3H), 3.96 – 3.90 (m, 4H), 3.63 (d, *J* = 13.5 Hz, 1H), 3.05 (d, *J* = 13.5 Hz, 1H), 2.20 – 2.00 (m, 3H), 1.83 (s, 3H), 1.76 (s, 3H), 1.42 – 1.20 (m, 6H), 0.85 (t, *J* = 6.8 Hz, 3H) ppm; **^{13}C{^{1}H} NMR** (101 MHz, d_6-DMSO) δ = 156.8, 154.8, 136.6, 136.5, 133.7, 133.5, 127.1, 126.9, 126.6, 126.4, 126.3, 126.2, 125.88, 125.85, 125.7, 124.4, 124.2, 123.3, 106.9 (d, *J* = 7.5 Hz), 106.2, 105.6, 101.7 (d, *J* = 6.7 Hz), 101.6 (d, *J* = 6.6 Hz), 93.9 (d, *J* = 8.4 Hz), 92.2 (d, *J* = 8.0 Hz), 55.5, 55.4, 31.0, 26.9, 22.7, 22.1, 21.9, 21.5, 13.8, 9.1, 8.6 ppm; **IR** (ATR) ṽ = 2954 (w), 2926 (m), 2855 (w), 1619 (w), 1597 (m), 1452 (s), 1423 (m), 1388 (w), 1330 (w), 1295 (w), 1236 (m), 1197 (w), 1174 (w), 1162 (w), 1158 (w), 1109 (s), 1080 (w), 1021 (m), 863 (w), 830 (w), 746 (m) cm^{-1}; **HRMS** (APCI/QTOF) *m/z* = calcd. for $[C_{36}H_{37}ClO_2Rh]^+$, $[(M/2)-Cl^-]^+$: 639.1532, found: 639.1539; $[\alpha]_D^{26}$ = -20.8 (*c* = 0.04, $CHCl_3$).

Rhodium complex (R_a)-**Rh10**

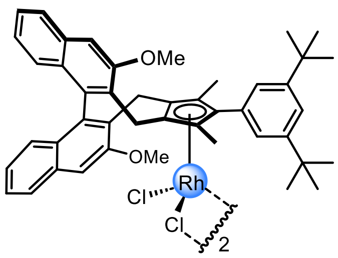

According to *General Procedure 4a*, starting from an isolated mixture of Cp^VH isomers **Cp^V13** and spirodiene **S13** (76:24 ratio, 70 µmol of Cp^VH), chiral Rh(III)-complex **Rh10** (30 mg, 38 µmol of monomer, 54% yield) was obtained as a dark red solid.

^{1}H NMR (400 MHz, d_6-DMSO) δ = 7.92 (t, *J* = 7.5 Hz, 2H), 7.70 – 7.60 (m, 2H), 7.55 (s, 1H), 7.47 – 7.43 (m, 1H), 7.43 – 7.38 (m, 3H), 7.17 – 7.12 (m, 1H), 7.10 – 7.04 (m, 1H), 6.90 (d, *J* = 8.5 Hz, 1H), 6.80 (d, *J* = 8.4 Hz, 1H), 4.03 (d, *J* = 16.0 Hz, 1H), 4.00 (s, 3H), 3.95 (s, 3H), 3.71 (d, *J* = 13.4 Hz, 1H), 3.24 (d, *J* = 13.4 Hz, 1H), 2.24 (d, *J* = 15.8 Hz, 1H), 1.86 (s, 3H), 1.78 (s, 3H), 1.29 (s, 18H) ppm; **^{13}C{^{1}H} NMR** (101 MHz, d_6-DMSO) δ = 157.2, 154.9, 150.2, 136.7, 136.4, 133.7, 133.5, 127.7, 127.1, 126.9, 126.7, 126.4, 126.2, 125.9, 125.85, 125.77, 125.1, 124.19, 124.17, 123.3, 121.8, 107.7 (d, *J* = 7.2 Hz), 106.2, 105.5, 105.0 (d, *J* = 6.9 Hz), 98.5 (d, *J* = 6.6 Hz), 94.0 (d, *J* = 8.1 Hz), 93.6 (d, *J* = 8.5 Hz), 55.49, 55.45, 34.8, 31.2, 22.5, 21.6, 10.01, 10.00 ppm; **IR** (ATR) ṽ = 2960 (s), 2905 (w), 2867 (w), 1619 (w), 1596 (m), 1450 (m), 1422 (m), 1392 (w), 1363 (w), 1330 (w), 1295 (w), 1245 (w), 1237 (w), 1223 (w), 1198 (w), 1163 (w), 1110 (s), 1028 (m), 862 (w), 831 (w), 745 (m), 732 (w) cm^{-1}; **HRMS** (APCI/QTOF) *m/z* = calcd. for $[C_{45}H_{47}ClO_2Rh]^+$, $[(M/2)-Cl^-]^+$: 757.2314, found: 757.2327.; $[\alpha]_D^{26}$ = -11.9 (*c* = 0.07, $CHCl_3$).

Rhodium complex (R_a)-**Rh11**

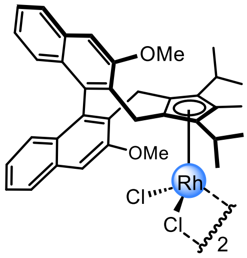

According to *General Procedure 4a*, starting from an isolated mixture of Cp^VH isomers **Cp^V14** and spirodiene **S14** (25:75 ratio, 34 µmol of Cp^VH), chiral Rh(III)-complex **Rh11** (7 mg, 11 µmol of monomer, 32% yield) was obtained as a dark red solid.

^{1}H NMR (600 MHz, d_6-DMSO) δ = 7.92 (d, J = 8.2 Hz, 1H), 7.90 (d, J = 8.3 Hz, 1H), 7.56 (s, 1H), 7.45 – 7.38 (m, 3H), 7.12 – 7.08 (m, 1H), 7.08 – 7.04 (m, 1H), 6.74 (d, J = 8.5 Hz, 1H), 6.70 (d, J = 8.5 Hz, 1H), 4.29 (d, J = 15.4 Hz, 1H), 4.03 (d, J = 14.7 Hz, 1H), 4.01 (s, 3H), 3.93 (s, 3H), 3.07 (d, J = 14.7 Hz, 1H), 2.94 – 2.85 (m, 2H), 2.20 (d, J = 15.3 Hz, 1H), 1.86 (s, 3H), 1.41 – 1.37 (m, 6H), 1.33 (d, J = 7.0 Hz, 3H), 1.15 (d, J = 7.1 Hz, 3H) ppm; **^{13}C{^{1}H} NMR** (151 MHz, d_6-DMSO) δ = 155.8, 154.5, 137.2, 137.1, 133.7, 133.5, 127.1, 126.9, 126.5, 126.4, 126.18, 126.16, 125.9, 124.2, 124.1, 123.8, 123.6, 107.1, 106.4, 105.8, 102.6 (d, J = 6.4 Hz), 102.5 (d, J = 6.9 Hz), 98.7 (d, J = 6.4 Hz), 55.5, 55.4, 25.3, 24.2, 22.7, 21.8, 20.95, 20.86, 20.2, 20.0, 11.0 ppm; **IR** (ATR) ṽ = 2966 (w), 2933 (w), 1619 (w), 1597 (m), 1573 (w), 1453 (m), 1423 (m), 1387 (w), 1366 (w), 1329 (w), 1296 (w), 1237 (m), 1198 (w), 1163 (w), 1151 (w), 1111 (m), 1076 (w), 1020 (m), 910 (m), 863 (w), 832 (w), 730 (s), 637 (w) cm^{-1}; **HRMS** (nanochip-ESI/LTQ-Orbitrap) m/z = calcd. for $[C_{36}H_{37}ClO_2Rh]^+$, $[(M/2)\text{-}Cl^-]^+$: 639.1532, found: 639.1537; $[\boldsymbol{\alpha}]_{\mathbf{D}}^{\mathbf{22}}$ = -33.3 (c = 0.02, $CHCl_3$).

Rhodium complex (S_a)-**Rh12**

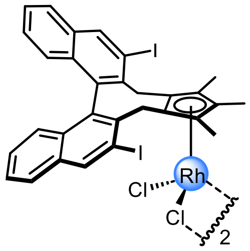

According to *General Procedure 4b*, starting from an isolated mixture of Cp^VH isomers **Cp^V16** and spirodiene **S16** (80:20 ratio, 70 µmol of Cp^VH) and using $[Rh(COD)OAc]_2$ as precursor and SO_2Cl_2 as oxidant, chiral Rh(III)-complex **Rh12** (29 mg, 36 µmol of monomer, 51% yield over 2 steps) was obtained as a dark red solid. Notably, the Cp-ring's ^{13}C NMR signals were not all well resolved.

^{1}H NMR (400 MHz, d_6-DMSO) δ = 8.78 (s, 1H), 8.63 (s, 1H), 7.99 – 7.94 (m, 2H), 7.52 – 7.44 (m, 2H), 7.30 – 7.23 (m, 2H), 6.72 (d, J = 8.5 Hz, 1H), 6.59 (d, J = 8.5 Hz, 1H), 3.96 (d, J = 15.9 Hz, 1H), 3.71 (d, J = 14.6 Hz, 1H), 3.58 (d, J = 14.4 Hz, 1H), 2.72 (d, J = 15.9 Hz, 1H), 2.20 (s, 3H), 1.81 (s, 3H), 1.54 (s, 3H) ppm; **^{13}C{^{1}H} NMR** (151 MHz, d_6-DMSO) δ = 140.0, 138.8, 137.2, 135.8, 134.4, 133.6, 133.5, 132.9, 130.8, 130.7, 127.5, 127.1, 127.0, 126.8, 126.4, 125.9, 125.8, 103.3, 100.6 (d, J = 7.0 Hz), 97.7, 95.2, 88.7 (d, J = 7.6 Hz), 34.9, 31.8, 12.2, 10.2, 7.9 ppm; **IR** (ATR) ṽ = 2919 (w), 1558 (w), 1445 (w), 1377 (w), 1023 (w), 976 (w), 909 (m), 864 (w), 851 (w), 818 (w), 776 (w), 729 (s), 644 (w), 445 (w) cm^{-1}; **HRMS** (ESI/ QTOF) m/z = calcd. for $[C_{30}H_{23}ClI_2Rh]^+$, $[(M/2)\text{-}Cl^-]^+$: 774.8627, found: 774.8623; $[\boldsymbol{\alpha}]_{\mathbf{D}}^{\mathbf{22}}$ = -130.0 (c = 0.05, $CHCl_3$).

Rhodium complex (S_a)-**Rh13**

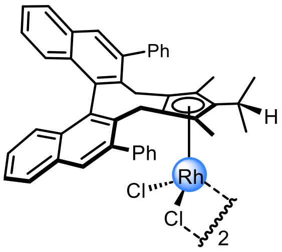

According to *General Procedure 4a*, starting from an isolated mixture of Cp^VH isomers **Cp^V17** and spirodiene **S17** (41:59 ratio, 77 µmol of Cp^VH), chiral Rh(III)-complex **Rh13** (47 mg, 64 µmol of monomer, 83% yield) was obtained as a dark red solid.

^{1}H NMR (400 MHz, d_6-DMSO) δ = 8.08 (d, J = 8.2 Hz, 1H), 8.01 (d, J = 8.3 Hz, 1H), 7.93 (s, 2H), 7.61 – 7.22 (m, 14H), 6.99 (d, J = 8.5 Hz, 1H), 6.62 (d, J = 8.5 Hz, 1H), 3.79 (d, J = 14.1 Hz, 1H), 3.67 – 3.58 (m, 2H), 2.44 (d, J = 16.3 Hz, 1H), 2.41 – 2.31 (m, 1H), 1.47 (s, 3H), 1.21 (d, J = 7.1 Hz, 3H), 0.95 (d, J = 7.0 Hz, 3H), 0.67 (s, 3H) ppm; **^{13}C{^{1}H} NMR** (101 MHz, d_6-DMSO) δ = 142.3, 141.0, 140.8, 139.2, 136.9, 136.3, 132.1, 132.0, 131.2, 131.1, 130.9, 130.2, 128.49, 128.46, 128.2, 128.1, 128.0, 127.8, 126.9, 126.8, 126.3, 126.1, 125.9, 125.7, 111.0 (d, J = 6.3 Hz), 101.0 (d, J = 8.2 Hz), 99.2 (d, J = 7.2 Hz), 94.9 (d, J = 8.9 Hz), 94.4 (d, J = 7.4 Hz), 27.2, 24.3, 24.2, 20.5, 20.1, 10.4, 6.2 ppm; **IR** (ATR) ṽ = 3054 (w), 2967 (w), 2930 (w), 1493 (w), 1447 (w), 1025 (w), 907 (m), 764 (w), 750 (m), 728 (s), 703 (s), 645 (w) cm^{-1}; **HRMS** (nanochip-ESI/LTQ-Orbitrap) m/z = calcd. for $[C_{44}H_{37}ClRh]^+$, $[(M/2)\text{-}Cl^-]^+$: 703.1633, found: 703.1630; $[\alpha]_D^{26}$ = -122.2 (c = 0.06, $CHCl_3$).

Rhodium complex (S_a)-**Rh14**

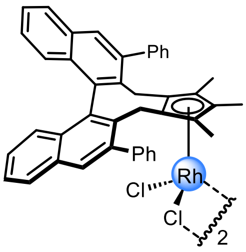

According to *General Procedure 4a*, starting from an isolated mixture of Cp^VH isomers **Cp^V18** and spirodiene **S18** (66:34 ratio, 128 µmol of Cp^VH), chiral Rh(III)-complex **Rh14** (44 mg, 61 µmol of monomer, 48% yield) was obtained as a dark red solid. Notably, the complex' other enantiomer (+)-(R_a)-**Rh14** is reported (and characterized in $CDCl_3$),[23] but its synthesis required 8 steps from dibromide (R_a)-**L5** contrary to the 2-step dialkylation-complexation sequence described herein.

^{1}H NMR (600 MHz, d_6-DMSO) δ = 8.10 – 7.99 (m, 3H), 7.95 (d, J = 9.6 Hz, 2H), 7.60 – 7.56 (m, 2H), 7.54 – 7.43 (m, 8H), 7.41 – 7.37 (m, 1H), 7.32 – 7.28 (m, 1H), 7.27 – 7.23 (m, 1H), 6.96 – 6.91 (m, 1H), 6.65 – 6.61 (m, 1H), 3.81 (d, J = 14.3 Hz, 1H), 3.67 – 3.59 (m, 2H), 2.51 (d, J = 16.0 Hz, 1H), 1.35 (s, 3H), 1.33 (s, 3H), 0.72 (s, 3H) ppm; **^{13}C{^{1}H} NMR** (151 MHz, d_6-DMSO) δ = 142.2, 140.9, 140.8, 139.1, 136.9, 136.2, 132.1, 132.0, 131.3, 131.2, 131.0, 130.1, 128.5, 128.2, 128.1, 127.7, 126.9, 126.8, 126.3, 126.1, 125.9, 125.8, 125.6, 111.9 (d, J = 4.2 Hz), 102.8 (d, J = 7.1 Hz), 99.1 (d, J = 5.9 Hz), 92.5 (d, J = 5.8 Hz), 86.9 (d, J = 6.7 Hz), 27.4, 24.3, 9.6, 7.7, 6.2 ppm; **IR** (ATR) ṽ = 3053 (w), 2919 (w), 1589 (w), 1492 (w), 1448 (w), 1376 (w), 1266 (w), 1223 (w), 1074 (w), 1025 (w), 893 (w), 784 (w), 764 (m), 751 (m), 733 (m), 703 (s), 621 (w), 522 (w) cm^{-1}; **HRMS** (ESI/ QTOF) m/z = calcd. for $[C_{42}H_{33}ClRh]^+$, $[(M/2)\text{-}Cl^-]^+$: 675.1320, found: 675.1326; $[\alpha]_D^{26}$ = -23.3 (c = 0.06, $CHCl_3$).

Rhodium complex (*R*)-**Rh15**

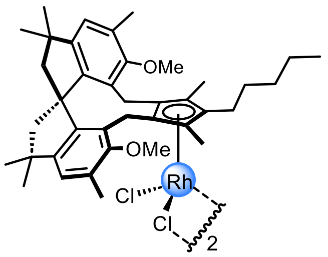

According to *General Procedure 4a*, starting from an isolated mixture of $Cp^{V}H$ isomers **Cp^{V}19** and spirodiene **S19** (98:2 ratio, 119 µmol of $Cp^{V}H$), chiral Rh(III)-complex **Rh15** (36 mg, 50 µmol of monomer, 42% yield) was obtained as a dark red solid.

^{1}H NMR (400 MHz, $CDCl_3$) δ = 6.97 (s, 1H), 6.92 (s, 1H), 3.81 (s, 3H), 3.67 (s, 3H), 3.38 (d, *J* = 14.6 Hz, 1H), 3.24 (d, *J* = 14.7 Hz, 1H), 3.16 (d, *J* = 14.5 Hz, 1H), 2.63 – 2.51 (m, 2H), 2.44 (d, *J* = 13.0 Hz, 1H), 2.24 – 2.19 (m, 7H), 2.18 – 2.10 (m, 2H), 2.02 (d, *J* = 13.0 Hz, 1H), 1.97 (s, 3H), 1.56 (s, 3H), 1.46 (s, 3H), 1.42 (s, 3H), 1.37 (s, 3H), 1.33 – 1.31 (m, 4H), 1.31 – 1.25 (m, 5H), 0.88 (t, *J* = 6.7 Hz, 3H) ppm; **^{13}C{^{1}H} NMR** (101 MHz, $CDCl_3$) δ = 157.0, 156.8, 147.9, 147.3, 146.9, 145.2, 133.0, 131.0, 125.3, 124.7, 123.9, 122.1, 97.7, 95.3 (d, *J* = 7.3 Hz), 90.4, 60.3, 58.9, 58.64, 58.60, 57.2, 42.2, 41.8, 32.0, 31.8, 31.4, 31.2, 26.9, 24.7, 22.5, 21.2, 20.0, 17.4, 16.7, 14.0, 10.6, 9.0 ppm; **IR** (ATR) ṽ = 2954 (s), 2927 (m), 2861 (w), 1465 (m), 1405 (w), 1362 (w), 1325 (w), 1312 (w), 1218 (w), 1092 (w), 1062 (w), 1022 (w), 997 (w), 920 (w), 730 (m) cm^{-1}; **HRMS** (ESI/QTOF) *m/z* = calcd. for $[C_{39}H_{51}ClO_2Rh]^+$, $[(M/2)\text{-}Cl^-]^+$: 689.2627, found: 689.2642; $[\alpha]_D^{26}$ = -288.9 (*c* = 0.03, $CHCl_3$).

Rhodium complex (*R*)-**Rh16**

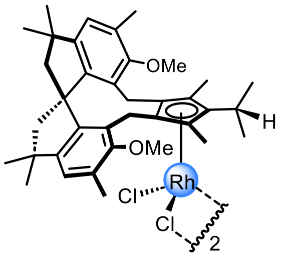

According to *General Procedure 4a*, starting from an isolated mixture of $Cp^{V}H$ isomers **Cp^{V}20** and spirodiene **S20** (98:2 ratio, 109 µmol of $Cp^{V}H$), chiral Rh(III)-complex **Rh16** (65 mg, 93 µmol of monomer, 85% yield) was obtained as a dark red solid. Notably, the Cp-ring's ^{13}C NMR signals were not well resolved.

^{1}H NMR (400 MHz, $CDCl_3$) δ = 6.96 (s, 1H), 6.92 (s, 1H), 3.79 (s, 3H), 3.67 (s, 3H), 3.40 (d, *J* = 14.7 Hz, 1H), 3.30 (d, *J* = 14.7 Hz, 1H), 3.17 (d, *J* = 14.4 Hz, 1H), 2.81 (hept, *J* = 7.1 Hz, 1H), 2.52 (d, *J* = 13.2 Hz, 1H), 2.43 (d, *J* = 13.0 Hz, 1H), 2.23 (s, 3H), 2.21 (s, 3H), 2.17 (d, *J* = 14.4 Hz, 1H), 2.12 (d, *J* = 13.2 Hz, 1H), 2.04 (d, *J* = 13.0 Hz, 1H), 2.00 (s, 3H), 1.65 (s, 3H), 1.46 – 1.41 (m, 9H), 1.37 (s, 3H), 1.33 – 1.29 (m, 6H) ppm; **^{13}C{^{1}H} NMR** (101 MHz, $CDCl_3$) δ = 157.2, 156.9, 147.9, 147.3, 147.0, 145.2, 132.9, 131.0, 125.4, 124.7, 123.8, 121.5, 104.0, 101.8, 95.6 (d, *J* = 7.6 Hz), 93.0, 90.8, 60.3, 58.8, 58.63, 58.60, 57.3, 42.2, 41.8, 32.0, 31.4, 31.3, 31.1, 26.0, 21.3, 21.2, 20.0, 19.8, 17.3, 16.7, 11.9, 9.3 ppm; **IR** (ATR) ṽ = 2954 (s), 2862 (w), 1461 (s), 1404 (w), 1381 (w), 1362 (w), 1325 (m), 1312 (w), 1265 (w), 1210 (m), 1164 (w), 1091 (m), 1054 (w), 1022 (w), 1008 (w), 992 (m), 921 (w), 876 (w), 731 (s), 699 (w) cm^{-1}; **HRMS** (APCI/QTOF) *m/z* = calcd. for $[C_{37}H_{47}ClO_2Rh]^+$, $[(M/2)\text{-}Cl^-]^+$: 661.2314, found: 661.2330; $[\alpha]_D^{26}$ = -320.8 (*c* = 0.04, $CHCl_3$).

Rhodium complex (*R*)-**Rh17**

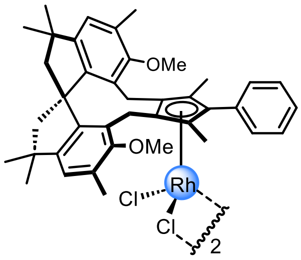

According to *General Procedure 4a*, starting from an isolated mixture of $Cp^{V}H$ isomers **Cp^{V}21** and spirodiene **S21** (97:3 ratio, 95 µmol of $Cp^{V}H$), chiral Rh(III)-complex **Rh17** (60 mg, 82 µmol of monomer, 86% yield) was obtained as a dark red solid. Notably, the Cp-ring's ^{13}C NMR signals were not well resolved.

^{1}H NMR (400 MHz, $CDCl_3$) δ = 7.92 – 7.80 (m, 2H), 7.50 – 7.37 (m, 3H), 7.01 – 6.90 (m, 2H), 3.80 (s, 3H), 3.70 (s, 3H), 3.63 – 3.49 (m, 2H), 3.25 (d, *J* = 14.2 Hz, 1H), 2.52 (d, *J* = 13.2 Hz, 1H), 2.45 (d, *J* = 13.1 Hz, 1H), 2.27 (s, 3H), 2.23 – 2.18 (m, 4H), 2.15 – 2.07 (m, 2H), 1.98 (s, 3H), 1.57 (s, 3H), 1.46 (s, 3H), 1.44 (s, 3H), 1.40 (s, 3H), 1.32 (s, 3H) ppm; **$^{13}C\{^{1}H\}$ NMR** (101 MHz, $CDCl_3$) δ = 157.3, 156.9, 147.5, 147.1, 145.1, 132.5, 131.0, 130.7, 129.6, 128.7, 128.0, 125.5, 124.8, 123.6, 95.1, 60.4, 59.1, 58.8, 58.6, 57.7, 42.2, 41.8, 32.0, 31.5, 31.4, 31.2, 21.4, 20.5, 17.2, 16.7, 11.8, 9.8 ppm; **IR** (ATR) ṽ = 2954 (m), 2924 (w), 2861 (w), 1463 (m), 1447 (m), 1405 (w), 1362 (w), 1326 (w), 1313 (w), 1218 (m), 1092 (w), 1065 (w), 1018 (w), 994 (m), 911 (m), 765 (w), 729 (s), 700 (w) cm^{-1}; **HRMS** (APCI/QTOF) *m/z* = calcd. for $[C_{40}H_{45}ClO_2Rh]^+$, $[(M/2)-Cl^-]^+$: 695.2158, found: 695.2185; $[\alpha]_D^{26}$ = -387.1 (*c* = 0.07, $CHCl_3$).

Rhodium complex (*R*)-**Rh18**

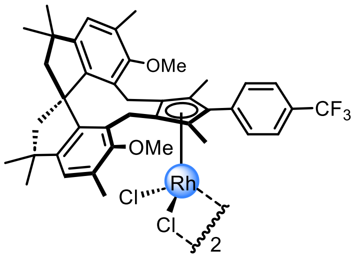

According to *General Procedure 4a*, starting from an isolated mixture of $Cp^{V}H$ isomers **Cp^{V}22** and spirodiene **S22** (92:8 ratio, 40 µmol of $Cp^{V}H$), chiral Rh(III)-complex **Rh18** (25 mg, 31 µmol of monomer, 78% yield) was obtained as a dark red solid. Notably, the complex slowly decomposes in d_6-DMSO solution (as seen during ^{13}C NMR acquisition) toward fulvene **SI41** (2.7:1 ratio **Rh18/SI41** after 10 h, and full conversion within 3 days).

^{1}H NMR (400 MHz, d_6-DMSO) δ = 7.91 (d, *J* = 8.1 Hz, 2H), 7.77 (d, *J* = 8.3 Hz, 2H), 7.04 (s, 1H), 6.89 (s, 1H), 3.76 – 3.69 (m, 4H), 3.65 (s, 3H), 3.48 – 3.35 (m, 2H), 2.73 (d, *J* = 14.1 Hz, 1H), 2.34 – 2.27 (m, 2H), 2.23 (s, 3H), 2.18 (s, 3H), 2.00 (d, *J* = 12.9 Hz, 1H), 1.86 (s, 3H), 1.80 (d, *J* = 13.4 Hz, 1H), 1.75 (s, 3H), 1.41 (s, 3H), 1.36 (s, 3H), 1.28 (s, 3H), 1.23 (s, 4H) ppm; **$^{13}C\{^{1}H\}$ NMR** (101 MHz, d_6-DMSO) δ = 157.9, 156.7, 147.1, 146.2, 145.0, 144.9, 133.9, 131.3, 130.3, 129.4, 128.8 (q, *J* = 32.0 Hz), 125.0, 124.7, 124.2 (q, *J* = 272.5 Hz), 124.1, 122.8 – 122.7 (m), 109.6 (d, *J* = 6.0 Hz), 103.1, 102.6, 97.9 (d, *J* = 6.2 Hz), 88.3 (d, *J* = 9.5 Hz), 60.0, 59.6, 59.1, 58.5, 56.6, 40.7, 40.4, 32.6, 31.6, 30.7, 30.5, 20.3, 20.2, 16.9, 16.3, 11.6, 10.0 ppm; **$^{19}F\{^{1}H\}$ NMR** (376 MHz, d_6-DMSO) δ = -61.14 ppm; **IR** (ATR) ṽ = 2955 (w), 2862 (w), 1464 (w), 1406 (w), 1362 (w), 1323 (s), 1218 (w), 1166 (m), 1128 (m), 1111 (w), 1093 (w), 1067 (m), 1020 (w), 996 (w), 910 (w), 731 (m) cm^{-1}; **HRMS** (APCI/QTOF) *m/z* = calcd. for $[C_{41}H_{44}ClF_3O_2Rh]^+$, $[(M/2)-Cl^-]^+$: 763.2031, found: 763.2026; $[\alpha]_D^{26}$ = -273.3 (*c* = 0.05, $CHCl_3$).

Fulvene (*R*)-**SI41**

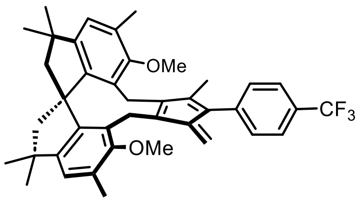

Fulvene **SI41** was isolated from a d_6-DMSO solution of **Rh18** after 3 days *via* lyophilization and was obtained as a light-brown solid. The HSQC spectrum clearly identified its characteristic olefinic ^{1}H and ^{13}C NMR signals (δ_H = 5.51 and 6.07 ppm; δ_C = 116.8 ppm in d_6-DMSO).

^{1}H NMR (400 MHz, d_6-DMSO) δ = 7.73 (d, *J* = 8.2 Hz, 2H), 7.34 (d, *J* = 8.1 Hz, 2H), 6.93 (s, 1H), 6.92 (s, 1H), 6.07 (s, 1H), 5.51 (s, 1H), 3.65 (d, *J* = 13.9 Hz, 1H), 3.58 (s, 3H), 3.50 – 3.45 (m, 4H), 3.16 – 3.10 (m, 1H), 3.10 – 3.03 (m, 1H), 2.39 (d, *J* = 13.2 Hz, 2H), 2.19 (s, 3H), 2.16 (s, 3H), 2.05 (s, 3H), 2.00 – 1.93 (m, 2H), 1.40 (s, 6H), 1.26 (s, 6H) ppm; **^{13}C{^{1}H} NMR** (101 MHz, d_6-DMSO) δ = 155.6, 155.3, 150.9, 146.1, 146.0, 145.7, 145.5, 142.63, 142.59, 139.6, 130.8, 130.6, 129.8, 129.7, 127.4, 126.4 (q, *J* = 31.7 Hz), 125.6, 125.5, 125.0 (q, *J* = 3.4 Hz), 124.5 (q, *J* = 271.9 Hz), 123.1, 122.9, 116.8, 59.8, 59.7, 58.0, 57.7, 57.5, 40.8, 40.2, 31.6, 31.5, 30.84, 30.82, 21.2, 20.0, 16.34, 16.29, 11.8 ppm; **^{19}F{^{1}H} NMR** (376 MHz, d_6-DMSO) δ = -60.71 ppm; **IR** (ATR) ṽ = 2954 (w), 2931 (w), 2861 (w), 1614 (w), 1463 (w), 1406 (w), 1361 (w), 1324 (s), 1218 (w), 1162 (w), 1124 (m), 1091 (w), 1066 (m), 1018 (w), 823 (w), 775 (w) cm^{-1}; **HRMS** (ESI/QTOF) *m/z* = calcd. for $[C_{41}H_{44}F_3O_2]^+$, $[M+H]^+$: 625.3288, found: 625.3295.

Rhodium complex (*R*)-**Rh19**

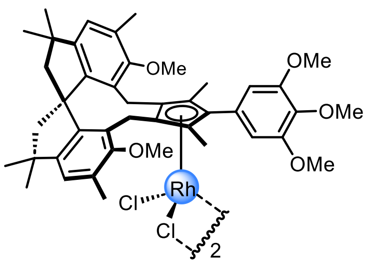

According to *General Procedure 4a*, starting from an isolated mixture of Cp^{V}H isomers **Cp^{V}23** and spirodiene **S23** (94:6 ratio, 71 µmol of Cp^{V}H), chiral Rh(III)-complex **Rh19** (54 mg, 66 µmol of monomer, 93% yield) was obtained as a dark red solid.

^{1}H NMR (400 MHz, $CDCl_3$) δ = 7.22 (s, 2H), 6.97 (s, 1H), 6.95 (s, 1H), 3.91 (s, 6H), 3.89 (s, 3H), 3.79 (s, 3H), 3.73 (s, 3H), 3.67 (d, *J* = 14.6 Hz, 1H), 3.56 (d, *J* = 14.5 Hz, 1H), 3.25 (d, *J* = 13.9 Hz, 1H), 2.52 (d, *J* = 13.3 Hz, 1H), 2.45 (d, *J* = 13.1 Hz, 1H), 2.28 (s, 3H), 2.23 – 2.15 (m, 4H), 2.15 – 2.06 (m, 2H), 2.01 (s, 3H), 1.58 (s, 3H), 1.46 (s, 3H), 1.43 (s, 3H), 1.40 (s, 3H), 1.31 (s, 3H) ppm; **^{13}C{^{1}H} NMR** (101 MHz, $CDCl_3$) δ = 157.3, 156.8, 153.3, 147.9, 147.6, 147.2, 145.1, 139.0, 132.9, 131.0, 125.6, 124.8, 123.5, 123.0, 120.4, 108.4, 102.8, 100.4, 94.9, 94.2 (d, *J* = 6.8 Hz), 93.4, 60.9, 60.4, 59.3, 58.8, 58.5, 57.9, 56.8, 42.2, 41.8, 32.0, 31.6, 31.4, 31.2, 21.4, 20.6, 17.1, 16.7, 11.9, 9.8 ppm; **IR** (ATR) ṽ = 2954 (m), 2862 (w), 1584 (w), 1464 (m), 1411 (m), 1362 (w), 1325 (w), 1310 (w), 1241 (w), 1218 (w), 1162 (w), 1125 (s), 1093 (w), 1073 (w), 995 (m), 910 (m), 728 (s), 645 (w) cm^{-1}; **HRMS** (nanochip-ESI/LTQ-Orbitrap) *m/z* = calcd. for $[C_{43}H_{51}ClO_5Rh]^+$, $[(M/2)-Cl^-]^+$: 785.2475, found: 785.2503; $[\alpha]_D^{26}$ = -428.6 (*c* = 0.07, $CHCl_3$).

Rhodium complex (*R*)-**Rh20a**

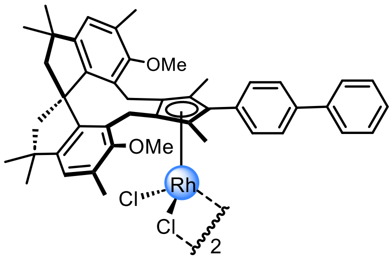

According to *General Procedure 4a*, starting from an isolated mixture of Cp^VH isomers $\mathbf{Cp^V24}$ and spirodiene **S24** (98:2 ratio, 50 µmol of Cp^VH), chiral Rh(III)-complex **Rh20a** (30 mg, 38 µmol of monomer, 75% yield) was obtained as a dark red solid.

^{1}H NMR (600 MHz, d_6-DMSO) δ = 7.78 (d, *J* = 8.0 Hz, 2H), 7.75 – 7.73 (m, 3H), 7.73 – 7.70 (m, 2H), 7.48 (t, *J* = 7.7 Hz, 2H), 7.39 (t, *J* = 7.4 Hz, 1H), 7.04 (s, 1H), 6.89 (s, 1H), 3.76 – 3.70 (m, 4H), 3.66 (s, 3H), 3.44 (d, *J* = 14.2 Hz, 1H), 3.37 (d, *J* = 13.7 Hz, 1H), 2.72 (d, *J* = 14.0 Hz, 1H), 2.34 – 2.28 (m, 2H), 2.23 (s, 3H), 2.18 (s, 3H), 2.00 (d, *J* = 12.9 Hz, 1H), 1.89 (s, 3H), 1.83 – 1.77 (m, 4H), 1.42 (s, 4H), 1.37 (s, 4H), 1.28 (s, 3H), 1.24 (s, 3H) ppm; **^{13}C{^{1}H} NMR** (151 MHz, d_6-DMSO) δ = 157.9, 156.7, 147.1, 146.2, 145.0, 144.9, 140.1, 139.4, 131.0, 130.3, 129.4, 129.0, 128.2, 127.8, 126.8, 126.7, 126.5, 126.3, 124.7, 124.0, 122.9, 122.8, 109.2, 102.3, 97.8, 89.8, 60.0, 59.6, 59.1, 58.4, 56.7, 40.8, 40.5, 32.6, 31.6, 30.7, 30.5, 20.4, 20.2, 16.9, 16.3, 11.8, 10.1 ppm; **IR** (ATR) ṽ = 2952 (s), 2923 (m), 2859 (w), 1489 (w), 1461 (w), 1447 (s), 1404 (s), 1383 (m), 1361 (w), 1325 (w), 1312 (w), 1264 (w), 1217 (m), 1164 (w), 1092 (m), 1065 (w), 1019 (m), 996 (s), 918 (w), 875 (w), 854 (w), 837 (w), 765 (s), 732 (s), 697 (s) cm^{-1}; **HRMS** (ESI/QTOF) *m/z* = calcd. for $[C_{46}H_{49}ClO_2Rh]^+$, $[(M/2)-Cl^-]^+$: 771.2471, found: 771.2452; $[\alpha]_D^{26}$ = -356.7 (*c* = 0.05, $CHCl_3$).

Rhodium complex (*R*)-**Rh21**

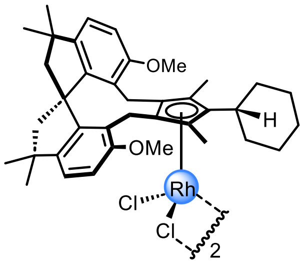

According to *General Procedure 4a*, starting from an isolated mixture of Cp^VH isomers $\mathbf{Cp^V25}$ and spirodiene **S25** (98:2 ratio, 177 µmol of Cp^VH), chiral Rh(III)-complex **Rh21** (106 mg, 149 µmol of monomer, 84% yield) was obtained as a dark red solid.

^{1}H NMR (400 MHz, d_6-DMSO) δ = 7.13 (d, *J* = 8.3 Hz, 1H), 6.98 (d, *J* = 8.3 Hz, 1H), 6.90 (d, *J* = 8.4 Hz, 1H), 6.65 (d, *J* = 8.3 Hz, 1H), 3.73 (s, 3H), 3.68 (s, 3H), 3.61 (d, *J* = 13.7 Hz, 1H), 3.49 (d, *J* = 14.0 Hz, 1H), 3.30 (d, *J* = 14.1 Hz, 1H), 2.47 (d, *J* = 14.0 Hz, 1H), 2.35 – 2.27 (m, 2H), 2.21 – 2.11 (m, 2H), 1.96 (d, *J* = 12.5 Hz, 1H), 1.93 – 1.84 (m, 6H), 1.78 – 1.68 (m, 4H), 1.67 – 1.58 (m, 2H), 1.42 (s, 3H), 1.36 (s, 3H), 1.32 – 1.24 (m, 5H), 1.23 – 1.13 (m, 5H) ppm; **^{13}C{^{1}H} NMR** (101 MHz, d_6-DMSO) δ = 157.8, 156.8, 148.3, 146.9, 143.8, 141.3, 122.3, 121.8, 117.7, 117.5, 110.7, 110.4, 109.7, 101.3, 97.8, 97.1, 93.7, 59.0, 58.3, 56.3, 55.9, 55.2, 40.4, 34.8, 33.0, 31.8, 30.8, 30.6, 30.5, 29.95, 29.88, 26.5, 26.4, 25.5, 19.8, 18.9, 12.1, 11.1, 9.9 ppm; **IR** (ATR) ṽ = 2952 (m), 2927 (m), 2856 (w), 1591 (w), 1477 (m), 1464 (m), 1450 (m), 1362 (w), 1319 (m), 1260 (s), 1207 (w), 1097 (m), 1073 (w), 1001 (w), 910 (m), 808 (w), 730 (s), 645 (w) cm^{-1}; **HRMS** (ESI/QTOF) *m/z* = calcd. for $[C_{38}H_{47}ClO_2Rh]^+$, $[(M/2)-Cl^-]^+$: 673.2314, found: 673.2320; $[\alpha]_D^{22}$ = -289.3 (*c* = 0.09, $CHCl_3$).

Rhodium complex (*R*)-**Rh22**

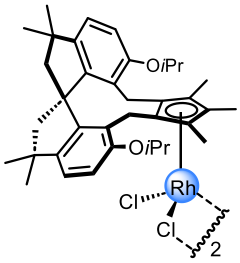

According to *General Procedure 4a*, starting from an isolated mixture of Cp^VH isomers **Cp^V26** and spirodiene **S26** (92:8 ratio, 185 µmol of Cp^VH), chiral Rh(III)-complex **Rh22** (105 mg, 150 µmol of monomer, 81% yield) was obtained as a dark red solid.

^{1}H NMR (500 MHz, d_6-DMSO) δ = 7.09 (d, *J* = 8.4 Hz, 1H), 6.96 (d, *J* = 8.4 Hz, 1H), 6.94 (d, *J* = 8.5 Hz, 1H), 6.70 (d, *J* = 8.5 Hz, 1H), 4.55 (hept, *J* = 6.1 Hz, 1H), 4.46 (hept, *J* = 5.9 Hz, 1H), 3.63 – 3.57 (m, 2H), 3.37 (d, *J* = 13.6 Hz, 1H), 2.55 (d, *J* = 13.8 Hz, 1H), 2.29 – 2.22 (m, 2H), 1.94 (d, *J* = 12.9 Hz, 1H), 1.84 (s, 3H), 1.77 (s, 3H), 1.75 – 1.71 (m, 1H), 1.53 – 1.49 (m, 6H), 1.40 (s, 3H), 1.34 – 1.31 (m, 6H), 1.27 – 1.24 (m, 6H), 1.22 – 1.19 (m, 6H) ppm; **^{13}C{^{1}H} NMR** (126 MHz, d_6-DMSO) δ = 155.9, 155.7, 148.3, 147.5, 143.6, 140.9, 122.4, 121.7, 118.7, 118.0, 114.0, 112.4, 108.9, 100.7, 97.4 (d, *J* = 6.9 Hz), 94.8 (d, *J* = 8.4 Hz), 92.3, 71.8, 69.2, 59.6, 58.6, 56.5, 40.4, 40.1, 32.9, 31.8, 30.8, 30.6, 22.9, 22.3, 21.5, 20.0, 19.3, 10.7, 9.9, 8.5 ppm; **IR** (ATR) ṽ = 2956 (m), 2924 (w), 2862 (w), 1473 (m), 1383 (w), 1362 (w), 1313 (w), 1256 (s), 1208 (w), 1135 (w), 1115 (s), 1072 (w), 1050 (w), 949 (w), 910 (m), 808 (w), 729 (s), 644 (w) cm^{-1}; **HRMS** (ESI/QTOF) *m/z* = calcd. for $[C_{37}H_{47}ClO_2Rh]^+$, $[(M/2)\text{-}Cl^-]^+$: 661.2314, found: 661.2327; $[\alpha]_D^{22}$ = -187.5 (*c* = 0.08, $CHCl_3$).

Rhodium complex (*R*)-**Rh23**

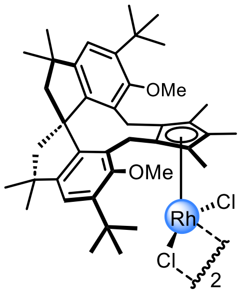

According to *General Procedure 4a*, starting from an isolated mixture of Cp^VH isomers **Cp^V27** and spirodiene **S27** (98:2 ratio, 184 µmol of Cp^VH), chiral Rh(III)-complex **Rh23** (118 mg, 157 µmol of monomer, 85% yield) was obtained as a dark red solid.

^{1}H NMR (500 MHz, d_6-DMSO) δ = 7.07 (s, 1H), 7.01 (s, 1H), 3.74 (s, 3H), 3.66 (s, 3H), 3.63 (d, *J* = 14.0 Hz, 1H), 3.44 (d, *J* = 14.4 Hz, 1H), 3.31 (d, *J* = 14.0 Hz, 1H), 2.56 (d, *J* = 14.5 Hz, 1H), 2.32 – 2.25 (m, 2H), 1.99 (d, *J* = 12.9 Hz, 1H), 1.83 (d, *J* = 13.2 Hz, 1H), 1.79 (s, 3H), 1.77 (s, 3H), 1.50 (s, 3H), 1.38 – 1.35 (m, 12H), 1.33 – 1.31 (m, 9H), 1.31 – 1.29 (m, 6H), 1.26 (s, 3H) ppm; **^{13}C{^{1}H} NMR** (126 MHz, d_6-DMSO) δ = 159.6, 158.4, 147.3, 146.6, 146.2, 144.5, 141.9, 141.5, 123.9, 123.8, 120.7, 120.3, 108.1 (d, *J* = 5.4 Hz), 99.8 (d, *J* = 6.3 Hz), 98.1 (d, *J* = 8.5 Hz), 96.0 (d, *J* = 7.5 Hz), 93.4 (d, *J* = 6.3 Hz), 62.6, 62.1, 59.1, 58.7, 57.7, 41.0, 40.9, 34.85, 34.82, 32.3, 32.0, 31.3, 31.2, 31.0, 30.6, 21.0, 20.3, 9.9, 9.8, 8.4 ppm; **IR** (ATR) ṽ = 2955 (s), 2864 (w), 1450 (w), 1399 (w), 1361 (w), 1314 (w), 1251 (w), 1223 (m), 1079 (w), 1023 (w), 999 (w), 988 (w), 910 (m), 731 (s) cm^{-1}; **HRMS** (ESI/QTOF) *m/z* = calcd. for $[C_{41}H_{55}ClO_2Rh]^+$, $[(M/2)\text{-}Cl^-]^+$: 717.2940, found: 717.2946; $[\alpha]_D^{22}$ = -110.0 (*c* = 0.10, $CHCl_3$).

Rhodium complex (*R*)-**Rh24**

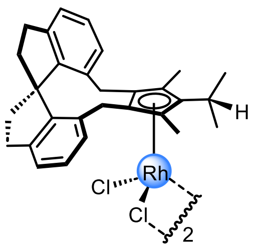

According to *General Procedure 4a*, starting from an isolated mixture of Cp^VH isomers **Cp^V28** and spirodiene **S28** (88:12 ratio, 176 µmol of Cp^VH), chiral Rh(III)-complex **Rh24** (96 mg, 174 µmol of monomer, 99% yield) was obtained as a dark red solid.

^{1}H NMR (600 MHz, d_6-DMSO) δ = 7.59 (d, *J* = 7.4 Hz, 1H), 7.20 – 7.14 (m, 2H), 7.12 – 7.05 (m, 2H), 6.90 (d, *J* = 7.2 Hz, 1H), 3.57 (d, *J* = 13.6 Hz, 1H), 3.31 – 3.24 (m, 2H), 3.03 – 2.91 (m, 2H), 2.85 – 2.78 (m, 2H), 2.70 (hept, *J* = 7.1 Hz, 1H), 2.63 (d, *J* = 15.2 Hz, 1H), 2.25 – 2.18 (m, 2H), 1.88 – 1.84 (m, 4H), 1.83 (s, 3H), 1.77 – 1.70 (m, 1H), 1.33 (d, *J* = 7.0 Hz, 3H), 1.13 (d, *J* = 7.1 Hz, 3H) ppm; **^{13}C{^{1}H} NMR** (151 MHz, d_6-DMSO) δ = 147.3, 146.6, 143.5, 141.9, 132.8, 130.8, 130.2, 128.1, 128.0, 127.5, 124.1, 123.6, 107.5, 107.3, 105.5, 92.9, 89.5, 60.9, 38.8, 37.5, 29.6, 29.4, 25.3, 25.1, 23.9, 20.5, 19.8, 10.2, 8.6 ppm; **IR** (ATR) ṽ = 2946 (m), 2848 (w), 1470 (w), 1449 (m), 1431 (w), 1367 (w), 1033 (w), 910 (m), 790 (w), 769 (w), 756 (m), 730 (s), 645 (w) cm^{-1}; **HRMS** (ESI/QTOF) *m/z* = calcd. for $[C_{29}H_{31}ClRh]^+$, $[(M/2)-Cl^-]^+$: 517.1164, found: 517.1174; $[\alpha]_D^{26}$ = -182.3 (*c* = 0.10, $CHCl_3$).

Rhodium complex (*R*)-**Rh25**

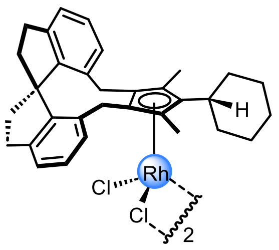

According to *General Procedure 4a*, starting from an isolated mixture of Cp^VH isomers **Cp^V29** and spirodiene **S29** (96:4 ratio, 301 µmol of Cp^VH), chiral Rh(III)-complex **Rh25** (164 mg, 277 µmol of monomer, 92% yield) was obtained as a dark red solid.

^{1}H NMR (500 MHz, d_6-DMSO) δ = 7.61 – 7.58 (m, 1H), 7.19 – 7.13 (m, 2H), 7.11 – 7.04 (m, 2H), 6.92 – 6.89 (m, 1H), 3.57 (d, *J* = 13.6 Hz, 1H), 3.30 – 3.23 (m, 2H), 3.03 – 2.91 (m, 2H), 2.86 – 2.78 (m, 2H), 2.62 (d, *J* = 15.1 Hz, 1H), 2.32 – 2.25 (m, 1H), 2.24 – 2.18 (m, 2H), 2.13 (d, *J* = 12.0 Hz, 1H), 1.90 – 1.81 (m, 7H), 1.77 – 1.63 (m, 5H), 1.55 – 1.46 (m, 1H), 1.40 – 1.27 (m, 2H), 1.26 – 1.14 (m, 2H) ppm; **^{13}C{^{1}H} NMR** (126 MHz, d_6-DMSO) δ = 147.3, 146.5, 143.5, 141.9, 132.9, 130.8, 130.2, 128.08, 128.05, 127.4, 124.0, 123.6, 107.9 (d, *J* = 5.5 Hz), 105.4 (d, *J* = 7.9 Hz), 105.3 (d, *J* = 5.4 Hz), 92.8 (d, *J* = 8.3 Hz), 89.9 (d, *J* = 6.9 Hz), 60.9, 38.8, 37.4, 35.8, 30.2, 29.6, 29.5, 29.4, 26.42, 26.39, 25.5, 25.3, 24.0, 10.5, 8.8 ppm; **IR** (ATR) ṽ = 2926 (m), 2851 (w), 1469 (w), 1449 (m), 1373 (w), 910 (m), 791 (w), 773 (w), 756 (m), 729 (s), 644 (w) cm^{-1}; **HRMS** (ESI/QTOF) *m/z* = calcd. for $[C_{32}H_{35}ClRh]^+$, $[(M/2)-Cl^-]^+$: 557.1477, found: 557.1486; $[\alpha]_D^{22}$ = -275.0 (*c* = 0.08, $CHCl_3$).

Rhodium complex (*R*)-**Rh26**

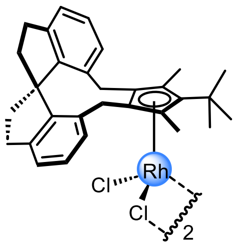

According to *General Procedure 4a*, starting from an isolated mixture of $Cp^{V}H$ isomers **Cp^{V}30** and spirodiene **S30** (88:12 ratio, 372 µmol of $Cp^{V}H$) at 80 °C for 20 h instead of 140 °C for 10 min, chiral Rh(III)-complex **Rh26** (57 mg, 101 µmol of monomer, 27% yield) was obtained as a dark red solid. When *General Procedure 4a* was followed without modifications, the complexation yield was much higher (83%) but partial cleavage of the *t*Bu-frontarm occurred, thus furnishing a mixture of **Rh26** and the tetrasubstituted complex (2:1 ratio). When *General Procedure 4b* was followed, using $[Rh(COD)OAc]_2$ as precursor (at 25 °C instead of 70 °C) and SO_2Cl_2 as oxidant, a lower yield of pure **Rh26** (8% over 2 steps) was obtained.

^{1}H NMR (600 MHz, *d*$_6$-DMSO) δ = 7.62 (d, *J* = 7.5 Hz, 1H), 7.20 – 7.14 (m, 2H), 7.11 – 7.04 (m, 2H), 6.90 (d, *J* = 7.3 Hz, 1H), 3.62 (d, *J* = 13.8 Hz, 1H), 3.33 (d, *J* = 13.8 Hz, 1H), 3.25 (d, *J* = 15.2 Hz, 1H), 3.03 – 2.92 (m, 2H), 2.85 – 2.78 (m, 2H), 2.60 (d, *J* = 15.2 Hz, 1H), 2.24 – 2.19 (m, 2H), 2.07 (s, 3H), 1.93 (s, 3H), 1.89 – 1.82 (m, 1H), 1.76 – 1.70 (m, 1H), 1.43 (s, 9H) ppm; **^{13}C{^{1}H} NMR** (151 MHz, *d*$_6$-DMSO) δ = 147.4, 146.6, 143.4, 142.0, 132.7, 130.8, 130.1, 128.0, 127.4, 124.0, 123.7, 110.1 (d, *J* = 6.0 Hz), 109.4 (d, *J* = 5.6 Hz), 106.3 (d, *J* = 6.1 Hz), 92.1 (d, *J* = 8.2 Hz), 88.2 (d, *J* = 6.8 Hz), 60.8, 38.8, 37.4, 34.1, 30.3, 29.6, 29.4, 25.3, 23.2, 12.9, 12.1 ppm; **IR** (ATR) ṽ = 2946 (m), 1469 (w), 1451 (w), 1430 (w), 1390 (w), 1368 (w), 1033 (w), 909 (m), 790 (w), 759 (w), 726 (s), 645 (w) cm^{-1}; **HRMS** (ESI/QTOF) *m/z* = calcd. for $[C_{30}H_{33}ClRh]^+$, $[(M/2)\text{-}Cl^-]^+$: 531.1320, found: 531.1326; $[\alpha]_D^{22}$ = -157.4 (*c* = 0.09, $CHCl_3$).

Rhodium complex (*R*)-**Rh27**

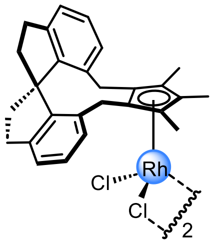

According to *General Procedure 4a*, starting from an isolated mixture of $Cp^{V}H$ isomers **Cp^{V}31** and spirodiene **S31** (99:1 ratio, 297 µmol of $Cp^{V}H$), chiral Rh(III)-complex **Rh27** (143 mg, 273 µmol of monomer, 92% yield) was obtained as a dark red solid.

^{1}H NMR (600 MHz, *d*$_6$-DMSO) δ = 7.73 (d, *J* = 7.5 Hz, 1H), 7.20 – 7.11 (m, 3H), 7.09 – 7.06 (m, 1H), 6.88 (d, *J* = 7.4 Hz, 1H), 3.52 (d, *J* = 13.6 Hz, 1H), 3.29 – 3.23 (m, 2H), 3.03 – 2.92 (m, 2H), 2.86 – 2.79 (m, 2H), 2.69 (d, *J* = 15.2 Hz, 1H), 2.25 – 2.19 (m, 2H), 1.92 – 1.84 (m, 1H), 1.79 – 1.70 (m, 7H), 1.66 (s, 3H) ppm; **^{13}C{^{1}H} NMR** (151 MHz, *d*$_6$-DMSO) δ = 147.3, 146.6, 143.4, 142.0, 133.1, 130.7, 130.3, 128.2, 128.1, 127.4, 124.0, 123.6, 108.4 (d, *J* = 6.9 Hz), 104.2 (d, *J* = 6.5 Hz), 101.7 (d, *J* = 6.6 Hz), 92.9 (d, *J* = 7.9 Hz), 89.2 (d, *J* = 7.7 Hz), 60.9, 38.9, 37.3, 29.5, 29.4, 25.7, 24.2, 9.2, 9.0, 8.4 ppm; **IR** (ATR) ṽ = 2943 (w), 2847 (w), 1474 (w), 1448 (m), 1376 (w), 1023 (w), 909 (m), 783 (m), 756 (m), 727 (s), 644 (w) cm^{-1}; **HRMS** (ESI/QTOF) *m/z* = calcd. for $[C_{27}H_{27}ClRh]^+$, $[(M/2)\text{-}Cl^-]^+$: 489.0851, found: 489.0856; $[\alpha]_D^{22}$ = -222.2 (*c* = 0.03, $CHCl_3$).

Rhodium complex (*R*)-**Rh28**

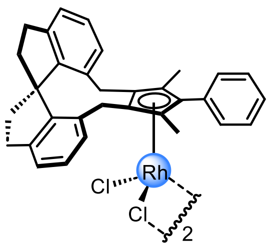

According to *General Procedure 4a*, starting from an isolated mixture of $Cp^{V}H$ isomers **Cp^{V}33** and spirodiene **S33** (92:8 ratio, 243 µmol of $Cp^{V}H$), chiral Rh(III)-complex **Rh28** (97 mg, 165 µmol of monomer, 68% yield) was obtained as a dark red solid.

^{1}H NMR (600 MHz, d_6-DMSO) δ = 7.62 – 7.59 (m, 2H), 7.56 – 7.54 (m, 1H), 7.47 – 7.42 (m, 3H), 7.23 – 7.19 (m, 2H), 7.14 – 7.08 (m, 2H), 7.06 – 7.04 (m, 1H), 3.70 (d, *J* = 13.6 Hz, 1H), 3.38 (d, *J* = 13.6 Hz, 1H), 3.32 (d, *J* = 15.1 Hz, 1H), 3.05 – 2.94 (m, 2H), 2.87 – 2.81 (m, 2H), 2.78 (d, *J* = 15.1 Hz, 1H), 2.26 – 2.20 (m, 2H), 1.90 (s, 3H), 1.89 – 1.84 (m, 1H), 1.81 – 1.75 (m, 1H), 1.74 (s, 3H) ppm; **$^{13}C\{^{1}H\}$ NMR** (151 MHz, d_6-DMSO) δ = 147.4, 146.5, 143.6, 141.9, 132.9, 130.8, 130.6, 129.9, 128.9, 128.5, 128.2, 128.1, 127.5, 124.1, 123.7, 108.1 (d, *J* = 5.1 Hz), 106.8 (d, *J* = 5.7 Hz), 100.9 (d, *J* = 6.8 Hz), 92.8 (d, *J* = 7.7 Hz), 91.6 (d, *J* = 7.6 Hz), 60.9, 38.7, 37.6, 29.6, 29.4, 25.9, 24.1, 10.0, 9.9 ppm; **IR** (ATR) ṽ = 2944 (w), 2847 (w), 1594 (w), 1446 (w), 1430 (w), 1389 (w), 1164 (w), 1076 (w), 1031 (w), 909 (m), 793 (w), 754 (m), 728 (s), 701 (m), 645 (w), 574 (w) cm^{-1}; **HRMS** (ESI/QTOF) *m/z* = calcd. for $[C_{32}H_{29}ClRh]^{+}$, $[(M/2)\text{-}Cl^{-}]^{+}$: 551.1007, found: 551.1023; $[\alpha]_D^{22}$ = -325.2 (*c* = 0.07, $CHCl_3$).

Rhodium complex (*R*)-**Rh29**

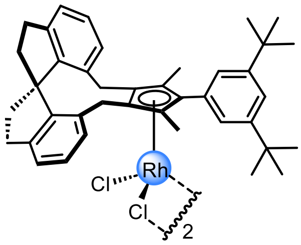

According to *General Procedure 4a*, starting from an isolated mixture of $Cp^{V}H$ isomers **Cp^{V}34** and spirodiene **S34** (98:2 ratio, 315 µmol of $Cp^{V}H$), chiral Rh(III)-complex **Rh29** (125 mg, 179 µmol of monomer, 57% yield) was obtained as a dark red solid.

^{1}H NMR (500 MHz, d_6-DMSO) δ = 7.56 – 7.50 (m, 3H), 7.45 – 7.43 (m, 1H), 7.23 – 7.20 (m, 2H), 7.14 – 7.09 (m, 2H), 7.09 – 7.06 (m, 1H), 3.73 (d, *J* = 13.7 Hz, 1H), 3.39 – 3.32 (m, 2H), 3.05 – 2.93 (m, 2H), 2.88 – 2.79 (m, 3H), 2.26 – 2.20 (m, 2H), 1.91 (s, 3H), 1.91 – 1.83 (m, 1H), 1.82 – 1.74 (m, 4H), 1.30 (s, 18H) ppm; **$^{13}C\{^{1}H\}$ NMR** (126 MHz, d_6-DMSO) δ = 149.9, 147.4, 146.4, 143.5, 141.8, 133.0, 130.8, 129.9, 128.7, 128.2, 127.5, 127.2, 125.3, 124.1, 123.6, 122.1, 111.9 (d, *J* = 6.5 Hz), 108.1 (d, *J* = 5.9 Hz), 106.2 (d, *J* = 6.8 Hz), 100.8 (d, *J* = 7.8 Hz), 93.0 (d, *J* = 7.7 Hz), 38.7, 37.6, 34.7, 31.2, 29.5, 29.4, 25.9, 24.3, 10.2, 10.0 ppm; **IR** (ATR) ṽ = 2952 (s), 2905 (w), 2866 (w), 1594 (w), 1473 (m), 1446 (m), 1393 (w), 1363 (w), 1249 (w), 909 (m), 880 (w), 790 (w), 757 (m), 731 (s), 644 (w) cm^{-1}; **HRMS** (ESI/QTOF) *m/z* = calcd. for $[C_{40}H_{45}ClRh]^{+}$, $[(M/2)\text{-}Cl^{-}]^{+}$: 663.2259, found: 663.2266; $[\alpha]_D^{22}$ = -248.2 (*c* = 0.09, $CHCl_3$).

Rhodium complex (*R*)-**Rh30**

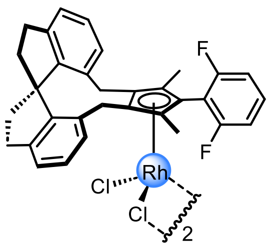

According to *General Procedure 4a*, starting from an isolated mixture of $Cp^{V}H$ isomers **Cp^{V}35** and spirodiene **S35** (88:12 ratio, 275 µmol of $Cp^{V}H$), chiral Rh(III)-complex **Rh30** (139 mg, 223 µmol of monomer, 81% yield) was obtained as a dark red solid.

^{1}H NMR (600 MHz, *d*$_6$-DMSO) δ = 7.67 – 7.57 (m, 2H), 7.25 – 7.18 (m, 4H), 7.16 – 7.08 (m, 2H), 7.02 – 6.98 (m, 1H), 3.67 (d, *J* = 13.5 Hz, 1H), 3.38 (d, *J* = 13.6 Hz, 1H), 3.33 (d, *J* = 15.1 Hz, 1H), 3.06 – 2.93 (m, 2H), 2.88 – 2.81 (m, 2H), 2.79 (d, *J* = 15.2 Hz, 1H), 2.27 – 2.21 (m, 2H), 1.92 – 1.86 (m, 1H), 1.84 (d, *J* = 3.6 Hz, 3H), 1.81 – 1.75 (m, 1H), 1.66 (d, *J* = 1.8 Hz, 3H) ppm; **^{13}C{^{1}H} NMR** (151 MHz, *d*$_6$-DMSO) δ = 160.1 (dd, *J* = 248.6, 5.7 Hz), 158.7 (dd, *J* = 256.5, 6.6 Hz), 147.5, 146.6, 143.7, 142.0, 133.0, 132.5 (t, *J* = 10.3 Hz), 130.6, 129.9, 128.13, 128.06, 127.6, 124.2, 123.8, 112.9 (d, *J* = 21.2 Hz), 111.6 (d, *J* = 21.7 Hz), 105.7 (t, *J* = 17.7 Hz), 92.5 (d, *J* = 5.8 Hz), 88.9, 61.0, 38.8, 37.5, 29.6, 29.4, 25.9, 24.4, 10.2 (d, *J* = 7.5 Hz), 9.9 ppm; **^{19}F{^{1}H} NMR** (376 MHz, *d*$_6$-DMSO) δ = -110.01 ppm; **IR** (ATR) ṽ = 2945 (w), 2848 (w), 1626 (m), 1587 (w), 1471 (m), 1449 (m), 1430 (w), 1385 (w), 1277 (w), 1238 (w), 1001 (m), 909 (m), 784 (m), 757 (m), 727 (s), 645 (w), 575 (w), 551 (w) cm^{-1}; **HRMS** (ESI/QTOF) *m/z* = calcd. for $[C_{32}H_{27}ClF_2Rh]^+$, $[(M/2)-Cl^-]^+$: 587.0819, found: 587.0838; $[\alpha]_D^{22}$ = -247.2 (*c* = 0.06, $CHCl_3$).

Rhodium complex (*S*$_a$)-**Rh32**

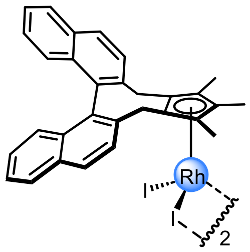

According to *General Procedure 4b*, starting from an isolated mixture of $Cp^{V}H$ isomers **Cp^{V}36** and spirodiene **S36** (63:37 ratio, 130 µmol of $Cp^{V}H$) and using $[Rh(COD)Cl]_2$ as precursor and I_2 as oxidant, chiral Rh(III)-complex **Rh32** (27 mg, 37 µmol of monomer, 28% yield over 2 steps) was obtained as a purple-black solid. Notably, the TMS-sidewalls of **Cp^{V}36** were cleaved off in the process (see **Section 5.8**), formally equaling complexation of a (*S*$_a$)-**Cp^{V}3** ligand.

^{1}H NMR (600 MHz, *d*$_6$-DMSO) δ = 8.06 (d, *J* = 8.4 Hz, 1H), 8.04 – 7.99 (m, 4H), 7.53 (d, *J* = 8.5 Hz, 1H), 7.52 – 7.48 (m, 1H), 7.48 – 7.45 (m, 1H), 7.34 – 7.29 (m, 1H), 7.27 – 7.22 (m, 1H), 7.01 (d, *J* = 8.5 Hz, 1H), 6.85 (d, *J* = 8.5 Hz, 1H), 3.82 (d, *J* = 16.5 Hz, 1H), 3.78 (d, *J* = 14.2 Hz, 1H), 3.56 (d, *J* = 14.1 Hz, 1H), 2.89 (d, *J* = 16.4 Hz, 1H), 2.04 (s, 3H), 2.03 (s, 3H), 1.95 (s, 3H) ppm; **^{13}C{^{1}H} NMR** (151 MHz, *d*$_6$-DMSO) δ = 135.5, 135.1, 134.4, 132.4, 132.28, 132.26, 131.5, 131.2, 131.1, 129.4, 128.3, 128.2, 128.0, 127.0, 126.8, 126.2, 126.1, 125.9, 125.8, 125.5, 107.1 (d, *J* = 5.5 Hz), 105.1 (d, *J* = 6.3 Hz), 102.4 (d, *J* = 6.2 Hz), 97.3 (d, *J* = 6.6 Hz), 93.8 (d, *J* = 6.8 Hz), 30.5, 30.3, 11.0, 10.4, 9.5 ppm; **IR** (ATR) ṽ = 2944 (w), 1593 (w), 1508 (w), 1452 (m), 1375 (w), 1362 (w), 1336 (w), 1248 (w), 1224 (w), 1145 (w), 1017 (w), 907 (m), 864 (w), 851 (w), 837 (w), 824 (w), 810 (m), 775 (w), 751 (m), 730 (s), 707 (w) cm^{-1}; **HRMS** (nanochip-ESI/LTQ-Orbitrap) *m/z* = calcd. for $[C_{30}H_{25}IRh]^+$, $[(M/2)-I^-]^+$: 615.0050, found: 615.0044; $[\alpha]_D^{22}$ = -420.3 (*c* = 0.05, $CHCl_3$).

5.4 Pentasubstituted Cp^V Iridium Complexes

General Procedure 5 – Iridium(III) Complexation of Chiral Cyclopentadienes.

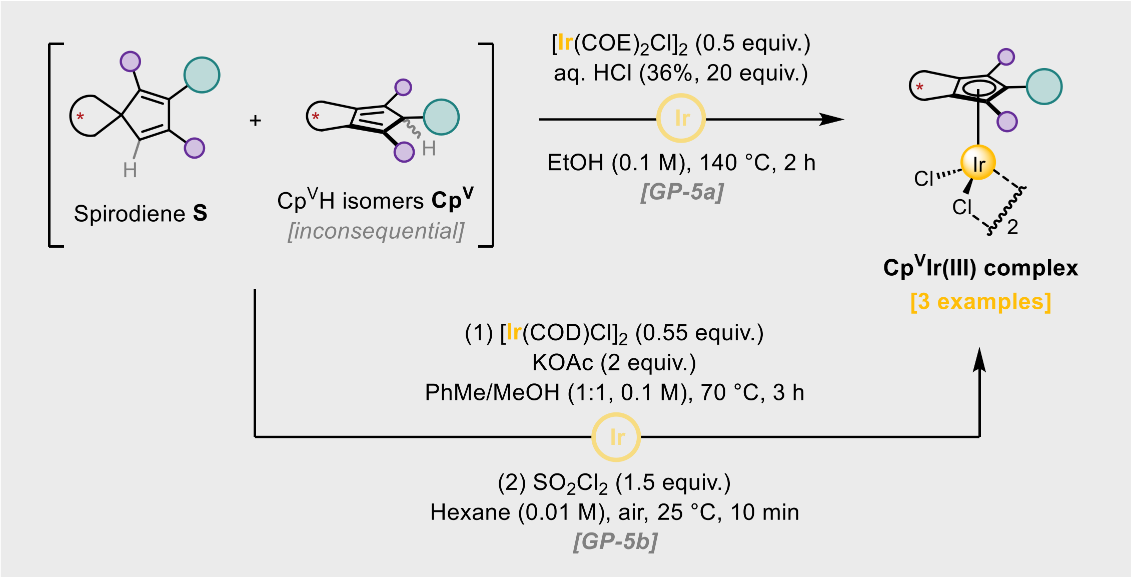

Scheme S19. Iridium(III) complexation of chiral cyclopentadienes towards [Cp^{V}IrCl$_2$]$_2$-type dimers.

***General Procedure 5a*: With [Ir(COE)$_2$Cl]$_2$ and HCl.**

An oven-dried microwave vial was charged with a mixture of pentasubstituted chiral cyclopentadiene isomers **Cp^V** and spirodiene **S** (1 equiv. of Cp^{V}H isomers, as determined by qNMR). In a nitrogen-filled glovebox, bis(cyclooctene) iridium(I) chloride dimer (0.5 equiv.) was added, and the vial was capped. Outside of the glovebox, the solids were suspended in ethanol (10 mL/mmol). Next, aqueous HCl (36%, 20 equiv.) was added dropwise, resulting in some precipitation, and the reaction mixture was stirred in a heating block at 140 °C for 2 hours. After cooling to room temperature (25 °C) and carefully releasing the built-up pressure, the obtained dark yellow suspension was first homogenized and transferred by addition of DCM, after which all volatiles were evaporated *in vacuo*. The brown residue was redissolved in DCM and filtered through a pad of celite (3 cm) and Na_2SO_4 (2 cm) with DCM as eluent. After evaporation of all volatiles *in vacuo*, the residue was redissolved in toluene and filtered through a pad of celite (3 cm) with toluene as eluent. A homogeneous orange solution eluted, whereas brown solids were retained on top. After evaporation of all volatiles *in vacuo*, the residue was purified by small-scale flash column chromatography on silica (wet loading with DCM, 5 cm column height, 0.5 cm diameter), eluting first with DCM (to remove impurities; usually a yellow fraction) and then with ethyl acetate (to collect the retained complex; a brown fraction). Removal of all volatiles *in vacuo* afforded the corresponding dimeric chiral [Cp^{V}IrCl$_2$]$_2$-type iridium(III) complex as an orange-brown solid.

***General Procedure 5b*: With [Ir(COD)Cl]$_2$ and SO_2Cl_2.**

Step 1. An oven-dried microwave vial was charged with a mixture of pentasubstituted chiral cyclopentadiene isomers **Cp^V** and spirodiene **S** (1 equiv. of Cp^{V}H isomers, as determined by qNMR) and potassium acetate (2 equiv.). In a nitrogen-filled glovebox, cyclooctadiene iridium(I) chloride dimer (0.55 equiv.) was added, and the vial was capped. Outside of the glovebox, the solids were dissolved by

sequentially adding anhydrous toluene (5 mL/mmol) and anhydrous methanol (5 mL/mmol). Next, the orange reaction mixture was stirred in a heating block at 70 °C for 3 hours, gradually turning yellow. After cooling to room temperature (25 °C), all volatiles were evaporated *in vacuo*. The residue was redissolved in toluene, and the resulting suspension was passed through a pad of silica gel (3 cm) with toluene as eluent to give a homogeneous light-yellow solution. After removal of all volatiles *in vacuo*, the residue was redissolved in hexane (50 mL/mmol) and immediately subjected to the oxidation step.

Step 2. At room temperature (25 °C) and under air, a solution of sulfuryl chloride (1.5 equiv.) in hexane (50 mL/mmol) was added to the light-yellow hexane solution of crude $Cp^{V}Ir(COD)$ complex under vigorous stirring, which resulted in an immediate color change to orange and precipitation. After 10 minutes of stirring, all volatiles were evaporated under reduced pressure. The residue was purified by small-scale flash column chromatography on silica (wet loading with DCM, 5 cm column height, 0.5 cm diameter), eluting first with DCM (to remove impurities; usually a yellow fraction) and then with ethyl acetate (to collect the retained complex; an orange fraction). Removal of all volatiles *in vacuo* afforded the corresponding dimeric chiral $[Cp^{V}IrCl_2]_2$-type iridium(III) complex as an orange-brown solid.

Note 1: For small-scale complexations, the **Cp^{V}/S** mixture foam could be transferred to the microwave vial as a THF solution, which was then evaporated *in vacuo* before adding the iridium precursor.

Note 2: The preferred solvent for the NMR characterization of $[Cp^{V}IrCl_2]_2$-type complexes was d_6-DMSO. It breaks up the dimers by forming monomeric adducts, resulting in more resolved spectra with substantially sharper signals, contrary to the often broad and unclear signals in CD_2Cl_2 or $CDCl_3$.

Iridium complex (R_a)-**Ir1**

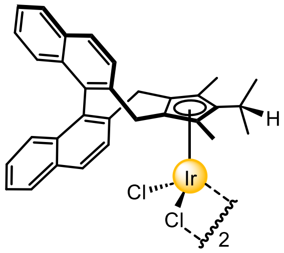

According to *General Procedure 5a*, starting from an isolated mixture of $Cp^{V}H$ isomers **$Cp^{V}1$** and spirodiene **S1** (56:44 ratio, 239 µmol of $Cp^{V}H$), chiral Ir(III)-complex **Ir1** (60 mg, 89 µmol of monomer, 37% yield) was obtained as an orange-brown solid. When *General Procedure 5b* was followed, a lower yield of **Ir1** (19% yield over 2 steps) was obtained.

^{1}H NMR (400 MHz, d_6-DMSO) δ = 8.08 (d, *J* = 8.4 Hz, 1H), 8.03 (d, *J* = 8.0 Hz, 1H), 8.01 – 7.96 (m, 2H), 7.70 (d, *J* = 8.5 Hz, 1H), 7.57 (d, *J* = 8.5 Hz, 1H), 7.52 – 7.42 (m, 2H), 7.34 – 7.29 (m, 1H), 7.26 – 7.21 (m, 1H), 7.00 (d, *J* = 8.5 Hz, 1H), 6.86 (d, *J* = 8.7 Hz, 1H), 3.49 – 3.42 (m, 2H), 3.08 (d, *J* = 14.0 Hz, 1H), 2.65 (d, *J* = 15.4 Hz, 1H), 2.62 – 2.51 (m, 1H), 1.91 (s, 3H), 1.89 (s, 3H), 1.25 – 1.21 (m, 6H) ppm; **^{13}C{^{1}H} NMR** (101 MHz, d_6-DMSO) δ = 135.1, 134.6, 133.8, 132.8, 132.32, 132.26, 131.5, 131.2, 130.6, 129.2, 128.2, 128.1, 127.7, 127.3, 126.7, 126.2, 126.0, 125.84, 125.79, 125.4, 101.9, 98.5, 95.4, 91.3, 82.1, 29.6, 27.1, 24.7, 20.5, 19.9, 8.8, 8.4 ppm; **IR** (ATR) ṽ = 3049 (w), 2965 (m), 2928 (w), 2872 (w), 1736 (w), 1508 (w), 1462 (w), 1446 (w), 1429 (w), 1371 (w), 1265 (w), 1243 (w), 1225 (w), 1058 (w), 1026 (w), 866 (w), 813 (s), 774 (w), 752 (s), 735 (m), 703 (w) cm^{-1}; **HRMS** (ESI/QTOF) *m/z* = calcd. for $[C_{32}H_{29}ClIr]^{+}$, $[(M/2)\text{-}Cl^{-}]^{+}$: 641.1582, found: 641.1586; $[\boldsymbol{\alpha}]_{\mathbf{D}}^{\mathbf{26}}$ = +41.7 (*c* = 0.06, $CHCl_3$).

Iridium complex (R_a)-**Ir2**

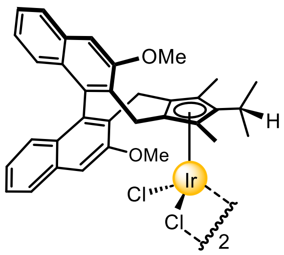

According to *General Procedure 5a*, starting from an isolated mixture of Cp^VH isomers **Cp^{V}10** and spirodiene **S10** (45:55 ratio, 32 µmol of Cp^VH), chiral Ir(III)-complex **Ir2** (14 mg, 19 µmol of monomer, 60% yield) was obtained as a dark brown solid.

^{1}H NMR (400 MHz, d_6-DMSO) δ = 7.92 (d, *J* = 8.2 Hz, 1H), 7.88 (d, *J* = 8.3 Hz, 1H), 7.53 (s, 1H), 7.45 – 7.41 (m, 1H), 7.40 – 7.37 (m, 1H), 7.36 (s, 1H), 7.14 – 7.09 (m, 1H), 7.07 – 7.02 (m, 1H), 6.86 (d, *J* = 8.5 Hz, 1H), 6.78 (d, *J* = 8.4 Hz, 1H), 4.01 (s, 3H), 3.91 (s, 3H), 3.88 (d, *J* = 15.4 Hz, 1H), 3.56 (d, *J* = 13.5 Hz, 1H), 2.93 (d, *J* = 13.6 Hz, 1H), 2.58 – 2.50 (m, 1H), 2.30 (d, *J* = 15.2 Hz, 1H), 1.93 (s, 3H), 1.81 (s, 3H), 1.22 (d, *J* = 7.1 Hz, 3H), 1.19 (d, *J* = 7.0 Hz, 3H) ppm; **^{13}C{^{1}H} NMR** (101 MHz, d_6-DMSO) δ = 156.8, 154.9, 136.51, 136.48, 133.6, 133.4, 127.1, 126.9, 126.6, 126.4, 126.3, 126.2, 126.1, 125.84, 125.78, 124.6, 124.1, 123.3, 106.1, 105.3, 103.2, 98.1, 93.6, 88.7, 84.1, 55.50, 55.45, 24.5, 22.0, 21.0, 20.9, 20.1, 9.12, 9.05 ppm; **IR** (ATR) ṽ = 2960 (w), 2926 (w), 1619 (w), 1597 (w), 1452 (w), 1423 (w), 1329 (w), 1295 (w), 1228 (w), 1197 (w), 1151 (w), 1111 (m), 1060 (w), 1019 (m), 909 (m), 822 (m), 771 (w), 729 (s), 645 (w) cm^{-1}; **HRMS** (ESI/QTOF) *m/z* = calcd. for $[C_{34}H_{33}ClIrO_2]^+$, $[(M/2)-Cl^-]^+$: 701.1793, found: 701.1783; $[\alpha]_D^{22}$ = -77.4 (*c* = 0.14, $CHCl_3$).

Iridium complex (*R*)-**Ir3**

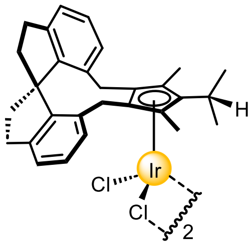

According to *General Procedure 5a*, starting from an isolated mixture of Cp^VH isomers **Cp^{V}28** and spirodiene **S28** (88:12 ratio, 178 µmol of Cp^VH), chiral Ir(III)-complex **Ir3** (63 mg, 98 µmol of monomer, 55% yield) was obtained as a light-brown solid.

^{1}H NMR (400 MHz, d_6-DMSO) δ = 7.43 (dd, *J* = 6.4, 2.4 Hz, 1H), 7.21 – 7.15 (m, 2H), 7.10 – 7.04 (m, 2H), 6.94 – 6.88 (m, 1H), 3.46 (d, *J* = 13.7 Hz, 1H), 3.21 – 3.11 (m, 2H), 3.04 – 2.89 (m, 2H), 2.89 – 2.74 (m, 2H), 2.69 (d, *J* = 14.8 Hz, 1H), 2.60 (hept, *J* = 7.2 Hz, 1H), 2.25 – 2.15 (m, 2H), 1.94 – 1.86 (m, 4H), 1.84 (s, 3H), 1.77 – 1.66 (m, 1H), 1.24 (d, *J* = 6.9 Hz, 3H), 1.20 (d, *J* = 7.2 Hz, 3H) ppm; **^{13}C{^{1}H} NMR** (101 MHz, d_6-DMSO) δ = 147.4, 146.5, 143.4, 141.8, 132.5, 131.3, 130.8, 128.0, 127.9, 127.3, 123.9, 123.4, 103.2, 100.9, 99.2, 84.2, 83.3, 60.9, 38.8, 37.6, 29.5, 29.4, 25.1, 24.8, 23.6, 20.6, 19.7, 9.4, 8.6 ppm; **IR** (ATR) ṽ = 2946 (m), 1469 (w), 1450 (m), 1431 (w), 1367 (w), 1033 (w), 910 (m), 790 (w), 756 (m), 730 (s) cm^{-1}; **HRMS** (ESI/QTOF) *m/z* = calcd. for $[C_{29}H_{31}ClIr]^+$, $[(M/2)-Cl^-]^+$: 607.1738, found: 607.1735; $[\alpha]_D^{26}$ = -136.2 (*c* = 0.08, $CHCl_3$).

5.5 Rh(III)-Mediated [1,5]-Alkyl Shift-Complexation Cascade

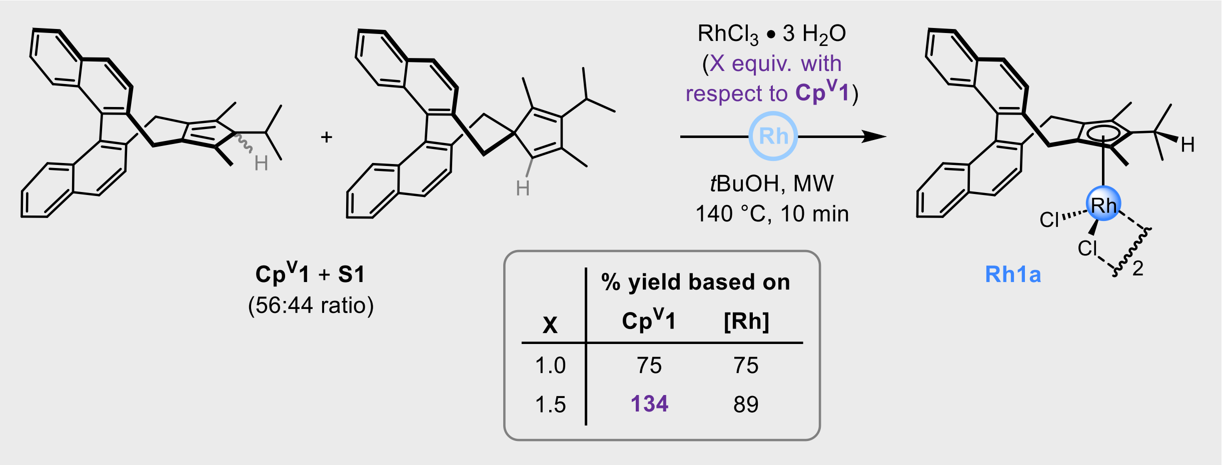

X	% yield based on CpV1	[Rh]
1.0	75	75
1.5	134	89

Scheme S20. Rhodium(III) complexation of **Cp^{V}1** with excess $RhCl_3 \cdot 3\ H_2O$ affording a seemingly impossible yield.

As previously described (see **Section 5.3**), according to *General Procedure 4a*, starting from an isolated mixture of Cp^{V}H isomers **Cp^{V}1** and spirodiene **S1** (56:44 ratio) and using $RhCl_3 \cdot 3\ H_2O$ (1 equiv. with respect to the content of **Cp^{V}1** as determined by qNMR), chiral rhodium(III) complex **Rh1a** was obtained in 75% yield based on the amount of **Cp^{V}1**. However, when an excess of $RhCl_3 \cdot 3\ H_2O$ precursor was used (1.5 equiv. with respect to **Cp^{V}1**), pure complex **Rh1a** was isolated in a seemingly impossible 134% yield based on the amount of **Cp^{V}1**, and 89% yield based on the amount of rhodium (**Scheme S20**).

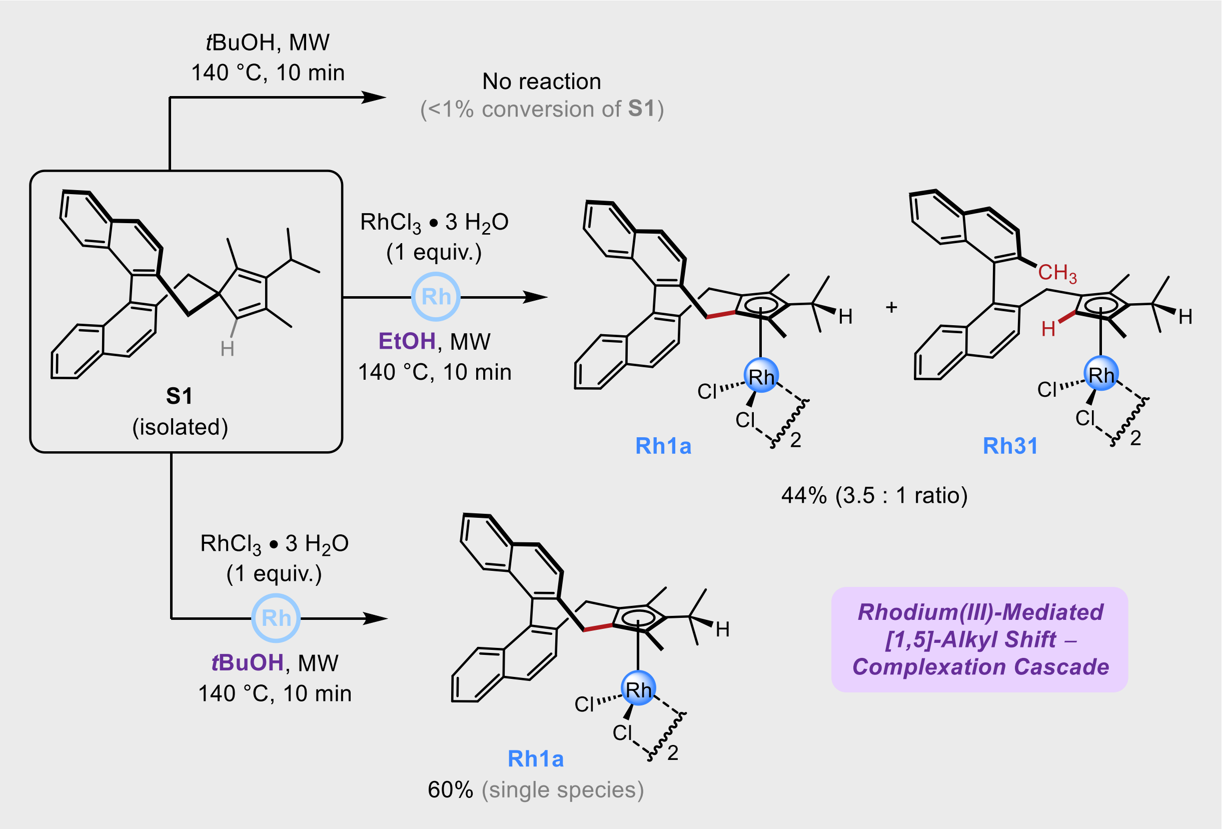

Scheme S21. Tandem Rh(III)-mediated rearrangement-complexation of spirodiene **S1** toward **Rh1a**.

To investigate the non-innocent behavior of spirodiene **S1** under the applied conditions, isolated **S1** (10.0 mg, 24.1 µmol) was separately subjected to $RhCl_3·3\ H_2O$ in *t*BuOH at 140 °C for 10 min, according to *General Procedure 4a* (**Scheme S21**). Intriguingly, **Rh1a** was formed and could be isolated in 60% yield (17.0 mg, 14.5 µmol), which suggests the occurrence of a unique rhodium(III)-mediated [1,5]-alkyl shift-complexation cascade. Control experiments confirmed that spirodiene **S1** is completely inert in the absence of rhodium trichloride and does not first rearrange at 140 °C to cyclopentadiene **Cp^{V}1**.

Furthermore, when the reaction was performed in ethanol as solvent instead of *tert*-butanol, an inseparable mixture of two Rh(III) complexes **Rh1a/Rh31** (3.5:1 ratio, 44% yield) was obtained. The NMR data of the minor tetrasubstituted complex **Rh31** is provided *in infra*. The HSQC spectrum clearly identified the characteristic methyl and Cp-ring ^{1}H and ^{13}C NMR signals of **Rh31** (δ_H = 0.84 and 5.11 ppm; δ_C = 8.8 and 82.8 ppm in CD_2Cl_2). The HRMS spectrum (**Figure S11**) was able to discriminate between the isotopic *m/z* signals of **Rh1a** and **Rh31**. When the solvent was switched to DCM, no reaction occurred and spirodiene **S1** was recovered.

<u>Rhodium complex **Rh31**</u>

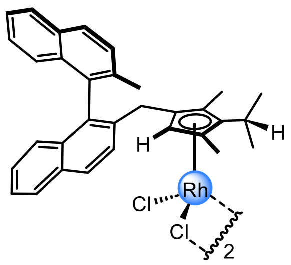

Dark red solid. Isolated as a **Rh1a**/**Rh31** mixture (3.5:1 ratio). **^{1}H NMR** (800 MHz, *d*$_6$-DMSO) δ = 8.11 (d, *J* = 8.3 Hz, 1H), 8.05 – 8.03 (m, 1H), 8.02 – 8.00 (m, 1H), 7.95 – 7.93 (m, 1H), 7.74 (d, *J* = 8.6 Hz, 1H), 7.63 (d, *J* = 8.5 Hz, 1H), 7.50 – 7.48 (m, 1H), 7.39 – 7.36 (m, 1H), 7.29 – 7.26 (m, 1H), 7.15 – 7.13 (m, 1H), 6.82 – 6.80 (m, 1H), 6.63 – 6.61 (m, 1H), 5.11 (s, 1H), 3.45 (d, *J* = 15.8 Hz, 1H), 3.14 (d, *J* = 15.8 Hz, 1H), 2.20 (hept, *J* = 7.2 Hz, 1H), 2.01 (s, 3H), 1.54 (s, 3H), 0.89 (d, *J* = 7.1 Hz, 3H), 0.87 (d, *J* = 7.1 Hz, 3H), 0.84 (s, 3H) ppm; **^{13}C{^{1}H} NMR** (151 MHz, *d*$_6$-DMSO) δ = 135.3, 134.1, 133.6, 133.3, 132.5, 132.1, 131.9, 131.7, 129.0, 128.8, 128.4, 128.1, 126.7, 125.9, 125.4, 125.3, 125.0, 107.3 (d, *J* = 5.6 Hz), 105.0 (d, *J* = 7.0 Hz), 101.0 (d, *J* = 6.2 Hz), 97.3 (d, *J* = 7.1 Hz), 82.8 (d, *J* = 7.2 Hz), 27.8, 24.4, 20.4, 20.0, 19.7, 11.0, 8.8 ppm.

HRMS (nanochip-based ESI/LTQ-Orbitrap) for **Rh1a**:

- *Main isotope:* calcd. for $[C_{32}H_{29}ClRh]^+$, $[(M/2)\text{-}Cl^-]^+$: *m/z* = 551.1008 (100%), found: 551.1003.
- *M+2 isotope:* calcd. for $[C_{32}H_{29}ClRh]^+$, $[(M/2)\text{-}Cl^-]^+$: *m/z* = 553.0978 (32%), found: 553.0976.

HRMS (nanochip-based ESI/LTQ-Orbitrap) for **Rh31**:

- *Main isotope:* calcd. for $[C_{32}H_{31}ClRh]^+$, $[(M/2)\text{-}Cl^-]^+$: *m/z* = 553.1164 (100%), found: 553.1155.

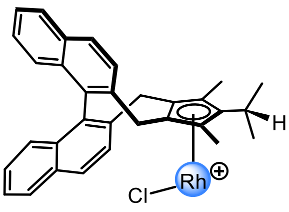

[(**Rh1a**/2)-Cl$^-$]$^+$
Calculated for $C_{32}H_{29}ClRh^+$
m/z: 551.1008 (100.0%), 552.1041 (34.6%), **553.0978** (32.0%), 554.1012 (11.1%), 553.1075 (5.8%), 555.1045 (1.9%)

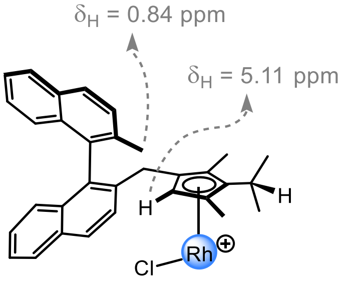

[(**Rh31**/2)-Cl$^-$]$^+$
Calculated for $C_{32}H_{31}ClRh^+$
m/z: **553.1164** (100.0%), 554.1198 (34.6%), 555.1135 (32.0%), 556.1168 (11.1%), 555.1231 (5.8%), 557.1202 (1.9%)

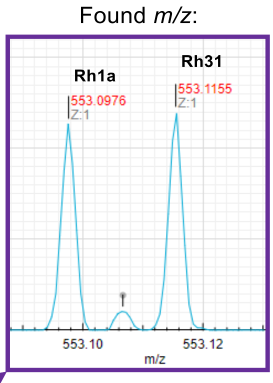

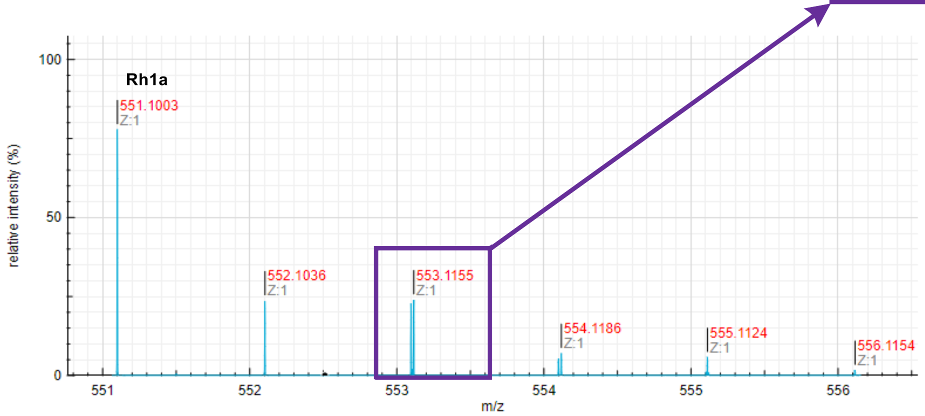

Figure S11. HRMS spectrum (nanochip-based ESI/LTQ-Orbitrap) of the **Rh1a**/**Rh31** mixture (3.5:1 ratio).

5.6 Cationic Cp^V Cobalt(III) and Rhodium(III) Complexes

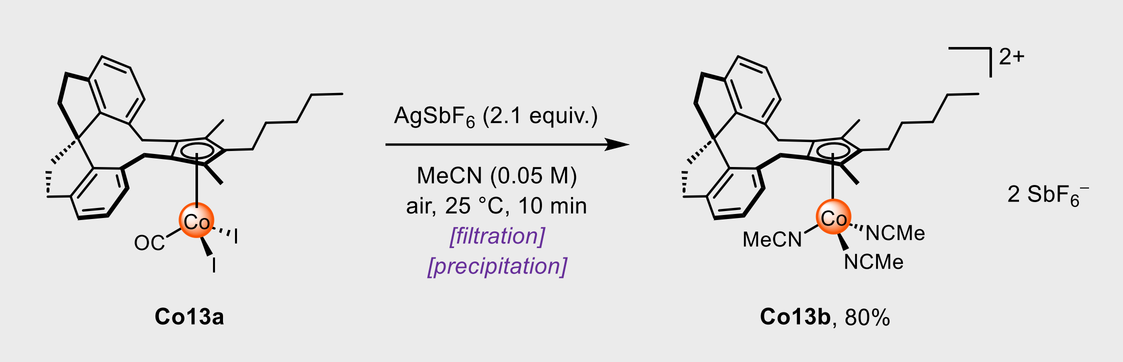

Scheme S22. Preparation of a bench-stable cationic [Cp^{V}Co(MeCN)$_3$](SbF$_6$)$_2$-type complex.

<u>Cobalt complex (*R*)-**Co13b**</u>

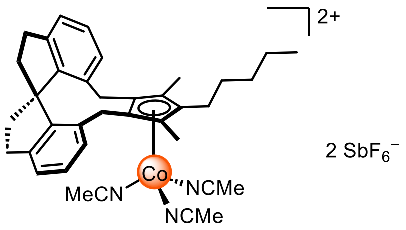

According to the following procedure (**Scheme S22**), cationic chiral Co(III)-complex **Co13b** was obtained as a bench-stable bordeaux-purple solid (**Figure S12**). The cobalt complex exists as a single 18-electron monomeric species.

Without protection from oxygen or moisture, an oven-dried microwave vial was charged with cobalt complex **Co13a** (51 mg, 68 µmol), which was then dissolved in anhydrous acetonitrile (10 mL/mmol). Next, a solution of silver(I) hexafluoroantimonate (49 mg, 143 µmol, 2.1 equiv.) in anhydrous acetonitrile (10 mL/mmol) was added dropwise to the black-purple solution. Immediately, fine bubbles appeared (i.e. evolution of CO gas) and within 30 seconds the reaction mixture turned bordeaux-purple, containing a fine off-white precipitate (i.e. AgI). After stirring for 10 minutes at room temperature (25 °C) under air, the suspension was filtered through a pad of celite (3 cm) with acetonitrile as eluent. After evaporation of all volatiles *in vacuo*, the residue was redissolved in DCM and again filtered through a pad of celite (3 cm) with DCM as eluent. After removal of all volatiles *in vacuo*, the residue was redissolved in acetonitrile and filtered for a third time through a pad of celite (3 cm) with acetonitrile as eluent to remove the final observed precipitates. After removal of all volatiles *in vacuo*, the residue was redissolved once more in minimal acetonitrile (*ca*. 0.5 mL). Subsequent addition of Et$_2$O (*ca.* 20 mL) afforded a light-red suspension, which was filtered on celite (2 cm). The retained solids were washed with Et$_2$O, and then collected with DCM. Removal of all volatiles *in vacuo* provided cationic Co(III)-complex **Co13b** (58 mg, 54 µmol, 80% yield) as a bordeaux-purple solid.

^{1}H NMR (400 MHz, CD$_2$Cl$_2$) δ = 7.44 – 7.36 (m, 3H), 7.25 (d, *J* = 7.4 Hz, 1H), 7.14 (t, *J* = 7.5 Hz, 1H), 6.70 (d, *J* = 7.6 Hz, 1H), 3.57 (d, *J* = 13.8 Hz, 1H), 3.42 – 3.33 (m, 2H), 3.16 – 2.97 (m, 3H), 2.96 – 2.87 (m, 1H), 2.42 (s, 9H), 2.41 – 2.32 (m, 3H), 2.31 – 2.21 (m, 1H), 2.07 – 1.93 (m, 3H), 1.59 (s, 3H), 1.55 (s, 3H), 1.44 – 1.28 (m, 6H), 0.90 (t, *J* = 6.8 Hz, 3H) ppm; **^{13}C{^{1}H} NMR** (151 MHz, CD$_2$Cl$_2$) δ = 148.9, 147.9, 145.3, 144.8, 132.4, 131.7, 129.3, 128.9, 128.49, 128.47, 127.0, 125.9, 125.8, 113.5, 112.0, 99.8, 94.4, 90.3, 62.1, 39.2, 38.5, 32.1, 30.5, 30.3, 26.7, 26.5, 26.0, 24.7, 22.7, 13.9, 11.3, 10.3, 4.6 ppm; **IR** (ATR) ṽ = 2941 (w), 2859 (w), 2328 (w), 2301 (w), 1452 (m), 1378 (w), 1268 (w), 1039 (w), 789 (w), 761 (w), 735 (m), 703 (w), 655

(s), 573 (w), 557 (w) cm^{-1}; **HRMS** (ESI/QTOF) *m/z* = calcd. for $[C_{33}H_{38}CoF_6NSb]^+$, $[M\text{-}2MeCN\text{-}SbF_6^-]^+$: 742.1273, found: 742.1278; $[\alpha]_D^{22}$ = -156.7 (*c* = 0.05, $CHCl_3$).

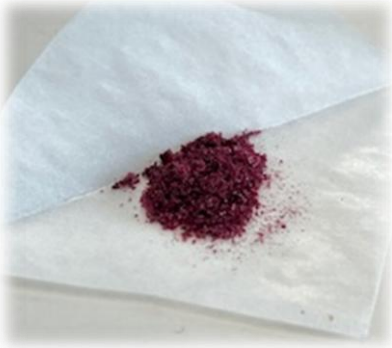

Figure S12. Isolation of cationic chiral Cp^{V}Co(III) complex (*R*)-**Co13b** as a bench-stable purple solid.

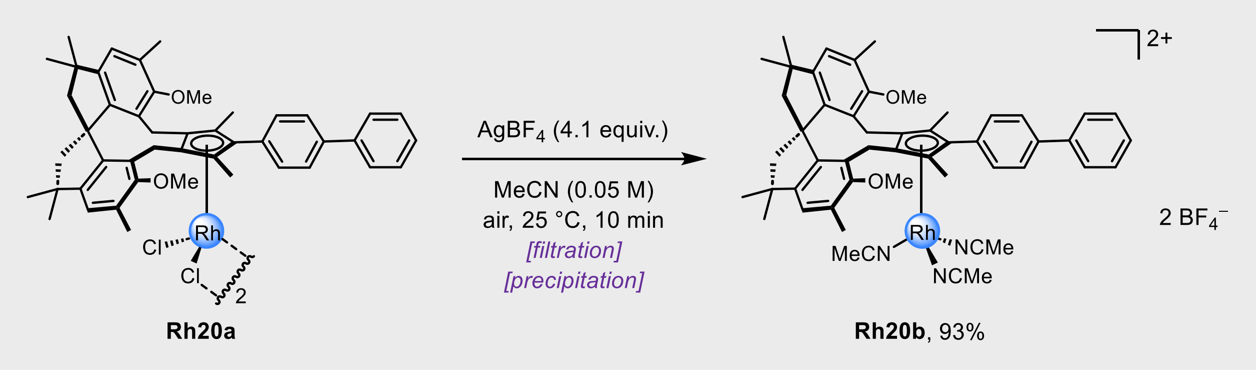

Scheme S23. Preparation of a cationic $[Cp^VRh(MeCN)_3](BF_4)_2$-type complex.

Rhodium complex (*R*)-**Rh20b**

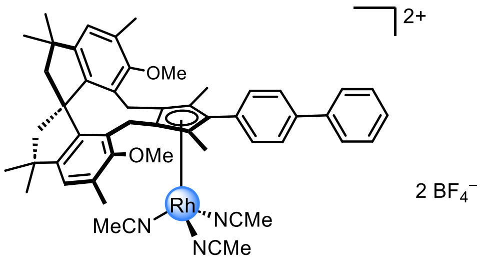

According to the following procedure (**Scheme S23**), cationic Rh(III)-complex **Rh20b** was obtained as a yellow solid. Notably, the acetonitrile ligands' ^{1}H NMR signal overlapped with the water signal, and the Cp-ring's ^{13}C NMR signals were not resolved. A suitable crystal for X-ray analysis (**Figure S13**) was obtained by slow liquid diffusion (*ca.* one month) of Et_2O into a concentrated solution in MeCN.

Without protection from oxygen or moisture, an oven-dried microwave vial was charged with rhodium complex **Rh20a** (28 mg, 35 µmol of monomer), which was then dissolved in anhydrous acetonitrile (10 mL/mmol). Next, a solution of silver(I) tetrafluoroborate (14 mg, 72 µmol, 4.1 equiv.) in anhydrous acetonitrile (10 mL/mmol) was added dropwise to the orange solution. Within 30 seconds, the reaction mixture turned light-yellow, containing a fine off-white precipitate (i.e. AgCl). After stirring for 10 minutes at room temperature (25 °C) under air, the suspension was filtered through a pad of celite (3 cm) with acetonitrile as eluent. After evaporation of all volatiles *in vacuo*, the residue was redissolved in DCM and again filtered through a pad of celite (3 cm) with DCM as eluent to remove the gray precipitates, providing a homogeneous yellow solution. After removal of all volatiles *in vacuo*, the

residue was redissolved in minimal acetonitrile (*ca*. 0.5 mL). Subsequent addition of Et_2O (*ca.* 20 mL) afforded a yellow suspension, which was filtered on celite (2 cm). The retained solids were washed with Et_2O, and then collected with DCM. Removal of all volatiles *in vacuo* provided cationic Rh(III)-complex **Rh20b** (34 mg, 33 µmol, 93% yield) as a yellow solid.

^{1}H NMR (400 MHz, CD_2Cl_2) δ = 7.82 – 7.78 (m, 2H), 7.73 – 7.69 (m, 2H), 7.61 – 7.57 (m, 2H), 7.55 – 7.50 (m, 2H), 7.48 – 7.43 (m, 1H), 7.12 (s, 1H), 7.05 (s, 1H), 3.98 (s, 3H), 3.84 – 3.78 (m, 4H), 3.41 (d, *J* = 14.0 Hz, 1H), 3.34 (d, *J* = 14.4 Hz, 1H), 2.67 (d, *J* = 14.4 Hz, 1H), 2.53 – 2.45 (m, 2H), 2.40 (s, 3H), 2.30 (s, 3H), 2.19 (d, *J* = 13.4 Hz, 1H), 2.01 (d, *J* = 13.2 Hz, 1H), 1.98 (s, 3H), 1.76 (s, 3H), 1.48 (s, 3H), 1.43 (s, 3H), 1.42 (s, 3H), 1.31 (s, 3H) ppm; **^{13}C{^{1}H} NMR** (101 MHz, CD_2Cl_2) δ = 157.4, 148.3, 147.6, 146.4, 143.6, 140.1, 132.4, 132.2, 130.6, 129.2, 128.3, 128.2, 127.4, 126.2, 125.9, 124.5, 124.4, 120.4, 120.2, 62.0, 60.9, 59.6, 58.5, 57.5, 42.2, 41.9, 32.1, 31.5, 31.1, 30.9, 21.5, 20.0, 16.63, 16.58, 11.5, 10.2, 2.7 ppm; **^{19}F{^{1}H} NMR** (376 MHz, CD_2Cl_2) δ = -151.84, -151.90 ppm; **IR** (ATR) ṽ = 2950 (w), 1629 (w), 1463 (w), 1022 (s), 919 (w), 767 (w), 520 (w) cm^{-1}; **HRMS** (nanochip-ESI/LTQ-Orbitrap) *m/z* = calcd. for $[C_{48}H_{52}NO_2Rh]^{2+}$, [M-2MeCN-$2BF_4^-]^{2+}$: 388.6521, found: 388.6531; $[\alpha]_D^{22}$ = -183.3 (*c* = 0.01, $CHCl_3$); **XRD** (Cu*Kα*, R_1 = 2.57%) CCDC: 2497532.

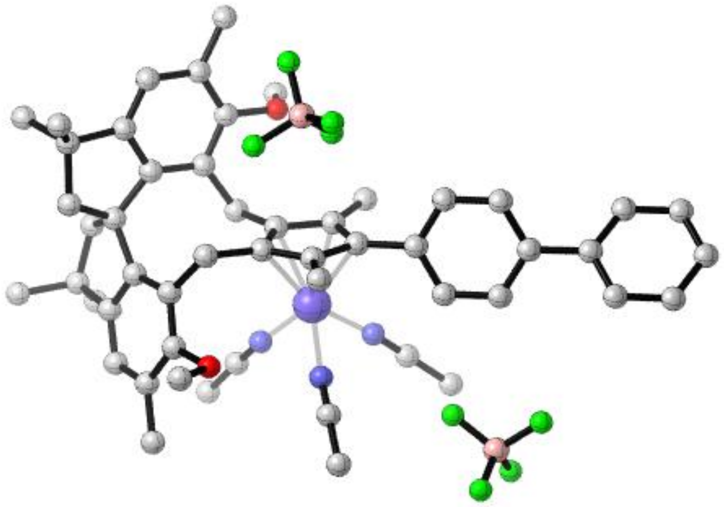

Figure S13. Solid-state X-ray structure of (*R*)-**Rh20b** (CCDC: 2497532) showing 50% probability thermal ellipsoids. Hydrogen atoms are omitted for clarity.

5.7 Cp^V Cobalt(III) and Rhodium(III) Phosphite Adducts

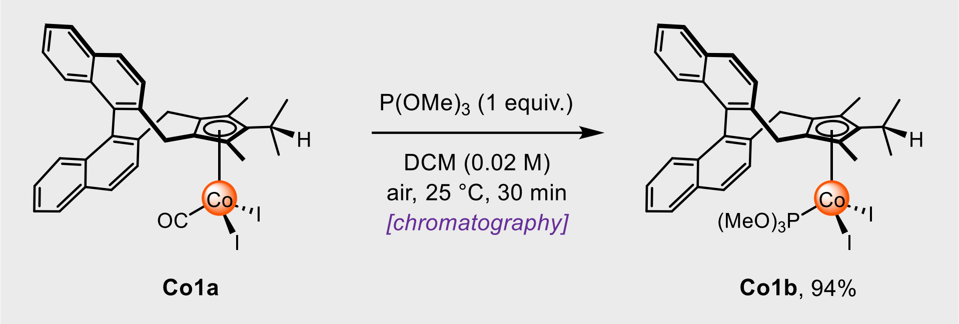

Scheme S24. Preparation of a Cp^{V}CoI$_2$P(OMe)$_3$-type complex amenable to chiral HPLC anaylsis.

<u>Cobalt complex (R_a)-**Co1b**</u>

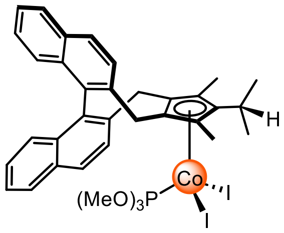

According to the following procedure (**Scheme S24**), chiral Co(III)-complex **Co1b** was obtained as a purple solid. The cobalt complex exists as a single 18-electron monomeric species. Notably, this phosphite adduct (−)-(R_a)-**Co1b** displayed an increased stability on silica gel, and its enantiopurity could be confirmed by chiral HPLC analysis (unlike for CO-bearing **Co1a**, which decomposed during HPLC).

Without protection from oxygen or moisture, an oven-dried microwave vial was charged with chiral Cp^{V}Co(CO)I$_2$-type cobalt complex **Co1a** (9.3 mg, 12.3 µmol), which was then dissolved in anhydrous DCM (50 mL/mmol). A trimethyl phosphite solution (0.10 M in DCM, 1 equiv.) was added, and the dark purple solution was stirred for 30 minutes at room temperature (25 °C) under air. Next, the reaction mixture was filtered through a pad of silica gel (1 cm) with DCM as eluent. After evaporation of all volatiles *in vacuo*, the residue was purified by small-scale flash column chromatography on silica (wet loading with pentane/DCM = 4:1, 5 cm column height, 0.5 cm diameter), eluting first with pentane/DCM = 4:1 (to remove impurities) and then with Et$_2$O/DCM = 1:4 (to collect the retained complex; a purple fraction). Removal of all volatiles *in vacuo* provided phosphite adduct **Co1b** (9.8 mg, 11.5 µmol, 94% yield, >99.9:0.1 er) as a purple solid.

^{1}H NMR (400 MHz, CD$_2$Cl$_2$) δ = 8.00 – 7.94 (m, 3H), 7.91 – 7.87 (m, 2H), 7.46 – 7.40 (m, 2H), 7.26 (d, J = 8.6 Hz, 1H), 7.24 – 7.16 (m, 2H), 7.10 – 7.07 (m, 1H), 6.98 (d, J = 8.2 Hz, 1H), 3.71 (dd, J = 16.7, 1.4 Hz, 1H), 3.66 (dd, J = 13.8, 4.9 Hz, 1H), 3.46 (d, J = 9.9 Hz, 9H), 3.36 (d, J = 13.9 Hz, 1H), 3.22 (hept, J = 7.2 Hz, 1H), 2.75 (dd, J = 16.7, 2.6 Hz, 1H), 2.28 (d, J = 5.7 Hz, 3H), 1.85 (d, J = 1.2 Hz, 3H), 1.61 (d, J = 6.9 Hz, 3H), 1.05 (d, J = 7.0 Hz, 3H) ppm; **^{13}C{^{1}H} NMR** (101 MHz, CD$_2$Cl$_2$) δ = 136.7 (d, J = 3.4 Hz), 136.3, 135.1, 134.2, 133.2, 132.9, 132.7, 132.6, 132.3, 129.5, 128.4, 128.2, 127.6, 127.2, 126.8 (d, J = 2.3 Hz), 126.6, 126.0 (d, J = 2.4 Hz), 125.8, 113.2 (d, J = 7.7 Hz), 101.4 (d, J = 9.9 Hz), 99.5 (d, J = 3.1 Hz), 94.4 (d, J = 2.5 Hz), 85.3 (d, J = 1.3 Hz), 56.9 (d, J = 10.2 Hz), 31.7, 30.1, 28.1 (d, J = 1.9 Hz), 22.4 (d, J = 5.6 Hz), 22.1, 13.2 (d, J = 2.9 Hz), 10.0 ppm; **^{31}P{^{1}H} NMR** (162 MHz, CD$_2$Cl$_2$) δ = 130.28 ppm; **IR** (ATR) ṽ = 3052 (w), 2946 (w), 2925 (w), 2847 (w), 1507 (w), 1445 (w), 1417 (w), 1382 (w), 1363 (w), 1177 (w), 1039 (s), 1017 (s), 952 (w),

814 (w), 794 (w), 773 (m), 752 (m), 733 (m), 709 (m), 677 (w), 566 (w), 532 (w) cm^{-1}; **HRMS** (ESI/QTOF) *m/z* = calcd. for $[C_{35}H_{38}CoIO_3P]^+$, $[M\text{-}I^-]^+$: 723.0930, found: 723.0924; $[\boldsymbol{\alpha}]_{\mathbf{D}}^{\mathbf{22}}$ = -405.6 (*c* = 0.06, CH_2Cl_2); **Chiral HPLC** (Chiralpak ID, 4.6 x 150 mm, 3 µm, Hexane/*i*PrOH 90:10, 1.0 mL/min, 35 °C, 254 nm) t_r (minor) = 8.40 min, t_r (major) = 11.03 min, >99.9:0.1 er.

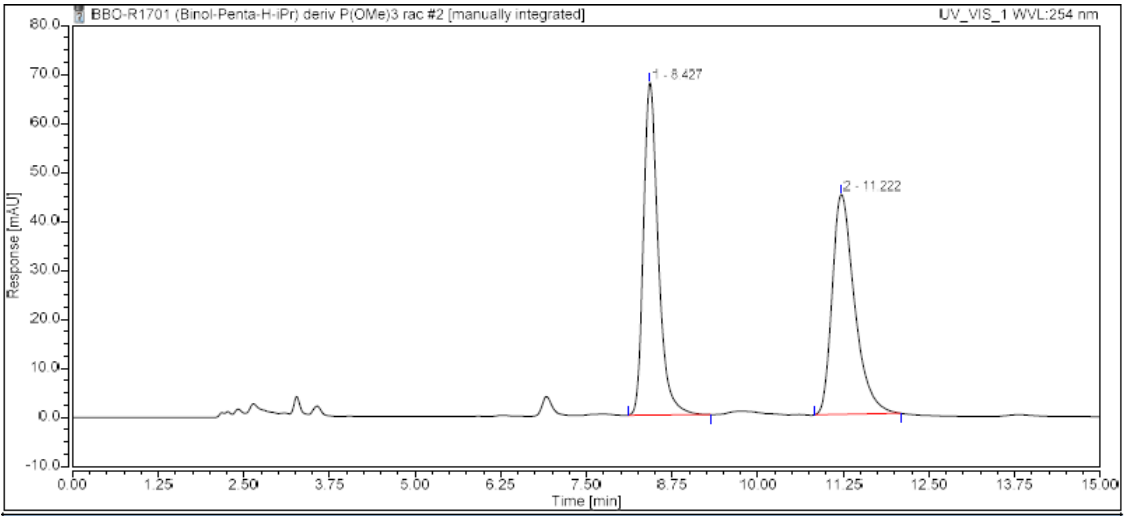

Integration Results

No.	Peak Name	Retention Time min	Area mAU*min	Height mAU	Relative Area %	Relative Height %	Amount n.a.
1		8.427	16.982	67.880	49.91	60.17	n.a.
2		11.222	17.046	44.928	50.09	39.83	n.a.

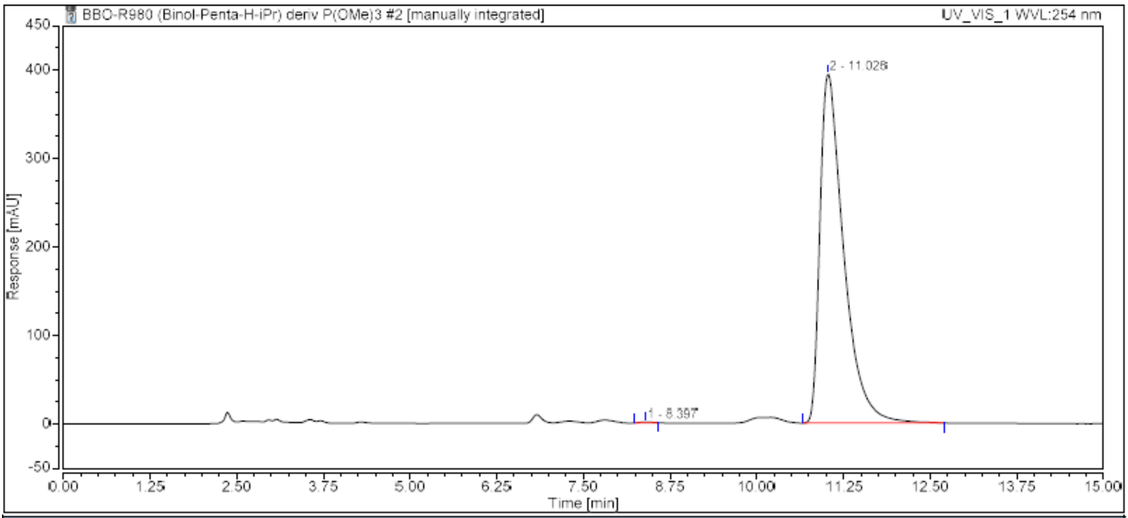

Integration Results

No.	Peak Name	Retention Time min	Area mAU*min	Height mAU	Relative Area %	Relative Height %	Amount n.a.
1		8.397	0.153	0.778	0.10	0.20	n.a.
2		11.028	156.069	394.230	99.90	99.80	n.a.
**Total:**			**156.222**	**395.008**	**100.00**	**100.00**	

***General Procedure 6* – In-situ Conversion of [$Cp^V RhCl_2$]$_2$ Complexes into Phosphite Adducts.**

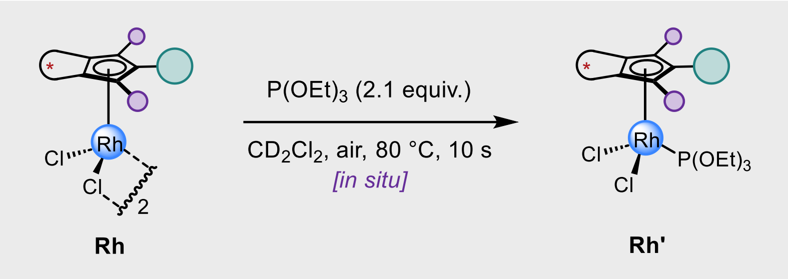

Scheme S25. In-situ conversion of chiral [$Cp^V RhCl_2$]$_2$ complexes into monomeric phosphite adducts.

Without protection from oxygen or moisture, a screw-cap vial was charged with an analytical sample of dimeric chiral [$Cp^V RhCl_2$]$_2$-type rhodium complex **Rh** (*ca.* 2 mg, 1.0 equiv.), which was then dissolved in deuterated DCM (0.5 mL). Next, a triethyl phosphite solution (0.13 M in CD_2Cl_2, 2.1 equiv.) was added and the vial was closed tightly under air. The orange-red solution was shortly heated (*ca.* 10 seconds) at 80 °C to form the monomeric $Cp^V RhCl_2P(OEt)_3$ adduct **Rh'** in situ. After cooling to room temperature (25 °C), the ^{31}P NMR spectrum was recorded immediately. If desired, purification could be performed by flash column chromatography on silica gel (wet loading with DCM, 5 cm column height, isocratic: DCM *then* DCM/Et_2O = 1:1), affording the phosphite adduct **Rh'** as a red solid.

Rhodium phosphite adduct (R_a)-**Rh^{tri}1'**

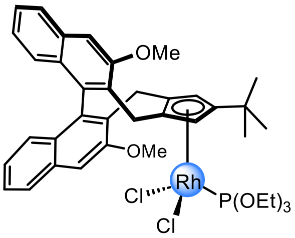

According to *General Procedure 6*, starting from an analytical sample of dimeric Cp^VRh(III) complex **Rh^{tri}1** (1.3 mg), phosphite adduct **Rh^{tri}1'** was formed in situ.

^{31}P{^{1}H} NMR (162 MHz, CD_2Cl_2) δ = 107.53 (d, *J* = 211.8 Hz) ppm; **HRMS** (ESI/QTOF) *m/z* = calcd. for [$C_{39}H_{46}ClO_5PRh$]$^+$, [M-Cl$^-$]$^+$: 763.1821, found: 763.1831.

Rhodium phosphite adduct (R_a)-**Rh^{tri}2'**

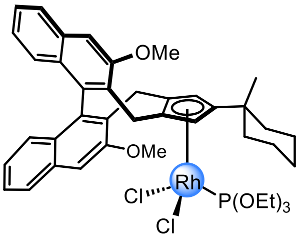

According to *General Procedure 6*, starting from an analytical sample of dimeric Cp^VRh(III) complex **Rh^{tri}2''** (1.6 mg), phosphite adduct **Rh^{tri}2'** was formed in situ.

^{31}P{^{1}H} NMR (162 MHz, CD_2Cl_2) δ = 107.92 (d, *J* = 211.9 Hz) ppm; **HRMS** (ESI/QTOF) *m/z* = calcd. for [$C_{42}H_{50}ClO_5PRh$]$^+$, [M-Cl$^-$]$^+$: 803.2134, found: 803.2130.

Rhodium phosphite adduct (R_a)-**Rh1a'**

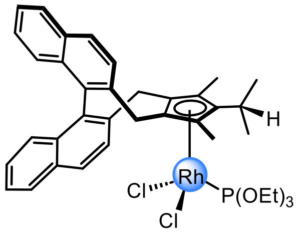

According to *General Procedure 6*, starting from an analytical sample of dimeric Cp^VRh(III) complex **Rh1a** (1.7 mg), phosphite adduct **Rh1a'** was formed in situ.

^{31}P{^{1}H} NMR (162 MHz, CD_2Cl_2) δ = 109.94 (d, *J* = 216.2 Hz) ppm; **HRMS** (ESI/QTOF) *m/z* = calcd. for $[C_{38}H_{44}ClO_3PRh]^+$, $[M-Cl^-]^+$: 717.1766, found: 717.1771.

Rhodium phosphite adduct (R_a)-**Rh4'**

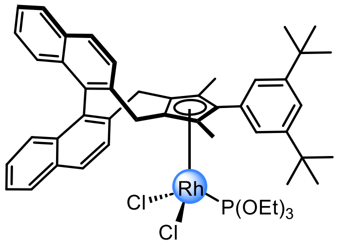

According to *General Procedure 6*, starting from an analytical sample of dimeric Cp^VRh(III) complex **Rh4** (1.6 mg), phosphite adduct **Rh4'** was formed in situ.

^{31}P{^{1}H} NMR (162 MHz, CD_2Cl_2) δ = 110.82 (d, *J* = 214.6 Hz) ppm; **HRMS** (ESI/QTOF) *m/z* = calcd. for $[C_{49}H_{58}ClO_3PRh]^+$, $[M-Cl^-]^+$: 863.2862, found: 863.2860.

Rhodium phosphite adduct (R_a)-**Rh5'**

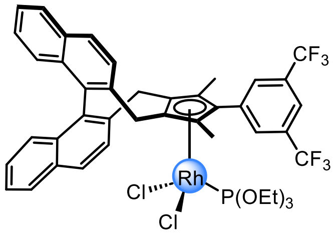

According to *General Procedure 6*, starting from an analytical sample of dimeric Cp^VRh(III) complex **Rh5** (3.1 mg), phosphite adduct **Rh5'** was formed in situ.

^{31}P{^{1}H} NMR (162 MHz, CD_2Cl_2) δ = 106.17 (d, *J* = 211.7 Hz) ppm; **HRMS** (ESI/QTOF) *m/z* = calcd. for $[C_{43}H_{40}ClF_6O_3PRh]^+$, $[M-Cl^-]^+$: 887.1357, found: 887.1352.

Rhodium phosphite adduct (R_a)-**Rh6'**

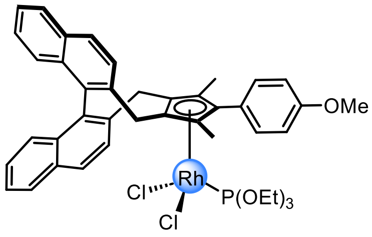

According to *General Procedure 6*, starting from an analytical sample of dimeric Cp^VRh(III) complex **Rh6** (2.3 mg), phosphite adduct **Rh6'** was formed in situ.

^{31}P{^{1}H} NMR (162 MHz, CD_2Cl_2) δ = 109.75 (d, *J* = 215.0 Hz) ppm; **HRMS** (ESI/QTOF) *m/z* = calcd. for $[C_{42}H_{44}ClO_4PRh]^+$, $[M-Cl^-]^+$: 781.1715, found: 781.1723.

Rhodium phosphite adduct (R_a)-**Rh7'**

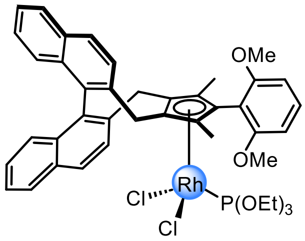

According to *General Procedure 6*, starting from an analytical sample of dimeric $Cp^VRh(III)$ complex **Rh7** (1.3 mg), phosphite adduct **Rh7'** was formed in situ.

$^{31}P\{^1H\}$ NMR (162 MHz, CD_2Cl_2) δ = 110.20 (d, *J* = 218.4 Hz) ppm; **HRMS** (ESI/QTOF) *m/z* = calcd. for $[C_{43}H_{46}ClO_5PRh]^+$, $[M\text{-}Cl^-]^+$: 811.1821, found: 811.1821.

Rhodium phosphite adduct (R_a)-**Rh8a'**

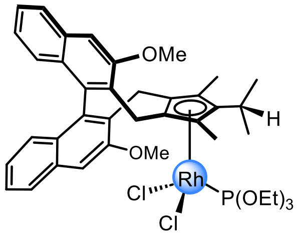

According to *General Procedure 6*, starting from an analytical sample of dimeric $Cp^VRh(III)$ complex **Rh8a** (1.4 mg), phosphite adduct **Rh8a'** was formed in situ.

$^{31}P\{^1H\}$ NMR (162 MHz, CD_2Cl_2) δ = 111.78 (d, *J* = 216.7 Hz) ppm; **HRMS** (ESI/QTOF) *m/z* = calcd. for $[C_{40}H_{48}ClO_5PRh]^+$, $[M\text{-}Cl^-]^+$: 777.1977, found: 777.1971.

Rhodium phosphite adduct (S_a)-**Rh13'**

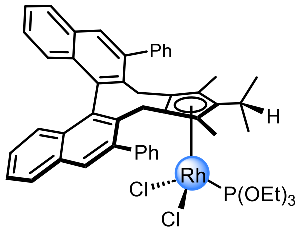

According to *General Procedure 6*, starting from an analytical sample of dimeric $Cp^VRh(III)$ complex **Rh13** (1.0 mg), phosphite adduct **Rh13'** was formed in situ.

$^{31}P\{^1H\}$ NMR (162 MHz, CD_2Cl_2) δ = 107.89 (d, *J* = 213.1 Hz) ppm; **HRMS** (ESI/QTOF) *m/z* = calcd. for $[C_{50}H_{52}ClO_3PRh]^+$, $[M\text{-}Cl^-]^+$: 869.2392, found: 869.2396.

Rhodium phosphite adduct (R)-**Rh16'**

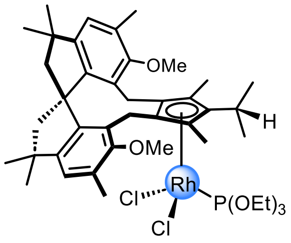

According to *General Procedure 6*, starting from an analytical sample of dimeric $Cp^VRh(III)$ complex **Rh16** (1.7 mg), phosphite adduct **Rh16'** was formed in situ.

$^{31}P\{^1H\}$ NMR (162 MHz, CD_2Cl_2) δ = 107.10 (d, *J* = 217.1 Hz) ppm; **HRMS** (ESI/QTOF) *m/z* = calcd. for $[C_{43}H_{62}ClO_5PRh]^+$, $[M\text{-}Cl^-]^+$: 827.3073, found: 827.3076.

Rhodium phosphite adduct (*R*)-**Rh17’**

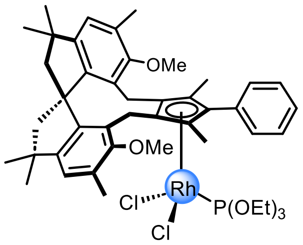

According to *General Procedure 6*, starting from an analytical sample of dimeric Cp^{V}Rh(III) complex **Rh17** (1.1 mg), phosphite adduct **Rh17’** was formed in situ.

^{31}P{^{1}H} NMR (162 MHz, CD_2Cl_2) δ = 104.40 (d, *J* = 213.1 Hz) ppm; **HRMS** (ESI/QTOF) *m/z* = calcd. for $[C_{46}H_{60}ClO_5PRh]^+$, $[M\text{-}Cl^-]^+$: 861.2916, found: 861.2917.

Rhodium phosphite adduct (*R*)-**Rh18’**

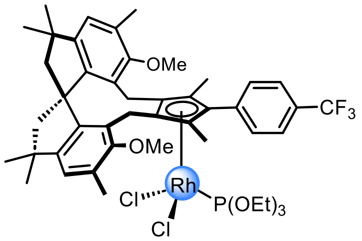

According to *General Procedure 6*, starting from an analytical sample of dimeric Cp^{V}Rh(III) complex **Rh18** (0.7 mg), phosphite adduct **Rh18’** was formed in situ.

^{31}P{^{1}H} NMR (162 MHz, CD_2Cl_2) δ = 103.01 (d, *J* = 211.0 Hz) ppm; **HRMS** (ESI/QTOF) *m/z* = calcd. for $[C_{47}H_{59}ClF_3O_5PRh]^+$, $[M\text{-}Cl^-]^+$: 929.2790, found: 929.2781.

Rhodium phosphite adduct (*R*)-**Rh19’**

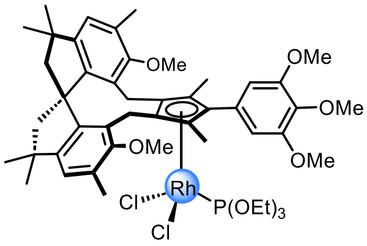

According to *General Procedure 6*, starting from an analytical sample of dimeric Cp^{V}Rh(III) complex **Rh19** (1.2 mg), phosphite adduct **Rh19’** was formed in situ.

^{31}P{^{1}H} NMR (162 MHz, CD_2Cl_2) δ = 104.11 (d, *J* = 213.8 Hz) ppm; **HRMS** (ESI/QTOF) *m/z* = calcd. for $[C_{49}H_{66}ClO_8PRh]^+$, $[M\text{-}Cl^-]^+$: 951.3233, found: 951.3219.

Rhodium phosphite adduct (*R*)-**Rh22’**

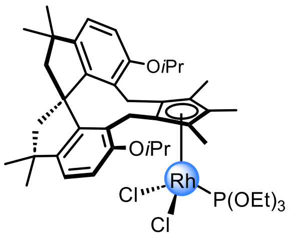

According to *General Procedure 6*, starting from an analytical sample of dimeric Cp^{V}Rh(III) complex **Rh22** (2.9 mg), phosphite adduct **Rh22’** was formed in situ.

^{31}P{^{1}H} NMR (162 MHz, CD_2Cl_2) δ = 107.91 (d, *J* = 215.7 Hz) ppm; **HRMS** (ESI/QTOF) *m/z* = calcd. for $[C_{43}H_{62}ClO_5PRh]^+$, $[M\text{-}Cl^-]^+$: 827.3073, found: 827.3073.

Rhodium phosphite adduct (*R*)-**Rh24’**

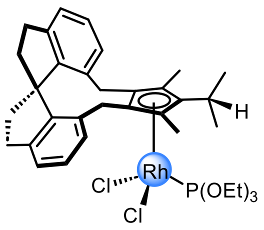

According to *General Procedure 6*, starting from an analytical sample of dimeric Cp^VRh(III) complex **Rh24** (1.7 mg), phosphite adduct **Rh24’** was formed in situ.

$^{31}P\{^1H\}$ NMR (162 MHz, CD_2Cl_2) δ = 109.33 (d, *J* = 218.8 Hz) ppm; **HRMS** (ESI/QTOF) *m/z* = calcd. for $[C_{35}H_{46}ClO_3PRh]^+$, $[M-Cl^-]^+$: 683.1923, found: 683.1928.

Rhodium phosphite adduct (*R*)-**Rh28’**

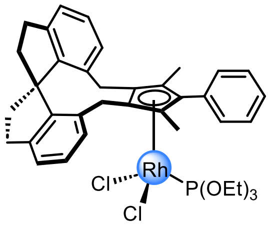

According to *General Procedure 6*, starting from an analytical sample of dimeric Cp^VRh(III) complex **Rh28** (1.2 mg), phosphite adduct **Rh28’** was formed in situ.

$^{31}P\{^1H\}$ NMR (162 MHz, CD_2Cl_2) δ = 107.37 (d, *J* = 217.4 Hz) ppm; **HRMS** (ESI/QTOF) *m/z* = calcd. for $[C_{38}H_{44}ClO_3PRh]^+$, $[M-Cl^-]^+$: 717.1766, found: 717.1776.

Rhodium phosphite adduct (*R*)-**Rh30’**

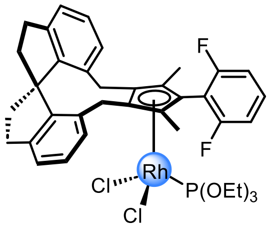

According to *General Procedure 6*, starting from an analytical sample of dimeric Cp^VRh(III) complex **Rh30** (2.8 mg), phosphite adduct **Rh30’** was formed in situ.

$^{31}P\{^1H\}$ NMR (162 MHz, CD_2Cl_2) δ = 104.87 (dd, *J* = 218.2, 3.2 Hz) ppm; **HRMS** (ESI/QTOF) *m/z* = calcd. for $[C_{38}H_{42}ClF_2O_3PRh]^+$, $[M-Cl^-]^+$: 753.1578, found: 753.1579.

5.8 Challenging Dialkylation-Complexation Sequences

A brief overview of the preliminary results for dialkylation-complexation sequences toward some challenging heteroatom-bearing ligand architectures is provided in **Scheme S26**.

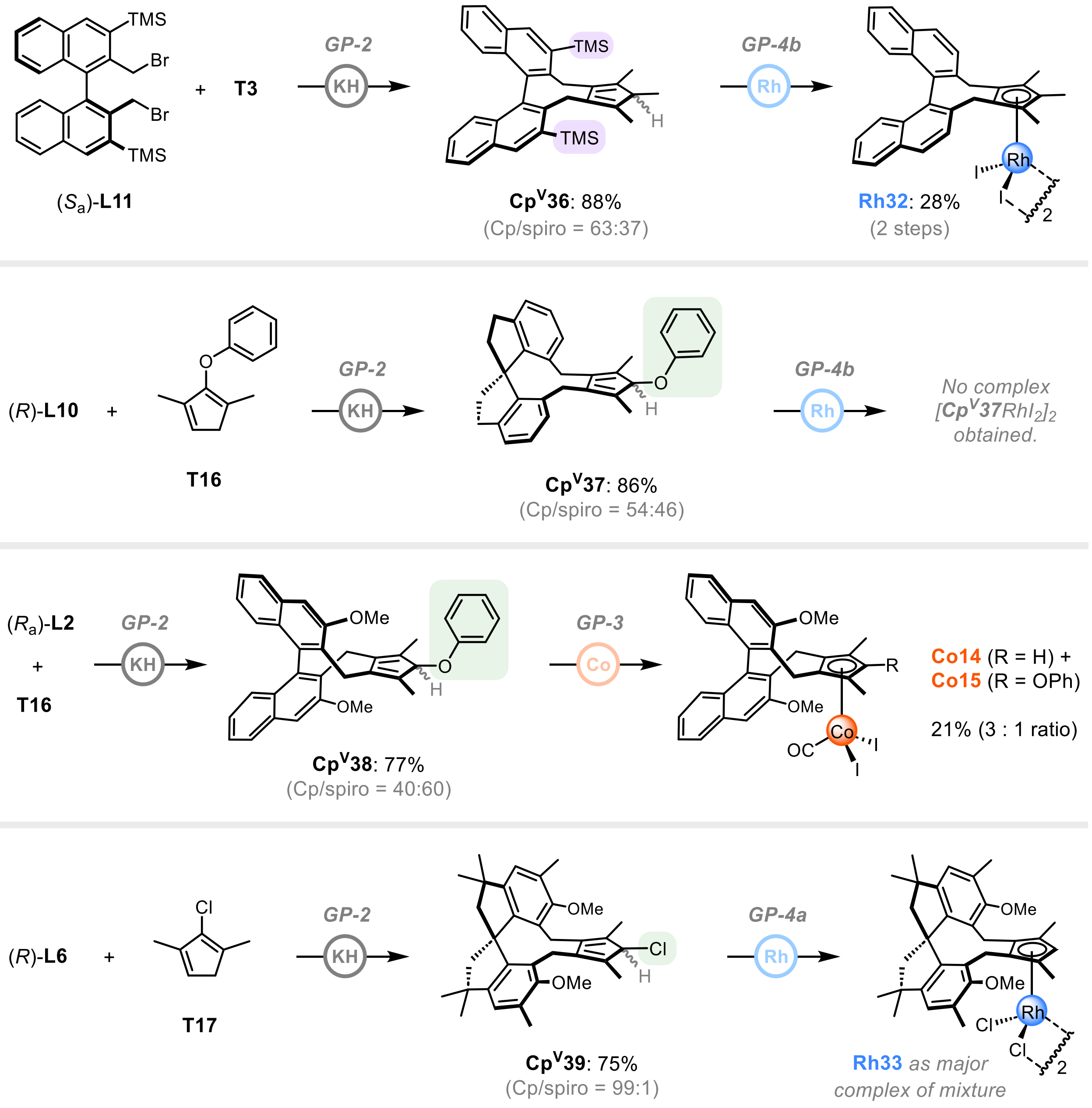

Scheme S26. Brief overview of challenging dialkylation-complexation sequences.

Using chiral dibromide (S_a)-**L11** with TMS-sidewalls on the binaphthyl core, the dialkylation of 1,2,3-Cp **T3** toward **Cp^{V}36** proceeded uneventfully in 88% yield. However, upon two-step Rh(III)-complexation using $[Rh(COD)Cl]_2$ as precursor and I_2 as oxidant (*General Procedure 4b*), the TMS-sidewalls were cleaved off, exclusively furnishing complex **Rh32** with H-sidewalls (28% yield over 2 steps). Notably, this process formally equals complexation of (S_a)-**Cp^{V}3**.

Pleasingly, the dialkylation reactions of phenoxy-substituted 1,2,3-Cp **T16** with dibromides (*R*)-**L10** and (R_a)-**L2** were both successful, furnishing interesting oxygen-bearing chiral Cp^VH ligands **Cp^{V}37** and **Cp^{V}38** in good yields (77-86%). However, subsequent rhodium complexation of **Cp^{V}37** using $[Rh(COD)OAc]_2$ as precursor and I_2 as oxidant (*General Procedure 4b*) failed to deliver the desired Rh(III) complex.

On the other hand, cobalt complexation of **Cp^{V}38** according to *General Procedure 3* provided a mixture of two Co(III) complexes **Co14**/**Co15** (3:1 ratio, 21% yield) due to partial cleavage of the phenoxy frontarm. The NMR data of the major tetrasubstituted complex **Co14** is provided *in infra*. The HSQC spectrum clearly identified the characteristic Cp-ring ^1H and ^{13}C NMR signals of **Co14** (δ_H = 5.33 ppm; δ_C = 93.6 ppm in CD_2Cl_2). The HRMS spectrum (**Figure S14**) distinguished the *m/z* signals of **Co14** and **Co15**.

Noteworthily, the dialkylation of more delicate chloro-bearing 1,2,3-Cp **T17** with dibromide (*R*)-**L6** was successful (75% yield). However, rhodium complexation of the resulting **Cp^{V}39** using $RhCl_3 \cdot 3\ H_2O$ at 140 °C (*General Procedure 4a*) also resulted in substantial frontarm cleavage. We obtained a mixture of species, predominantly comprising the tetrasubstituted Rh(III) complex **Rh33** (characteristic signals at δ_H = 5.10 ppm; δ_C = 75.4 ppm in d_6-DMSO) and only traces of the desired pentasubstituted complex, as confirmed by HRMS (**Figure S15**). Similar behavior has been observed for the *tert*-butyl frontarm of **Cp^{V}30** upon complexation toward **Rh26** using $RhCl_3 \cdot 3\ H_2O$ at 140 °C, and milder conditions proved successful in circumventing *t*Bu-cleavage.

Conclusion: The above results further illustrate the robustness of our combinatorial dialkylation approach, since its scope can be extended to incorporating delicate and rare heteroatom substituents in chiral cyclopentadiene scaffolds. Yet, regarding their metal complexation, further investigation is needed to determine suitable conditions that leave the chalcogen or halogen functionality untouched.

Cobalt complex **Co14**

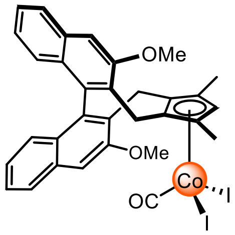

Black solid. Isolated as a **Co14**/**Co15** mixture (3:1 ratio). **^{1}H NMR** (600 MHz, CD_2Cl_2) δ = 7.87 (d, *J* = 8.2 Hz, 1H), 7.82 (d, *J* = 8.2 Hz, 1H), 7.45 – 7.42 (m, 2H), 7.39 (s, 1H), 7.29 (s, 1H), 7.13 – 7.08 (m, 2H), 7.06 (d, *J* = 8.4 Hz, 1H), 6.98 (d, *J* = 8.4 Hz, 1H), 5.33 (s, 1H), 4.13 (s, 3H), 4.09 (d, *J* = 16.2 Hz, 1H), 3.99 (s, 3H), 3.87 (d, *J* = 14.4 Hz, 1H), 3.82 (d, *J* = 14.4 Hz, 1H), 2.66 (s, 3H), 2.58 (d, *J* = 16.2 Hz, 1H), 2.12 (s, 3H) ppm; **^{13}C{^{1}H} NMR** (151 MHz, CD_2Cl_2) δ = 207.6, 156.4, 155.5, 138.7, 137.5, 134.5, 134.3, 130.3, 128.4, 127.4, 127.33, 127.29, 127.26, 127.2, 126.95, 126.92, 126.86, 124.5, 124.3, 119.5, 110.2, 106.3, 101.7, 98.7, 97.9, 93.6, 55.8, 55.7, 26.2, 23.1, 13.0, 12.2 ppm; **IR** (ATR) ṽ = 2957 (w), 2923 (w), 2054 (s), 1619 (w), 1596 (w), 1487 (w), 1454 (m), 1425 (w), 1409 (w), 1375 (w), 1330 (w), 1294 (w), 1237 (w), 1199 (m), 1162 (w), 1108 (m), 1019 (w), 909 (w), 865 (w), 833 (w), 748 (w), 731 (w), 468 (w) cm^{-1}; **HRMS** (nanochip-ESI/LTQ-Orbitrap) *m/z* = calcd. for $[C_{31}H_{27}CoIO_2]^+$, $[M\text{-}CO\text{-}I^-]^+$: 617.0382, found: 617.0377.

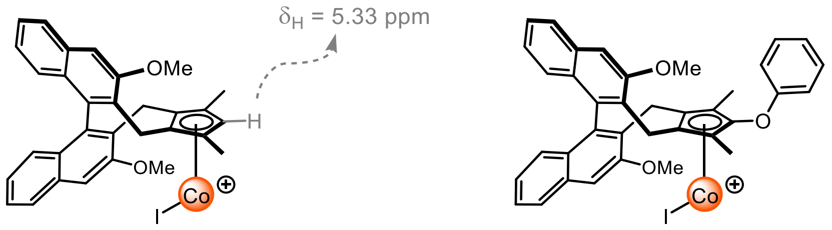

[**Co14**-CO-I$^-$]$^+$
Calculated for $C_{31}H_{27}CoIO_2^+$
m/z: **617.0383** (100.0%), 618.0416 (33.5%), 619.0450 (5.4%)

[**Co15**-CO-I$^-$]$^+$
Calculated for $C_{37}H_{31}CoIO_3^+$
m/z: **709.0645** (100.0%), 710.0678 (40.0%), 711.0712 (7.8%), 712.0745 (1.0%)

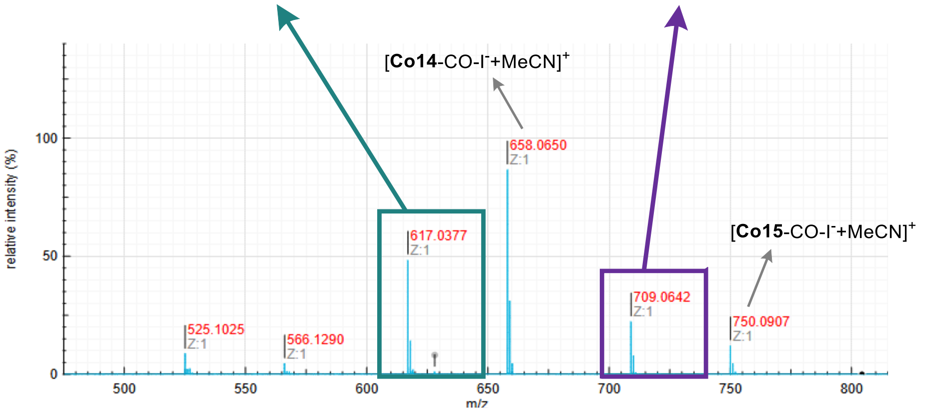

Figure S14. HRMS spectrum (nanochip-based ESI/LTQ-Orbitrap) of the **Co14**/**Co15** mixture (3:1 ratio).

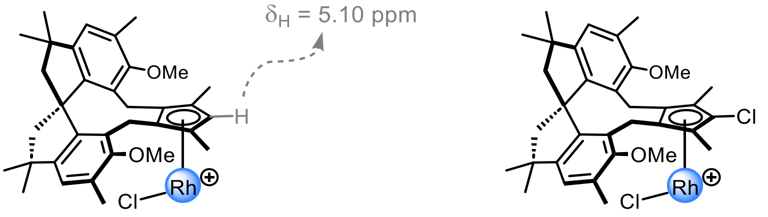

[(**Rh33**/2)-Cl$^-$]$^+$
Calculated for $C_{34}H_{41}ClO_2Rh^+$
m/z: **619.1845** (100.0%), 620.1879 (36.8%), 621.1816 (32.0%), 622.1849 (11.8%), 621.1912 (6.6%), 623.1883 (2.1%)

[**Cp^{V}39RhCl**]$^+$
Calculated for $C_{34}H_{40}Cl_2O_2Rh^+$
m/z: **653.1455** (100.0%), 655.1426 (64.0%), 654.1489 (36.8%), 656.1459 (23.5%), 657.1396 (10.2%), 655.1522 (6.6%)

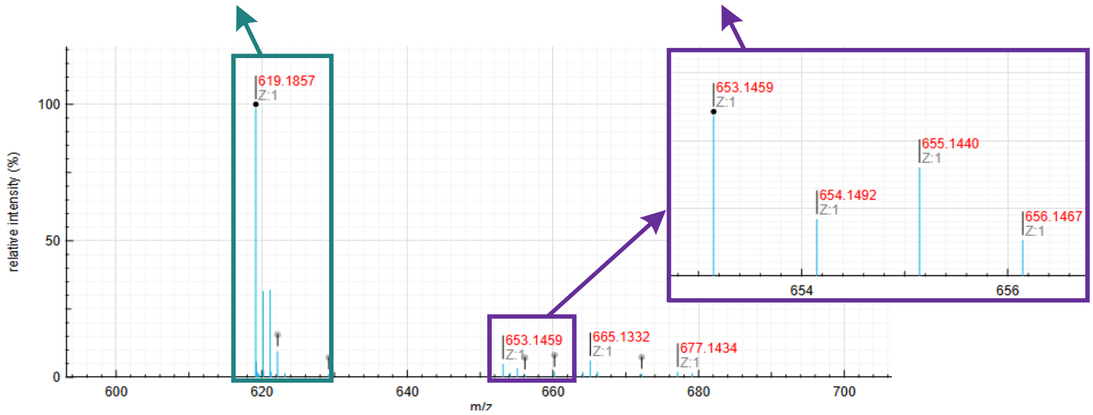

Figure S15. HRMS spectrum (ESI/QTOF) of a mixture predominantly containing complex **Rh33**.

6. Asymmetric Catalytic Transformations

6.1 Rh-catalyzed Allylic C-H Amination of Unactivated Alkenes

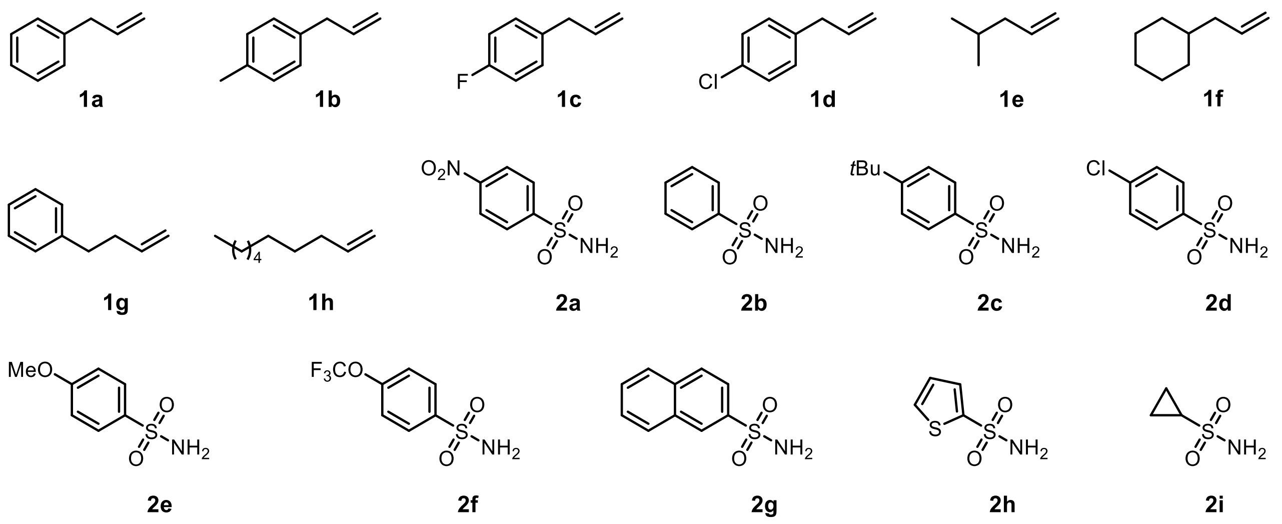

Figure S16. Substrates used in this work for the $Cp^{V}Rh(III)$-catalyzed enantioselective allylic C-H amination.

All substrates **1a-h** and **2a-i** were commercially available and were used as obtained from the supplier. Racemic allylic amines **3** were prepared according to *General Procedure 7a* using achiral complex $[Cp^*RhCl_2]_2$ as a catalyst.

<u>*General Procedure 7*</u> *– $Cp^{V}Rh(III)$-Catalyzed Allylic C-H Amination of Unactivated Alkenes.*

<u>General Procedure 7a</u>: Catalyst Screening – Without protection from oxygen or moisture, an oven-dried microwave vial was charged with **Rh** catalyst (1.25 mol% dimer), silver(I) hexafluoroantimonate (1.7 mg, 10 mol%), magnesium(II) oxide (2.0 mg, 1 equiv.), bis(*tert*-butylcarbonyloxy)iodobenzene (40.6 mg, 2 equiv.), and 4-nitrobenzenesulfonamide **2a** (10.1 mg, 0.05 mmol). A solution of allylbenzene **1a** (13 µL, 2 equiv.) in 2,2,2-trifluoroethanol (10 mL/mmol, i.e. 500 µL) was added. The vial was capped under air, and the reaction mixture was stirred at room temperature (25 °C) for 24 hours. Next, the reaction was diluted with ethyl acetate and then filtered through a pad of silica gel (3 cm) with ethyl acetate as eluent. After removing all volatiles *in vacuo*, the crude residue was analyzed by qNMR in $CDCl_3$ with 1,3,5-trimethoxybenzene as an internal standard. Notably, the $CDCl_3$ solution was first passed through a hydrophilic PTFE syringe filter (0.22 µm pore size) before qNMR analysis. To determine the enantiomeric ratio, purification was performed by Prep. TLC on silica (pentane/EtOAc = 7:3), affording chiral branched allylic amine **3a** as a white solid, which was then analyzed by HPLC using a chiral stationary phase (Chiralpak IB, 4.6 x 150 mm, 3 µm, Hexane/*i*PrOH 90:10, 1.0 mL/min, 35 °C, 254 nm).

The results of the catalyst screening via *General Procedure 7a* are visualized in **Scheme S27**.

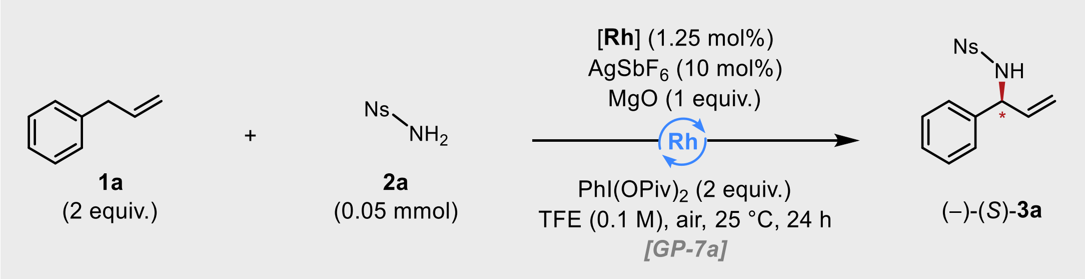

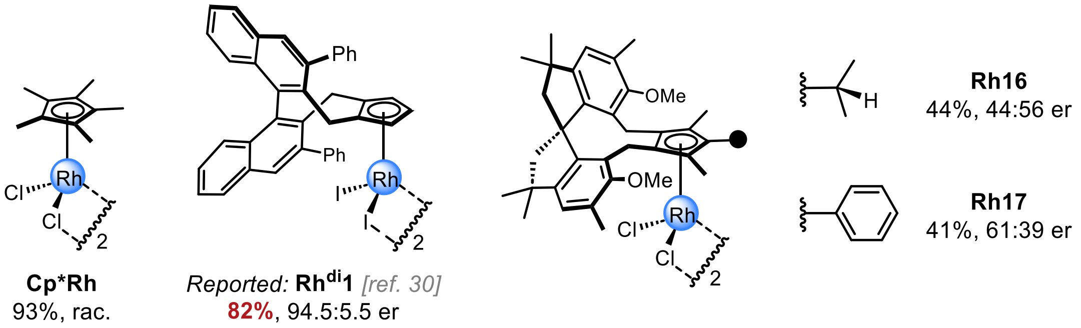

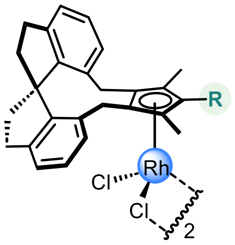

	Frontarm R	% yield	er
**Rh24**	*i*Pr	92	96:4
**Rh25**	Cy	97	96:4
**Rh27**	Me	91	89:11
**Rh26**	*t*Bu	94	89:11
**Rh28**	Ph	**97**	**97:3**
**Rh30**	2,6-F-C_6H_3	97	94:6
**Rh29**	3,5-*t*Bu-C_6H_3	98	96:4

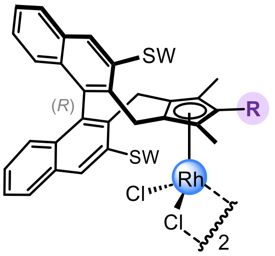

	SW	Frontarm R	% yield	er
**Rh1a**	H	*i*Pr	90	34:66
**Rh6**	H	4-OMe-C_6H_4	94	**23:77**
**Rh7**	H	2,6-OMe-C_6H_3	92	**80:20**
(S_a)-**Rh13**	Ph	*i*Pr	64	53:47
(S_a)-**Rh14**	Ph	Me	94	25:75

Enantio-inversion

Scheme S27. Screening of Cp^VRh(III) catalysts for the enantioselective allylic C-H amination toward **3a**.

***General Procedure 7b*: *Reaction Scope* –** Without protection from oxygen or moisture, an oven-dried microwave vial was charged with (*R*)-**Rh28** catalyst (1.47 mg, 1.25 mol%), silver(I) hexafluoroantimonate (3.4 mg, 10 mol%), magnesium(II) oxide (4.0 mg, 1 equiv.), bis(*tert*-butylcarbonyloxy)iodobenzene (81.3 mg, 2 equiv.), and sulfonamide **2** (0.10 mmol). A solution of alkene **1** (2 equiv.) in 2,2,2-trifluoroethanol (10 mL/mmol, i.e. 1 mL) was added. The vial was capped under air, and the reaction mixture was stirred at room temperature (25 °C) for 24 hours. After removal of all volatiles *in vacuo*, the crude residue was redissolved in DCM, and the resulting suspension was filtered through a pad of silica gel (3 cm) with DCM as eluent, which retained most color and provided a homogeneous light-yellow solution. After removal of all volatiles *in vacuo*, the obtained residue was purified by Prep. TLC on silica (pentane/EtOAc), affording chiral branched allylic amine **3**. Subsequent analysis by HPLC using a chiral stationary phase (Hexane/*i*PrOH) provided the enantiomeric ratio.

The results of the reaction scope investigation via *General Procedure 7b* are visualized in **Scheme S28**.

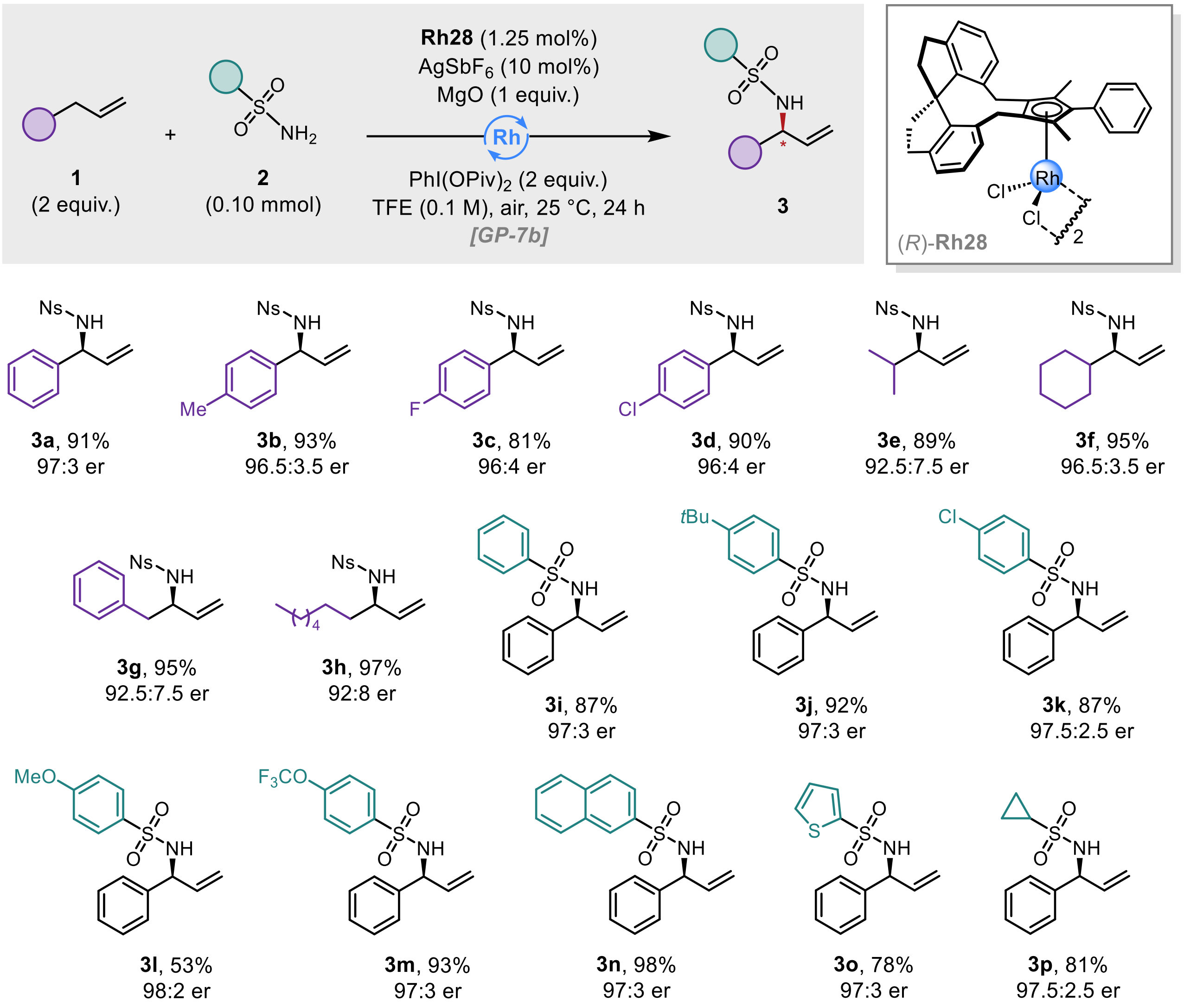

Scheme S28. Scope of the Cp^VRh(III)-catalyzed enantioselective allylic C-H amination of unactivated alkenes.

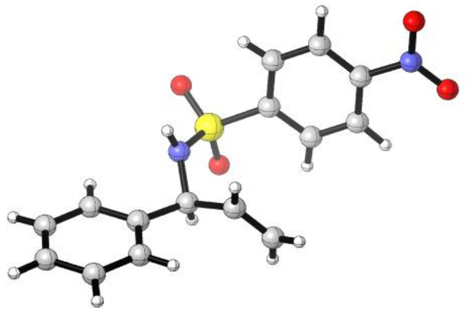

Figure S17. Solid-state X-ray structure of (−)-(*S*)-**3a** (CCDC: 2519618) showing 50% probability thermal ellipsoids.

(*S*)-4-Nitro-*N*-(1-phenylallyl)benzenesulfonamide (**3a**)

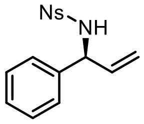

According to *General Procedure 7b*, starting from **1a** and **2a** (scale: 105 µmol), allylic amine (*S*)-**3a** (30.5 mg, 96 µmol, 91% yield, 97:3 er) was obtained as a white solid with the characterization data matching those previously reported.[29,30] Purification was performed by Prep. TLC on silica (pentane/EtOAc = 7:3). A suitable crystal for X-ray analysis (**Figure S17**) was obtained by slow evaporation of a concentrated solution in DCM, and confirmed the absolute configuration of (−)-**3a** as *S*.

^{1}H NMR (400 MHz, $CDCl_3$) δ = 8.20 – 8.16 (m, 2H), 7.86 – 7.82 (m, 2H), 7.23 – 7.19 (m, 3H), 7.11 – 7.06 (m, 2H), 5.90 (ddd, *J* = 17.0, 10.3, 5.8 Hz, 1H), 5.22 – 5.18 (m, 1H), 5.17 – 5.12 (m, 1H), 5.11 – 5.06 (m, 1H), 4.94 (d, *J* = 7.3 Hz, 1H) ppm; **R_f** (pentane/EtOAc, 4:1) = 0.26; $[\alpha]_D^{22}$ = -21.9 (*c* = 0.57, $CHCl_3$); **Chiral HPLC** (Chiralpak IB, 4.6 x 150 mm, 3 µm, Hexane/*i*PrOH 90:10, 1.0 mL/min, 35 °C, 254 nm) t_r (minor) = 10.05 min, t_r (major) = 10.68 min, 97:3 er; **XRD** (Cu*Kα*, R_1 = 3.14%) CCDC: 2519618.

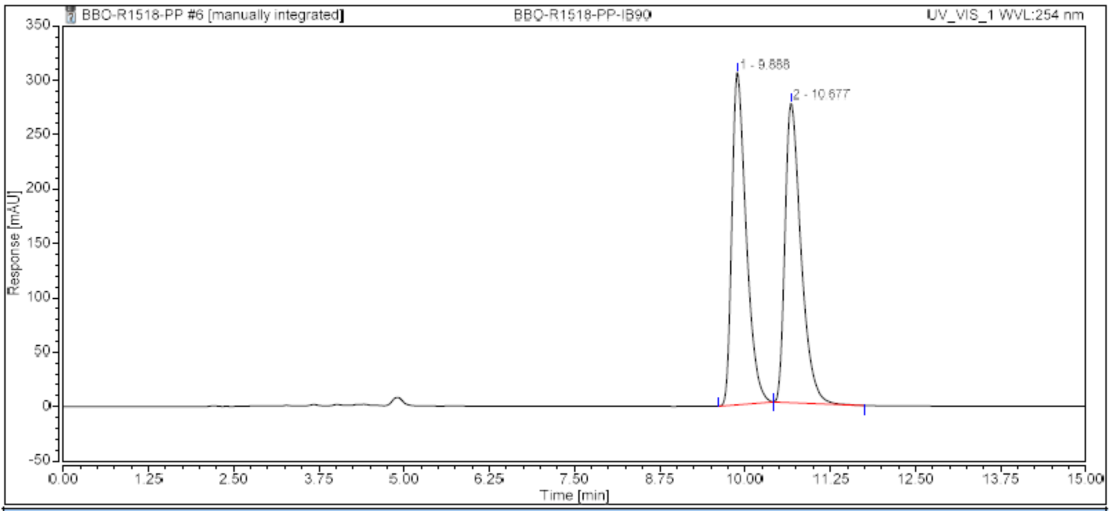

Integration Results

No.	Peak Name	Retention Time min	Area mAU*min	Height mAU	Relative Area %	Relative Height %	Amount n.a.
1		9.888	74.570	305.253	49.90	52.55	n.a.
2		10.677	74.869	275.641	50.10	47.45	n.a.

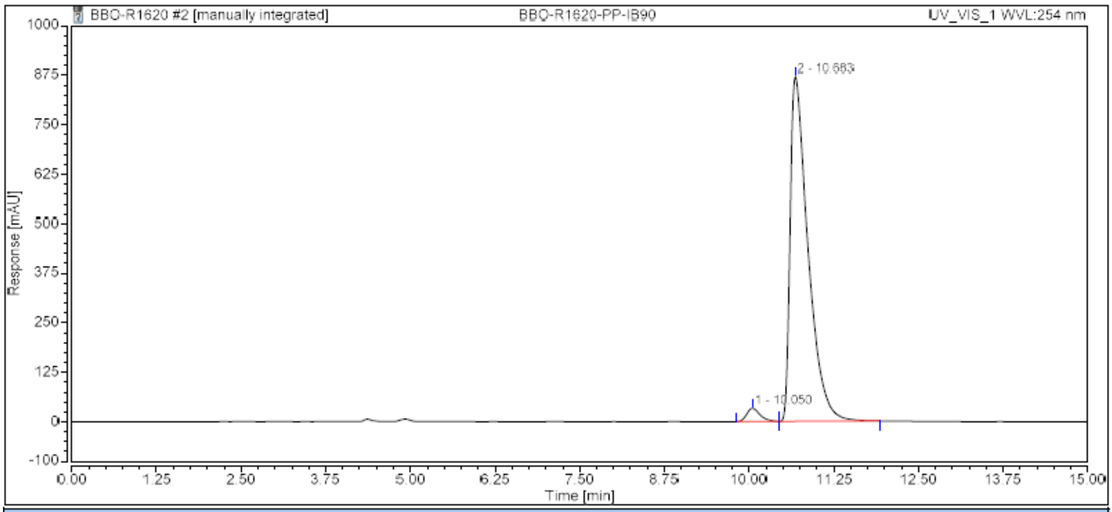

Integration Results

No.	Peak Name	Retention Time min	Area mAU*min	Height mAU	Relative Area %	Relative Height %	Amount n.a.
1		10.050	7.665	33.800	2.80	3.74	n.a.
2		10.683	265.803	870.974	97.20	96.26	n.a.

(*S*)-4-Nitro-*N*-(1-(*p*-tolyl)allyl)benzenesulfonamide (**3b**)

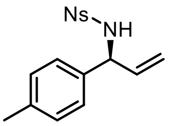

According to *General Procedure 7b*, starting from **1b** and **2a** (scale: 108 µmol), allylic amine (*S*)-**3b** (33.3 mg, 100 µmol, 93% yield, 96.5:3.5 er) was obtained as a white solid with the characterization data matching those previously reported.[29,30] Purification was performed by Prep. TLC on silica (pentane/EtOAc = 7:3).

^{1}H NMR (400 MHz, $CDCl_3$) δ = 8.20 – 8.15 (m, 2H), 7.86 – 7.81 (m, 2H), 7.02 – 6.97 (m, 2H), 6.97 – 6.93 (m, 2H), 5.87 (ddd, *J* = 17.0, 10.3, 5.7 Hz, 1H), 5.19 – 5.08 (m, 3H), 5.06 – 5.00 (m, 1H), 2.27 (s, 3H) ppm; **R_f** (pentane/EtOAc, 3:1) = 0.42; $[\alpha]_D^{22}$ = -24.6 (*c* = 0.57, $CHCl_3$); **Chiral HPLC** (Chiralpak IH, 4.6 x 150 mm, 3 µm, Hexane/*i*PrOH 80:20, 1.0 mL/min, 35 °C, 254 nm) t_r (minor) = 9.85 min, t_r (major) = 11.25 min, 96.5:3.5 er.

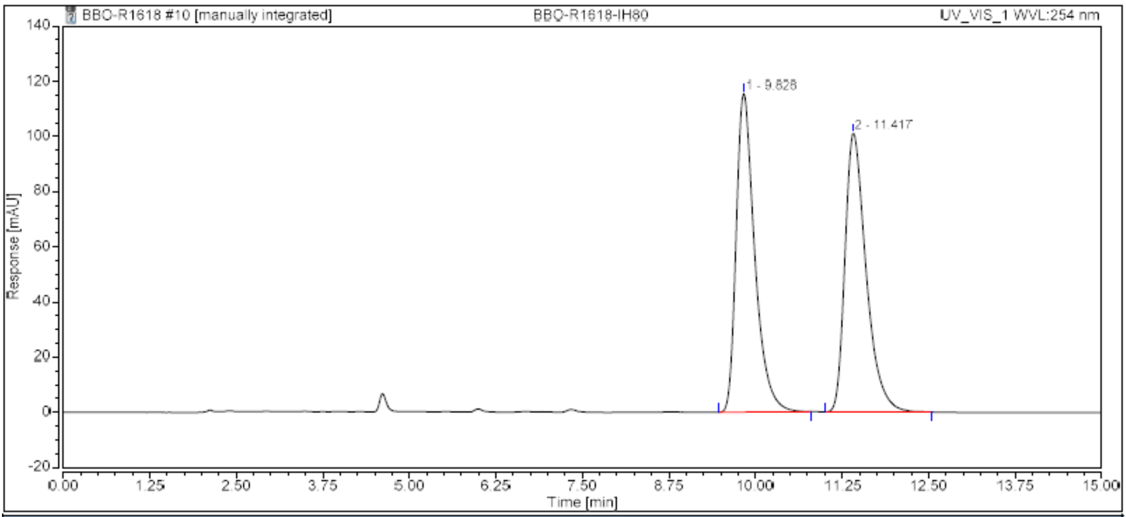

Integration Results

No.	Peak Name	Retention Time min	Area mAU*min	Height mAU	Relative Area %	Relative Height %	Amount n.a.
1		9.828	35.854	115.546	50.01	53.32	n.a.
2		11.417	35.846	101.164	49.99	46.68	n.a.

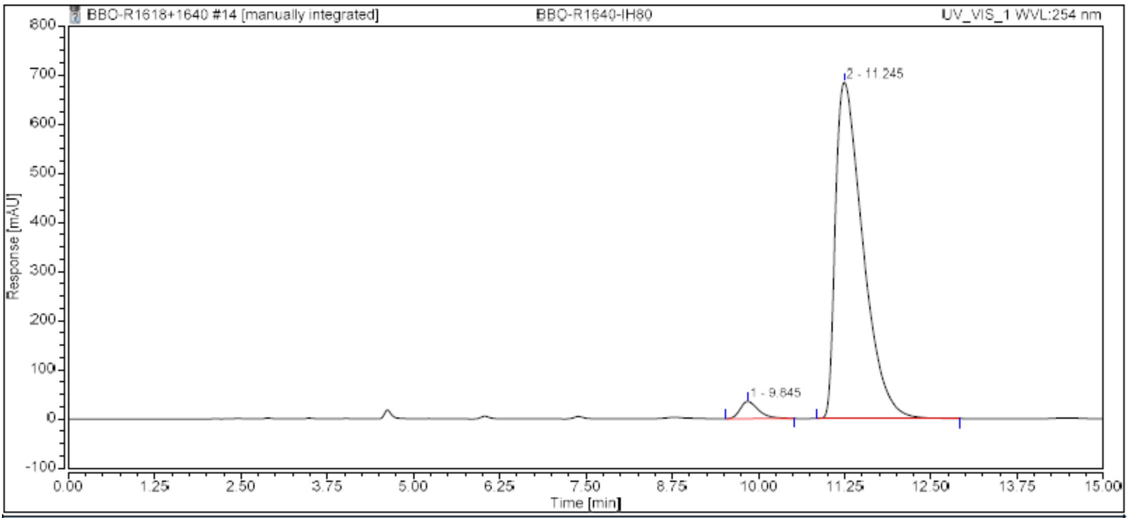

Integration Results

No.	Peak Name	Retention Time min	Area mAU*min	Height mAU	Relative Area %	Relative Height %	Amount n.a.
1		9.845	10.847	35.005	3.34	4.87	n.a.
2		11.245	314.031	684.124	96.66	95.13	n.a.

(*S*)-*N*-(1-(4-Fluorophenyl)allyl)-4-nitrobenzenesulfonamide (**3c**)

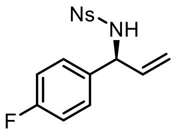

According to *General Procedure 7b*, starting from **1c** and **2a** (scale: 103 µmol), allylic amine (*S*)-**3c** (28.1 mg, 84 µmol, 81% yield, 96:4 er) was obtained as a white solid with the characterization data matching those previously reported.[29] Purification was performed by Prep. TLC on silica (pentane/EtOAc = 3:1).

^{1}H NMR (400 MHz, $CDCl_3$) δ = 8.27 – 8.21 (m, 2H), 7.91 – 7.86 (m, 2H), 7.14 – 7.08 (m, 2H), 6.96 – 6.89 (m, 2H), 5.85 (ddt, *J* = 16.7, 10.2, 2.9 Hz, 1H), 5.19 (d, *J* = 10.3 Hz, 1H), 5.14 – 5.05 (m, 3H) ppm; **^{19}F{^{1}H} NMR** (376 MHz, $CDCl_3$) δ = -113.16 ppm; **R_f** (pentane/EtOAc, 4:1) = 0.32; $[\alpha]_D^{22}$ = -23.9 (*c* = 0.65, $CHCl_3$); **Chiral HPLC** (Chiralpak IH, 4.6 x 150 mm, 3 µm, Hexane/*i*PrOH 85:15, 1.0 mL/min, 35 °C, 254 nm) t_r (minor) = 15.61 min, t_r (major) = 17.17 min, 96:4 er.

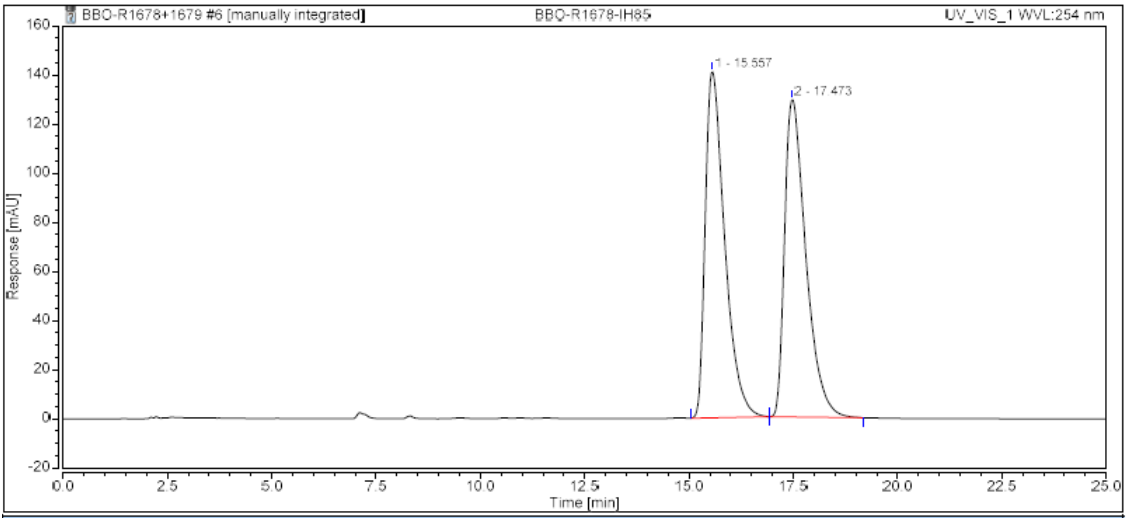

Integration Results

No.	Peak Name	Retention Time min	Area mAU*min	Height mAU	Relative Area %	Relative Height %	Amount n.a.
1		15.557	76.127	141.103	49.98	52.17	n.a.
2		17.473	76.183	129.356	50.02	47.83	n.a.

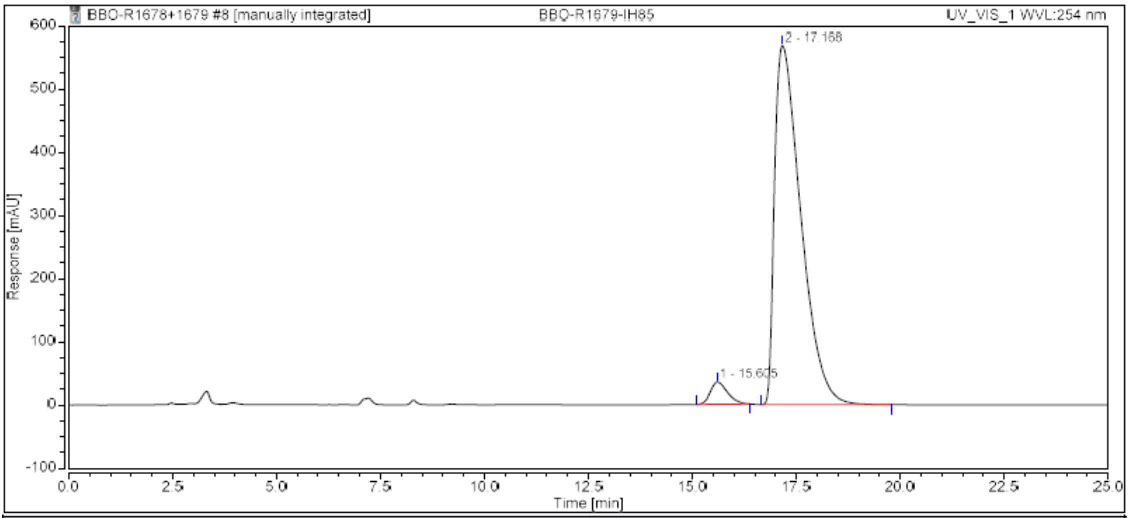

Integration Results

No.	Peak Name	Retention Time min	Area mAU*min	Height mAU	Relative Area %	Relative Height %	Amount n.a.
1		15.605	17.074	35.387	3.94	5.86	n.a.
2		17.168	415.978	568.144	96.06	94.14	n.a.

(*S*)-*N*-(1-(4-Chlorophenyl)allyl)-4-nitrobenzenesulfonamide (**3d**)

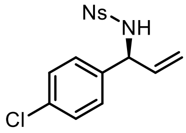

According to *General Procedure 7b*, starting from **1d** and **2a** (scale: 106 µmol), allylic amine (*S*)-**3d** (33.7 mg, 96 µmol, 90% yield, 96:4 er) was obtained as a white solid with the characterization data matching those previously reported.[29] Purification was performed by Prep. TLC on silica (pentane/EtOAc = 7:3).

^{1}H NMR (400 MHz, $CDCl_3$) δ = 8.26 – 8.22 (m, 2H), 7.90 – 7.85 (m, 2H), 7.23 – 7.18 (m, 2H), 7.09 – 7.04 (m, 2H), 5.83 (ddd, *J* = 17.0, 10.3, 5.3 Hz, 1H), 5.22 – 5.17 (m, 1H), 5.14 – 5.03 (m, 3H) ppm; **R$_f$** (pentane/EtOAc, 3:1) = 0.45; $[\alpha]_D^{22}$ = -27.2 (*c* = 0.63, $CHCl_3$); **Chiral HPLC** (Chiralpak ID, 4.6 x 150 mm, 3 µm, Hexane/*i*PrOH 95:5, 1.0 mL/min, 35 °C, 254 nm) t$_r$ (major) = 16.01 min, t$_r$ (minor) = 17.85 min, 96:4 er.

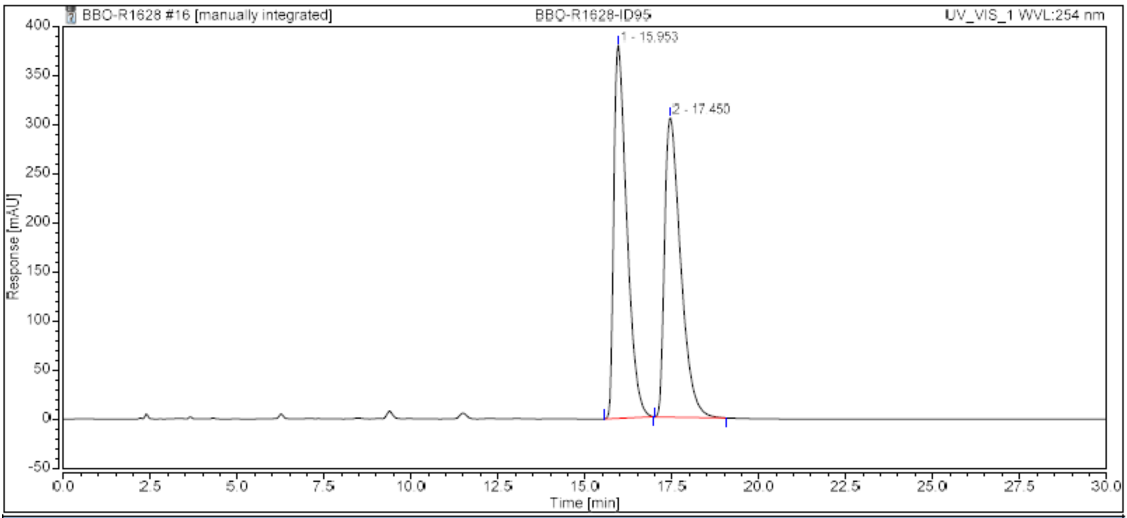

Integration Results							
No.	Peak Name	Retention Time min	Area mAU*min	Height mAU	Relative Area %	Relative Height %	Amount n.a.
1		15.953	164.666	379.749	50.16	55.44	n.a.
2		17.450	163.626	305.189	49.84	44.56	n.a.

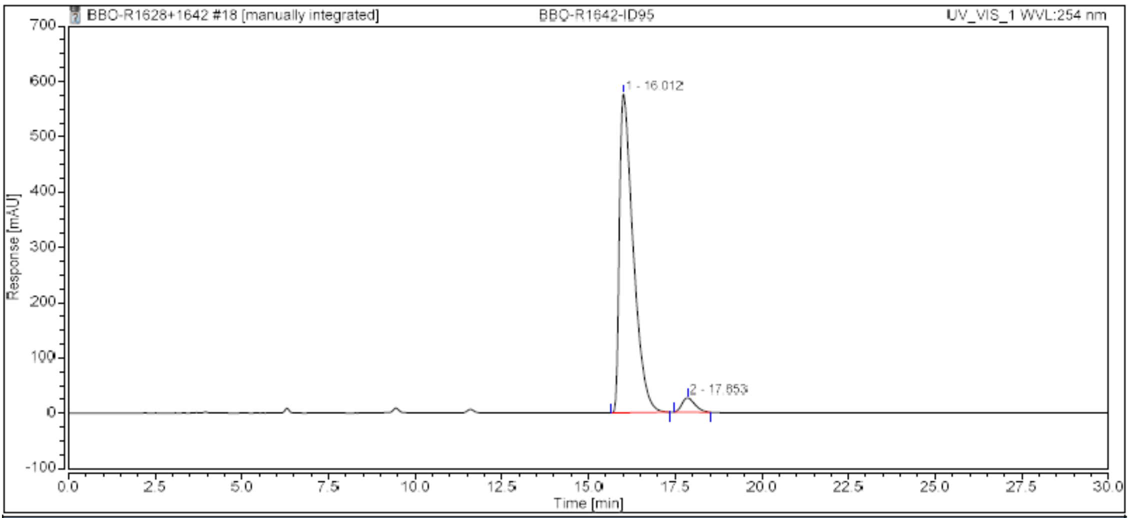

Integration Results							
No.	Peak Name	Retention Time min	Area mAU*min	Height mAU	Relative Area %	Relative Height %	Amount n.a.
1		16.012	265.706	576.303	95.91	95.69	n.a.
2		17.853	11.327	25.948	4.09	4.31	n.a.

(*R*)-*N*-(4-Methylpent-1-en-3-yl)-4-nitrobenzenesulfonamide (**3e**)

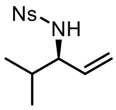

According to *General Procedure 7b*, starting from **1e** and **2a** (scale: 109 µmol), allylic amine (*R*)-**3e** (27.6 mg, 97 µmol, 89% yield, 92.5:7.5 er) was obtained as a white solid with the characterization data matching those previously reported.[30] Purification was performed by Prep. TLC on silica (pentane/EtOAc = 3:1).

^{1}H NMR (400 MHz, $CDCl_3$) δ = 8.35 – 8.31 (m, 2H), 8.05 – 8.01 (m, 2H), 5.50 (ddd, *J* = 17.2, 10.4, 6.9 Hz, 1H), 4.98 (dt, *J* = 10.5, 1.1 Hz, 1H), 4.91 (dt, *J* = 17.1, 1.2 Hz, 1H), 4.74 (d, *J* = 8.8 Hz, 1H), 3.75 – 3.67 (m, 1H), 1.84 – 1.71 (m, 1H), 0.88 (d, *J* = 6.8 Hz, 3H), 0.87 (d, *J* = 6.8 Hz, 3H) ppm; **R_f** (pentane/EtOAc, 4:1) = 0.38; $[\alpha]_D^{22}$ = -36.8 (*c* = 0.58, $CHCl_3$); **Chiral HPLC** (Chiralpak IC, 4.6 x 150 mm, 3 µm, Hexane/*i*PrOH 80:20, 1.0 mL/min, 35 °C, 254 nm) t_r (major) = 6.85 min, t_r (minor) = 8.00 min, 92.5:7.5 er.

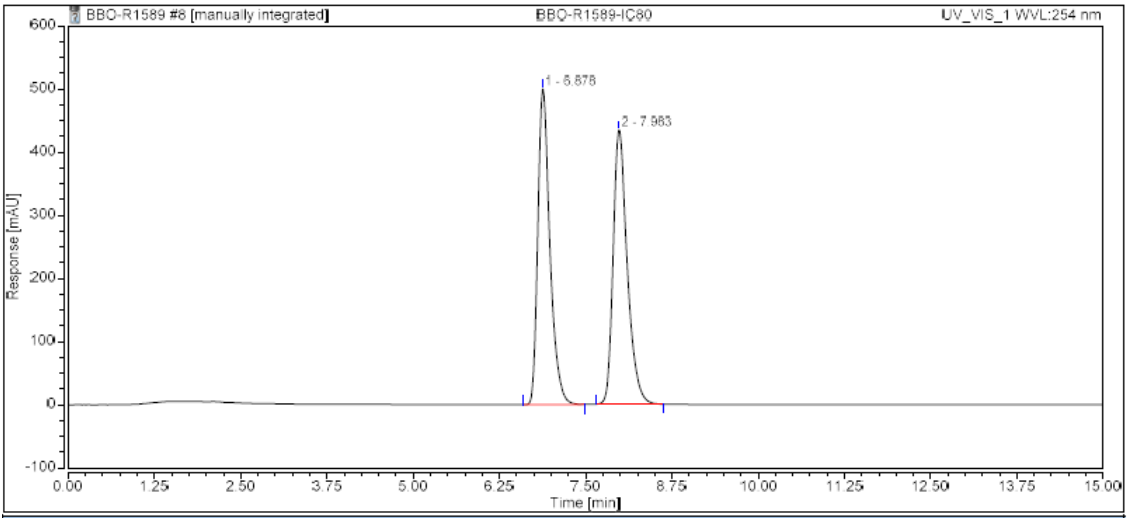

Integration Results

No.	Peak Name	Retention Time min	Area mAU*min	Height mAU	Relative Area %	Relative Height %	Amount n.a.
1		6.878	101.204	499.907	49.63	53.50	n.a.
2		7.983	102.718	434.456	50.37	46.50	n.a.

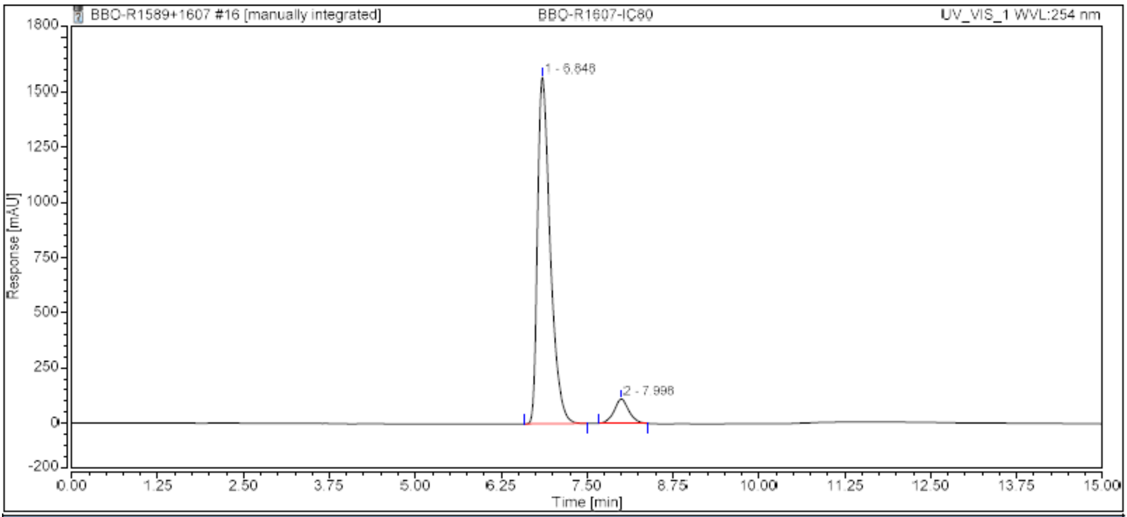

Integration Results

No.	Peak Name	Retention Time min	Area mAU*min	Height mAU	Relative Area %	Relative Height %	Amount n.a.
1		6.848	335.800	1568.307	92.47	93.44	n.a.
2		7.998	27.362	110.133	7.53	6.56	n.a.

(*S*)-*N*-(1-Cyclohexylallyl)-4-nitrobenzenesulfonamide (**3f**)

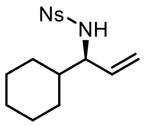

According to *General Procedure 7b*, starting from **1f** and **2a** (scale: 105 µmol), allylic amine (*S*)-**3f** (32.4 mg, 100 µmol, 95% yield, 96.5:3.5 er) was obtained as a white solid with the characterization data matching those previously reported.[30] Purification was performed by Prep. TLC on silica (pentane/EtOAc = 4:1).

^{1}H NMR (400 MHz, $CDCl_3$) δ = 8.35 – 8.30 (m, 2H), 8.04 – 8.00 (m, 2H), 5.48 (ddd, *J* = 17.3, 10.4, 7.1 Hz, 1H), 4.95 (dt, *J* = 10.4, 1.1 Hz, 1H), 4.88 (dt, *J* = 17.2, 1.2 Hz, 1H), 4.66 (d, *J* = 8.8 Hz, 1H), 3.74 – 3.66 (m, 1H), 1.79 – 1.69 (m, 3H), 1.68 – 1.53 (m, 2H), 1.46 – 1.35 (m, 1H), 1.24 – 1.03 (m, 3H), 1.03 – 0.84 (m, 2H) ppm; **R_f** (pentane/EtOAc, 4:1) = 0.47; $[\alpha]_D^{22}$ = -49.0 (*c* = 0.33, $CHCl_3$); **Chiral HPLC** (Chiralpak IA, 4.6 x 150 mm, 3 µm, Hexane/*i*PrOH 90:10, 1.0 mL/min, 35 °C, 254 nm) t_r (minor) = 8.03 min, t_r (major) = 10.05 min, 96.5:3.5 er.

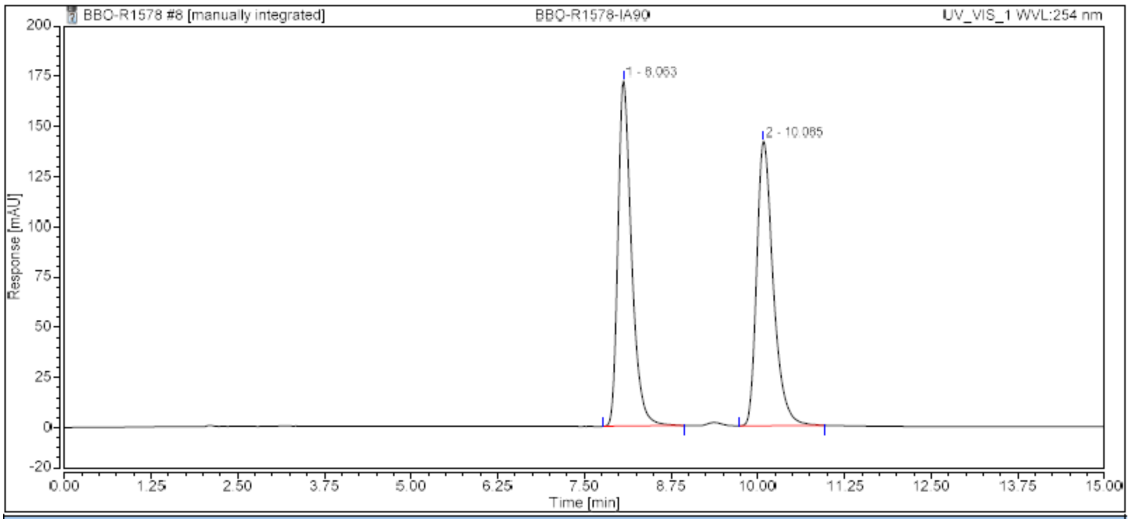

Integration Results

No.	Peak Name	Retention Time min	Area mAU*min	Height mAU	Relative Area %	Relative Height %	Amount n.a.
1		8.063	39.244	172.028	50.11	54.79	n.a.
2		10.085	39.076	141.952	49.89	45.21	n.a.

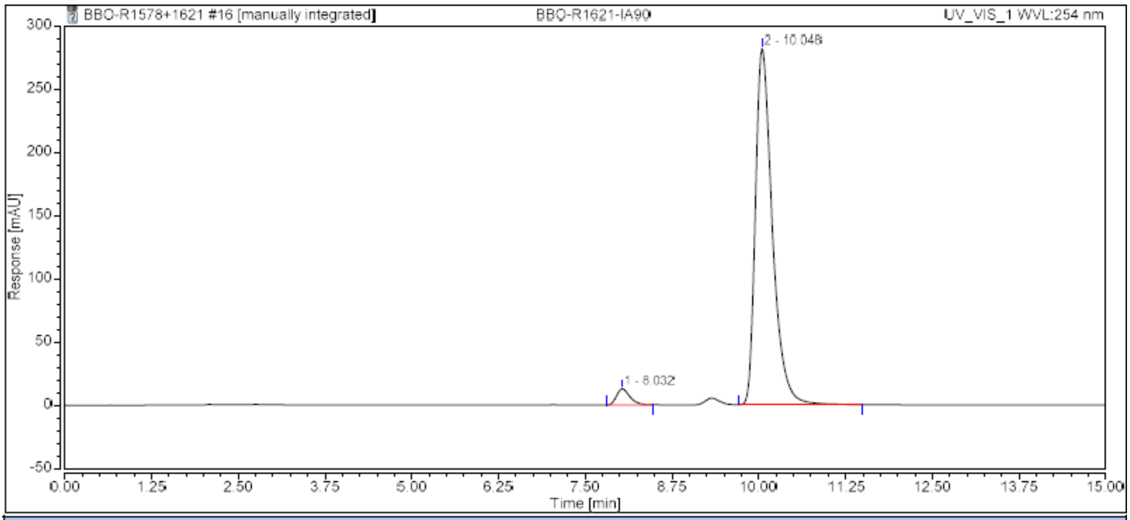

Integration Results

No.	Peak Name	Retention Time min	Area mAU*min	Height mAU	Relative Area %	Relative Height %	Amount n.a.
1		8.032	2.886	12.893	3.49	4.37	n.a.
2		10.048	79.710	281.996	96.51	95.63	n.a.

(*R*)-4-Nitro-*N*-(1-phenylbut-3-en-2-yl)benzenesulfonamide (**3g**)

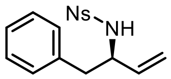

According to *General Procedure 7b*, starting from **1g** and **2a** (scale: 102 µmol), allylic amine (*R*)-**3g** (32.4 mg, 97 µmol, 95% yield, 92.5:7.5 er) was obtained as a white solid with the characterization data matching those previously reported.[29,30] Purification was performed by Prep. TLC on silica (pentane/EtOAc = 3:1).

^{1}H NMR (400 MHz, $CDCl_3$) δ = 8.20 – 8.16 (m, 2H), 7.80 – 7.76 (m, 2H), 7.23 – 7.15 (m, 3H), 7.05 – 6.99 (m, 2H), 5.74 (ddd, *J* = 17.1, 10.4, 6.0 Hz, 1H), 5.17 – 5.08 (m, 2H), 4.60 (d, *J* = 7.9 Hz, 1H), 4.14 – 4.05 (m, 1H), 2.91 (dd, *J* = 13.9, 5.6 Hz, 1H), 2.70 (dd, *J* = 13.9, 8.1 Hz, 1H) ppm; **R_f** (pentane/EtOAc, 4:1) = 0.29; $[\alpha]_D^{22}$ = +7.5 (*c* = 0.51, $CHCl_3$); **Chiral HPLC** (Chiralpak IB, 4.6 x 150 mm, 3 µm, Hexane/*i*PrOH 90:10, 1.0 mL/min, 35 °C, 254 nm) t_r (minor) = 10.11 min, t_r (major) = 11.52 min, 92.5:7.5 er.

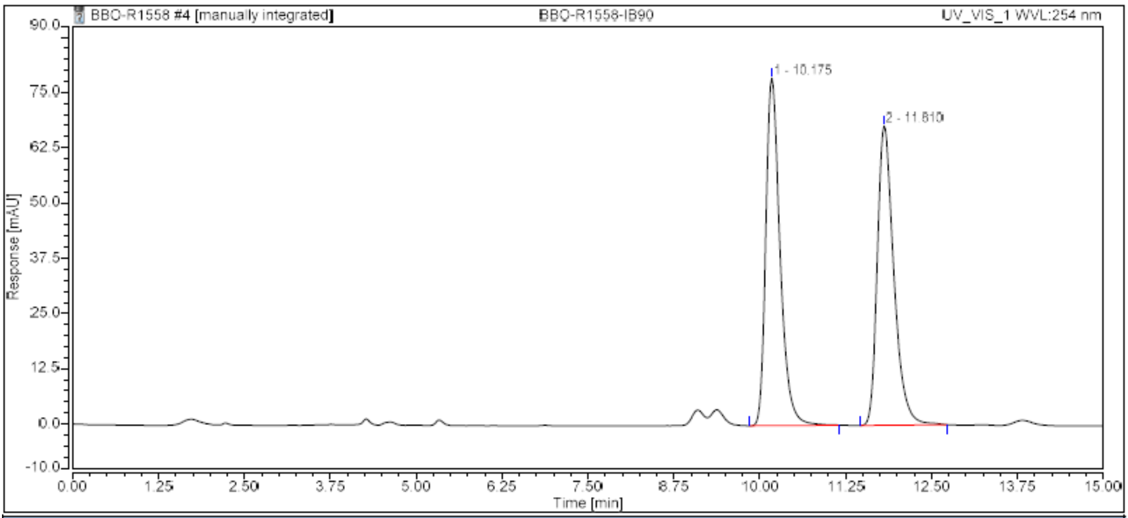

Integration Results

No.	Peak Name	Retention Time min	Area mAU*min	Height mAU	Relative Area %	Relative Height %	Amount n.a.
1		10.175	18.887	78.602	50.03	53.71	n.a.
2		11.810	18.862	67.743	49.97	46.29	n.a.

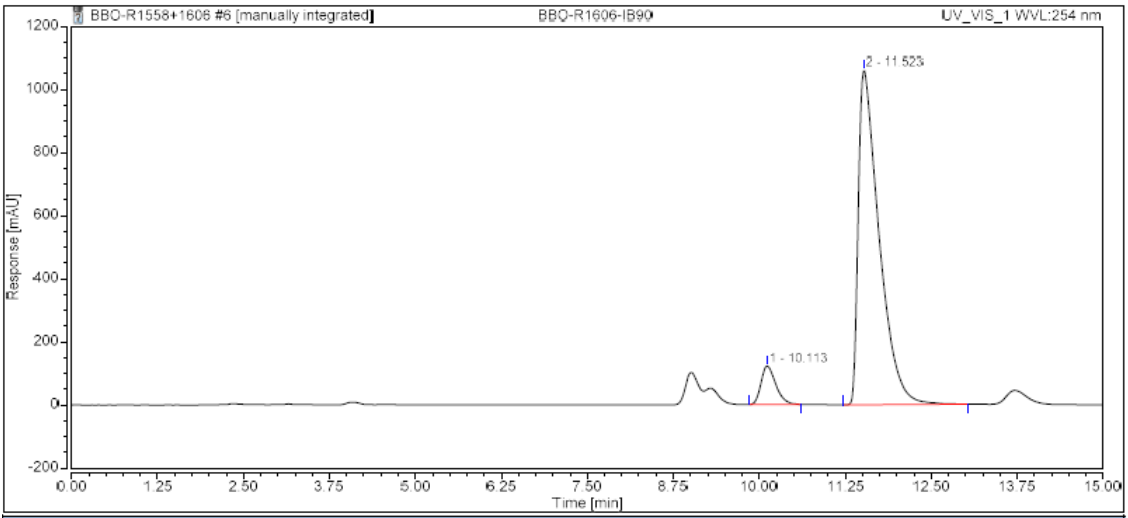

Integration Results

No.	Peak Name	Retention Time min	Area mAU*min	Height mAU	Relative Area %	Relative Height %	Amount n.a.
1		10.113	30.260	122.091	7.55	10.32	n.a.
2		11.523	370.569	1060.872	92.45	89.68	n.a.

(*R*)-*N*-(Dec-1-en-3-yl)-4-nitrobenzenesulfonamide (**3h**)

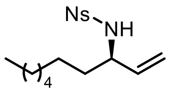

According to *General Procedure 7b*, starting from **1h** and **2a** (scale: 106 µmol), allylic amine (*R*)-**3h** (35.0 mg, 103 µmol, 97% yield, 92:8 er) was obtained as a white solid with the characterization data matching those previously reported.[29,30] Purification was performed by Prep. TLC on silica (pentane/EtOAc = 4:1).

^{1}H NMR (400 MHz, $CDCl_3$) δ = 8.36 – 8.31 (m, 2H), 8.05 – 8.01 (m, 2H), 5.50 (ddd, *J* = 17.1, 10.3, 6.8 Hz, 1H), 5.01 – 4.95 (m, 2H), 4.59 (d, *J* = 8.2 Hz, 1H), 3.91 – 3.82 (m, 1H), 1.53 – 1.45 (m, 2H), 1.31 – 1.17 (m, 10H), 0.86 (t, *J* = 6.9 Hz, 3H) ppm; **R_f** (pentane/EtOAc, 4:1) = 0.47; $[\alpha]_D^{22}$ = -27.5 (*c* = 0.60, $CHCl_3$); **Chiral HPLC** (Chiralpak IB, 4.6 x 150 mm, 3 µm, Hexane/*i*PrOH 95:5, 1.0 mL/min, 35 °C, 254 nm) t_r (major) = 9.60 min, t_r (minor) = 11.20 min, 92:8 er.

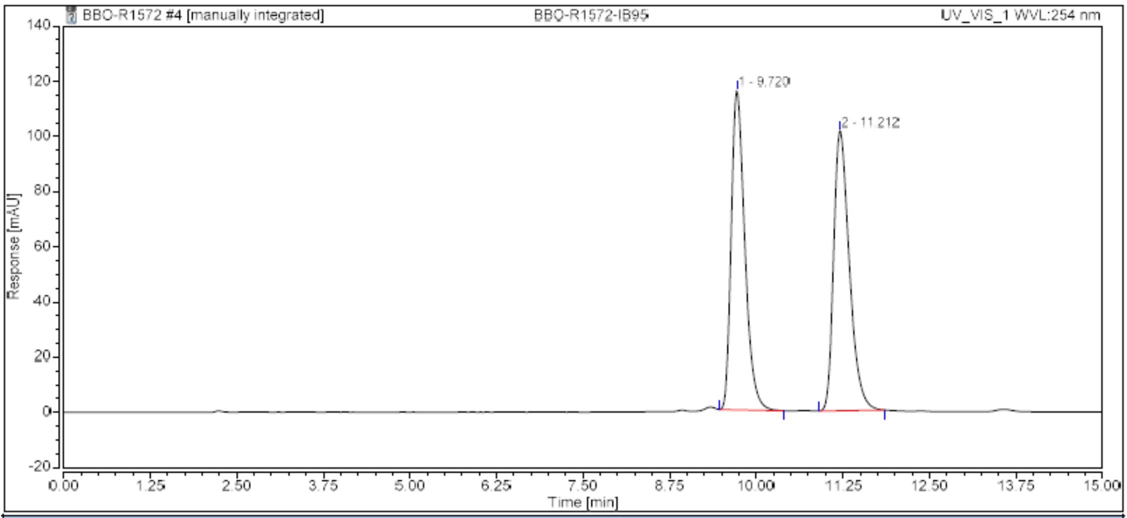

Integration Results

No.	Peak Name	Retention Time min	Area mAU*min	Height mAU	Relative Area %	Relative Height %	Amount n.a.
1		9.720	26.224	115.852	49.67	53.31	n.a.
2		11.212	26.576	101.471	50.33	46.69	n.a.

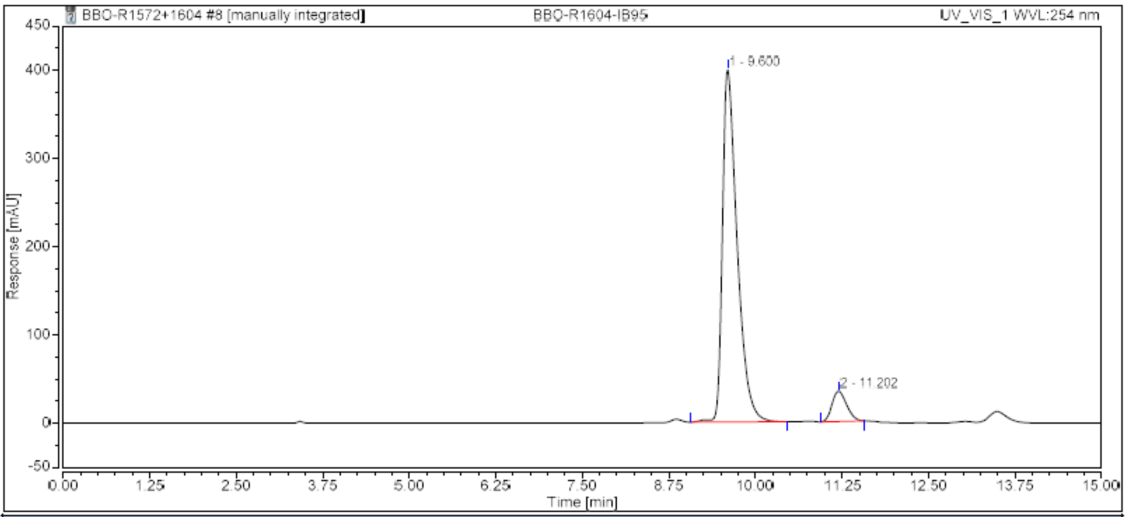

Integration Results

No.	Peak Name	Retention Time min	Area mAU*min	Height mAU	Relative Area %	Relative Height %	Amount n.a.
1		9.600	98.390	399.134	92.17	92.08	n.a.
2		11.202	8.358	34.336	7.83	7.92	n.a.

(*S*)-*N*-(1-Phenylallyl)benzenesulfonamide (**3i**)

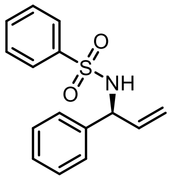

According to *General Procedure 7b*, starting from **1a** and **2b** (scale: 105 µmol), allylic amine (*S*)-**3i** (25.0 mg, 92 µmol, 87% yield, 97:3 er) was obtained as a white solid with the characterization data matching those previously reported.[31] Purification was performed by Prep. TLC on silica (pentane/EtOAc = 7:3).

^{1}**H NMR** (400 MHz, $CDCl_3$) δ = 7.77 – 7.73 (m, 2H), 7.53 – 7.48 (m, 1H), 7.43 – 7.37 (m, 2H), 7.24 – 7.19 (m, 3H), 7.12 – 7.06 (m, 2H), 5.87 (ddd, *J* = 16.5, 10.3, 5.8 Hz, 1H), 5.17 – 5.09 (m, 2H), 5.01 – 4.94 (m, 1H), 4.80 (d, *J* = 7.3 Hz, 1H) ppm; **R$_f$** (pentane/EtOAc, 4:1) = 0.32; $[\alpha]_D^{22}$ = -42.2 (*c* = 0.73, $CHCl_3$); **Chiral HPLC** (Chiralpak IH, 4.6 x 150 mm, 3 µm, Hexane/*i*PrOH 80:20, 1.0 mL/min, 35 °C, 210 nm) t_r (minor) = 10.15 min, t_r (major) = 11.30 min, 97:3 er.

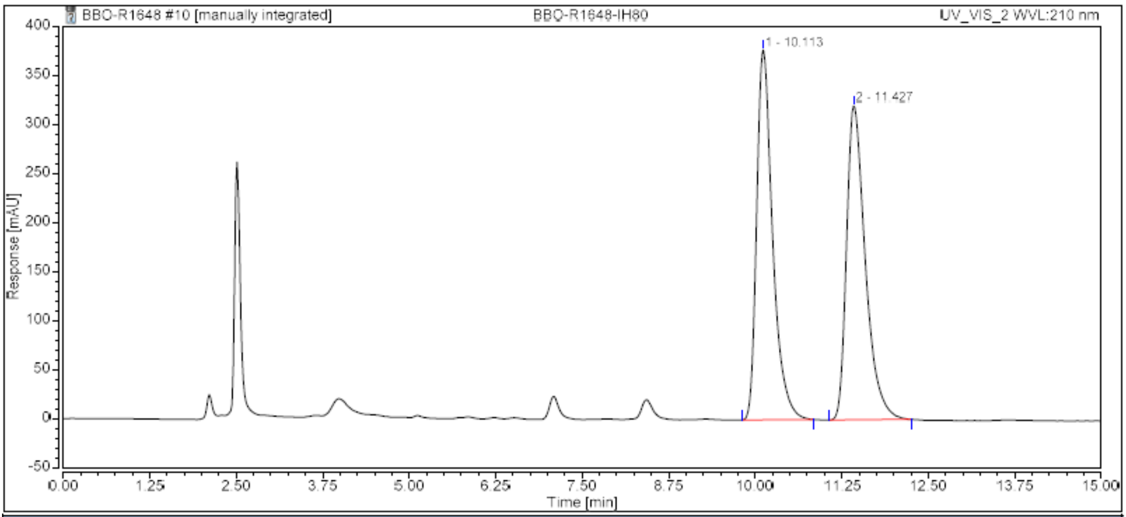

Integration Results

No.	Peak Name	Retention Time min	Area mAU*min	Height mAU	Relative Area %	Relative Height %	Amount n.a.
1		10.113	100.660	376.827	49.94	54.05	n.a.
2		11.427	100.901	320.355	50.06	45.95	n.a.

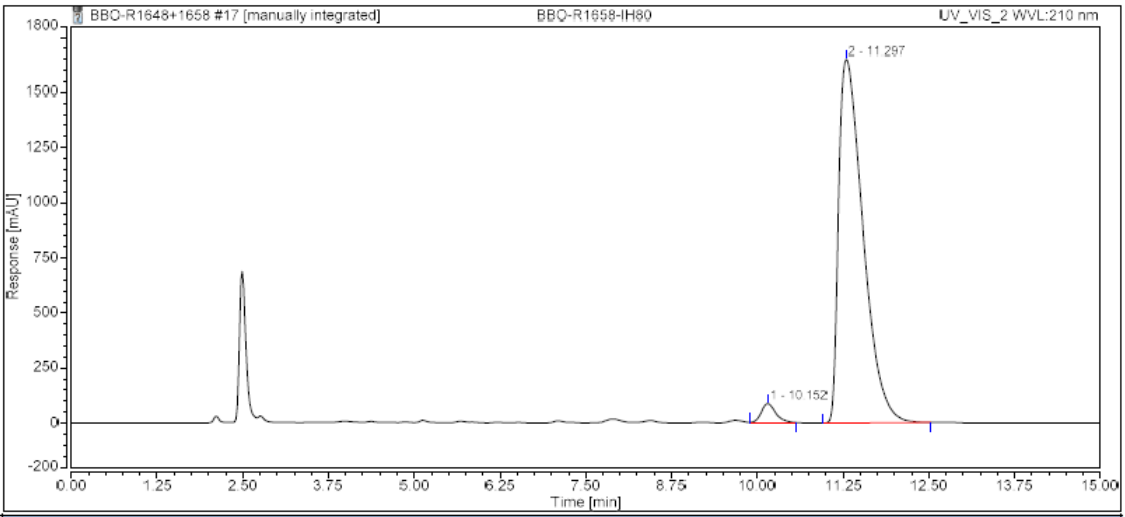

Integration Results

No.	Peak Name	Retention Time min	Area mAU*min	Height mAU	Relative Area %	Relative Height %	Amount n.a.
1		10.152	20.398	84.754	2.92	4.89	n.a.
2		11.297	678.556	1649.054	97.08	95.11	n.a.

(*S*)-4-(*tert*-Butyl)-*N*-(1-phenylallyl)benzenesulfonamide (**3j**)

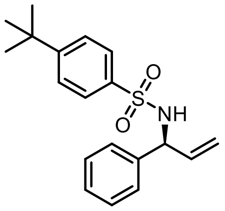

According to *General Procedure 7b*, starting from **1a** and **2c** (scale: 107 µmol), allylic amine (*S*)-**3j** (32.7 mg, 99 µmol, 92% yield, 97:3 er) was obtained as a white solid. Purification was performed by Prep. TLC on silica (pentane/EtOAc = 4:1).

^{1}H NMR (400 MHz, $CDCl_3$) δ = 7.65 – 7.61 (m, 2H), 7.39 – 7.35 (m, 2H), 7.20 – 7.14 (m, 3H), 7.08 – 7.02 (m, 2H), 5.89 (ddd, *J* = 16.9, 10.4, 5.6 Hz, 1H), 5.17 – 5.09 (m, 2H), 4.98 – 4.93 (m, 1H), 4.88 (d, *J* = 7.5 Hz, 1H), 1.31 (s, 9H) ppm; **^{13}C{^{1}H} NMR** (101 MHz, $CDCl_3$) δ = 156.4, 139.4, 137.6, 137.3, 128.7, 127.9, 127.23, 127.17, 125.9, 117.0, 60.0, 35.2, 31.2 ppm; **IR** (ATR) ṽ = 3273 (w), 2963 (w), 1455 (w), 1431 (w), 1399 (w), 1327 (m), 1292 (w), 1161 (s), 1112 (m), 1088 (w), 1050 (w), 1026 (w), 924 (w), 835 (w), 752 (w), 700 (m), 675 (w), 633 (m), 584 (w), 548 (w) cm^{-1}; **HRMS** (ESI/QTOF) *m/z* = calcd. for $[C_{19}H_{23}NNaO_2S]^+$, $[M+Na]^+$: 352.1342, found: 352.1340; **R$_f$** (pentane/EtOAc, 4:1) = 0.47; **M.p.** = 71-72 °C; $[\alpha]_D^{22}$ = -32.0 (*c* = 0.75, $CHCl_3$); **Chiral HPLC** (Chiralpak IB, 4.6 x 150 mm, 3 µm, Hexane/*i*PrOH 97:3, 1.0 mL/min, 35 °C, 210 nm) t_r (minor) = 10.34 min, t_r (major) = 11.33 min, 97:3 er.

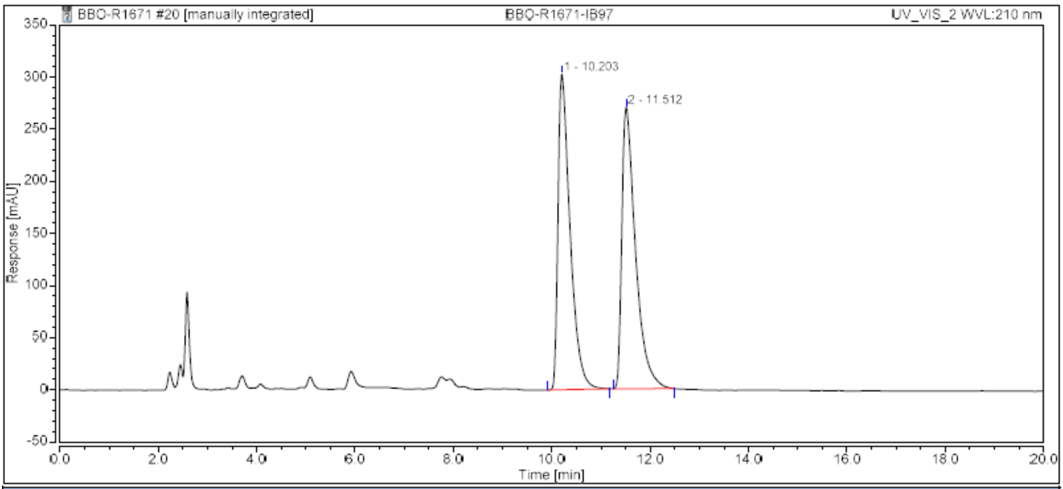

Integration Results							
No.	Peak Name	Retention Time min	Area mAU*min	Height mAU	Relative Area %	Relative Height %	Amount n.a.
1		10.203	86.136	302.710	49.83	52.88	n.a.
2		11.512	86.713	269.737	50.17	47.12	n.a.

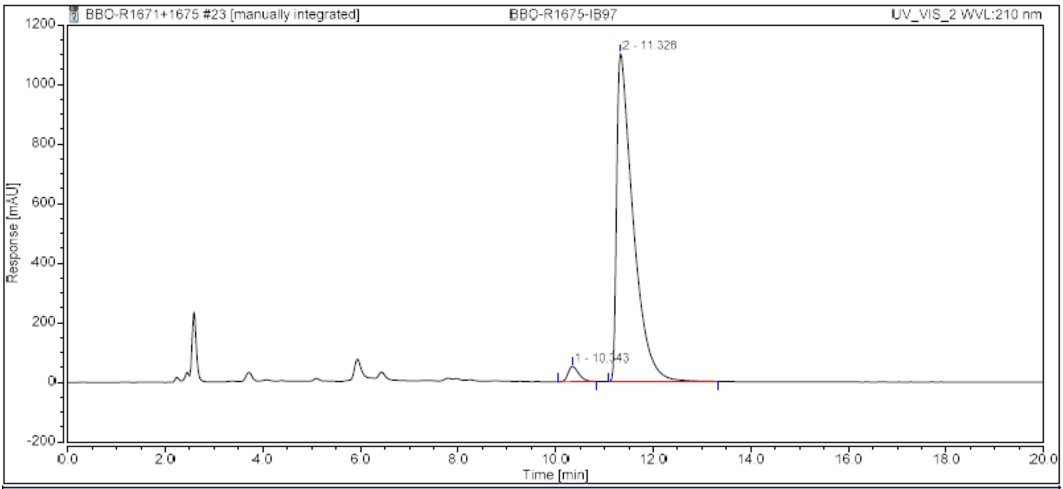

Integration Results							
No.	Peak Name	Retention Time min	Area mAU*min	Height mAU	Relative Area %	Relative Height %	Amount n.a.
1		10.343	12.686	51.538	2.85	4.47	n.a.
2		11.328	431.670	1102.420	97.15	95.53	n.a.

<u>(*S*)-4-Chloro-*N*-(1-phenylallyl)benzenesulfonamide</u> (**3k**)

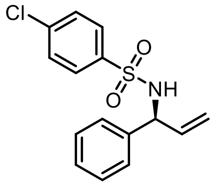

According to *General Procedure 7b*, starting from **1a** and **2d** (scale: 108 µmol), allylic amine (*S*)-**3k** (29.0 mg, 94 µmol, 87% yield, 97.5:2.5 er) was obtained as a white solid with the characterization data matching those previously reported.[29] Purification was performed by Prep. TLC on silica (pentane/EtOAc = 4:1).

^{1}H NMR (400 MHz, $CDCl_3$) δ = 7.66 – 7.62 (m, 2H), 7.37 – 7.32 (m, 2H), 7.25 – 7.20 (m, 3H), 7.12 – 7.06 (m, 2H), 5.88 (ddd, *J* = 16.6, 10.3, 5.7 Hz, 1H), 5.20 – 5.10 (m, 2H), 5.02 – 4.96 (m, 1H), 4.90 (d, *J* = 7.4 Hz, 1H) ppm; **R$_f$** (pentane/EtOAc, 4:1) = 0.42; $[\alpha]_D^{22}$ = -32.1 (*c* = 0.53, $CHCl_3$); **Chiral HPLC** (Chiralpak IH, 4.6 x 150 mm, 3 µm, Hexane/*i*PrOH 80:20, 1.0 mL/min, 35 °C, 254 nm) t_r (minor) = 8.33 min, t_r (major) = 9.10 min, 97.5:2.5 er.

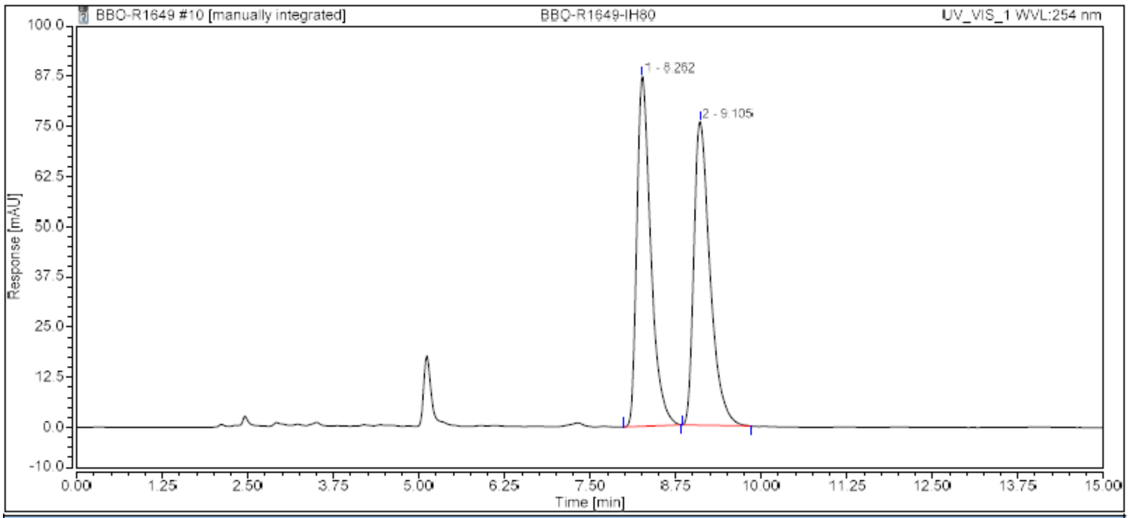

Integration Results

No.	Peak Name	Retention Time min	Area mAU*min	Height mAU	Relative Area %	Relative Height %	Amount n.a.
1		8.262	20.606	87.225	49.85	53.56	n.a.
2		9.105	20.729	75.644	50.15	46.44	n.a.

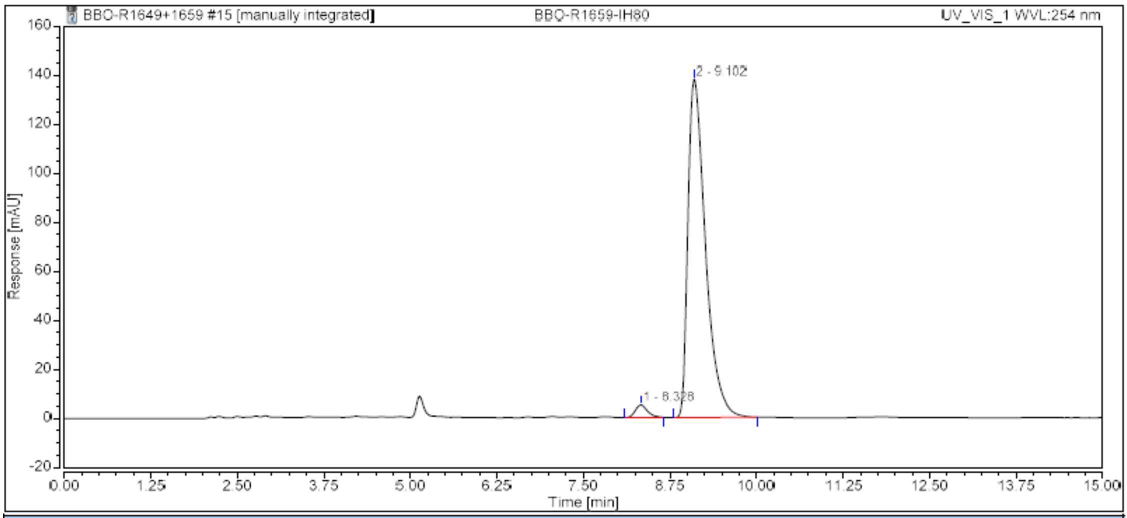

Integration Results

No.	Peak Name	Retention Time min	Area mAU*min	Height mAU	Relative Area %	Relative Height %	Amount n.a.
1		8.328	1.058	5.142	2.55	3.59	n.a.
2		9.102	40.406	138.008	97.45	96.41	n.a.

(*S*)-4-Methoxy-*N*-(1-phenylallyl)benzenesulfonamide (**3l**)

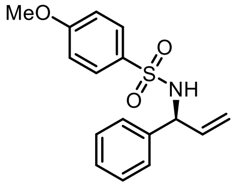

According to *General Procedure 7b*, starting from **1a** and **2e** (scale: 105 µmol), allylic amine (*S*)-**3l** (16.9 mg, 56 µmol, 53% yield, 98:2 er) was obtained as a white solid with the characterization data matching those previously reported.[29] Purification was performed by Prep. TLC on silica (pentane/EtOAc = 7:3).

^{1}H NMR (400 MHz, $CDCl_3$) δ = 7.70 – 7.65 (m, 2H), 7.25 – 7.20 (m, 3H), 7.13 – 7.08 (m, 2H), 6.89 – 6.84 (m, 2H), 5.87 (ddd, *J* = 16.6, 10.4, 5.9 Hz, 1H), 5.17 – 5.09 (m, 2H), 4.93 (t, *J* = 6.5 Hz, 1H), 4.75 (d, *J* = 7.2 Hz, 1H), 3.85 (s, 3H) ppm; **R_f** (pentane/EtOAc, 7:3) = 0.41; $[\alpha]_D^{22}$ = -43.0 (*c* = 0.45, $CHCl_3$); **Chiral HPLC** (Chiralpak IB, 4.6 x 150 mm, 3 µm, Hexane/*i*PrOH 97:3, 1.0 mL/min, 35 °C, 254 nm) t_r (minor) = 21.69 min, t_r (major) = 23.05 min, 98:2 er.

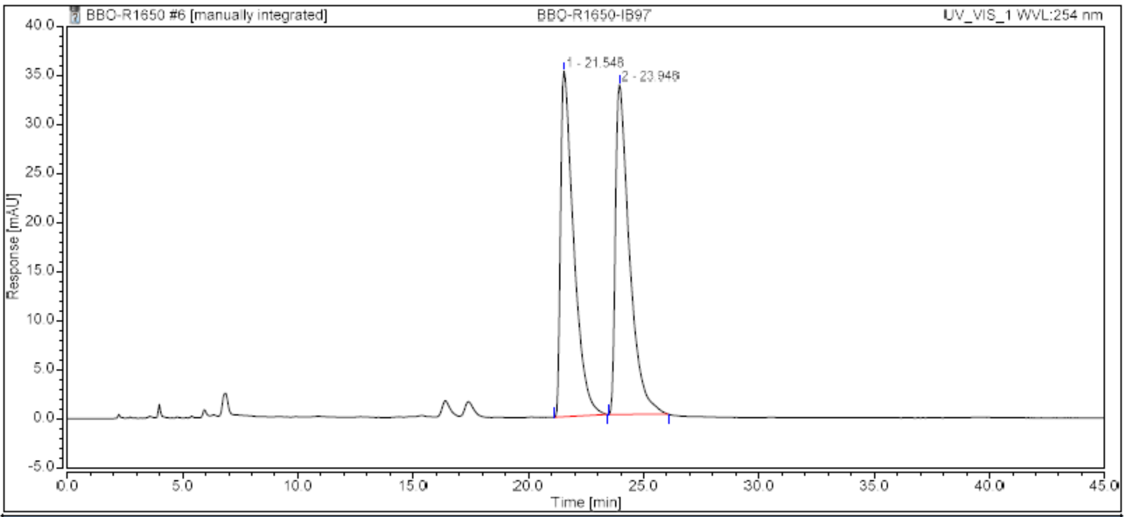

Integration Results

No.	Peak Name	Retention Time min	Area mAU*min	Height mAU	Relative Area %	Relative Height %	Amount n.a.
1		21.548	23.280	35.218	49.57	51.15	n.a.
2		23.948	23.687	33.637	50.43	48.85	n.a.

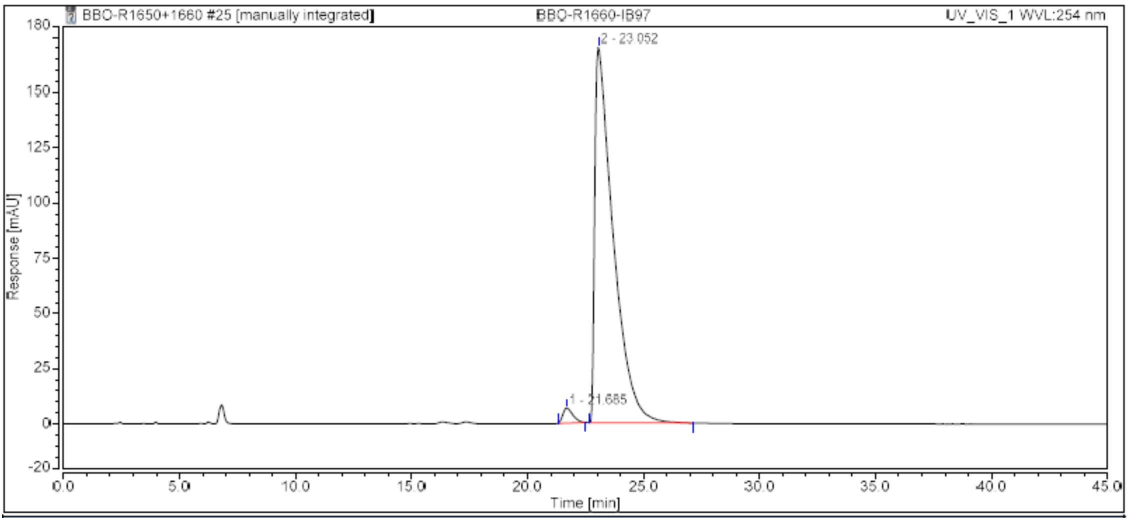

Integration Results

No.	Peak Name	Retention Time min	Area mAU*min	Height mAU	Relative Area %	Relative Height %	Amount n.a.
1		21.685	3.335	6.823	2.09	3.86	n.a.
2		23.052	155.861	170.051	97.91	96.14	n.a.

(*S*)-*N*-(1-Phenylallyl)-4-(trifluoromethoxy)benzenesulfonamide (**3m**)

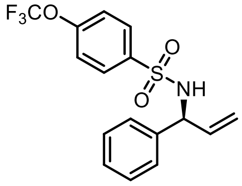

According to *General Procedure 7b*, starting from **1a** and **2f** (scale: 104 µmol), allylic amine (*S*)-**3m** (34.5 mg, 97 µmol, 93% yield, 97:3 er) was obtained as a colourless oil. Purification was performed by Prep. TLC on silica (pentane/EtOAc = 4:1).

^{1}H NMR (400 MHz, $CDCl_3$) δ = 7.74 – 7.69 (m, 2H), 7.21 – 7.14 (m, 5H), 7.08 – 7.02 (m, 2H), 5.94 – 5.84 (m, 1H), 5.19 – 5.09 (m, 2H), 5.04 – 4.98 (m, 2H) ppm; **^{13}C{^{1}H} NMR** (101 MHz, $CDCl_3$) δ = 152.0 (q, *J* = 1.8 Hz), 139.3, 138.8, 137.0, 129.4, 128.8, 128.1, 127.3, 120.8, 120.3 (q, *J* = 259.2 Hz), 117.3, 60.2 ppm; **^{19}F{^{1}H} NMR** (376 MHz, $CDCl_3$) δ = -57.74 ppm; **IR** (ATR) ṽ = 3277 (w), 1331 (w), 1297 (w), 1253 (m), 1210 (m), 1156 (s), 1093 (w), 926 (w), 701 (m), 670 (w), 609 (w), 573 (w) cm^{-1}; **HRMS** (nanochip-ESI/LTQ-Orbitrap) *m/z* = calcd. for $[C_{16}H_{14}F_3NNaO_3S]^+$, $[M+Na]^+$: 380.0539, found: 380.0538; **R$_f$** (pentane/EtOAc, 4:1) = 0.50; $[\alpha]_D^{22}$ = -19.9 (*c* = 0.52, $CHCl_3$); **Chiral HPLC** (Chiralpak IB, 4.6 x 150 mm, 3 µm, Hexane/*i*PrOH 95:5, 1.0 mL/min, 35 °C, 210 nm) t$_r$ (minor) = 7.40 min, t$_r$ (major) = 9.43 min, 97:3 er.

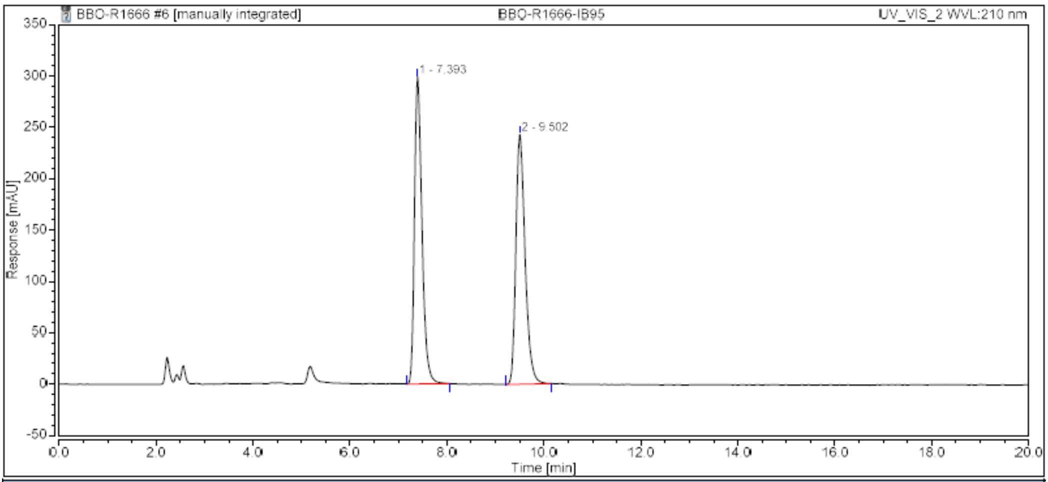

Integration Results

No.	Peak Name	Retention Time min	Area mAU*min	Height mAU	Relative Area %	Relative Height %	Amount n.a.
1		7.393	53.495	298.493	50.04	55.11	n.a.
2		9.502	53.404	243.117	49.96	44.89	n.a.

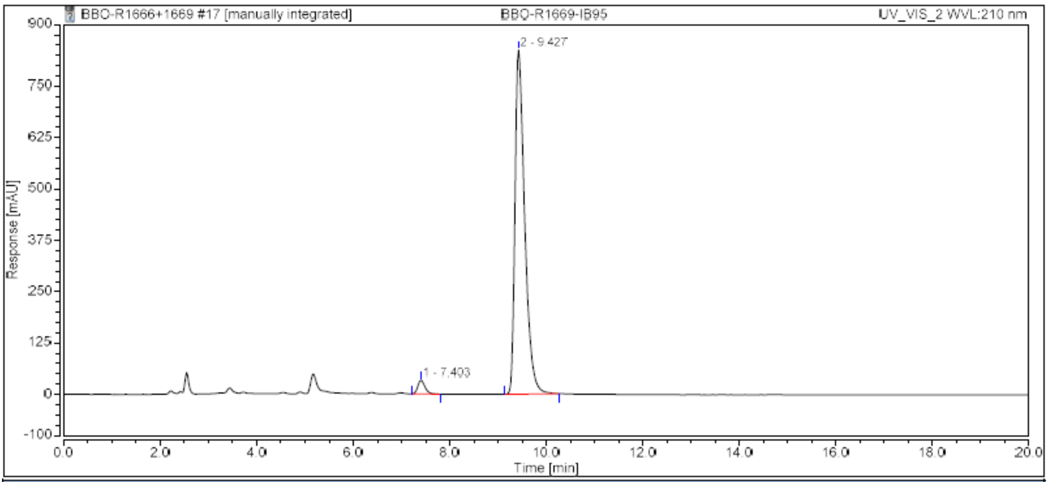

Integration Results

No.	Peak Name	Retention Time min	Area mAU*min	Height mAU	Relative Area %	Relative Height %	Amount n.a.
1		7.403	5.811	33.486	2.89	3.84	n.a.
2		9.427	195.375	837.949	97.11	96.16	n.a.

<u>(*S*)-*N*-(1-Phenylallyl)naphthalene-2-sulfonamide</u> (**3n**)

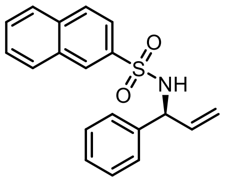

According to *General Procedure 7b*, starting from **1a** and **2g** (scale: 112 µmol), allylic amine (*S*)-**3n** (35.4 mg, 110 µmol, 98% yield, 97:3 er) was obtained as a white solid. Purification was performed by Prep. TLC on silica (pentane/EtOAc = 7:3).

^{1}H NMR (400 MHz, $CDCl_3$) δ = 8.30 (d, *J* = 1.8 Hz, 1H), 7.90 – 7.82 (m, 3H), 7.72 (dd, *J* = 8.7, 1.9 Hz, 1H), 7.66 – 7.55 (m, 2H), 7.17 – 7.06 (m, 5H), 5.87 (ddd, *J* = 17.1, 10.2, 5.5 Hz, 1H), 5.16 – 5.09 (m, 2H), 5.05 – 4.97 (m, 2H) ppm; **^{13}C{^{1}H} NMR** (101 MHz, $CDCl_3$) δ = 139.3, 137.5, 137.2, 134.8, 132.1, 129.4, 129.3, 128.84, 128.79, 128.7, 127.99, 127.96, 127.5, 127.2, 122.5, 117.1, 60.2 ppm; **IR** (ATR) ṽ = 3269 (w), 1323 (m), 1155 (s), 1131 (m), 1075 (m), 954 (w), 925 (w), 816 (w), 748 (m), 700 (m), 678 (m), 660 (m), 642 (w), 618 (w), 572 (w), 548 (m), 476 (w) cm^{-1}; **HRMS** (ESI/QTOF) *m/z* = calcd. for $[C_{19}H_{17}NNaO_2S]^+$, $[M+Na]^+$: 346.0872, found: 346.0877; **R$_f$** (pentane/EtOAc, 7:3) = 0.50; **M.p.** = 119-120 °C; $[\boldsymbol{\alpha}]_{\mathbf{D}}^{\mathbf{22}}$ = -65.6 (*c* = 0.62, $CHCl_3$); **Chiral HPLC** (Chiralpak IB, 4.6 x 150 mm, 3 µm, Hexane/*i*PrOH 95:5, 1.0 mL/min, 35 °C, 254 nm) t$_r$ (minor) = 10.99 min, t$_r$ (major) = 12.29 min, 97:3 er.

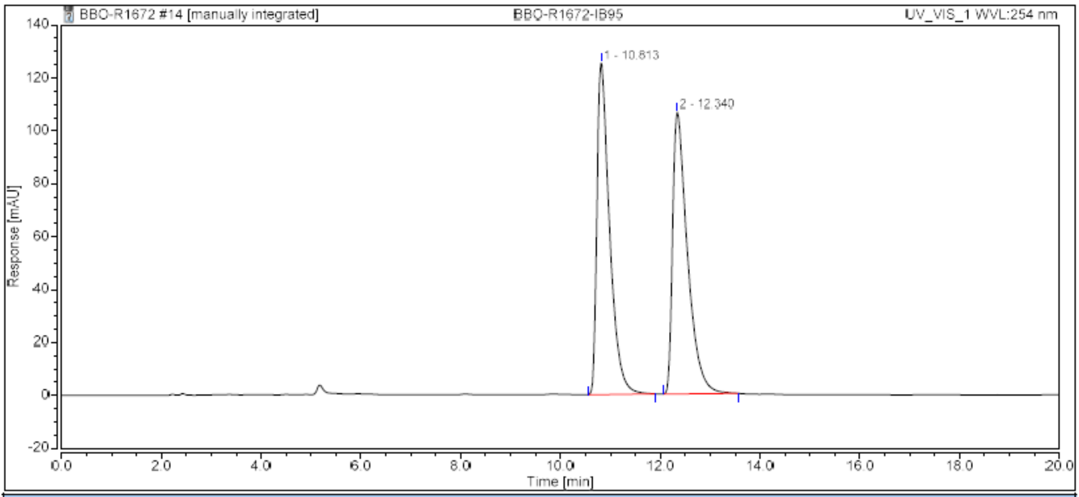

Integration Results

No.	Peak Name	Retention Time min	Area mAU*min	Height mAU	Relative Area %	Relative Height %	Amount n.a.
1		10.813	36.788	125.372	50.06	54.06	n.a.
2		12.340	36.703	106.542	49.94	45.94	n.a.

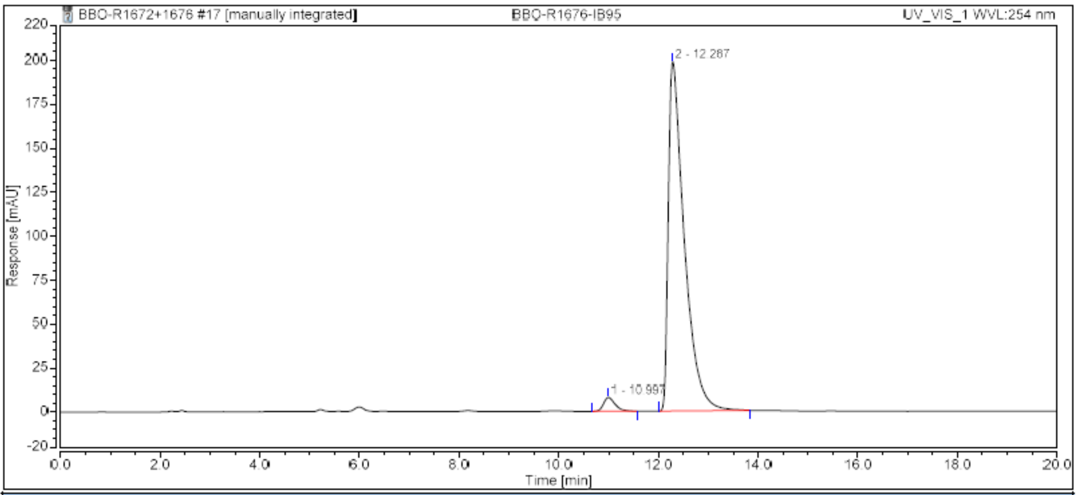

Integration Results

No.	Peak Name	Retention Time min	Area mAU*min	Height mAU	Relative Area %	Relative Height %	Amount n.a.
1		10.997	2.090	7.867	2.76	3.81	n.a.
2		12.287	73.583	198.547	97.24	96.19	n.a.

(*S*)-*N*-(1-Phenylallyl)thiophene-2-sulfonamide (**3o**)

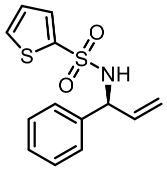

According to *General Procedure 7b*, starting from **1a** and **2h** (scale: 105 µmol), allylic amine (*S*)-**3o** (23.0 mg, 82 µmol, 78% yield, 97:3 er) was obtained as a white solid. Purification was performed by Prep. TLC on silica (pentane/EtOAc = 7:3).

^{1}H NMR (400 MHz, $CDCl_3$) δ = 7.53 – 7.50 (m, 1H), 7.49 – 7.46 (m, 1H), 7.30 – 7.23 (m, 3H), 7.17 – 7.12 (m, 2H), 6.99 – 6.96 (m, 1H), 5.92 (ddd, *J* = 16.5, 10.5, 5.7 Hz, 1H), 5.21 – 5.14 (m, 2H), 5.06 – 5.00 (m, 1H), 4.91 (d, *J* = 7.5 Hz, 1H) ppm; **^{13}C{^{1}H} NMR** (101 MHz, $CDCl_3$) δ = 141.8, 139.2, 137.0, 132.6, 132.0, 128.9, 128.1, 127.3, 127.2, 117.2, 60.3 ppm; **IR** (ATR) ṽ = 3275 (w), 1405 (w), 1329 (m), 1154 (s), 1092 (w), 1047 (w), 1018 (m), 927 (w), 721 (w), 700 (m), 679 (w), 596 (w), 577 (w) cm^{-1}; **HRMS** (nanochip-ESI/LTQ-Orbitrap) *m/z* = calcd. for $[C_{13}H_{13}NNaO_2S_2]^+$, $[M+Na]^+$: 302.0280, found: 302.0278; **R$_f$** (pentane/EtOAc, 4:1) = 0.35; **M.p.** = 48-49 °C; $[\alpha]_D^{22}$ = -27.5 (*c* = 0.77, $CHCl_3$); **Chiral HPLC** (Chiralpak IH, 4.6 x 150 mm, 3 µm, Hexane/*i*PrOH 80:20, 1.0 mL/min, 35 °C, 254 nm) t_r (minor) = 9.79 min, t_r (major) = 12.21 min, 97:3 er.

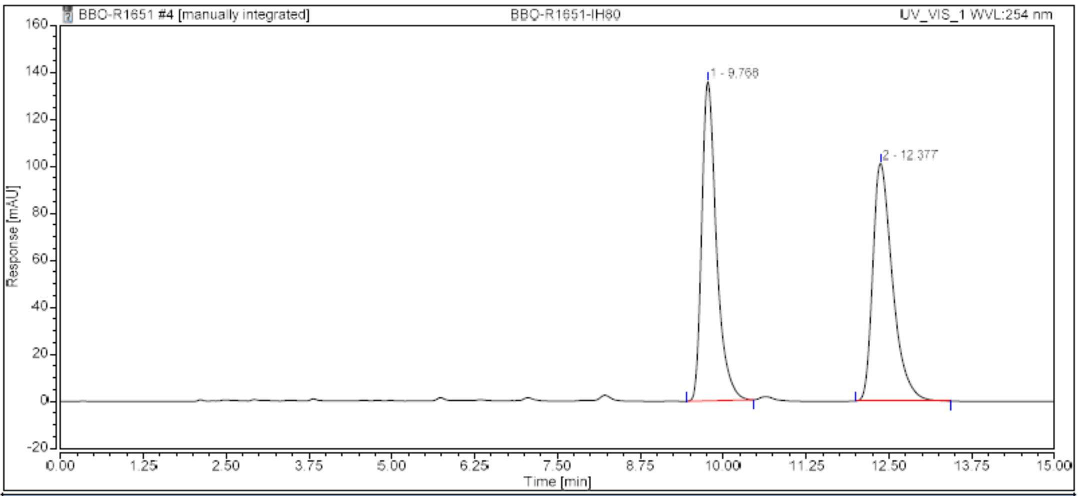

Integration Results

No.	Peak Name	Retention Time min	Area mAU*min	Height mAU	Relative Area %	Relative Height %	Amount n.a.
1		9.768	34.802	135.906	49.88	57.32	n.a.
2		12.377	34.964	101.213	50.12	42.68	n.a.

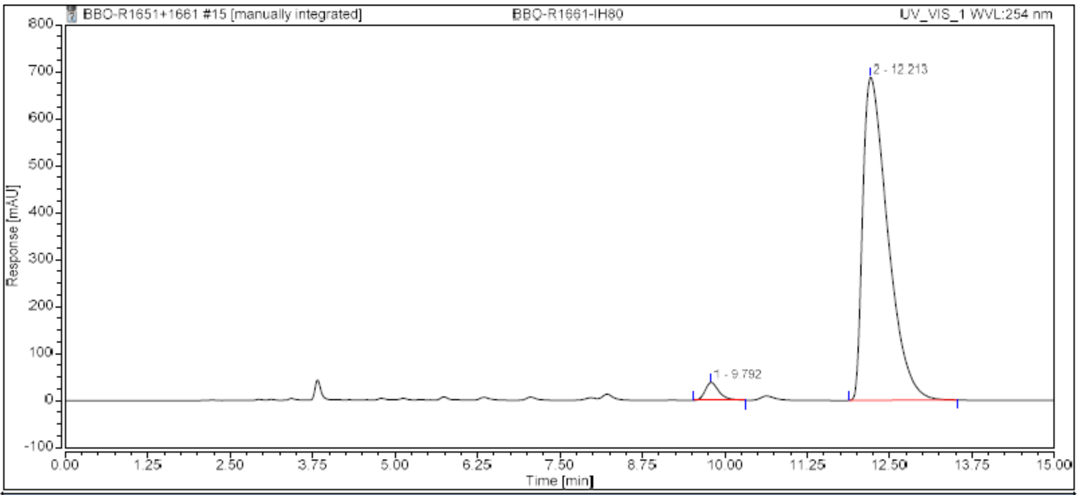

Integration Results

No.	Peak Name	Retention Time min	Area mAU*min	Height mAU	Relative Area %	Relative Height %	Amount n.a.
1		9.792	9.250	37.993	2.98	5.23	n.a.
2		12.213	301.563	687.993	97.02	94.77	n.a.

(*S*)-*N*-(1-Phenylallyl)cyclopropanesulfonamide (**3p**)

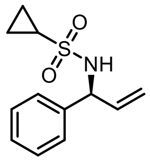

According to *General Procedure 7b*, starting from **1a** and **2i** (scale: 150 µmol), allylic amine (*S*)-**3p** (28.9 mg, 122 µmol, 81% yield, 97.5:2.5 er) was obtained as a colourless oil. Purification was performed by Prep. TLC on silica (pentane/EtOAc = 7:3). Notably, the compound is only very weakly visible under UV light (254 nm), and a PMA stain helps with visualization.

^{1}H NMR (400 MHz, $CDCl_3$) δ = 7.40 – 7.28 (m, 5H), 6.04 (ddd, *J* = 16.9, 10.3, 5.8 Hz, 1H), 5.33 – 5.26 (m, 2H), 5.15 – 5.09 (m, 1H), 4.71 (d, *J* = 7.4 Hz, 1H), 2.22 – 2.14 (m, 1H), 1.15 – 1.01 (m, 2H), 0.88 – 0.73 (m, 2H) ppm; **^{13}C{^{1}H} NMR** (101 MHz, $CDCl_3$) δ = 140.3, 138.0, 129.0, 128.2, 127.3, 117.0, 60.0, 31.8, 6.1, 6.0 ppm; **IR** (ATR) ṽ = 3272 (w), 1454 (w), 1421 (w), 1324 (m), 1301 (m), 1144 (s), 1043 (m), 991 (w), 923 (m), 889 (m), 826 (w), 760 (w), 700 (s), 587 (w), 519 (w) cm^{-1}; **HRMS** (Sicrit plasma/LTQ-Orbitrap) *m*/*z* = calcd. for $[C_{12}H_{14}NO_2S]^+$, $[M\text{-}H^-]^+$: 236.0740, found: 236.0739; **R$_f$** (pentane/EtOAc, 7:3) = 0.40; $[\alpha]_D^{22}$ = -25.6 (*c* = 0.97, $CHCl_3$); **Chiral HPLC** (Chiralpak IB, 4.6 x 150 mm, 3 µm, Hexane/*i*PrOH 97:3, 1.0 mL/min, 35 °C, 210 nm) t_r (minor) = 12.71 min, t_r (major) = 13.58 min, 97.5:2.5 er.

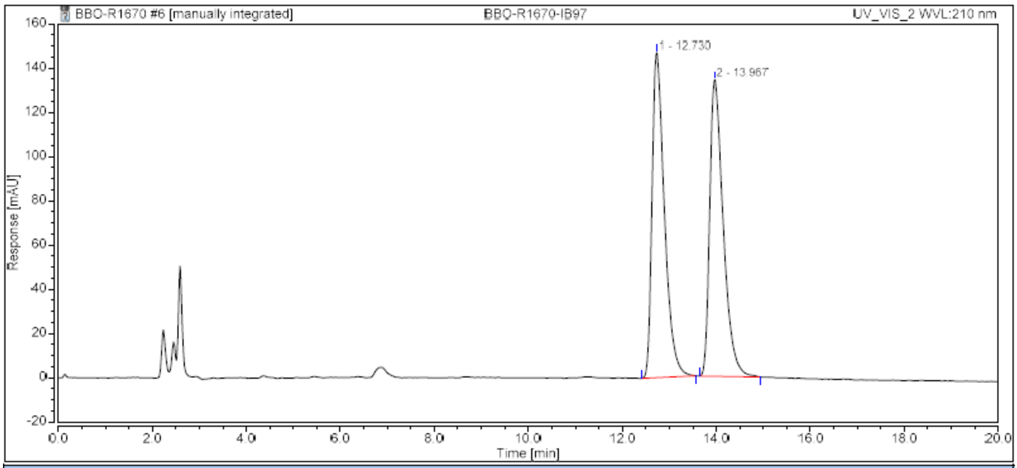

Integration Results							
No.	Peak Name	Retention Time min	Area mAU*min	Height mAU	Relative Area %	Relative Height %	Amount n.a.
1		12.730	44.777	146.904	50.02	52.28	n.a.
2		13.967	44.743	134.105	49.98	47.72	n.a.

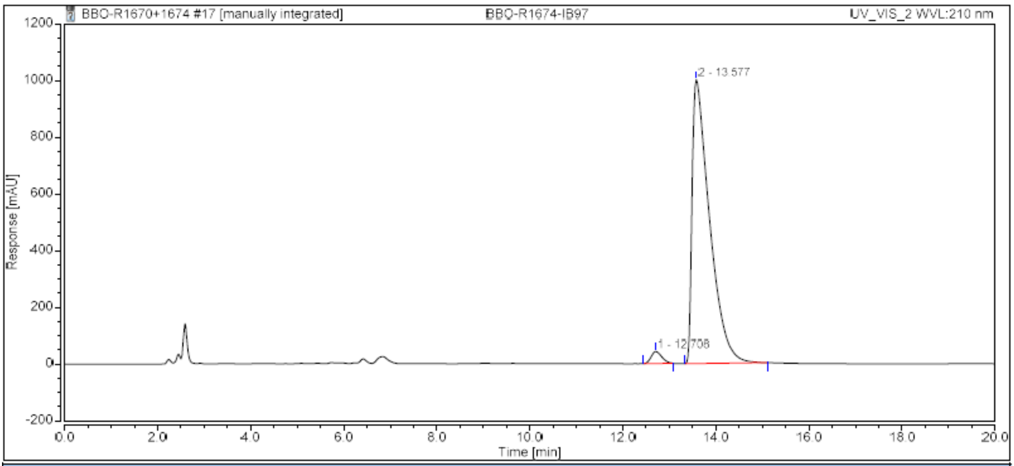

Integration Results							
No.	Peak Name	Retention Time min	Area mAU*min	Height mAU	Relative Area %	Relative Height %	Amount n.a.
1		12.708	11.142	42.463	2.51	4.07	n.a.
2		13.577	432.303	1001.919	97.49	95.93	n.a.

6.2 Rh-catalyzed Dearomative (3 + 2) C-H Spiroannulation of 2-Alkenylphenols

Alkenylphenol substrate **4** was prepared in 1 step from commercial 2-hydroxybenzophenone (CAS 117-99-7) *via* Wittig olefination according to a reported procedure.[32] Diphenylacetylene **5** was commercially available and was used as obtained from the supplier. Racemic spirocycle **6** was prepared according to *General Procedure 8* using achiral complex $[Cp^{*}RhCl_2]_2$ as a catalyst.

***General Procedure 8* – Cp^{V}Rh(III)-Catalyzed Dearomative (3 + 2) C-H Spiroannulation.**

Without protection from oxygen or moisture, an oven-dried microwave vial was charged with **Rh** catalyst (2.5 mol% dimer), copper(II) acetate monohydrate (10.0 mg, 1 equiv.), and diphenylacetylene **5** (8.9 mg, 0.05 mmol). A solution of 2-alkenylphenol **4** (29.4 mg, 3 equiv.) in anhydrous acetonitrile (5 mL/mmol, i.e. 250 µL) was added. The vial was capped under air, and the reaction mixture was stirred in a heating block at 40 °C with an air-filled balloon attached for 24 hours. After cooling to room temperature (25 °C), the reaction was diluted with ethyl acetate and then filtered through a pad of silica gel (3 cm) with ethyl acetate as eluent. After removal of all volatiles *in vacuo*, the resulting crude residue was analyzed by qNMR in $CDCl_3$ with ethylene carbonate as an internal standard. Notably, prior filtration of the $CDCl_3$ solution through a hydrophilic PTFE syringe filter (0.22 µm pore size) removed final traces of copper, thereby ensuring no broadening of the ^{1}H NMR signals. To determine the enantiomeric ratio, purification was performed by Prep. TLC on silica (pentane/DCM/EtOAc = 30:20:1), affording chiral spirocycle **6** as a yellow oil, which was then analyzed by HPLC using a chiral stationary phase (Chiralpak IC, 4.6 x 150 mm, 3 µm, Hexane/*i*PrOH 80:20, 1.0 mL/min, 35 °C, 254 nm).

The results of the catalyst screening via *General Procedure 8* are visualized in **Scheme S29**.

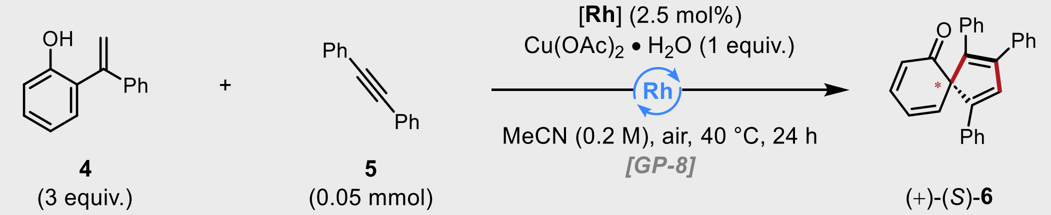

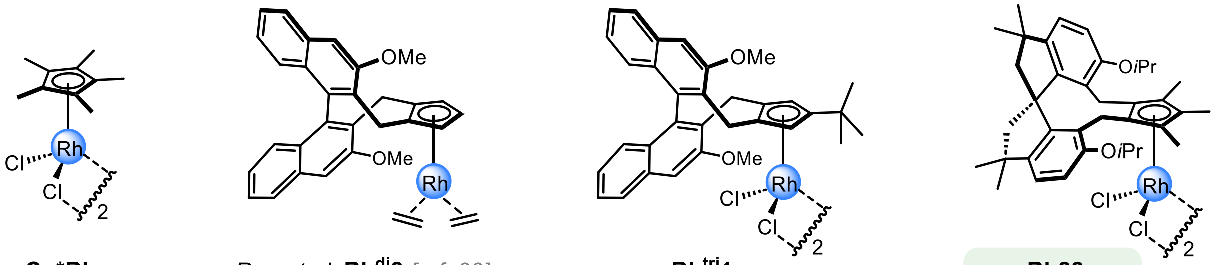

Cp*Rh
93%, rac.

Reported: **Rh^{di}2** *[ref. 33]*
51%, 69:31 er[a]

Rh^{tri}1
86%, 50:50 er

Rh22
98%, 22:78 er

[a] **Conditions:** $Cu(OAc)_2$ (2 equiv.), DMF/*t*AmylOH (1:1, 0.1 M), argon, 60 °C, 44 h, **4** (0.20 mmol), **5** (1.5 equiv.)

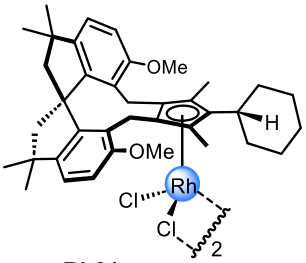

Rh21
78%, 21:79 er

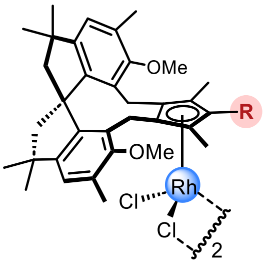

	Frontarm R	% yield	er
**Rh15**	Pen	99	67:33
**Rh16**	*i*Pr	80	56:44
**Rh17**	Ph	49	25:75
**Rh20a**	4-Ph-C_6H_4	69	26:74
**Rh19**	3,4,5-OMe-C_6H_3	27	36:64
**Rh19**[b]	3,4,5-OMe-C_6H_3	45	47:53
**Rh18**	4-CF_3-C_6H_4	64	22:78

[b] With AgOAc (2 equiv.) instead of $Cu(OAc)_2$ • H_2O

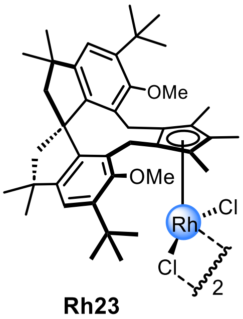

Rh23
88%, 59:41 er

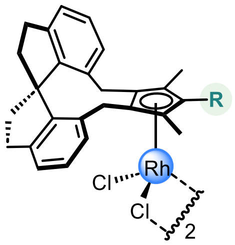

	Frontarm R	% yield	er
**Rh27**	Me	99	29:71
**Rh24**	*i*Pr	89	45:55
**Rh26**	*t*Bu	68	43.57
**Rh28**	Ph	76	28:72
**Rh30**	2,6-F-C_6H_3	38	30:70

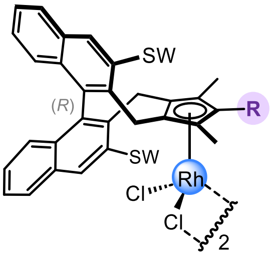

	SW	Frontarm R	% yield	er
**Rh1a**	H	*i*Pr	87	50:50
**Rh7**	H	2,6-OMe-C_6H_3	81	66:34
**Rh8a**	OMe	*i*Pr	62	59:41
**Rh9**	OMe	Pen	96	63:37
**Rh10**	OMe	3,5-*t*Bu-C_6H_3	77	44:56
(S_a)-**Rh13**	Ph	*i*Pr	82	28:72
(S_a)-**Rh14**	Ph	Me	98	33:67

Scheme S29. Screening of Cp^{V}Rh(III) catalysts for the enantioselective dearomative C-H spiroannulation toward **6**.

(*R*)-1,2,4-Triphenylspiro[4.5]deca-1,3,7,9-tetraen-6-one (**6**)

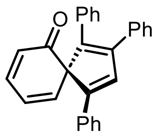

According to *General Procedure 8* (scale: 54 µmol), using (*R*)-**Rh22** as catalyst, chiral spirocycle (*R*)-**6** (19.8 mg, 53 µmol, 98% yield, 78:22 er) was obtained as a yellow oil with the characterization data matching those previously reported.[32,33] Purification was performed by Prep. TLC on silica (pentane/DCM/EtOAc = 30:20:1). The absolute configuration of (−)-**6** was assigned as *R* by comparison of its specific optical rotation with the literature value.[33]

^{1}H NMR (400 MHz, $CDCl_3$) δ = 7.46 (s, 1H), 7.31 – 7.27 (m, 6H), 7.24 – 7.15 (m, 7H), 7.12 – 7.07 (m, 3H), 6.49 (ddd, *J* = 9.2, 6.1, 0.7 Hz, 1H), 6.28 (dt, *J* = 9.9, 0.8 Hz, 1H), 6.23 – 6.19 (m, 1H) ppm; **R_f** (pentane/DCM/EtOAc, 30:20:1) = 0.38; $[\alpha]_D^{22}$ = -36.8 (*c* = 0.67, $CHCl_3$); **Chiral HPLC** (Chiralpak IC, 4.6 x 150 mm, 3 µm, Hexane/*i*PrOH 80:20, 1.0 mL/min, 35 °C, 254 nm) t_r (major) = 5.86 min, t_r (minor) = 8.70 min, 78:22 er.

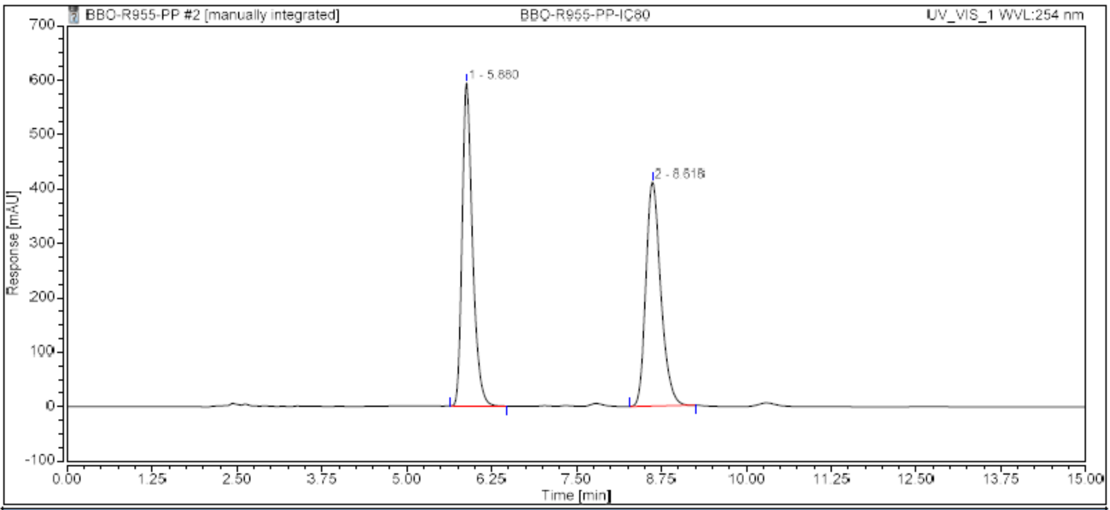

Integration Results

No.	Peak Name	Retention Time min	Area mAU*min	Height mAU	Relative Area %	Relative Height %	Amount n.a.
1		5.880	105.622	593.845	50.19	59.03	n.a.
2		8.618	104.831	412.175	49.81	40.97	n.a.

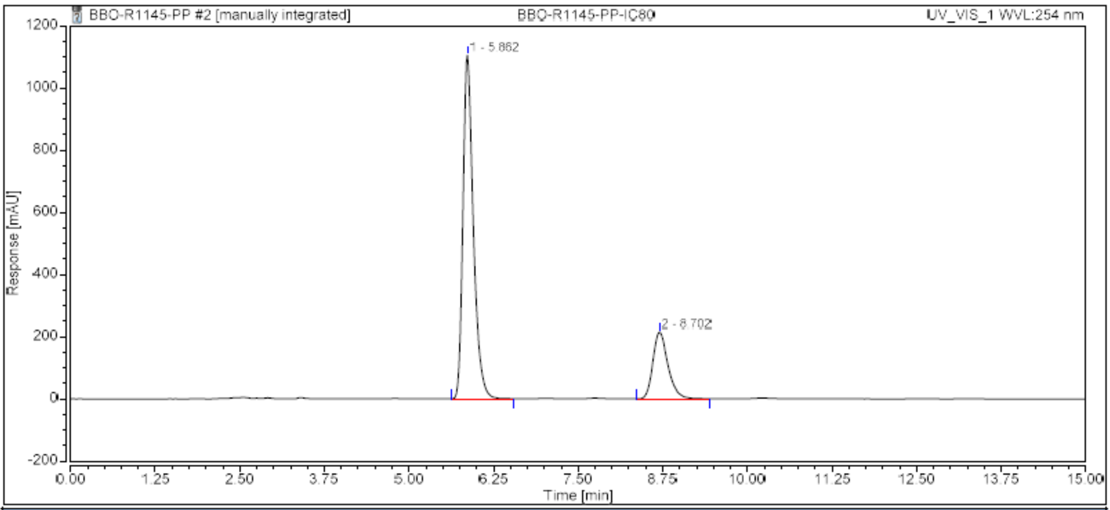

Integration Results

No.	Peak Name	Retention Time min	Area mAU*min	Height mAU	Relative Area %	Relative Height %	Amount n.a.
1		5.862	196.773	1104.734	78.04	83.72	n.a.
2		8.702	55.368	214.897	21.96	16.28	n.a.

6.3 Co-catalyzed (3 + 2) C-H Spiroannulation towards Chiral Benzosultams

N-Sulfonyl ketimine substrate **7** was prepared in 1 step from commercial saccharin (artificial sweetener, CAS 81-07-2) *via* addition–elimination according to a reported procedure.[34] Diphenylacetylene **5** was commercially available, and was used as obtained from the supplier. Chiral acid (R_a)-**9** was prepared in 2 steps from chiral dibromide precursor (R_a)-**L1** according to a reported procedure by Matsunaga.[35] Racemic spirocycle **8** was prepared according to *General Procedure 9* using achiral complex Cp*Co(CO)I_2 as a catalyst.

General Procedure 9 ***– Cp^{V}Co(III)-Catalyzed (3 + 2) C-H Spiroannulation.***

An oven-dried microwave vial was charged with **Co** catalyst (10 mol%), silver(I) hexafluoroantimonate (3.4 mg, 20 mol%), pivalic acid (1.0 mg, 20 mol%), *N*-sulfonyl ketimine **7** (12.2 mg, 0.05 mmol), and diphenylacetylene **5** (17.8 mg, 2 equiv.) under air. The vial was capped and placed under an atmosphere of nitrogen by Schlenk technique. Anhydrous 1,2-dichloroethane (10 mL/mmol, i.e. 500 µL) was added, and the reaction mixture was stirred in a heating block at 120 °C for 24 hours. After cooling to room temperature (25 °C), the reaction was diluted with ethyl acetate and then filtered through a pad of silica gel (3 cm) with ethyl acetate as eluent. After removal of all volatiles *in vacuo*, the resulting crude residue was analyzed by qNMR in $CDCl_3$ with 1,3,5-trimethoxybenzene as an internal standard. Notably, the $CDCl_3$ solution was first passed through a hydrophilic PTFE syringe filter (0.22 µm pore size) before qNMR analysis. To determine the enantiomeric ratio, purification was performed by Prep. TLC on silica (pentane/EtOAc = 7:3), affording chiral spirocycle **8** as a white solid, which was then analyzed by HPLC using a chiral stationary phase (Chiralpak IC, 4.6 x 150 mm, 3 µm, Hexane/*i*PrOH 80:20, 1.0 mL/min, 35 °C, 254 nm).

The results of the catalyst screening via *General Procedure 9* are visualized in **Scheme S30**.

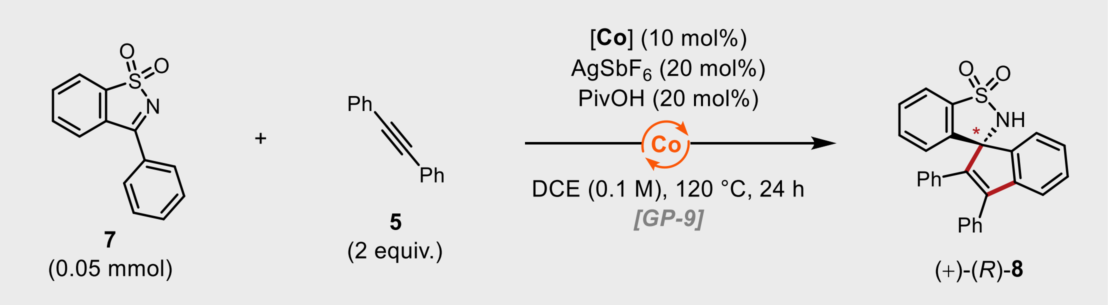

**Deviation** (using **Cp*Co**)	% yield	er
At 80 °C	6	-
At 100 °C	47	-
With (R_a)-**9** instead of PivOH	94	50:50

**Control experiments**	% yield
Without PivOH	8
Without **Co8**	0
Without $AgSbF_6$	0

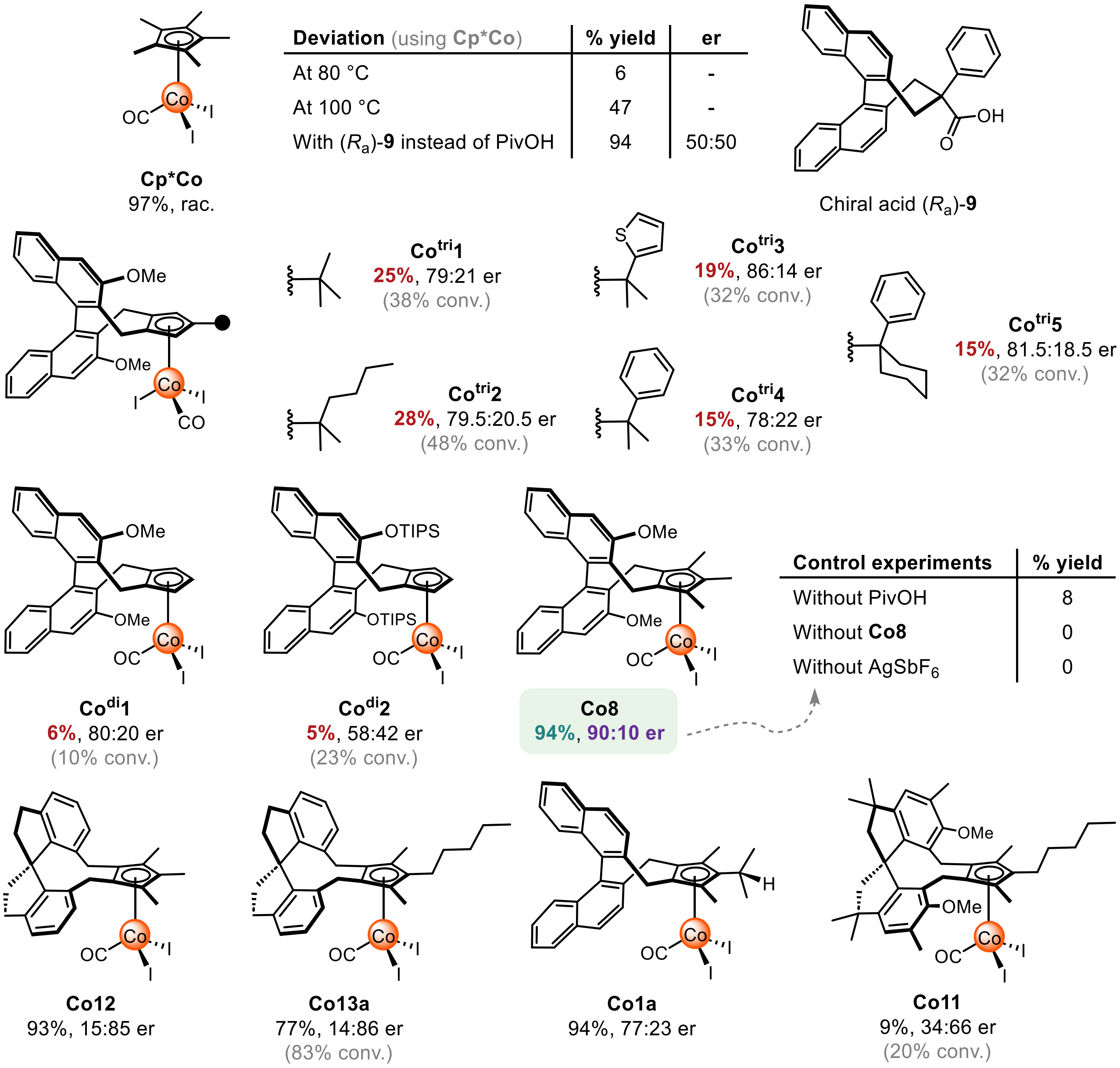

**Deviation** (using **Co13a**)	% yield	er
$AgNTf_2$ instead of $AgSbF_6$, 36 h	23	14:86
$AgBF_4$ instead of $AgSbF_6$, 36 h	19	13:87
AgOTf instead of $AgSbF_6$, 36 h	0	-
**Co13b** instead of **Co13a**, no $AgSbF_6$	10	18:82

Scheme S30. Screening of $Cp^{V}Co(III)$ catalysts for the enantioselective (3 + 2) C-H spiroannulation toward **8**.

(*R*)-2',3'-Diphenyl-2*H*-spiro[benzo[*d*]isothiazole-3,1'-indene] 1,1-dioxide (**8**)

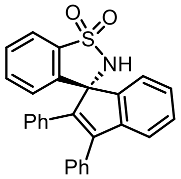

According to *General Procedure 9* (scale: 51 µmol), using (R_a)-**Co8** as catalyst, chiral spirocycle (*R*)-**8** (20.1 mg, 48 µmol, 94% yield, 90:10 er) was obtained as a white crystalline solid with the characterization data matching those previously reported.[36,37] Purification was performed by Prep. TLC on silica (pentane/EtOAc = 7:3). The absolute configuration of (+)-**8** was assigned as *R* by comparison of its specific optical rotation with the literature value.[36]

^{1}H NMR (400 MHz, $CDCl_3$) δ = 7.90 – 7.85 (m, 1H), 7.59 – 7.50 (m, 2H), 7.48 – 7.43 (m, 1H), 7.43 – 7.31 (m, 7H), 7.25 – 7.19 (m, 1H), 7.17 – 7.05 (m, 4H), 6.92 – 6.87 (m, 2H), 4.84 (s, 1H) ppm; **R_f** (pentane/EtOAc, 7:3) = 0.34; $[\alpha]_D^{22}$ = +118.4 (*c* = 0.39, $CHCl_3$); **Chiral HPLC** (Chiralpak IC, 4.6 x 150 mm, 3 µm, Hexane/*i*PrOH 80:20, 1.0 mL/min, 35 °C, 254 nm) t_r (major) = 8.68 min, t_r (minor) = 10.84 min, 90:10 er.

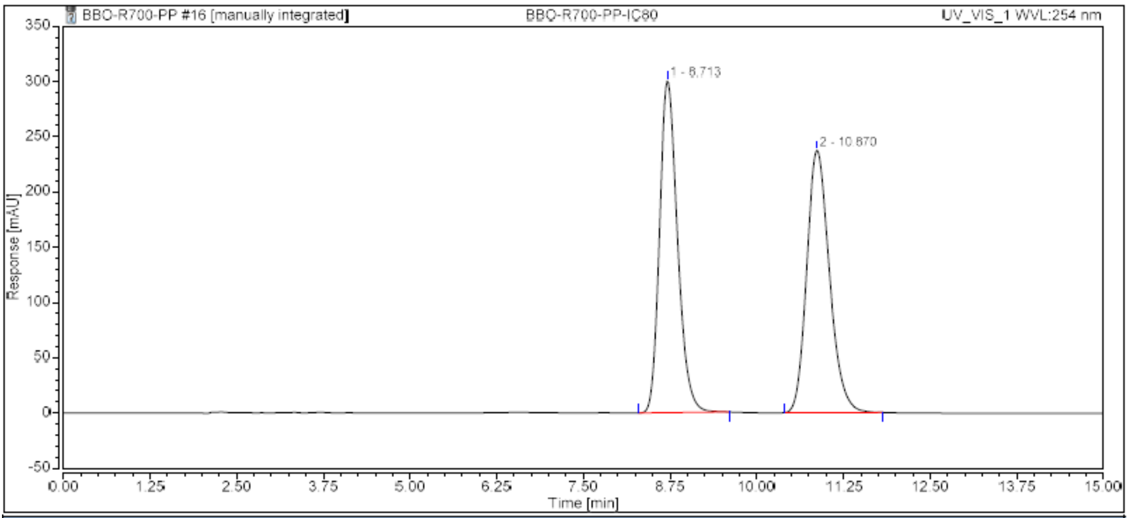

Integration Results

No.	Peak Name	Retention Time min	Area mAU*min	Height mAU	Relative Area %	Relative Height %	Amount n.a.
1		8.713	90.189	300.343	49.98	55.84	n.a.
2		10.870	90.252	237.500	50.02	44.16	n.a.

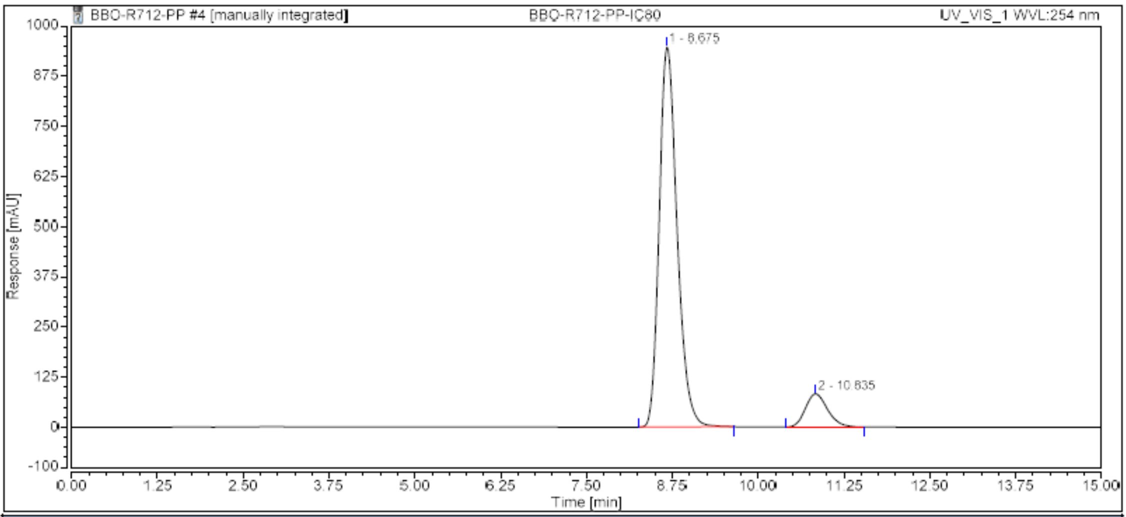

Integration Results

No.	Peak Name	Retention Time min	Area mAU*min	Height mAU	Relative Area %	Relative Height %	Amount n.a.
1		8.675	285.916	947.726	90.21	91.96	n.a.
2		10.835	31.029	82.838	9.79	8.04	n.a.

6.4 Rh-catalyzed (4 + 2) C-H Annulation for the Benzamidation of Cyclopropenes

O-Boc benzhydroxamate ester substrate **10** and its *O*-Piv analog **10’** were prepared in 1 step from commercial benzhydroxamic acid (CAS 495-18-1) according to reported procedures.[38,39] Cyclopropene substrate **11** was prepared in 4 steps from commercial acetophenone (CAS 98-86-2) according to a reported procedure.[40] Racemic cyclopropa[*c*]dihydroisoquinolone **12** was prepared according to *General Procedure 10* using achiral complex $[Cp^{*}RhCl_2]_2$ as a catalyst.

<u>General Procedure 10</u> – Cp^{V}Rh(III)-Catalyzed (4 + 2) C-H Annulation with Cyclopropenes.

Without protection from oxygen or moisture, an oven-dried microwave vial was charged with **Rh** catalyst (2.5 mol% dimer), cesium pivalate (2.9 mg, 25 mol%), and *O*-Boc benzhydroxamate ester **10** (11.9 mg, 0.05 mmol). Anhydrous methanol (5 mL/mmol, i.e. 250 µL) was added, and the mixture was stirred for 1 min before adding a solution of cyclopropene **11** (9.8 mg, 1.5 equiv.) in anhydrous DCM (5 mL/mmol, i.e. 250 µL). The vial was capped under air, and the red reaction mixture was stirred at room temperature (25 °C) for 36 hours. Next, the reaction was diluted with ethyl acetate and then filtered through a pad of silica gel (3 cm) with ethyl acetate as eluent. After removal of all volatiles *in vacuo*, the resulting crude residue was analyzed by qNMR in $CDCl_3$ with 1,3,5-trimethoxybenzene as an internal standard. Notably, the $CDCl_3$ solution was first passed through a hydrophilic PTFE syringe filter (0.22 µm pore size) before qNMR analysis. To determine the enantiomeric ratio, purification was performed by Prep. TLC on silica (pentane/EtOAc = 1:1), affording chiral cyclopropa[*c*]dihydroisoquinolone **12** as a white solid, which was then analyzed by HPLC using a chiral stationary phase (Chiralpak IA, 4.6 x 150 mm, 3 µm, Hexane/*i*PrOH 90:10, 1.0 mL/min, 35 °C, 254 nm).

The results of the catalyst screening via *General Procedure 10* are visualized in **Scheme S31**. Brief finetuning of the reaction conditions was done using catalyst (*R*)-**Rh25** and is visualized in **Scheme S32**.

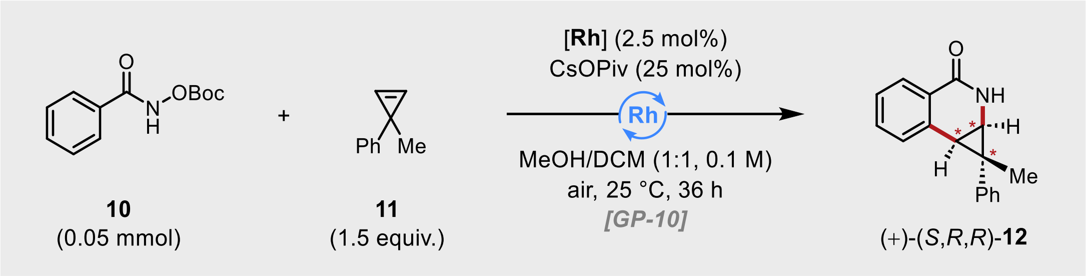

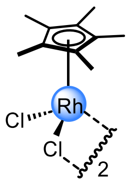

Cp*Rh
81%, 15:1 dr, rac.

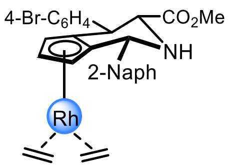

Reported: **Rh^{di}3** *[ref. 41]*
68%, **1:1.1 dr**, 91:9 er[a,b]

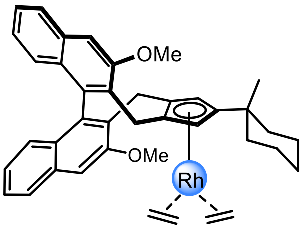

Reported: **Rh^{tri}2** *[ref. 40]*
22%, >20:1 dr, **64.5:35.5 er**[b]

[a] Er of (*S*,*S*,*S*)-diastereomer. [b] With $(BzO)_2$ instead of CsOPiv.

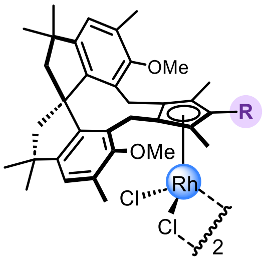

	Frontarm R	% yield	dr	er
**Rh16**	*i*Pr	38	16:1	72.28
**Rh20a**	4-Ph-C_6H_4	25	10:1	63:37
**Rh19**	3,4,5-OMe-C_6H_2	51	>20:1	87:13

Triple effect of arene tuning

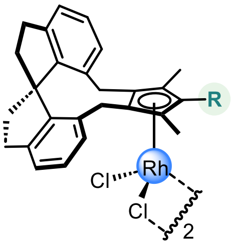

	Frontarm R	% yield	dr	er
**Rh24**	*i*Pr	61	>20:1	91:9
**Rh25**	Cy	**65**	**>20:1**	**92:8**
**Rh27**	Me	66	11:1	85:15
**Rh26**	*t*Bu	35	>20:1	70:30
**Rh28**	Ph	52	11:1	89:22
**Rh29**	3,5-*t*Bu-C_6H_3	60	12:1	88:12

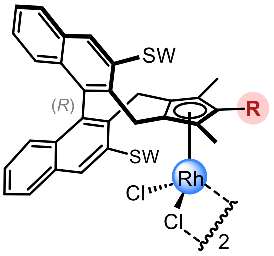

	SW	Frontarm R	% yield	dr	er
**Rh1a**	H	*i*Pr	40	14:1	34.5:65.5
**Rh4**	H	3,5-*t*Bu-C_6H_3	50	8:1	34:66
**Rh7**	H	2,6-OMe-C_6H_3	34	5:1	22:78
(S_a)-**Rh13**	Ph	*i*Pr	30	9:1	63:37

Scheme S31. Screening of Cp^{V}Rh(III) catalysts for the diastereo- and enantioselective (4 + 2) C-H annulation toward **12**.

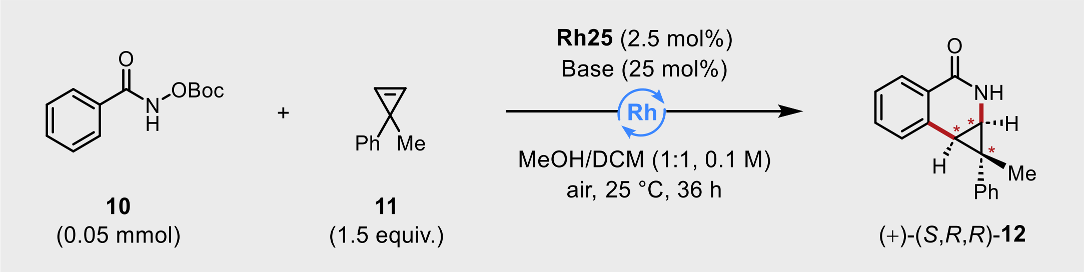

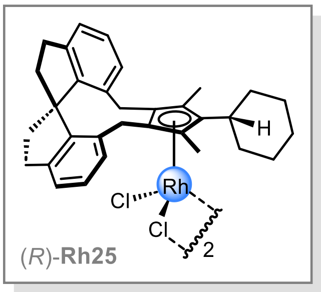

a Using (R)-Rh24

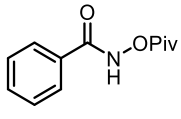

10'

Base	Other deviations	% yield	dr	er
CsOPiv	Ag_2CO_3 (5 mol%)[a]	29	>20:1	88:12
CsOPiv	$AgSbF_6$ (10 mol%)[a]	49	>20:1	87:13
CsOPiv	-	65	>20:1	92:8
CsOAc	-	66	>20:1	93.5:6.5
KOAc	-	**68**	**>20:1**	**93.5:6.5**
NaOAc	-	66	>20:1	93:7
LiOAc	-	67	>20:1	93:7
KOAc	MeOH	60	>20:1	94:6
KOAc	DCM	44	>20:1	90:10
KOAc	MeCN	39	>20:1	89:11
KOAc	Dioxane	23	>20:1	91:9
KOAc	10 °C	**68**	**>20:1**	**94.5:5.5**
KOAc	40 °C	62	17:1	93:7
KOAc	5 mol% **Rh25**	70	>20:1	93.5:6.5
KOAc	**10'** instead of **10**	79	17:1	86:14

Scheme S32. Finetuning the conditions of the diastereo- and enantioselective (4 + 2) C-H annulation toward **12**.

(1*S*,1a*R*,7b*R*)-1-Methyl-1-phenyl-1,1a,2,7b-tetrahydro-3*H*-cyclopropa[*c*]isoquinolin-3-one (**12**)

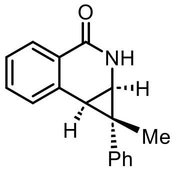

According to *General Procedure 10* (scale: 203 µmol of **10**), but with KOAc (25 mol%) at 10 °C instead of CsOPiv at 25 °C, and using (*R*)-**Rh25** as catalyst, annulation product (*S*,*R*,*R*)-**12** (31.5 mg, 126 µmol, 62% yield, 94.5:5.5 er) was obtained as a white solid with the characterization data matching those previously reported.[40,41] Purification was performed by Prep. TLC on silica (pentane/EtOAc = 1:1). A suitable crystal for X-ray analysis (**Figure S18**) was obtained by slow evaporation of a concentrated solution in hexane/isopropanol (9:1), and it confirmed the absolute configuration of (+)-(*S*,*R*,*R*)-**12**.

^{1}H NMR (400 MHz, $CDCl_3$) δ = 8.20 (dd, *J* = 7.8, 1.4 Hz, 1H), 7.54 – 7.49 (m, 1H), 7.45 – 7.32 (m, 6H), 7.29 – 7.24 (m, 1H), 6.21 (s, 1H), 3.47 (dd, *J* = 8.9, 3.4 Hz, 1H), 2.73 (d, *J* = 8.9 Hz, 1H), 1.14 (s, 3H) ppm; **R_f** (pentane/EtOAc, 1:1) = 0.36; $[\alpha]_D^{22}$ = +112.6 (*c* = 0.45, $CHCl_3$); **Chiral HPLC** (Chiralpak IA, 4.6 x 150 mm, 3 µm, Hexane/*i*PrOH 90:10, 1.0 mL/min, 35 °C, 254 nm) t_r (minor) = 6.17 min, t_r (major) = 7.34 min, 94.5:5.5 er; **XRD** (Cu*Kα*, R_1 = 3.24%) CCDC: 2530867.

Note: the characteristic ^{1}H NMR signal (400 MHz, $CDCl_3$) of the minor diastereomer, used to determine the dr of the crude reaction mixture, is located at δ = 3.36 (dd, *J* = 8.3, 3.2 Hz, 1H) ppm.

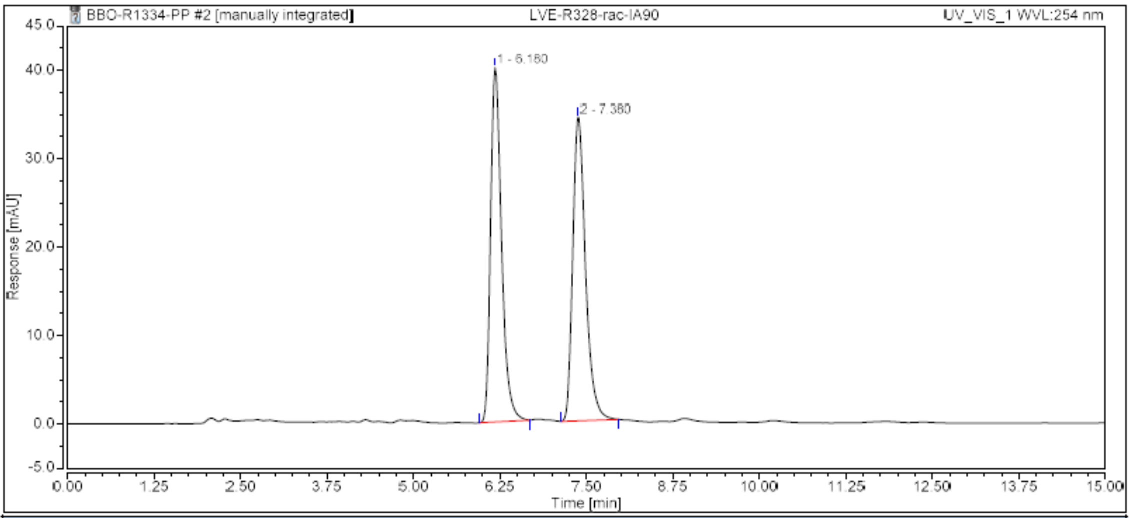

Integration Results

No.	Peak Name	Retention Time min	Area mAU*min	Height mAU	Relative Area %	Relative Height %	Amount n.a.
1		6.180	7.325	40.097	50.05	53.86	n.a.
2		7.380	7.311	34.354	49.95	46.14	n.a.

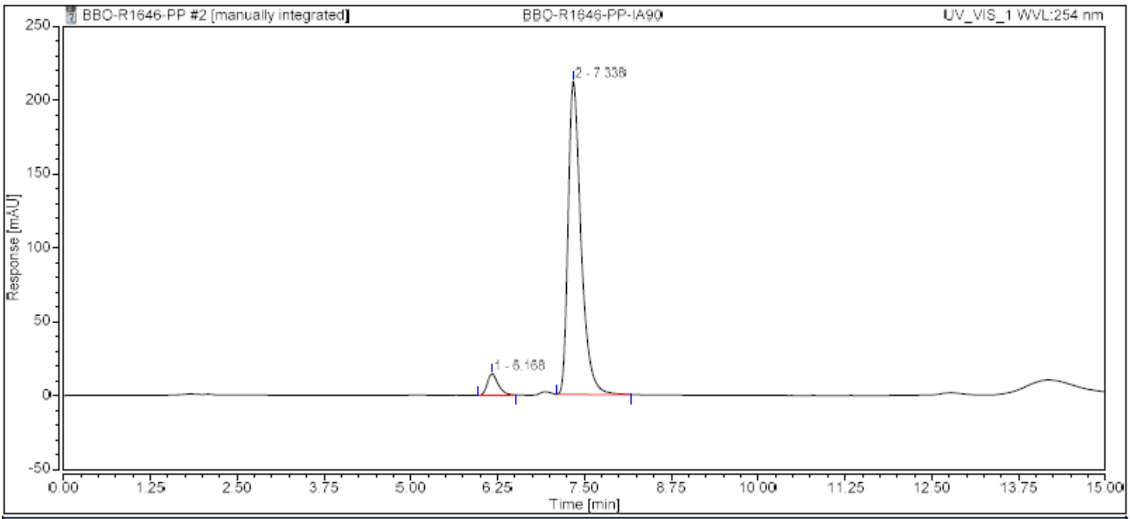

Integration Results

No.	Peak Name	Retention Time min	Area mAU*min	Height mAU	Relative Area %	Relative Height %	Amount n.a.
1		6.168	2.653	14.529	5.51	6.42	n.a.
2		7.338	45.508	211.939	94.49	93.58	n.a.

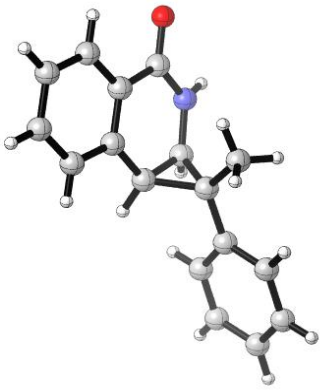

Figure S18. Solid-state X-ray structure of (+)-(*S*,*R*,*R*)-**12** (CCDC: 2530867) showing 50% probability thermal ellipsoids.

7. X-ray Crystallographic Data

Crystallographic data for the structures reported in this paper has been deposited at the Cambridge Crystallographic Data Center (CCDC) as Supplementary Publication No. 2202057 (**Co1a**), 2310524 (**Co6**), 2497532 (**Rh20b**), 2506334 (**Co12**), 2519618 (**3a**), and 2530867 (**12**). Copies of the data can be obtained free of charge on application to the CCDC via https://www.ccdc.cam.ac.uk/data_request/cif.

Cobalt(III) complex (R_a)-**Co1a**

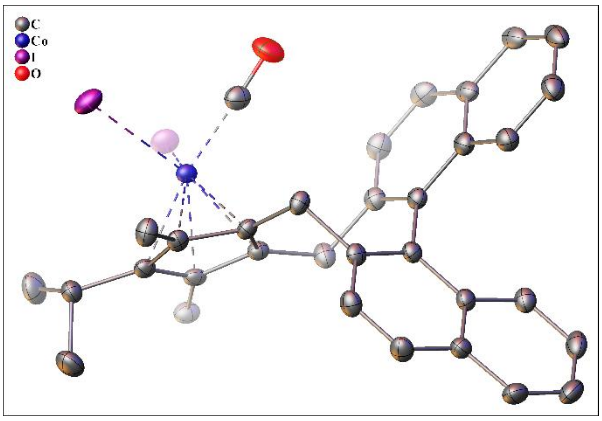

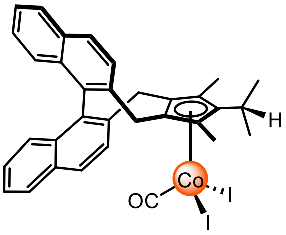

(R_a)-**Co1a**

Experimental. Single metallic dark brown plate-shaped crystals of (R_a)-**Co1a** were used as supplied. A suitable crystal with dimensions 0.12 × 0.09 × 0.03 mm^3 was selected and mounted on an XtaLAB Synergy R, DW system, HyPix-Arc 150 diffractometer. The crystal was kept at a steady T = 139.99(10) K during data collection. The structure was solved with the ShelXT 2018/2 solution program[42] using dual methods and by using Olex2 1.5 as the graphical interface.[43] The model was refined with ShelXL 2018/3 using full-matrix least-squares minimisation on F^2.[44]

Crystal Data. $C_{33}H_{29}CoI_2O$, M_r = 754.29, monoclinic, $P2_1$ (No. 4), a = 8.11637(6) Å, b = 19.71543(14) Å, c = 9.04355(7) Å, β = 92.0939(7)°, $\alpha = \gamma$ = 90°, V = 1446.163(18) Å^3, T = 139.99(10) K, Z = 2, Z' = 1, μ(Cu K$_\alpha$) = 21.587, 30246 reflections measured, 5911 unique (R_{int} = 0.0249) which were used in all calculations. The final wR_2 was 0.0407 (all data) and R_1 was 0.0166 (I≥2σ(I)).

**Compound**	(R_a)-**Co1a**
Formula	$C_{33}H_{29}CoI_2O$
$D_{calc.}$/ g cm^{-3}	1.732
μ/mm^{-1}	21.587
Formula Weight	754.29
Colour	metallic dark brown
Shape	plate-shaped
Size/mm^3	0.12×0.09×0.03
T/K	139.99(10)
Crystal System	monoclinic
Flack Parameter	-0.033(2)
Space Group	$P2_1$
a/Å	8.11637(6)
b/Å	19.71543(14)
c/Å	9.04355(7)
α/°	90
β/°	92.0939(7)
γ/°	90
V/Å^3	1446.163(18)
Z	2
Z'	1
Wavelength/Å	1.54184
Radiation type	Cu$K\alpha$
Θ_{min}/°	4.485
Θ_{max}/°	75.542
Measured Refl's.	30246
Indep't Refl's	5911
Refl's I≥2σ(I)	5831
R_{int}	0.0249
Parameters	338
Restraints	1
Largest Peak/e Å^{-3}	0.548
Deepest Hole/e Å^{-3}	-0.473
GooF	1.044
wR_2 (all data)	0.0407
wR_2	0.0406
R_1 (all data)	0.0169
R_1	0.0166
CCDC number	2202057

Cobalt(III) complex (R_a)-**Co6**

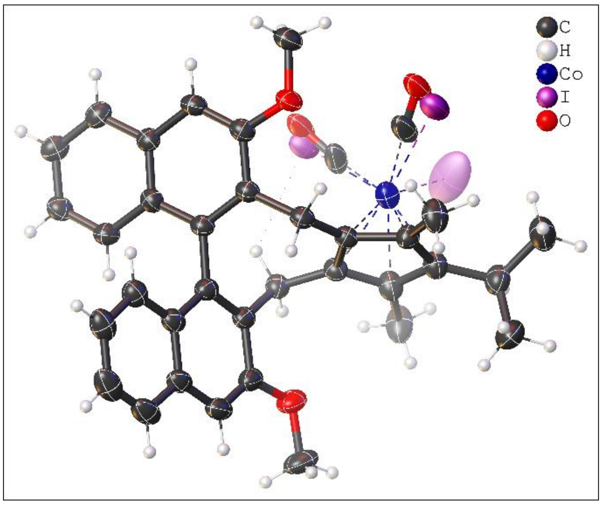

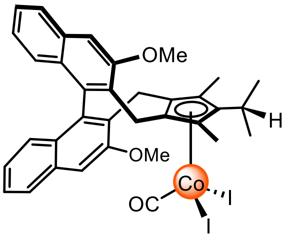

(R_a)-**Co6**

Experimental. Single metallic dark brown plate-shaped crystals of (R_a)-**Co6** were used as supplied. A suitable crystal with dimensions 0.15 × 0.08 × 0.02 mm³ was selected and mounted on an XtaLAB Synergy R, DW system, HyPix-Arc 150 diffractometer. The crystal was kept at a steady T = 139.99(10) K during data collection. The structure was solved with the ShelXT 2018/2 solution program[42] using dual methods and by using Olex2 1.5 as the graphical interface.[43] The model was refined with ShelXL 2019/3 using full-matrix least-squares minimisation on F^2.[44]

Crystal Data. $C_{35}H_{33}CoI_2O_3$, M_r = 814.34, orthorhombic, $P2_12_12_1$ (No. 19), a = 10.17308(13) Å, b = 15.51820(15) Å, c = 19.5117(3) Å, $\alpha = \beta = \gamma$ = 90°, V = 3080.27(6) Å³, T = 139.99(10) K, Z = 4, Z' = 1, μ(Cu K_α) = 20.372, 30842 reflections measured, 6236 unique (R_{int} = 0.0410) which were used in all calculations. The final wR_2 was 0.1206 (all data) and R_1 was 0.0484 (I≥2σ(I)).

**Compound**	(R_a)-**Co6**
Formula	$C_{35}H_{33}CoI_2O_3$
$D_{calc.}$/ g cm^{-3}	1.756
μ/mm^{-1}	20.372
Formula Weight	814.34
Colour	metallic dark brown
Shape	plate-shaped
Size/mm³	0.15×0.08×0.02
T/K	139.99(10)
Crystal System	orthorhombic
Flack Parameter	-0.020(4)
Space Group	$P2_12_12_1$
a/Å	10.17308(13)
b/Å	15.51820(15)
c/Å	19.5117(3)
α/°	90
β/°	90
γ/°	90
V/Å³	3080.27(6)
Z	4
Z'	1
Wavelength/Å	1.54184
Radiation type	Cu$K\alpha$
Θ_{min}/°	3.639
Θ_{max}/°	75.644
Measured Refl's.	30842
Indep't Refl's	6236
Refl's I≥2σ(I)	5811
R_{int}	0.0410
Parameters	398
Restraints	16
Largest Peak/e Å^{-3}	1.051
Deepest Hole/e Å^{-3}	-1.168
GooF	1.027
wR_2 (all data)	0.1206
wR_2	0.1187
R_1 (all data)	0.0517
R_1	0.0484
CCDC number	2310524

Rhodium(III) complex (*R*)-**Rh20b**

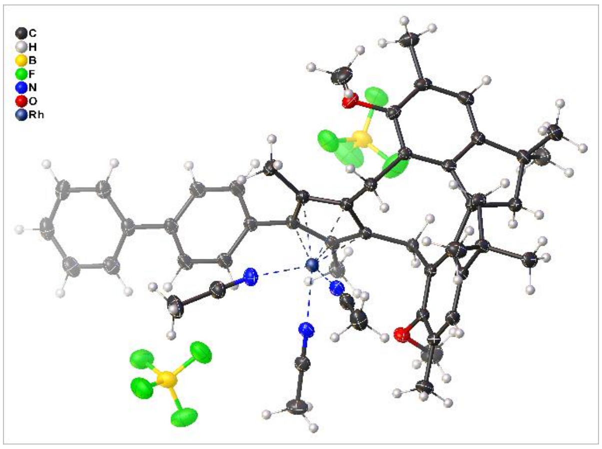

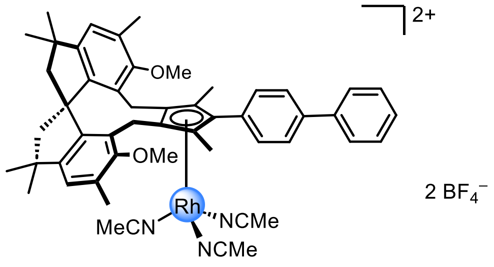

(*R*)-**Rh20b**

Experimental. Single clear intense yellow prism-shaped crystals of (*R*)-**Rh20b** were used as supplied. A suitable crystal with dimensions 0.13 × 0.05 × 0.03 mm was selected and mounted on a XtaLAB Synergy R, DW system, HyPix-Arc 150 diffractometer. The crystal was kept at a steady *T* = 140.00(10) K during data collection. The structure was solved with the ShelXT 2018/2 solution program[42] using dual methods and by using Olex2 1.5 as the graphical interface.[43] The model was refined with ShelXL 2019/3 using full-matrix least-squares minimisation on F^2.[44]

Crystal Data. $C_{52}H_{58}B_2F_8N_3O_2Rh$, M_r = 1033.54, hexagonal, $P6_5$ (No. 170), a = 11.74712(9) Å, b = 11.74712(9) Å, c = 60.7047(6) Å, α = 90°, β = 90°, γ = 120°, V = 7254.63(13) Å^3, T = 140.00(10) K, Z = 6, Z' = 1, μ(Cu K$_\alpha$) = 3.491, 85417 reflections measured, 9740 unique (R_{int} = 0.0290) which were used in all calculations. The final wR_2 was 0.0659 (all data) and R_1 was 0.0257 (I≥2σ(I)).

**Compound**	(*R*)-**Rh20b**
Formula	$C_{52}H_{58}B_2F_8N_3O_2Rh$
$D_{calc.}$/ g cm^{-3}	1.419
μ/mm^{-1}	3.491
Formula Weight	1033.54
Colour	clear intense yellow
Shape	prism-shaped
Size/mm	0.13×0.05×0.03
T/K	140.00(10)
Crystal System	hexagonal
Flack Parameter	-0.012(3)
Hooft Parameter	-0.0100(11)
Space Group	$P6_5$
a/Å	11.74712(9)
b/Å	11.74712(9)
c/Å	60.7047(6)
α/°	90
β/°	90
γ/°	120
V/Å^3	7254.63(13)
Z	6
Z'	1
Wavelength/Å	1.54184
Radiation type	Cu K$_\alpha$
Θ_{min}/°	4.346
Θ_{max}/°	74.737
Index range h	-9 ≤ h ≤ 14
Index range k	-14 ≤ k ≤ 13
Index range l	-75 ≤ l ≤ 74
Measured Refl's.	85417
Indep't Refl's	9740
Refl's I≥2σ(I)	9441
R_{int}	0.0290
Parameters	626
Restraints	1
Largest Peak/eÅ^3	0.941
Deepest Hole/eÅ^3	-0.367
GooF	1.041
R_1 (I≥2σ(I) / all)	0.0257 / 0.0270
wR_2 (I≥2σ(I) / all)	0.0654 / 0.0659
CCDC number	2497532

Cobalt(III) complex (*R*)-**Co12**

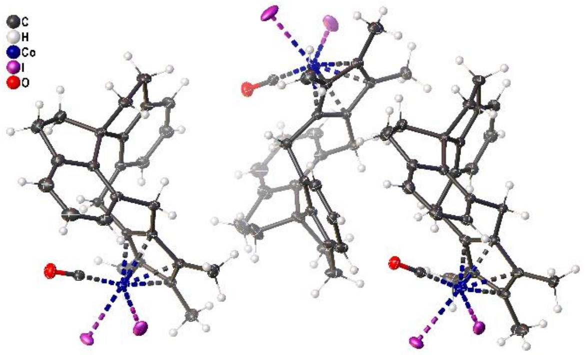

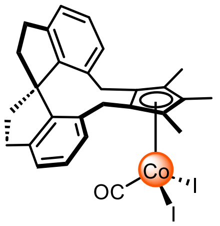

(*R*)-**Co12**

Experimental. Single metallic dark brown irregular-shaped crystals of (*R*)-**Co12** were used as supplied. A suitable crystal with dimensions 0.17 × 0.12 × 0.09 mm was selected and mounted on a XtaLAB Synergy R, DW system, HyPix-Arc 150 diffractometer. The crystal was kept at a steady T = 140.00(10) K during data collection. The structure was solved with the ShelXT 2018/2 solution program[42] using dual methods and by using Olex2 1.5 as the graphical interface.[43] The model was refined with ShelXL 2019/3 using full-matrix least-squares minimisation on $|F|^2$.[44]

Crystal Data. $C_{28}H_{27}CoI_2O$, M_r = 692.22, orthorhombic, $P2_12_12_1$ (No. 19), a = 12.40312(9) Å, b = 17.56709(13) Å, c = 34.5212(3) Å, $\alpha = \beta = \gamma$ = 90°, V = 7521.72(10) Å^3, T = 140.00(10) K, Z = 12, Z' = 3, μ(Mo K$_\alpha$) = 3.165, 279103 reflections measured, 36472 unique (R_{int} = 0.0284) which were used in all calculations. The final wR_2 was 0.0514 (all data) and R_1 was 0.0257 (I≥2σ(I)).

**Compound**	(*R*)-**Co12**
Formula	$C_{28}H_{27}CoI_2O$
$D_{calc.}$/ g cm^{-3}	1.834
μ/mm^{-1}	3.165
Formula Weight	692.22
Colour	metallic dark brown
Shape	irregular-shaped
Size/mm	0.17×0.12×0.09
T/K	140.00(10)
Crystal System	orthorhombic
Flack Parameter	-0.020(3)
Hooft Parameter	-0.020(2)
Space Group	$P2_12_12_1$
a/Å	12.40312(9)
b/Å	17.56709(13)
c/Å	34.5212(3)
α/°	90
β/°	90
γ/°	90
V/Å^3	7521.72(10)
Z	12
Z'	3
Wavelength/Å	0.71073
Radiation type	Mo K$_\alpha$
Θ_{min}/°	1.654
Θ_{max}/°	36.317
Index range h	-19 ≤ h ≤ 20
Index range k	-29 ≤ k ≤ 29
Index range l	-57 ≤ l ≤ 57
Measured Refl's.	279103
Indep't Refl's	36472
Refl's I≥2σ(I)	33457
R_{int}	0.0284
Parameters	874
Restraints	0
Largest Peak/eÅ^3	2.348
Deepest Hole/eÅ^3	-1.833
GooF	1.048
R_1 (I≥2σ(I) / all)	0.0257 / 0.0304
wR_2 (I≥2σ(I) / all)	0.0502 / 0.0514
CCDC number	2506334

Allylic amine (*S*)-**3a**

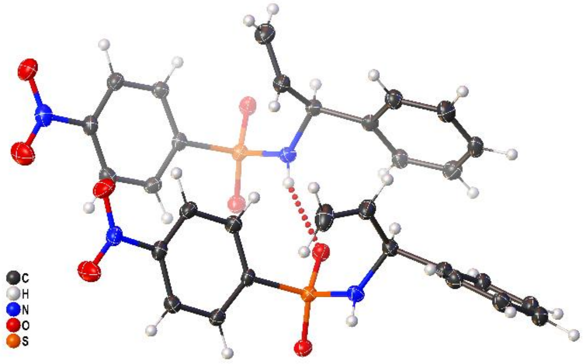

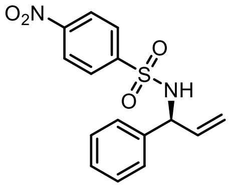

(*S*)-**3a**

Experimental. Single colourless prism-shaped crystals of (*S*)-**3a** were used as supplied. A suitable crystal with dimensions 0.13 × 0.11 × 0.05 mm was selected and mounted on a XtaLAB Synergy R, DW system, HyPix-Arc 150 diffractometer. The crystal was kept at a steady T = 100.00(10) K during data collection. The structure was solved with the ShelXT 2018/2 solution program[42] using dual methods and by using Olex2 1.5 as the graphical interface.[43] The model was refined with ShelXL 2019/3 using full matrix least squares minimisation on $|F|^2$.[44]

Crystal Data. $C_{15}H_{14}N_2O_4S$, M_r = 318.34, triclinic, $P1$ (No. 1), a = 6.71532(16) Å, b = 10.0429(2) Å, c = 12.1212(3) Å, α = 111.315(2)°, β = 96.927(2)°, γ = 102.914(2)°, V = 723.72(3) Å^3, T = 100.00(10) K, Z = 2, Z' = 2, μ(Cu K$_\alpha$) = 2.180, 19761 reflections measured, 5328 unique (R_{int} = 0.0319) which were used in all calculations. The final wR_2 was 0.0771 (all data) and R_1 was 0.0314 (I≥2σ(I)).

**Compound**	(*S*)-**3a**
Formula	$C_{15}H_{14}N_2O_4S$
$D_{calc.}$/ g cm^{-3}	1.461
μ/mm^{-1}	2.180
Formula Weight	318.34
Colour	colourless
Shape	prism-shaped
Size/mm	0.13×0.11×0.05
T/K	100.00(10)
Crystal System	triclinic
Flack Parameter	-0.023(8)
Hooft Parameter	-0.023(6)
Space Group	$P1$
a/Å	6.71532(16)
b/Å	10.0429(2)
c/Å	12.1212(3)
α/°	111.315(2)
β/°	96.927(2)
γ/°	102.914(2)
V/Å^3	723.72(3)
Z	2
Z'	2
Wavelength/Å	1.54184
Radiation type	Cu K$_\alpha$
Θ_{min}/°	4.015
Θ_{max}/°	75.820
Index range h	-8 ≤ h ≤ 7
Index range k	-12 ≤ k ≤ 12
Index range l	-15 ≤ l ≤ 14
Measured Refl's.	19761
Indep't Refl's	5328
Refl's I≥2σ(I)	5052
R_{int}	0.0319
Parameters	510
Restraints	3
Largest Peak/eÅ^3	0.203
Deepest Hole/eÅ^3	-0.200
GooF	1.067
R_1 (I≥2σ(I) / all)	0.0314 / 0.0337
wR_2 (I≥2σ(I) / all)	0.0759 / 0.0771
CCDC number	2519618

Cyclopropa[*c*]dihydroisoquinolone (*S*,*R*,*R*)-**12**

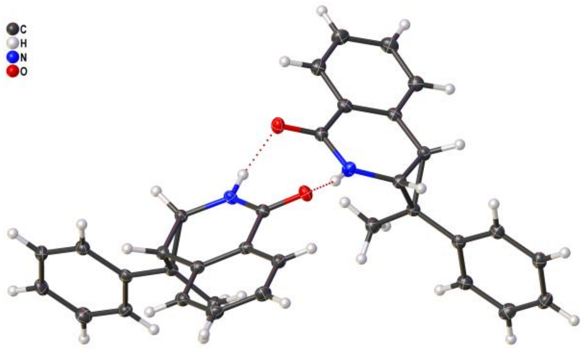

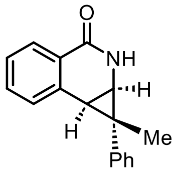

(*S*,*R*,*R*)-**12**

Experimental. Single colourless prism-shaped crystals of (*S*,*R*,*R*)-**12** were used as supplied. A suitable crystal with dimensions 0.38 × 0.05 × 0.05 mm was selected and mounted on a XtaLAB Synergy R, DW system, HyPix-Arc 150 diffractometer. The crystal was kept at a steady *T* = 100.00(10) K during data collection. The structure was solved with the ShelXT 2018/2 solution program[42] using dual methods and by using Olex2 1.5 as the graphical interface.[43] The model was refined with ShelXL 2019/3 using full matrix least squares minimisation on $|F|^2$.[44]

Crystal Data. $C_{17}H_{15}NO$, M_r = 249.30, orthorhombic, $P2_12_12_1$ (No. 19), a = 8.66451(15) Å, b = 13.4947(3) Å, c = 21.7234(5) Å, $\alpha = \beta = \gamma$ = 90°, *V* = 2540.00(9) Å^3, *T* = 100.00(10) K, *Z* = 8, *Z'* = 2, μ(Cu K$_\alpha$) = 0.635, 32259 reflections measured, 5203 unique (R_{int} = 0.0449) which were used in all calculations. The final wR_2 was 0.0779 (all data) and R_1 was 0.0324 (I≥2σ(I)).

**Compound**	(*S*,*R*,*R*)-**12**
Formula	$C_{17}H_{15}NO$
$D_{calc.}$/ g cm^{-3}	1.304
μ/mm^{-1}	0.635
Formula Weight	249.30
Colour	colourless
Shape	prism-shaped
Size/mm	0.38×0.05×0.05
T/K	100.00(10)
Crystal System	orthorhombic
Flack Parameter	0.2(2)
Hooft Parameter	-0.04(9)
Space Group	$P2_12_12_1$
a/Å	8.66451(15)
b/Å	13.4947(3)
c/Å	21.7234(5)
α/°	90
β/°	90
γ/°	90
V/Å^3	2540.00(9)
Z	8
Z'	2
Wavelength/Å	1.54184
Radiation type	CuK_α
Θ_{min}/°	3.856
Θ_{max}/°	75.817
Index range h	-8 ≤ h ≤ 10
Index range k	-16 ≤ k ≤ 16
Index range l	-26 ≤ l ≤ 27
Measured Refl's.	32259
Indep't Refl's	5203
Refl's I≥2σ(I)	4862
R_{int}	0.0449
Parameters	355
Restraints	0
Largest Peak/eÅ^3	0.199
Deepest Hole/eÅ^3	-0.150
GooF	1.033
R_1 (I≥2σ(I) / all)	0.0324 / 0.0361
wR_2 (I≥2σ(I) / all)	0.0767 / 0.0779
CCDC number	2530867

8. Topographical Steric Maps

Topographical steric maps for the solid-state X-ray structures of chiral metal complexes reported in this work (i.e. (R_a)-**Co1a**, (R_a)-**Co6**, (R)-**Rh20b**, and (R)-**Co12**) are provided below and were generated using the SambVca 2.1 tool (with 3.5 or 10 Å sphere radius, 0.1 Å mesh spacing, bondi radii scaled by 1.17, removal of non-Cp ligands, and inclusion of H atoms).[45] The web application can be used free of charge via https://www.aocdweb.com/OMtools/sambvca2.1/.

Cobalt(III) complex (R_a)-**Co1a**

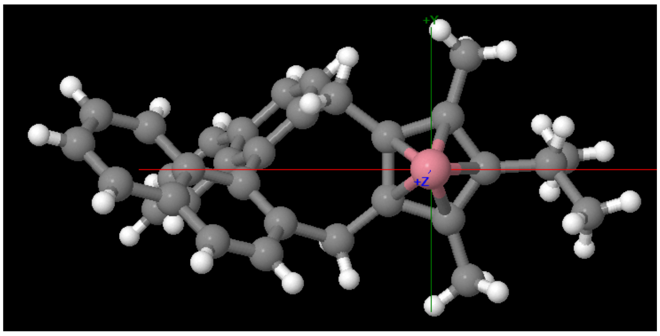

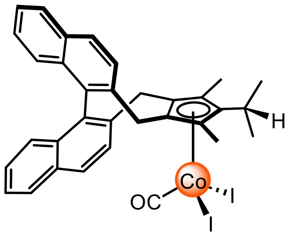

(R_a)-**Co1a**

%V Free	%V Buried	% V Tot/V Ex
47.0	53.0	99.9

Quadrant	V f	V b	V t	%V f	%V b
SW	19.0	25.8	44.9	42.4	57.6
NW	22.0	22.8	44.9	49.1	50.9
NE	22.5	22.4	44.9	50.2	49.8
SE	20.8	24.1	44.9	46.3	53.7

Steric Map (3.5 Å sphere radius)

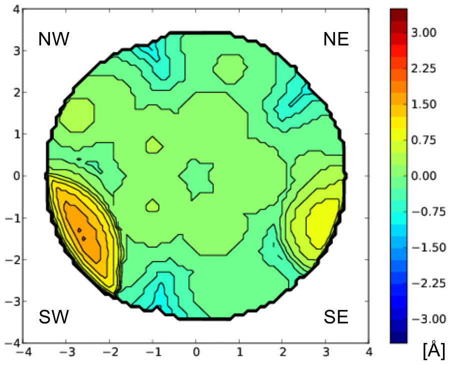

Steric Map (10 Å sphere radius)

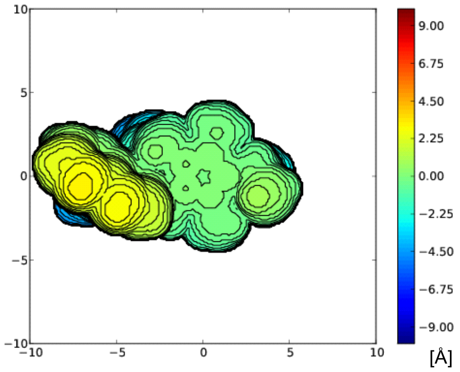

Cobalt(III) complex (R_a)-**Co6**

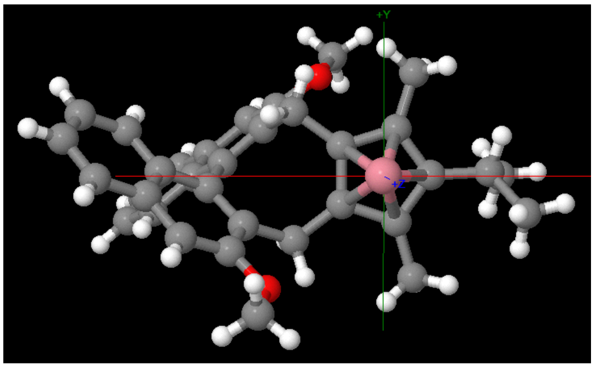

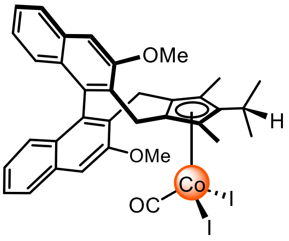

(R_a)-**Co6**

%V Free	%V Buried	% V Tot/V Ex
45.9	**54.1**	99.9

Quadrant	V f	V b	V t	%V f	%V b
SW	16.8	28.1	44.9	**37.4**	62.6
NW	23.1	21.8	44.9	**51.5**	48.5
NE	22.4	22.5	44.9	**49.9**	50.1
SE	20.2	24.7	44.9	**44.9**	55.1

Steric Map (3.5 Å sphere radius)

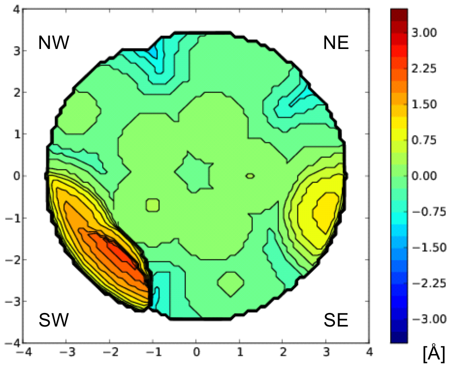

Steric Map (10 Å sphere radius)

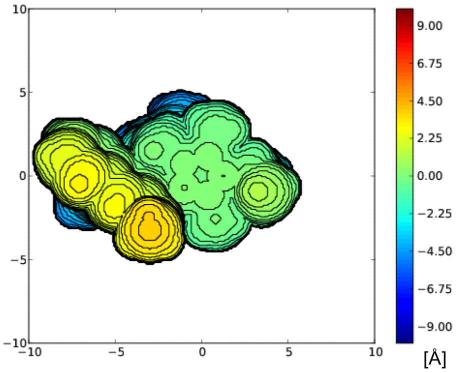

Rhodium(III) complex (*R*)-**Rh20b**

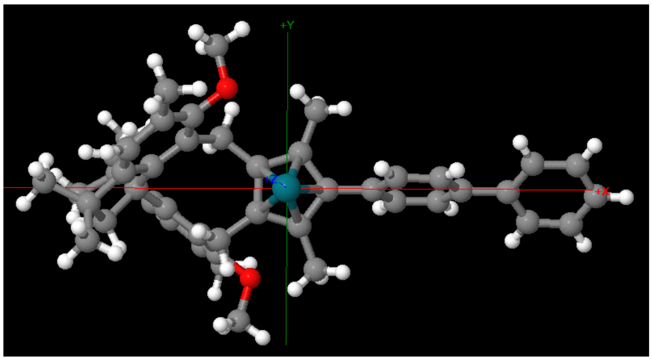

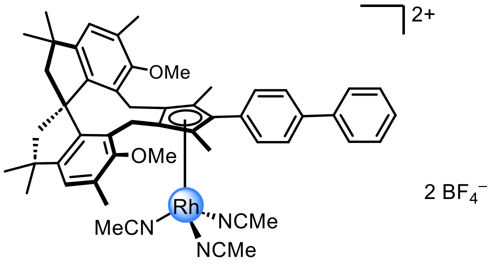

(*R*)-**Rh20b**

%V Free	%V Buried	% V Tot/V Ex
43.0	57.0	99.9

Quadrant	V f	V b	V t	%V f	%V b
SW	22.8	22.0	44.9	50.9	49.1
NW	13.4	31.4	44.9	29.9	70.1
NE	19.6	25.3	44.9	43.7	56.3
SE	21.3	23.6	44.9	47.5	52.5

Steric Map (3.5 Å sphere radius)

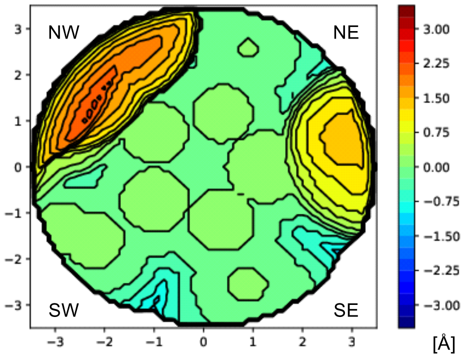

Steric Map (10 Å sphere radius)

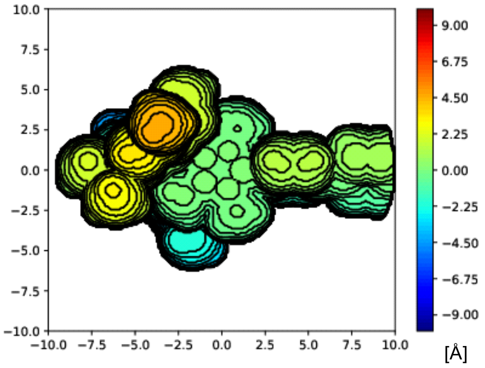

Cobalt(III) complex (*R*)-**Co12**

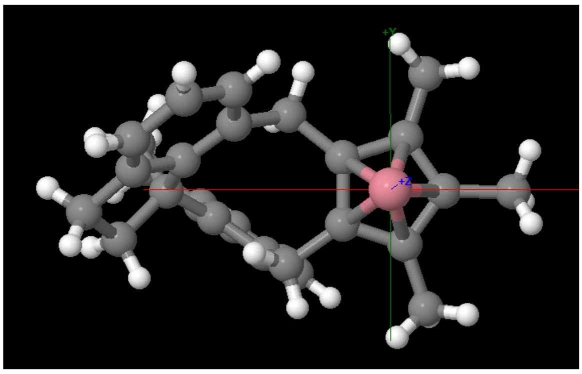

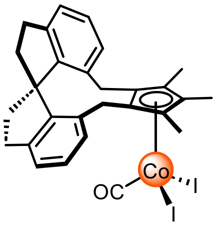

(*R*)-**Co12**

%V Free	%V Buried	% V Tot/V Ex
47.3	52.7	99.9

Quadrant	V f	V b	V t	%V f	%V b
SW	22.5	22.3	44.9	50.3	49.7
NW	17.2	27.7	44.9	38.3	61.7
NE	22.9	22.0	44.9	51.1	48.9
SE	22.2	22.7	44.9	49.5	50.5

Steric Map (3.5 Å sphere radius)

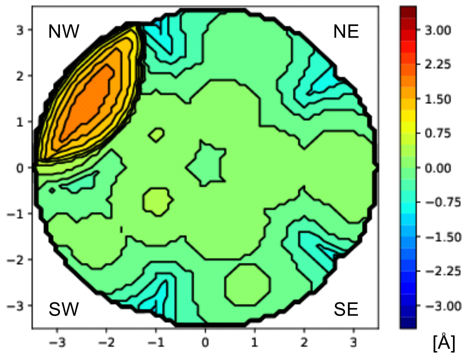

Steric Map (10 Å sphere radius)

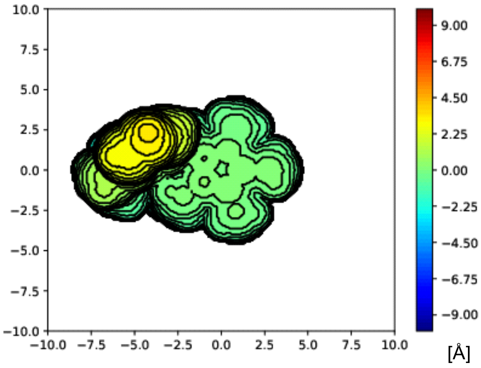

9. References

[1] B. Van Den Bossche, N. Cramer, *J. Am. Chem. Soc.* **2026**, *148*, 3775–3787.

[2] T. Ooi, M. Kameda, K. Maruoka, *J. Am. Chem. Soc.* **2003**, *125*, 5139–5151.

[3] M. Ikunaka, K. Maruoka, Y. Okuda, T. Ooi, *Org. Process Res. Dev.* **2003**, *7*, 644–648.

[4] B. Ye, N. Cramer, *J. Am. Chem. Soc.* **2013**, *135*, 636–639.

[5] S. Reddy Chidipudi, D. J. Burns, I. Khan, H. W. Lam, *Angew. Chem. Int. Ed.* **2015**, *54*, 13975–13979.

[6] M. Berliner, K. Belecki, *Org. Synth.* **2007**, *84*, 102.

[7] T. R. Wu, L. Shen, J. M. Chong, *Org. Lett.* **2004**, *6*, 2701–2704.

[8] M. Dieckmann, Y.-S. Jang, N. Cramer, *Angew. Chem. Int. Ed.* **2015**, *54*, 12149–12152.

[9] C. Duchemin, G. Smits, N. Cramer, *Organometallics* **2019**, *38*, 3939–3947.

[10] T. Ooi, Y. Uematsu, K. Maruoka, *J. Org. Chem.* **2003**, *68*, 4576–4578.

[11] D. Kossler, N. Cramer, *Chem. Sci.* **2017**, *8*, 1862–1866.

[12] H. Konishi, F. Hoshino, K. Manabe, *Chem. Pharm. Bull.* **2016**, *64*, 1438–1441.

[13] H. Egami, K. Sato, J. Asada, Y. Kawato, Y. Hamashima, *Tetrahedron* **2015**, *71*, 6384–6388.

[14] W. Guo, X. Pang, J. Jiang, J. Wang, *Org. Lett.* **2023**, *25*, 3823–3828.

[15] H. Gu, Z. Han, H. Xie, X. Lin, *Org. Lett.* **2018**, *20*, 6544–6549.

[16] H. Yang, R. Zhang, S. Z. Zhang, Q. Gu, S.-L. You, *ACS Catal.* **2023**, *13*, 8838–8844.

[17] Q. Zhou, R. Pan, H. Shan, X. Lin, *Synthesis* **2019**, *51*, 557–563.

[18] L. Wang, J. Zhong, X. Lin, *Angew. Chem. Int. Ed.* **2019**, *58*, 15824–15828.

[19] J.-H. Xie, L.-X. Wang, Y. Fu, S.-F. Zhu, B.-M. Fan, H.-F. Duan, Q.-L. Zhou, *J. Am. Chem. Soc.* **2003**, *125*, 4404–4405.

[20] S.-F. Zhu, Y. Yang, L.-X. Wang, B. Liu, Q.-L. Zhou, *Org. Lett.* **2005**, *7*, 2333–2335.

[21] L. Huang, Y. Kitakawa, K. Yamada, F. Kamiyama, M. Kojima, T. Yoshino, S. Matsunaga, *Angew. Chem. Int. Ed.* **2023**, *62*, e202305480.

[22] Y. Sun, N. Cramer, *Chem. Sci.* **2018**, *9*, 2981–2985.

[23] W.-J. Cui, Z.-J. Wu, Q. Gu, S.-L. You, *J. Am. Chem. Soc.* **2020**, *142*, 7379–7385.

[24] P. Gajewski, M. Renom-Carrasco, S. V. Facchini, L. Pignataro, L. Lefort, J. G. de Vries, R. Ferraccioli, U. Piarulli, C. Gennari, *Eur. J. Org. Chem.* **2015**, *2015*, 5526–5536.

[25] G. Smits, B. Audic, M. D. Wodrich, C. Corminboeuf, N. Cramer, *Chem. Sci.* **2017**, *8*, 7174–7179.

[26] K. Ozols, Y.-S. Jang, N. Cramer, *J. Am. Chem. Soc.* **2019**, *141*, 5675–5680.

[27] Y. Sun, N. Cramer, *Angew. Chem. Int. Ed.* **2018**, *57*, 15539–15543.

[28] M. Brauns, N. Cramer, *Angew. Chem. Int. Ed.* **2019**, *58*, 8902–8906.

[29] P. Sihag, M. Jeganmohan, *Chem. Commun.* **2021**, *57*, 6428–6431.

[30] Y.-Y. Liu, S. Cao, M.-P. Luo, S.-G. Wang, *CCS Chem.* **2025**, DOI: 10.31635/ccschem.025.202506179.

[31] A. G. Campaña, B. Bazdi, N. Fuentes, R. Robles, J. M. Cuerva, J. E. Oltra, S. Porcel, A. M. Echavarren, *Angew. Chem. Int. Ed.* **2008**, *47*, 7515–7519.

[32] A. Seoane, N. Casanova, N. Quiñones, J. L. Mascareñas, M. Gulías, *J. Am. Chem. Soc.* **2014**, *136*, 7607–7610.

[33] S. Zhang, W. Zhang, H. Yang, Q. Gu, S.-L. You, *Chin. J. Org. Chem.* **2023**, *43*, 2926-2933.

[34] T. Nishimura, A. Noishiki, G. Chit Tsui, T. Hayashi, *J. Am. Chem. Soc.* **2012**, *134*, 5056–5059.

[35] Y. Kato, L. Lin, M. Kojima, T. Yoshino, S. Matsunaga, *ACS Catal.* **2021**, *11*, 4271–4277.

[36] M. V. Pham, N. Cramer, *Chem. - Eur. J.* **2016**, *22*, 2270–2273.

[37] H. Liu, J. Li, M. Xiong, J. Jiang, J. Wang, *J. Org. Chem.* **2016**, *81*, 6093–6099.

[38] B. Ye, N. Cramer, *Science* **2012**, *338*, 504–506.

[39] N. Guimond, S. I. Gorelsky, K. Fagnou, *J. Am. Chem. Soc.* **2011**, *133*, 6449–6457.

[40] L. K. Verdhi, M. D. Wodrich, N. Cramer, *J. Am. Chem. Soc.* **2025**, *147*, 15041–15049.

[41] S. Shaaban, H. Li, C. Merten, A. P. Antonchick, H. Waldmann, *Synthesis* **2021**, *53*, 2192–2200.

[42] G. M. Sheldrick, *Acta Crystallogr. Sect. A Found. Adv.* **2015**, *71*, 3–8.

[43] O. V. Dolomanov, L. J. Bourhis, R. J. Gildea, J. A. K. Howard, H. Puschmann, *J. Appl. Crystallogr.* **2009**, *42*, 339–341.

[44] G. M. Sheldrick, *Acta Crystallogr. Sect. C Struct. Chem.* **2015**, *71*, 3–8.

[45] L. Falivene, Z. Cao, A. Petta, L. Serra, A. Poater, R. Oliva, V. Scarano, L. Cavallo, *Nat. Chem.* **2019**, *11*, 872–879.

10. NMR spectra

Copies of the ^{1}H NMR, $^{13}C\{^{1}H\}$ NMR, $^{19}F\{^{1}H\}$ NMR, and $^{31}P\{^{1}H\}$ NMR spectra of new unpublished compounds (or compounds of which the published NMR data was incomplete) are provided with indication of the nucleus' resonance frequency and the employed deuterated solvent. All spectra were recorded at 298 K, unless otherwise indicated.

Copies of the HSQC spectra of **Co14**, **SI41**, and **Rh31** are provided as well.

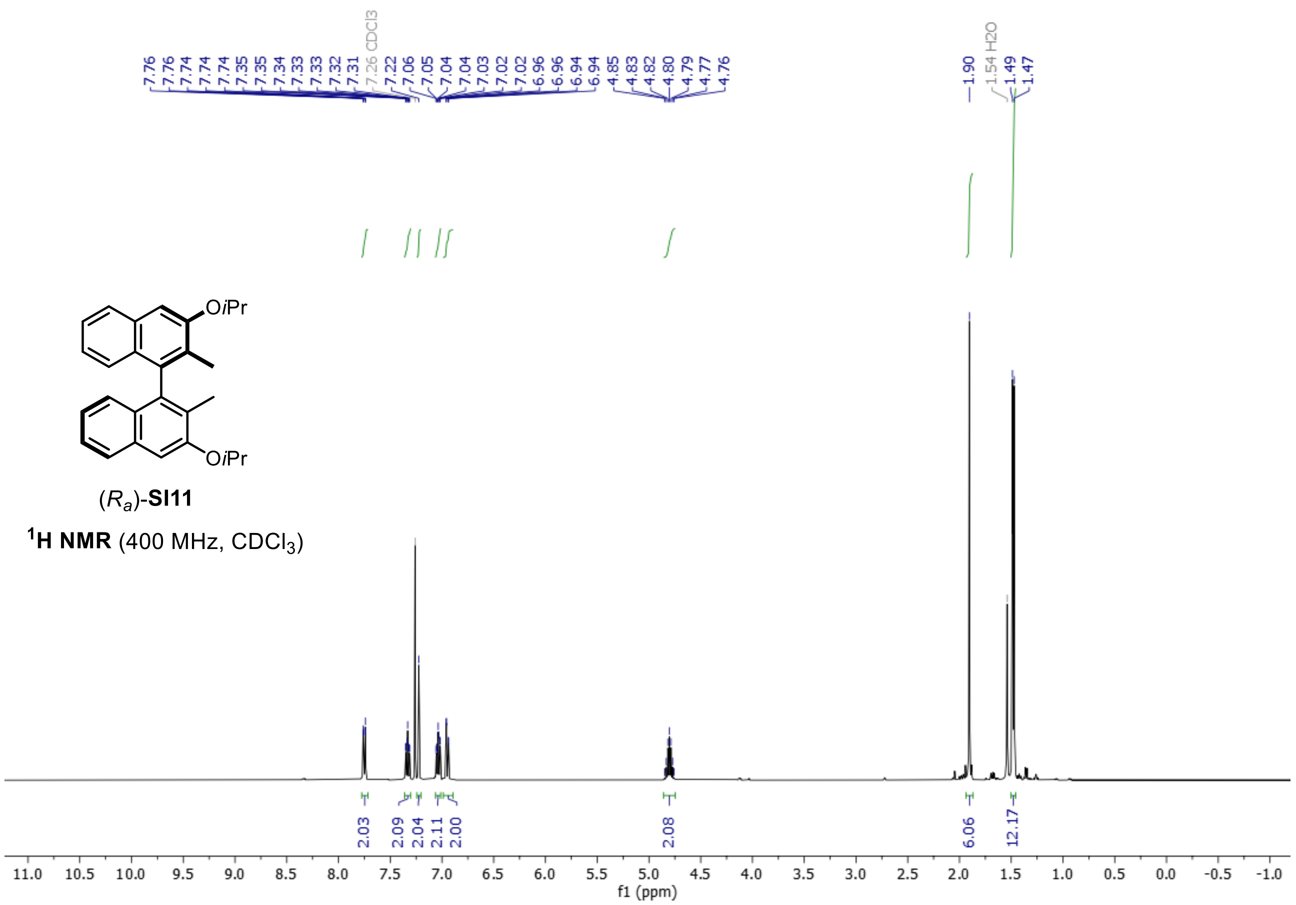
7.76 7.76 7.74 7.74 7.74 7.35 7.35 7.34 7.33 7.33 7.32 7.31 7.26 CDCl3 7.22 7.06 7.05 7.04 7.04 7.03 7.02 7.02 6.96 6.96 6.94 6.94 4.85 4.83 4.82 4.80 4.79 4.77 4.76
1.90 1.54 H2O 1.49 1.47
OiPr
OiPr
(Ra)-SI11
1H NMR (400 MHz, CDCl3)
2.03 2.09 2.04 2.11 2.00 2.08 6.06 12.17
f1 (ppm)

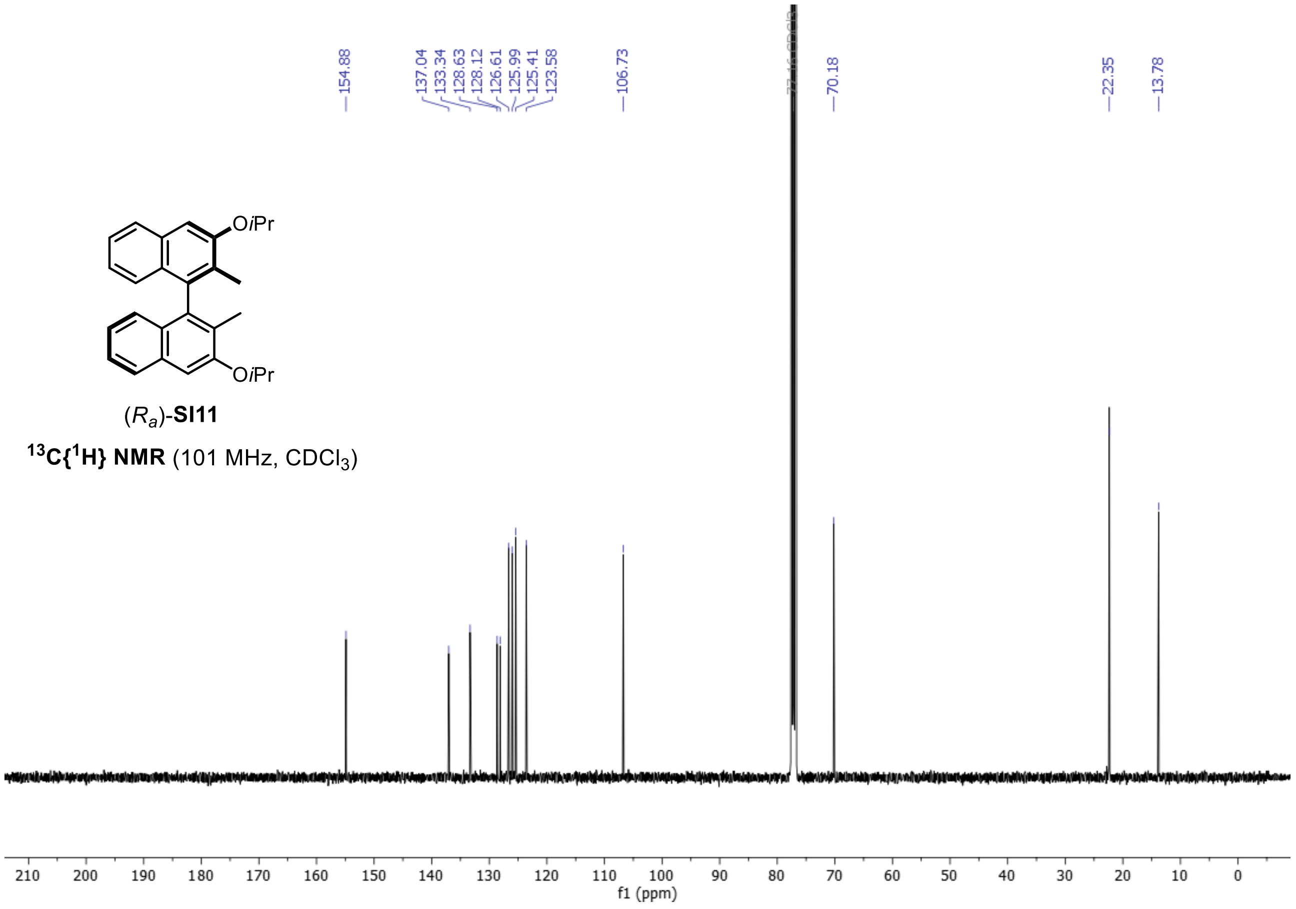
154.88
137.04 133.34 128.63 128.12 126.61 125.99 125.41 123.58
106.73
70.18
22.35
13.78
OiPr
OiPr
(Ra)-SI11
13C{1H} NMR (101 MHz, CDCl3)
f1 (ppm)

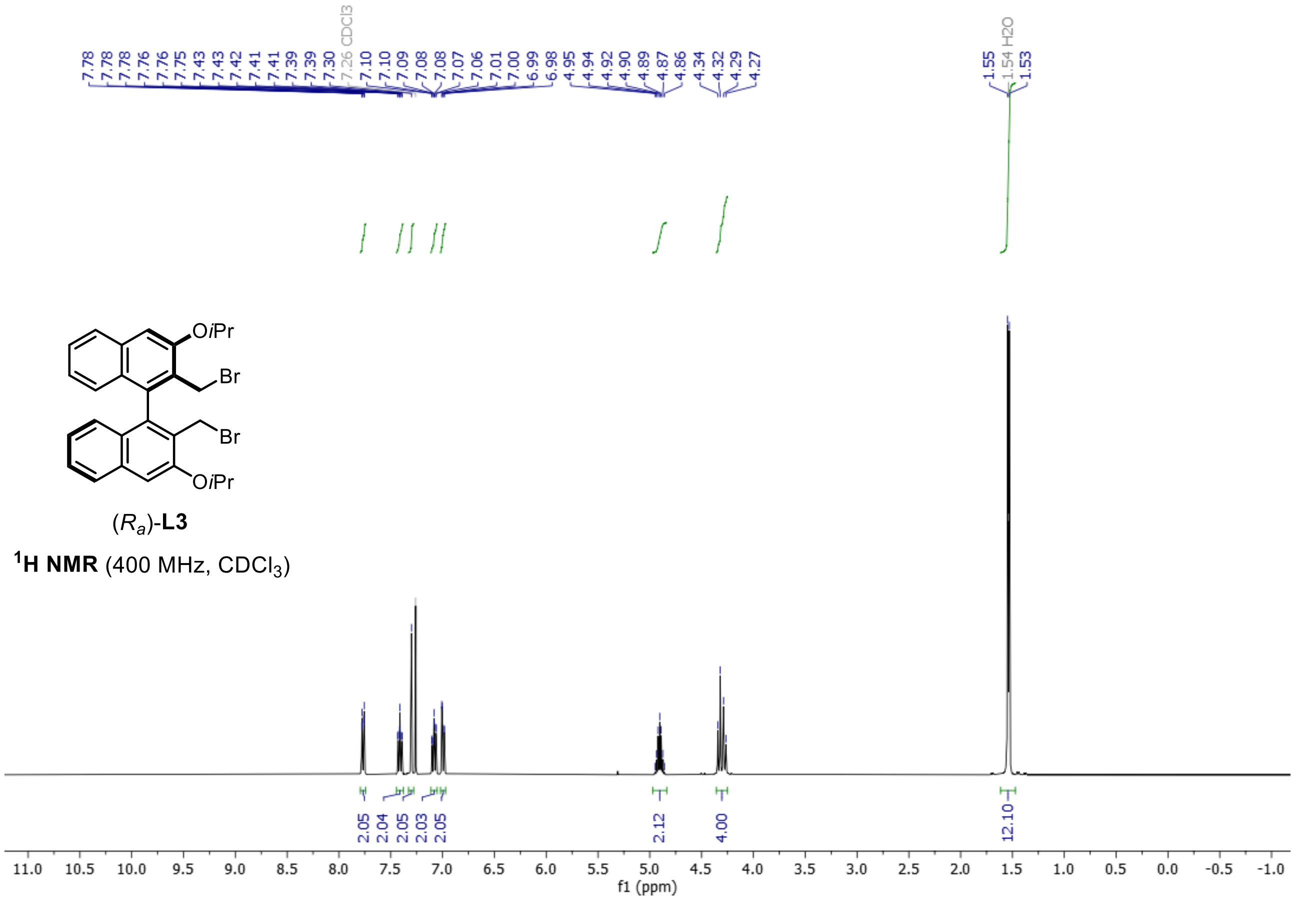

(R_a)-**L3**

^{1}H NMR (400 MHz, $CDCl_3$)

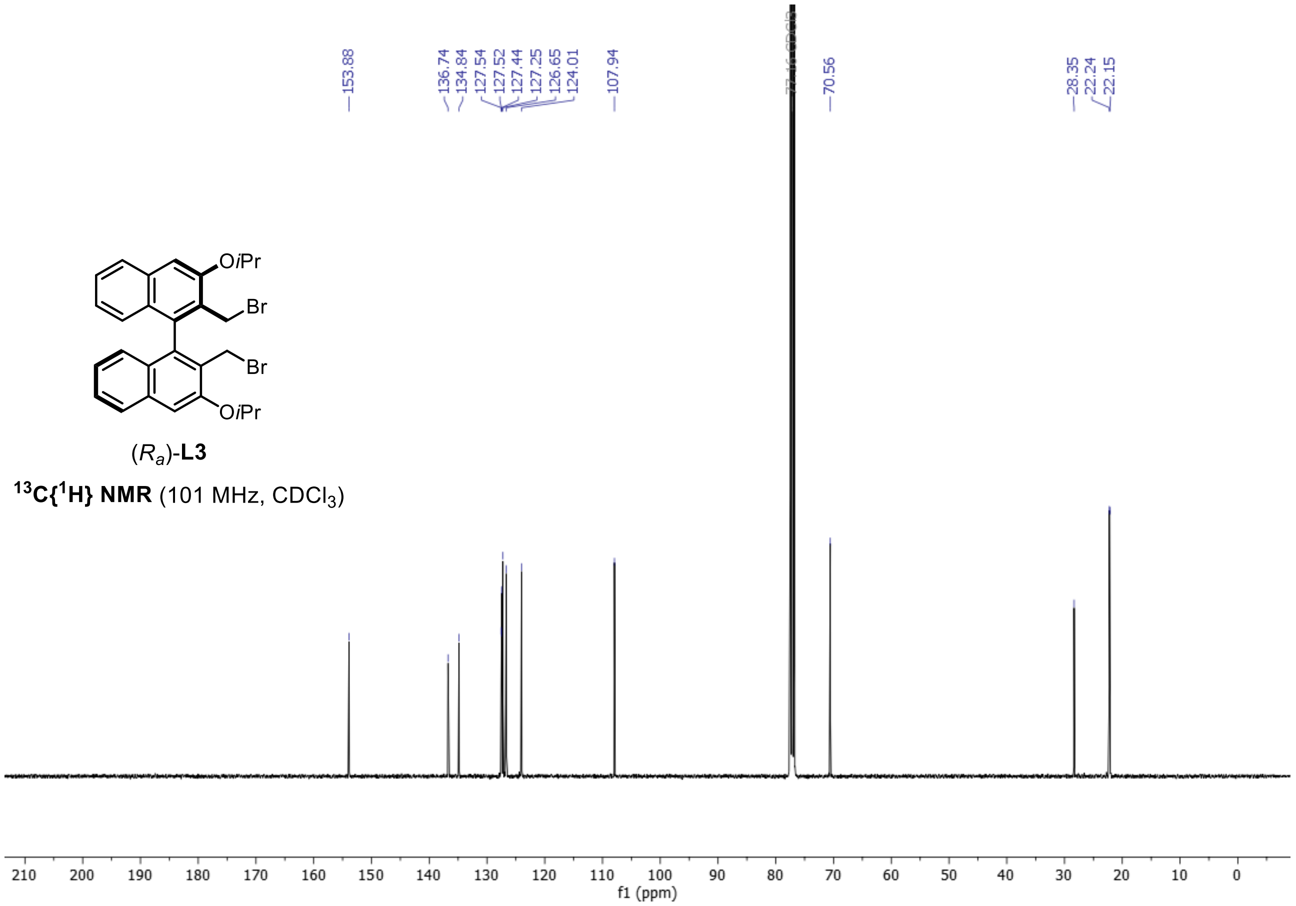

(R_a)-**L3**

^{13}C{^{1}H} NMR (101 MHz, $CDCl_3$)

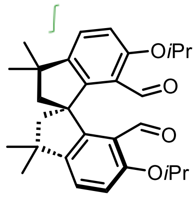

(*R*)-**SI30**

^{1}H NMR (400 MHz, $CDCl_3$)

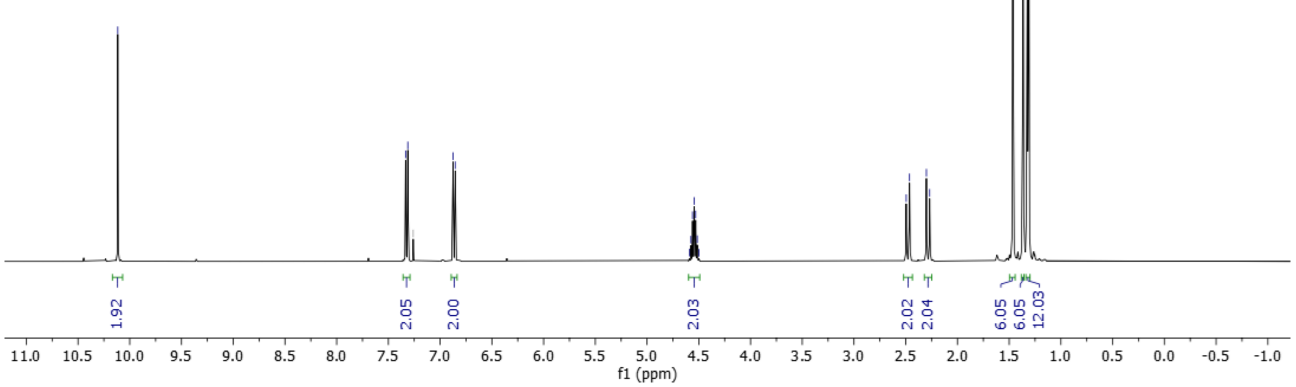

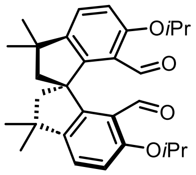

(*R*)-**SI30**

^{13}C{^{1}H} NMR (101 MHz, $CDCl_3$)

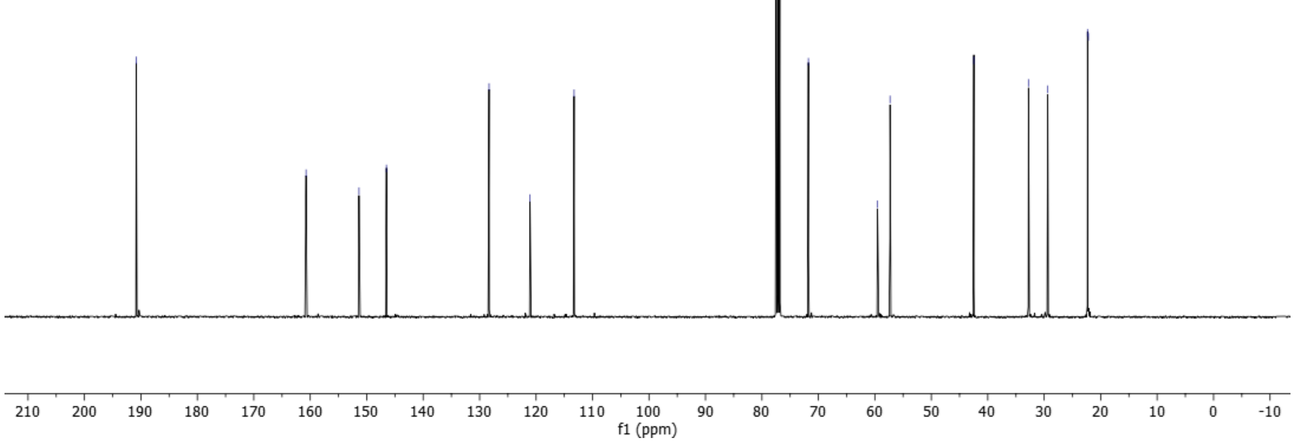

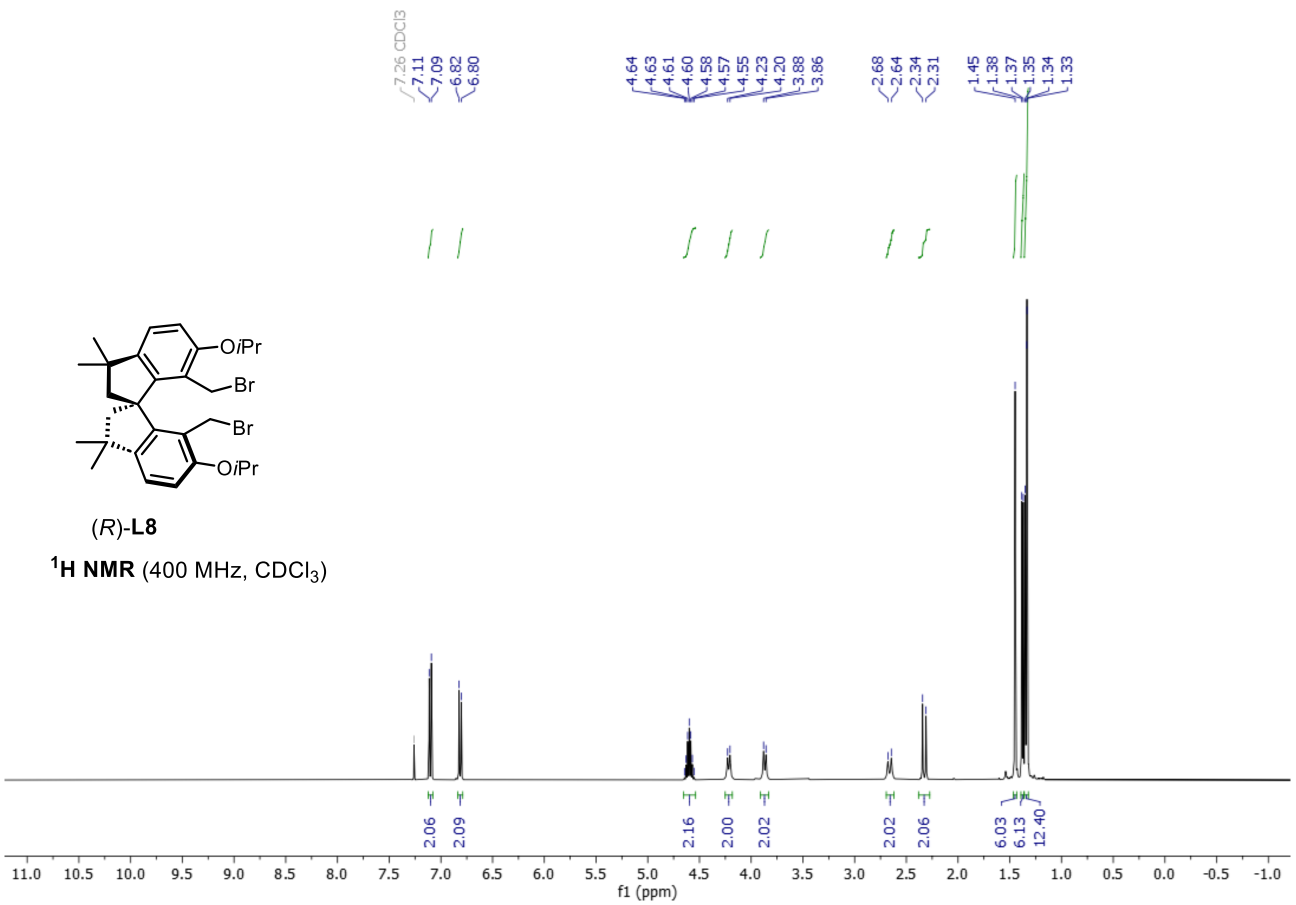
(R)-L8
1H NMR (400 MHz, CDCl3)
7.26 CDCl3
7.11
7.09
6.82
6.80
4.64
4.63
4.61
4.60
4.58
4.57
4.55
4.23
4.20
3.88
3.86
2.68
2.64
2.34
2.31
1.45
1.38
1.37
1.35
1.34
1.33
2.06
2.09
2.16
2.00
2.02
2.02
2.06
6.03
6.13
12.40
f1 (ppm)

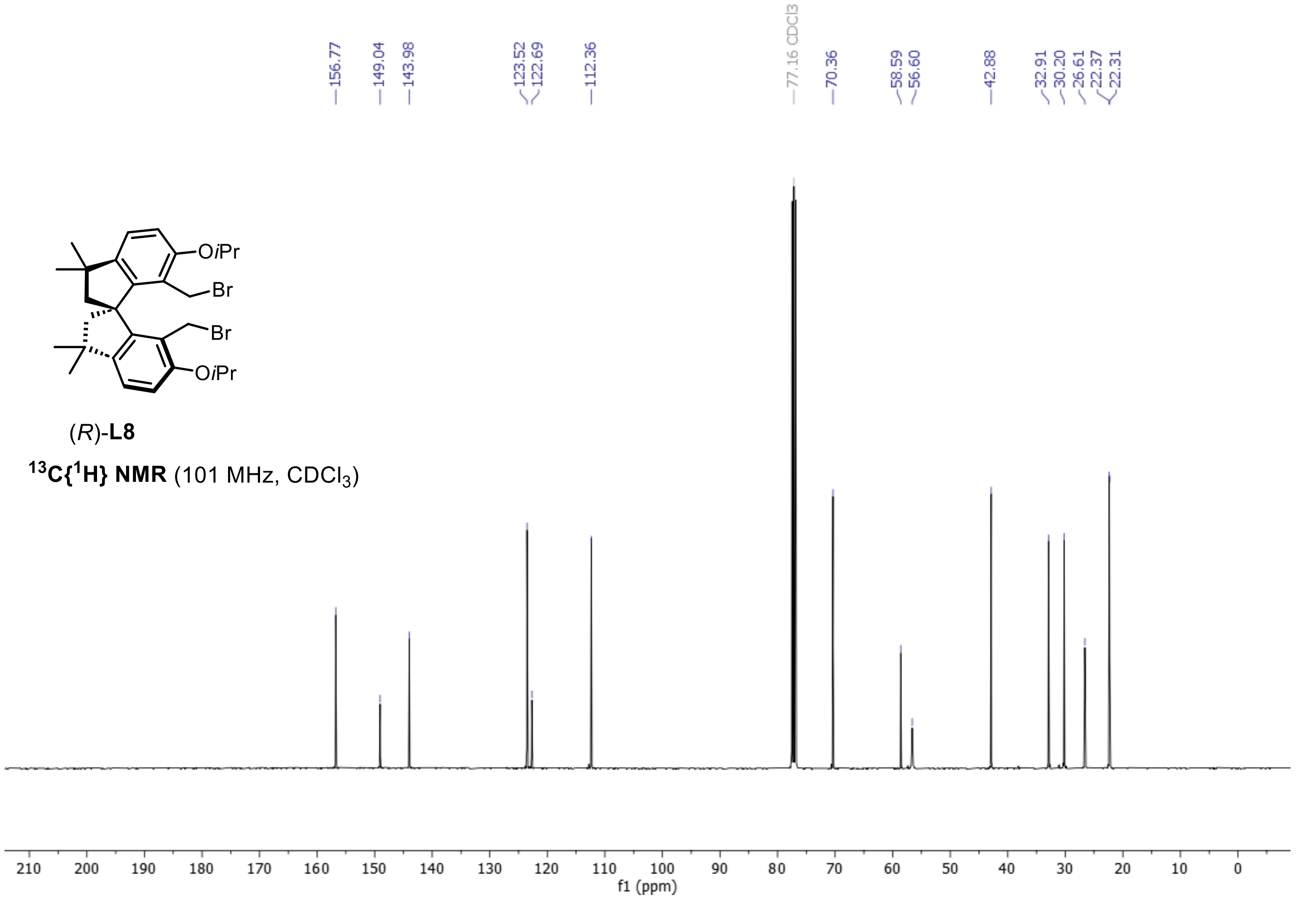
(R)-L8
13C{1H} NMR (101 MHz, CDCl3)
156.77
149.04
143.98
123.52
122.69
112.36
77.16 CDCl3
70.36
58.59
56.60
42.88
32.91
30.20
26.61
22.37
22.31
f1 (ppm)

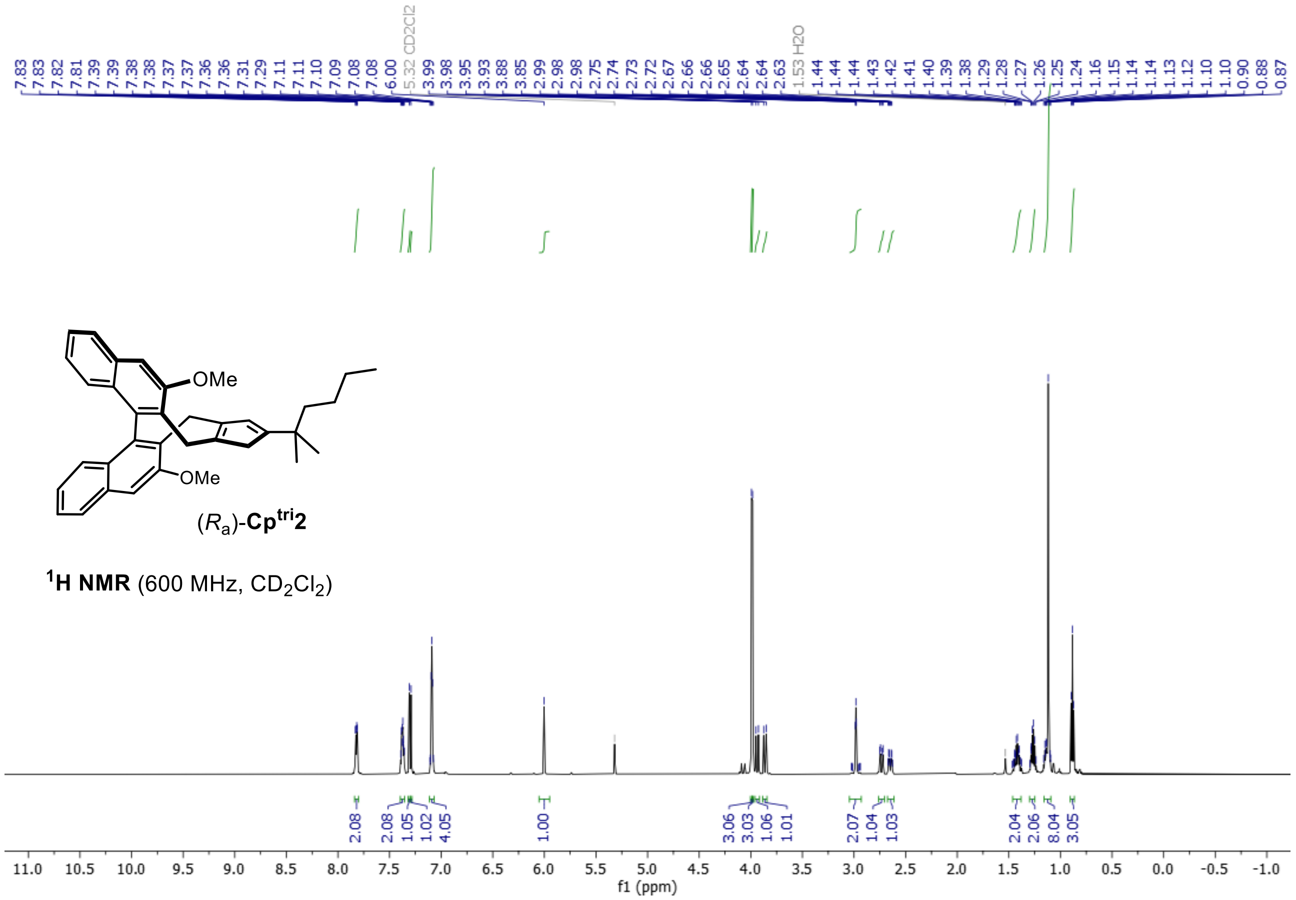
7.83 7.83 7.82 7.81 7.39 7.39 7.38 7.38 7.37 7.37 7.36 7.36 7.31 7.29 7.11 7.11 7.10 7.09 7.08 7.08 6.00 5.32 CD2Cl2 3.99 3.98 3.95 3.93 3.88 3.85 2.99 2.98 2.98 2.75 2.74 2.73 2.72 2.67 2.66 2.66 2.65 2.64 2.64 2.63 1.53 H2O 1.44 1.44 1.44 1.43 1.42 1.41 1.40 1.39 1.38 1.29 1.28 1.27 1.26 1.25 1.24 1.16 1.15 1.14 1.14 1.13 1.12 1.10 1.10 0.90 0.88 0.87
OMe
OMe
(R_a)-**Cp^{tri}2**
^{1}H NMR (600 MHz, CD_2Cl_2)
2.08 2.08 1.05 1.02 4.05 1.00 3.06 3.03 1.06 1.01 2.07 1.04 1.03 2.04 2.06 8.04 3.05
11.0 10.5 10.0 9.5 9.0 8.5 8.0 7.5 7.0 6.5 6.0 5.5 5.0 4.5 4.0 3.5 3.0 2.5 2.0 1.5 1.0 0.5 0.0 -0.5 -1.0
f1 (ppm)

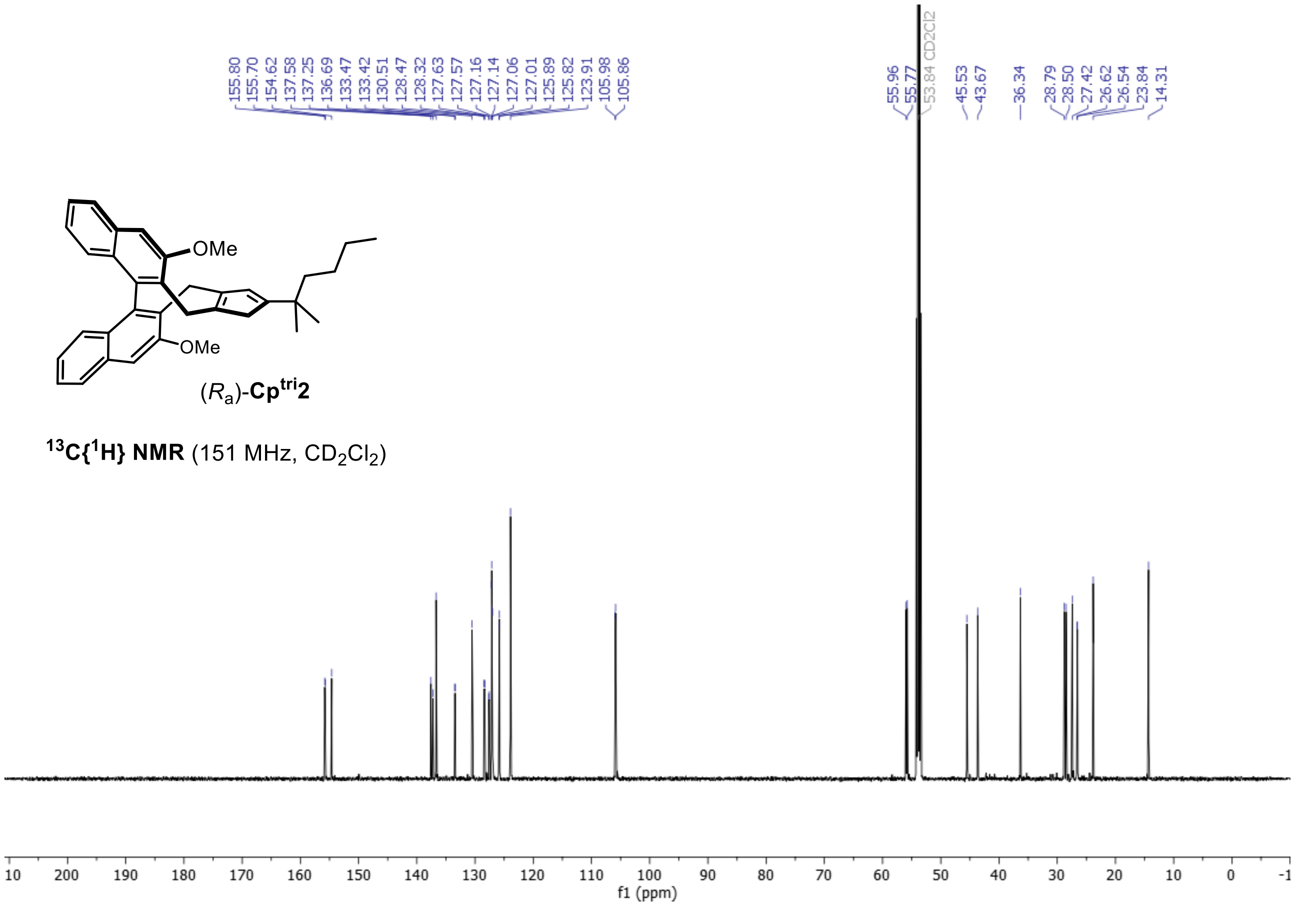
155.80 155.70 154.62 137.58 137.25 136.69 133.47 133.42 130.51 128.47 128.32 127.63 127.57 127.16 127.14 127.06 127.01 125.89 125.82 123.91 105.98 105.86
55.96 55.77 53.84 CD2Cl2 45.53 43.67 36.34 28.79 28.50 27.42 26.62 26.54 23.84 14.31
OMe
OMe
(R_a)-**Cp^{tri}2**
^{13}C{^{1}H} NMR (151 MHz, CD_2Cl_2)
10 200 190 180 170 160 150 140 130 120 110 100 90 80 70 60 50 40 30 20 10 0 -1
f1 (ppm)

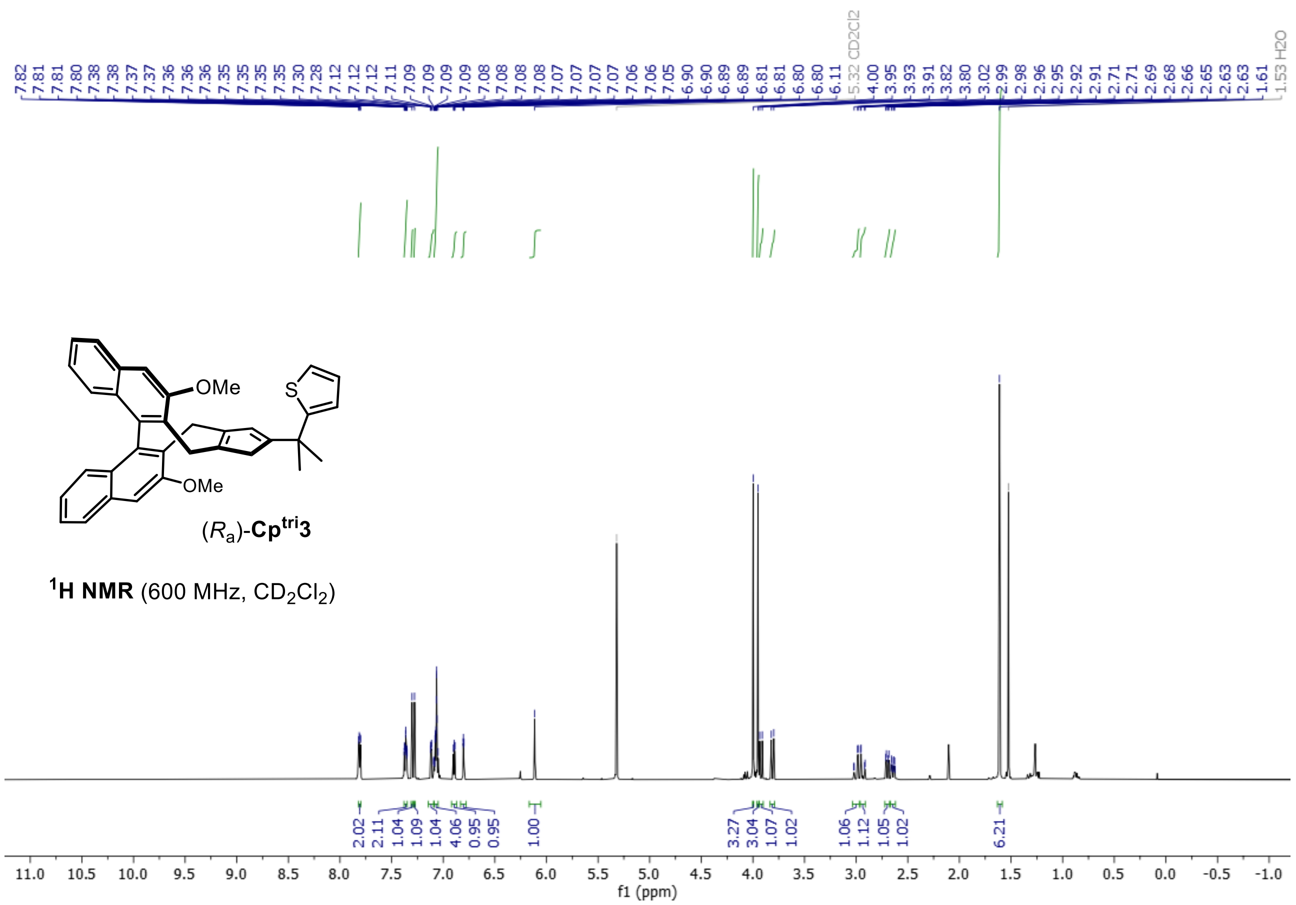
7.82 7.81 7.80 7.38 7.38 7.37 7.37 7.36 7.36 7.36 7.35 7.35 7.35 7.35 7.30 7.28 7.12 7.12 7.11 7.09 7.09 7.09 7.09 7.08 7.08 7.08 7.07 7.07 7.07 7.06 7.06 7.05 6.90 6.90 6.89 6.89 6.81 6.81 6.80 6.80 6.11 5.32 CD2Cl2 4.00 3.95 3.93 3.91 3.82 3.80 3.02 2.99 2.98 2.96 2.95 2.92 2.91 2.71 2.71 2.69 2.68 2.66 2.65 2.63 2.63 1.61 1.53 H2O
OMe
S
OMe
(Ra)-Cptri3
1H NMR (600 MHz, CD2Cl2)
2.02 2.11 1.04 1.09 1.04 4.06 0.95 0.95 1.00 3.27 3.04 1.07 1.02 1.06 1.12 1.05 1.02 6.21
11.0 10.5 10.0 9.5 9.0 8.5 8.0 7.5 7.0 6.5 6.0 5.5 5.0 4.5 4.0 3.5 3.0 2.5 2.0 1.5 1.0 0.5 0.0 -0.5 -1.0
f1 (ppm)

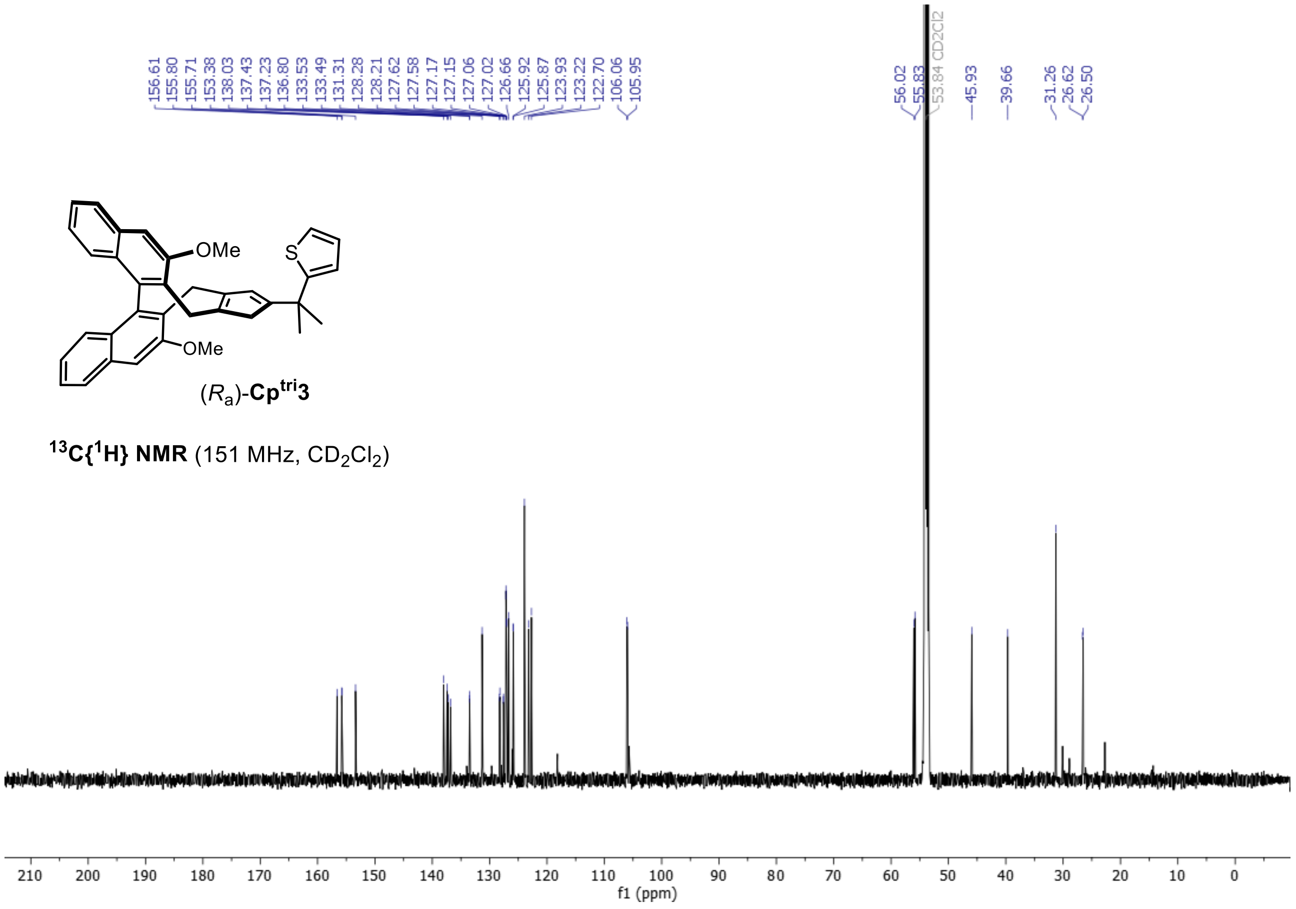
156.61 155.80 155.71 153.38 138.03 137.43 137.23 136.80 133.53 133.49 131.31 128.28 128.21 127.62 127.58 127.17 127.15 127.06 127.02 126.66 125.92 125.87 123.93 123.22 122.70 106.06 105.95
56.02 55.83 53.84 CD2Cl2 45.93 39.66 31.26 26.62 26.50
OMe
S
OMe
(Ra)-Cptri3
13C{1H} NMR (151 MHz, CD2Cl2)
210 200 190 180 170 160 150 140 130 120 110 100 90 80 70 60 50 40 30 20 10 0
f1 (ppm)

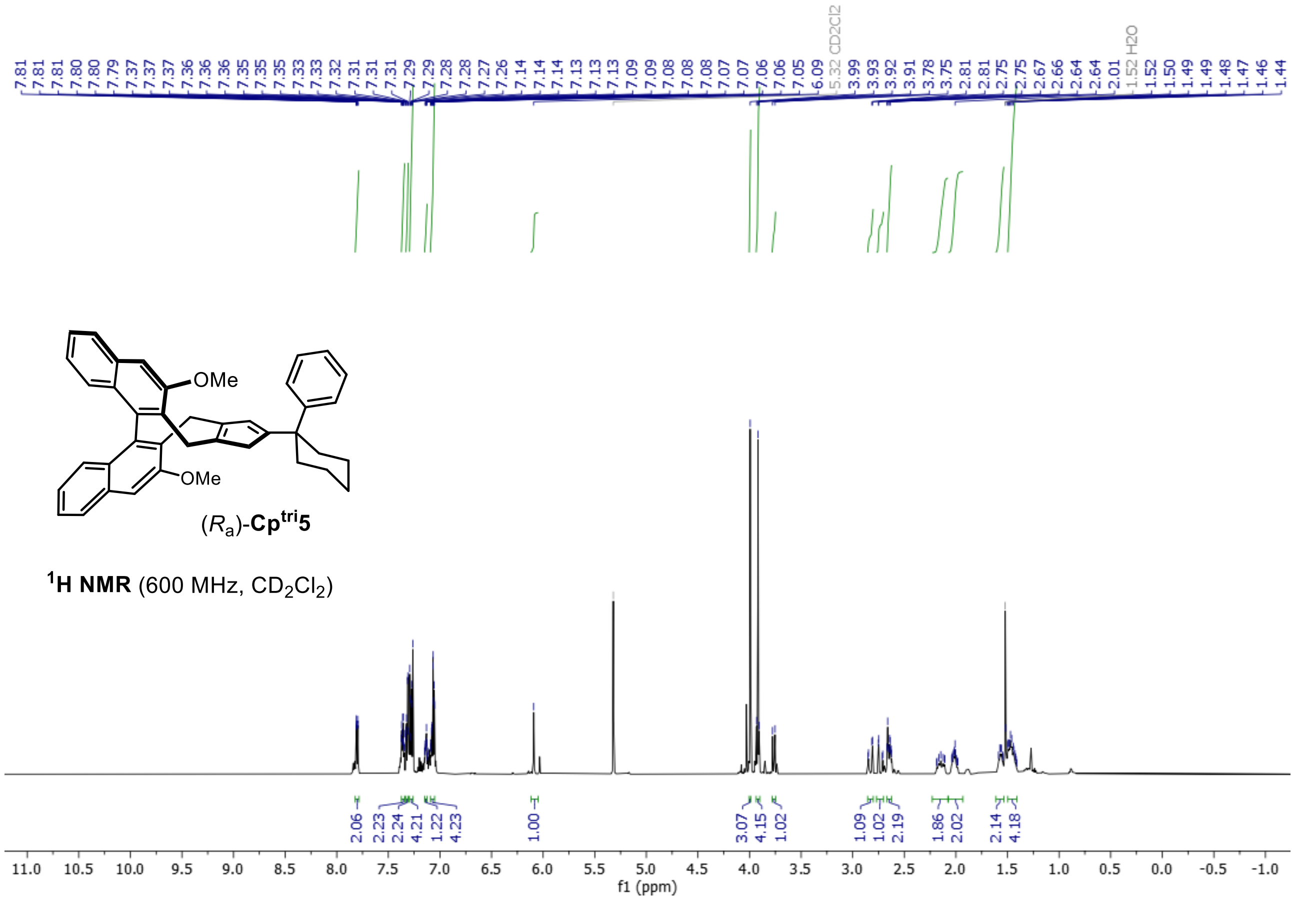
(R_a)-Cp^tri 5
1H NMR (600 MHz, CD2Cl2)
f1 (ppm)

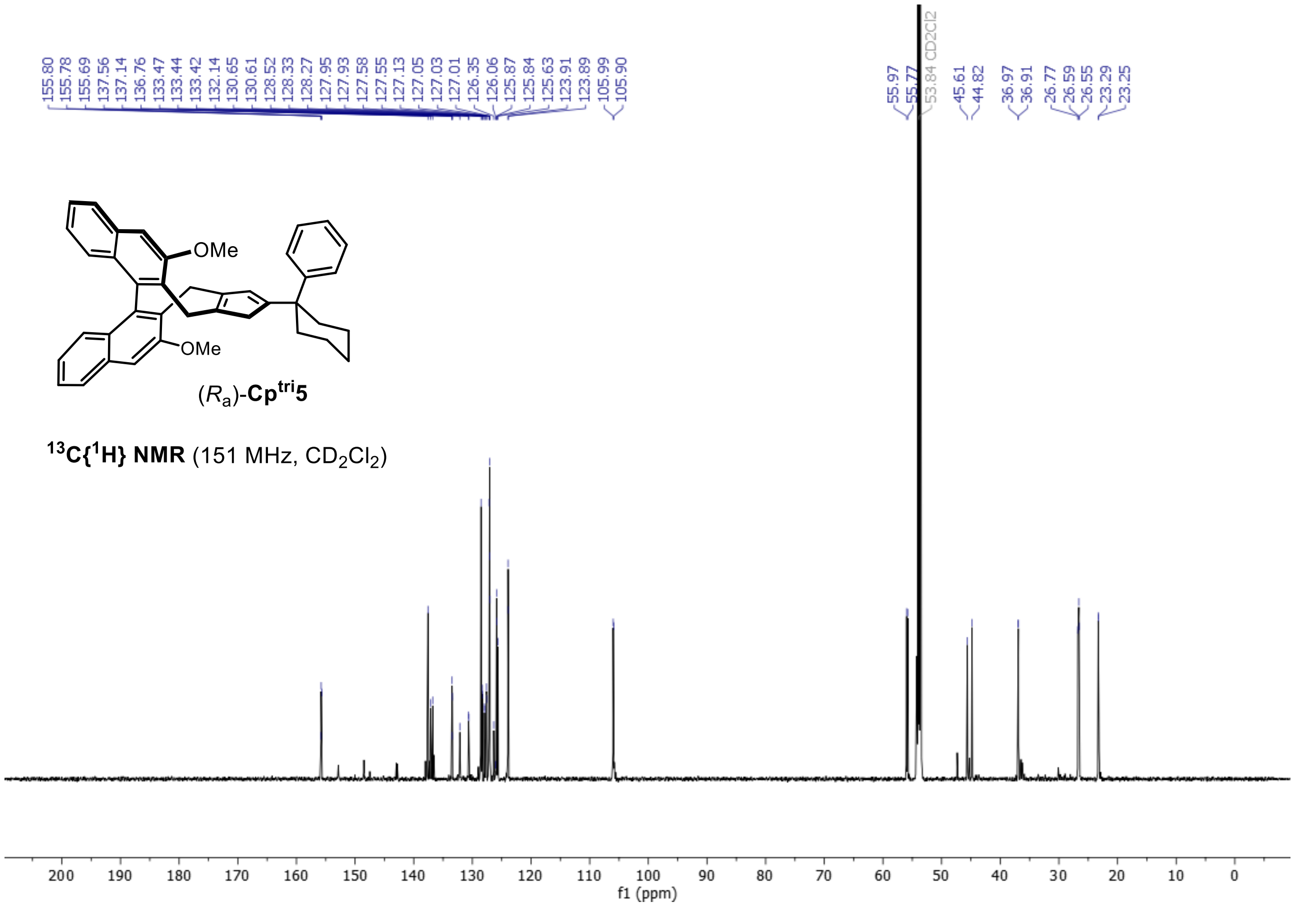
(R_a)-Cp^tri 5
13C{1H} NMR (151 MHz, CD2Cl2)
f1 (ppm)

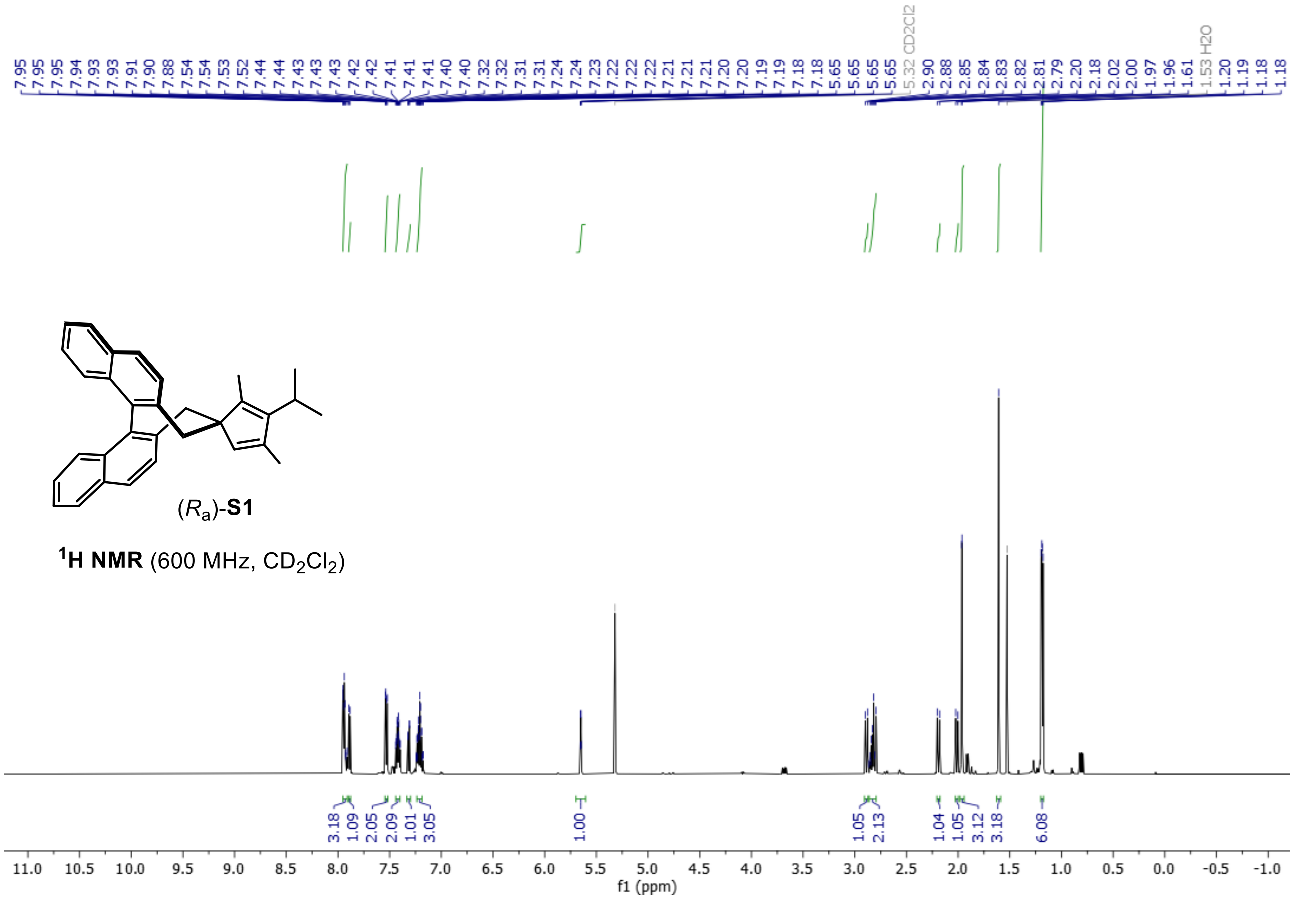
7.95 7.95 7.95 7.94 7.93 7.93 7.91 7.90 7.88 7.54 7.54 7.53 7.52 7.44 7.44 7.43 7.43 7.43 7.42 7.42 7.41 7.41 7.41 7.40 7.40 7.32 7.32 7.31 7.31 7.24 7.24 7.23 7.22 7.22 7.22 7.21 7.21 7.21 7.20 7.20 7.19 7.19 7.18 7.18 5.65 5.65 5.65
5.32 CD2Cl2
2.90 2.88 2.85 2.84 2.83 2.82 2.81 2.79 2.20 2.18 2.02 2.00 1.97 1.96 1.61
1.53 H2O
1.20 1.19 1.18 1.18
(Ra)-S1
1H NMR (600 MHz, CD2Cl2)
3.18 1.09 2.05 2.09 1.01 3.05 1.00 1.05 2.13 1.04 1.05 3.12 3.18 6.08
11.0 10.5 10.0 9.5 9.0 8.5 8.0 7.5 7.0 6.5 6.0 5.5 5.0 4.5 4.0 3.5 3.0 2.5 2.0 1.5 1.0 0.5 0.0 -0.5 -1.0
f1 (ppm)

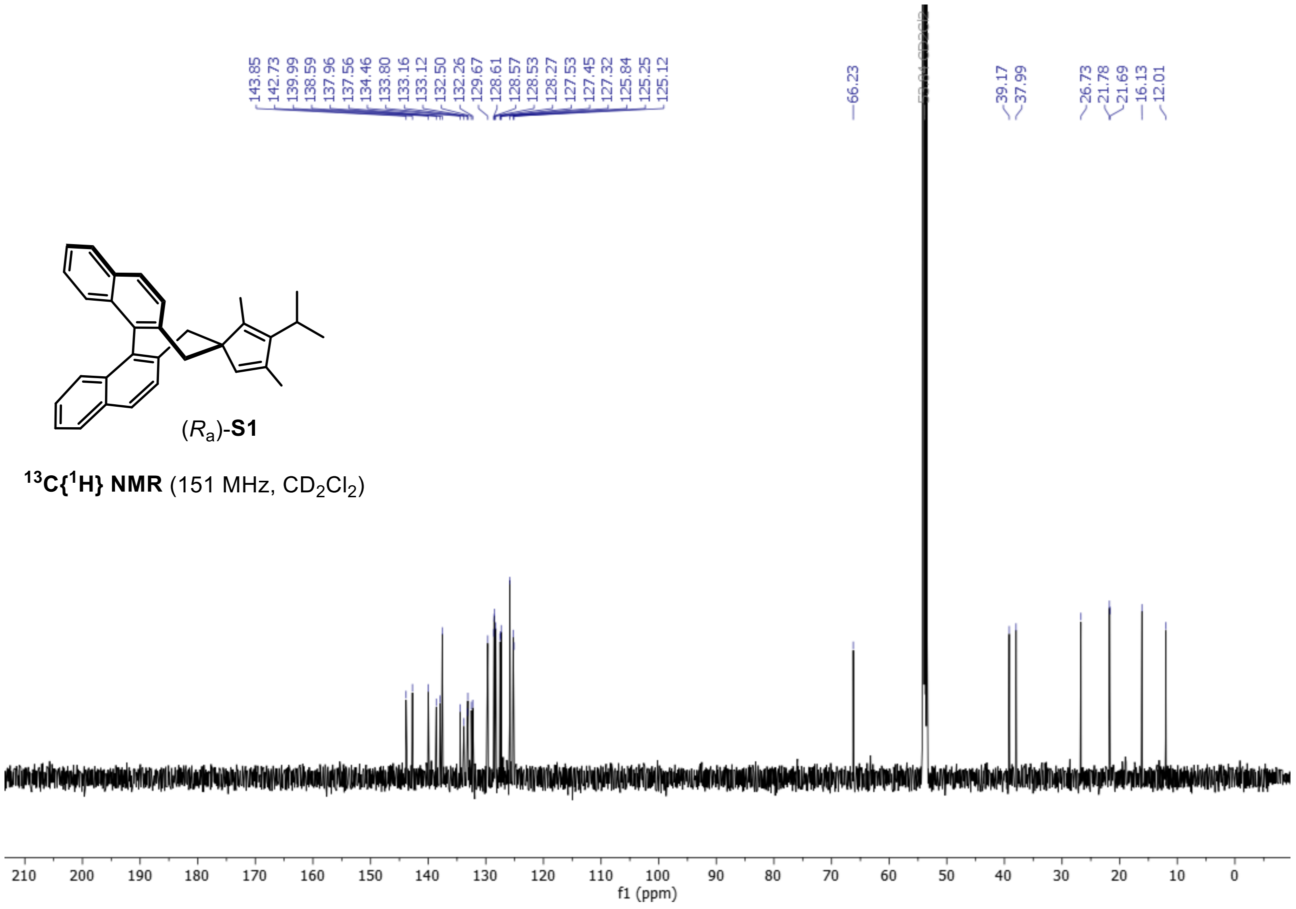
143.85 142.73 139.99 138.59 137.96 137.56 134.46 133.80 133.16 133.12 132.50 132.26 129.67 128.61 128.57 128.53 128.27 127.53 127.45 127.32 125.84 125.25 125.12
66.23
39.17 37.99
26.73 21.78 21.69 16.13 12.01
(Ra)-S1
13C{1H} NMR (151 MHz, CD2Cl2)
210 200 190 180 170 160 150 140 130 120 110 100 90 80 70 60 50 40 30 20 10 0
f1 (ppm)

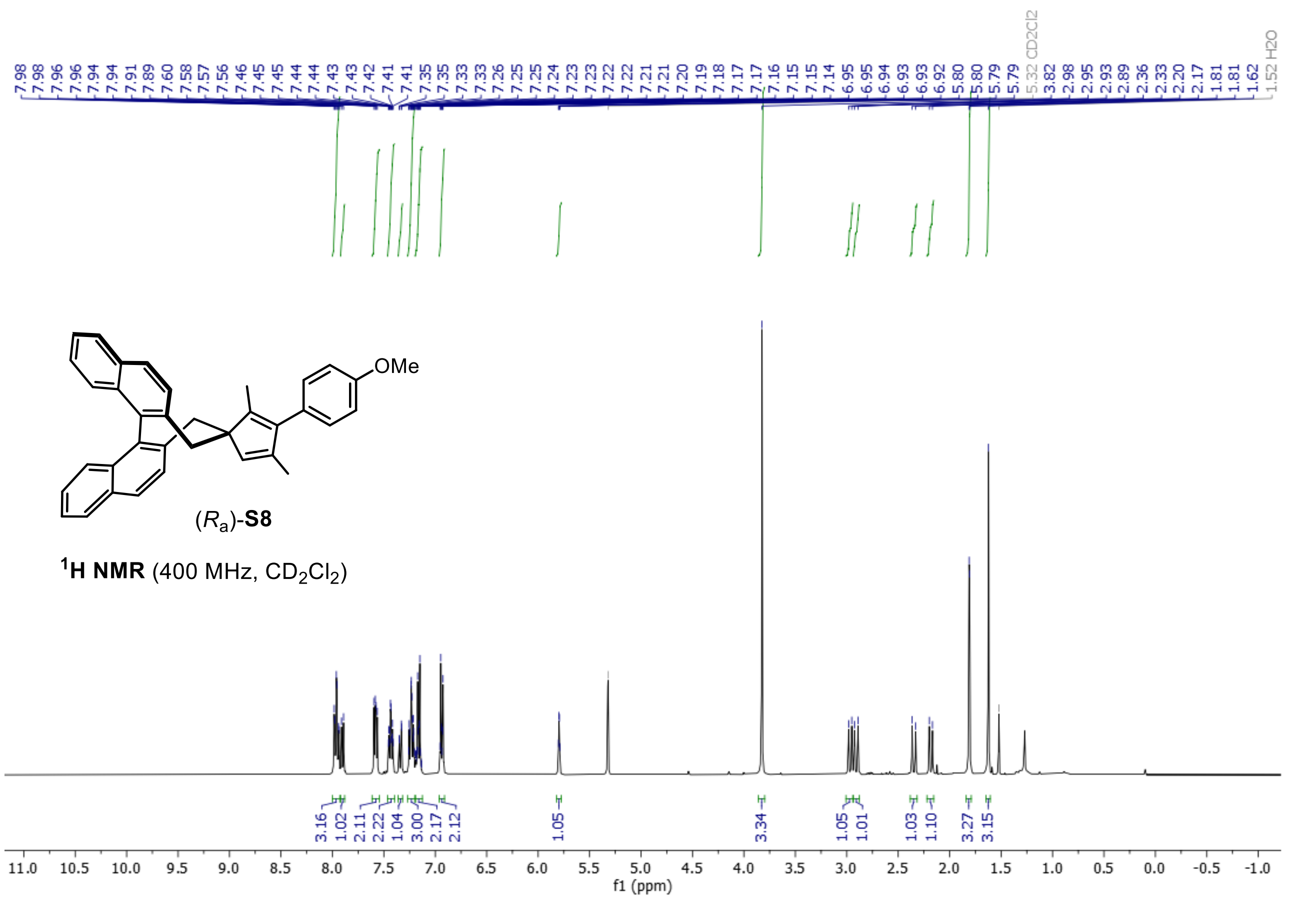
OMe
(Ra)-S8
1H NMR (400 MHz, CD2Cl2)
f1 (ppm)

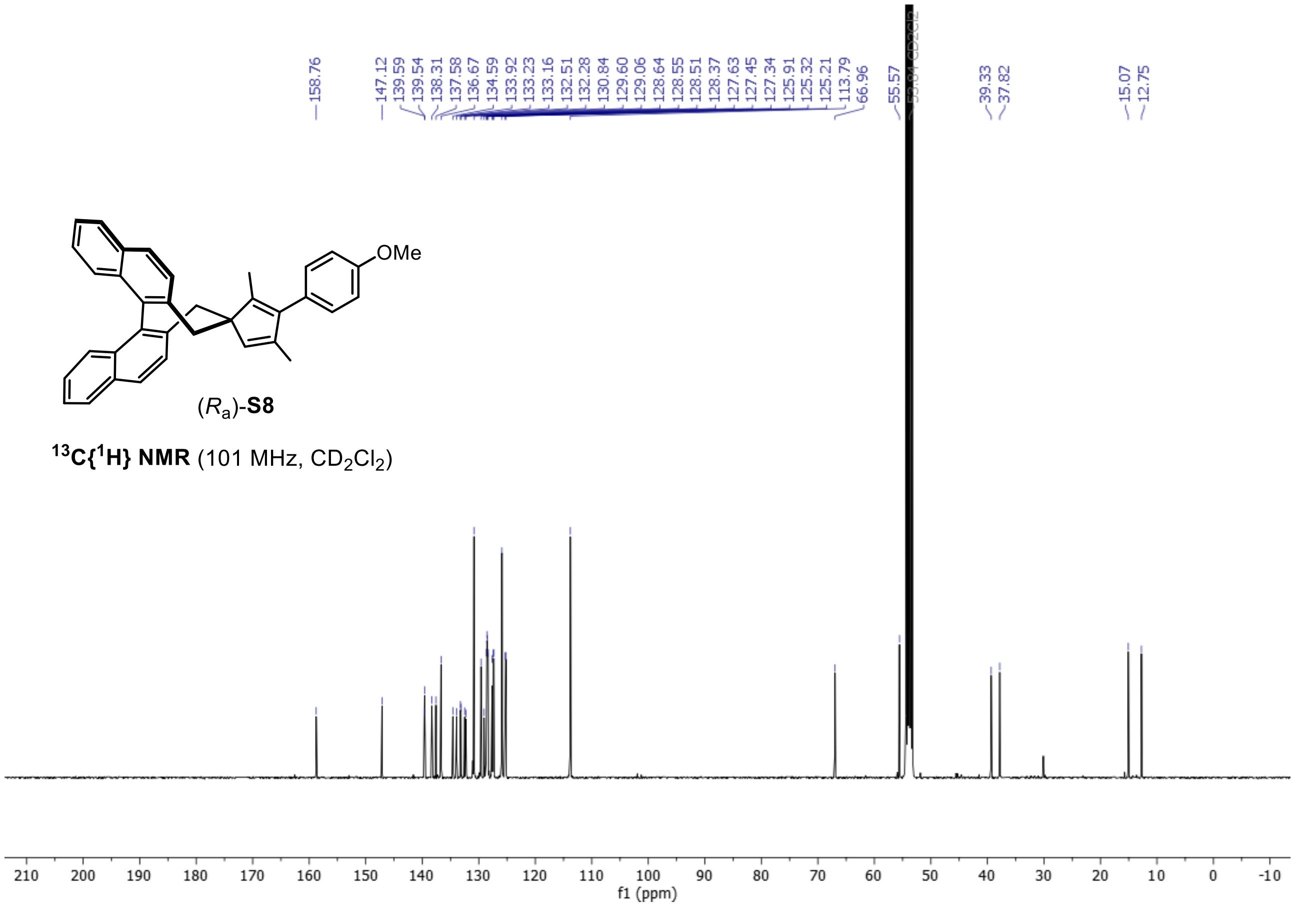
OMe
(Ra)-S8
13C{1H} NMR (101 MHz, CD2Cl2)
f1 (ppm)

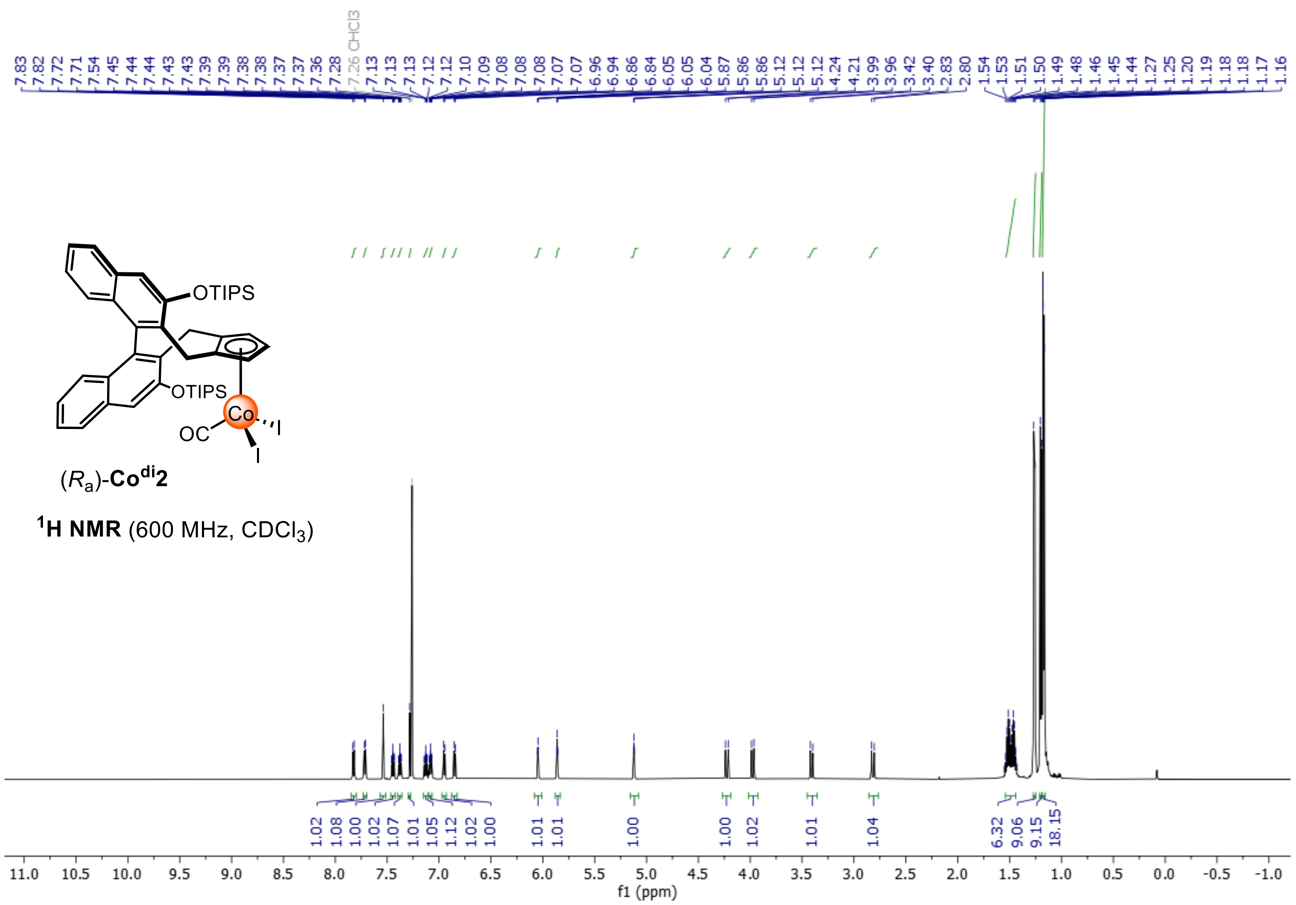
7.83 7.82 7.72 7.71 7.54 7.45 7.44 7.44 7.43 7.43 7.39 7.39 7.38 7.38 7.37 7.37 7.36 7.28 7.26 CHCl3 7.13 7.13 7.13 7.12 7.12 7.10 7.09 7.08 7.08 7.08 7.07 7.07 6.96 6.94 6.86 6.84 6.05 6.05 6.04 5.87 5.86 5.86 5.12 5.12 5.12 4.24 4.21 3.99 3.96 3.42 3.40 2.83 2.80 1.54 1.53 1.51 1.50 1.49 1.48 1.46 1.45 1.44 1.27 1.25 1.20 1.19 1.18 1.18 1.17 1.16
OTIPS
OTIPS
Co
OC
I
I
(R_a)-**Co^{di}2**
^{1}H NMR (600 MHz, $CDCl_3$)
1.02 1.08 1.00 1.02 1.07 1.01 1.05 1.12 1.02 1.00 1.01 1.01 1.00 1.00 1.02 1.01 1.04 6.32 9.06 9.15 18.15
11.0 10.5 10.0 9.5 9.0 8.5 8.0 7.5 7.0 6.5 6.0 5.5 5.0 4.5 4.0 3.5 3.0 2.5 2.0 1.5 1.0 0.5 0.0 -0.5 -1.0
f1 (ppm)

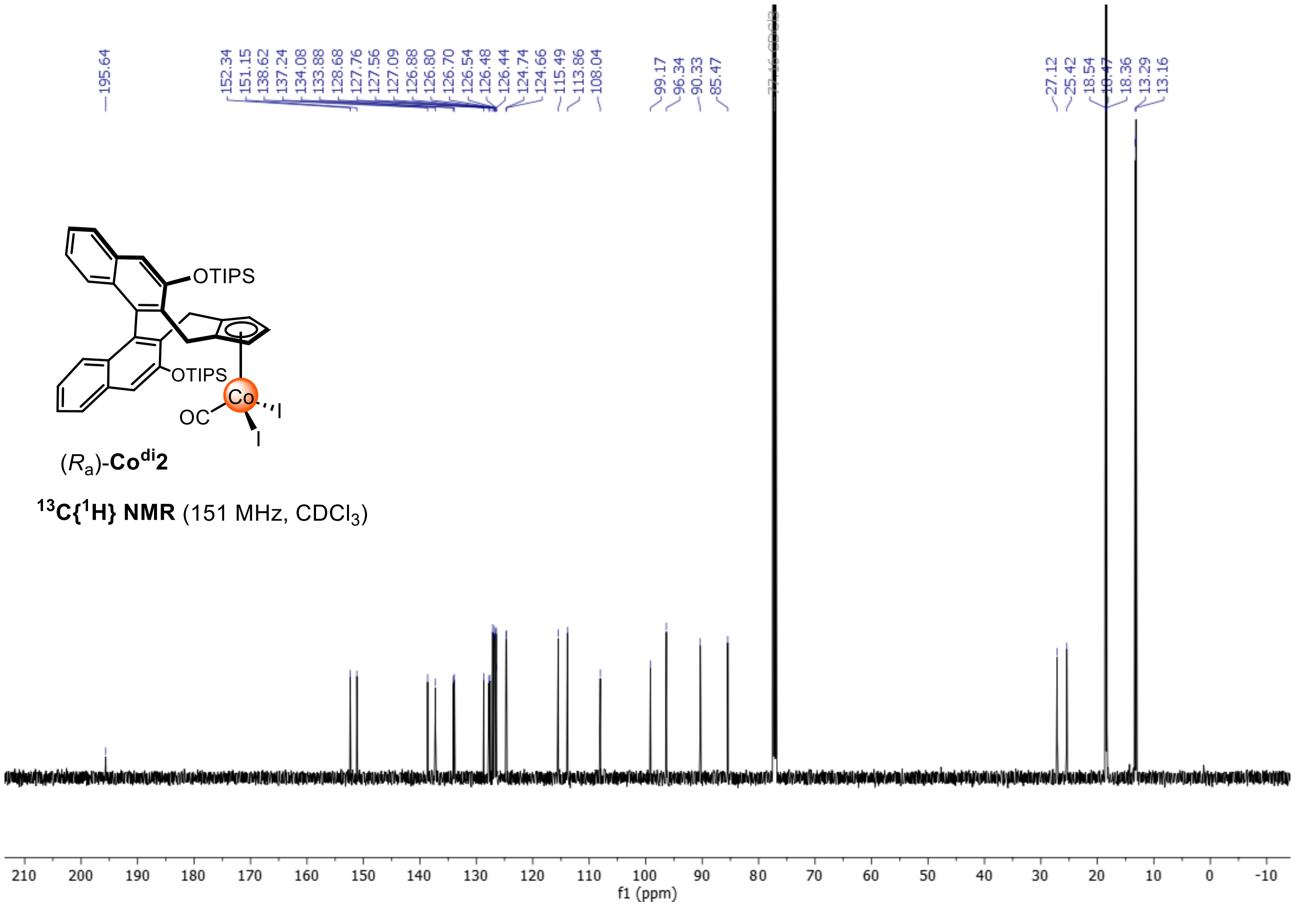
195.64
152.34 151.15 138.62 137.24 134.08 133.88 128.68 127.76 127.56 127.09 126.88 126.80 126.70 126.54 126.48 126.44 124.74 124.66 115.49 113.86 108.04
99.17 96.34 90.33 85.47
77.16 CDCl3
27.12 25.42 18.54 18.47 18.36 13.29 13.16
OTIPS
OTIPS
Co
OC
I
I
(R_a)-**Co^{di}2**
^{13}C{^{1}H} NMR (151 MHz, $CDCl_3$)
210 200 190 180 170 160 150 140 130 120 110 100 90 80 70 60 50 40 30 20 10 0 -10
f1 (ppm)

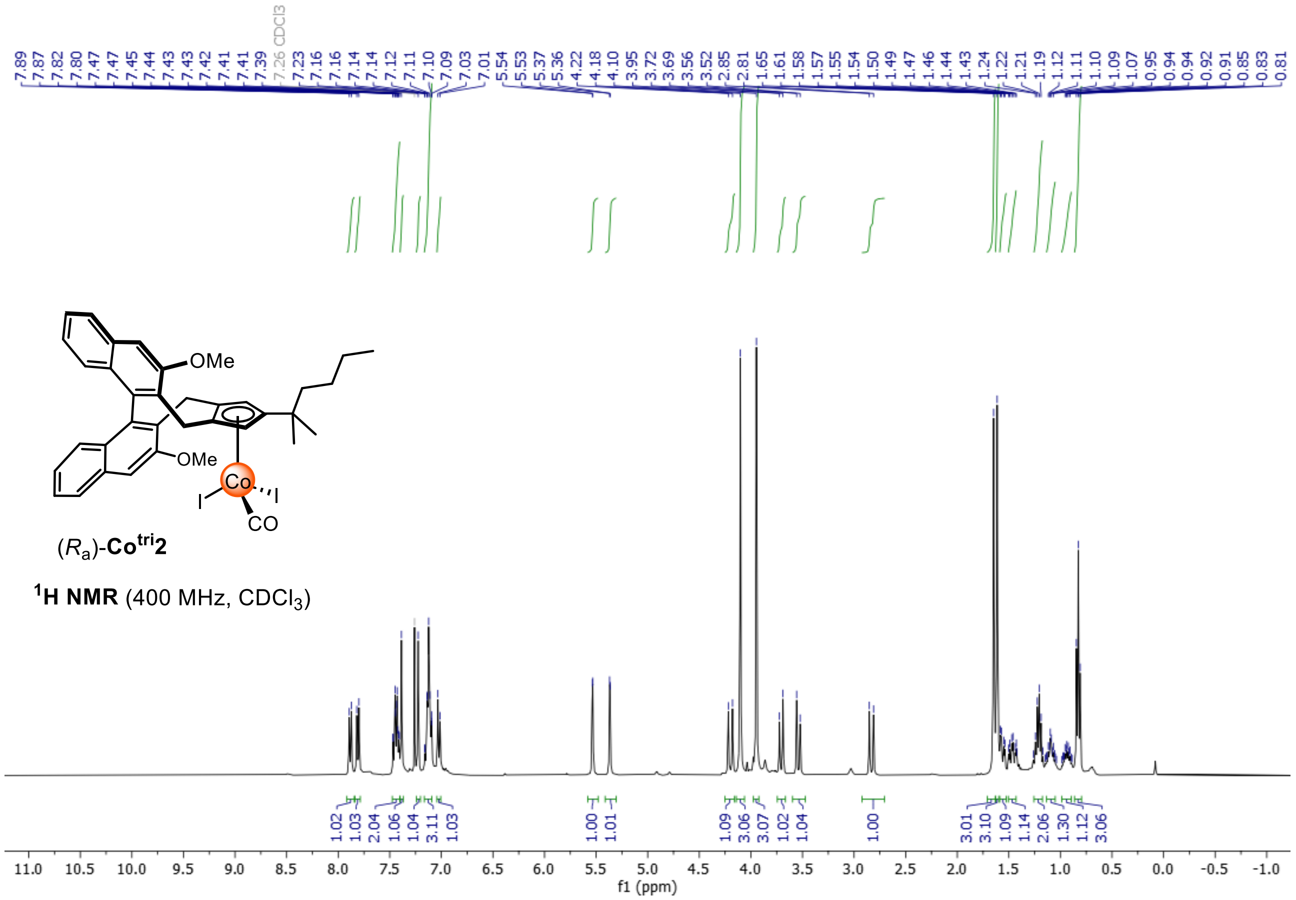
7.89 7.87 7.82 7.80 7.47 7.47 7.45 7.44 7.43 7.43 7.42 7.41 7.41 7.39 7.26 CDCl3 7.23 7.16 7.16 7.14 7.14 7.12 7.11 7.10 7.09 7.03 7.01 5.54 5.53 5.37 5.36 4.22 4.18 4.10 3.95 3.72 3.69 3.56 3.52 2.85 2.81 1.65 1.61 1.58 1.57 1.55 1.54 1.50 1.49 1.47 1.46 1.44 1.43 1.24 1.22 1.21 1.19 1.12 1.11 1.10 1.09 1.07 0.95 0.94 0.94 0.92 0.91 0.85 0.83 0.81
OMe
OMe
Co
I
I
CO
(R_a)-**Co^tri2**
^{1}H NMR (400 MHz, $CDCl_3$)
1.02 1.03 2.04 1.06 1.04 3.11 1.03 1.00 1.01 1.09 3.06 3.07 1.02 1.04 1.00 3.01 3.10 1.09 1.14 2.06 1.30 1.12 3.06
11.0 10.5 10.0 9.5 9.0 8.5 8.0 7.5 7.0 6.5 6.0 5.5 5.0 4.5 4.0 3.5 3.0 2.5 2.0 1.5 1.0 0.5 0.0 -0.5 -1.0
f1 (ppm)

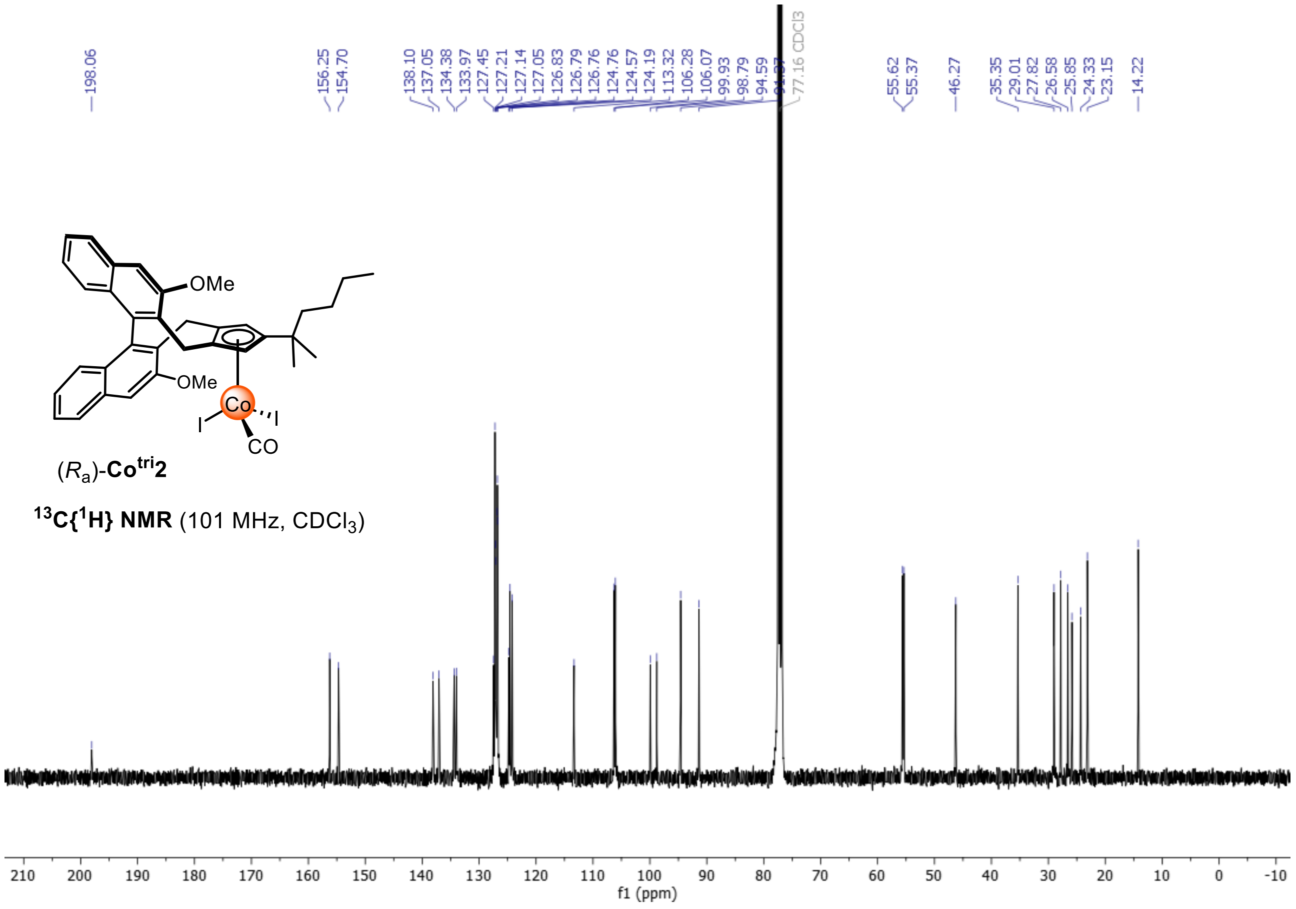
198.06 156.25 154.70 138.10 137.05 134.38 133.97 127.45 127.21 127.14 127.05 126.83 126.79 126.76 124.76 124.57 124.19 113.32 106.28 106.07 99.93 98.79 94.59 91.87 77.16 CDCl3 55.62 55.37 46.27 35.35 29.01 27.82 26.58 25.85 24.33 23.15 14.22
OMe
OMe
Co
I
I
CO
(R_a)-**Co^tri2**
^{13}C{^{1}H} NMR (101 MHz, $CDCl_3$)
210 200 190 180 170 160 150 140 130 120 110 100 90 80 70 60 50 40 30 20 10 0 -10
f1 (ppm)

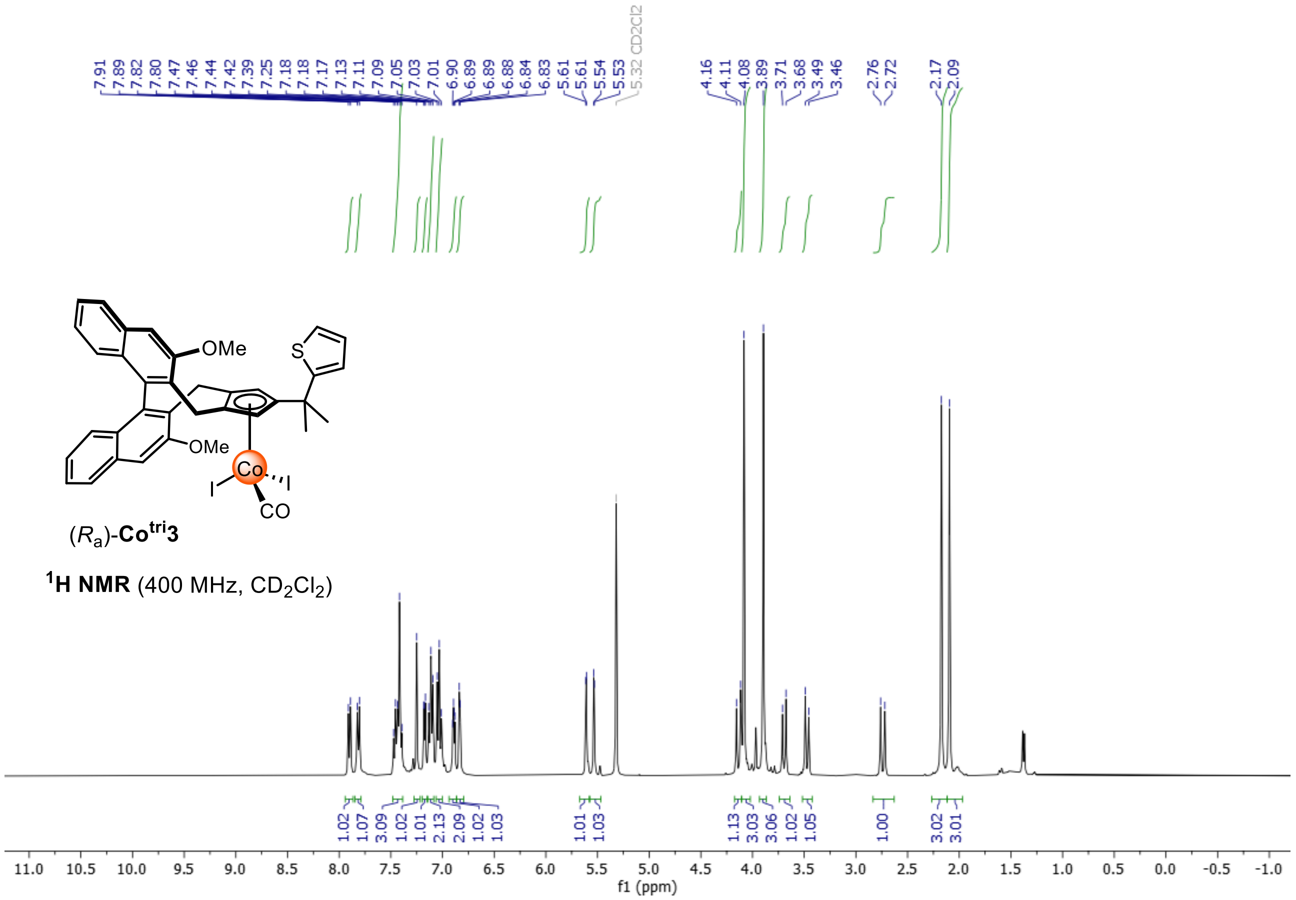
7.91 7.89 7.82 7.80 7.47 7.46 7.44 7.42 7.39 7.25 7.18 7.18 7.17 7.13 7.11 7.09 7.05 7.03 7.01 6.90 6.89 6.89 6.88 6.84 6.83 5.61 5.61 5.54 5.53 5.32 CD2Cl2 4.16 4.11 4.08 3.89 3.71 3.68 3.49 3.46 2.76 2.72 2.17 2.09
OMe
S
OMe
Co
I
I
CO
(Ra)-Cotri3
1H NMR (400 MHz, CD2Cl2)
1.02 1.07 3.09 1.02 1.01 2.13 2.09 1.02 1.03 1.01 1.03 1.13 3.03 3.06 1.02 1.05 1.00 3.02 3.01
11.0 10.5 10.0 9.5 9.0 8.5 8.0 7.5 7.0 6.5 6.0 5.5 5.0 4.5 4.0 3.5 3.0 2.5 2.0 1.5 1.0 0.5 0.0 -0.5 -1.0
f1 (ppm)

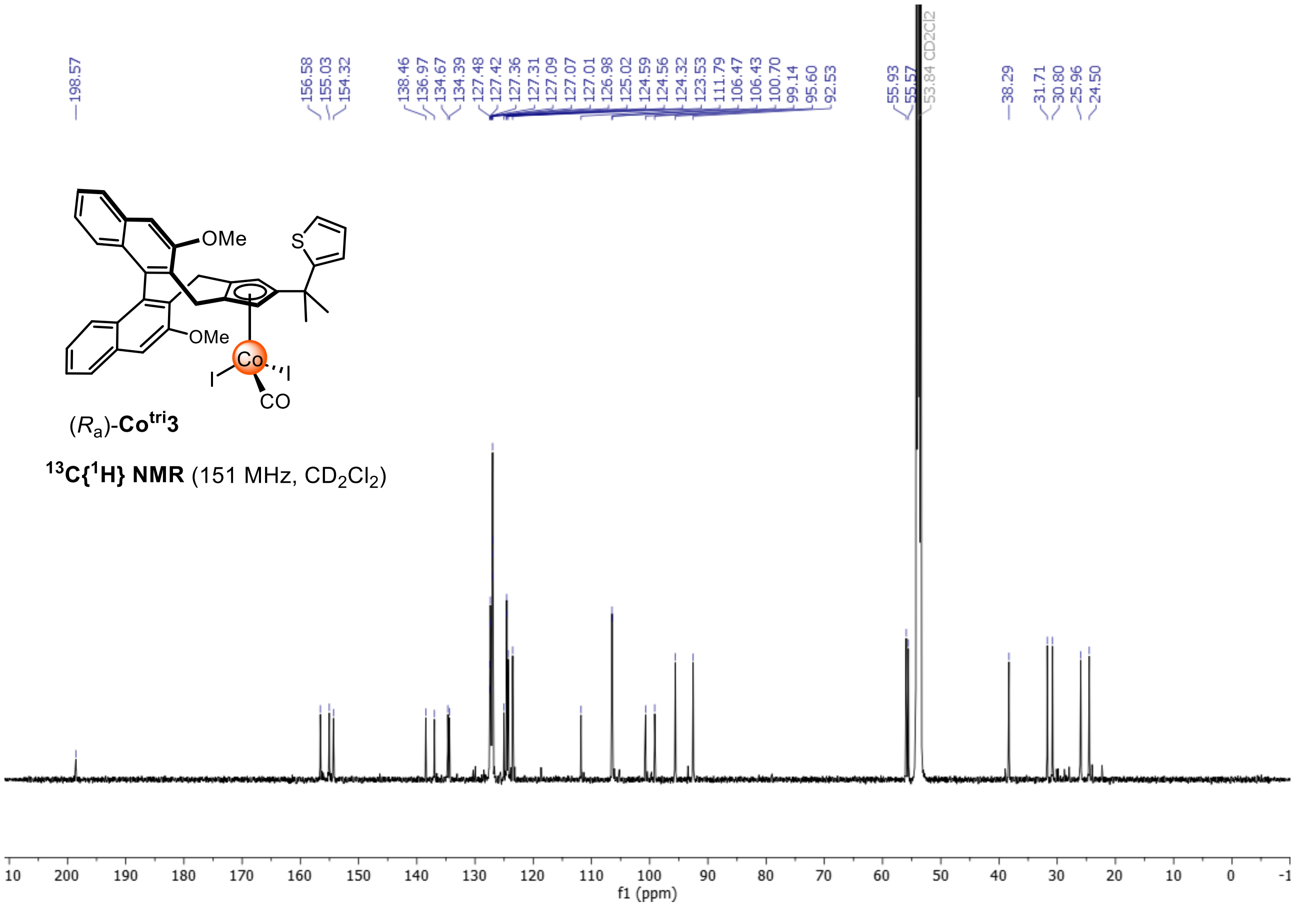
198.57 156.58 155.03 154.32 138.46 136.97 134.67 134.39 127.48 127.42 127.36 127.31 127.09 127.07 127.01 126.98 125.02 124.59 124.56 124.32 123.53 111.79 106.47 106.43 100.70 99.14 95.60 92.53 55.93 55.57 53.84 CD2Cl2 38.29 31.71 30.80 25.96 24.50
OMe
S
OMe
Co
I
I
CO
(Ra)-Cotri3
13C{1H} NMR (151 MHz, CD2Cl2)
10 200 190 180 170 160 150 140 130 120 110 100 90 80 70 60 50 40 30 20 10 0 -1
f1 (ppm)

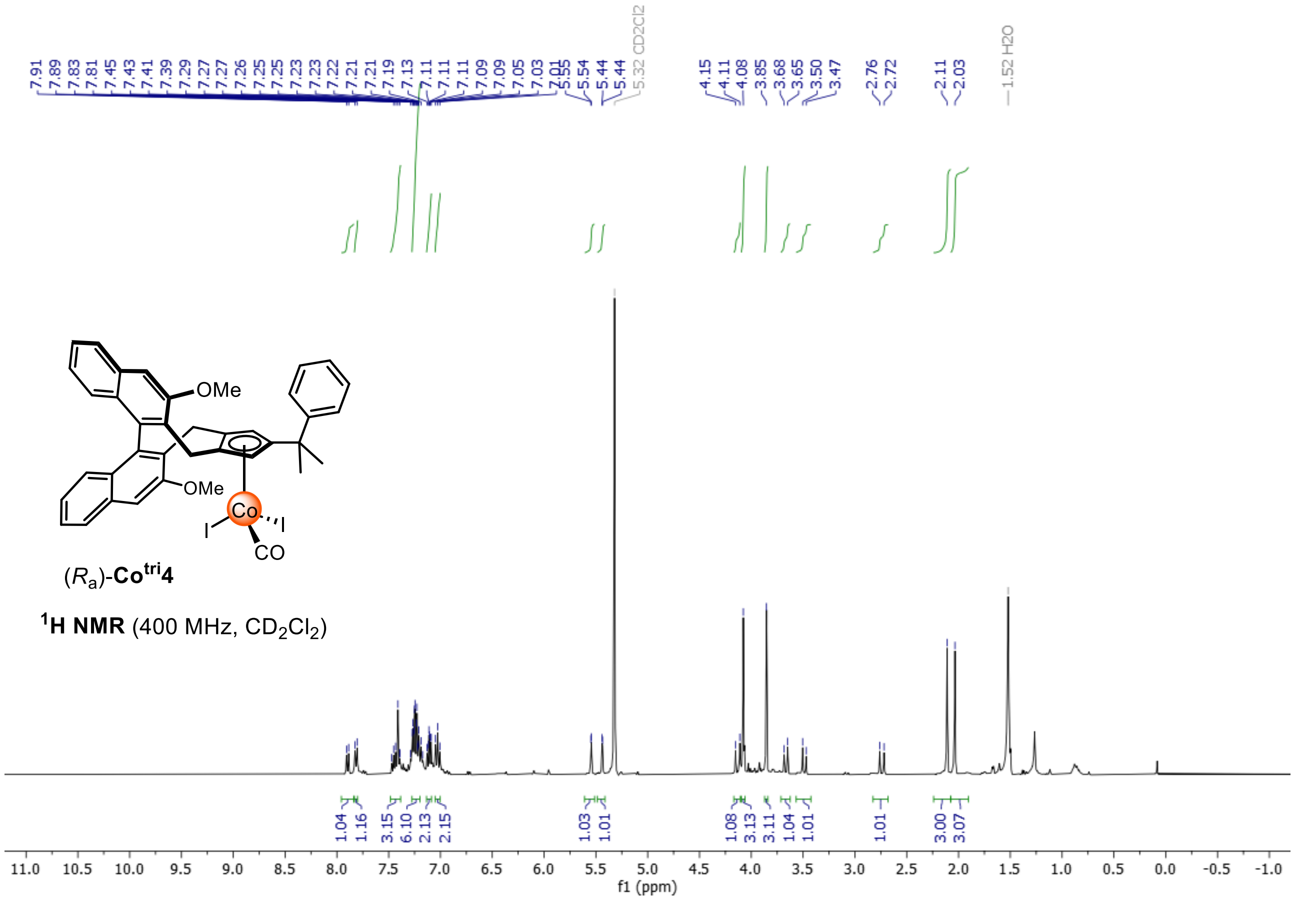
7.91
7.89
7.83
7.81
7.45
7.43
7.41
7.39
7.29
7.27
7.27
7.26
7.25
7.25
7.23
7.23
7.22
7.21
7.21
7.19
7.13
7.11
7.11
7.11
7.09
7.09
7.05
7.03
7.01
5.55
5.54
5.44
5.44
5.32 CD2Cl2
4.15
4.11
4.08
3.85
3.68
3.65
3.50
3.47
2.76
2.72
2.11
2.03
1.52 H2O
OMe
OMe
Co
I
I
CO
(R_a)-**Co**[tri]**4**
^{1}H NMR (400 MHz, CD_2Cl_2)
1.04
1.16
3.15
6.10
2.13
2.15
1.03
1.01
1.08
3.13
3.11
1.04
1.01
1.01
3.00
3.07
11.0 10.5 10.0 9.5 9.0 8.5 8.0 7.5 7.0 6.5 6.0 5.5 5.0 4.5 4.0 3.5 3.0 2.5 2.0 1.5 1.0 0.5 0.0 -0.5 -1.0
f1 (ppm)

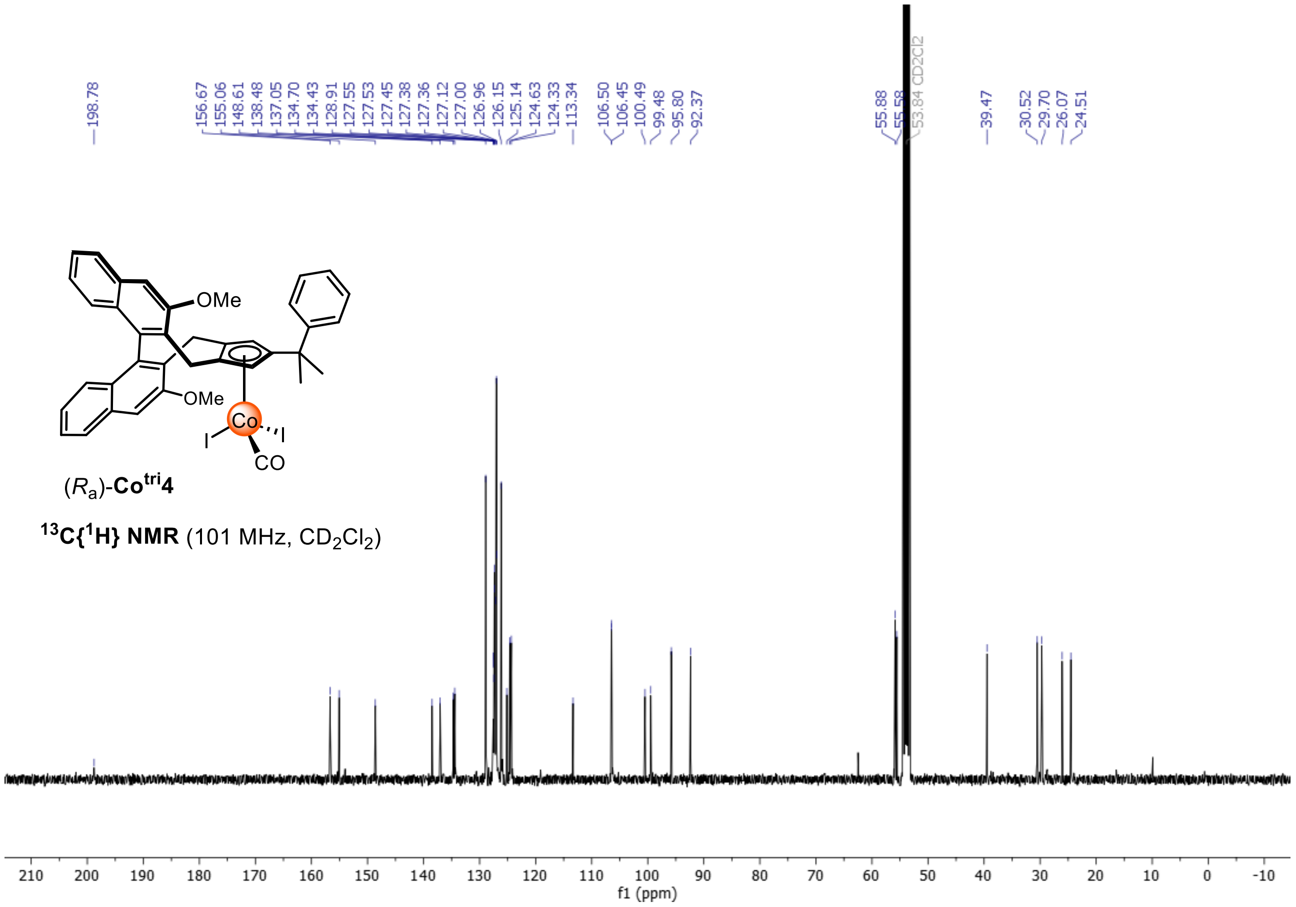
198.78
156.67
155.06
148.61
138.48
137.05
134.70
134.43
128.91
127.55
127.53
127.45
127.38
127.36
127.12
127.00
126.96
126.15
125.14
124.63
124.33
113.34
106.50
106.45
100.49
99.48
95.80
92.37
55.88
55.58
53.84 CD2Cl2
39.47
30.52
29.70
26.07
24.51
OMe
OMe
Co
I
I
CO
(R_a)-**Co**[tri]**4**
^{13}C{^{1}H} NMR (101 MHz, CD_2Cl_2)
210 200 190 180 170 160 150 140 130 120 110 100 90 80 70 60 50 40 30 20 10 0 -10
f1 (ppm)

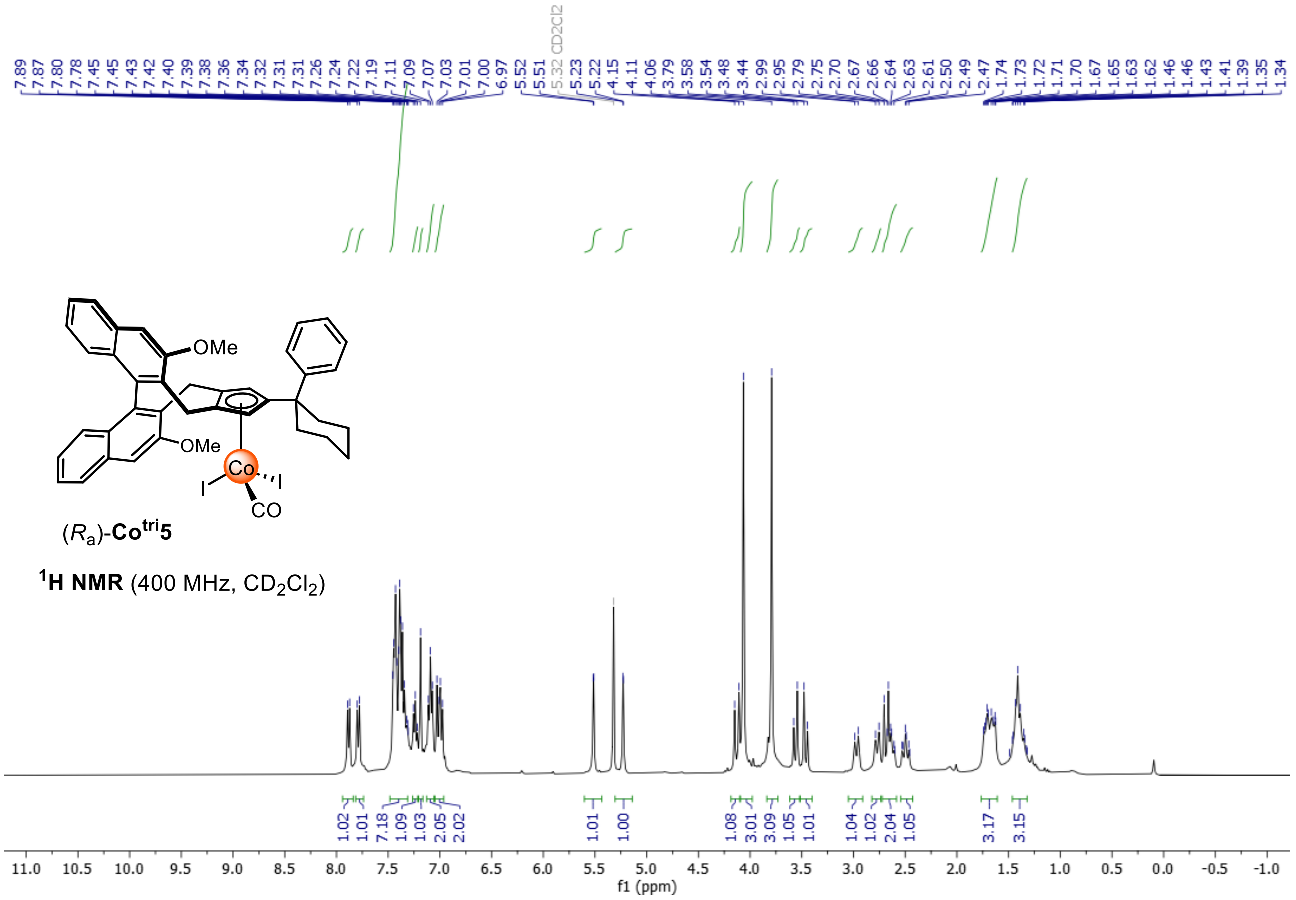

(R_a)-**Co^{tri}5**

^{1}H NMR (400 MHz, CD_2Cl_2)

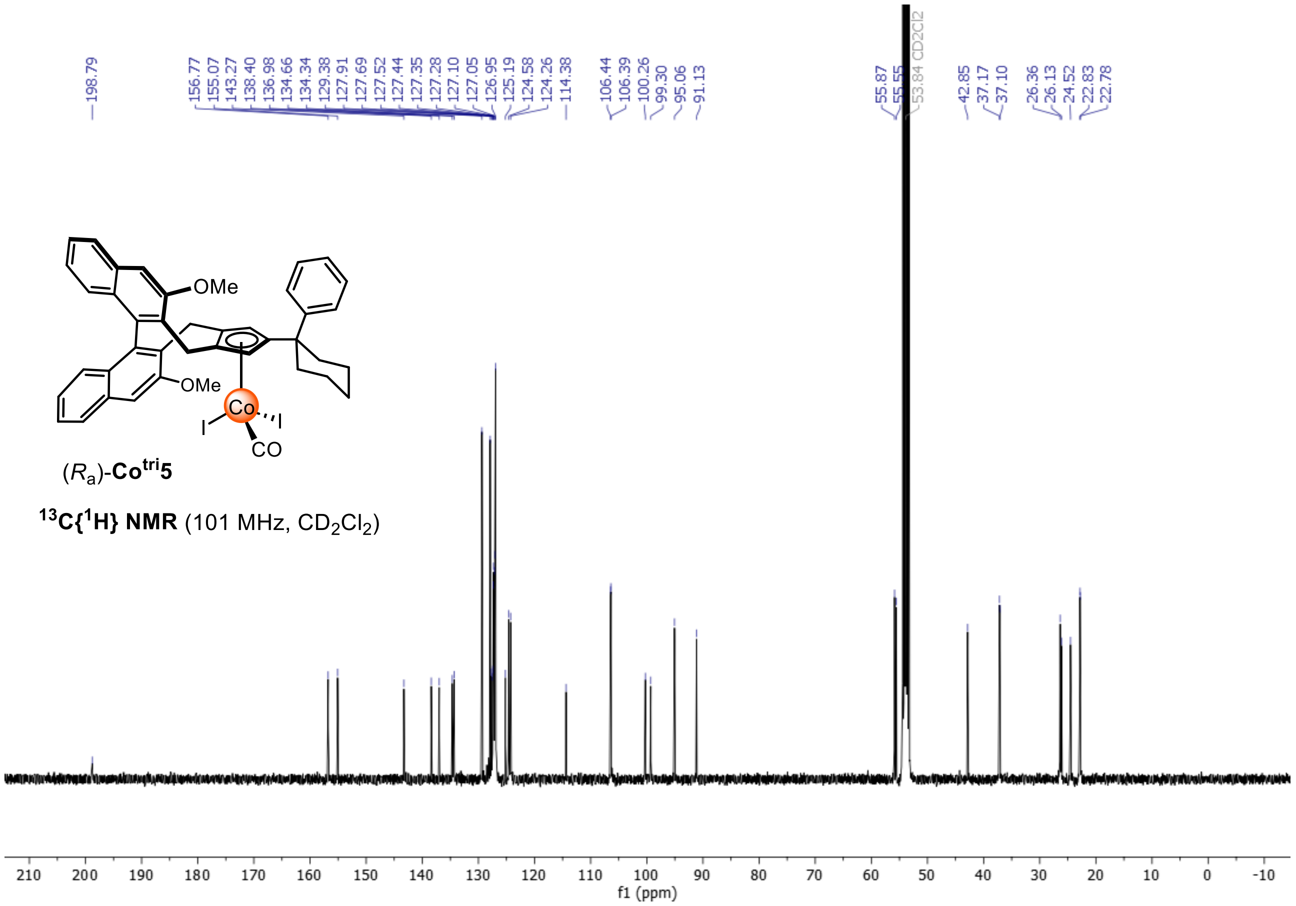

(R_a)-**Co^{tri}5**

^{13}C{^{1}H} NMR (101 MHz, CD_2Cl_2)

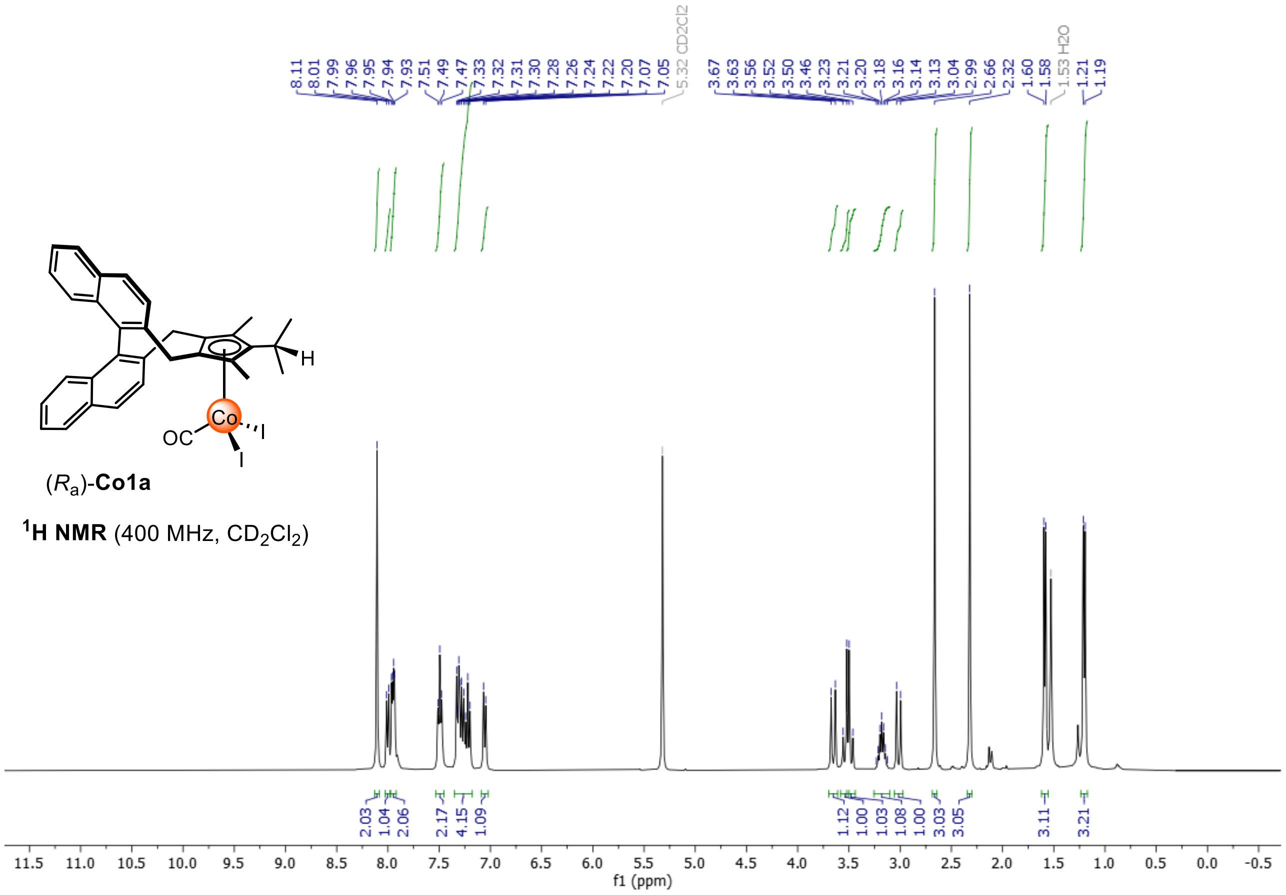

(R_a)-**Co1a**

^{1}H NMR (400 MHz, CD_2Cl_2)

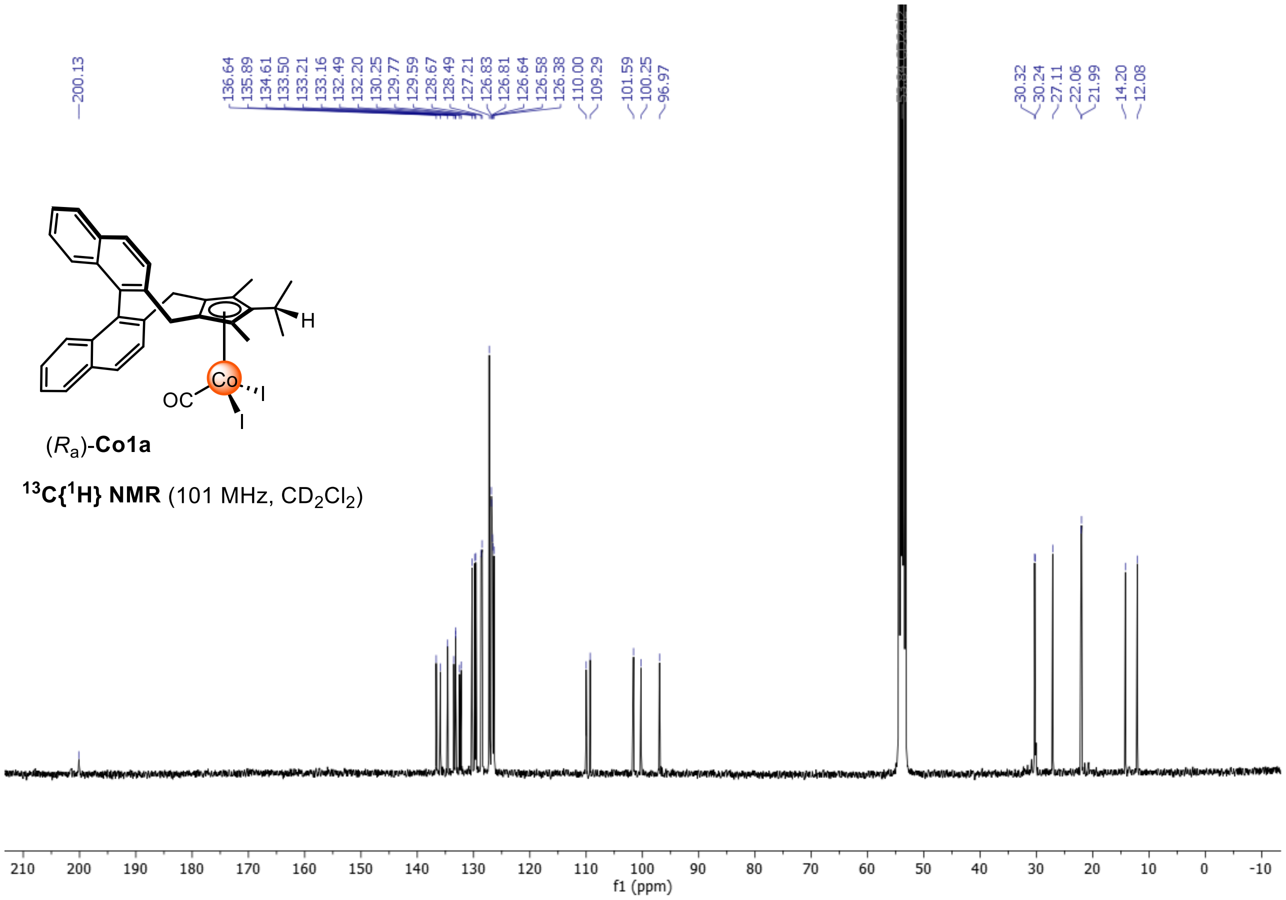

(R_a)-**Co1a**

^{13}C{^{1}H} NMR (101 MHz, CD_2Cl_2)

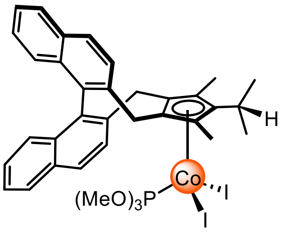

(*R*$_a$)-**Co1b**

^{1}H NMR (400 MHz, CD_2Cl_2)

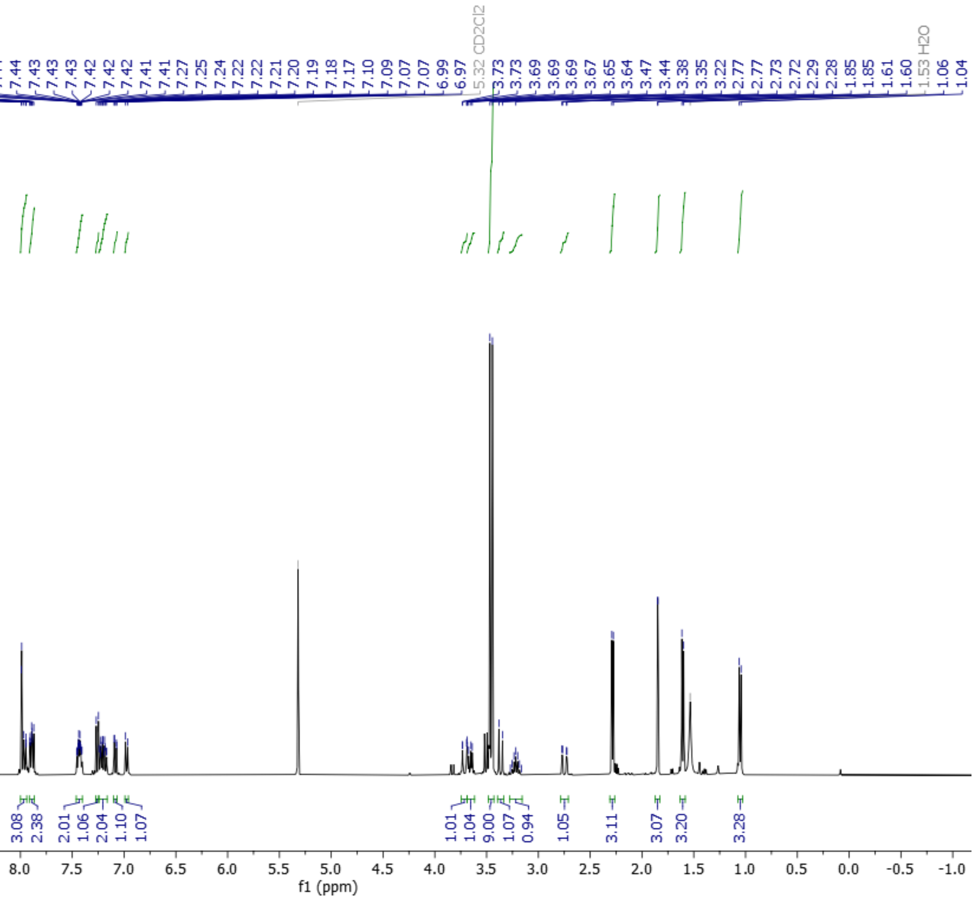

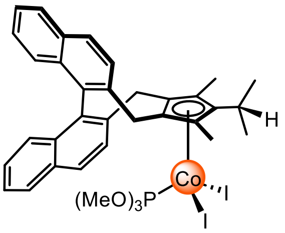

(*R*$_a$)-**Co1b**

$^{13}C\{^1H\}$ NMR (101 MHz, CD_2Cl_2)

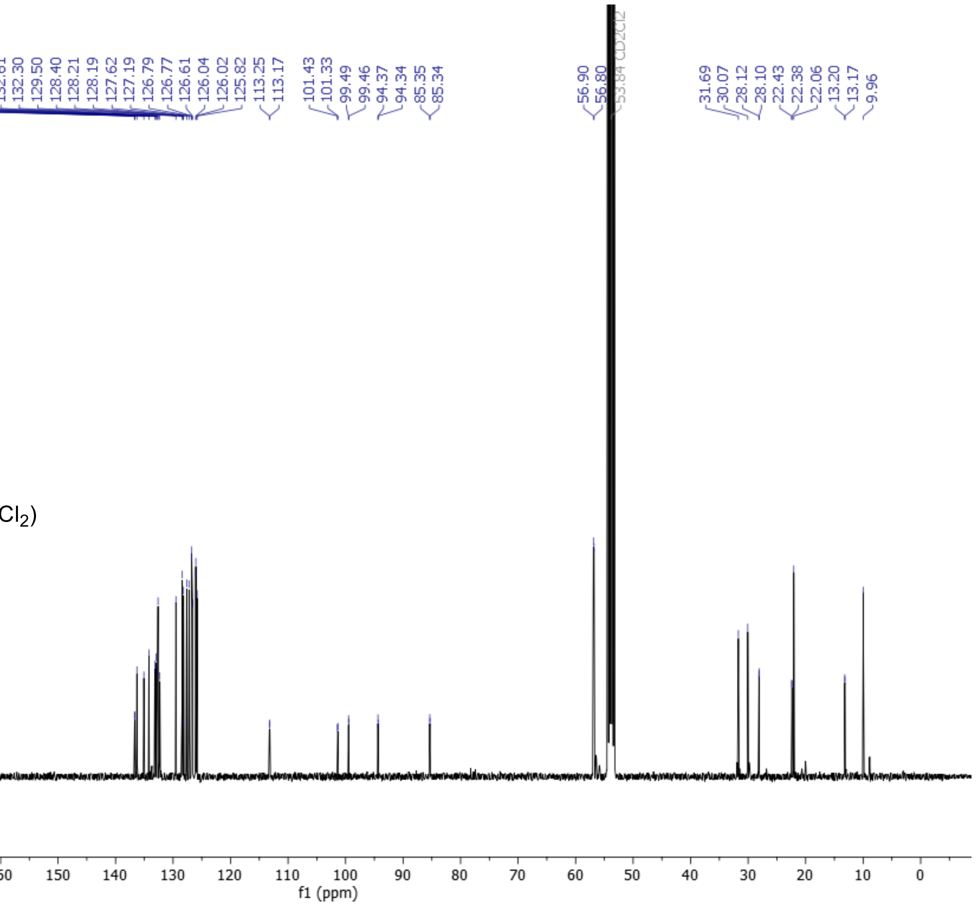

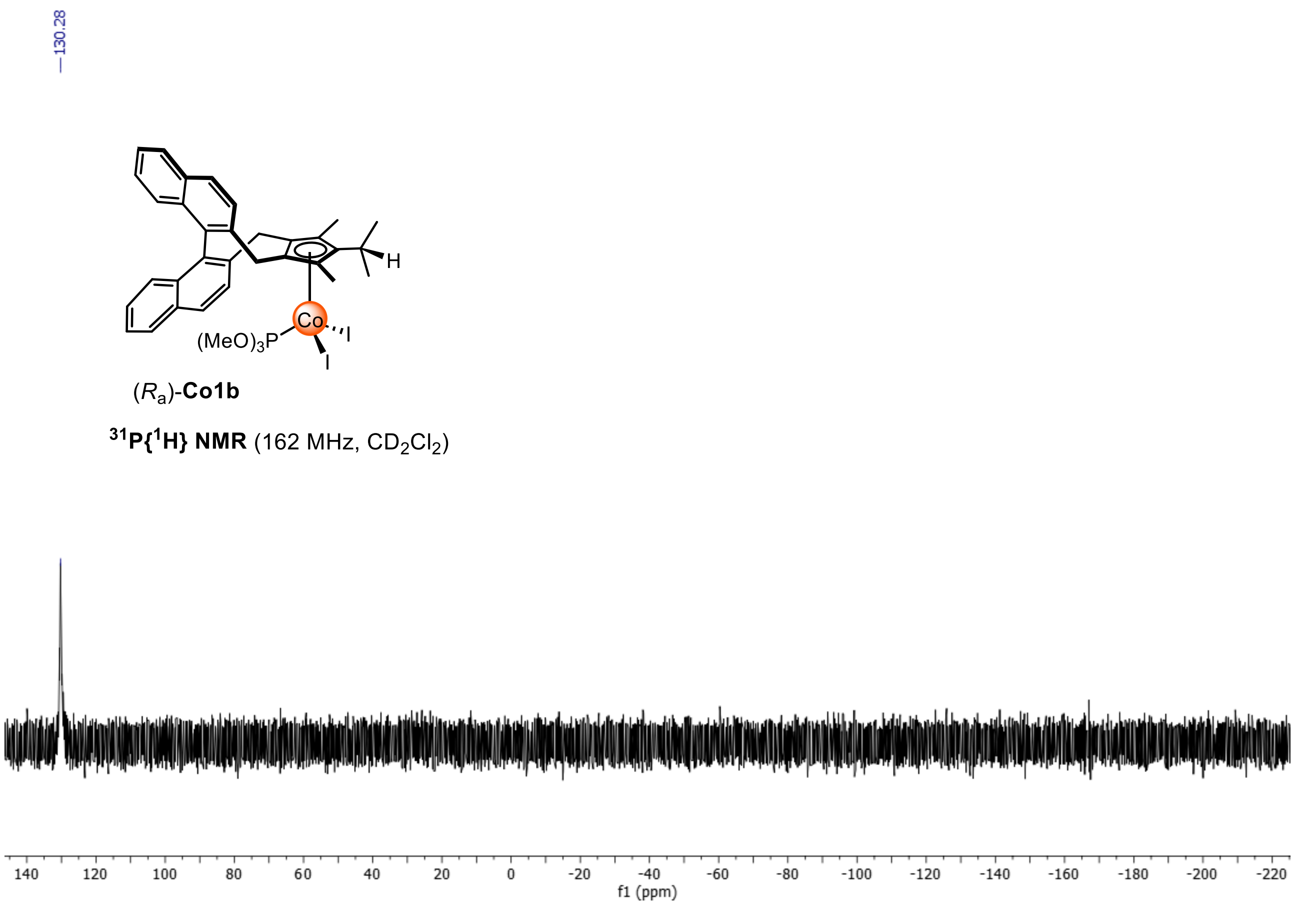
130.28
Co
(MeO)$_3$P
I
I
H
(R_a)-**Co1b**
^{31}P{^{1}H} NMR (162 MHz, CD_2Cl_2)
140 120 100 80 60 40 20 0 -20 -40 -60 -80 -100 -120 -140 -160 -180 -200 -220
f1 (ppm)

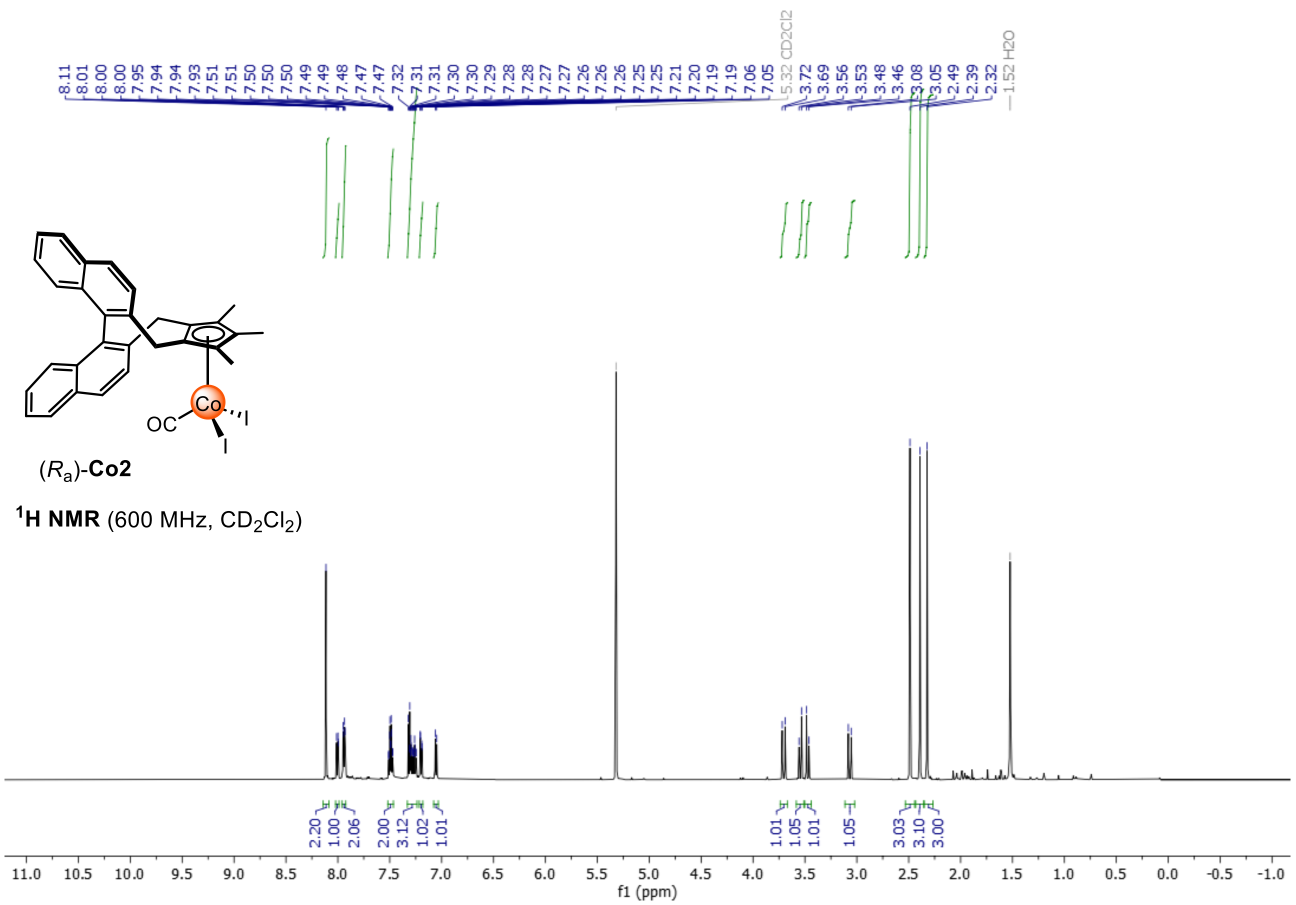

(R_a)-**Co2**

^{1}H NMR (600 MHz, CD_2Cl_2)

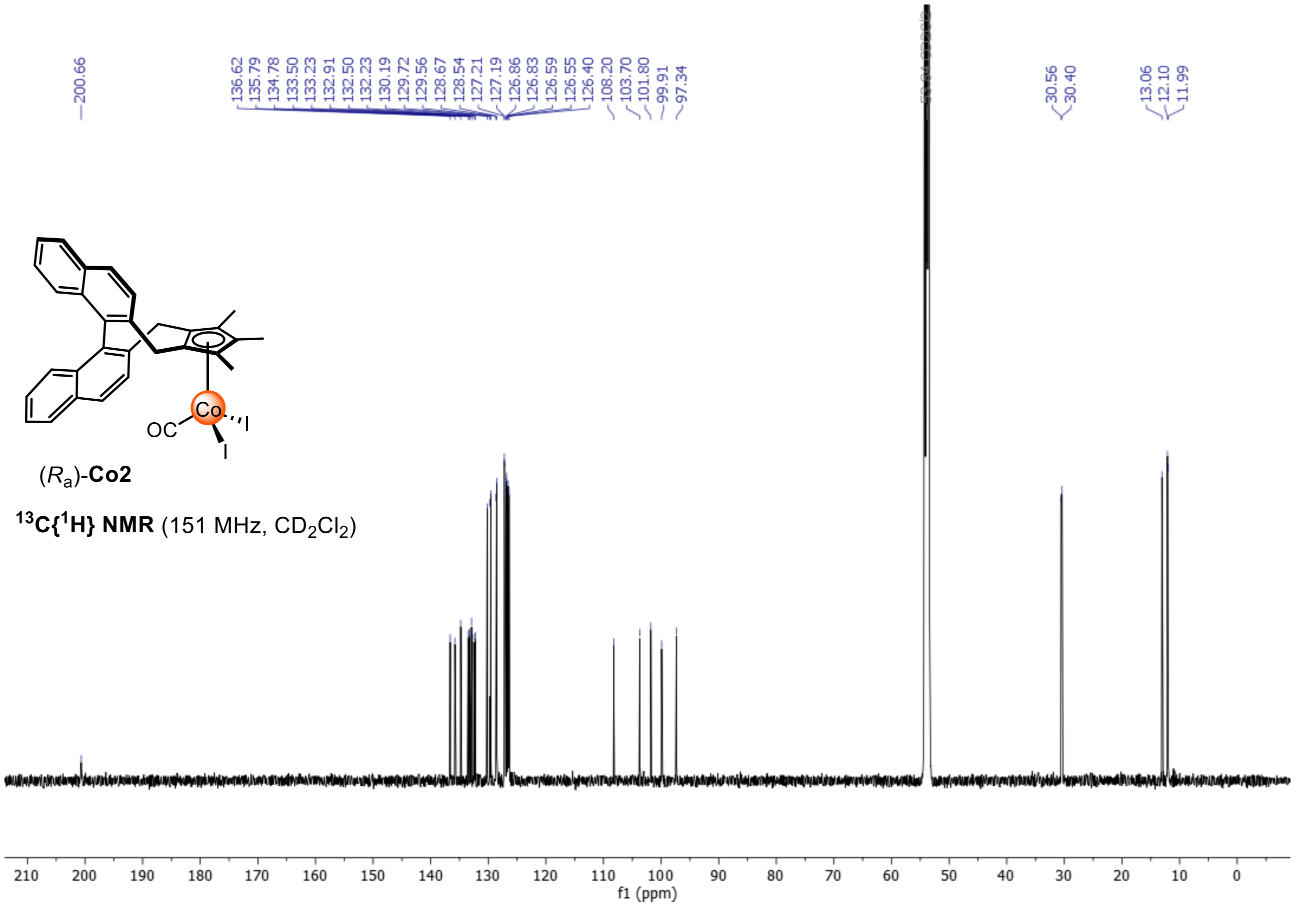

(R_a)-**Co2**

^{13}C{^{1}H} NMR (151 MHz, CD_2Cl_2)

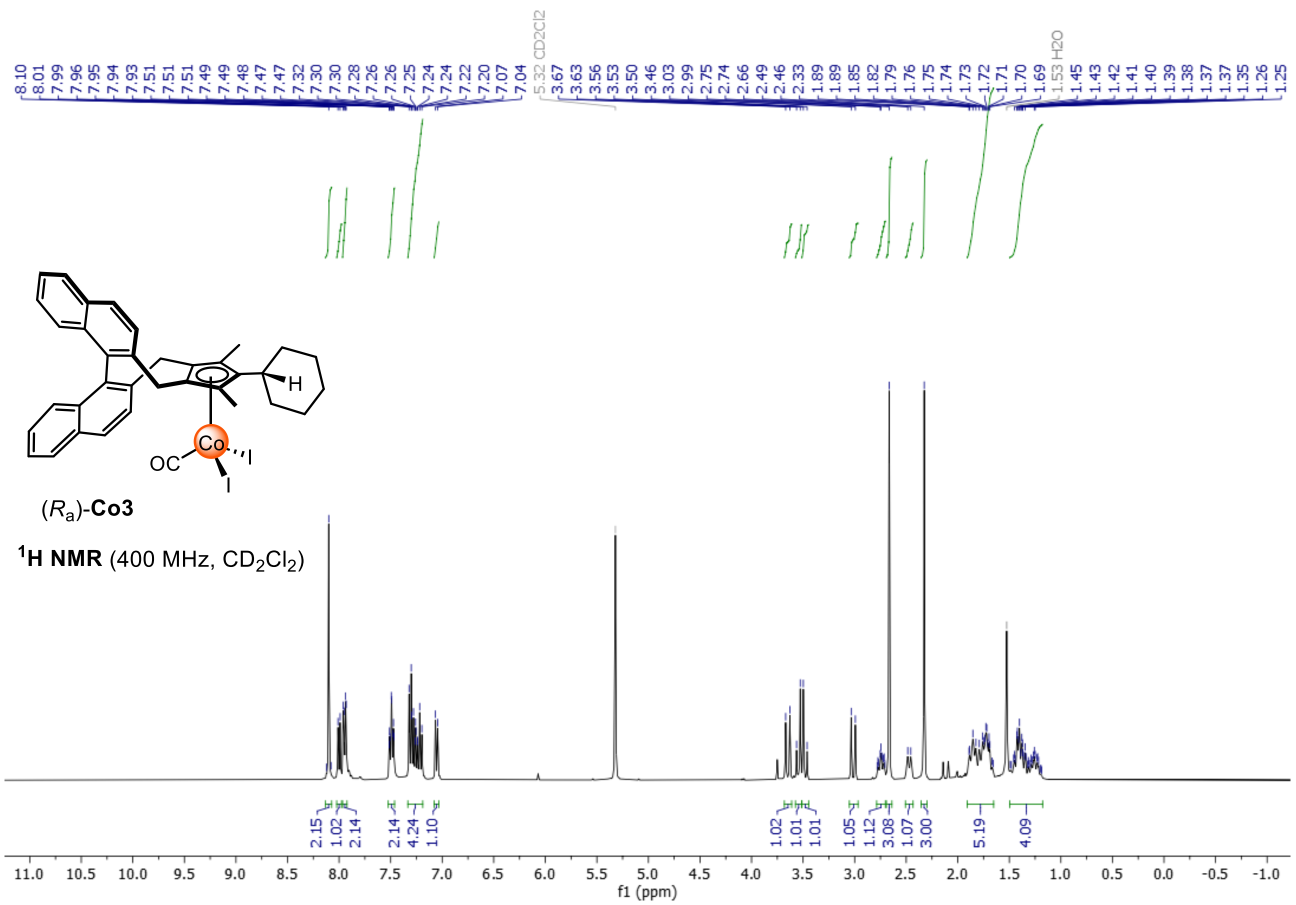
8.10
8.01
7.99
7.96
7.95
7.94
7.93
7.51
7.51
7.51
7.49
7.49
7.48
7.47
7.47
7.32
7.30
7.30
7.28
7.26
7.26
7.25
7.24
7.24
7.22
7.20
7.07
7.04
5.32 CD2Cl2
3.67
3.63
3.56
3.53
3.50
3.46
3.03
2.99
2.75
2.74
2.66
2.49
2.46
2.33
1.89
1.89
1.85
1.82
1.79
1.76
1.75
1.74
1.73
1.72
1.71
1.70
1.69
1.53 H2O
1.45
1.43
1.42
1.41
1.40
1.39
1.38
1.37
1.37
1.35
1.26
1.25
H
Co
OC
I
I
(R_a)-**Co3**
^{1}H NMR (400 MHz, CD_2Cl_2)
2.15
1.02
2.14
2.14
4.24
1.10
1.02
1.01
1.01
1.05
1.12
3.08
1.07
3.00
5.19
4.09
11.0 10.5 10.0 9.5 9.0 8.5 8.0 7.5 7.0 6.5 6.0 5.5 5.0 4.5 4.0 3.5 3.0 2.5 2.0 1.5 1.0 0.5 0.0 -0.5 -1.0
f1 (ppm)

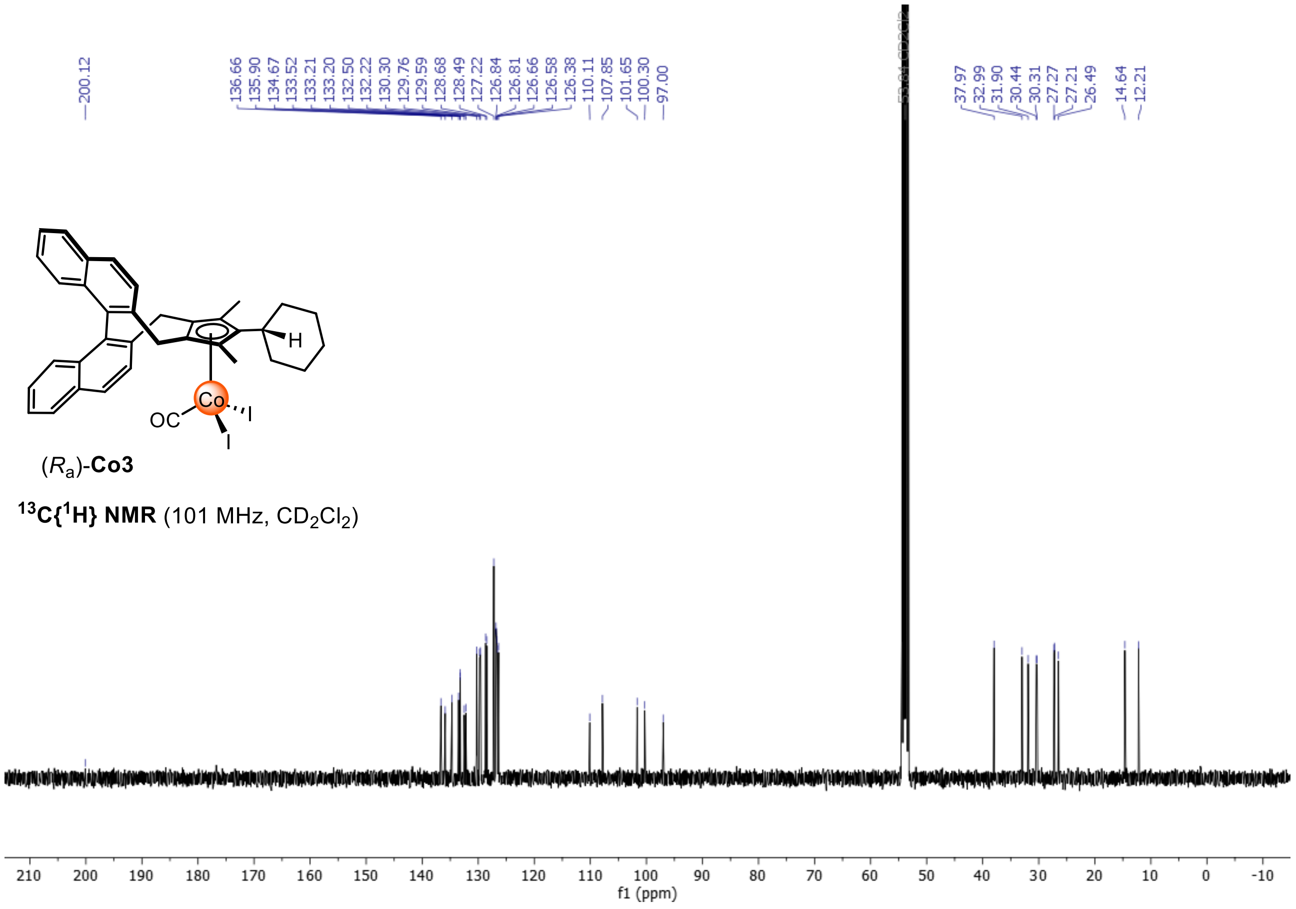
200.12
136.66
135.90
134.67
133.52
133.21
133.20
132.50
132.22
130.30
129.76
129.59
128.68
128.49
127.22
126.84
126.81
126.66
126.58
126.38
110.11
107.85
101.65
100.30
97.00
37.97
32.99
31.90
30.44
30.31
27.27
27.21
26.49
14.64
12.21
H
Co
OC
I
I
(R_a)-**Co3**
^{13}C{^{1}H} NMR (101 MHz, CD_2Cl_2)
210 200 190 180 170 160 150 140 130 120 110 100 90 80 70 60 50 40 30 20 10 0 -10
f1 (ppm)

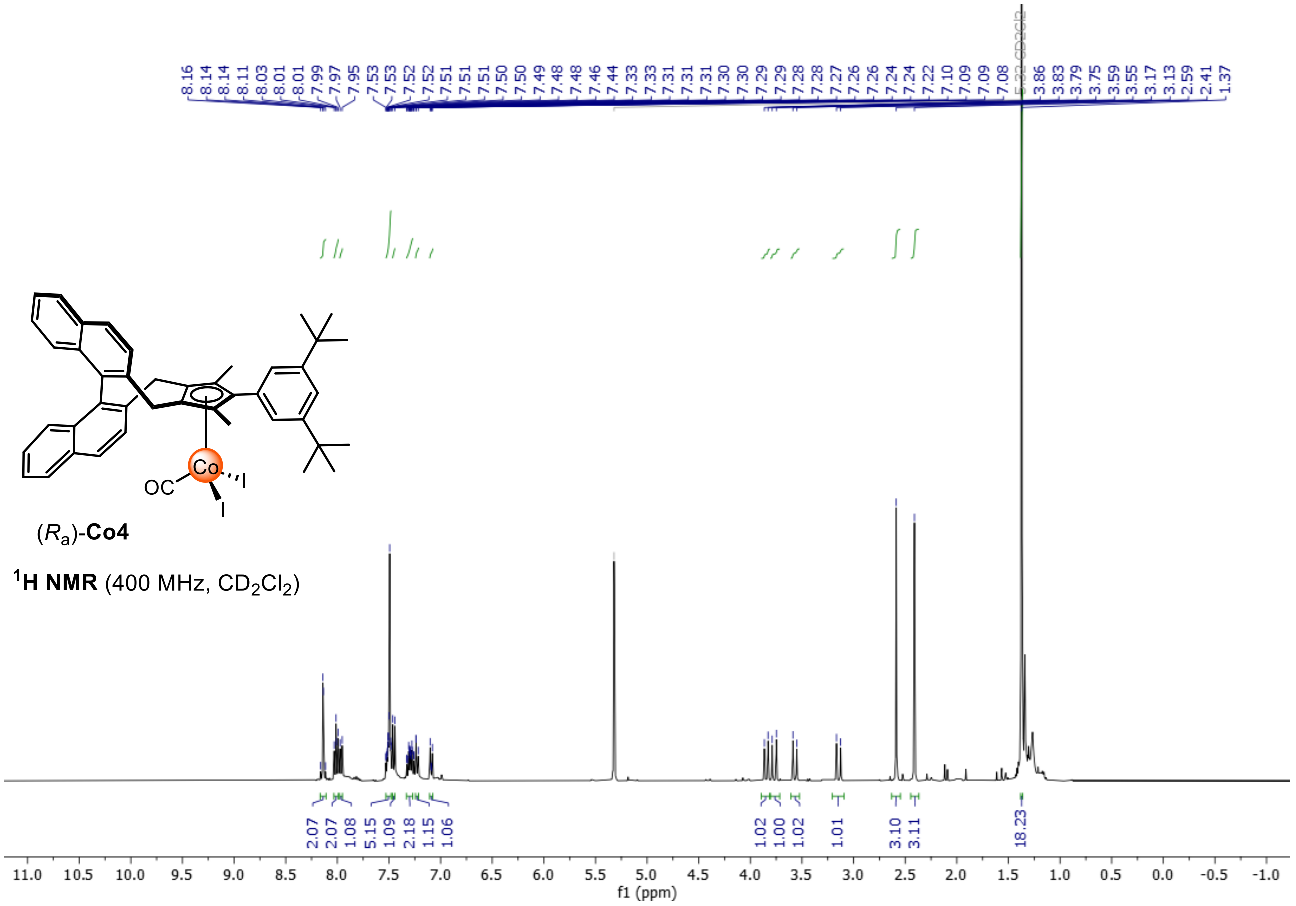
8.16 8.14 8.14 8.11 8.03 8.01 8.01 7.99 7.97 7.95 7.53 7.53 7.52 7.52 7.51 7.51 7.50 7.50 7.49 7.48 7.48 7.46 7.44 7.33 7.33 7.31 7.31 7.31 7.30 7.30 7.29 7.29 7.28 7.28 7.27 7.26 7.24 7.24 7.22 7.10 7.09 7.09 7.08 5.32 CD2Cl2 3.86 3.83 3.79 3.75 3.59 3.55 3.17 3.13 2.59 2.41 1.37
Co
OC
I
I
(Ra)-Co4
1H NMR (400 MHz, CD2Cl2)
2.07 2.07 1.08 5.15 1.09 2.18 1.15 1.06 1.02 1.00 1.02 1.01 3.10 3.11 18.23
f1 (ppm)

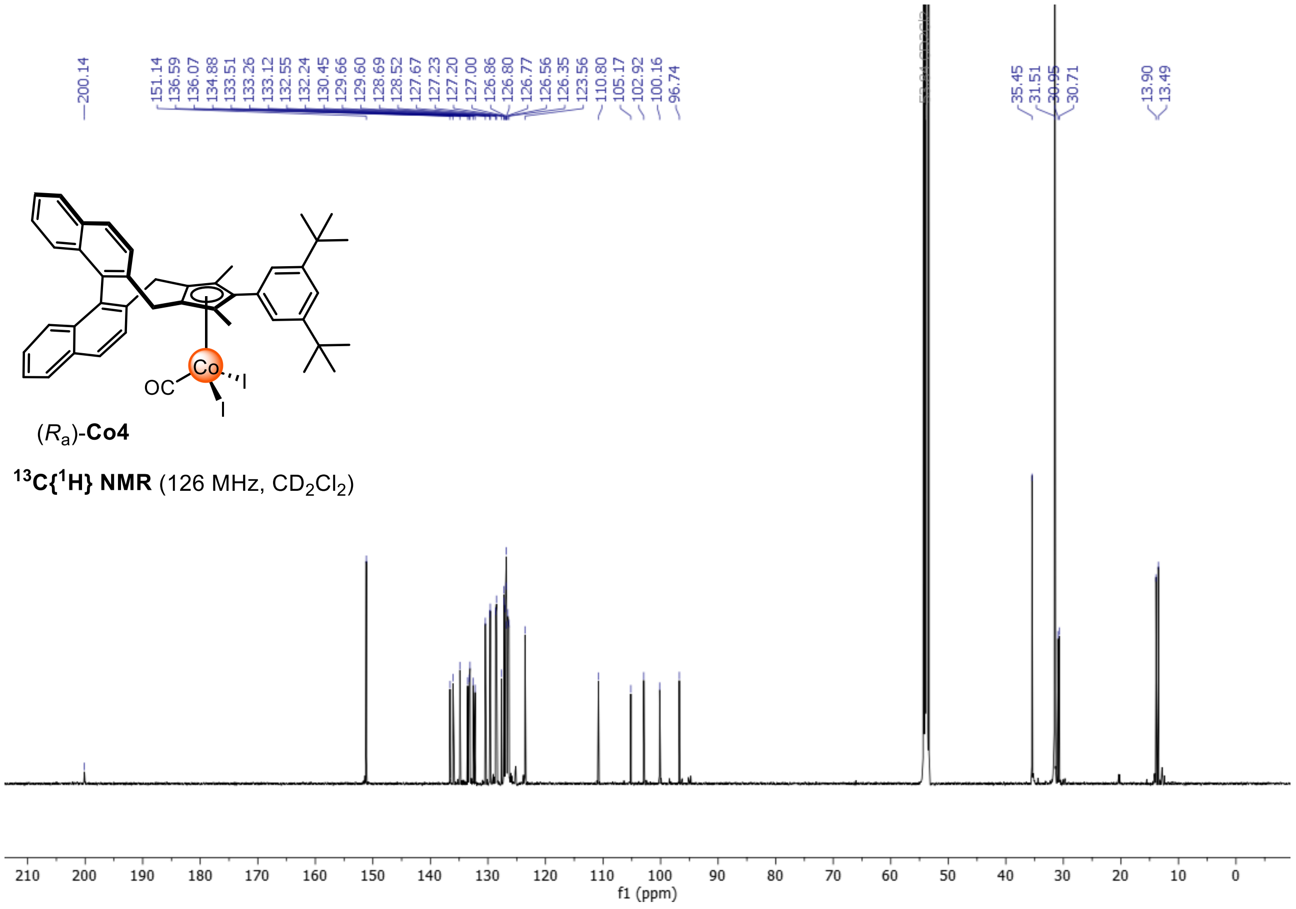
200.14
151.14 136.59 136.07 134.88 133.51 133.26 133.12 132.55 132.24 130.45 129.66 129.60 128.69 128.52 127.67 127.23 127.20 127.00 126.86 126.80 126.77 126.56 126.35 123.56 110.80 105.17 102.92 100.16 96.74
53.84 CD2Cl2
35.45 31.51 30.95 30.71
13.90 13.49
Co
OC
I
I
(Ra)-Co4
13C{1H} NMR (126 MHz, CD2Cl2)
f1 (ppm)

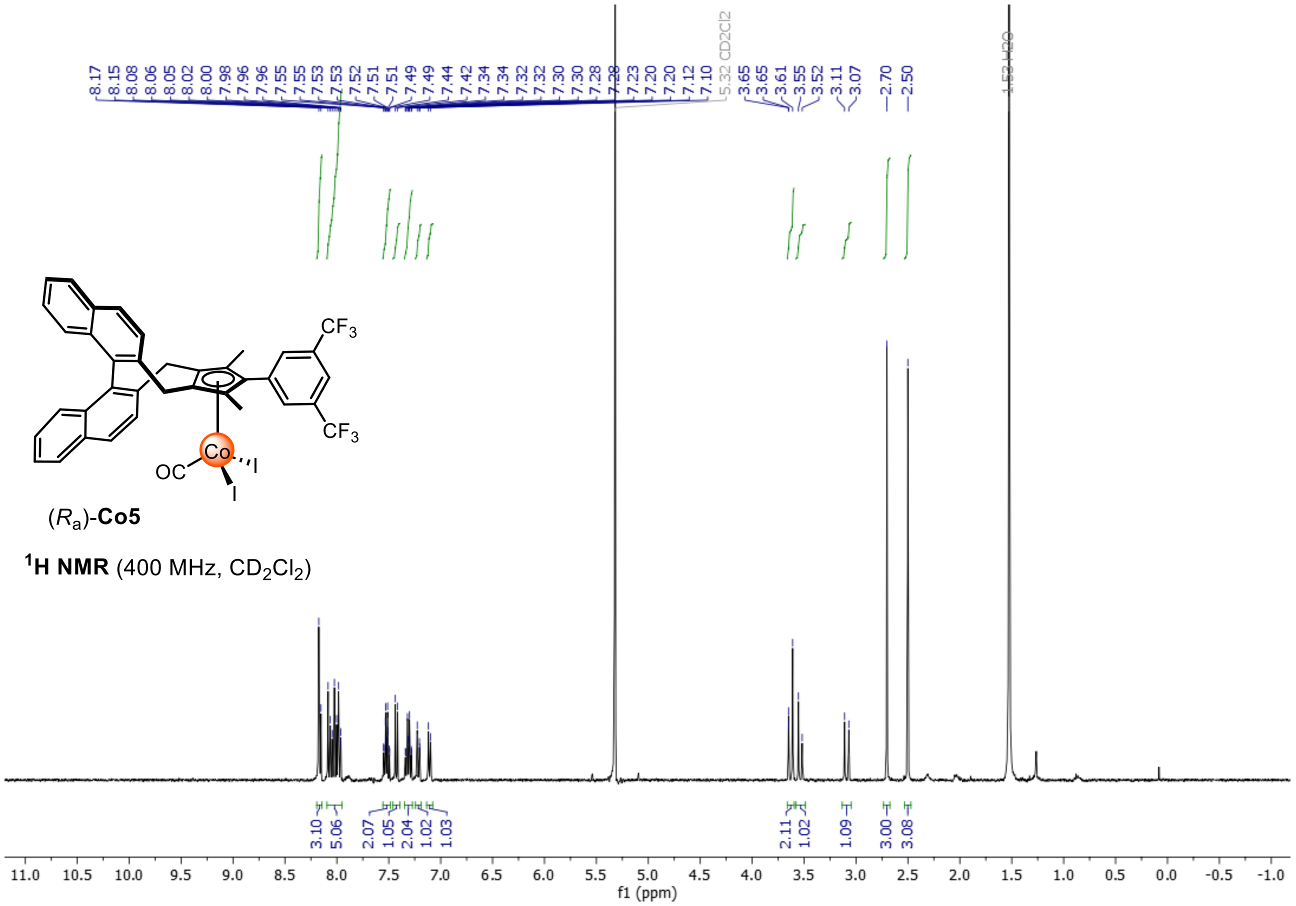
8.17 8.15 8.08 8.06 8.05 8.02 8.00 7.98 7.96 7.96 7.55 7.55 7.53 7.53 7.52 7.51 7.51 7.49 7.49 7.44 7.42 7.34 7.34 7.32 7.32 7.30 7.30 7.28 7.28 7.23 7.20 7.20 7.12 7.10
5.32 CD2Cl2
3.65 3.65 3.61 3.55 3.52 3.11 3.07 2.70 2.50
1.53 H2O
CF3
CF3
Co
OC
I
I
(R_a)-**Co5**
^{1}H NMR (400 MHz, CD_2Cl_2)
3.10 5.06 2.07 1.05 2.04 1.02 1.03 2.11 1.02 1.09 3.00 3.08
11.0 10.5 10.0 9.5 9.0 8.5 8.0 7.5 7.0 6.5 6.0 5.5 5.0 4.5 4.0 3.5 3.0 2.5 2.0 1.5 1.0 0.5 0.0 -0.5 -1.0
f1 (ppm)

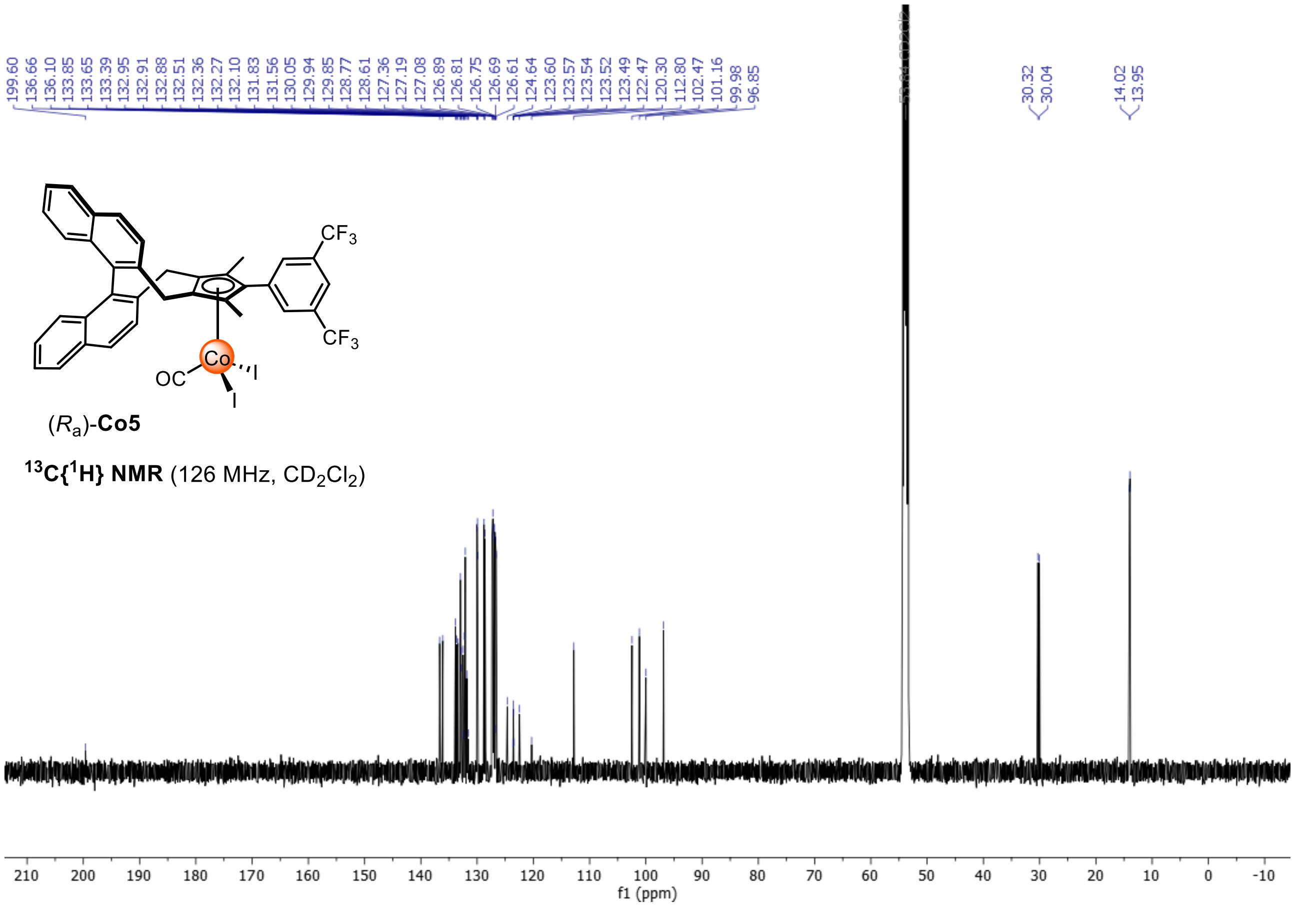
199.60 136.66 136.10 133.85 133.65 133.39 132.95 132.91 132.88 132.51 132.36 132.27 132.10 131.83 131.56 130.05 129.94 129.85 128.77 128.61 127.36 127.19 127.08 126.89 126.81 126.75 126.69 126.61 124.64 123.60 123.57 123.54 123.52 123.49 122.47 120.30 112.80 102.47 101.16 99.98 96.85
30.32 30.04
14.02 13.95
CF3
CF3
Co
OC
I
I
(R_a)-**Co5**
^{13}C{^{1}H} NMR (126 MHz, CD_2Cl_2)
210 200 190 180 170 160 150 140 130 120 110 100 90 80 70 60 50 40 30 20 10 0 -10
f1 (ppm)

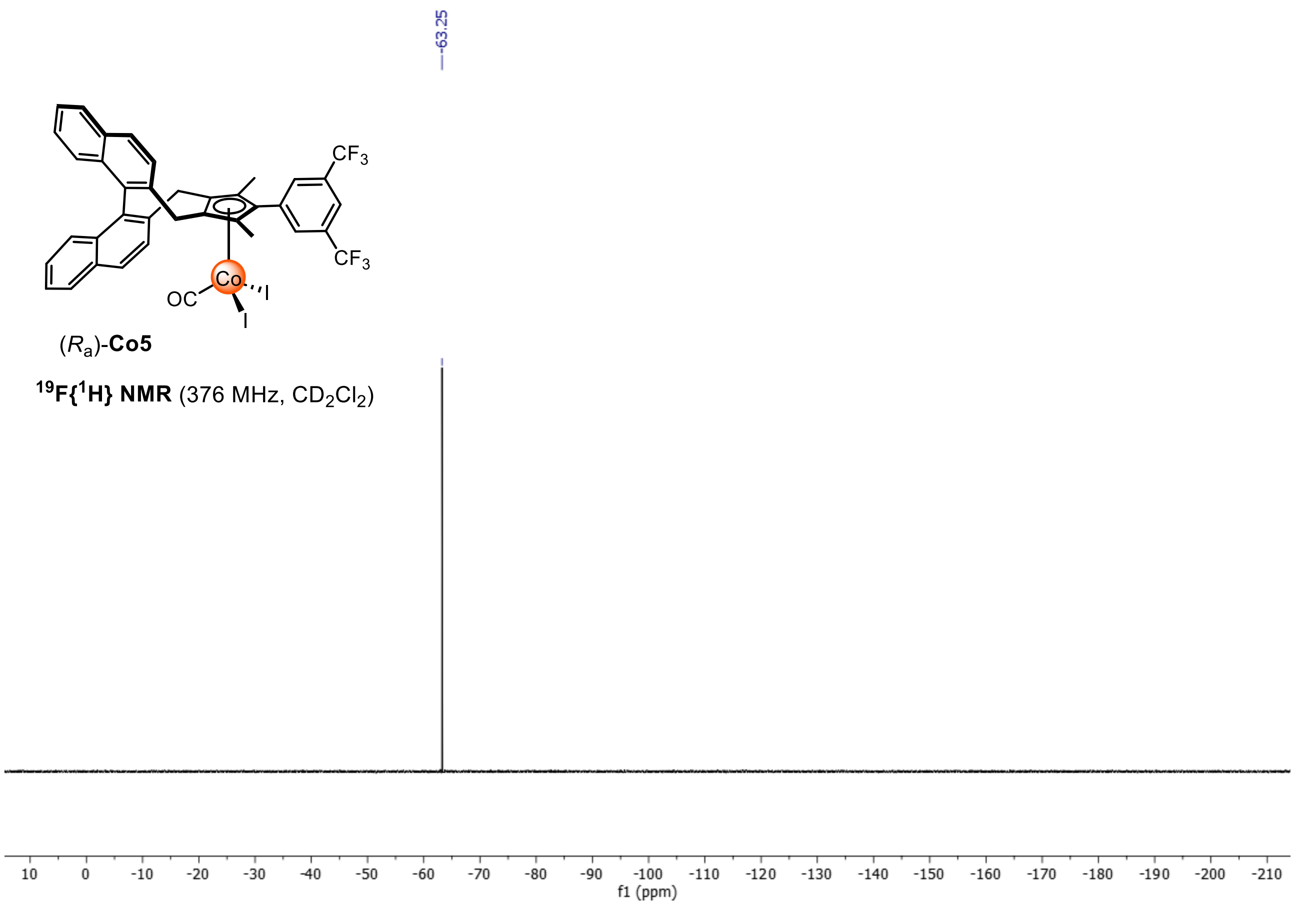
-63.25
CF3
CF3
Co
OC
I
I
(Ra)-Co5
19F{1H} NMR (376 MHz, CD2Cl2)
10 0 -10 -20 -30 -40 -50 -60 -70 -80 -90 -100 -110 -120 -130 -140 -150 -160 -170 -180 -190 -200 -210
f1 (ppm)

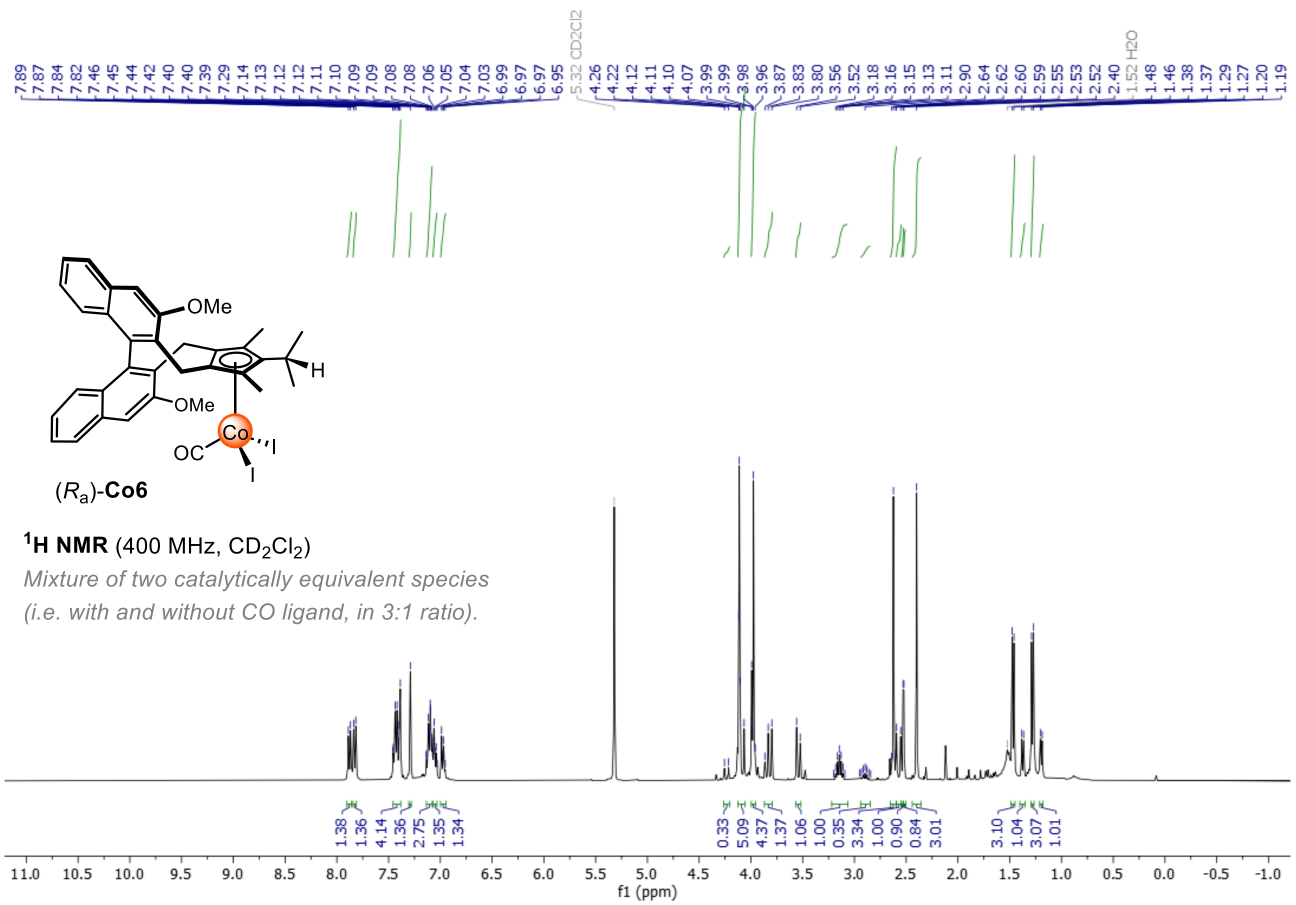
7.89 7.87 7.84 7.82 7.46 7.45 7.44 7.42 7.40 7.40 7.39 7.29 7.14 7.13 7.12 7.12 7.11 7.10 7.09 7.09 7.08 7.08 7.06 7.05 7.04 7.03 6.99 6.97 6.97 6.95
5.32 CD2Cl2
4.26 4.22 4.12 4.11 4.10 4.07 3.99 3.99 3.98 3.96 3.87 3.83 3.80 3.56 3.52 3.18 3.16 3.15 3.13 3.11 2.90 2.64 2.62 2.60 2.59 2.55 2.53 2.52 2.40
1.52 H2O
1.48 1.46 1.38 1.37 1.29 1.27 1.20 1.19
OMe
OMe
H
Co
OC
I
I
(R_a)-**Co6**
^{1}**H NMR** (400 MHz, CD_2Cl_2)
Mixture of two catalytically equivalent species
(i.e. with and without CO ligand, in 3:1 ratio).
1.38 1.36 4.14 1.36 2.75 1.35 1.34
0.33 5.09 4.37 1.37 1.06 1.00 0.35 3.34 1.00 0.90 0.84 3.01
3.10 1.04 3.07 1.01
11.0 10.5 10.0 9.5 9.0 8.5 8.0 7.5 7.0 6.5 6.0 5.5 5.0 4.5 4.0 3.5 3.0 2.5 2.0 1.5 1.0 0.5 0.0 -0.5 -1.0
f1 (ppm)

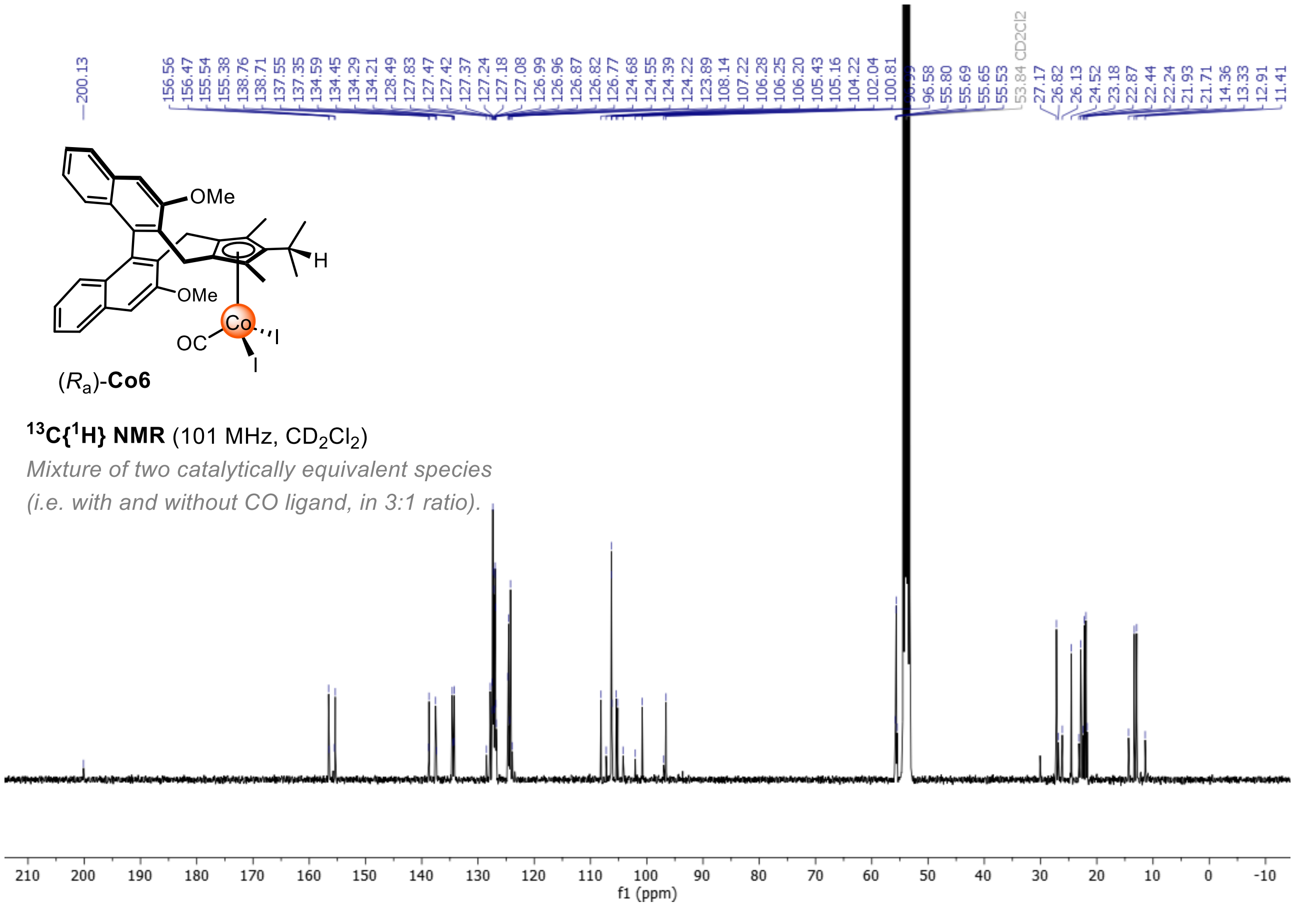
200.13
156.56 156.47 155.54 155.38 138.76 138.71 137.55 137.35 134.59 134.45 134.29 134.21 128.49 127.83 127.47 127.42 127.37 127.24 127.18 127.08 126.99 126.96 126.87 126.82 126.77 124.68 124.55 124.39 124.22 123.89 108.14 107.22 106.28 106.25 106.20 105.43 105.16 104.22 102.04 100.81
96.58 55.80 55.69 55.65 55.53
53.84 CD2Cl2
27.17 26.82 26.13 24.52 23.18 22.87 22.44 22.24 21.93 21.71 14.36 13.33 12.91 11.41
OMe
OMe
H
Co
OC
I
I
(R_a)-**Co6**
^{13}**C{^{1}H} NMR** (101 MHz, CD_2Cl_2)
Mixture of two catalytically equivalent species
(i.e. with and without CO ligand, in 3:1 ratio).
210 200 190 180 170 160 150 140 130 120 110 100 90 80 70 60 50 40 30 20 10 0 -10
f1 (ppm)

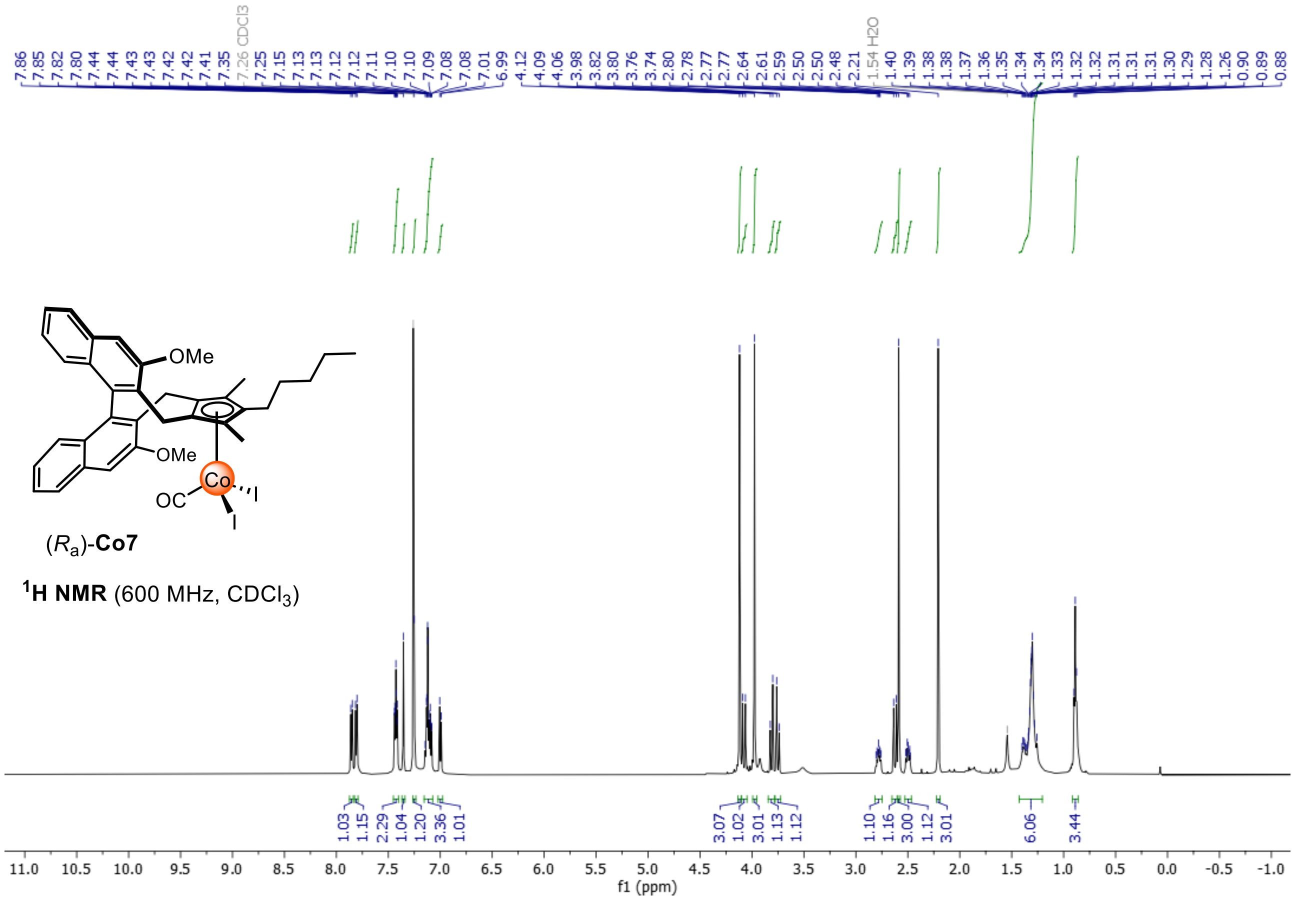
OMe
OMe
Co
OC
I
I
(R_a)-**Co7**
^{1}H NMR (600 MHz, $CDCl_3$)
f1 (ppm)

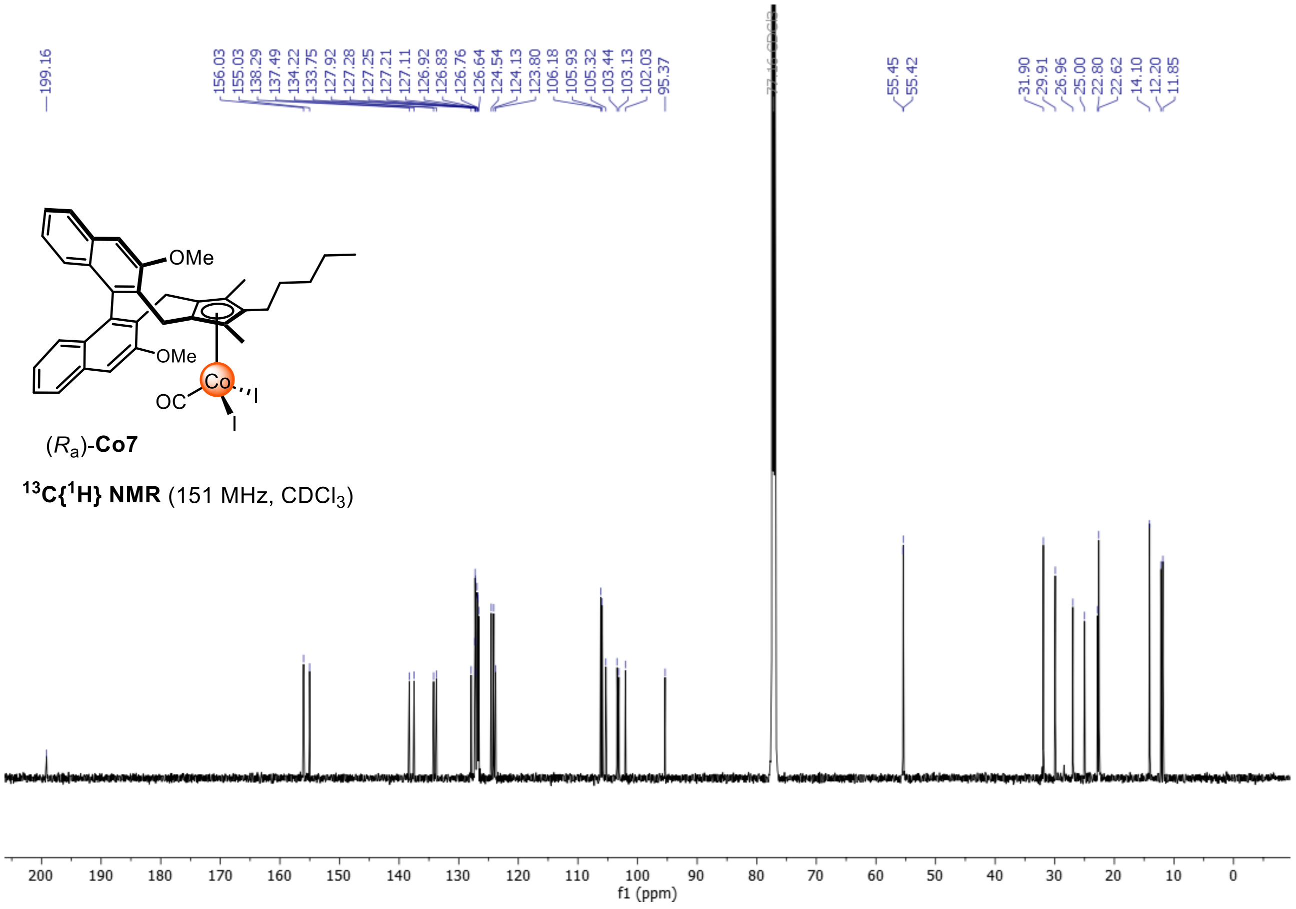
OMe
OMe
Co
OC
I
I
(R_a)-**Co7**
^{13}C{^{1}H} NMR (151 MHz, $CDCl_3$)
f1 (ppm)

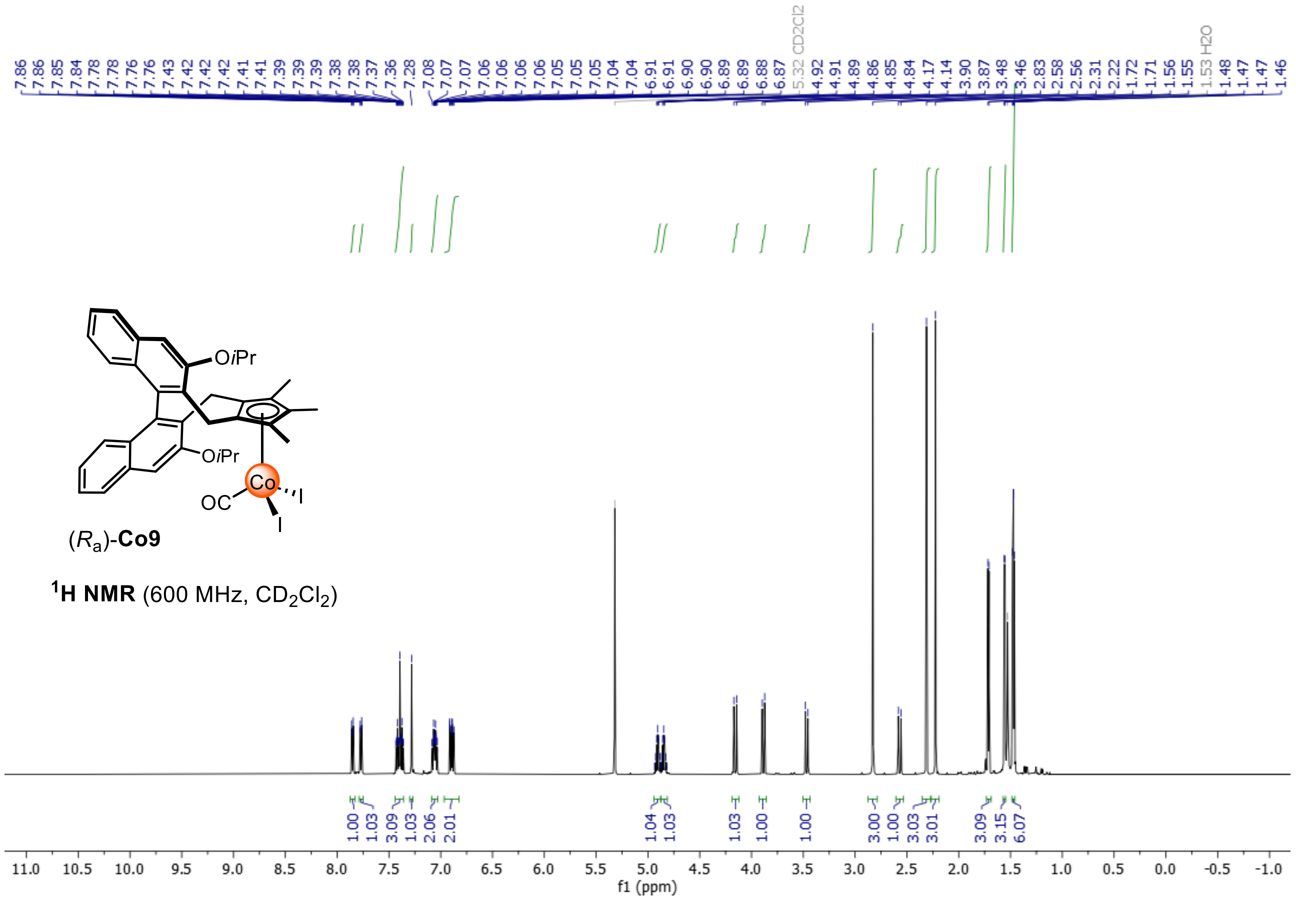

(R_a)-**Co9**

^{1}H NMR (600 MHz, CD_2Cl_2)

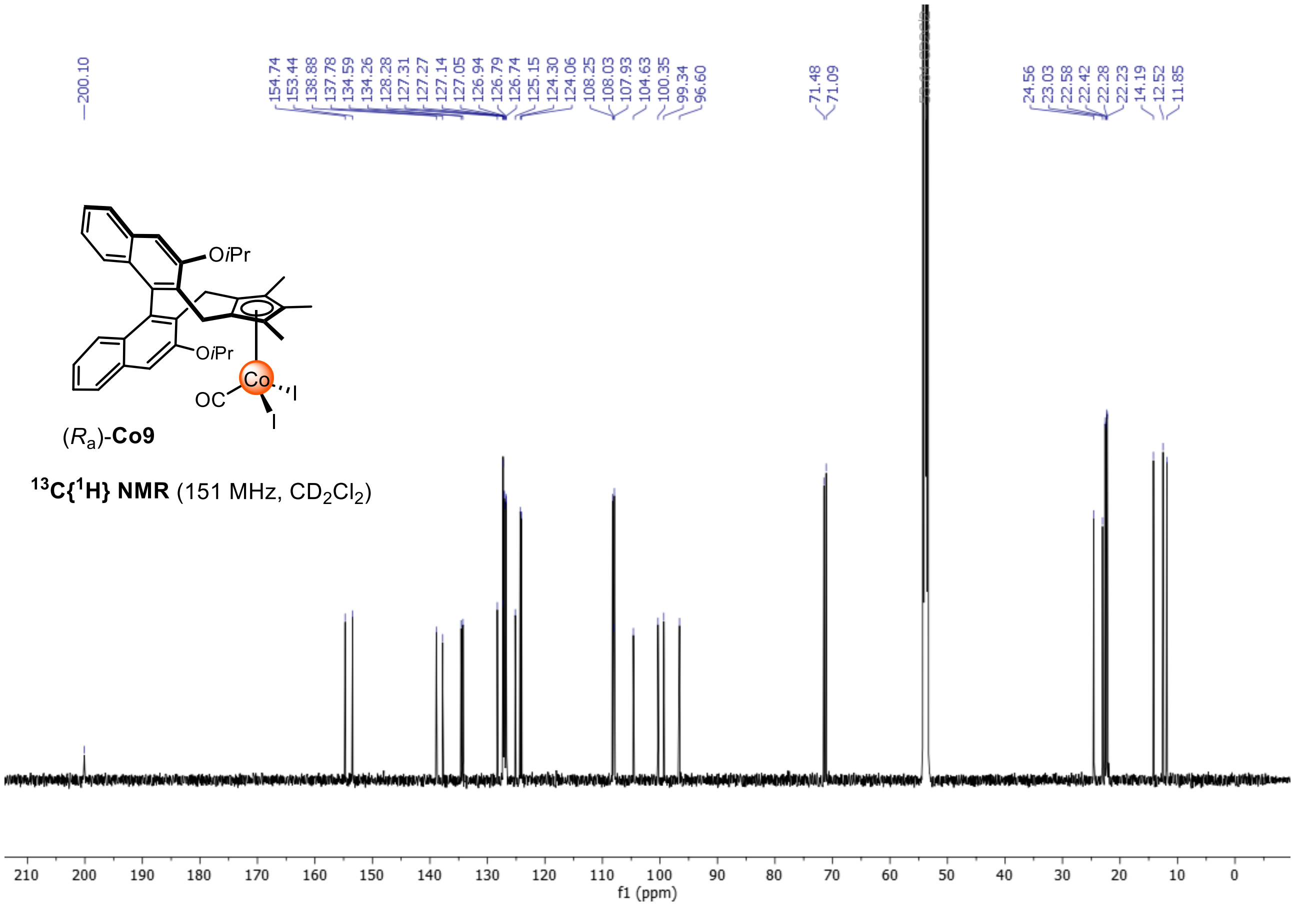

(R_a)-**Co9**

^{13}C{^{1}H} NMR (151 MHz, CD_2Cl_2)

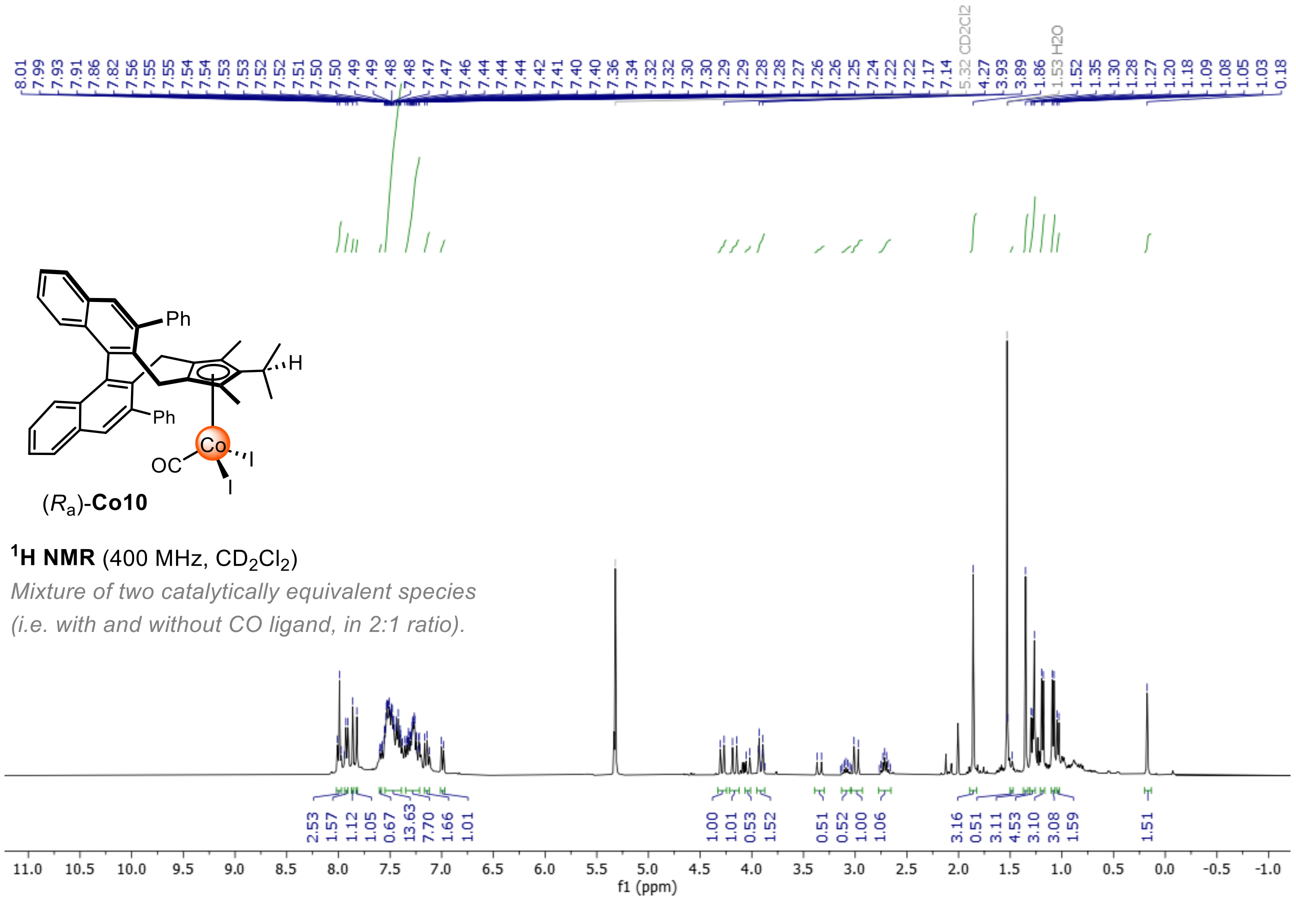
8.01 7.99 7.93 7.91 7.86 7.82 7.56 7.55 7.55 7.54 7.54 7.53 7.53 7.52 7.52 7.51 7.50 7.50 7.49 7.49 7.48 7.48 7.47 7.47 7.46 7.44 7.44 7.44 7.42 7.41 7.40 7.40 7.36 7.34 7.32 7.32 7.30 7.30 7.29 7.29 7.28 7.28 7.27 7.26 7.26 7.25 7.24 7.22 7.22 7.17 7.14
5.32 CD2Cl2
4.27 3.93 3.89 1.86
1.53 H2O
1.52 1.35 1.30 1.28 1.27 1.20 1.18 1.09 1.08 1.05 1.03 0.18
Ph
H
Ph
Co
OC
I
I
(R_a)-**Co10**
^{1}H NMR (400 MHz, CD_2Cl_2)
Mixture of two catalytically equivalent species
(i.e. with and without CO ligand, in 2:1 ratio).
2.53 1.57 1.12 1.05 0.67 13.63 7.70 1.66 1.01
1.00 1.01 0.53 1.52
0.51 0.52 1.00 1.06
3.16 0.51 3.11 4.53 3.10 3.08 1.59
1.51
11.0 10.5 10.0 9.5 9.0 8.5 8.0 7.5 7.0 6.5 6.0 5.5 5.0 4.5 4.0 3.5 3.0 2.5 2.0 1.5 1.0 0.5 0.0 -0.5 -1.0
f1 (ppm)

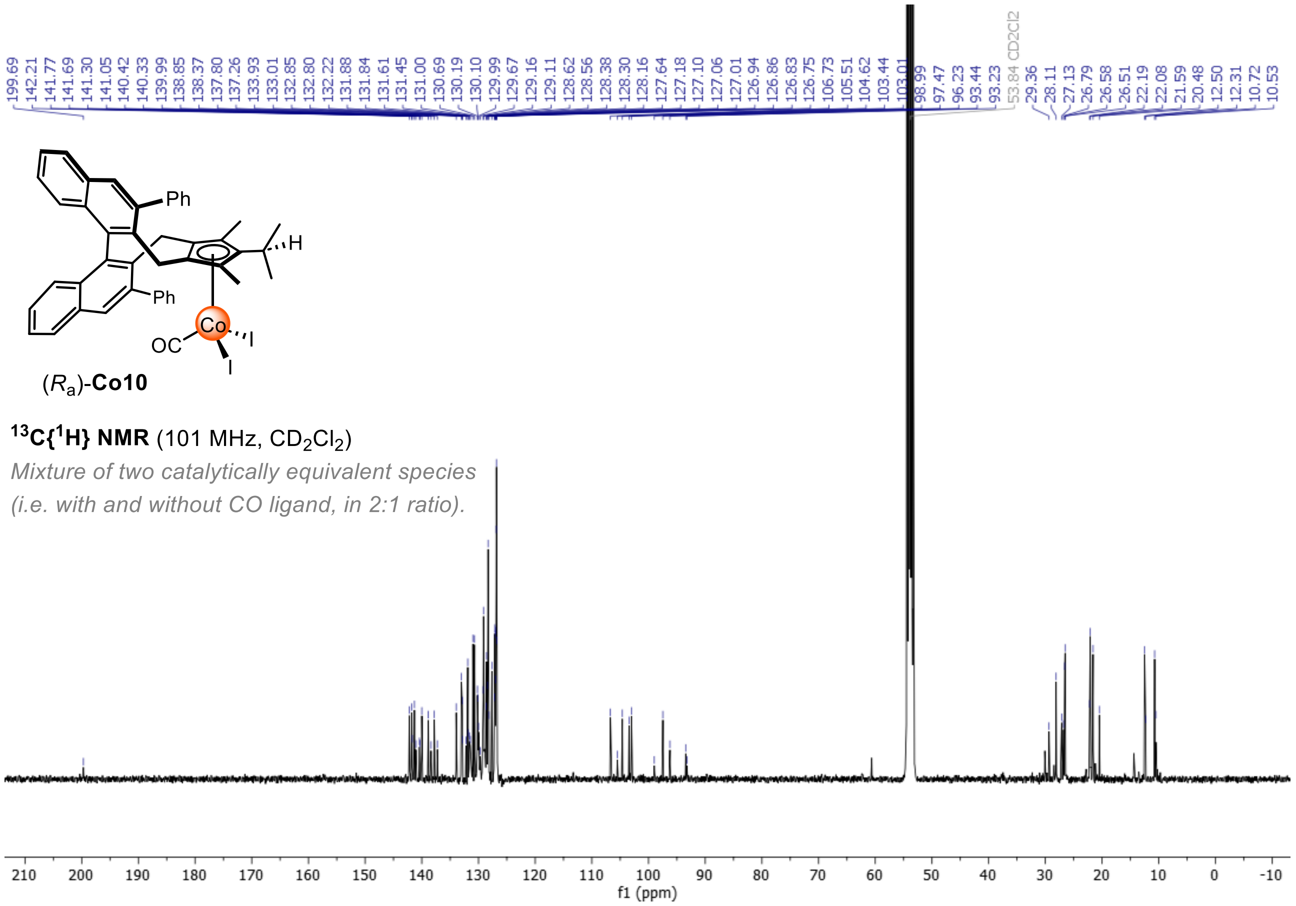
199.69 142.21 141.77 141.69 141.30 141.05 140.42 140.33 139.99 138.85 138.37 137.80 137.26 133.93 133.01 132.85 132.80 132.22 131.88 131.84 131.61 131.45 131.00 130.69 130.19 130.10 129.99 129.67 129.16 129.11 128.62 128.56 128.38 128.30 128.16 127.64 127.18 127.10 127.06 127.01 126.94 126.86 126.83 126.75 106.73 105.51 104.62 103.44 103.01 98.99 97.47 96.23 93.44 93.23
53.84 CD2Cl2
29.36 28.11 27.13 26.79 26.58 26.51 22.19 22.08 21.59 20.48 12.50 12.31 10.72 10.53
Ph
H
Ph
Co
OC
I
I
(R_a)-**Co10**
^{13}C{^{1}H} NMR (101 MHz, CD_2Cl_2)
Mixture of two catalytically equivalent species
(i.e. with and without CO ligand, in 2:1 ratio).
210 200 190 180 170 160 150 140 130 120 110 100 90 80 70 60 50 40 30 20 10 0 -10
f1 (ppm)

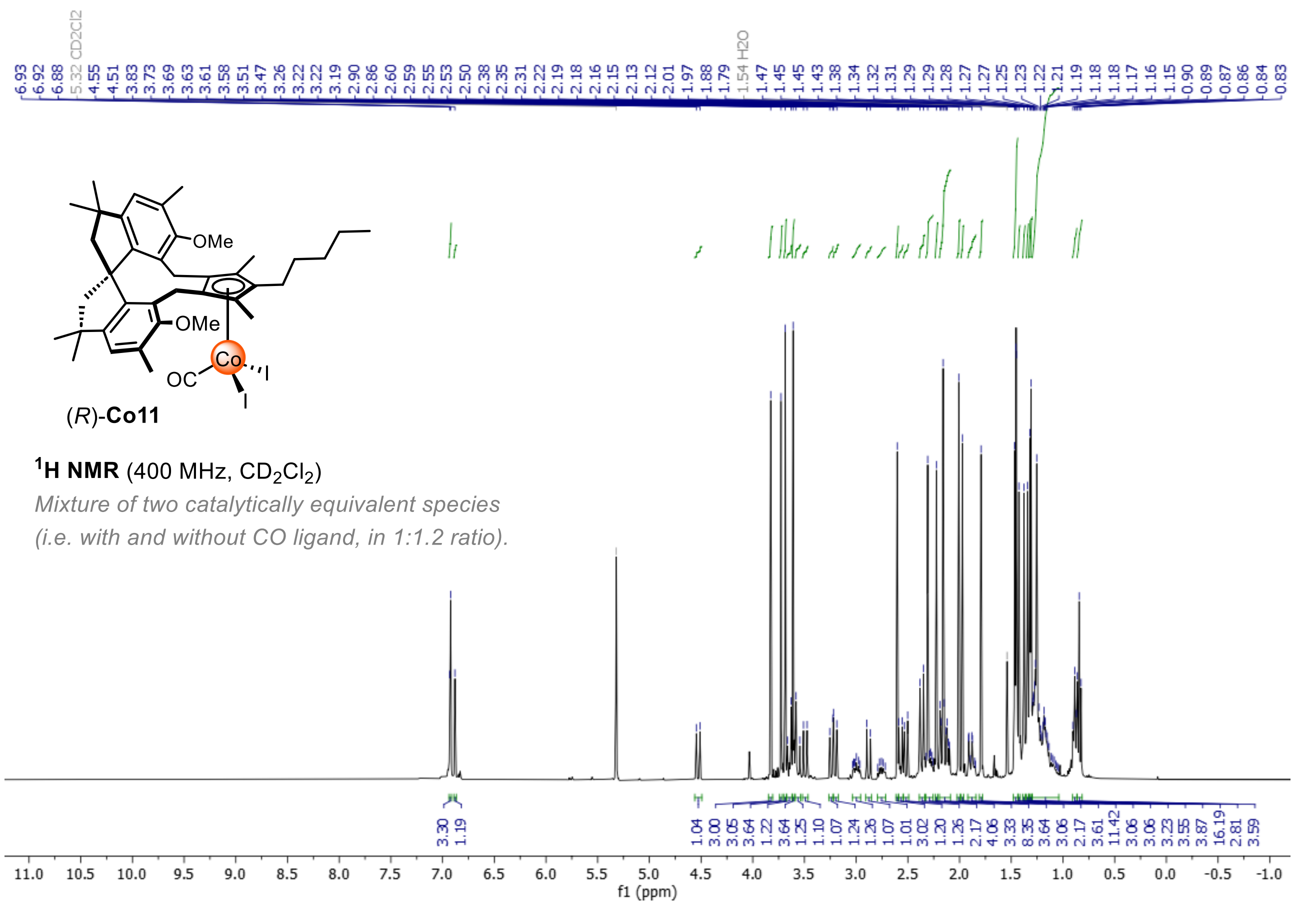
6.93
6.92
6.88
5.32 CD2Cl2
4.55
4.51
3.83
3.73
3.69
3.63
3.61
3.58
3.51
3.47
3.26
3.22
3.22
3.19
2.90
2.86
2.60
2.59
2.55
2.53
2.50
2.38
2.35
2.31
2.22
2.19
2.18
2.16
2.15
2.13
2.12
2.01
1.97
1.88
1.79
1.54 H2O
1.47
1.45
1.45
1.43
1.38
1.34
1.32
1.31
1.29
1.29
1.28
1.27
1.27
1.25
1.23
1.22
1.21
1.19
1.18
1.18
1.17
1.16
1.15
0.90
0.89
0.87
0.86
0.84
0.83
OMe
OMe
Co
OC
I
I
(*R*)-**Co11**
^{1}H NMR (400 MHz, CD_2Cl_2)
Mixture of two catalytically equivalent species
(i.e. with and without CO ligand, in 1:1.2 ratio).
3.30
1.19
1.04
3.00
3.05
3.64
1.22
3.64
1.25
1.10
1.07
1.24
1.26
1.07
1.01
3.02
1.20
1.26
2.17
4.06
3.33
8.35
3.64
3.06
2.17
3.61
11.42
3.06
3.06
3.23
3.55
3.87
16.19
2.81
3.59
11.0 10.5 10.0 9.5 9.0 8.5 8.0 7.5 7.0 6.5 6.0 5.5 5.0 4.5 4.0 3.5 3.0 2.5 2.0 1.5 1.0 0.5 0.0 -0.5 -1.0
f1 (ppm)

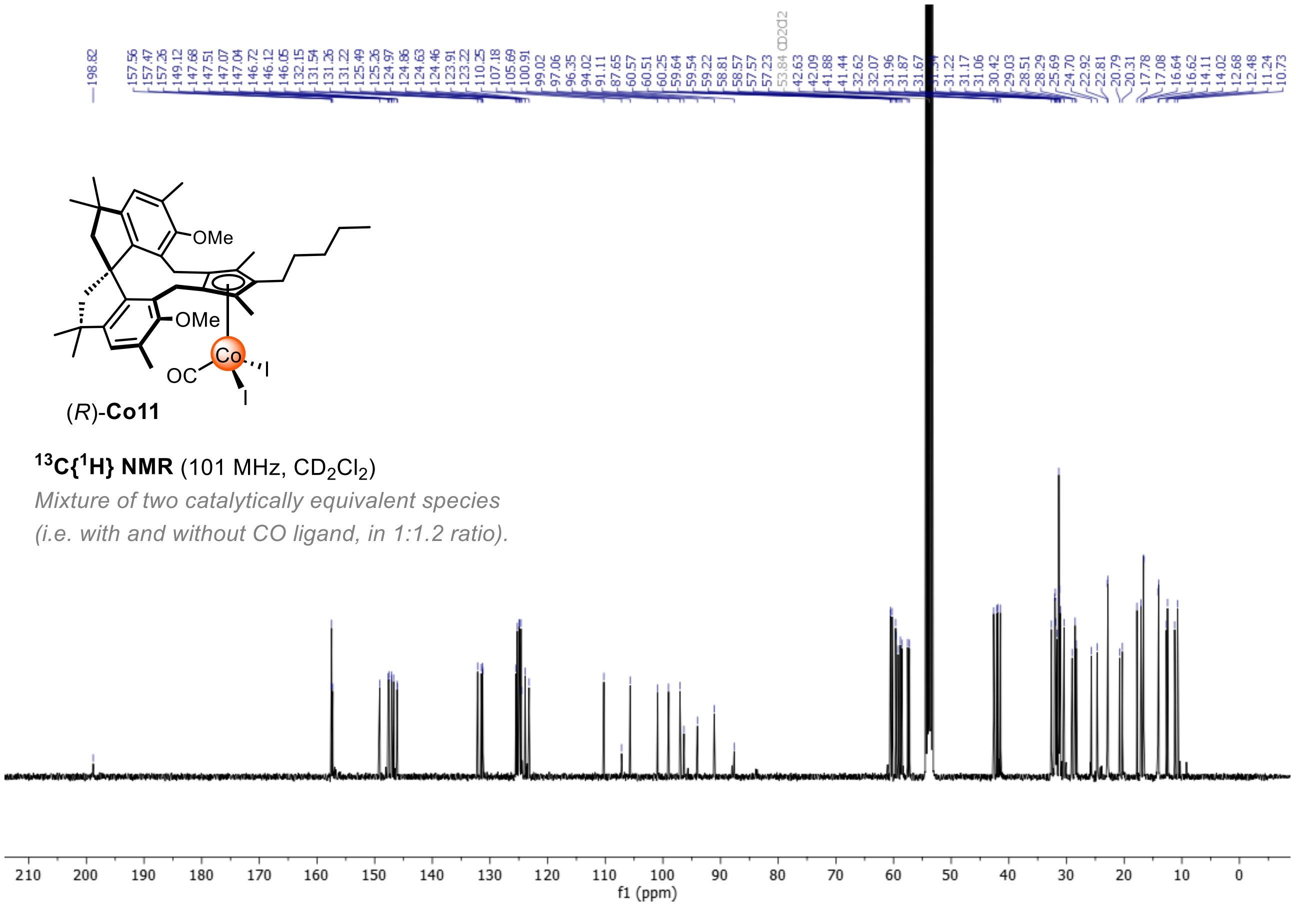
198.82
157.56
157.47
157.26
149.12
147.68
147.51
147.07
147.04
146.72
146.12
146.05
132.15
131.54
131.26
131.22
125.49
125.36
124.97
124.86
124.63
124.46
123.91
123.22
110.25
107.18
105.69
100.91
99.02
97.06
96.35
94.02
91.11
87.65
60.57
60.51
60.25
59.64
59.54
59.22
58.81
58.57
57.57
57.23
53.84 CD2Cl2
42.63
42.09
41.88
41.44
32.62
32.07
31.96
31.87
31.67
31.22
31.17
31.06
30.42
29.03
28.51
28.29
25.69
24.70
22.92
22.81
20.79
20.31
17.78
17.08
16.64
16.62
14.11
14.02
12.68
12.48
11.24
10.73
OMe
OMe
Co
OC
I
I
(*R*)-**Co11**
^{13}C{^{1}H} NMR (101 MHz, CD_2Cl_2)
Mixture of two catalytically equivalent species
(i.e. with and without CO ligand, in 1:1.2 ratio).
210 200 190 180 170 160 150 140 130 120 110 100 90 80 70 60 50 40 30 20 10 0
f1 (ppm)

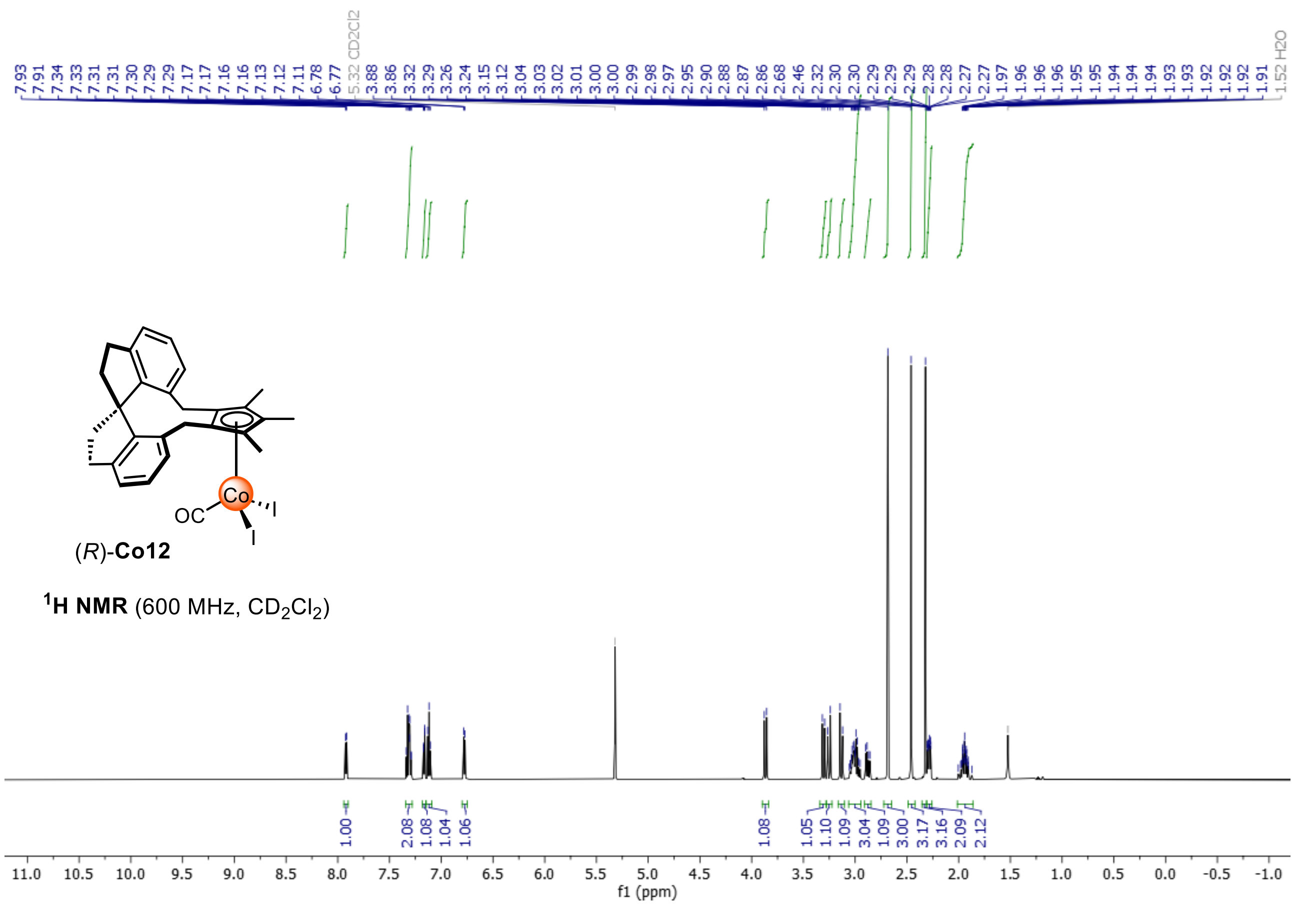
7.93 7.91 7.34 7.33 7.31 7.31 7.30 7.29 7.29 7.17 7.17 7.16 7.16 7.13 7.12 7.11 6.78 6.77 5.32 CD2Cl2 3.88 3.86 3.32 3.29 3.26 3.24 3.15 3.12 3.04 3.03 3.02 3.01 3.00 3.00 2.99 2.98 2.97 2.95 2.90 2.88 2.87 2.86 2.68 2.46 2.32 2.30 2.30 2.29 2.29 2.29 2.28 2.28 2.27 2.27 1.97 1.96 1.96 1.95 1.95 1.94 1.94 1.94 1.93 1.93 1.92 1.92 1.92 1.91 1.52 H2O
Co
OC
I
I
(R)-Co12
1H NMR (600 MHz, CD2Cl2)
1.00 2.08 1.08 1.04 1.06 1.08 1.05 1.10 1.09 3.04 1.09 3.00 3.17 3.16 2.09 2.12
11.0 10.5 10.0 9.5 9.0 8.5 8.0 7.5 7.0 6.5 6.0 5.5 5.0 4.5 4.0 3.5 3.0 2.5 2.0 1.5 1.0 0.5 0.0 -0.5 -1.0
f1 (ppm)

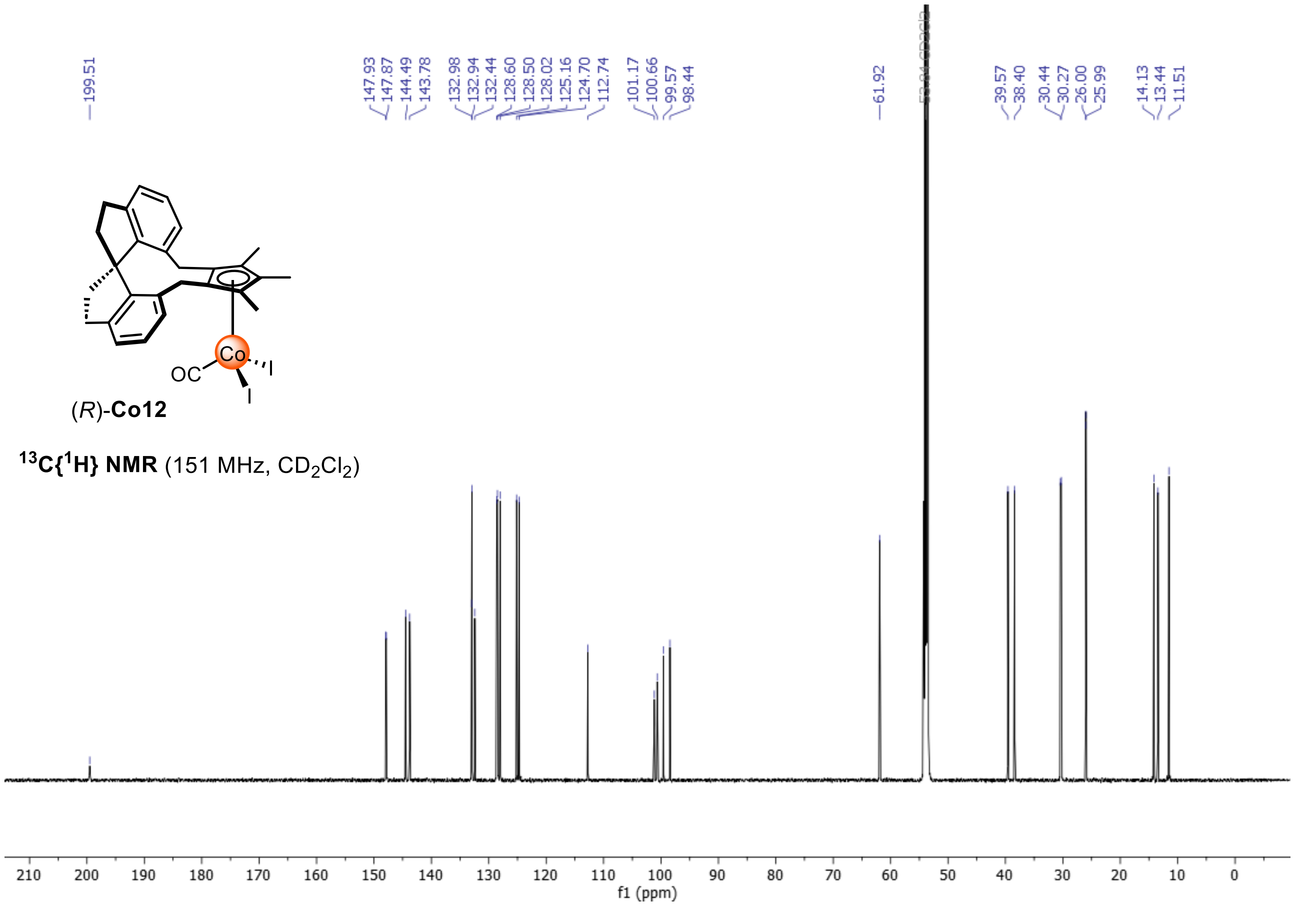
199.51 147.93 147.87 144.49 143.78 132.98 132.94 132.44 128.60 128.50 128.02 125.16 124.70 112.74 101.17 100.66 99.57 98.44 61.92 CD2Cl2 39.57 38.40 30.44 30.27 26.00 25.99 14.13 13.44 11.51
Co
OC
I
I
(R)-Co12
13C{1H} NMR (151 MHz, CD2Cl2)
210 200 190 180 170 160 150 140 130 120 110 100 90 80 70 60 50 40 30 20 10 0
f1 (ppm)

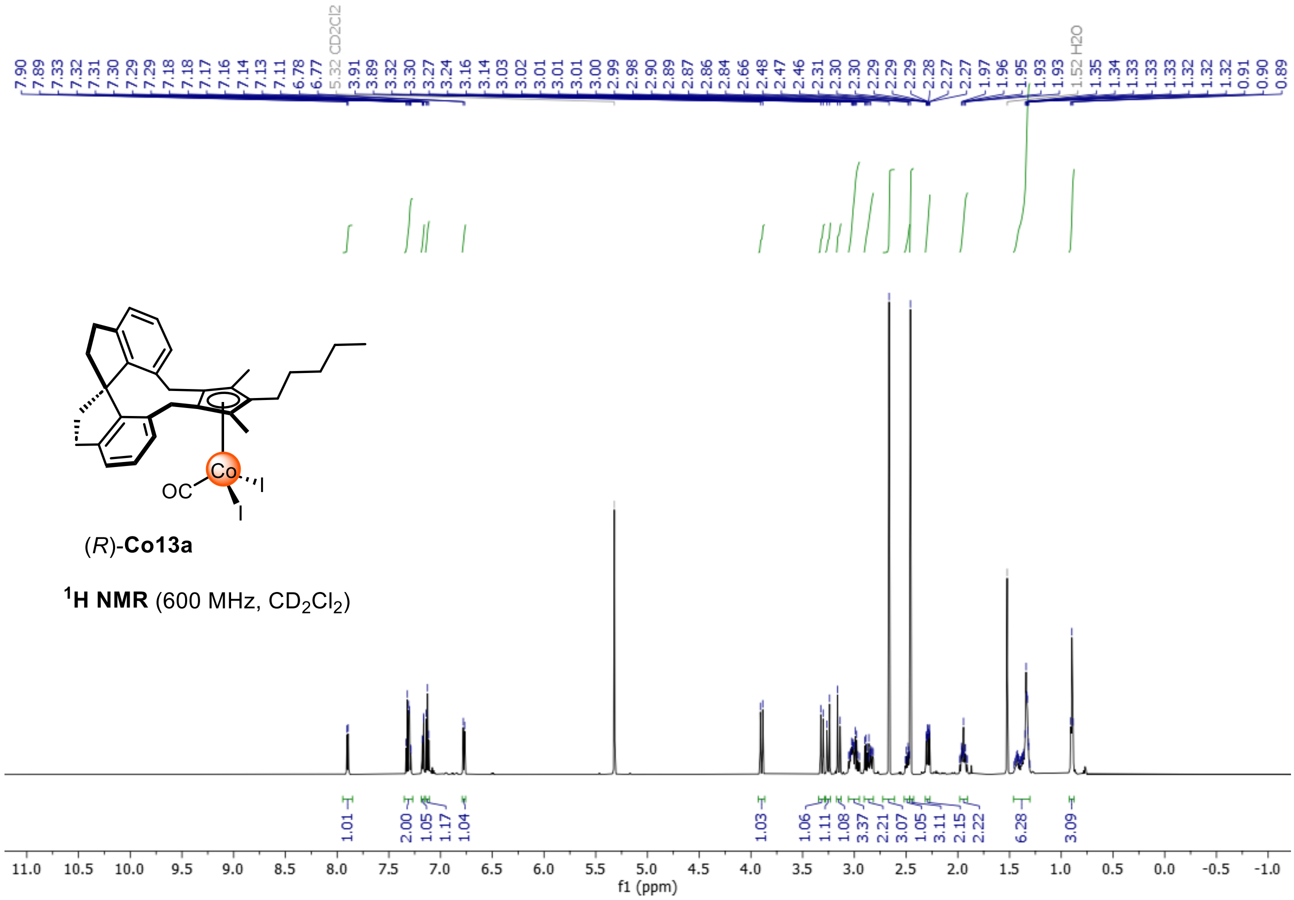

(*R*)-**Co13a**

^{1}H NMR (600 MHz, CD_2Cl_2)

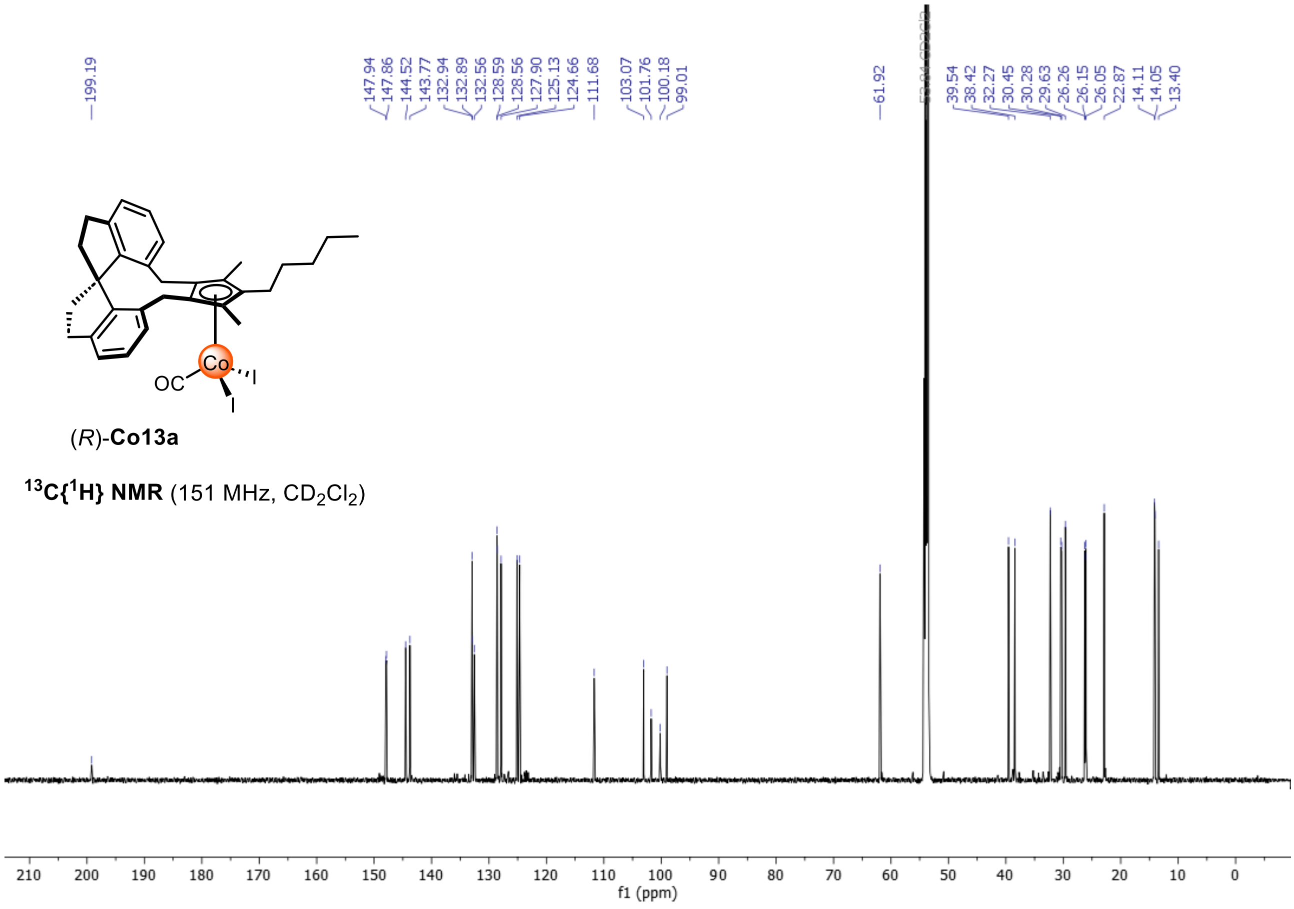

(*R*)-**Co13a**

^{13}C{^{1}H} NMR (151 MHz, CD_2Cl_2)

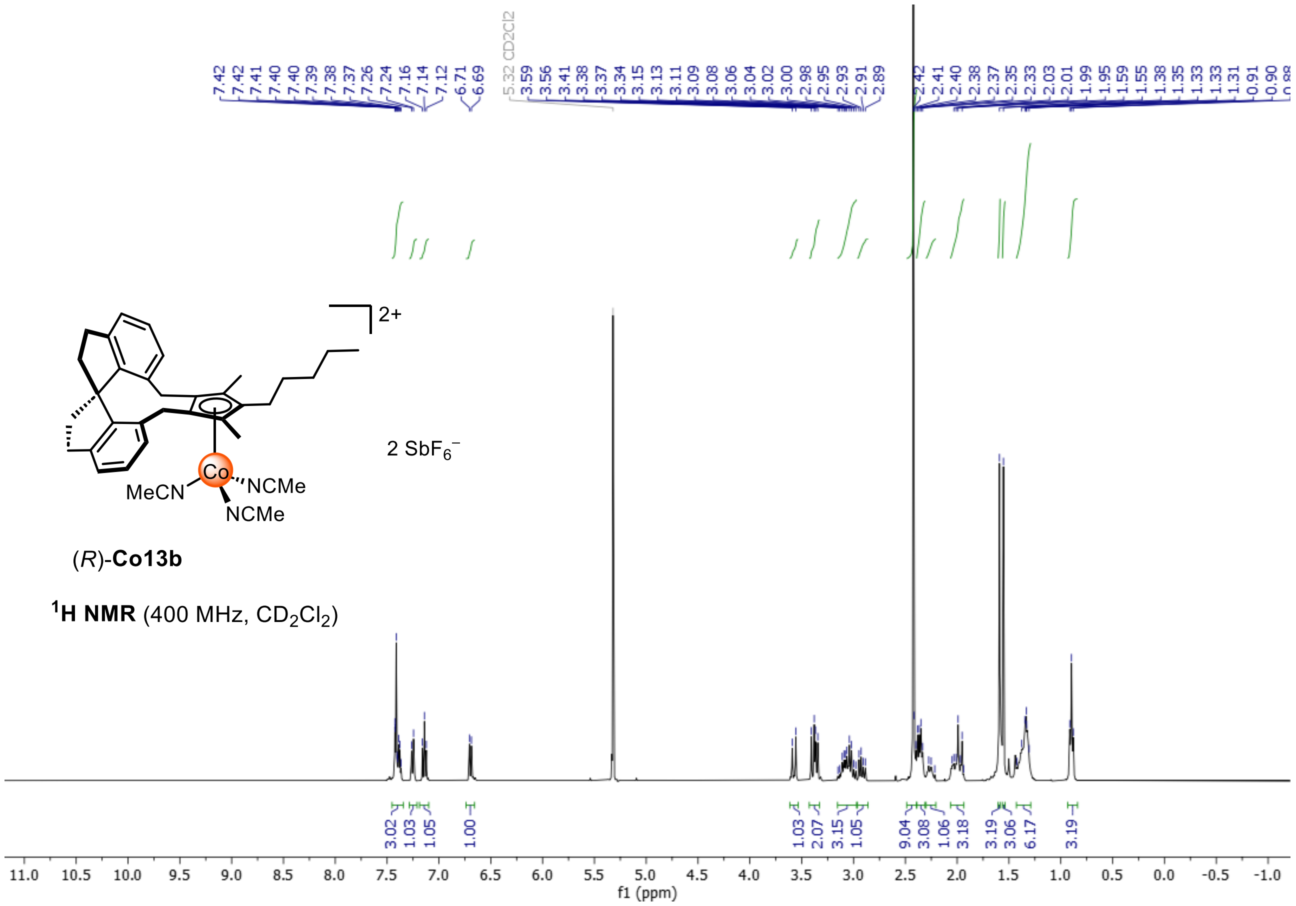
7.42
7.42
7.41
7.40
7.40
7.39
7.38
7.37
7.26
7.24
7.16
7.14
7.12
6.71
6.69
5.32 CD2Cl2
3.59
3.56
3.41
3.38
3.37
3.34
3.15
3.13
3.11
3.09
3.08
3.06
3.04
3.02
3.00
2.98
2.95
2.93
2.91
2.89
2.42
2.41
2.40
2.38
2.37
2.35
2.33
2.03
2.01
1.99
1.95
1.59
1.55
1.38
1.35
1.33
1.31
0.91
0.90
[illegible]
2+
2 SbF6⁻
Co
MeCN
NCMe
NCMe
(R)-Co13b
1H NMR (400 MHz, CD2Cl2)
3.02
1.03
1.05
1.00
1.03
2.07
3.15
1.05
9.04
3.08
1.06
3.18
3.19
3.06
6.17
3.19
11.0 10.5 10.0 9.5 9.0 8.5 8.0 7.5 7.0 6.5 6.0 5.5 5.0 4.5 4.0 3.5 3.0 2.5 2.0 1.5 1.0 0.5 0.0 -0.5 -1.0
f1 (ppm)

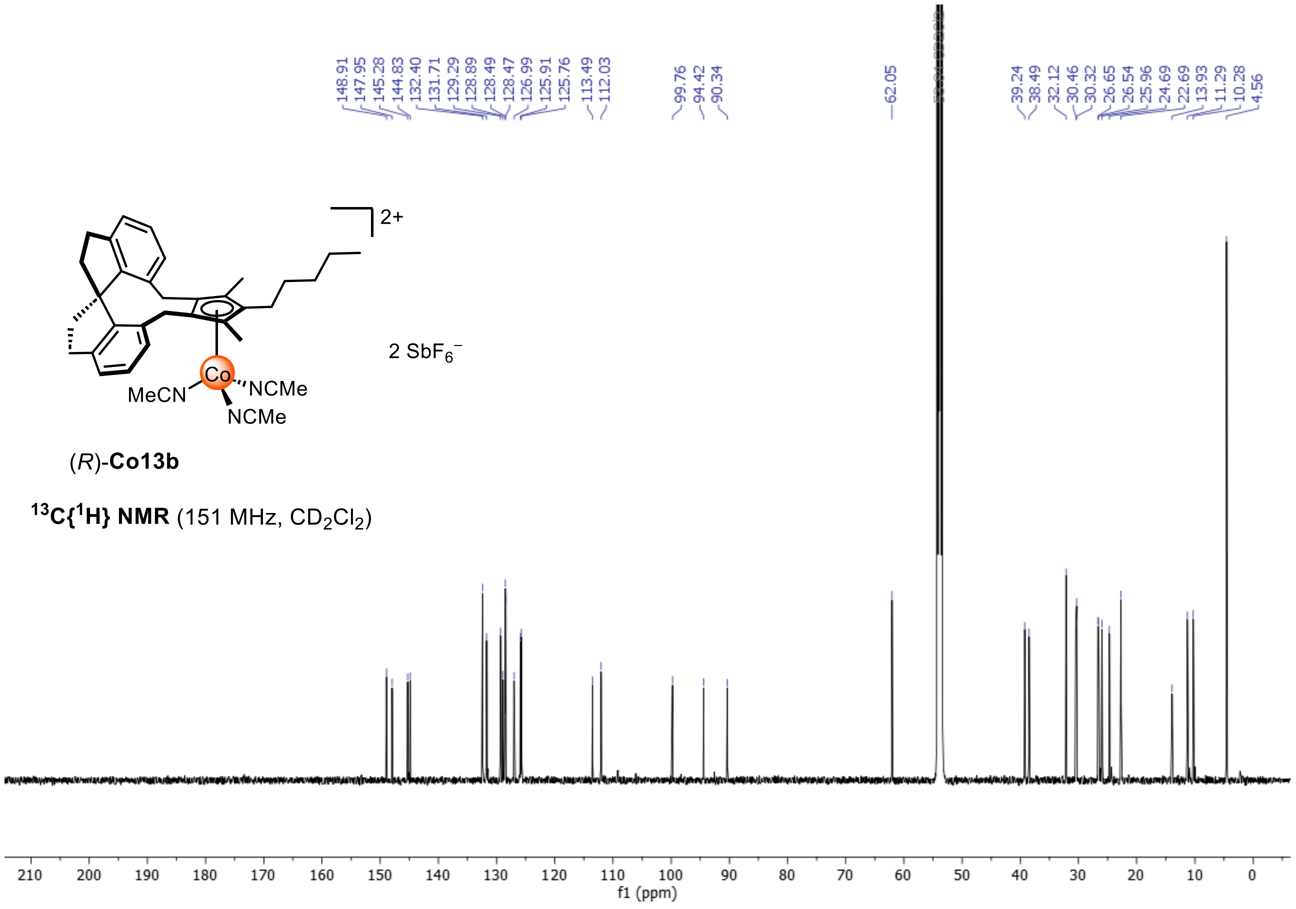
148.91
147.95
145.28
144.83
132.40
131.71
129.29
128.89
128.49
128.47
126.99
125.91
125.76
113.49
112.03
99.76
94.42
90.34
62.05
[illegible]
39.24
38.49
32.12
30.46
30.32
26.65
26.54
25.96
24.69
22.69
13.93
11.29
10.28
4.56
2+
2 SbF6⁻
Co
MeCN
NCMe
NCMe
(R)-Co13b
13C{1H} NMR (151 MHz, CD2Cl2)
210 200 190 180 170 160 150 140 130 120 110 100 90 80 70 60 50 40 30 20 10 0
f1 (ppm)

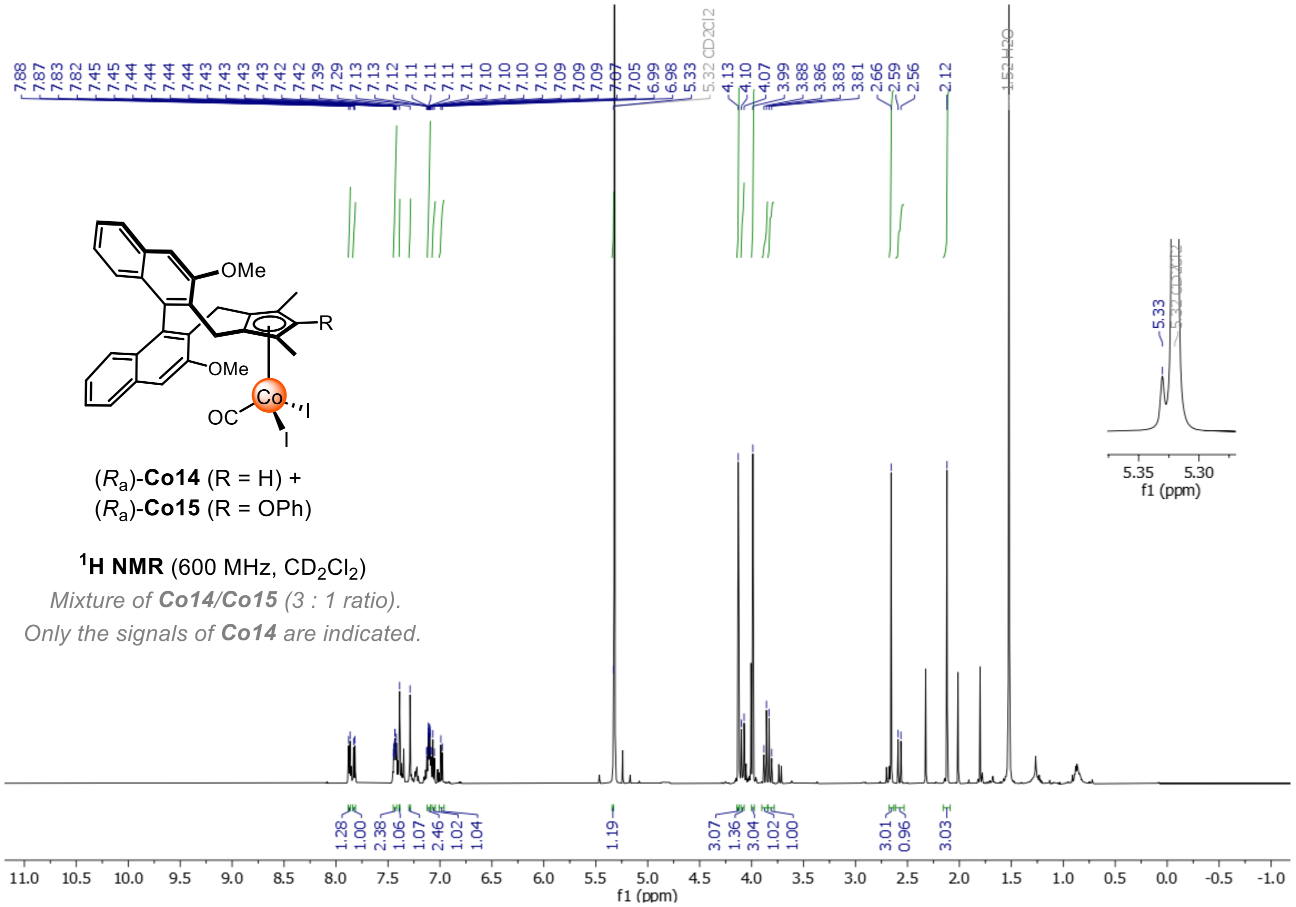
7.88 7.87 7.83 7.82 7.45 7.45 7.44 7.44 7.44 7.44 7.43 7.43 7.43 7.43 7.42 7.42 7.39 7.29 7.13 7.13 7.12 7.11 7.11 7.11 7.10 7.10 7.10 7.09 7.09 7.09 7.07 7.05 6.99 6.98 5.33
5.32 CD2Cl2
4.13 4.10 4.07 3.99 3.88 3.86 3.83 3.81 2.66 2.59 2.56 2.12
1.52 H2O
OMe
R
OMe
Co
OC
I
I
(Ra)-Co14 (R = H) +
(Ra)-Co15 (R = OPh)
1H NMR (600 MHz, CD2Cl2)
Mixture of Co14/Co15 (3 : 1 ratio).
Only the signals of Co14 are indicated.
5.33
5.32 CD2Cl2
5.35 5.30
f1 (ppm)
1.28 1.00 2.38 1.06 1.07 2.46 1.02 1.04 1.19 3.07 1.36 3.04 1.02 1.00 3.01 0.96 3.03
11.0 10.5 10.0 9.5 9.0 8.5 8.0 7.5 7.0 6.5 6.0 5.5 5.0 4.5 4.0 3.5 3.0 2.5 2.0 1.5 1.0 0.5 0.0 -0.5 -1.0
f1 (ppm)

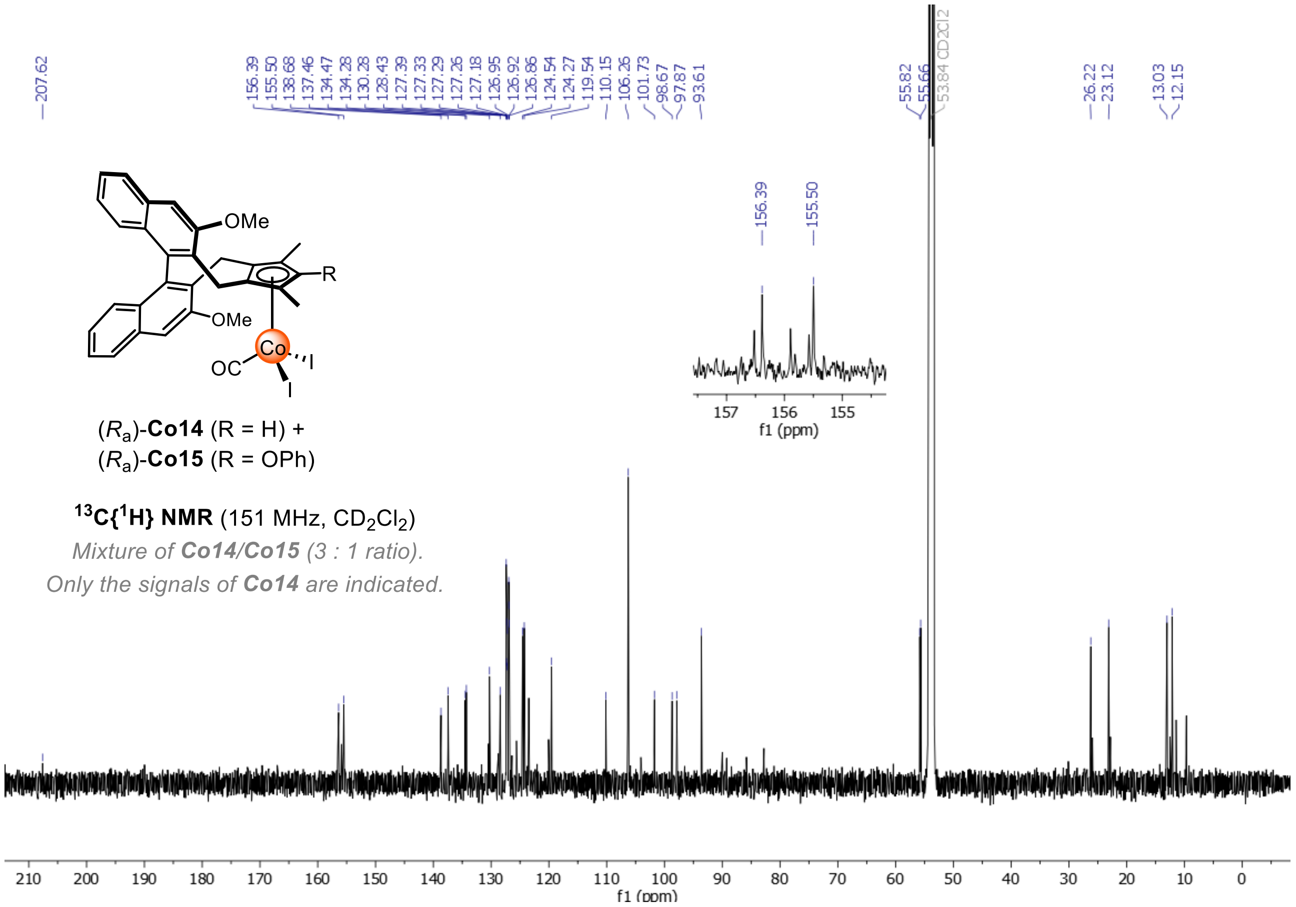
207.62
156.39 155.50 138.68 137.46 134.47 134.28 130.28 128.43 127.39 127.33 127.29 127.26 127.18 126.95 126.92 126.86 124.54 124.27 119.54 110.15 106.26 101.73 98.67 97.87 93.61
55.82 55.66
53.84 CD2Cl2
26.22 23.12 13.03 12.15
OMe
R
OMe
Co
OC
I
I
(Ra)-Co14 (R = H) +
(Ra)-Co15 (R = OPh)
13C{1H} NMR (151 MHz, CD2Cl2)
Mixture of Co14/Co15 (3 : 1 ratio).
Only the signals of Co14 are indicated.
156.39 155.50
157 156 155
f1 (ppm)
210 200 190 180 170 160 150 140 130 120 110 100 90 80 70 60 50 40 30 20 10 0
f1 (ppm)

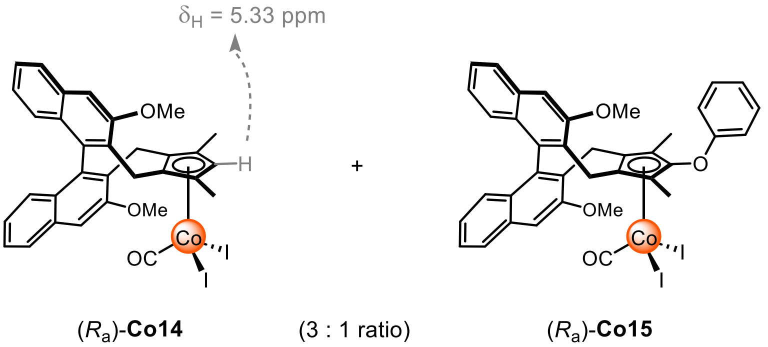

HSQC (600 MHz, CD_2Cl_2)

Key interaction of the major complex ***Co14*** *is indicated.*

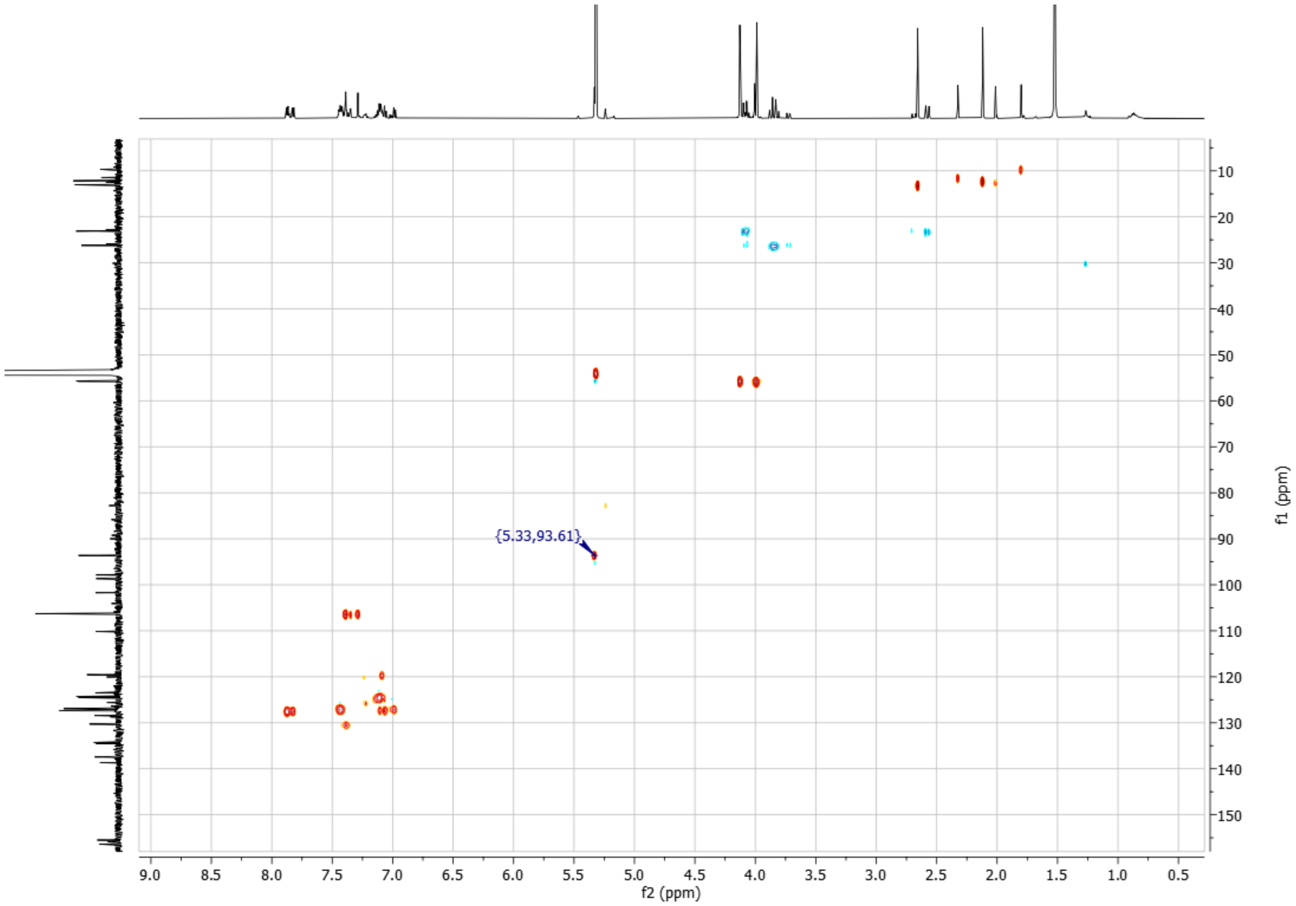

(*R*a)-**Rh1a**

^{1}H NMR (400 MHz, d_6-DMSO)

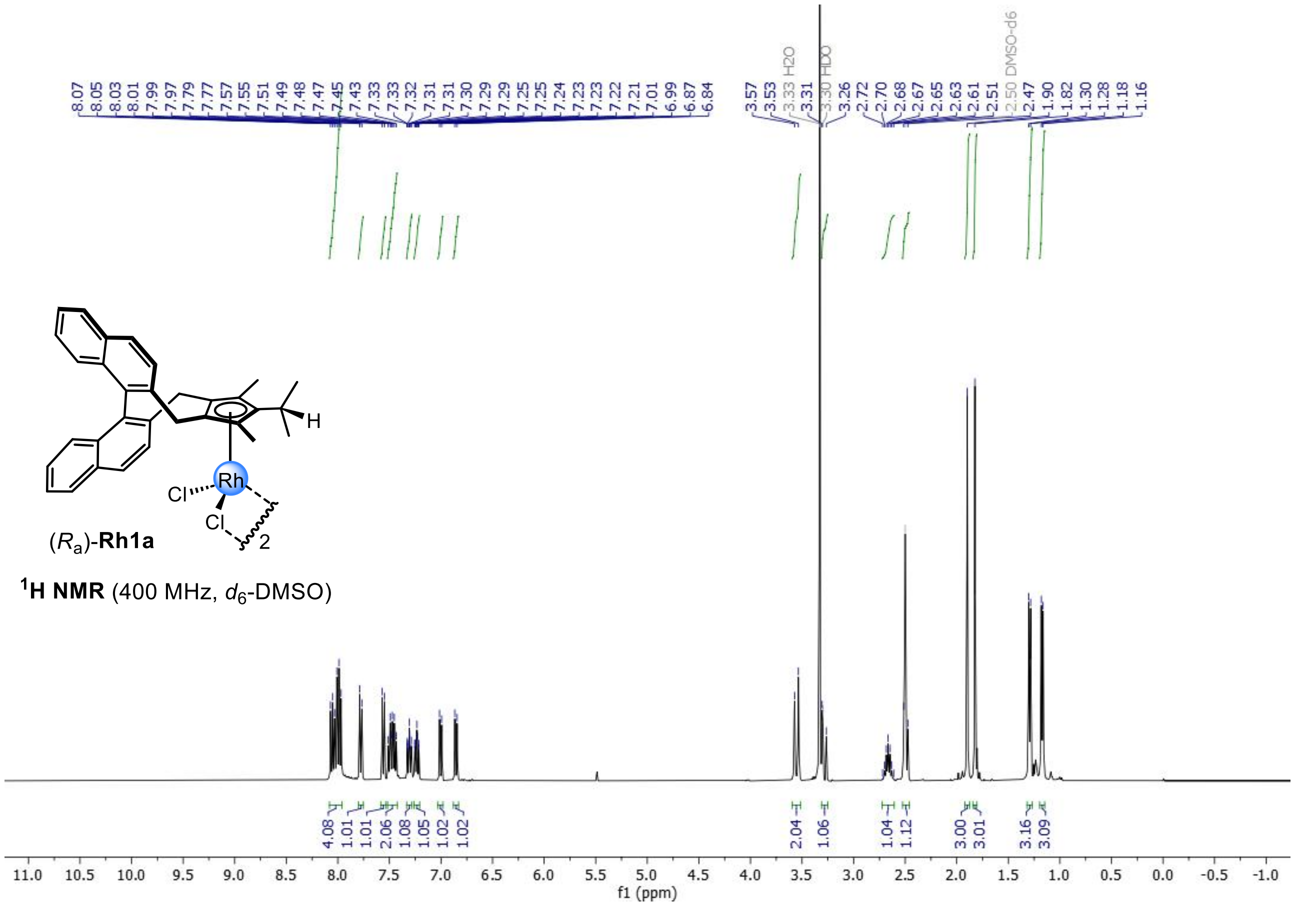

(*R*a)-**Rh1a**

^{13}C{^{1}H} NMR (101 MHz, d_6-DMSO)

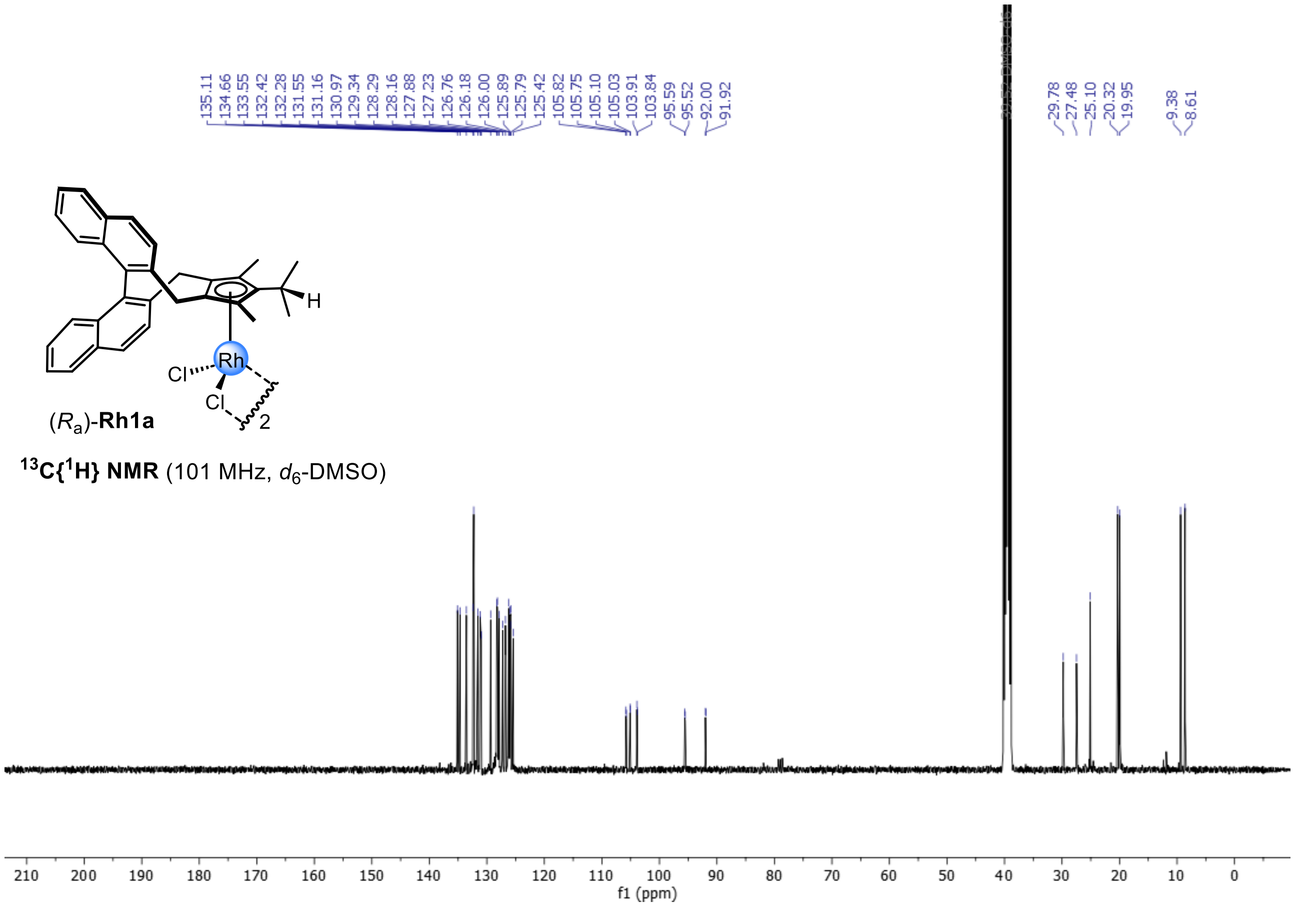

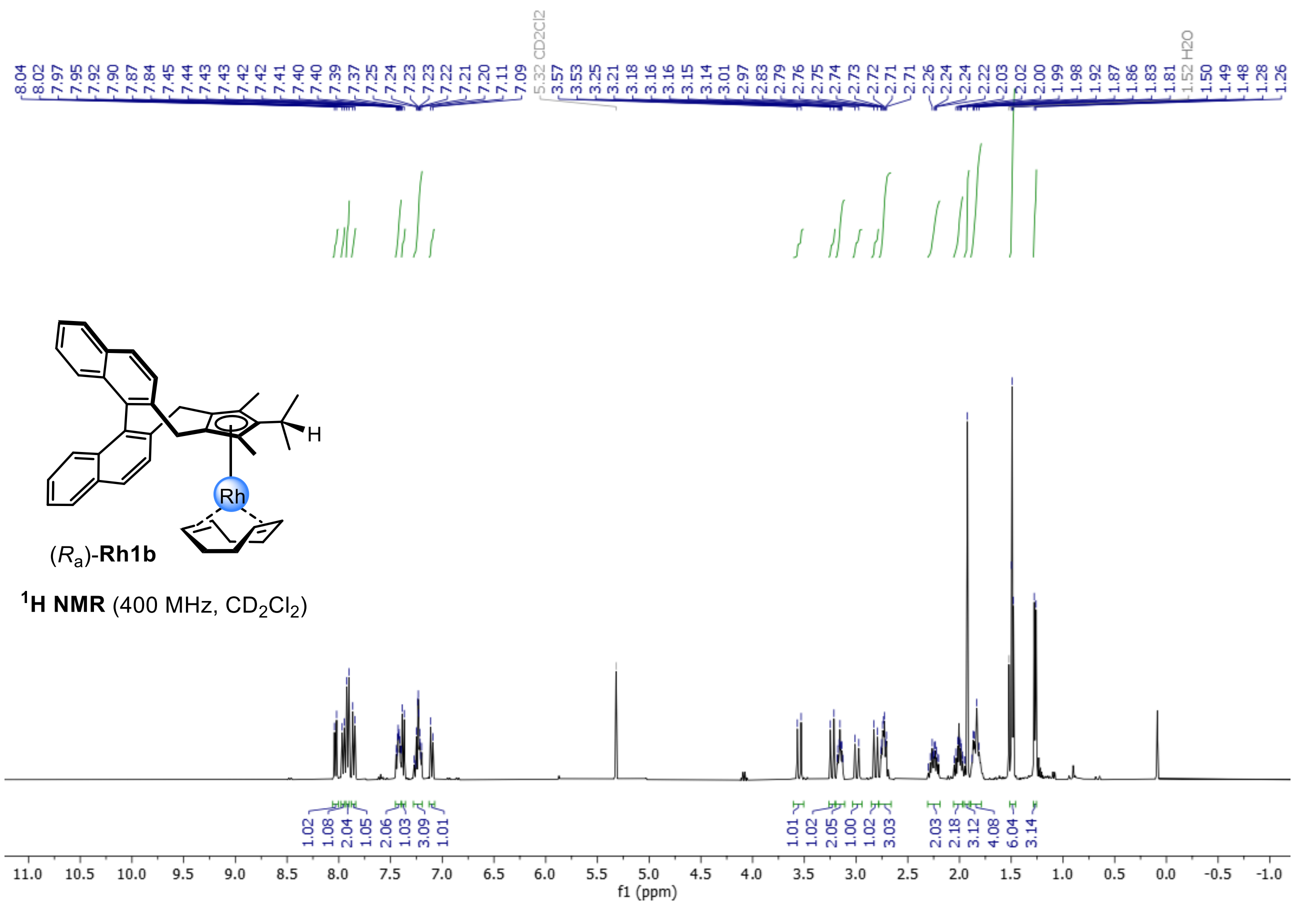
(Ra)-Rh1b
1H NMR (400 MHz, CD2Cl2)
Rh
H
f1 (ppm)

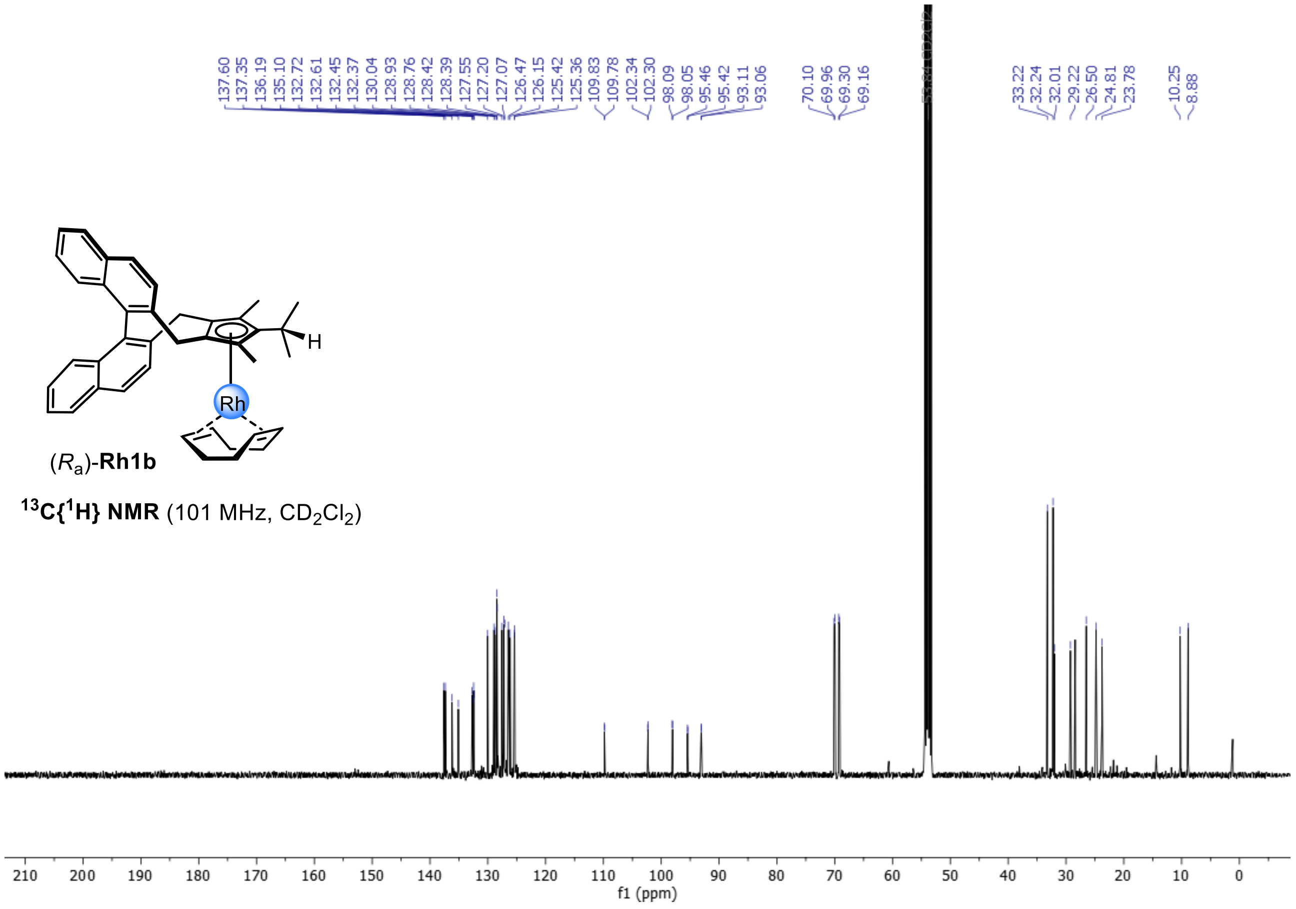
(Ra)-Rh1b
13C{1H} NMR (101 MHz, CD2Cl2)
Rh
H
f1 (ppm)

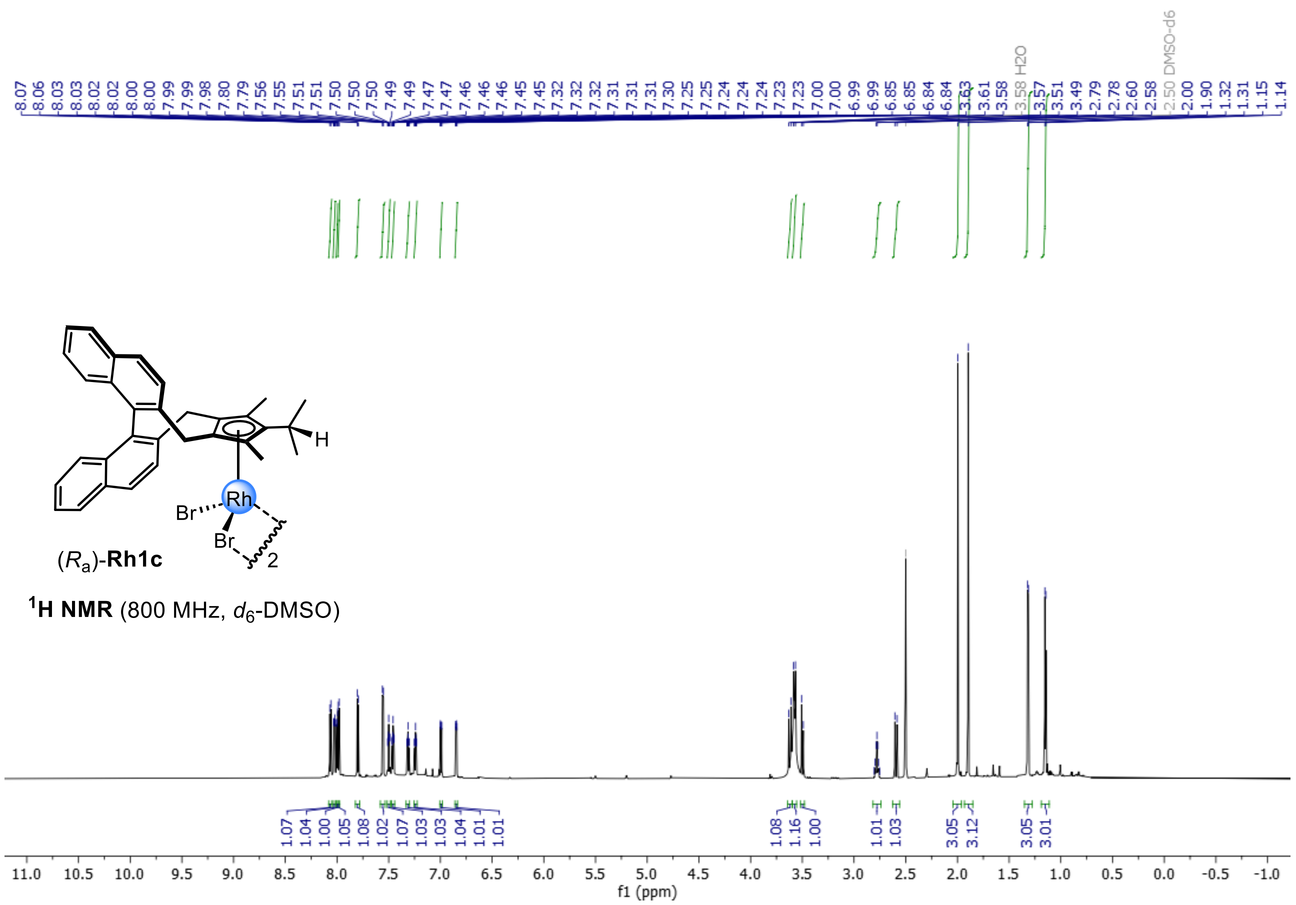

(R_a)-**Rh1c**

^{1}H NMR (800 MHz, d_6-DMSO)

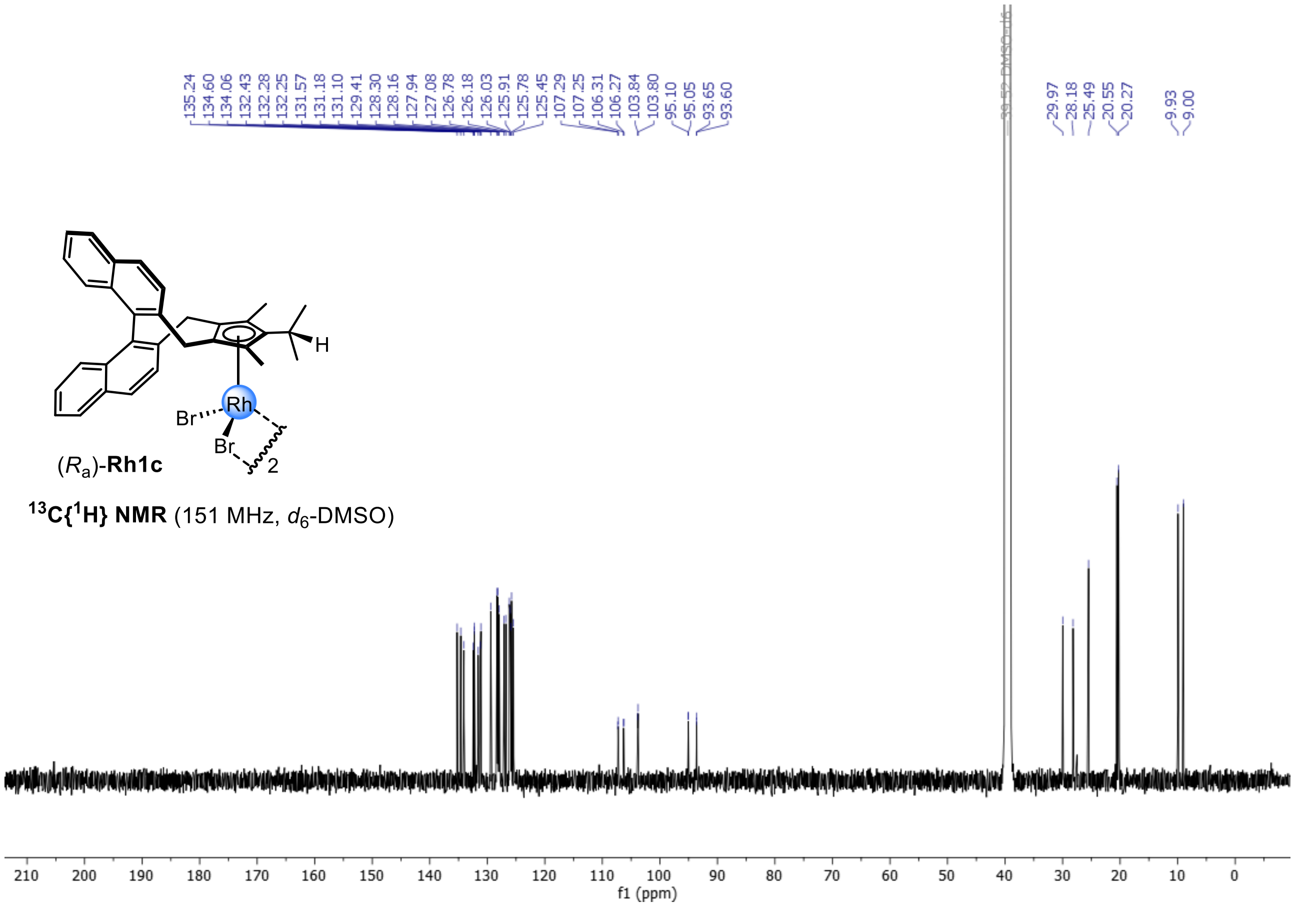

(R_a)-**Rh1c**

^{13}C{^{1}H} NMR (151 MHz, d_6-DMSO)

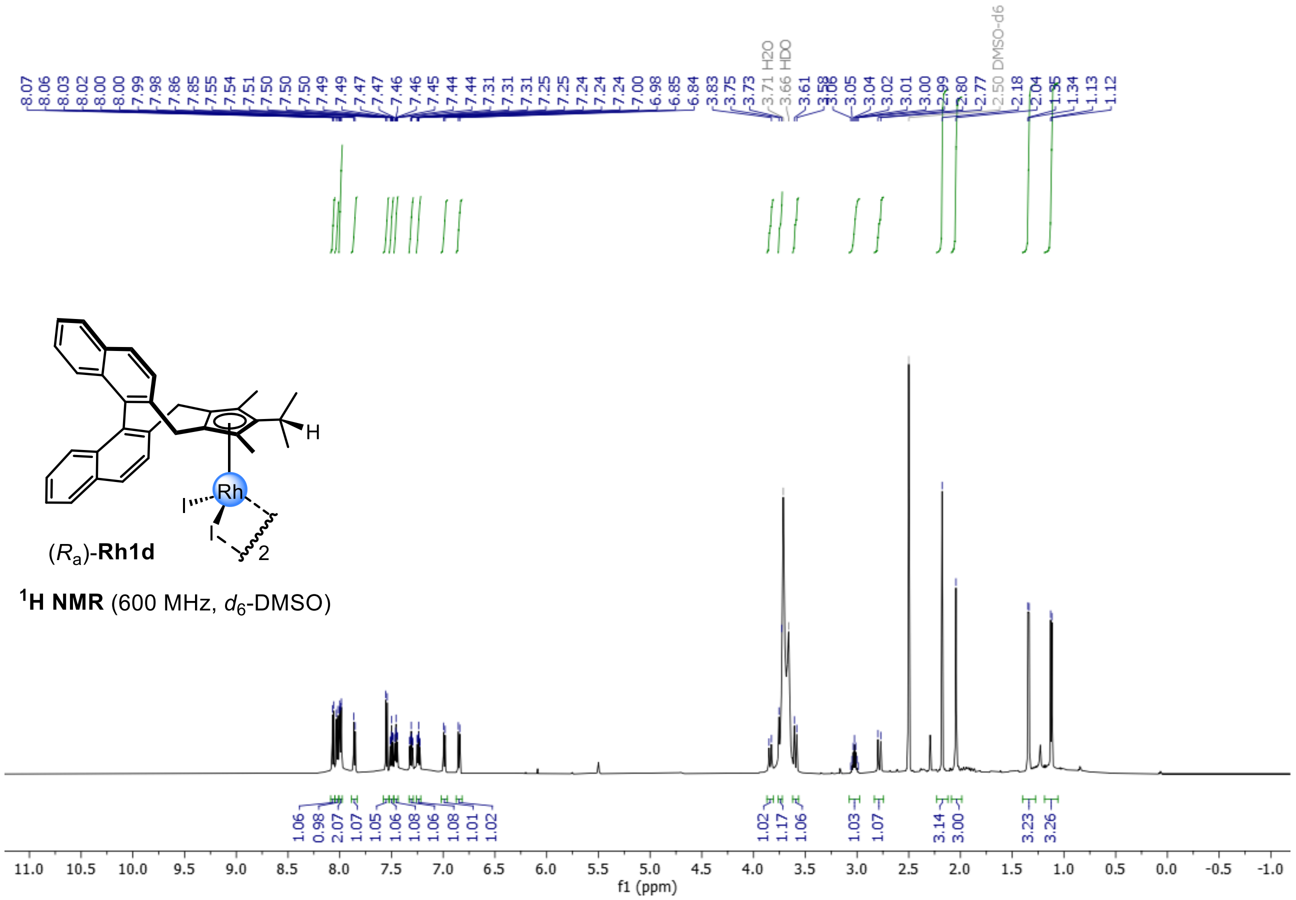

(R_a)-**Rh1d**

^{1}H NMR (600 MHz, d_6-DMSO)

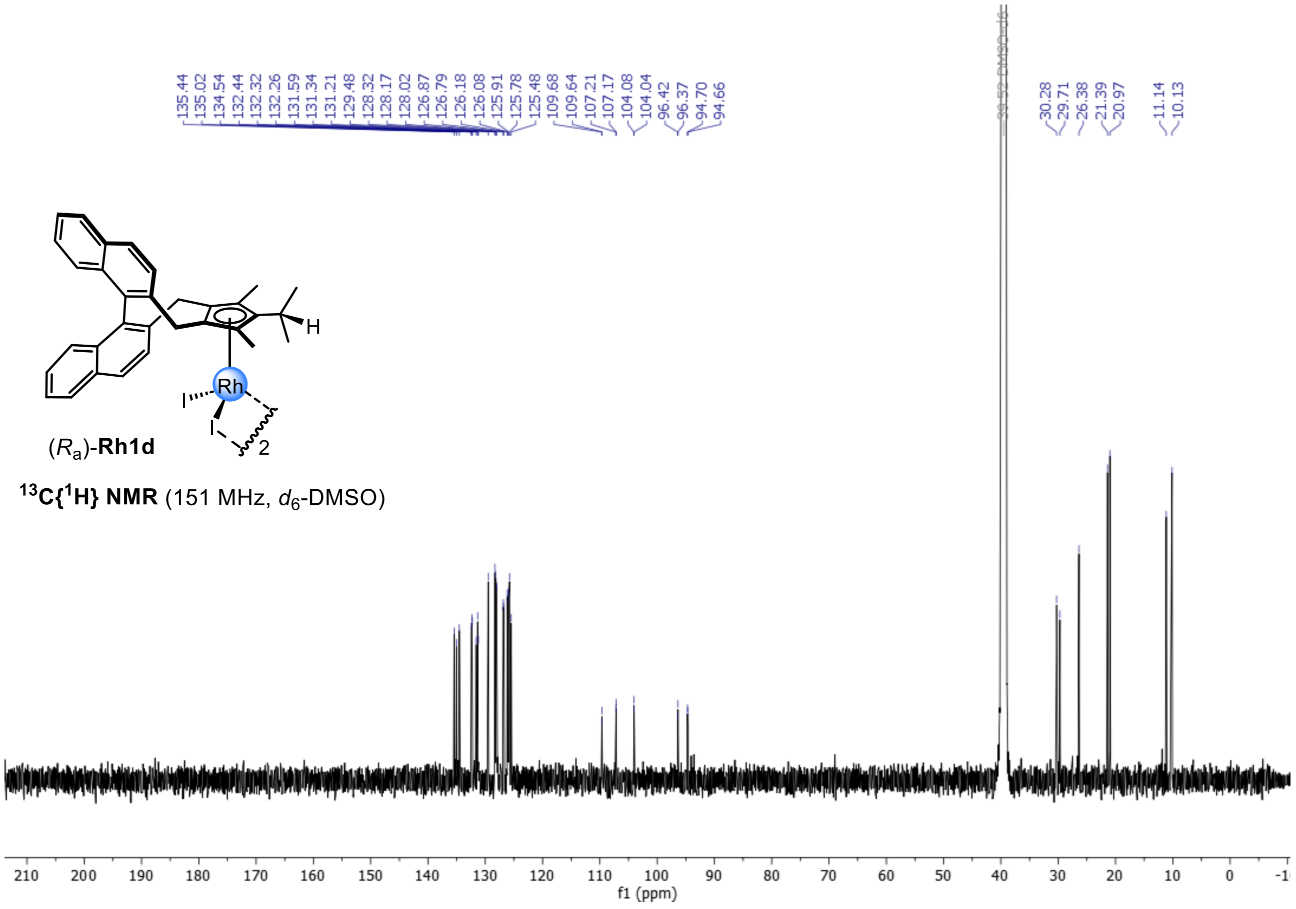

(R_a)-**Rh1d**

^{13}C{^{1}H} NMR (151 MHz, d_6-DMSO)

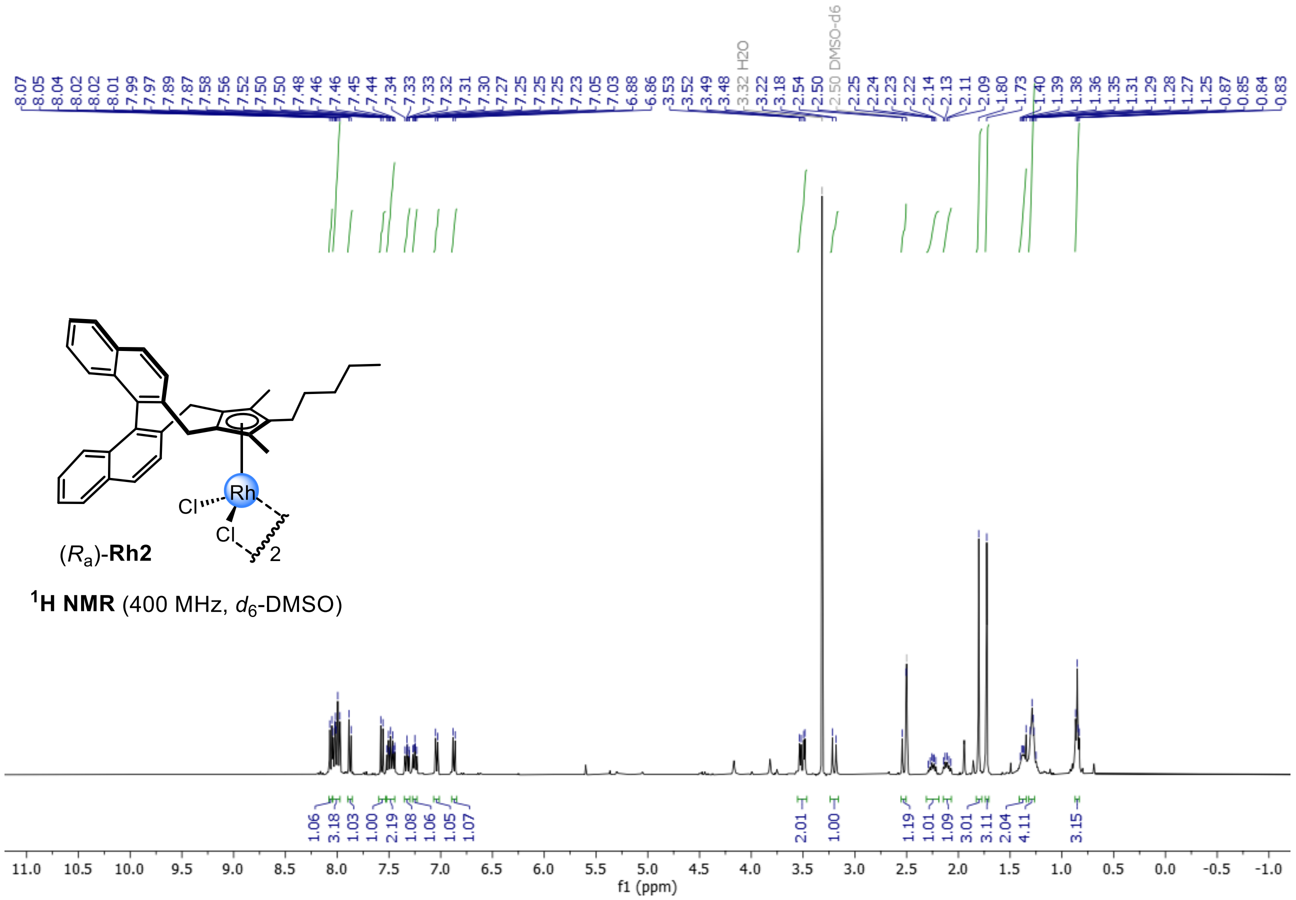
8.07 8.05 8.04 8.02 8.02 8.01 7.99 7.97 7.89 7.87 7.58 7.56 7.52 7.50 7.50 7.48 7.46 7.46 7.45 7.44 7.34 7.33 7.33 7.32 7.31 7.30 7.27 7.25 7.25 7.25 7.23 7.05 7.03 6.88 6.86 3.53 3.52 3.49 3.48 3.32 H2O 3.22 3.18 2.54 2.50 2.50 DMSO-d6 2.25 2.24 2.23 2.22 2.14 2.13 2.11 2.09 1.80 1.73 1.40 1.39 1.38 1.36 1.35 1.31 1.29 1.28 1.27 1.25 0.87 0.85 0.84 0.83
Cl
Rh
Cl
2
(Ra)-Rh2
1H NMR (400 MHz, d6-DMSO)
1.06 3.18 1.03 1.00 2.19 1.08 1.06 1.05 1.07 2.01 1.00 1.19 1.01 1.09 3.01 3.11 2.04 4.11 3.15
f1 (ppm)

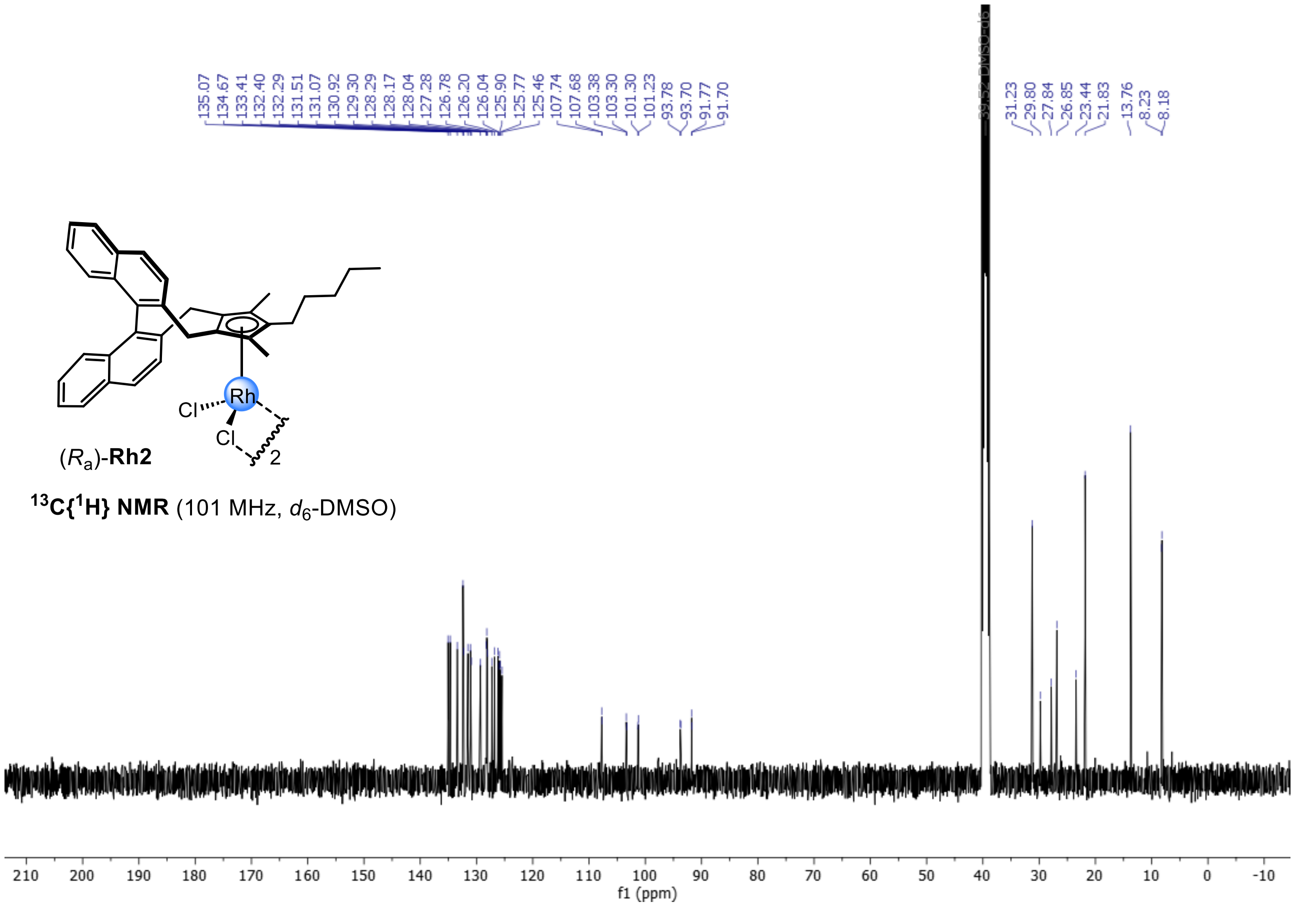
135.07 134.67 133.41 132.40 132.29 131.51 131.07 130.92 129.30 128.29 128.17 128.04 127.28 126.78 126.20 126.04 125.90 125.77 125.46 107.74 107.68 103.38 103.30 101.30 101.23 93.78 93.70 91.77 91.70
39.52 DMSO-d6
31.23 29.80 27.84 26.85 23.44 21.83 13.76 8.23 8.18
Cl
Rh
Cl
2
(Ra)-Rh2
13C{1H} NMR (101 MHz, d6-DMSO)
f1 (ppm)

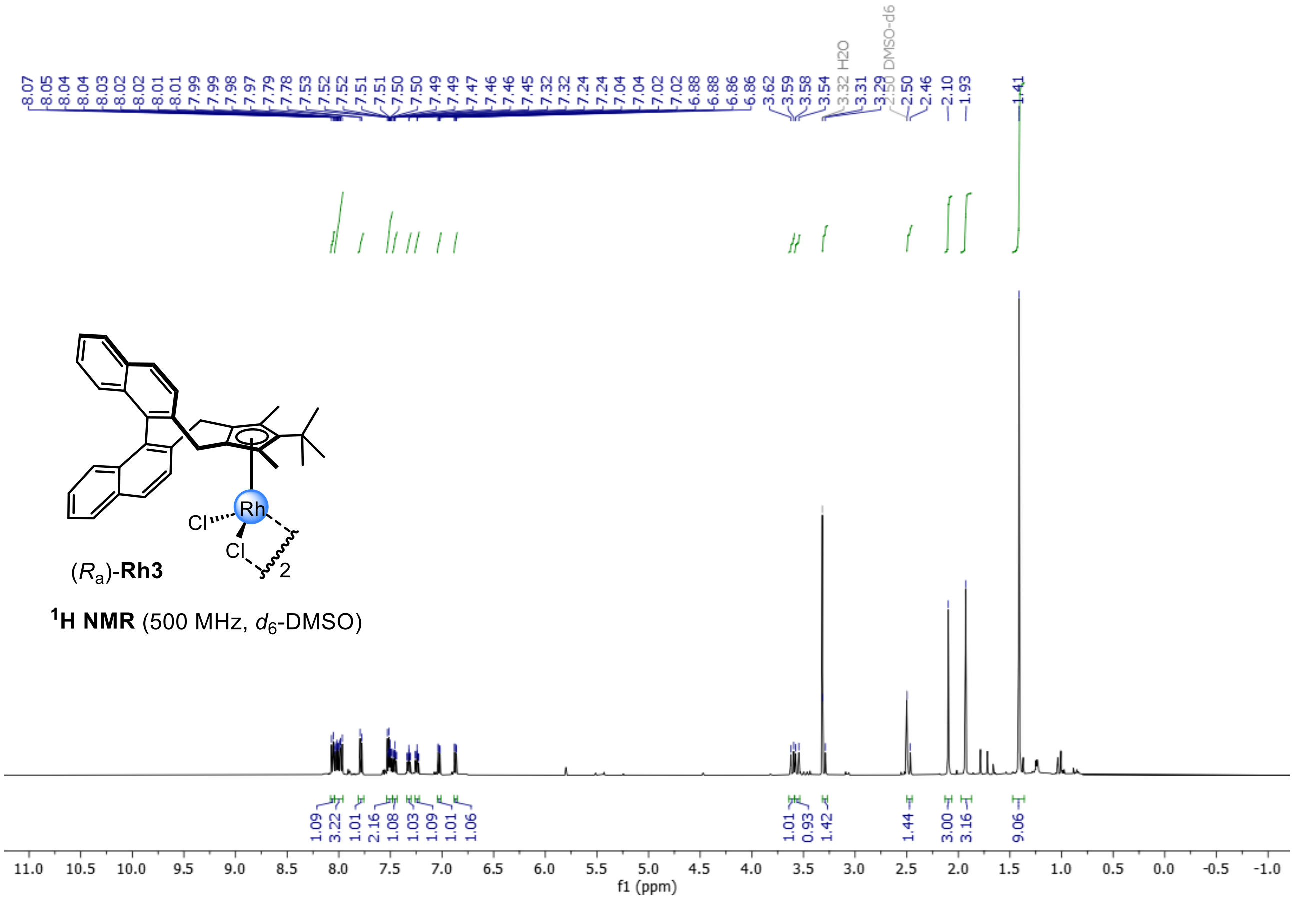
(Ra)-Rh3
1H NMR (500 MHz, d6-DMSO)
f1 (ppm)

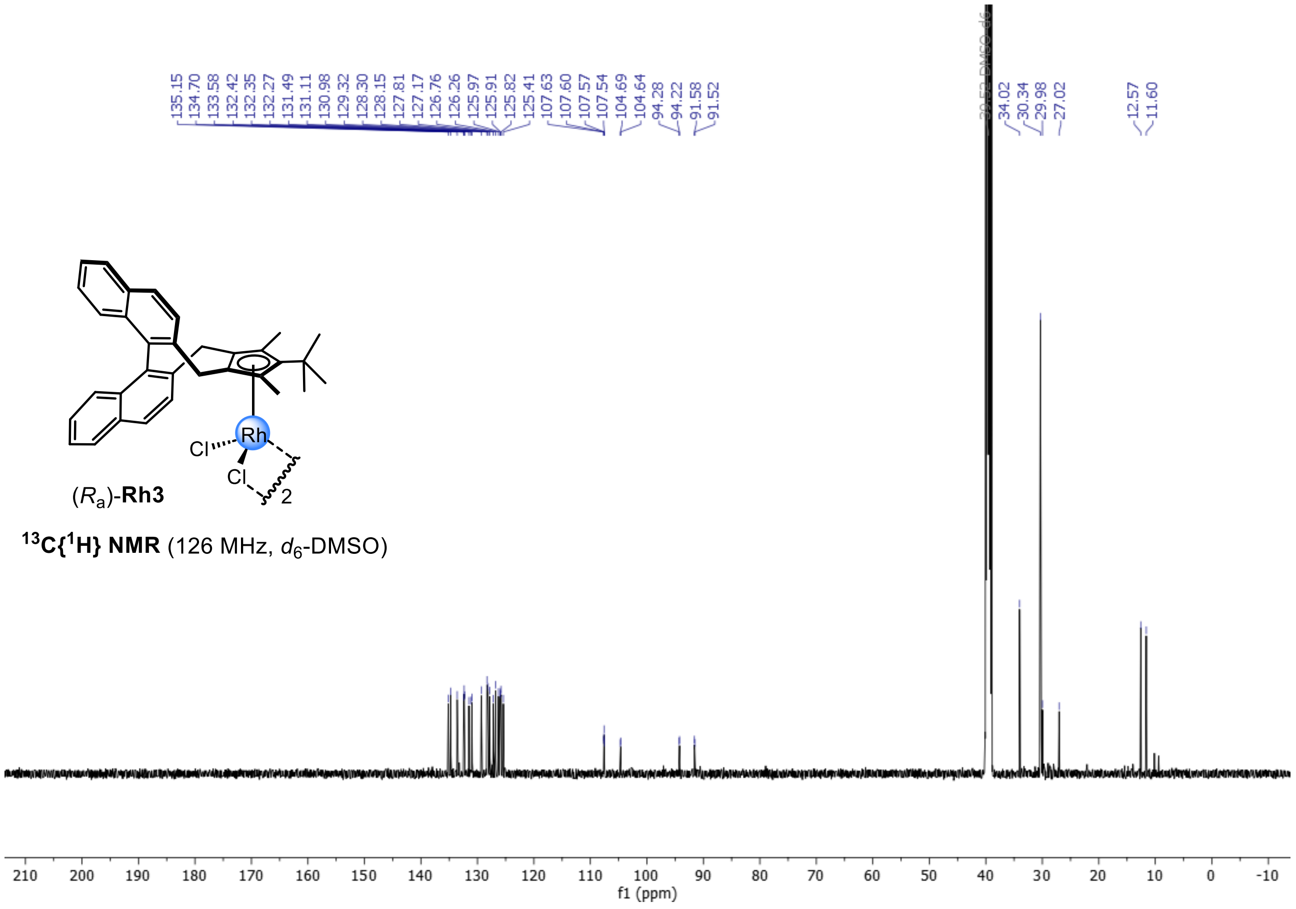
(Ra)-Rh3
13C{1H} NMR (126 MHz, d6-DMSO)
f1 (ppm)

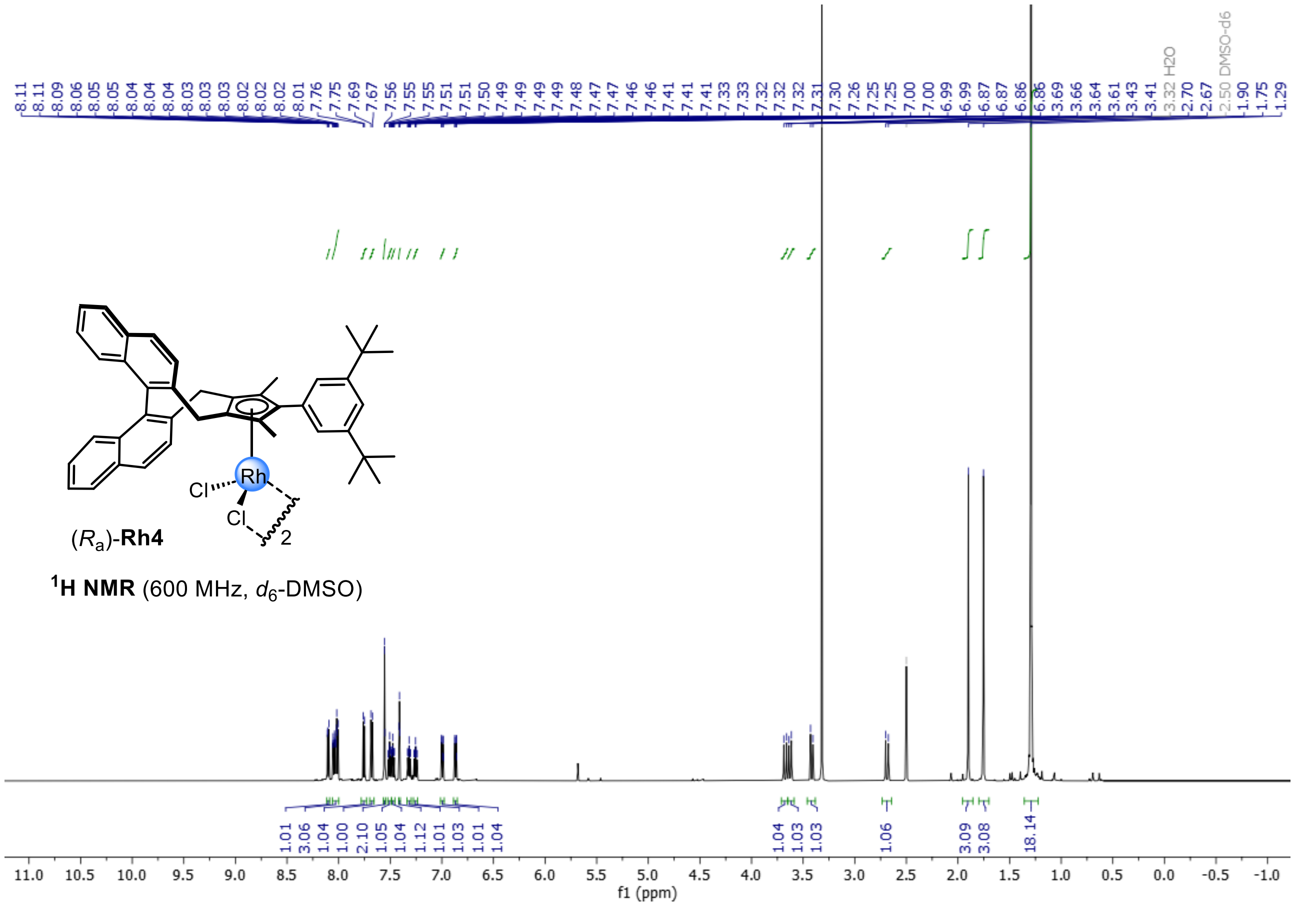
8.11 8.11 8.09 8.06 8.05 8.05 8.04 8.04 8.04 8.03 8.03 8.03 8.02 8.02 8.01 7.76 7.75 7.69 7.67 7.56 7.55 7.55 7.51 7.50 7.49 7.49 7.49 7.48 7.47 7.47 7.46 7.46 7.41 7.41 7.41 7.33 7.33 7.32 7.32 7.31 7.30 7.26 7.25 7.25 7.00 7.00 6.99 6.99 6.87 6.87 6.86 6.86 3.69 3.66 3.64 3.61 3.43 3.41
3.32 H2O
2.70 2.67
2.50 DMSO-d6
1.90 1.75 1.29
Cl
Rh
Cl
2
(Ra)-Rh4
1H NMR (600 MHz, d6-DMSO)
1.01 3.06 1.04 1.00 2.10 1.05 1.04 1.12 1.01 1.03 1.01 1.04 1.04 1.03 1.03 1.06 3.09 3.08 18.14
11.0 10.5 10.0 9.5 9.0 8.5 8.0 7.5 7.0 6.5 6.0 5.5 5.0 4.5 4.0 3.5 3.0 2.5 2.0 1.5 1.0 0.5 0.0 -0.5 -1.0
f1 (ppm)

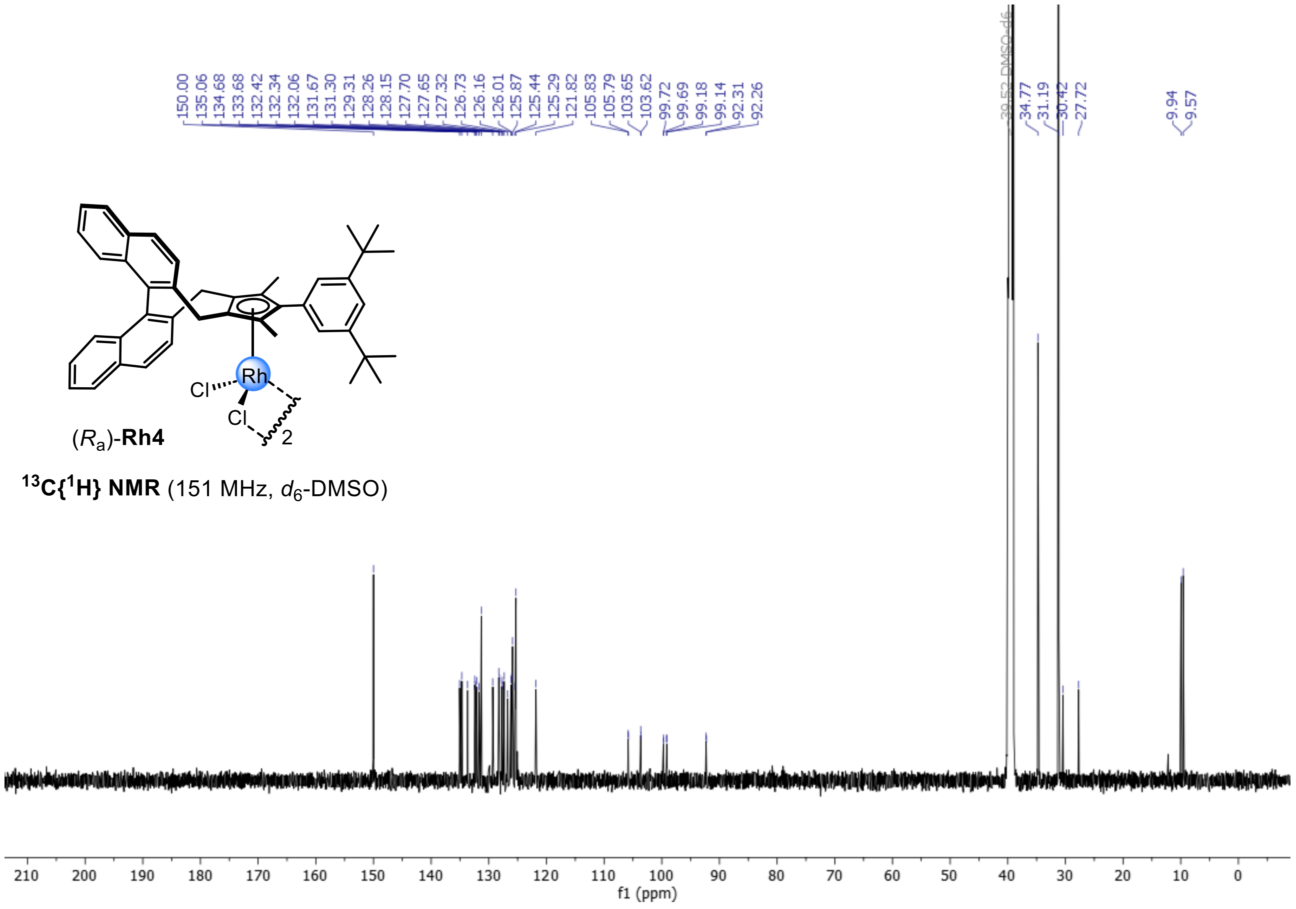
150.00 135.06 134.68 133.68 132.42 132.34 132.06 131.67 131.30 129.31 128.26 128.15 127.70 127.65 127.32 126.73 126.16 126.01 125.87 125.44 125.29 121.82 105.83 105.79 103.65 103.62 99.72 99.69 99.18 99.14 92.31 92.26
39.52 DMSO-d6
34.77 31.19 30.42 27.72 9.94 9.57
Cl
Rh
Cl
2
(Ra)-Rh4
13C{1H} NMR (151 MHz, d6-DMSO)
210 200 190 180 170 160 150 140 130 120 110 100 90 80 70 60 50 40 30 20 10 0
f1 (ppm)

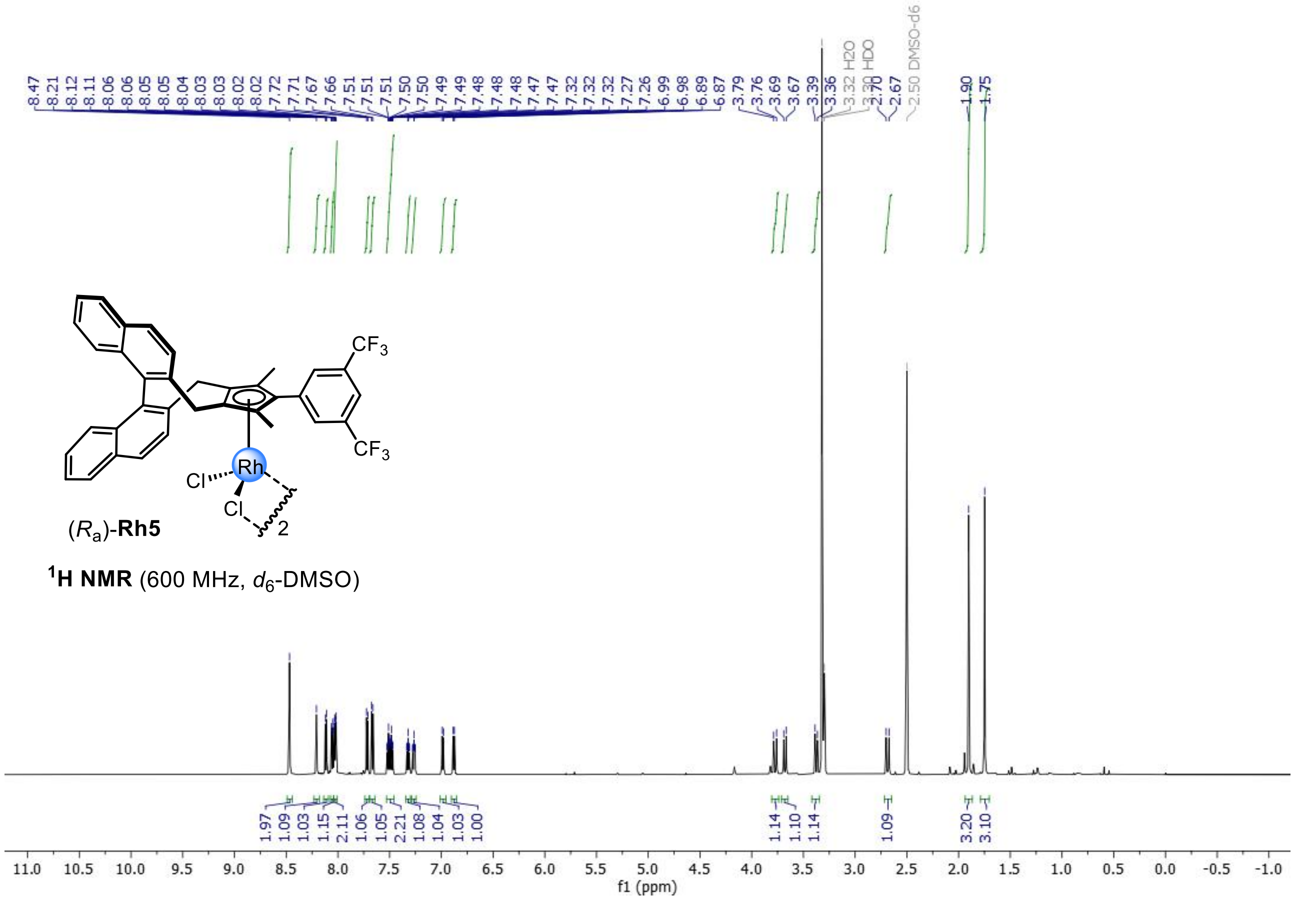
CF3
CF3
Rh
Cl
Cl
2
(Ra)-Rh5
1H NMR (600 MHz, d6-DMSO)
f1 (ppm)

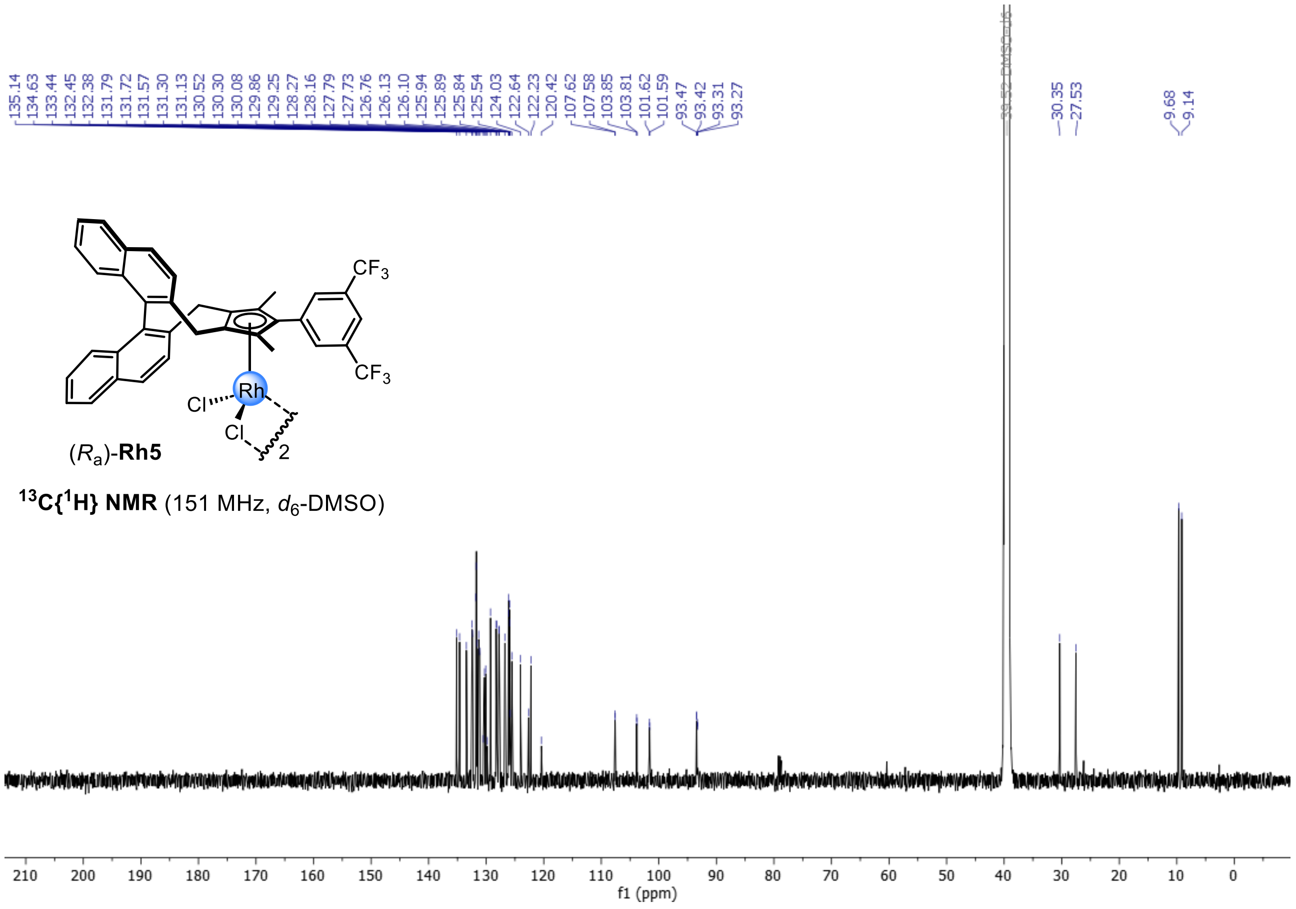
CF3
CF3
Rh
Cl
Cl
2
(Ra)-Rh5
13C{1H} NMR (151 MHz, d6-DMSO)
f1 (ppm)

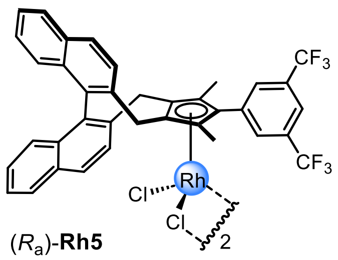

(R_a)-**Rh5**

^{19}F{^{1}H} NMR (376 MHz, d_6-DMSO)

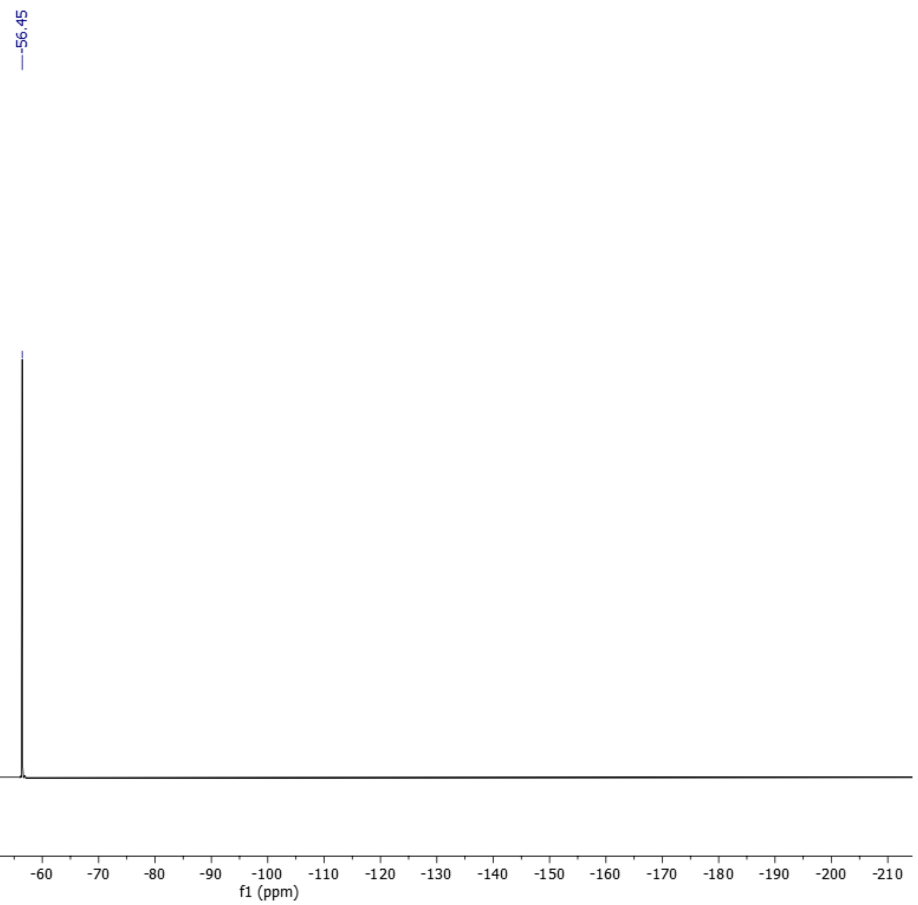

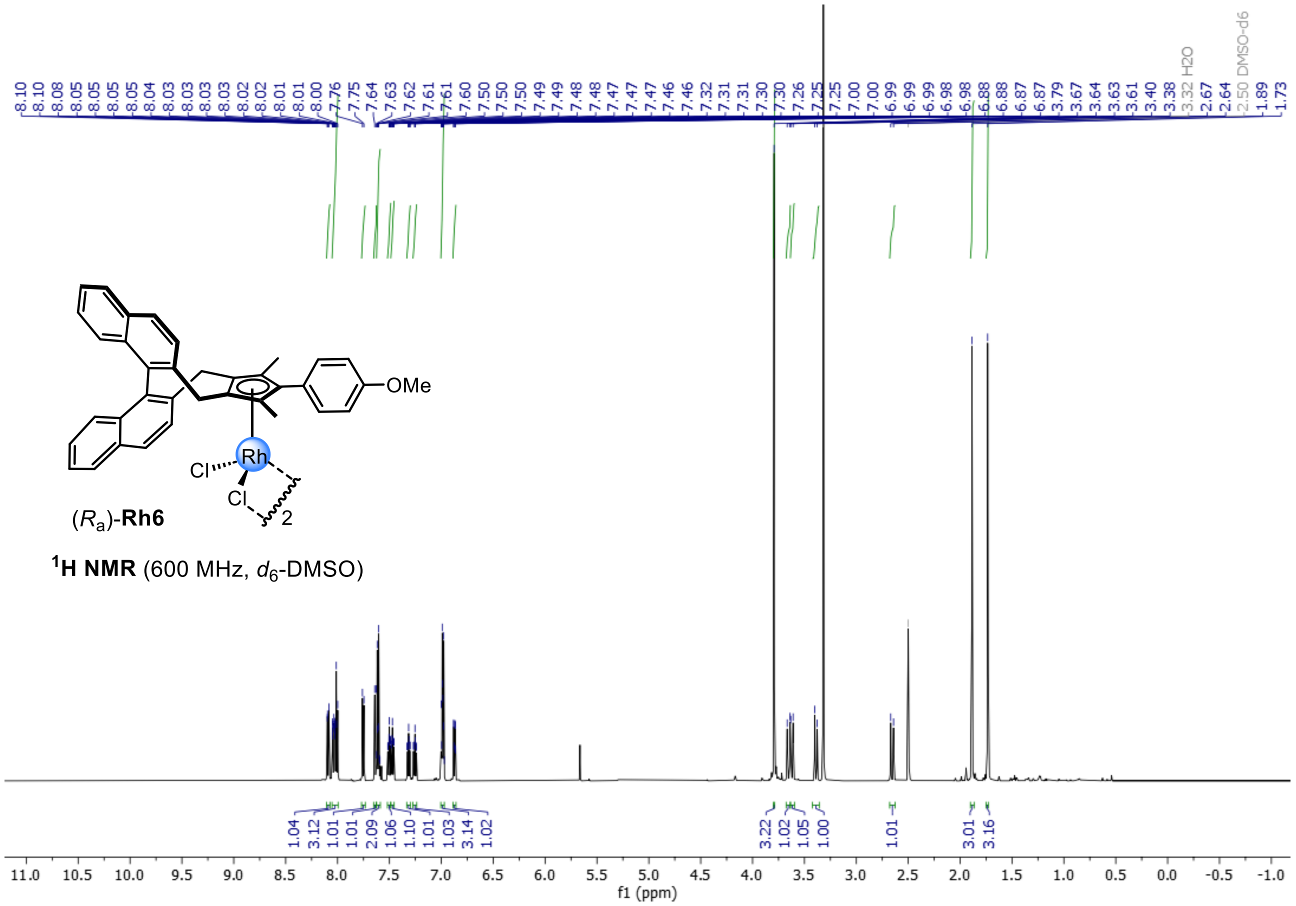
OMe
Rh
Cl
Cl
2
(Ra)-Rh6
1H NMR (600 MHz, d6-DMSO)
3.32 H2O
2.50 DMSO-d6
f1 (ppm)

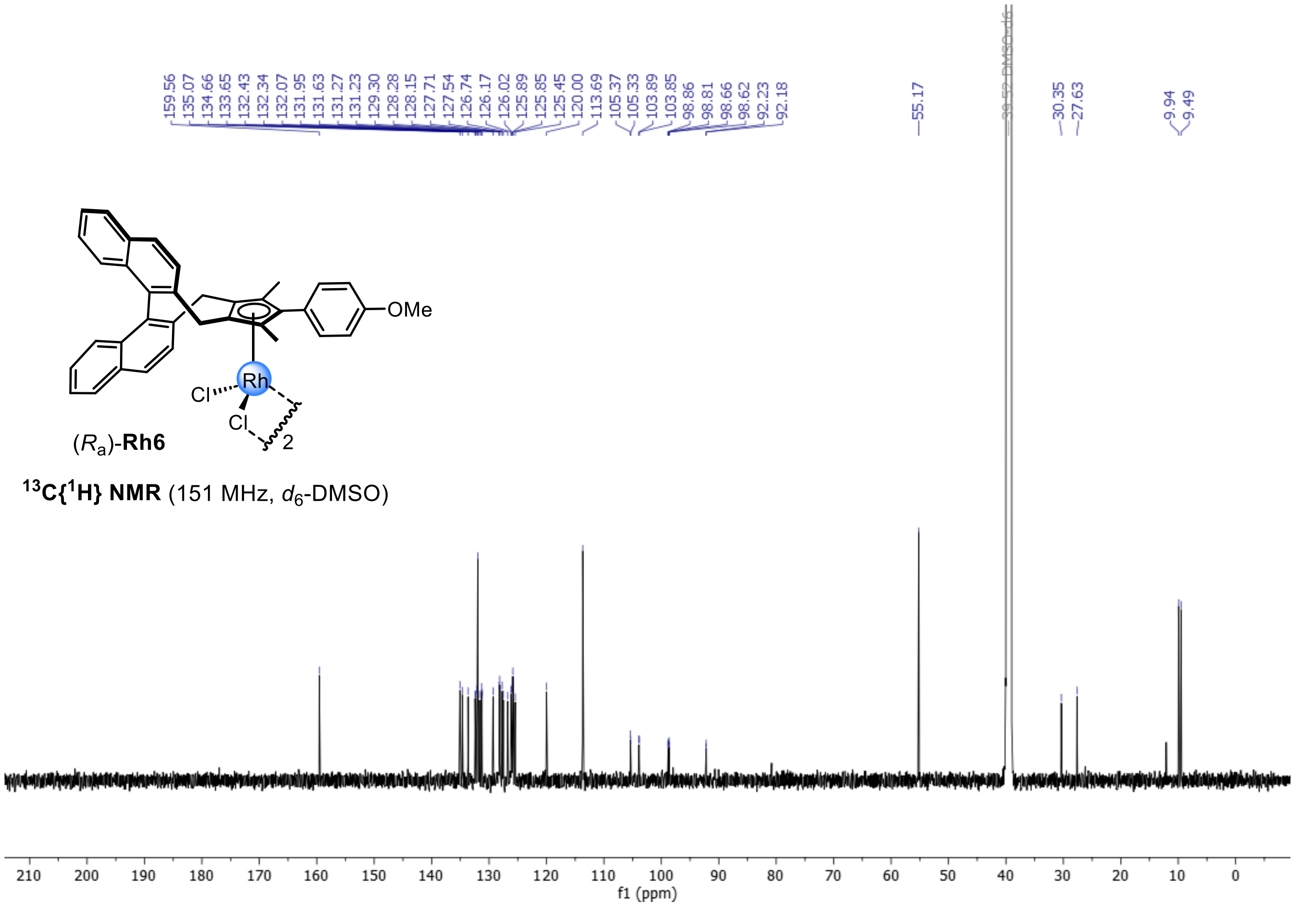
OMe
Rh
Cl
Cl
2
(Ra)-Rh6
13C{1H} NMR (151 MHz, d6-DMSO)
f1 (ppm)

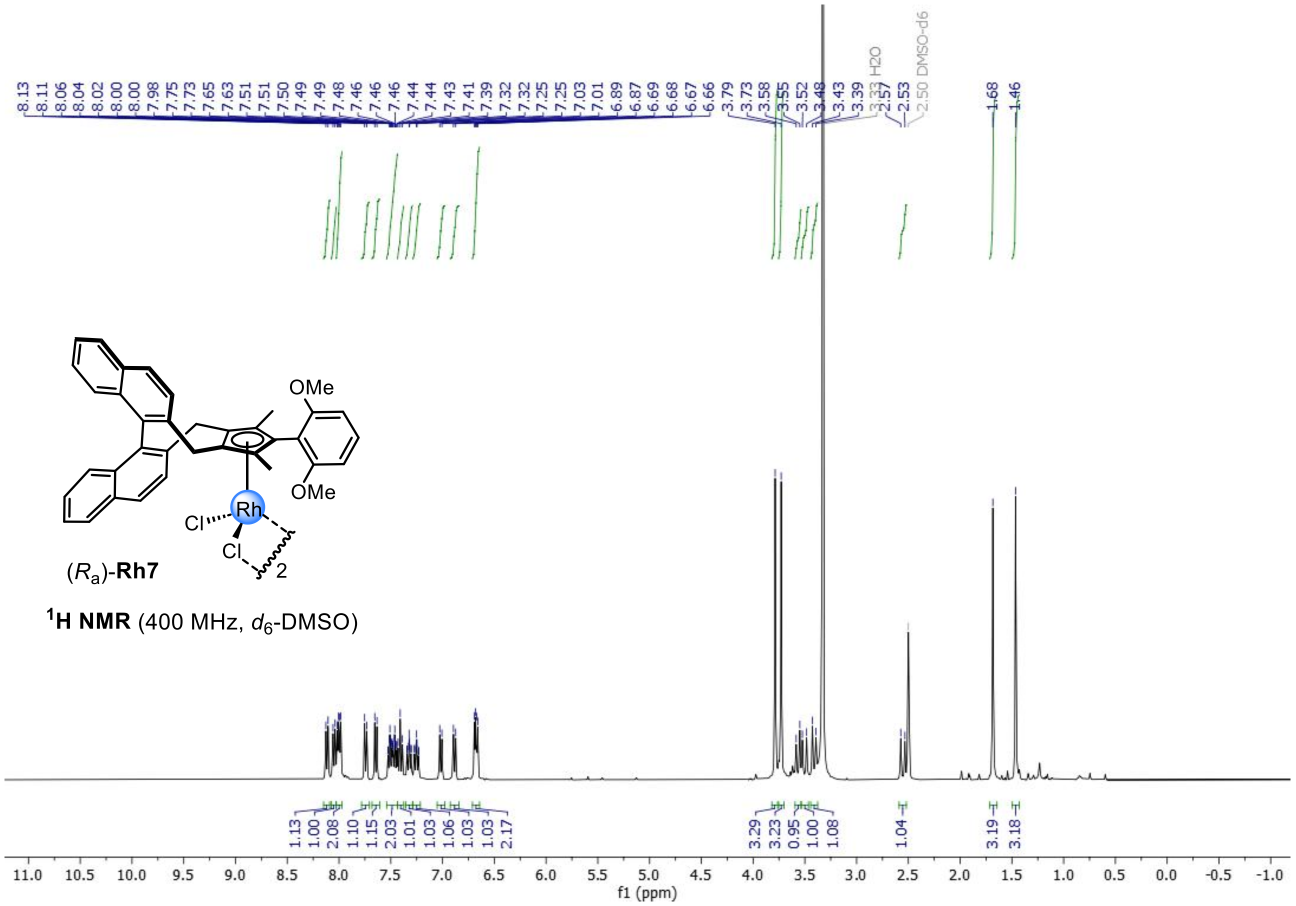
OMe
OMe
Rh
Cl
Cl
2
(Ra)-Rh7
1H NMR (400 MHz, d6-DMSO)
3.33 H2O
2.50 DMSO-d6
f1 (ppm)

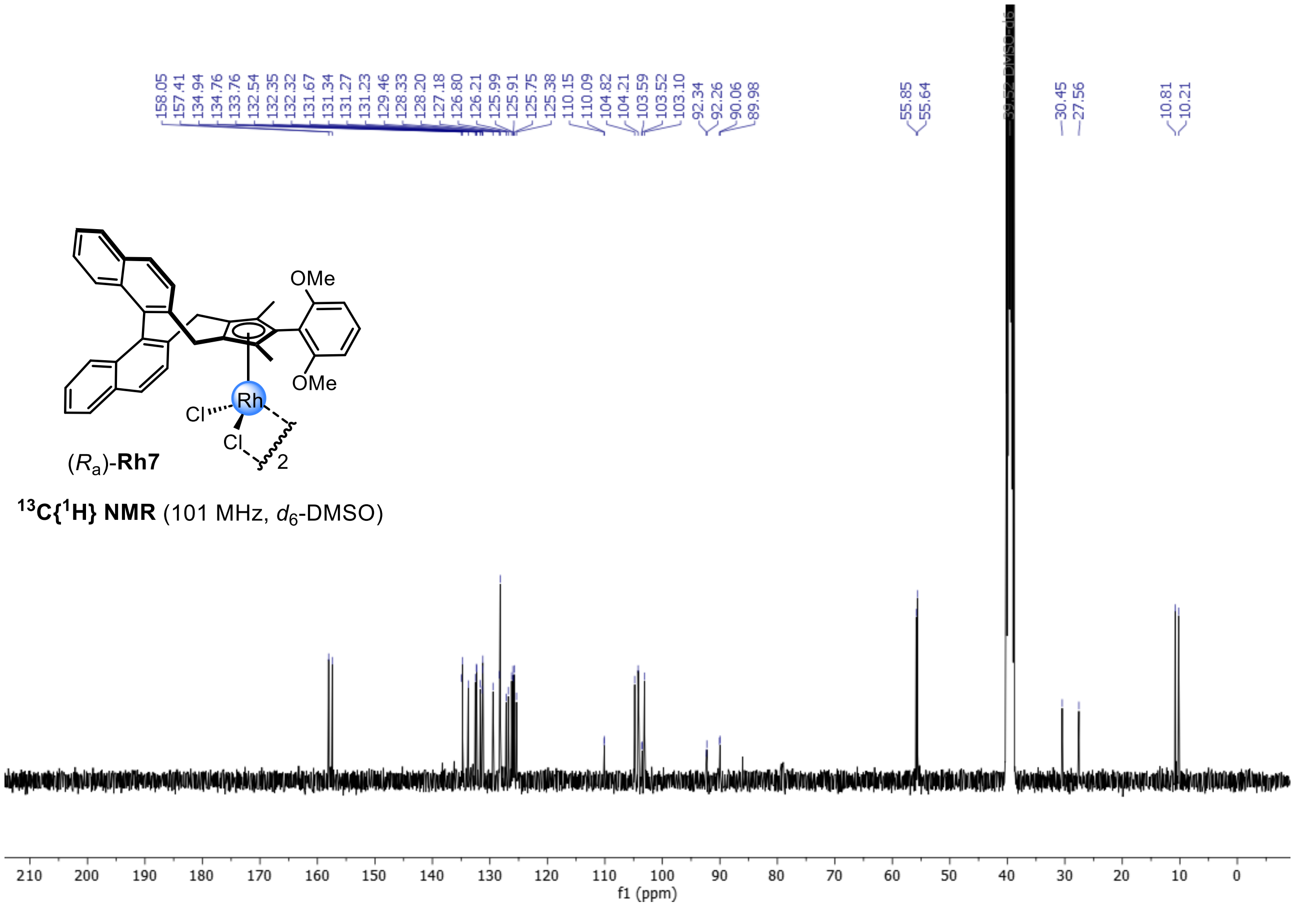
OMe
OMe
Rh
Cl
Cl
2
(Ra)-Rh7
13C{1H} NMR (101 MHz, d6-DMSO)
39.52 DMSO-d6
f1 (ppm)

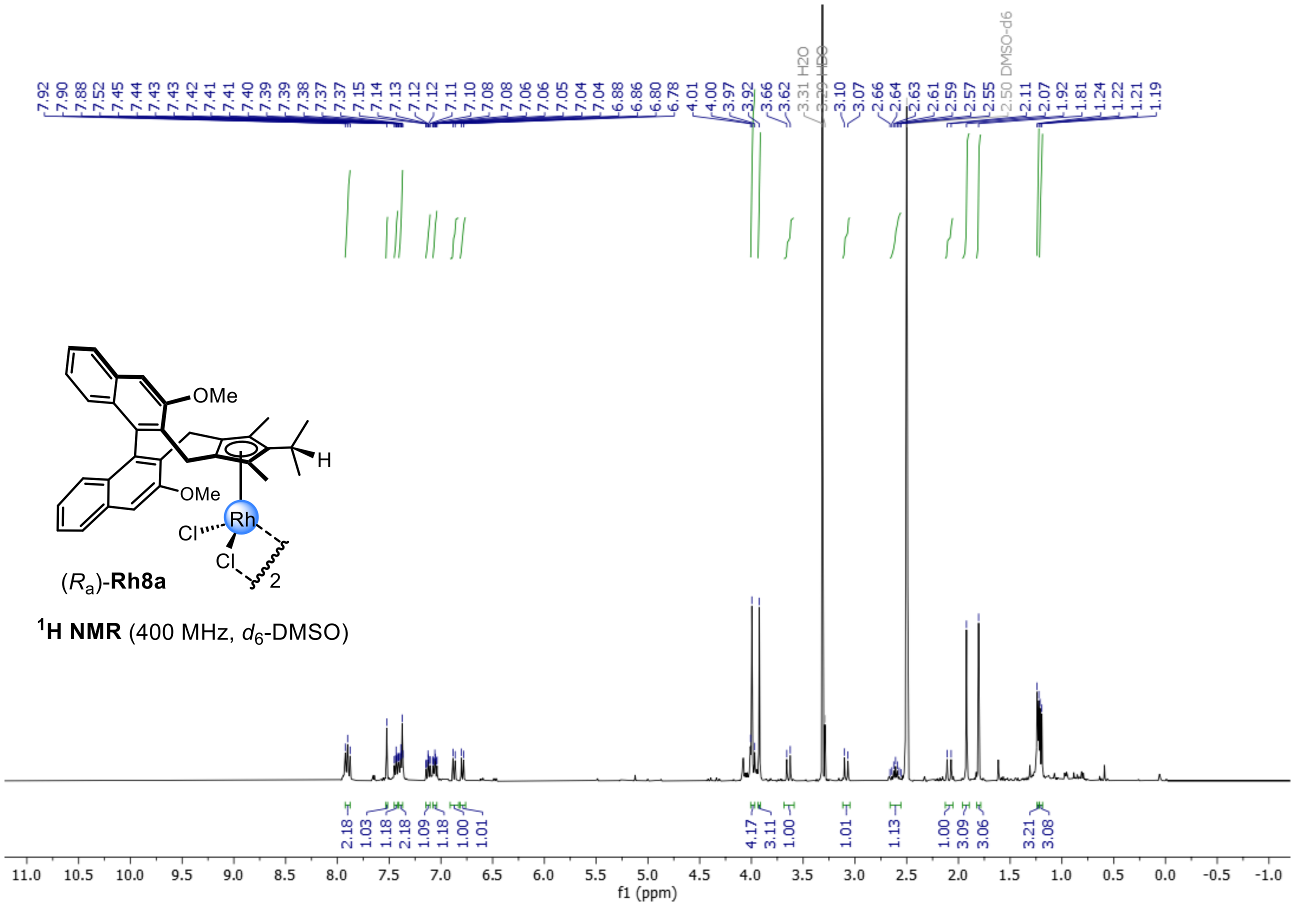

(R_a)-**Rh8a**

^{1}H NMR (400 MHz, d_6-DMSO)

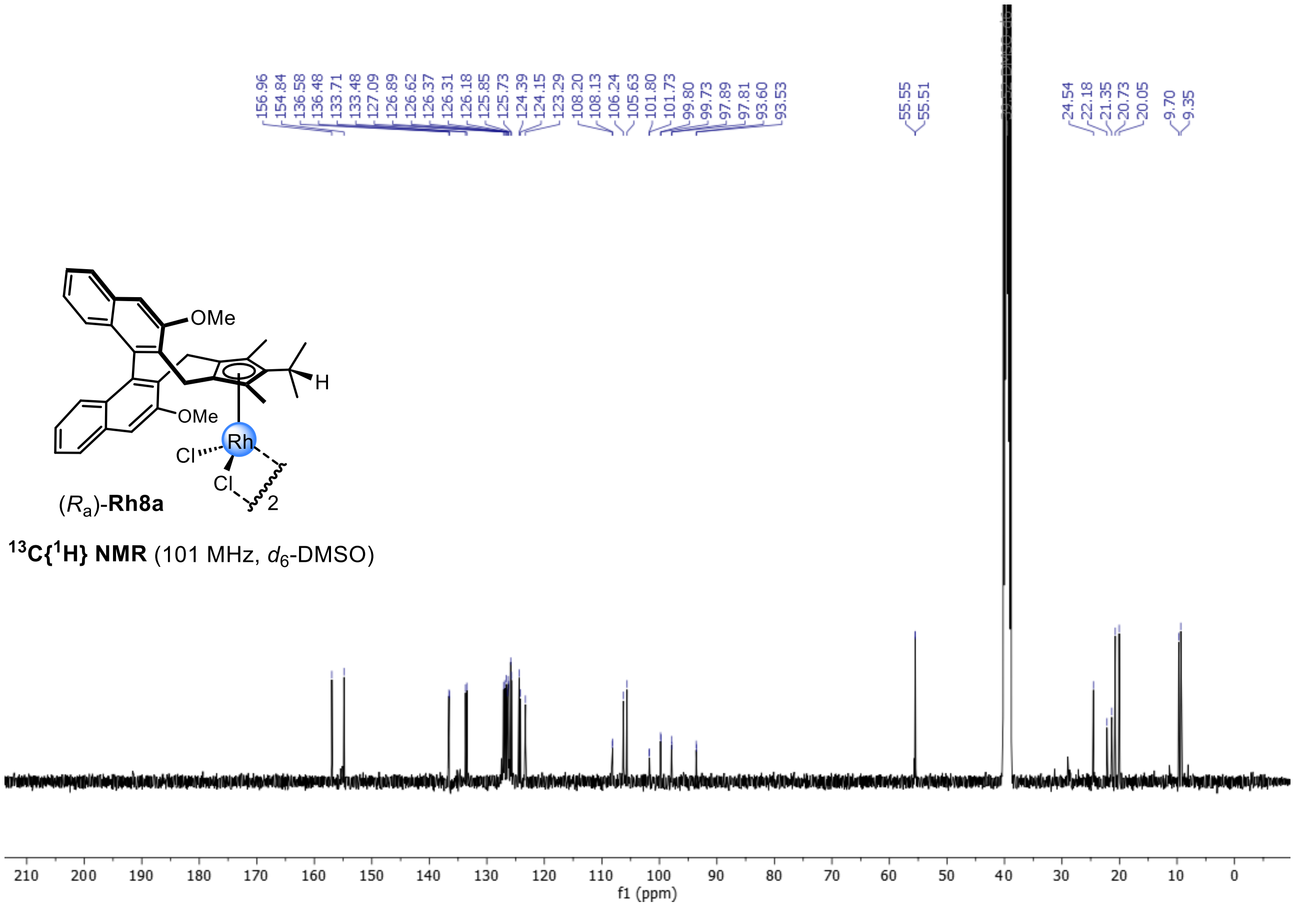

(R_a)-**Rh8a**

^{13}C{^{1}H} NMR (101 MHz, d_6-DMSO)

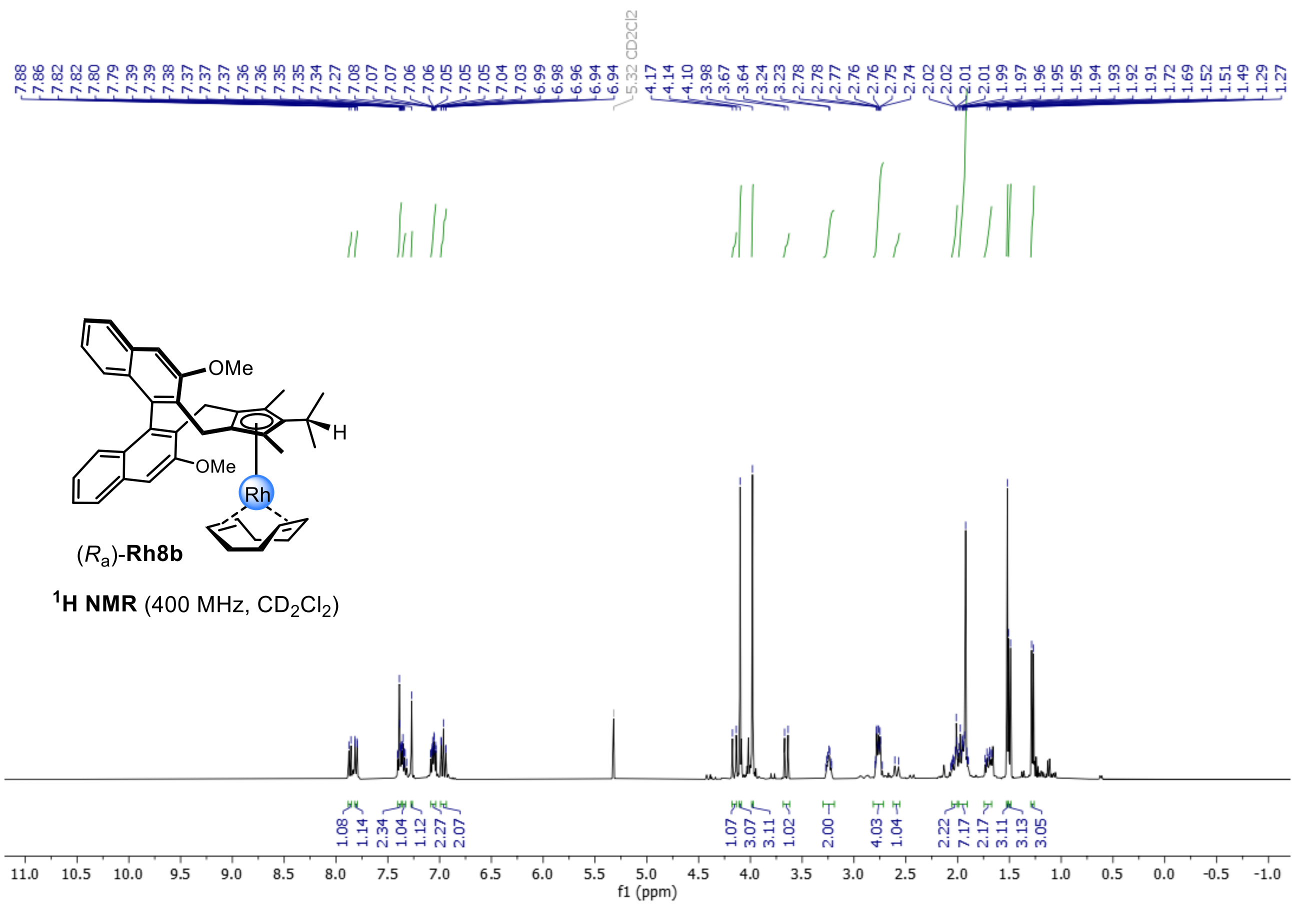
OMe
OMe
H
Rh
(R_a)-**Rh8b**
^{1}H NMR (400 MHz, CD_2Cl_2)
f1 (ppm)

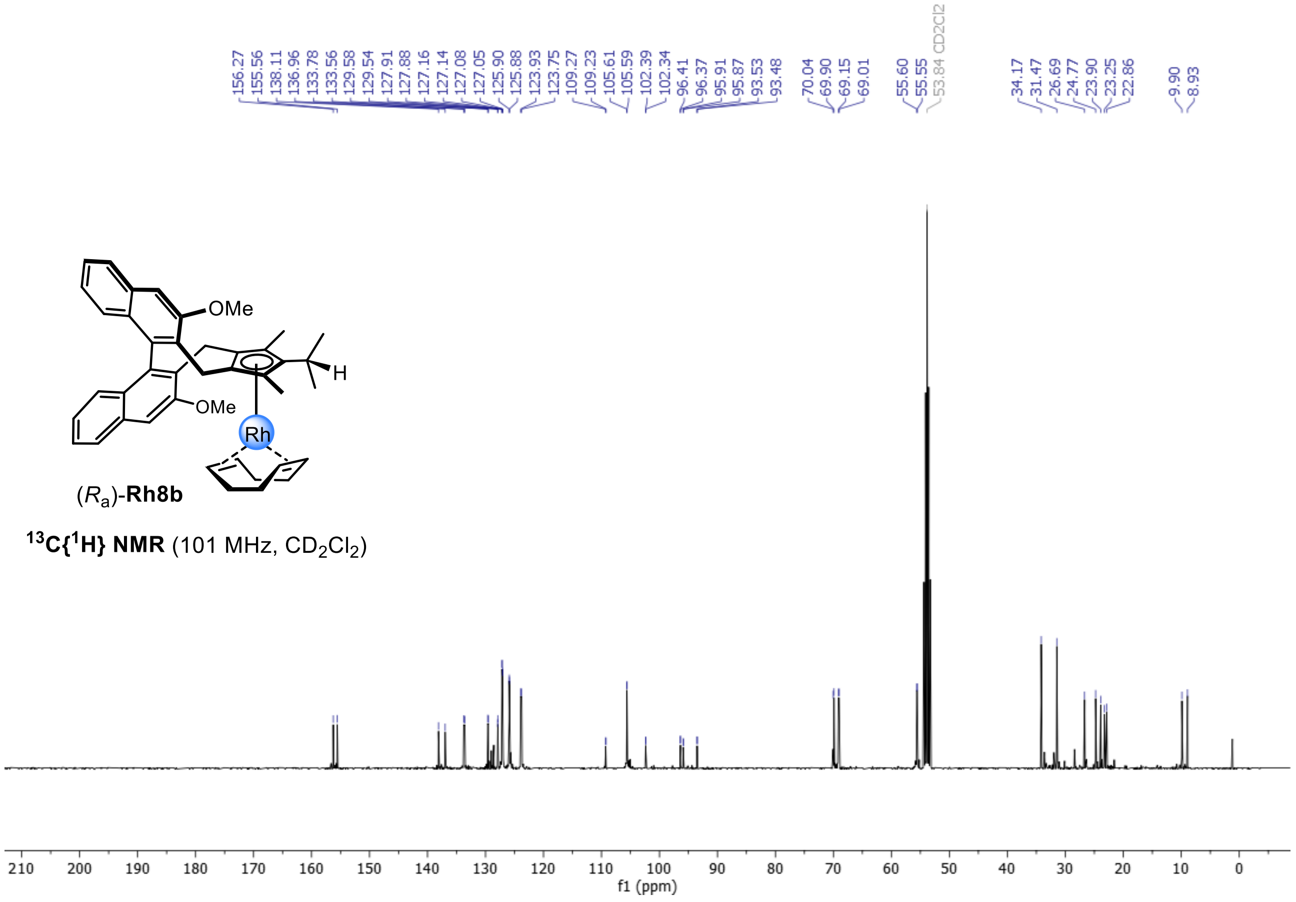
OMe
OMe
H
Rh
(R_a)-**Rh8b**
^{13}C{^{1}H} NMR (101 MHz, CD_2Cl_2)
f1 (ppm)

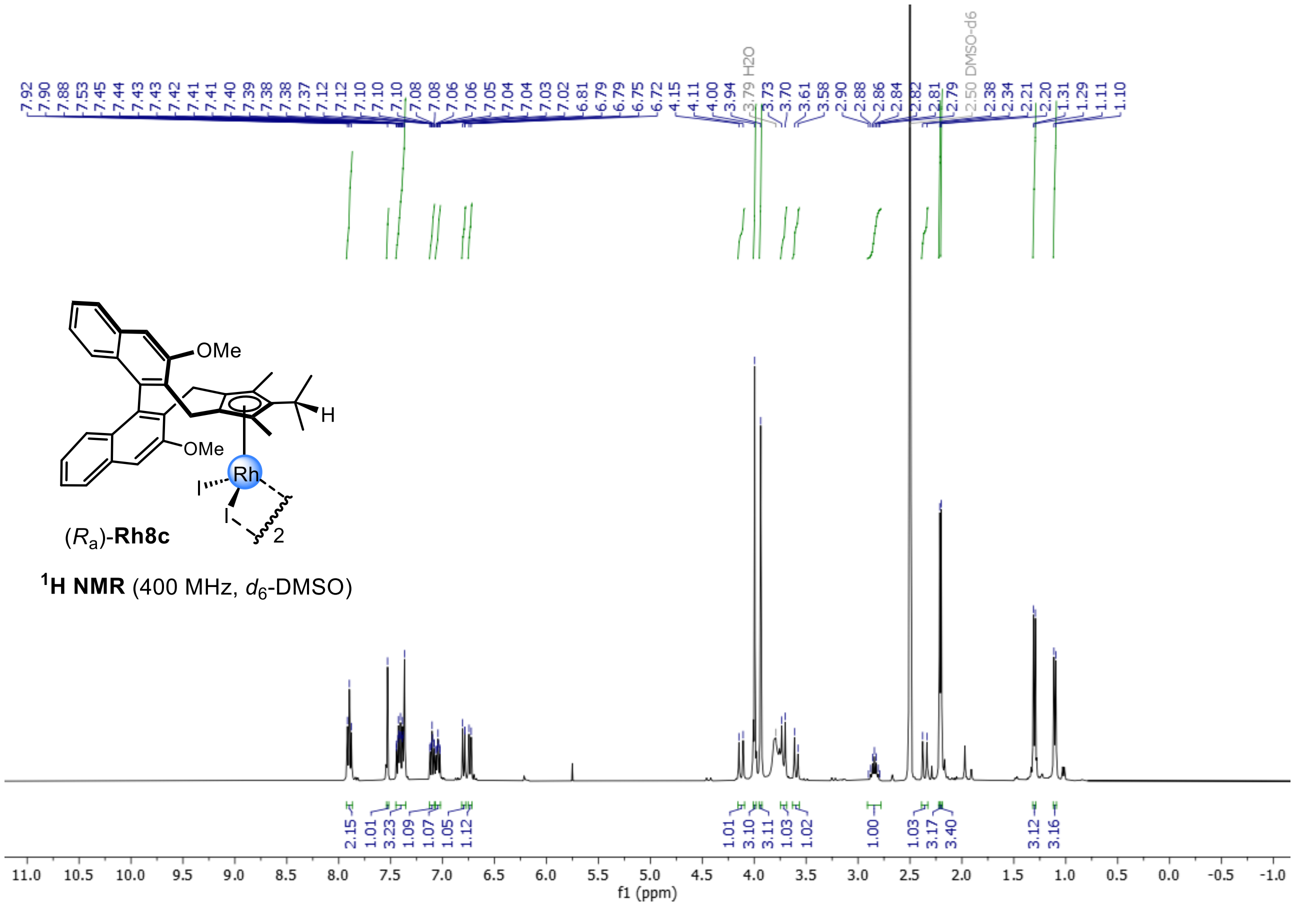
7.92 7.90 7.88 7.53 7.45 7.44 7.43 7.43 7.42 7.41 7.41 7.40 7.39 7.38 7.38 7.37 7.12 7.10 7.10 7.08 7.08 7.06 7.06 7.05 7.04 7.04 7.03 7.02 6.81 6.79 6.79 6.75 6.72 4.15 4.11 4.00 3.94 3.79 H2O 3.73 3.70 3.61 3.58 2.90 2.88 2.86 2.84 2.82 2.81 2.79 2.50 DMSO-d6 2.38 2.34 2.21 2.20 1.31 1.29 1.11 1.10
OMe
OMe
H
Rh
I
I
2
(Ra)-Rh8c
1H NMR (400 MHz, d6-DMSO)
2.15 1.01 3.23 1.09 1.07 1.05 1.12 1.01 3.10 3.11 1.03 1.02 1.00 1.03 3.17 3.40 3.12 3.16
11.0 10.5 10.0 9.5 9.0 8.5 8.0 7.5 7.0 6.5 6.0 5.5 5.0 4.5 4.0 3.5 3.0 2.5 2.0 1.5 1.0 0.5 0.0 -0.5 -1.0
f1 (ppm)

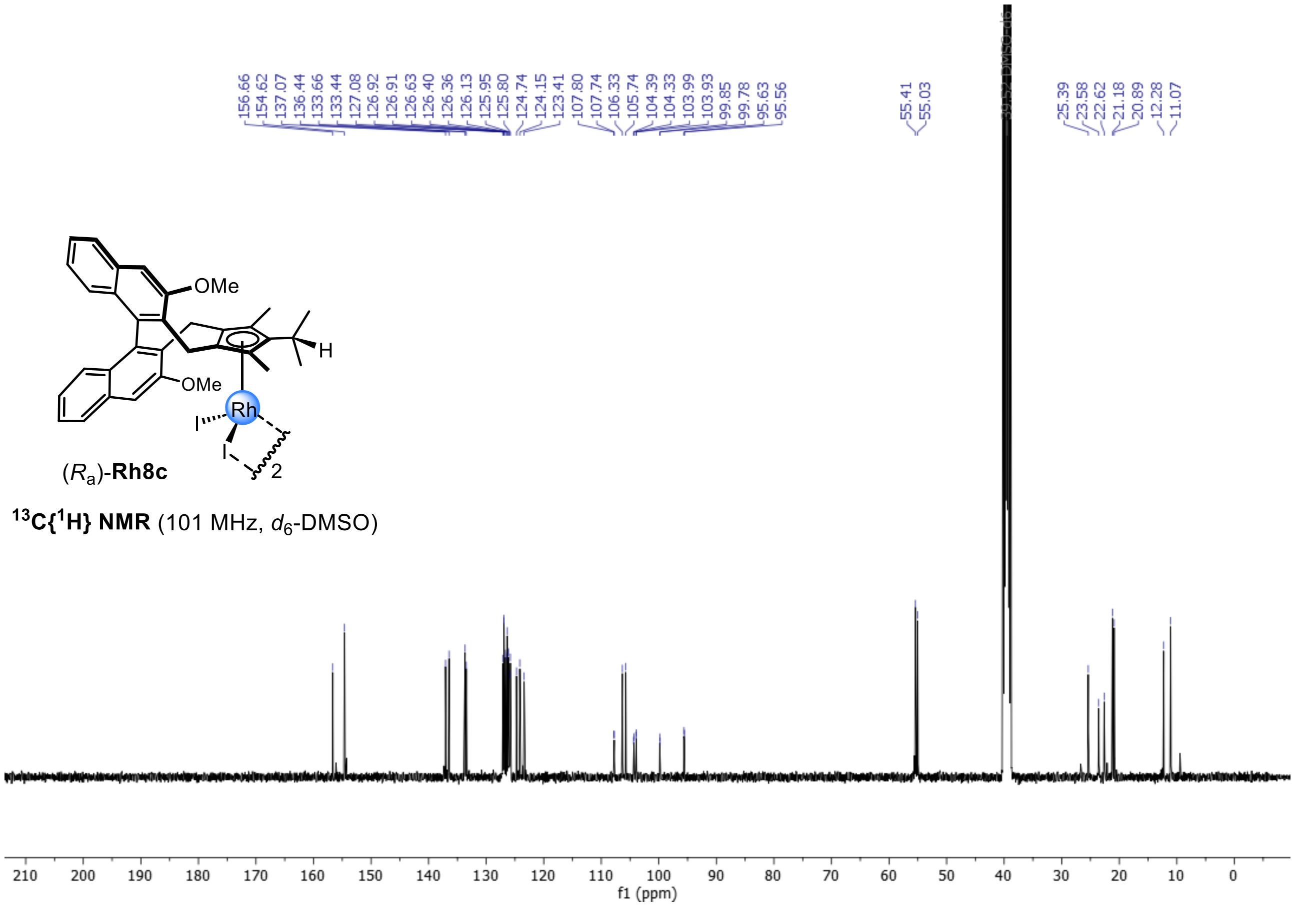
156.66 154.62 137.07 136.44 133.66 133.44 127.08 126.92 126.91 126.63 126.40 126.36 126.13 125.95 125.80 124.74 124.15 123.41 107.80 107.74 106.33 105.74 104.39 104.33 103.99 103.93 99.85 99.78 95.63 95.56
55.41 55.03
25.39 23.58 22.62 21.18 20.89 12.28 11.07
OMe
OMe
H
Rh
I
I
2
(Ra)-Rh8c
13C{1H} NMR (101 MHz, d6-DMSO)
210 200 190 180 170 160 150 140 130 120 110 100 90 80 70 60 50 40 30 20 10 0
f1 (ppm)

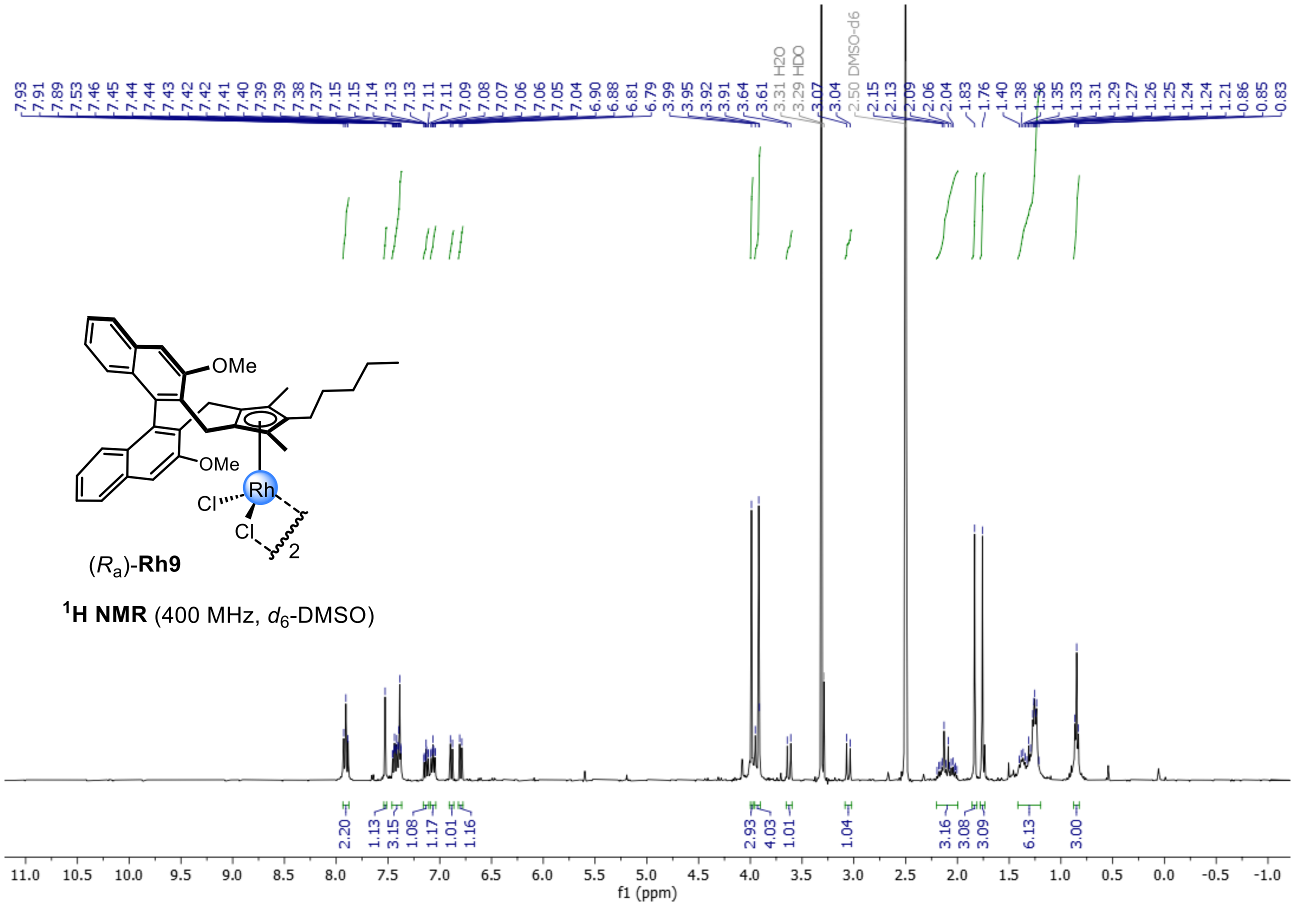

(R_a)-**Rh9**

^{1}H NMR (400 MHz, d_6-DMSO)

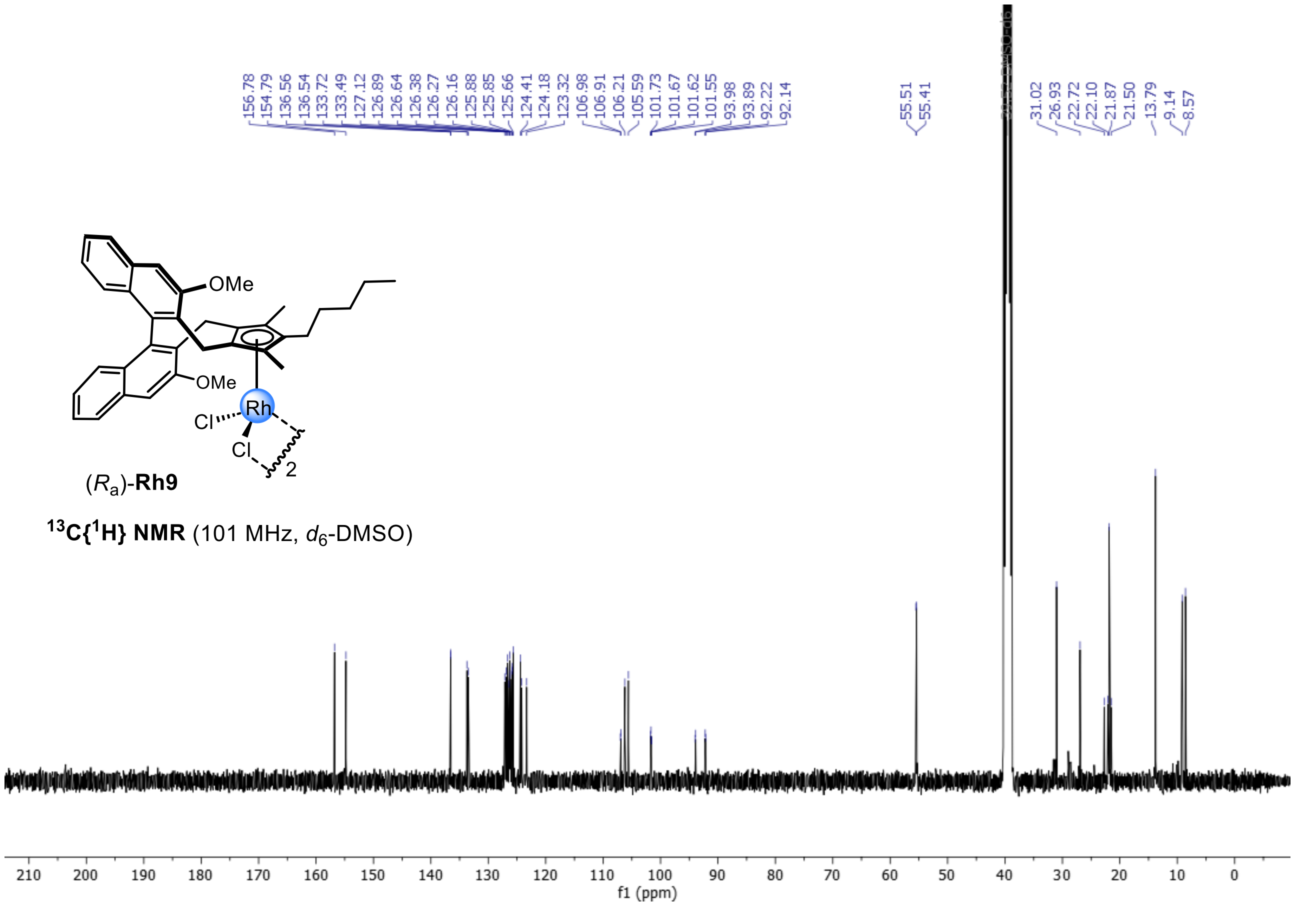

(R_a)-**Rh9**

^{13}C{^{1}H} NMR (101 MHz, d_6-DMSO)

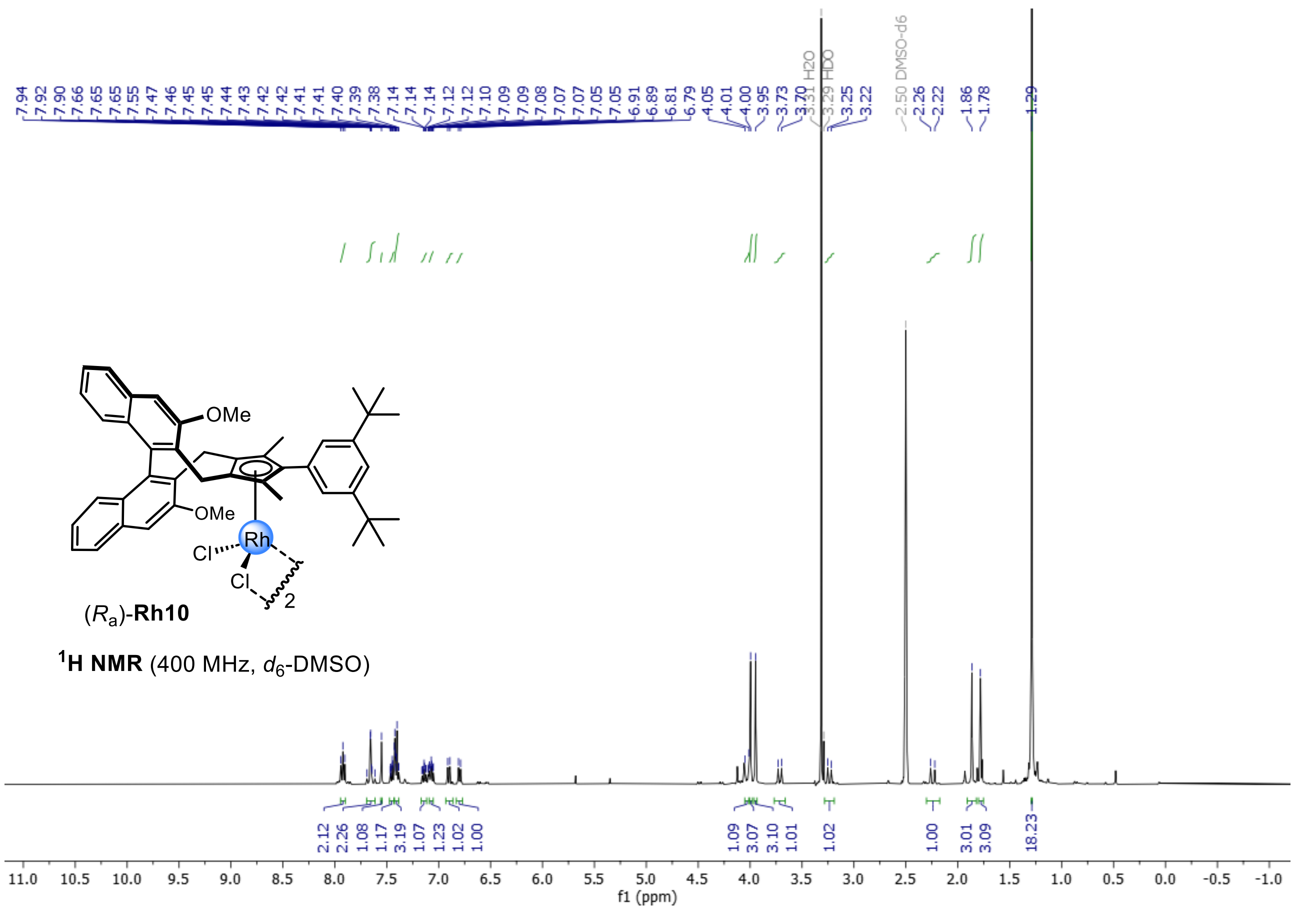
7.94 7.92 7.90 7.66 7.65 7.55 7.47 7.46 7.45 7.45 7.44 7.43 7.42 7.42 7.41 7.41 7.40 7.39 7.38 7.14 7.14 7.14 7.12 7.12 7.10 7.09 7.09 7.08 7.07 7.07 7.05 7.05 6.91 6.89 6.81 6.79 4.05 4.01 4.00 3.95 3.73 3.70 3.31 H2O 3.29 HDO 3.25 3.22 2.50 DMSO-d6 2.26 2.22 1.86 1.78 1.29
OMe
OMe
Rh
Cl
Cl
2
(Ra)-Rh10
1H NMR (400 MHz, d6-DMSO)
2.12 2.26 1.08 1.17 3.19 1.07 1.23 1.02 1.00 1.09 3.07 3.10 1.01 1.02 1.00 3.01 3.09 18.23
f1 (ppm)

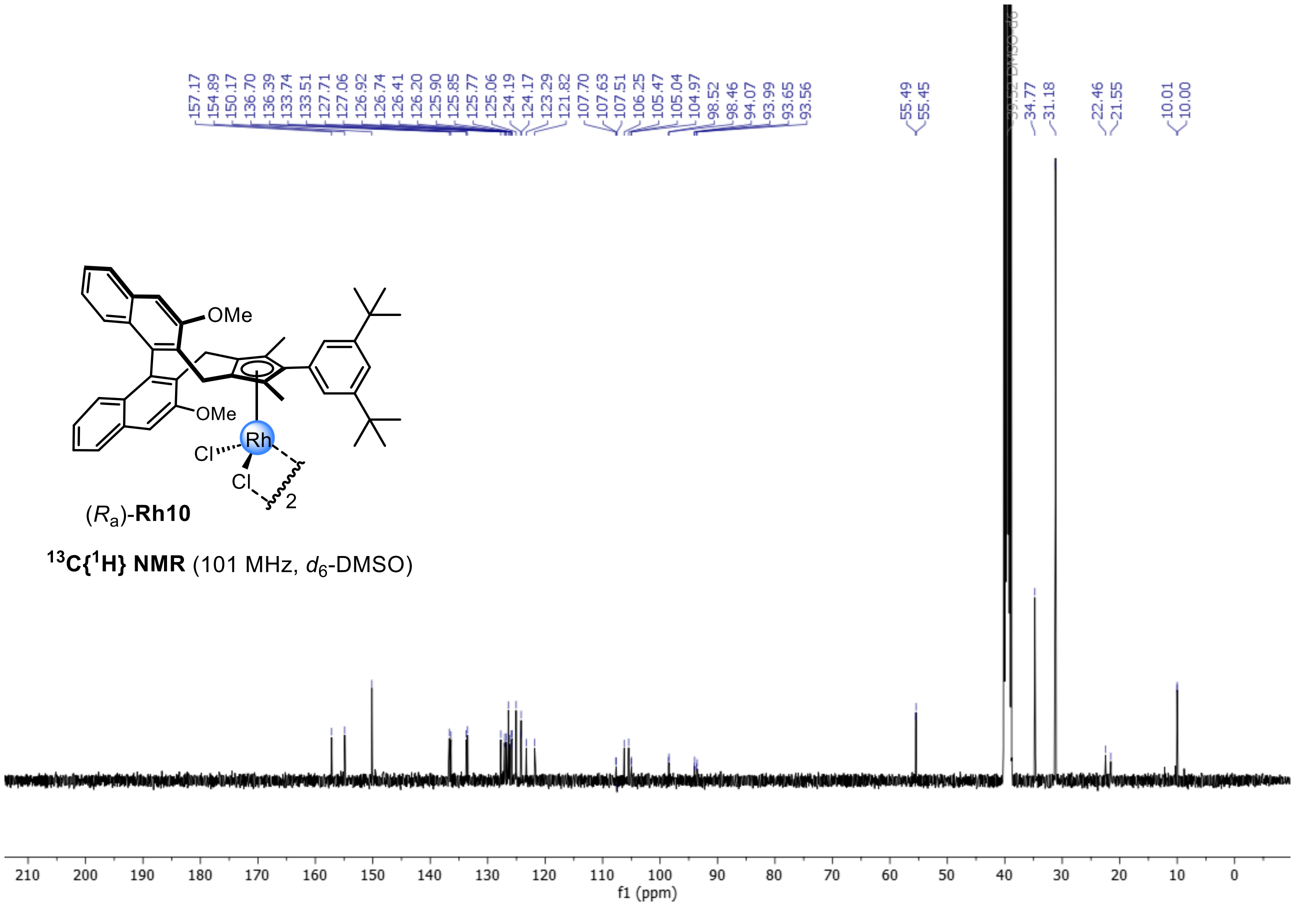
157.17 154.89 150.17 136.70 136.39 133.74 133.51 127.71 127.06 126.92 126.74 126.41 126.20 125.90 125.85 125.77 125.06 124.19 124.17 123.29 121.82 107.70 107.63 107.51 106.25 105.47 105.04 104.97 98.52 98.46 94.07 93.99 93.65 93.56 55.49 55.45 39.52 DMSO-d6 34.77 31.18 22.46 21.55 10.01 10.00
OMe
OMe
Rh
Cl
Cl
2
(Ra)-Rh10
13C{1H} NMR (101 MHz, d6-DMSO)
f1 (ppm)

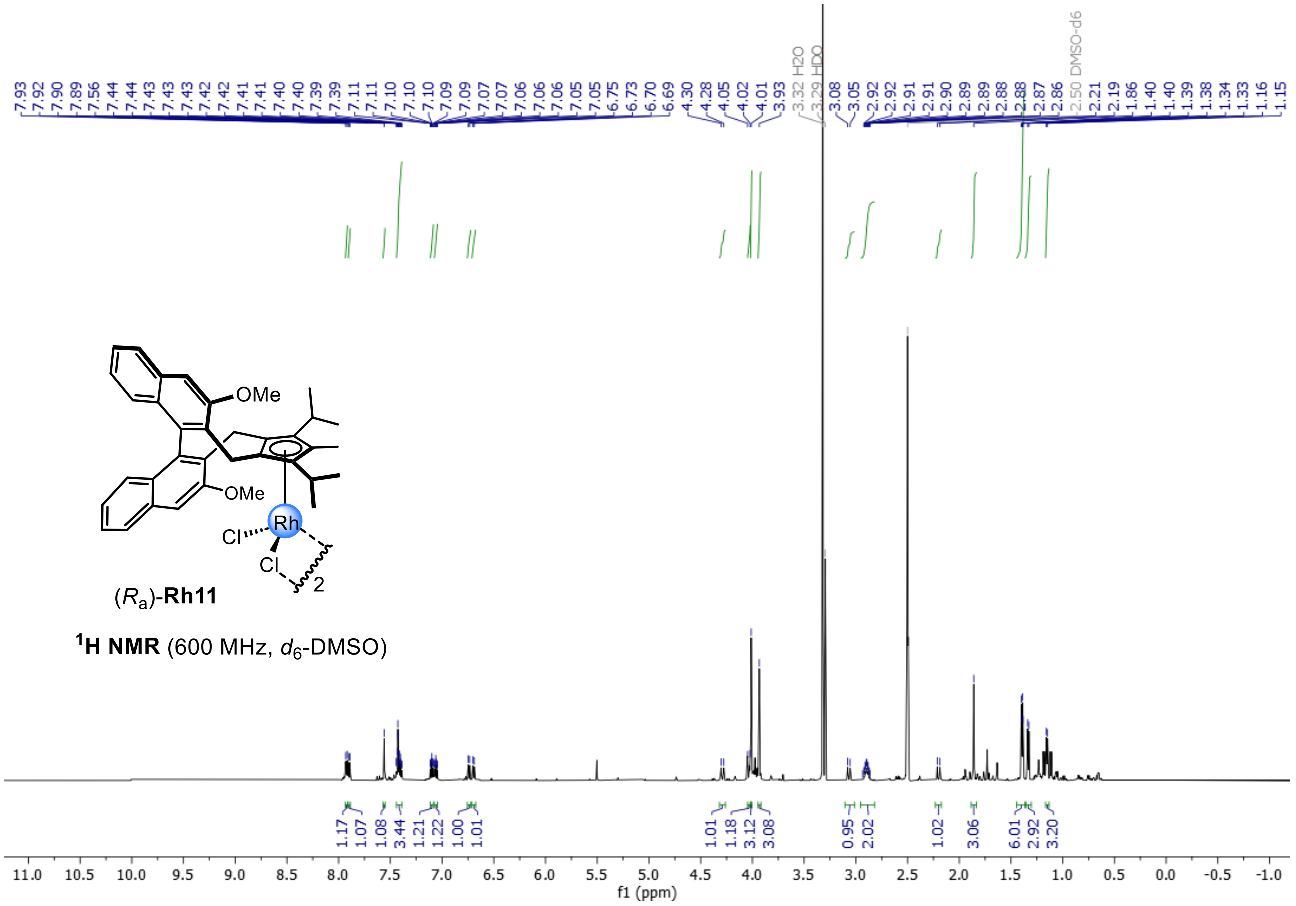
7.93 7.92 7.90 7.89 7.56 7.44 7.44 7.43 7.43 7.43 7.42 7.42 7.41 7.41 7.40 7.40 7.39 7.39 7.11 7.11 7.10 7.10 7.09 7.09 7.07 7.07 7.06 7.06 7.06 7.05 7.05 6.75 6.73 6.70 6.69 4.30 4.28 4.05 4.02 4.01 3.93 3.32 H2O 3.29 HDO 3.08 3.05 2.92 2.92 2.91 2.91 2.90 2.89 2.89 2.88 2.88 2.87 2.86 2.50 DMSO-d6 2.21 2.19 1.86 1.40 1.40 1.39 1.38 1.34 1.33 1.16 1.15
OMe
OMe
Rh
Cl
Cl
2
(R_a)-**Rh11**
^{1}H NMR (600 MHz, d_6-DMSO)
1.17 1.07 1.08 3.44 1.21 1.22 1.00 1.01 1.01 1.18 3.12 3.08 0.95 2.02 1.02 3.06 6.01 2.92 3.20
f1 (ppm)

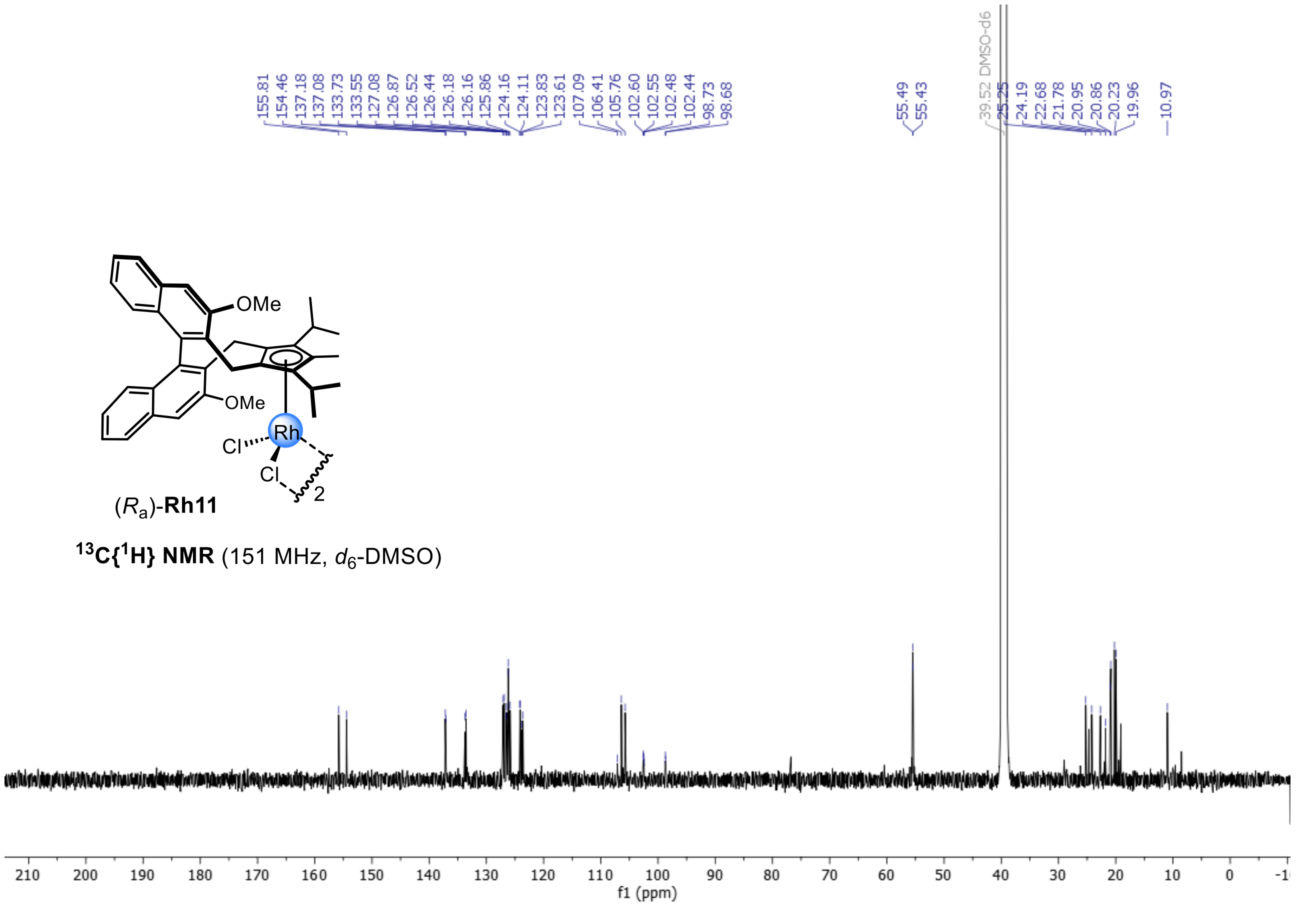
155.81 154.46 137.18 137.08 133.73 133.55 127.08 126.87 126.52 126.44 126.18 126.16 125.86 124.16 124.11 123.83 123.61 107.09 106.41 105.76 102.60 102.55 102.48 102.44 98.73 98.68 55.49 55.43 39.52 DMSO-d6 25.25 24.19 22.68 21.78 20.95 20.86 20.23 19.96 10.97
OMe
OMe
Rh
Cl
Cl
2
(R_a)-**Rh11**
^{13}C{^{1}H} NMR (151 MHz, d_6-DMSO)
f1 (ppm)

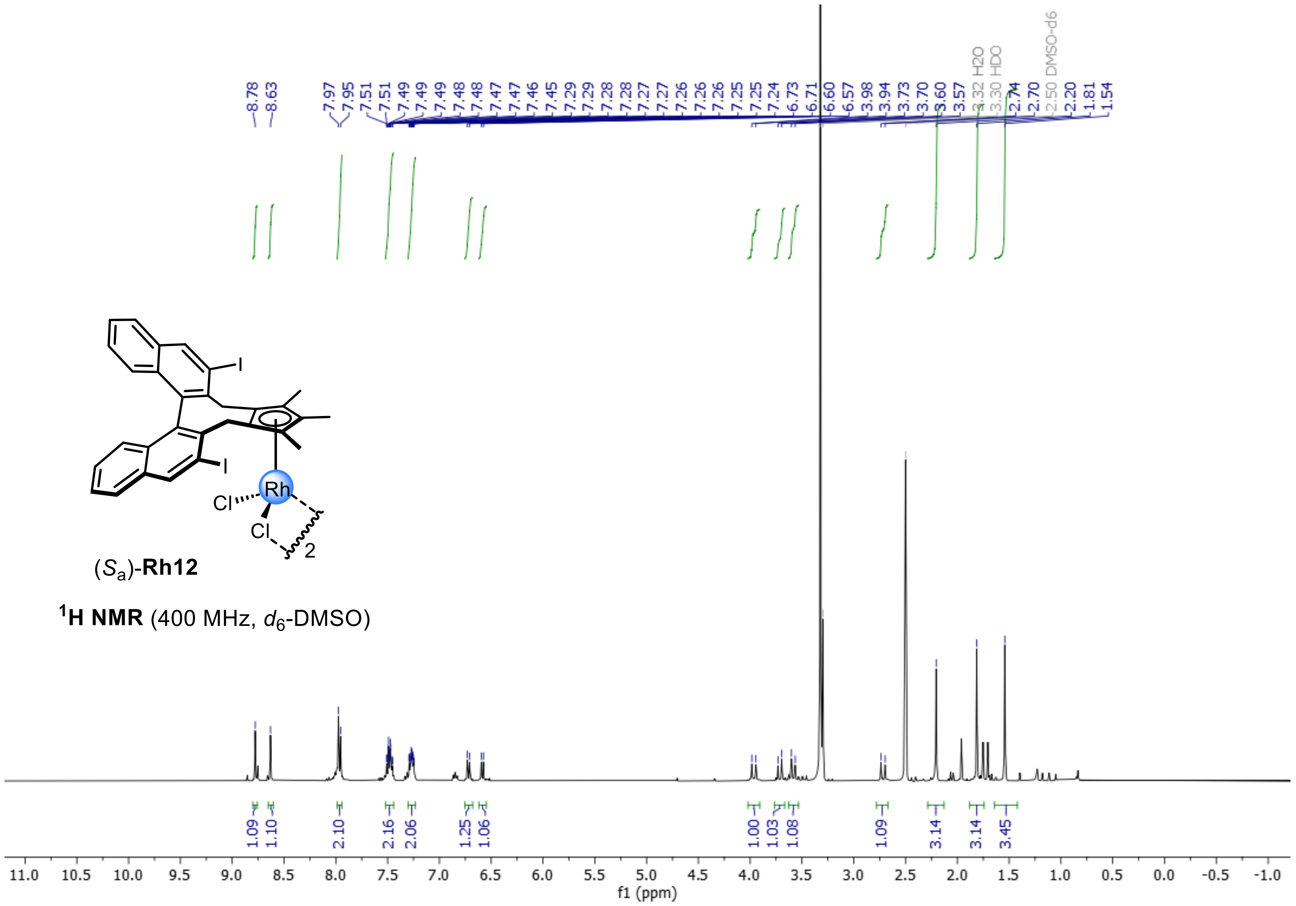
8.78
8.63
7.97
7.95
7.51
7.49
7.49
7.49
7.48
7.48
7.47
7.47
7.46
7.45
7.29
7.29
7.28
7.28
7.27
7.27
7.26
7.26
7.26
7.25
7.25
7.24
6.73
6.71
6.60
6.57
3.98
3.94
3.73
3.70
3.60
3.57
3.32 H2O
3.30 HDO
2.74
2.70
2.50 DMSO-d6
2.20
1.81
1.54
I
I
Rh
Cl
Cl
2
(S_a)-**Rh12**
^{1}H NMR (400 MHz, d_6-DMSO)
1.09
1.10
2.10
2.16
2.06
1.25
1.06
1.00
1.03
1.08
1.09
3.14
3.14
3.45
11.0 10.5 10.0 9.5 9.0 8.5 8.0 7.5 7.0 6.5 6.0 5.5 5.0 4.5 4.0 3.5 3.0 2.5 2.0 1.5 1.0 0.5 0.0 -0.5 -1.0
f1 (ppm)

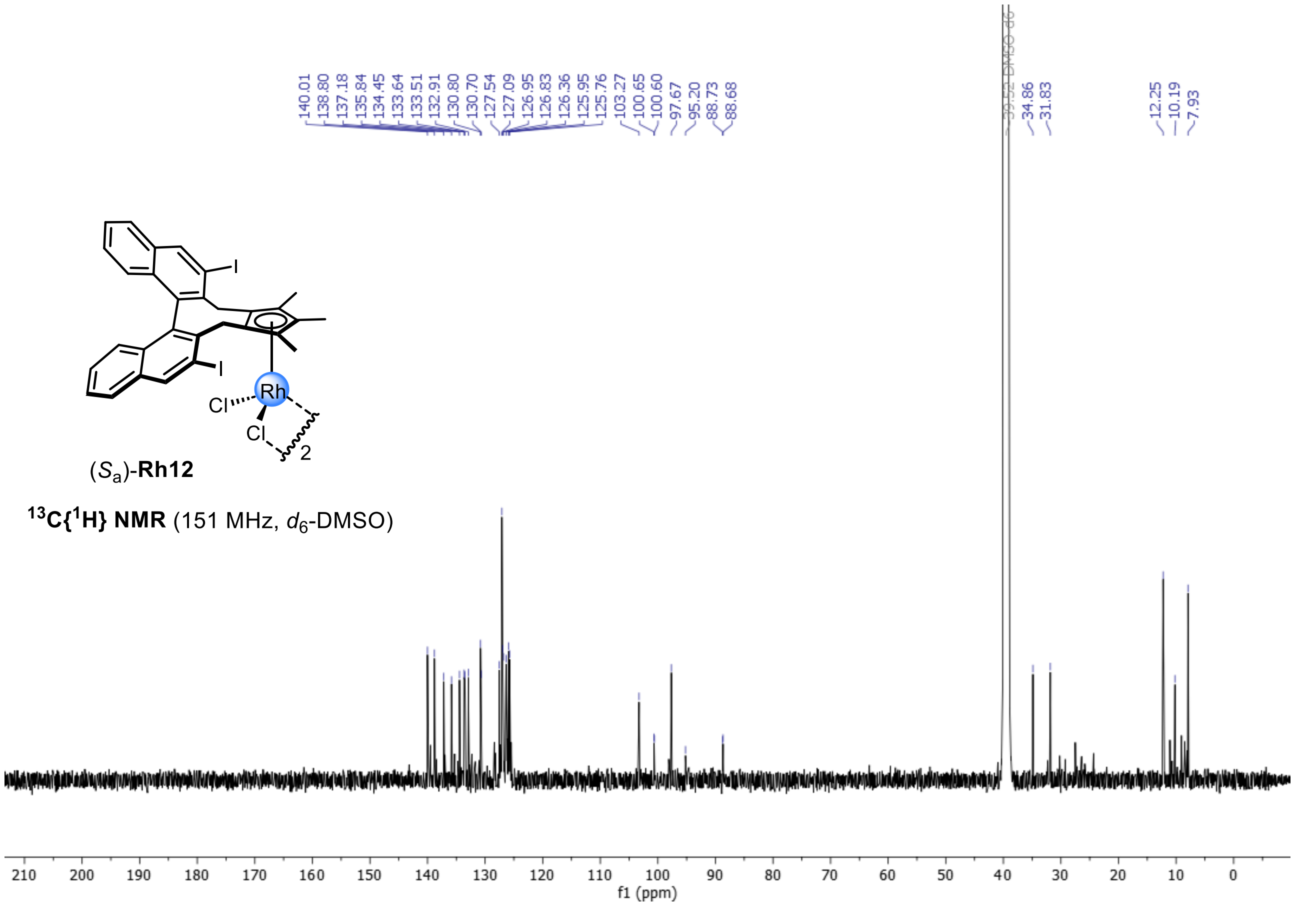
140.01
138.80
137.18
135.84
134.45
133.64
133.51
132.91
130.80
130.70
127.54
127.09
126.95
126.83
126.36
125.95
125.76
103.27
100.65
100.60
97.67
95.20
88.73
88.68
39.52 DMSO-d6
34.86
31.83
12.25
10.19
7.93
I
I
Rh
Cl
Cl
2
(S_a)-**Rh12**
^{13}C{^{1}H} NMR (151 MHz, d_6-DMSO)
210 200 190 180 170 160 150 140 130 120 110 100 90 80 70 60 50 40 30 20 10 0
f1 (ppm)

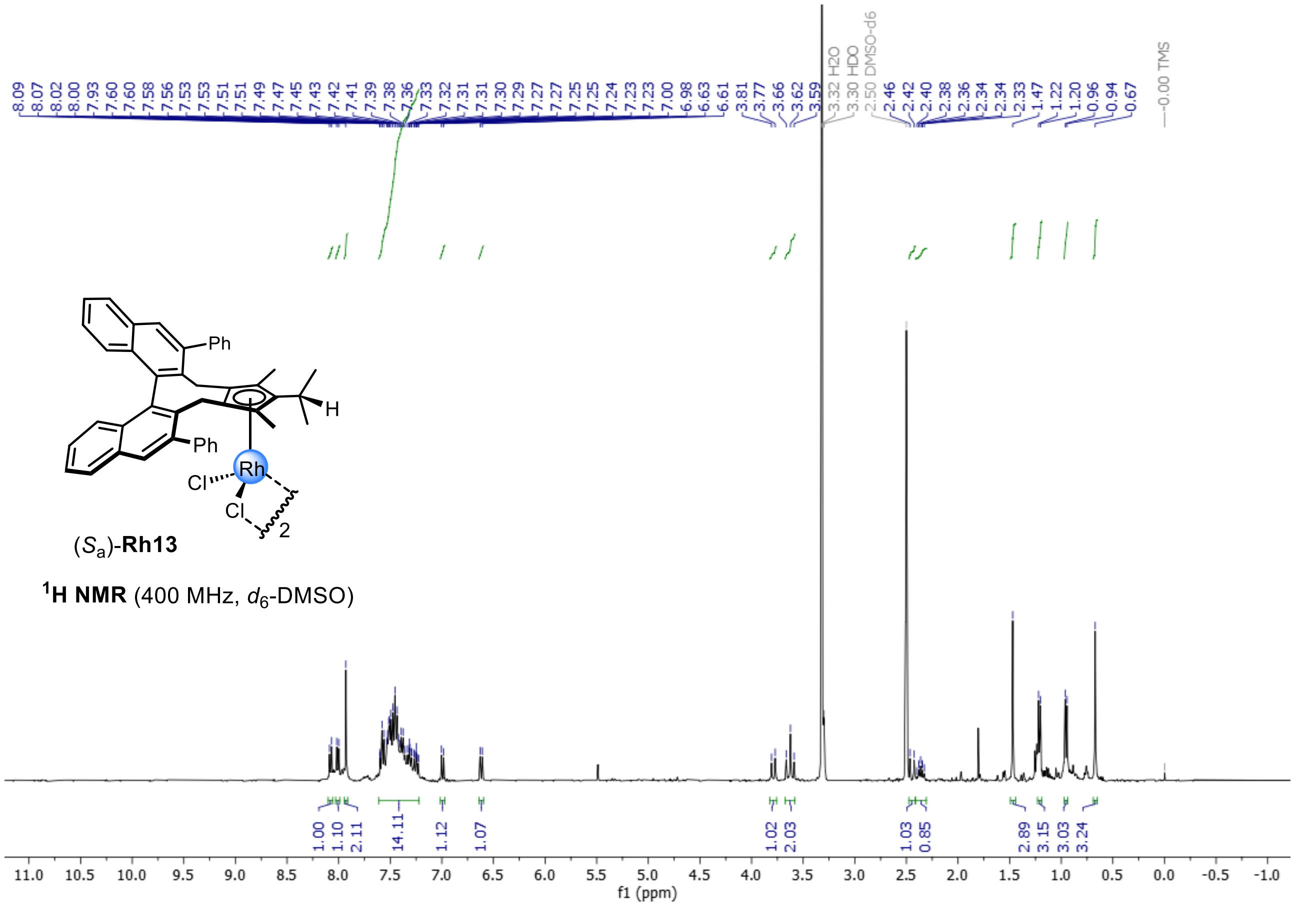
(Sa)-Rh13
1H NMR (400 MHz, d6-DMSO)
Ph
H
Rh
Cl
2
f1 (ppm)

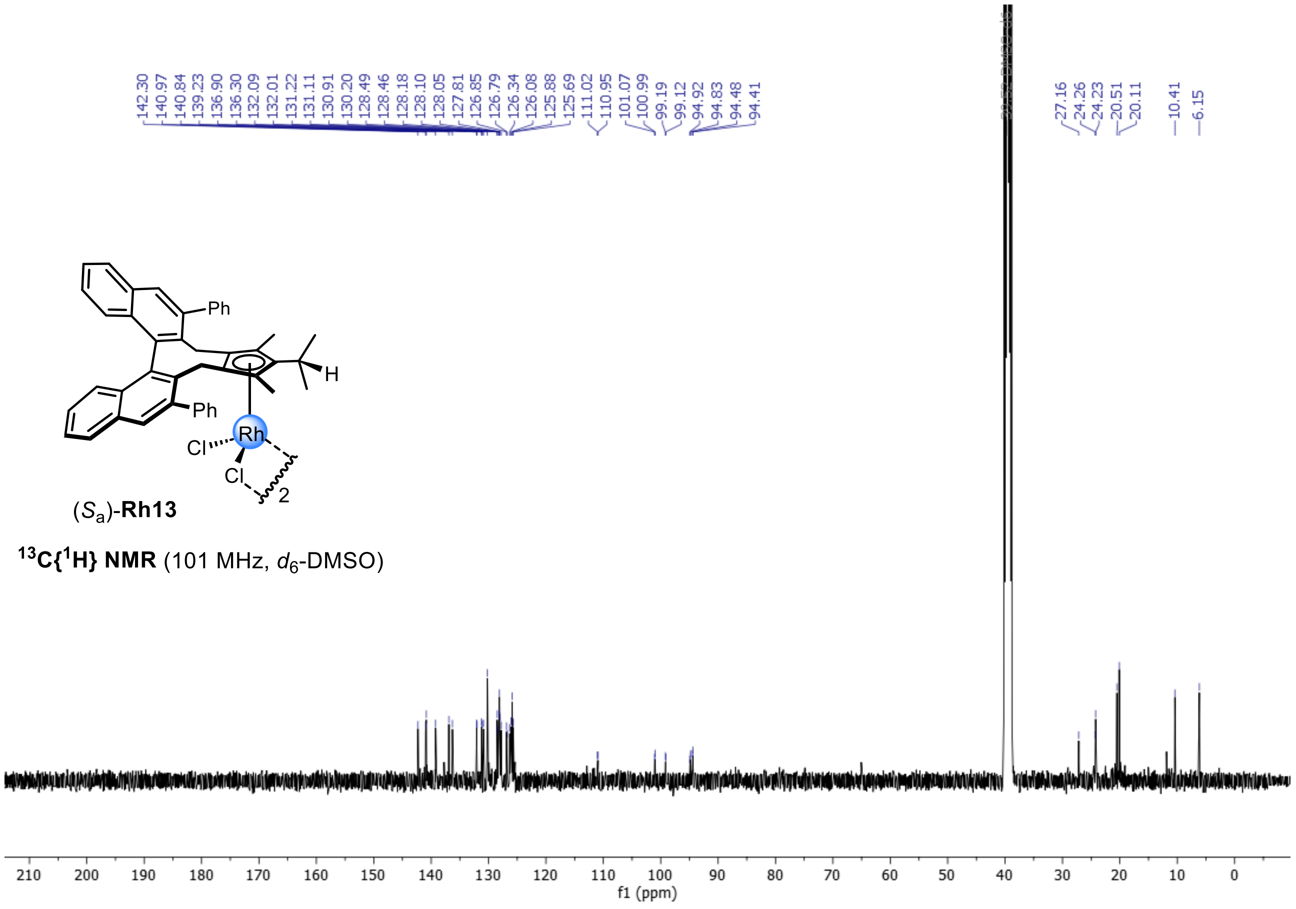
(Sa)-Rh13
13C{1H} NMR (101 MHz, d6-DMSO)
Ph
H
Rh
Cl
2
f1 (ppm)

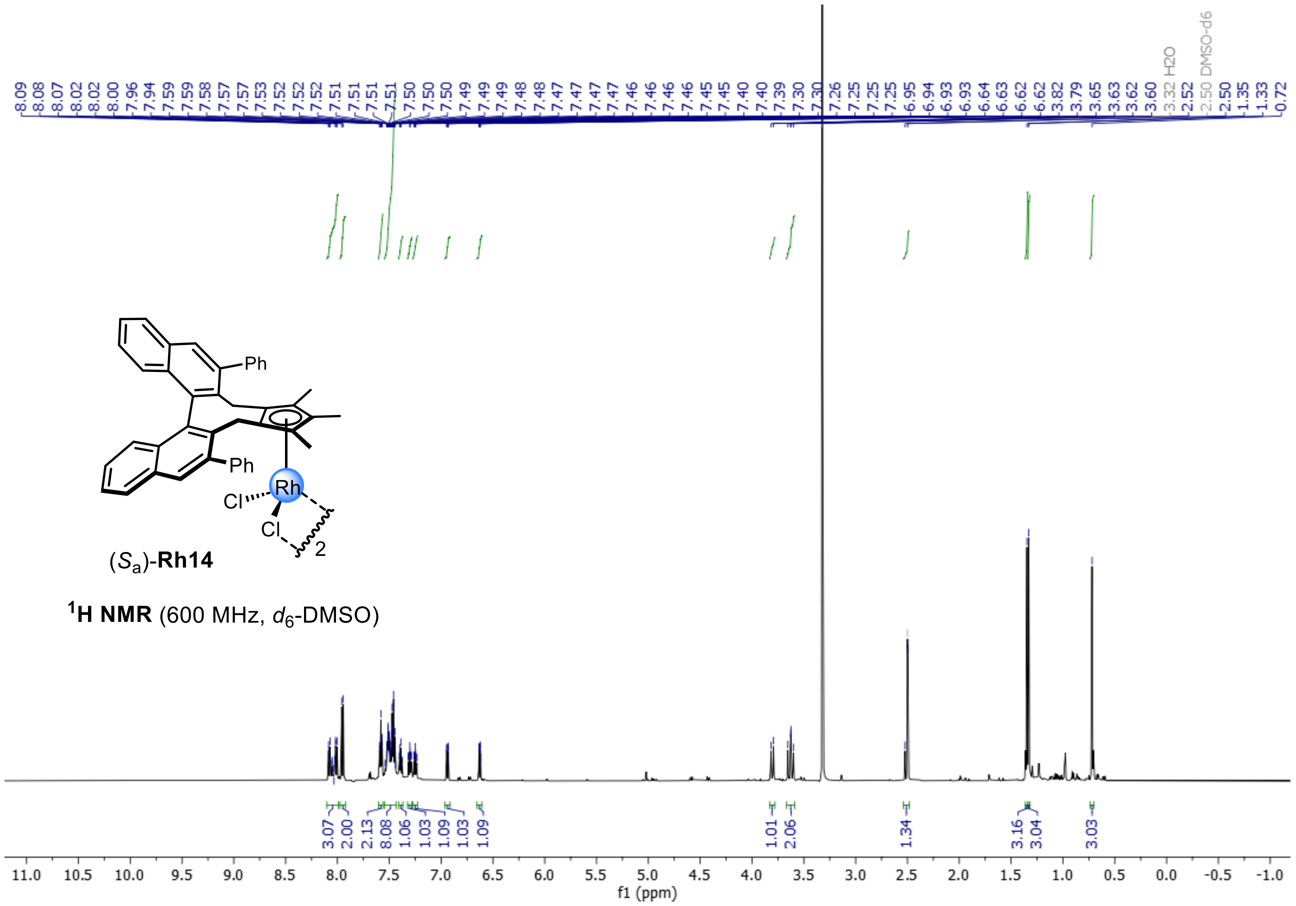
8.09 8.08 8.07 8.02 8.02 8.00 7.96 7.94 7.59 7.59 7.58 7.57 7.57 7.53 7.52 7.52 7.52 7.51 7.51 7.51 7.51 7.50 7.50 7.50 7.49 7.49 7.48 7.48 7.47 7.47 7.47 7.46 7.46 7.46 7.45 7.45 7.40 7.40 7.39 7.30 7.30 7.26 7.25 7.25 7.25 6.95 6.94 6.93 6.93 6.64 6.63 6.62 6.62 3.82 3.79 3.65 3.63 3.62 3.60 3.32 H2O 2.52 2.50 DMSO-d6 2.50 1.35 1.33 0.72
Ph
Ph
Rh
Cl
Cl
2
(S_a)-**Rh14**
^{1}H NMR (600 MHz, d_6-DMSO)
3.07 2.00 2.13 8.08 1.06 1.03 1.09 1.03 1.09 1.01 2.06 1.34 3.16 3.04 3.03
11.0 10.5 10.0 9.5 9.0 8.5 8.0 7.5 7.0 6.5 6.0 5.5 5.0 4.5 4.0 3.5 3.0 2.5 2.0 1.5 1.0 0.5 0.0 -0.5 -1.0
f1 (ppm)

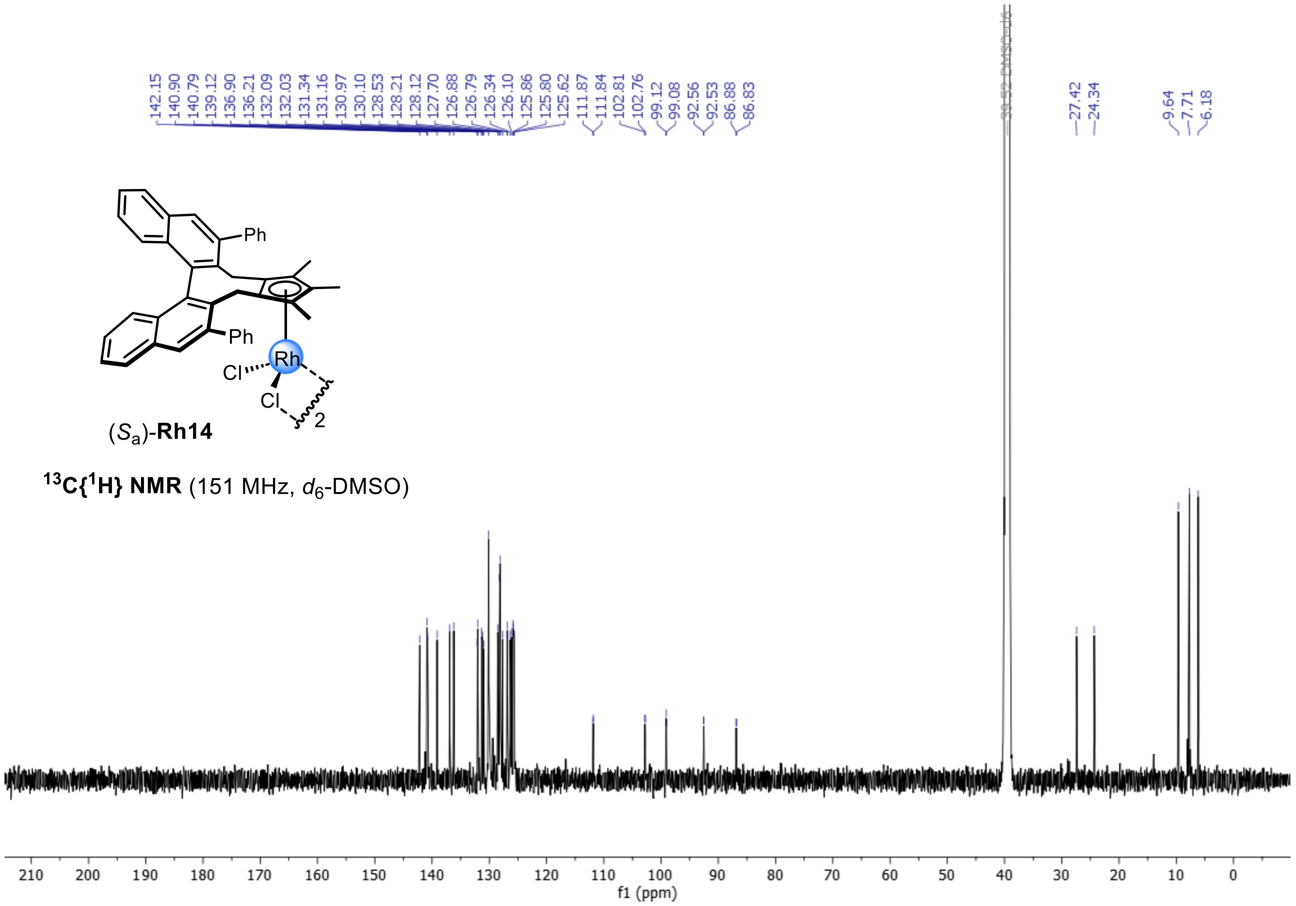
142.15 140.90 140.79 139.12 136.90 136.21 132.09 132.03 131.34 131.16 130.97 130.10 128.53 128.21 128.12 127.70 126.88 126.79 126.34 126.10 125.86 125.80 125.62 111.87 111.84 102.81 102.76 99.12 99.08 92.56 92.53 86.88 86.83
27.42 24.34
9.64 7.71 6.18
Ph
Ph
Rh
Cl
Cl
2
(S_a)-**Rh14**
^{13}C{^{1}H} NMR (151 MHz, d_6-DMSO)
210 200 190 180 170 160 150 140 130 120 110 100 90 80 70 60 50 40 30 20 10 0
f1 (ppm)

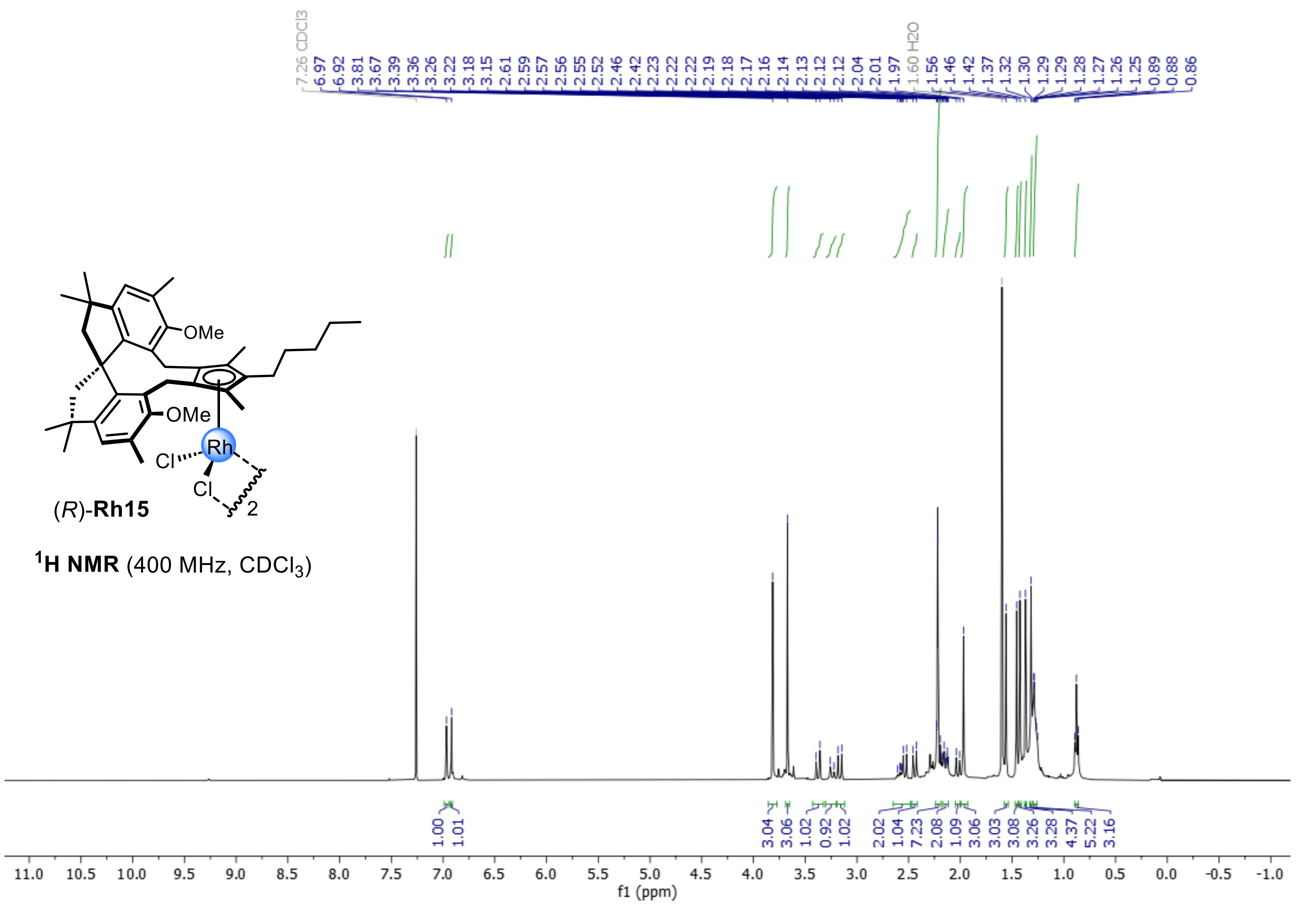
(*R*)-**Rh15**
^{1}H NMR (400 MHz, $CDCl_3$)
f1 (ppm)

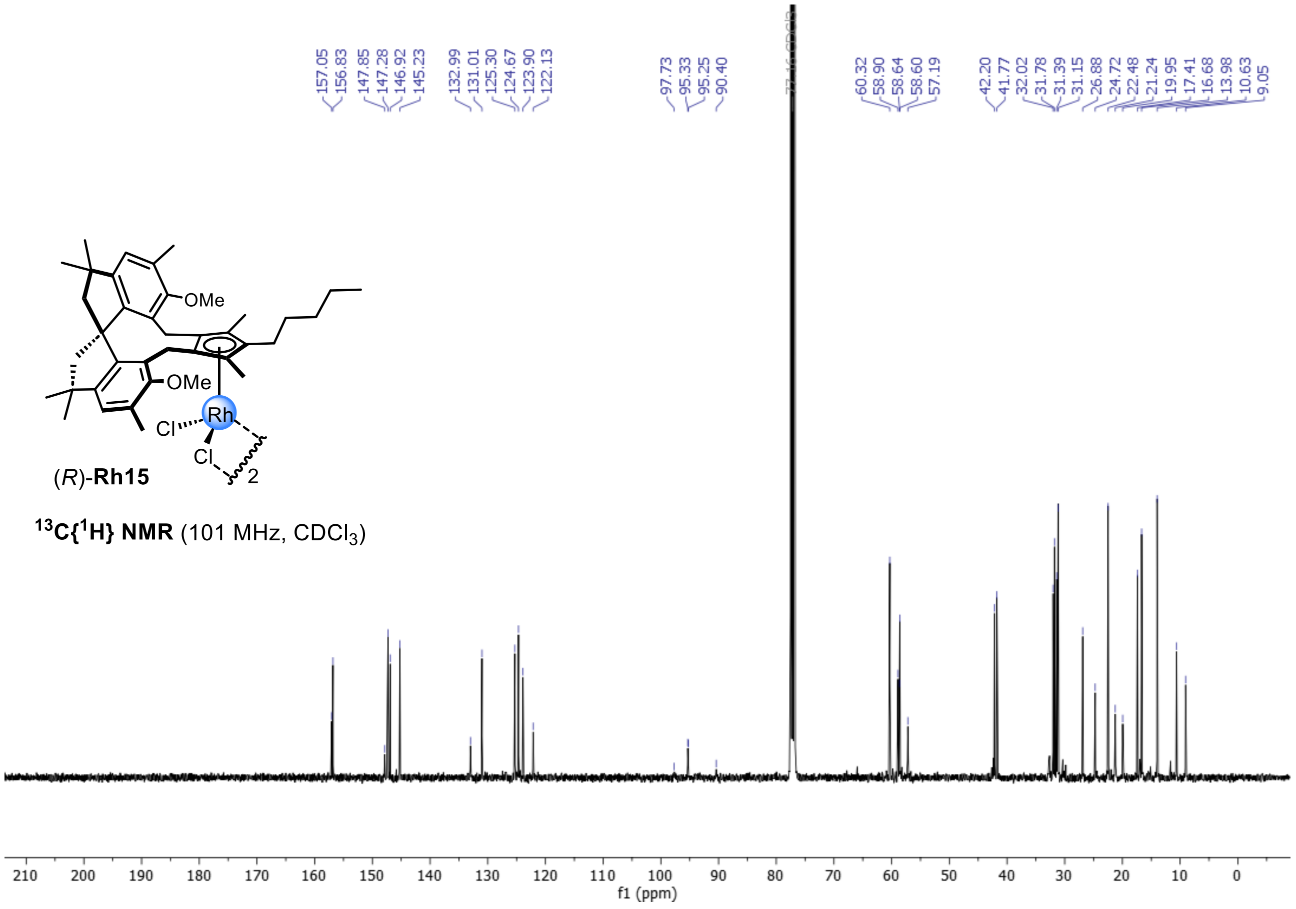
(*R*)-**Rh15**
^{13}C{^{1}H} NMR (101 MHz, $CDCl_3$)
f1 (ppm)

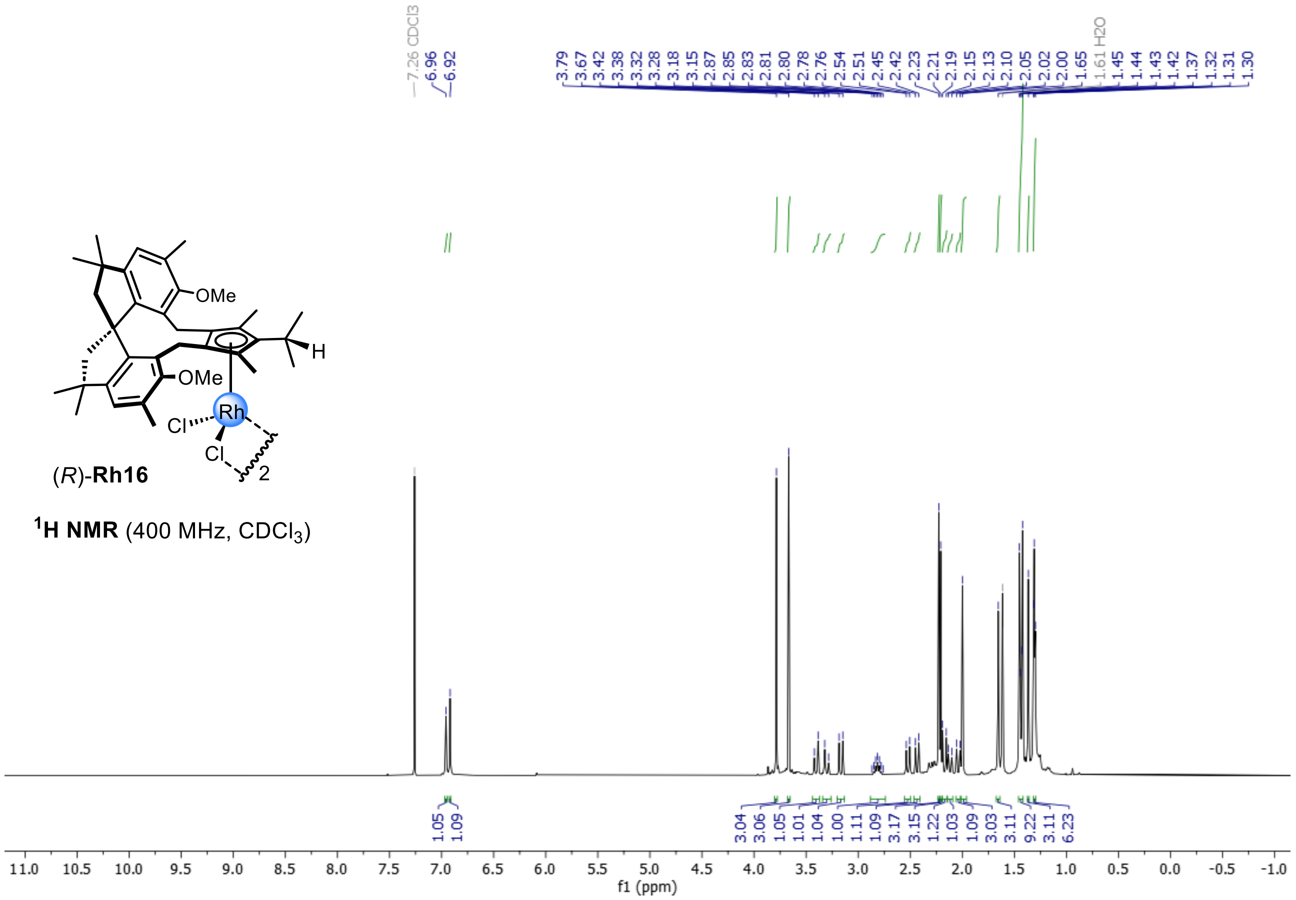
7.26 CDCl3
6.96
6.92
3.79
3.67
3.42
3.38
3.32
3.28
3.18
3.15
2.87
2.85
2.83
2.81
2.80
2.78
2.76
2.54
2.51
2.45
2.42
2.23
2.21
2.19
2.15
2.13
2.10
2.05
2.02
2.00
1.65
1.61 H2O
1.45
1.44
1.43
1.42
1.37
1.32
1.31
1.30
OMe
OMe
H
Rh
Cl
Cl
2
(R)-Rh16
1H NMR (400 MHz, CDCl3)
1.05
1.09
3.04
3.06
1.05
1.01
1.04
1.00
1.11
1.09
3.17
3.15
1.22
1.03
1.09
3.03
3.11
9.22
3.11
6.23
f1 (ppm)

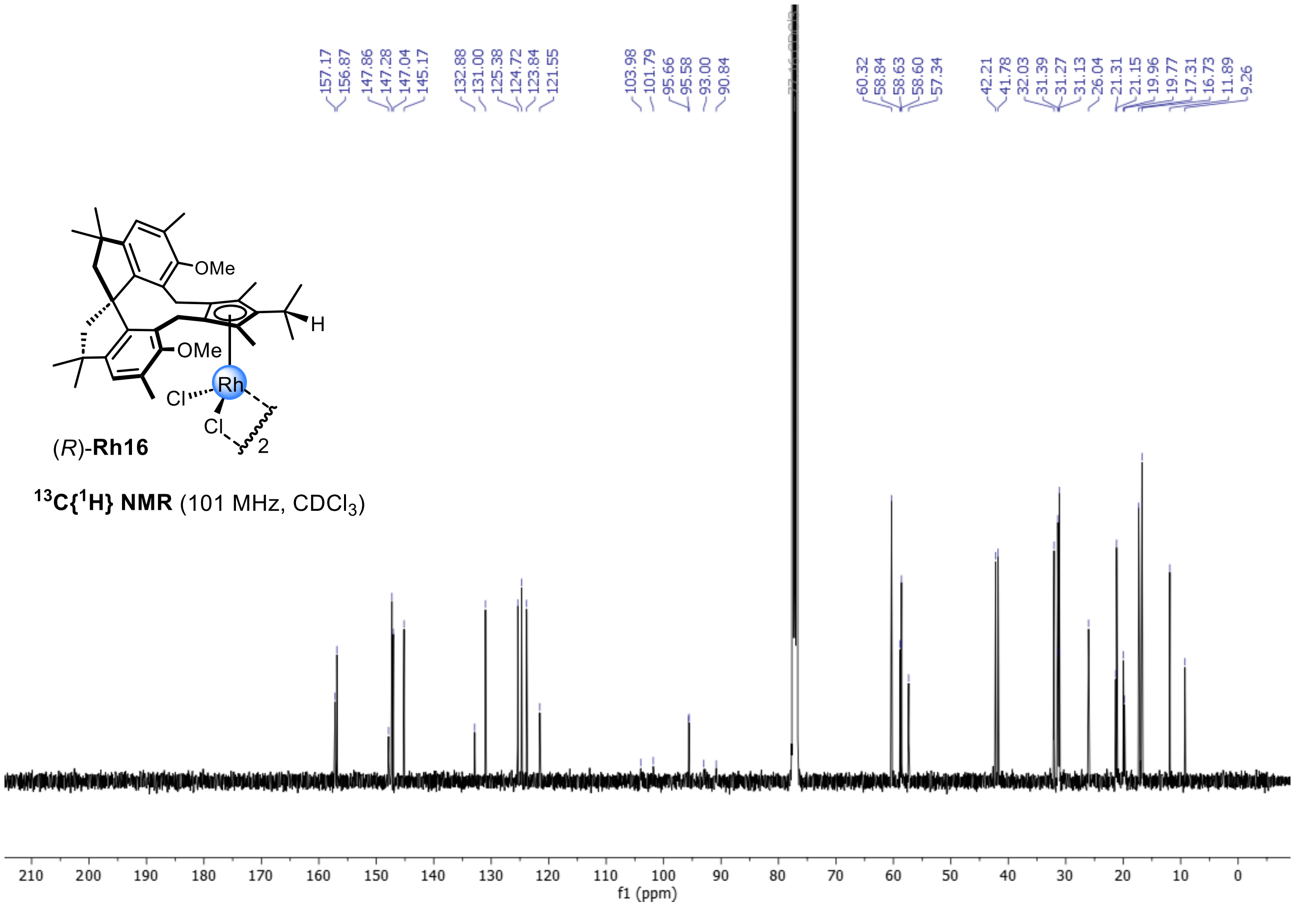
157.17
156.87
147.86
147.28
147.04
145.17
132.88
131.00
125.38
124.72
123.84
121.55
103.98
101.79
95.66
95.58
93.00
90.84
60.32
58.84
58.63
58.60
57.34
42.21
41.78
32.03
31.39
31.27
31.13
26.04
21.31
21.15
19.96
19.77
17.31
16.73
11.89
9.26
OMe
OMe
H
Rh
Cl
Cl
2
(R)-Rh16
13C{1H} NMR (101 MHz, CDCl3)
f1 (ppm)

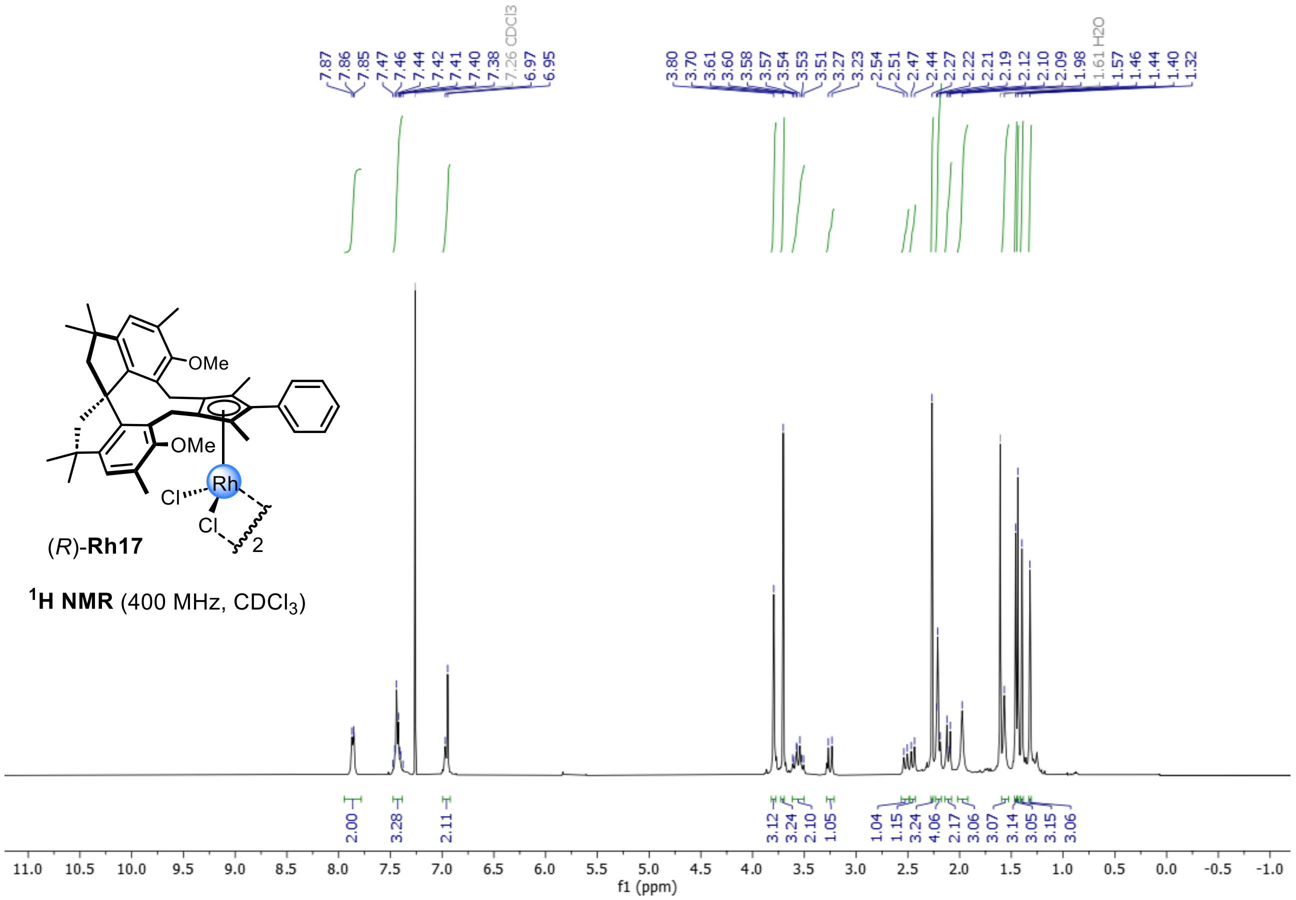
7.87
7.86
7.85
7.47
7.46
7.44
7.42
7.41
7.40
7.38
7.26 CDCl3
6.97
6.95
3.80
3.70
3.61
3.60
3.58
3.57
3.54
3.53
3.51
3.27
3.23
2.54
2.51
2.47
2.44
2.27
2.22
2.21
2.19
2.12
2.10
2.09
1.98
1.61 H2O
1.57
1.46
1.44
1.40
1.32
OMe
OMe
Rh
Cl
Cl
2
(R)-Rh17
1H NMR (400 MHz, CDCl3)
2.00
3.28
2.11
3.12
3.24
2.10
1.05
1.04
1.15
3.24
4.06
2.17
3.06
3.07
3.14
3.05
3.15
3.06
f1 (ppm)

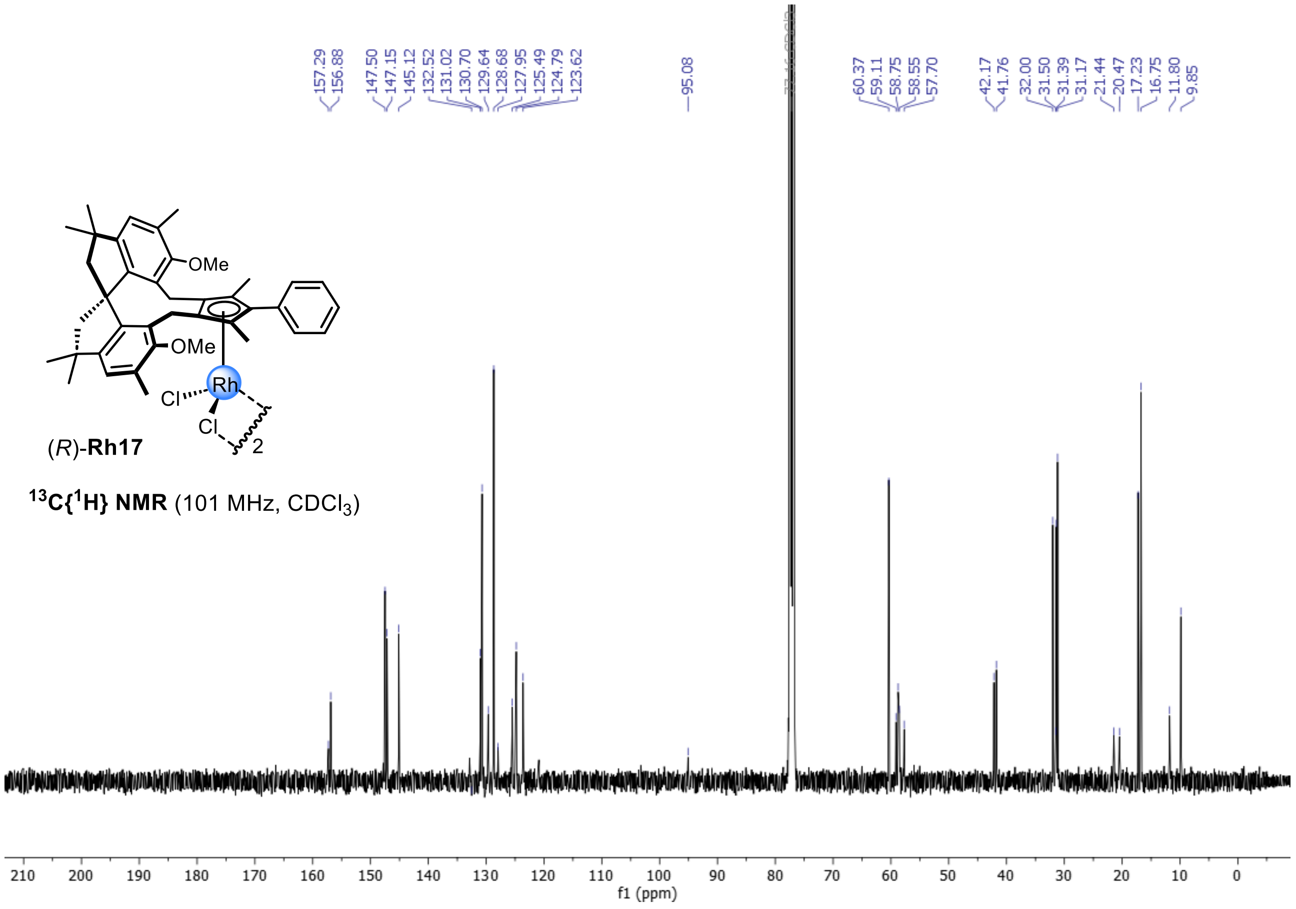
157.29
156.88
147.50
147.15
145.12
132.52
131.02
130.70
129.64
128.68
127.95
125.49
124.79
123.62
95.08
60.37
59.11
58.75
58.55
57.70
42.17
41.76
32.00
31.50
31.39
31.17
21.44
20.47
17.23
16.75
11.80
9.85
OMe
OMe
Rh
Cl
Cl
2
(R)-Rh17
13C{1H} NMR (101 MHz, CDCl3)
f1 (ppm)

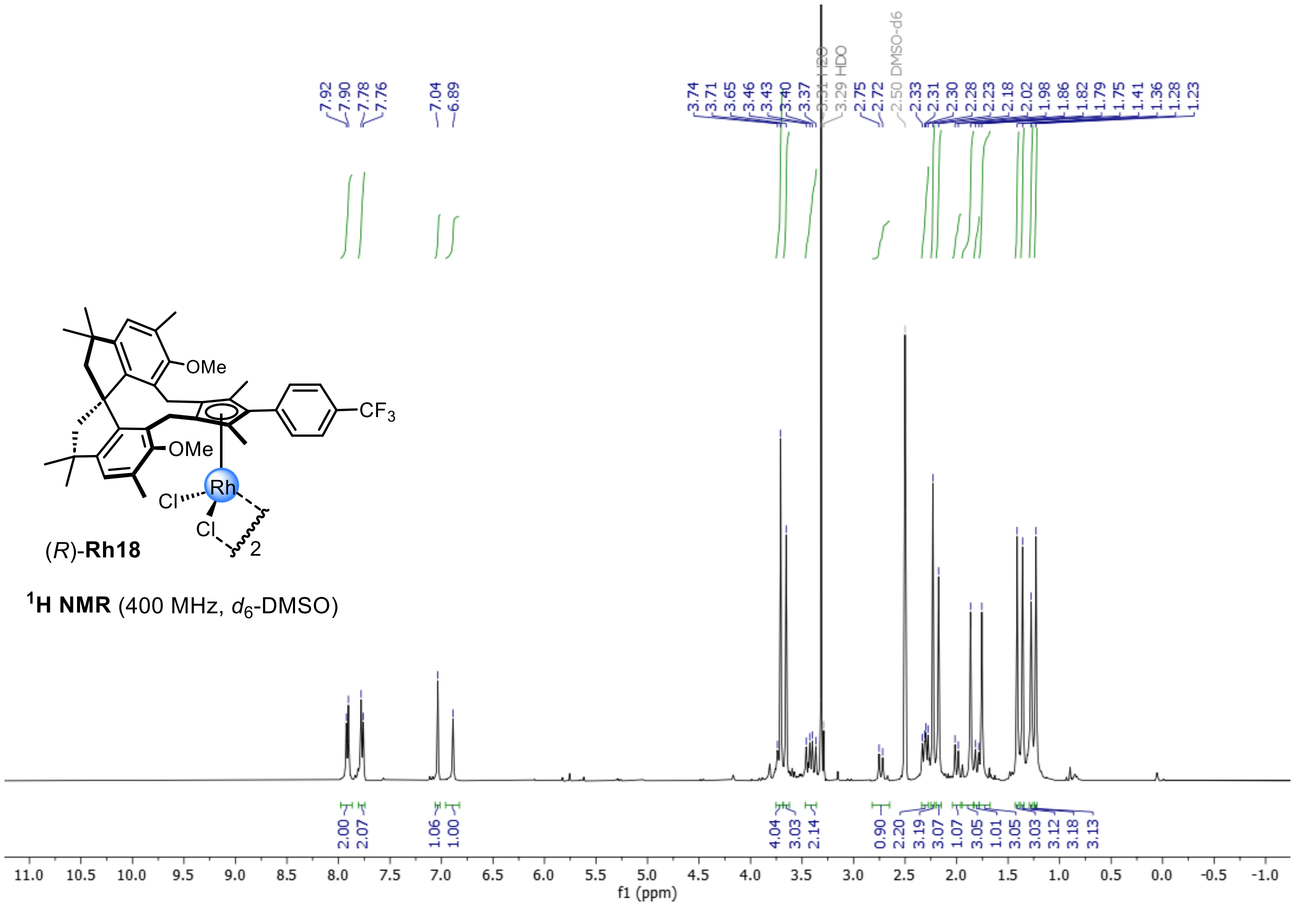
(R)-Rh18
¹H NMR (400 MHz, d6-DMSO)
f1 (ppm)

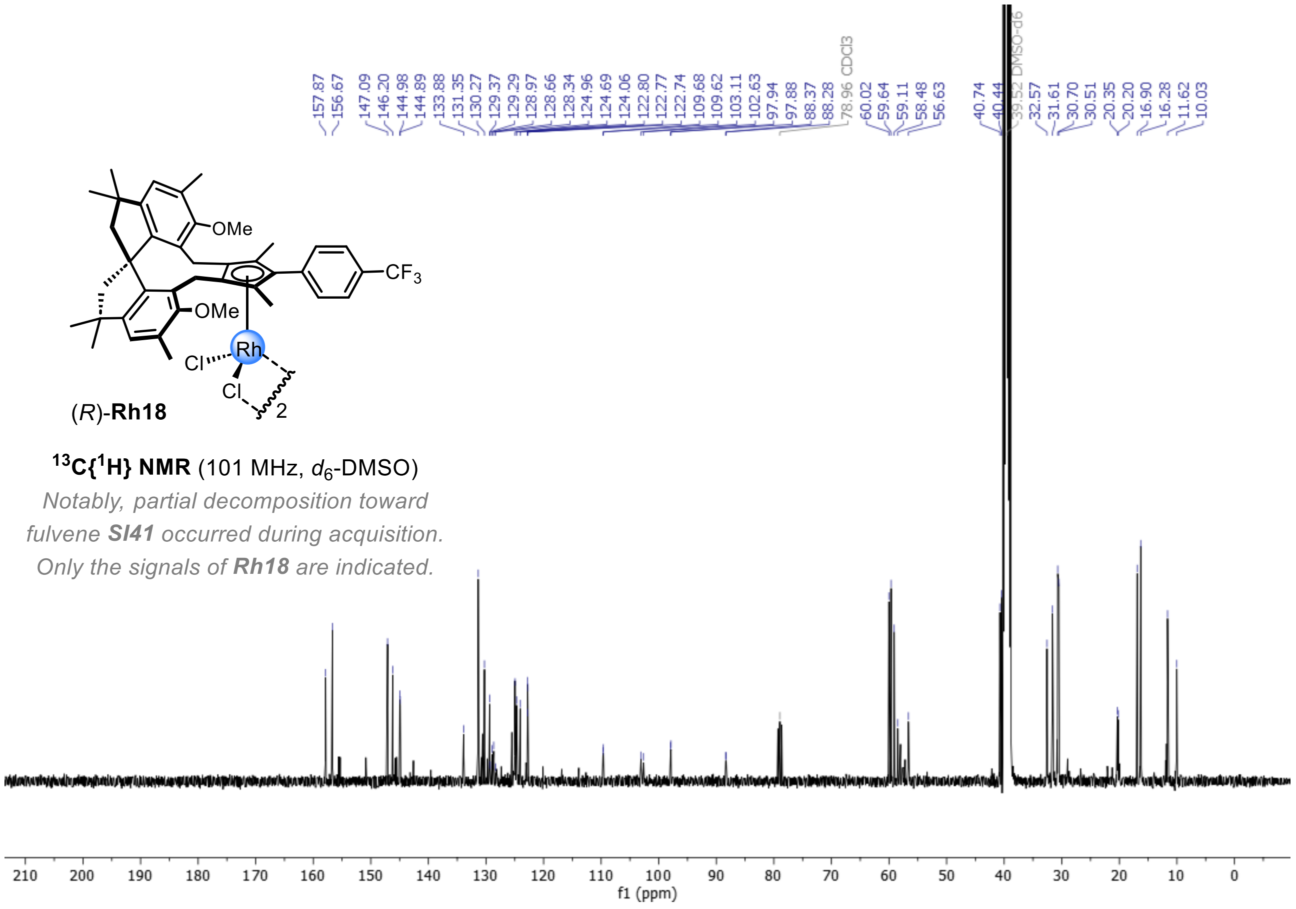
(R)-Rh18
¹³C{¹H} NMR (101 MHz, d6-DMSO)
Notably, partial decomposition toward fulvene SI41 occurred during acquisition.
Only the signals of Rh18 are indicated.
f1 (ppm)

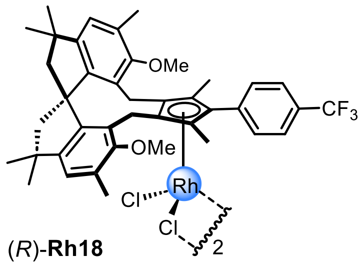

(*R*)-**Rh18**

$^{19}F\{^{1}H\}$ NMR (376 MHz, d_6-DMSO)

after 5 min in solution

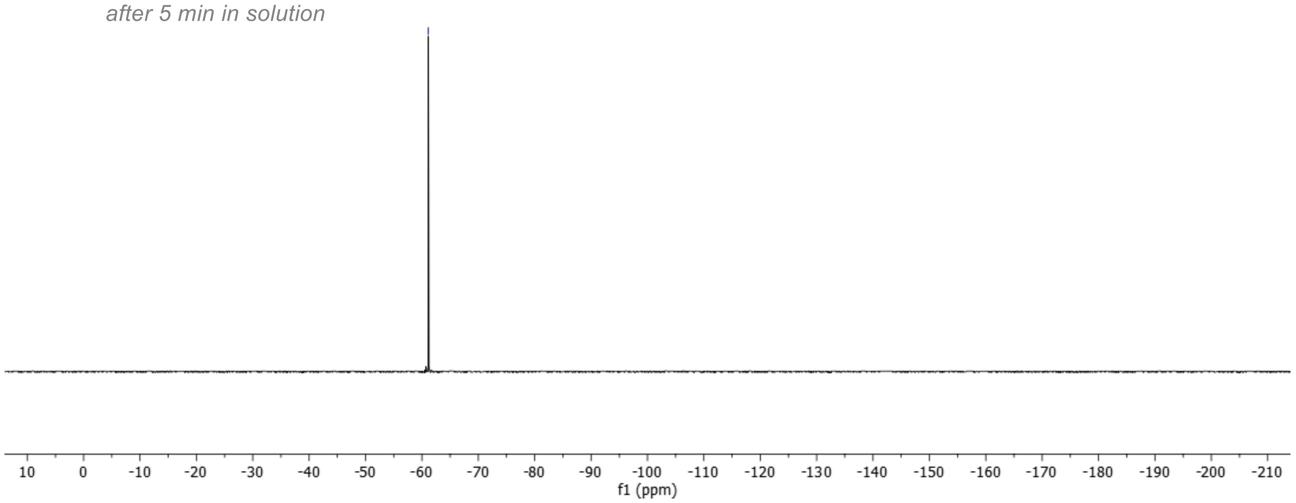

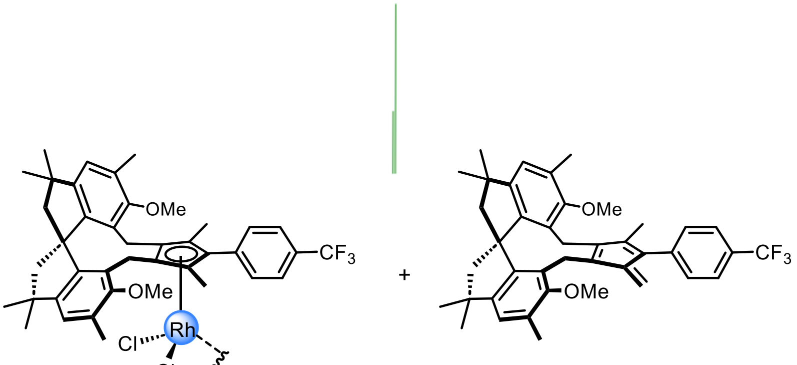

(*R*)-**Rh18** (2.7 : 1 ratio) (*R*)-**SI41**

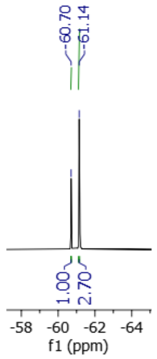

$^{19}F\{^{1}H\}$ NMR (376 MHz, d_6-DMSO)

after 10 h in solution

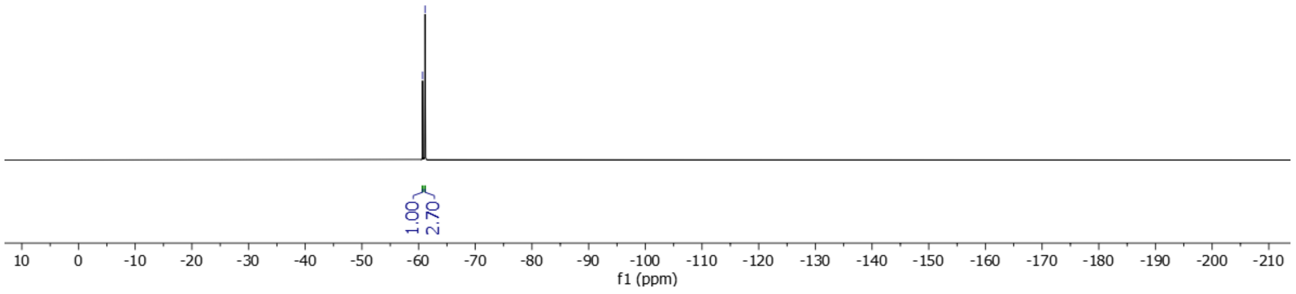

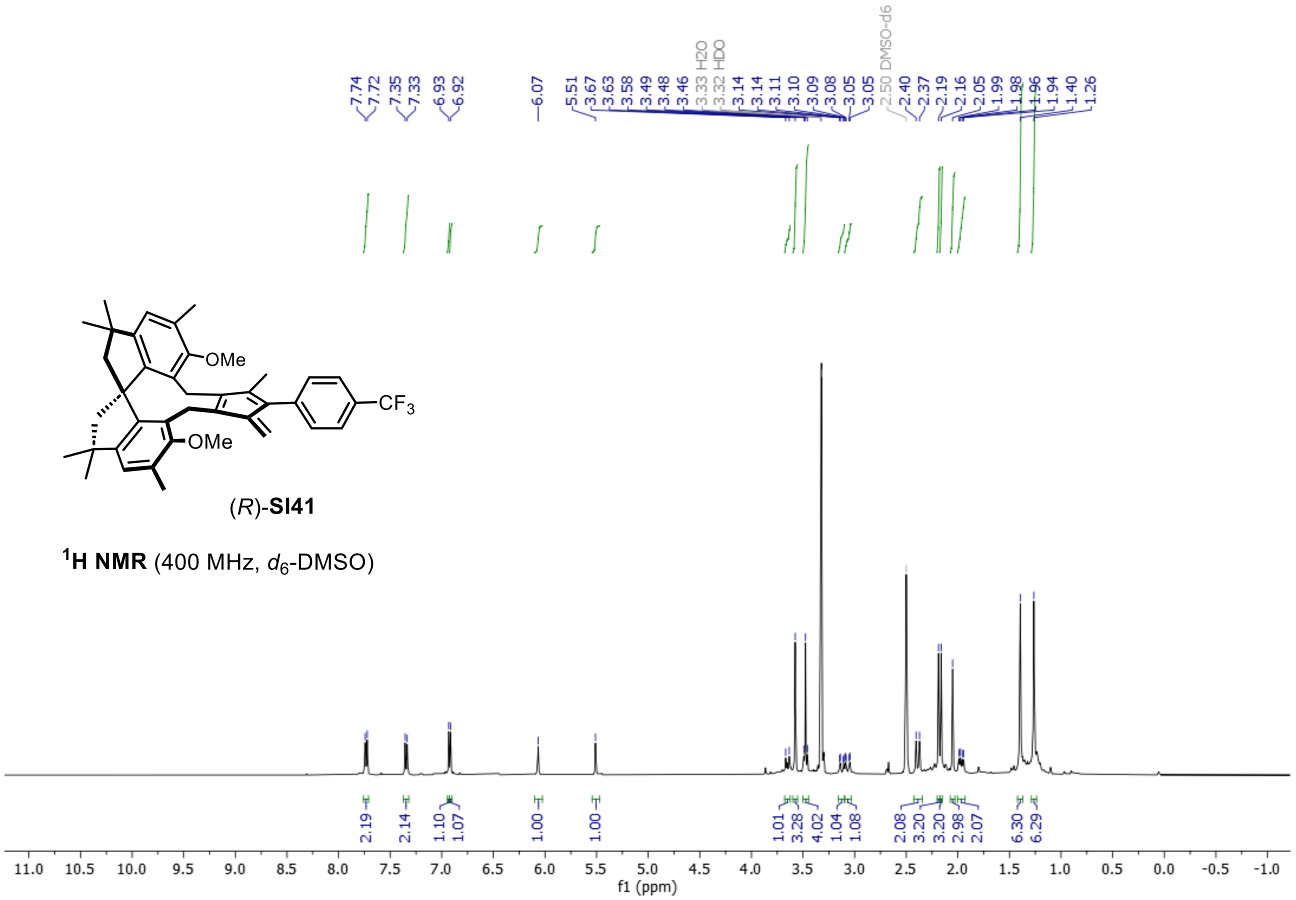

(*R*)-**SI41**

^{1}H NMR (400 MHz, d_6-DMSO)

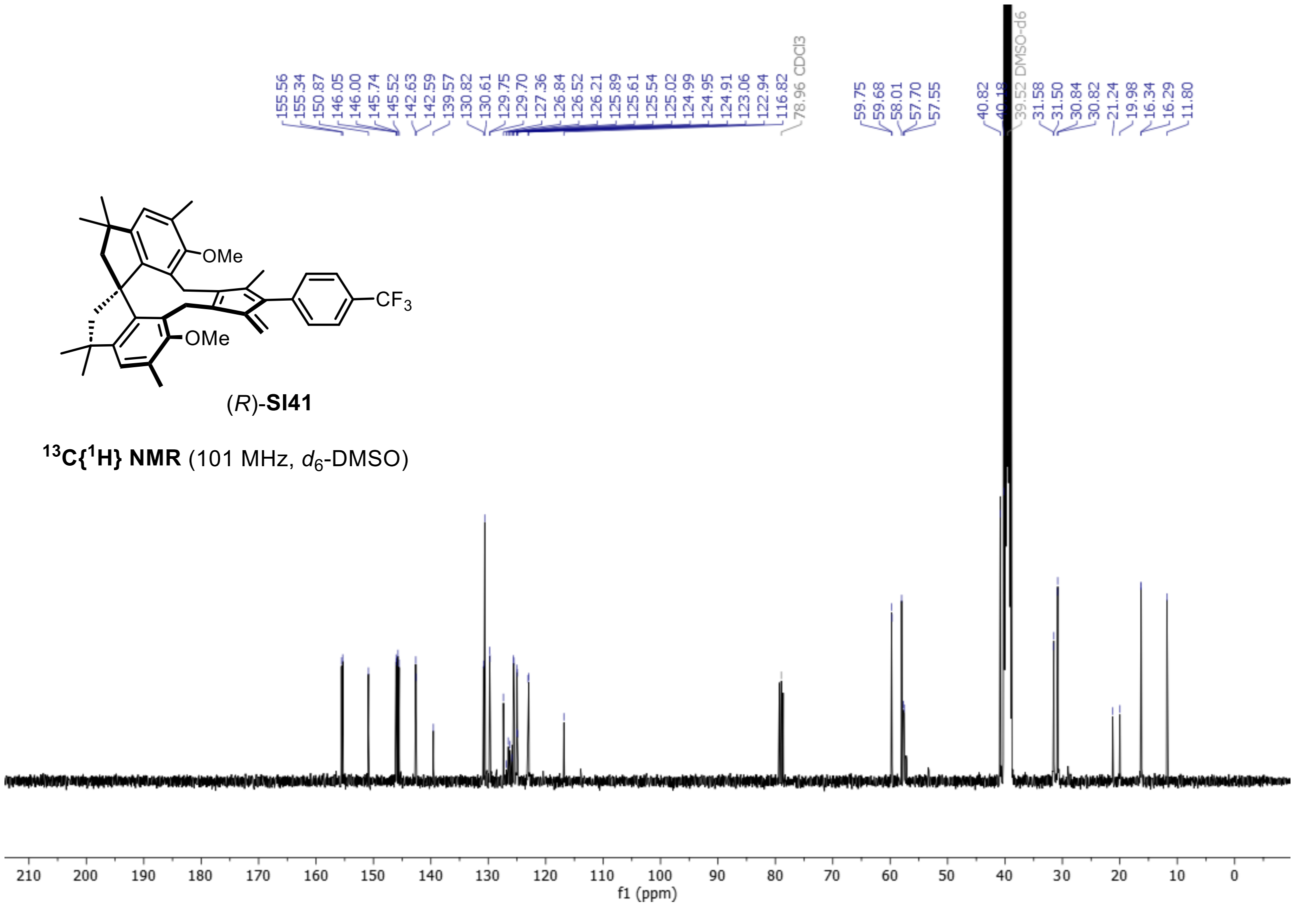

(*R*)-**SI41**

^{13}C{^{1}H} NMR (101 MHz, d_6-DMSO)

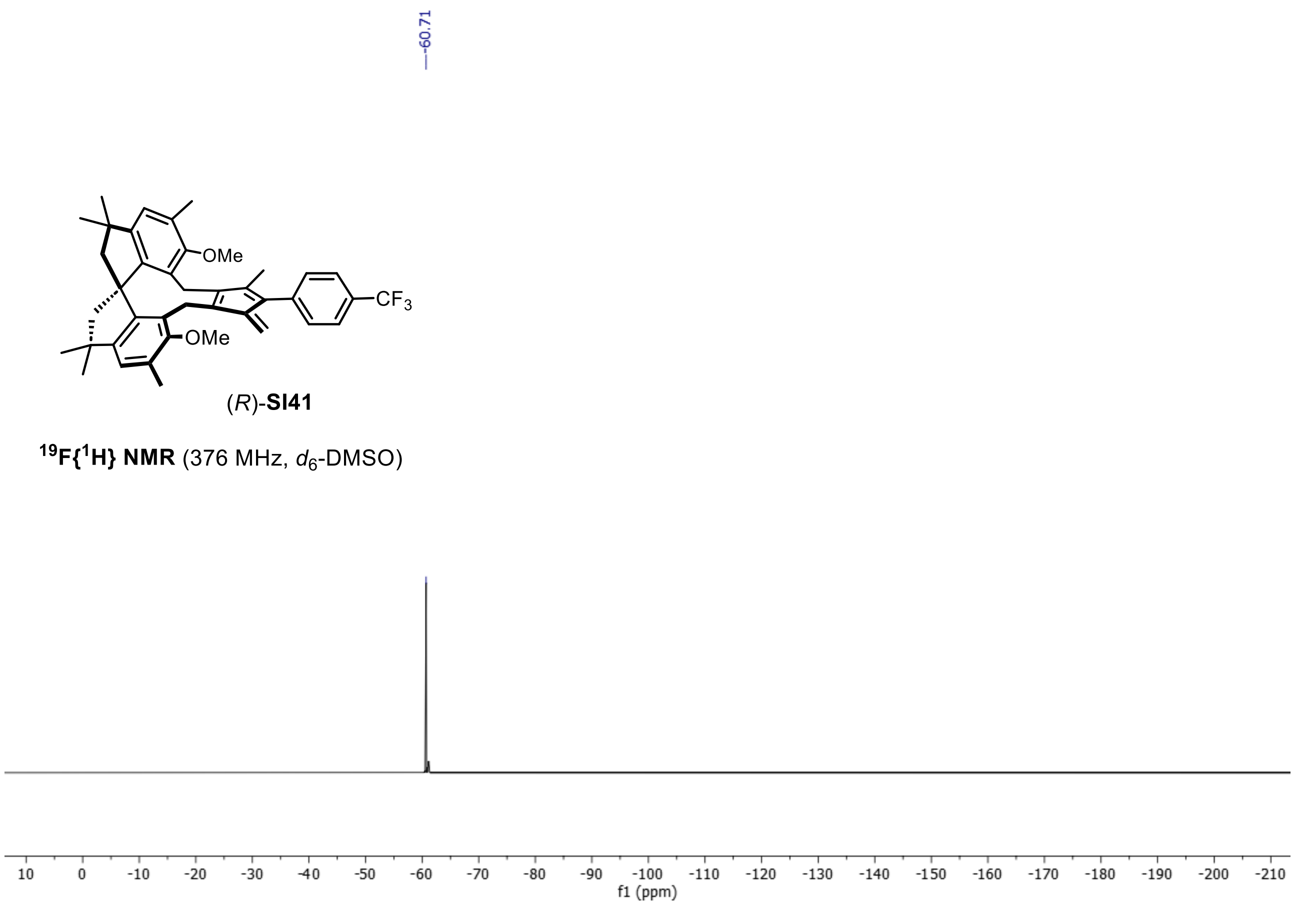

(*R*)-**SI41**

$^{19}F\{^{1}H\}$ NMR (376 MHz, d_6-DMSO)

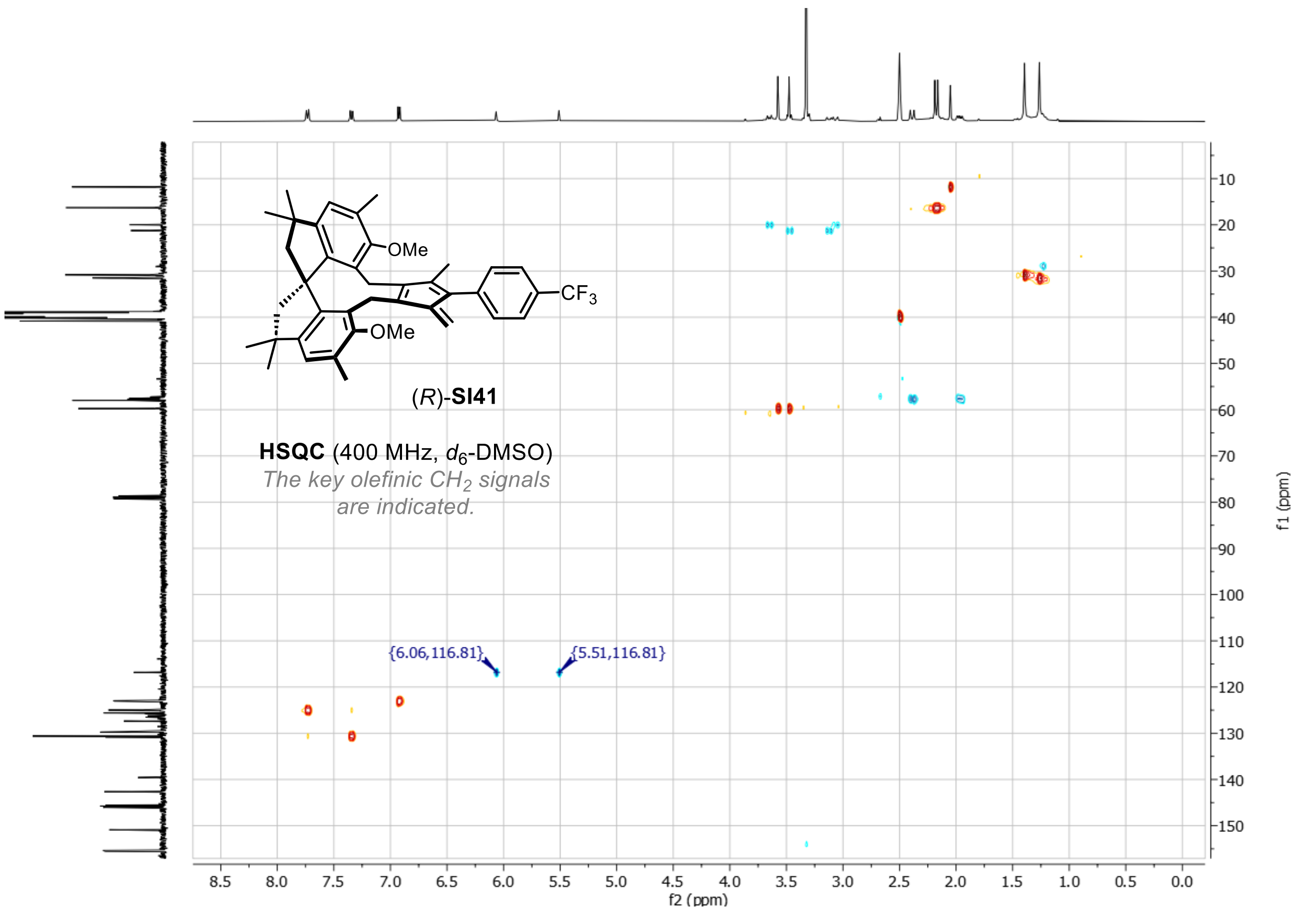

(*R*)-**SI41**

HSQC (400 MHz, d_6-DMSO)

The key olefinic CH_2 signals are indicated.

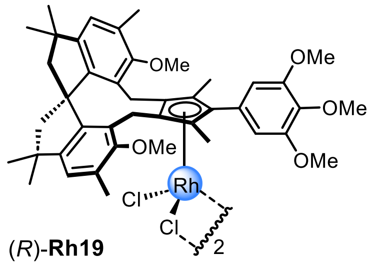

(*R*)-**Rh19**

^{1}H NMR (400 MHz, $CDCl_3$)

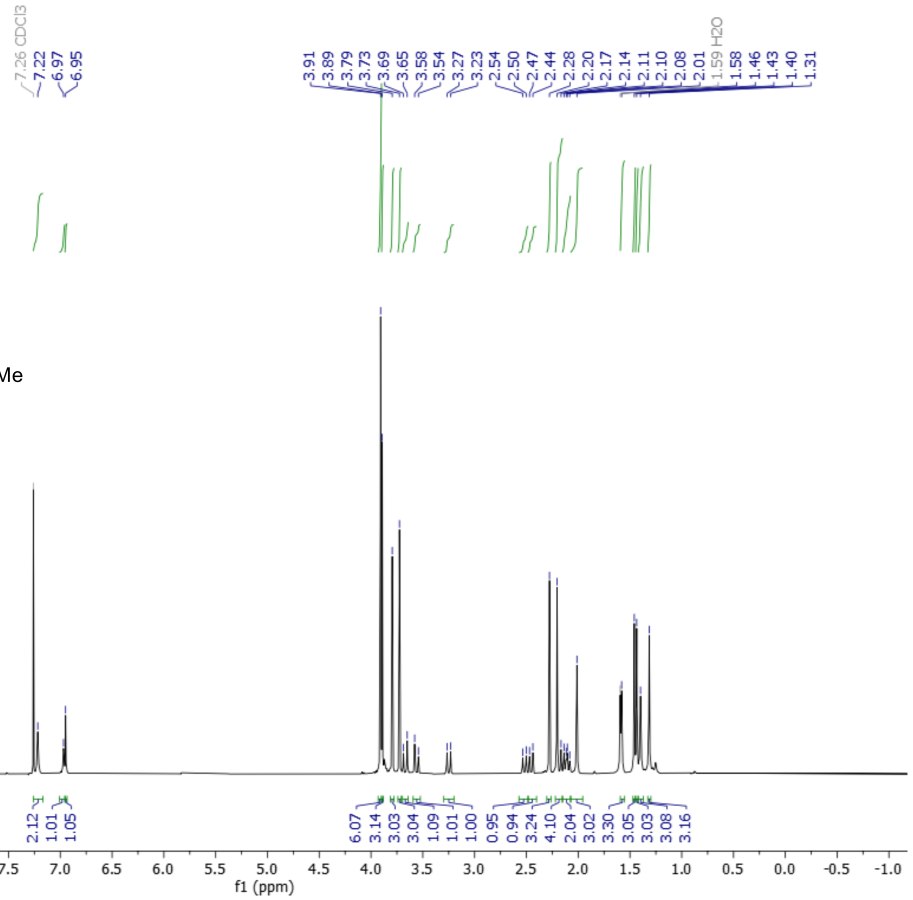

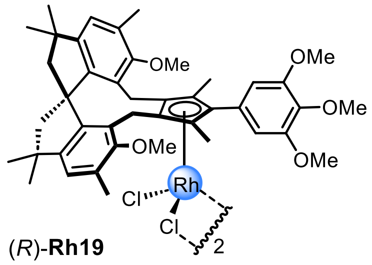

(*R*)-**Rh19**

^{13}C{^{1}H} NMR (101 MHz, $CDCl_3$)

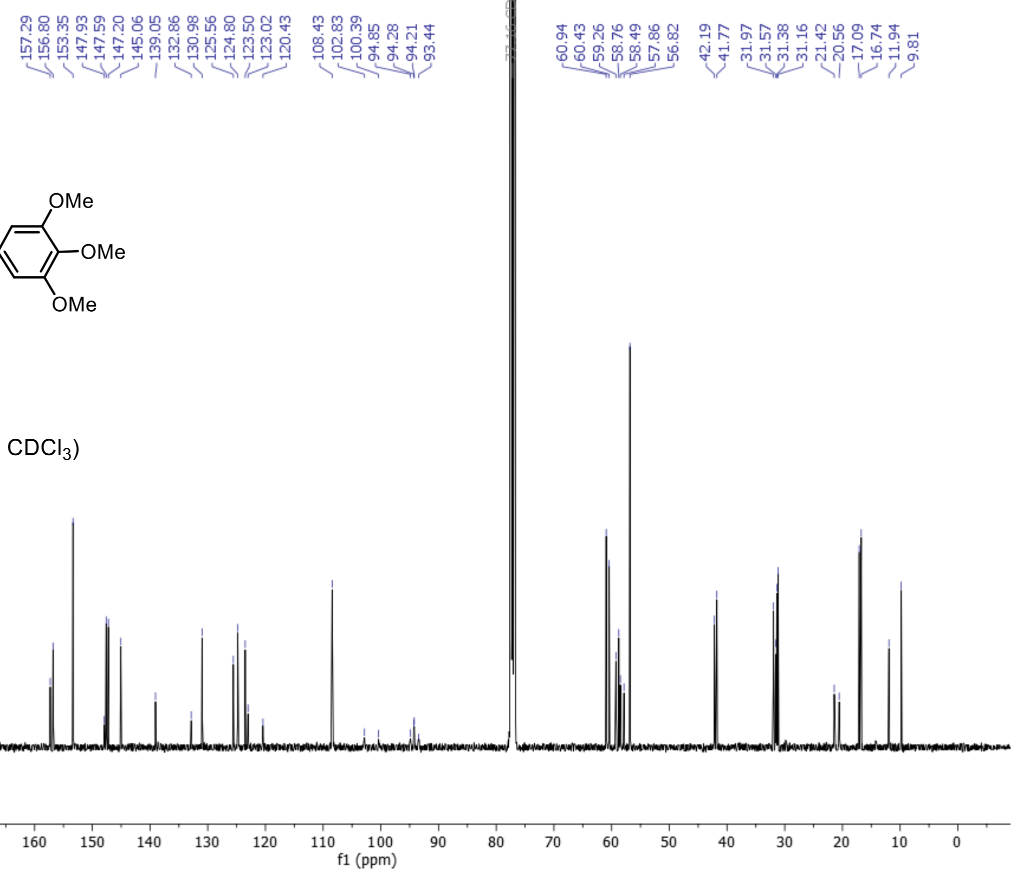

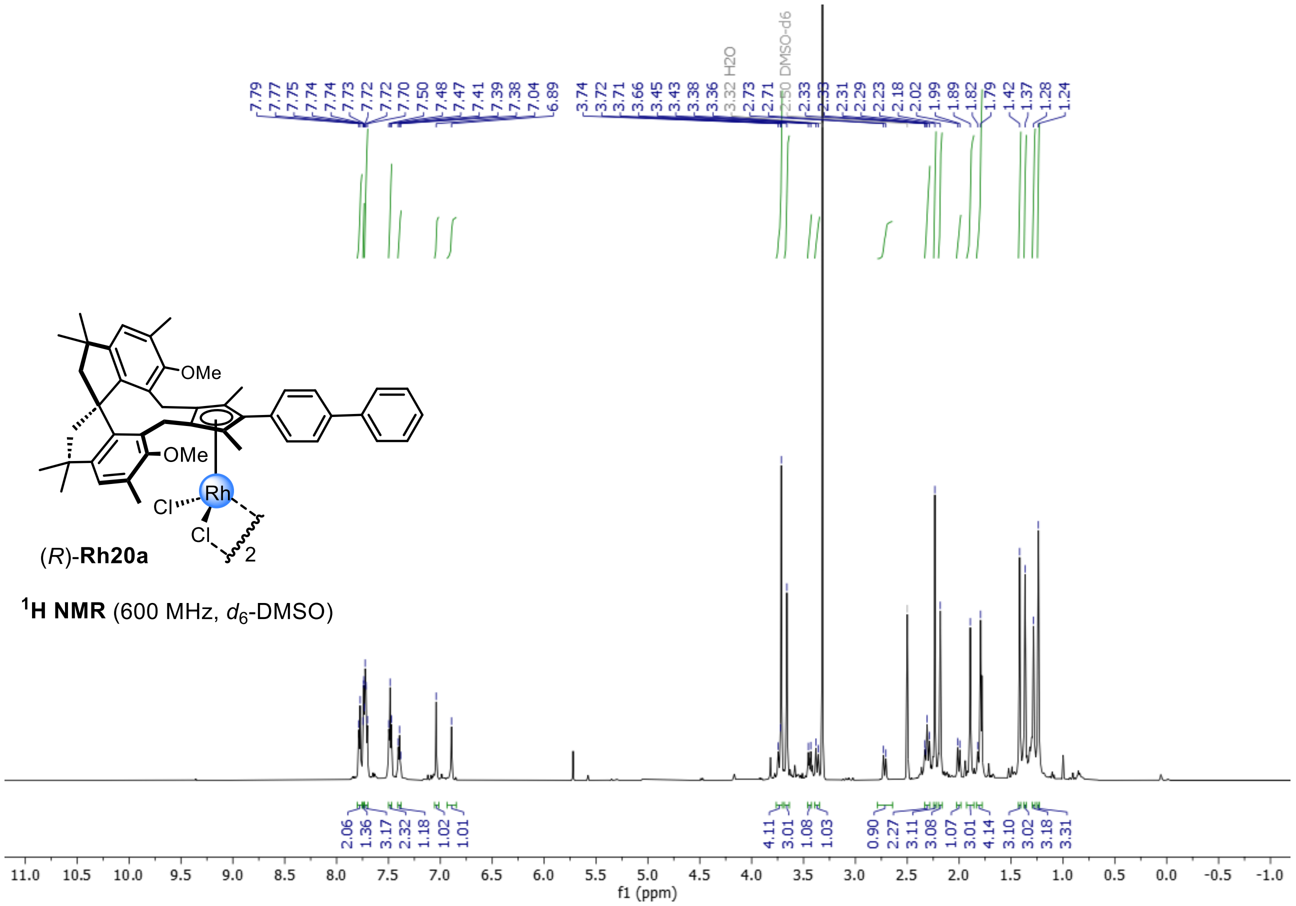
7.79 7.77 7.75 7.74 7.74 7.73 7.72 7.72 7.70 7.50 7.48 7.47 7.41 7.39 7.38 7.04 6.89
3.74 3.72 3.71 3.66 3.45 3.43 3.38 3.36 3.32 H2O 2.73 2.71 2.50 DMSO-d6 2.33 2.33 2.31 2.29 2.23 2.18 2.02 1.99 1.89 1.82 1.79 1.42 1.37 1.28 1.24
OMe
OMe
Rh
Cl
Cl
2
(R)-Rh20a
1H NMR (600 MHz, d6-DMSO)
2.06 1.36 3.17 2.32 1.18 1.02 1.01 4.11 3.01 1.08 1.03 0.90 2.27 3.11 3.08 1.07 3.01 4.14 3.10 3.02 3.18 3.31
f1 (ppm)

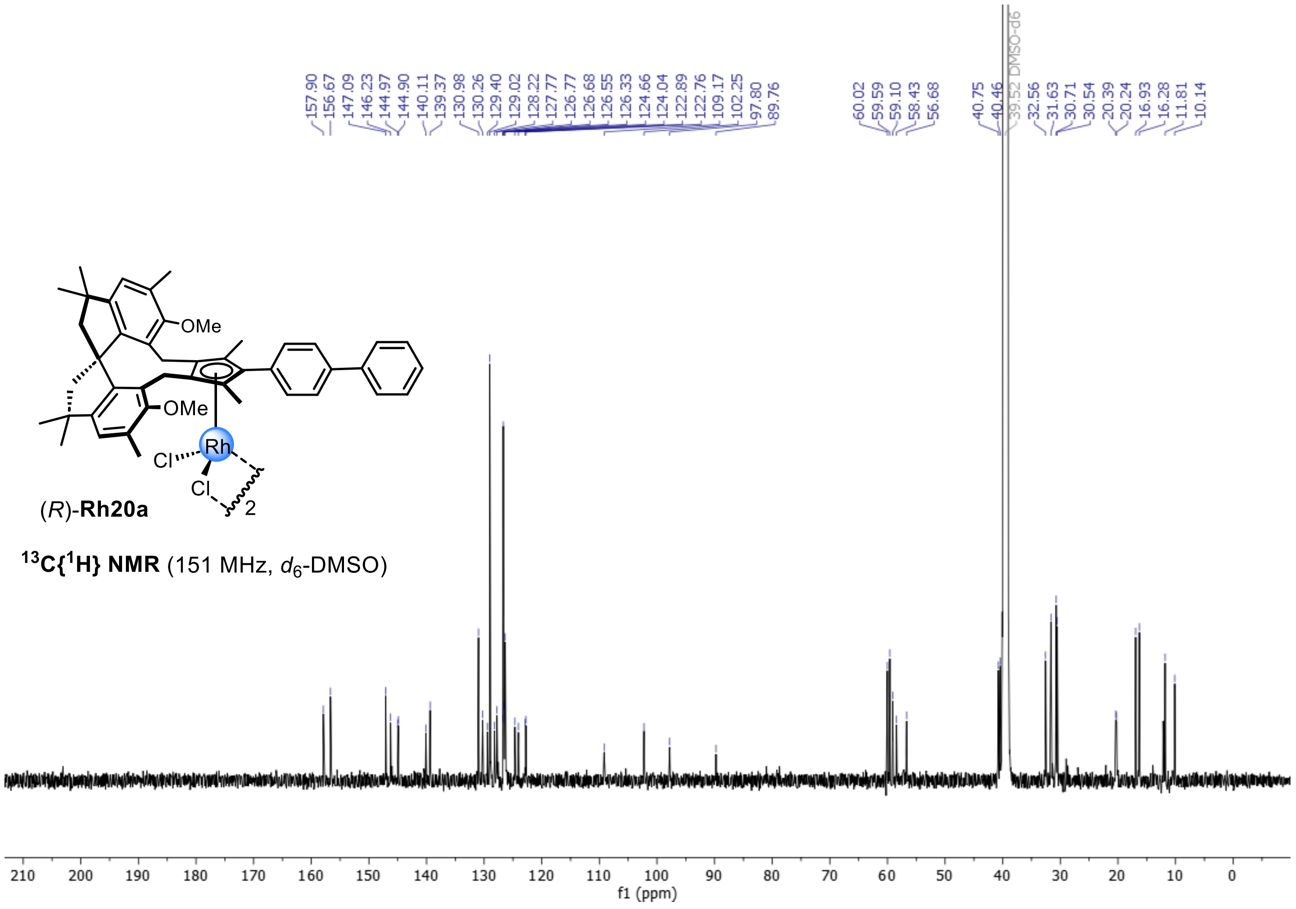
157.90 156.67 147.09 146.23 144.97 144.90 140.11 139.37 130.98 130.26 129.40 129.02 128.22 127.77 126.77 126.68 126.55 126.33 124.66 124.04 122.89 122.76 109.17 102.25 97.80 89.76
60.02 59.59 59.10 58.43 56.68 40.75 40.46 39.52 DMSO-d6 32.56 31.63 30.71 30.54 20.39 20.24 16.93 16.28 11.81 10.14
OMe
OMe
Rh
Cl
Cl
2
(R)-Rh20a
13C{1H} NMR (151 MHz, d6-DMSO)
f1 (ppm)

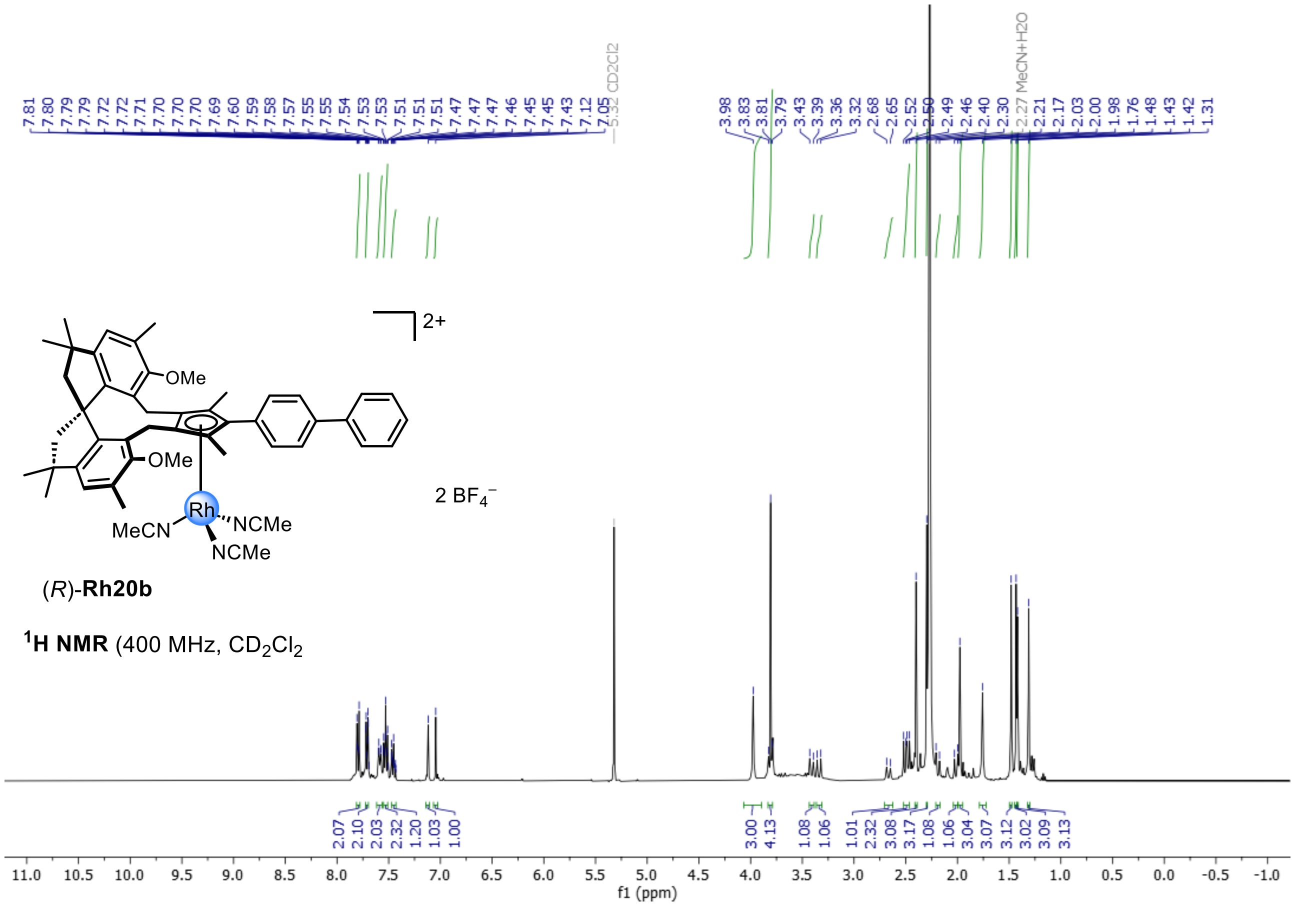

7.81 7.80 7.79 7.79 7.72 7.72 7.71 7.70 7.70 7.70 7.69 7.60 7.59 7.58 7.57 7.55 7.55 7.54 7.53 7.53 7.51 7.51 7.51 7.47 7.47 7.46 7.45 7.45 7.43 7.12 7.05
5.32 CD2Cl2
3.98 3.83 3.81 3.79 3.43 3.39 3.36 3.32 2.68 2.65 2.52 2.50 2.49 2.46 2.40 2.30
2.27 MeCN+H2O
2.21 2.17 2.03 2.00 1.98 1.76 1.48 1.43 1.42 1.31
2+
OMe
OMe
Rh
MeCN
NCMe
NCMe
2 BF4⁻
(R)-Rh20b
1H NMR (400 MHz, CD2Cl2
2.07 2.10 2.03 2.32 1.20 1.03 1.00
3.00 4.13 1.08 1.06 1.01 2.32 3.08 3.17 1.08 1.06 3.04 3.07 3.12 3.02 3.09 3.13
11.0 10.5 10.0 9.5 9.0 8.5 8.0 7.5 7.0 6.5 6.0 5.5 5.0 4.5 4.0 3.5 3.0 2.5 2.0 1.5 1.0 0.5 0.0 -0.5 -1.0
f1 (ppm)

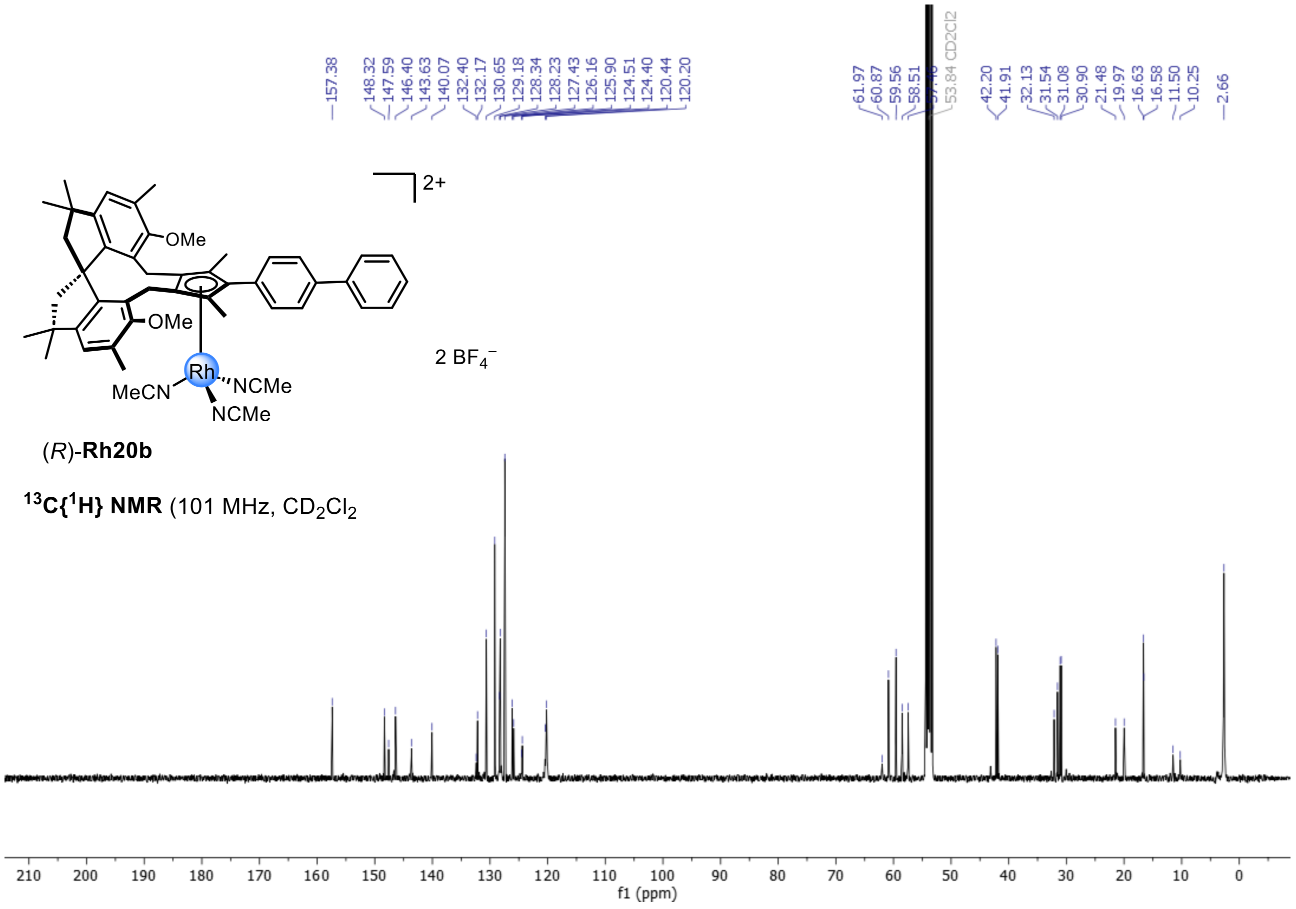

157.38
148.32 147.59 146.40 143.63 140.07 132.40 132.17 130.65 129.18 128.34 128.23 127.43 126.16 125.90 124.51 124.40 120.44 120.20
61.97 60.87 59.56 58.51
53.84 CD2Cl2
42.20 41.91 32.13 31.54 31.08 30.90 21.48 19.97 16.63 16.58 11.50 10.25 2.66
2+
OMe
OMe
Rh
MeCN
NCMe
NCMe
2 BF4⁻
(R)-Rh20b
13C{1H} NMR (101 MHz, CD2Cl2
210 200 190 180 170 160 150 140 130 120 110 100 90 80 70 60 50 40 30 20 10 0
f1 (ppm)

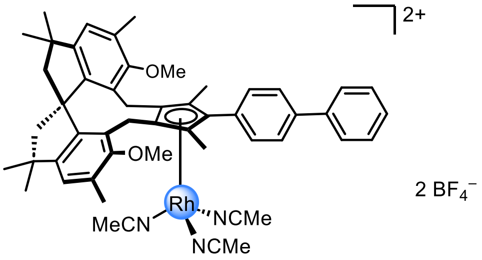

(*R*)-**Rh20b**

^{19}F{^{1}H} NMR (376 MHz, CD_2Cl_2

Two signals due to ^{10}B and ^{11}B isotopes.

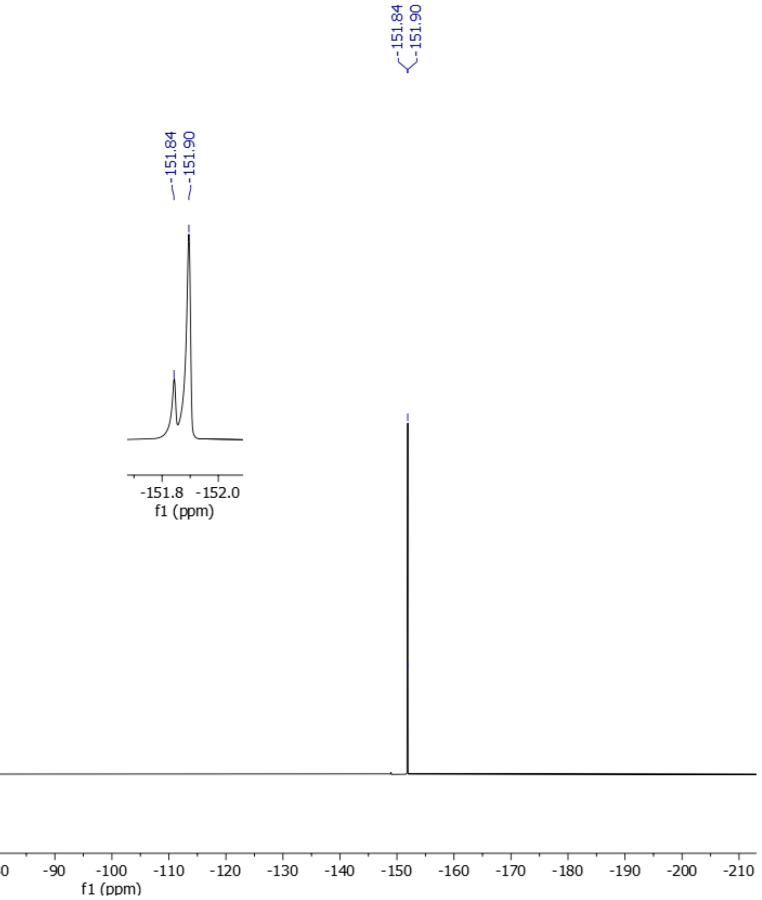

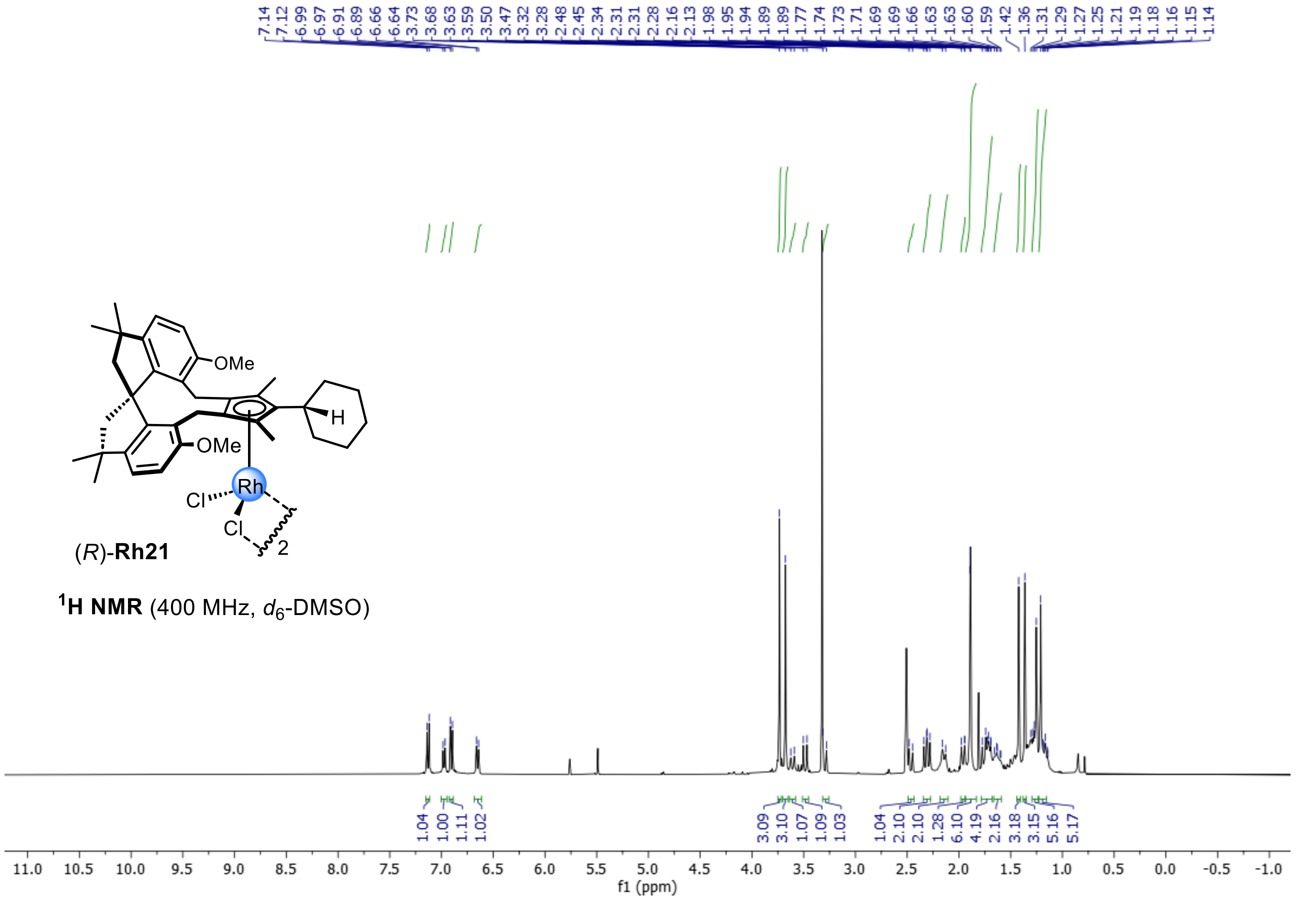

(*R*)-**Rh21**

^{1}H NMR (400 MHz, d_6-DMSO)

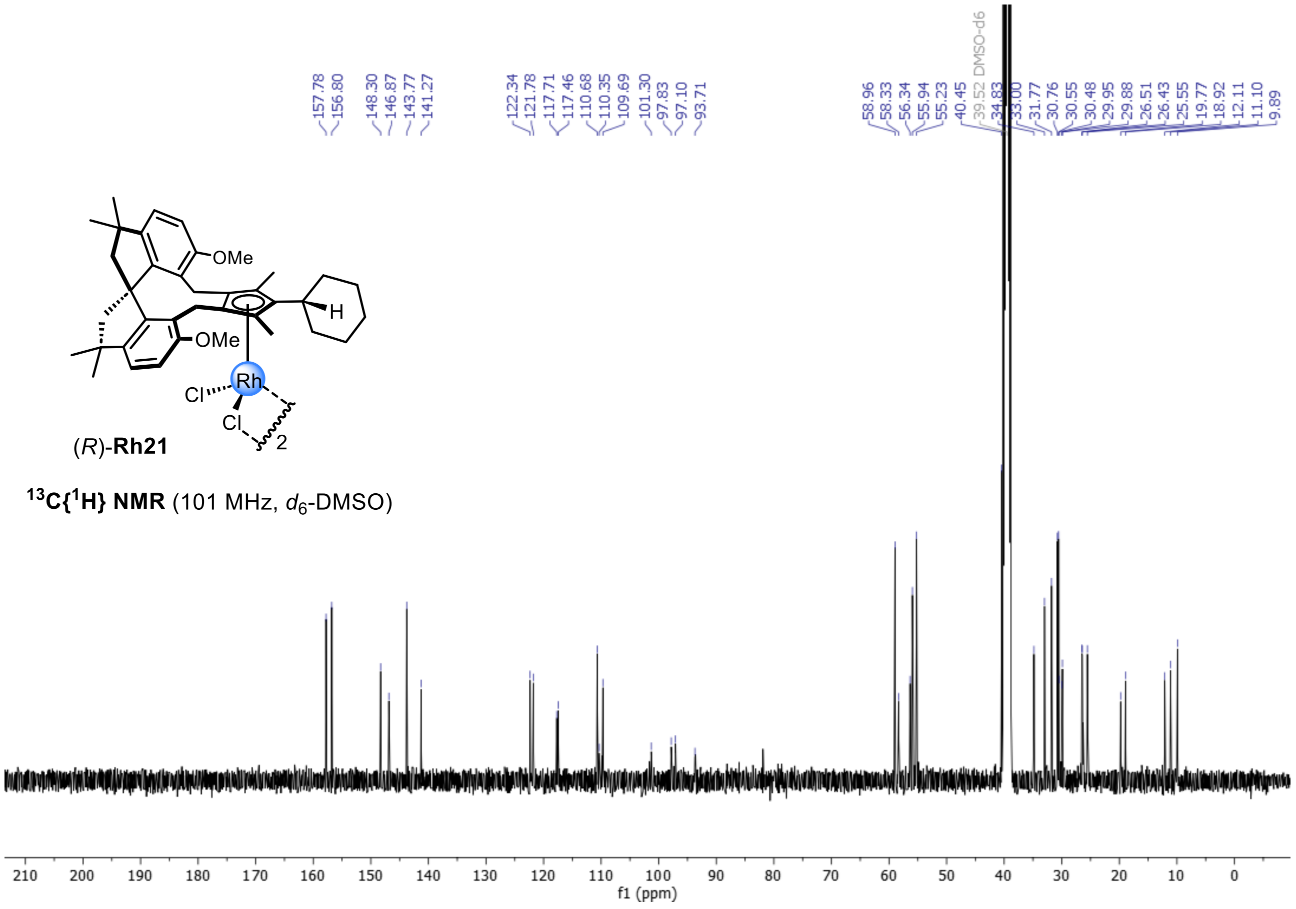

(*R*)-**Rh21**

^{13}C{^{1}H} NMR (101 MHz, d_6-DMSO)

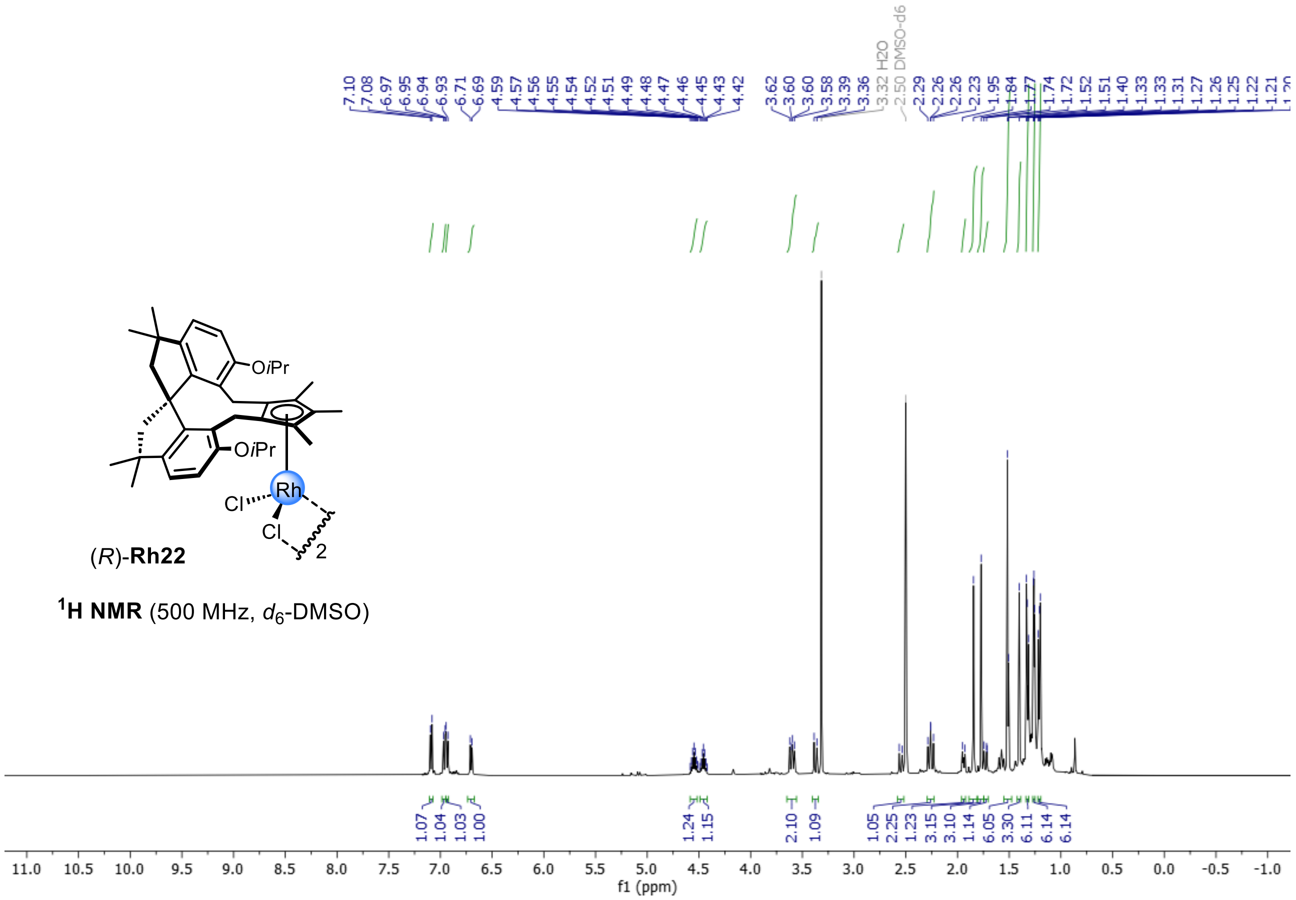

(*R*)-**Rh22**

^{1}H NMR (500 MHz, d_6-DMSO)

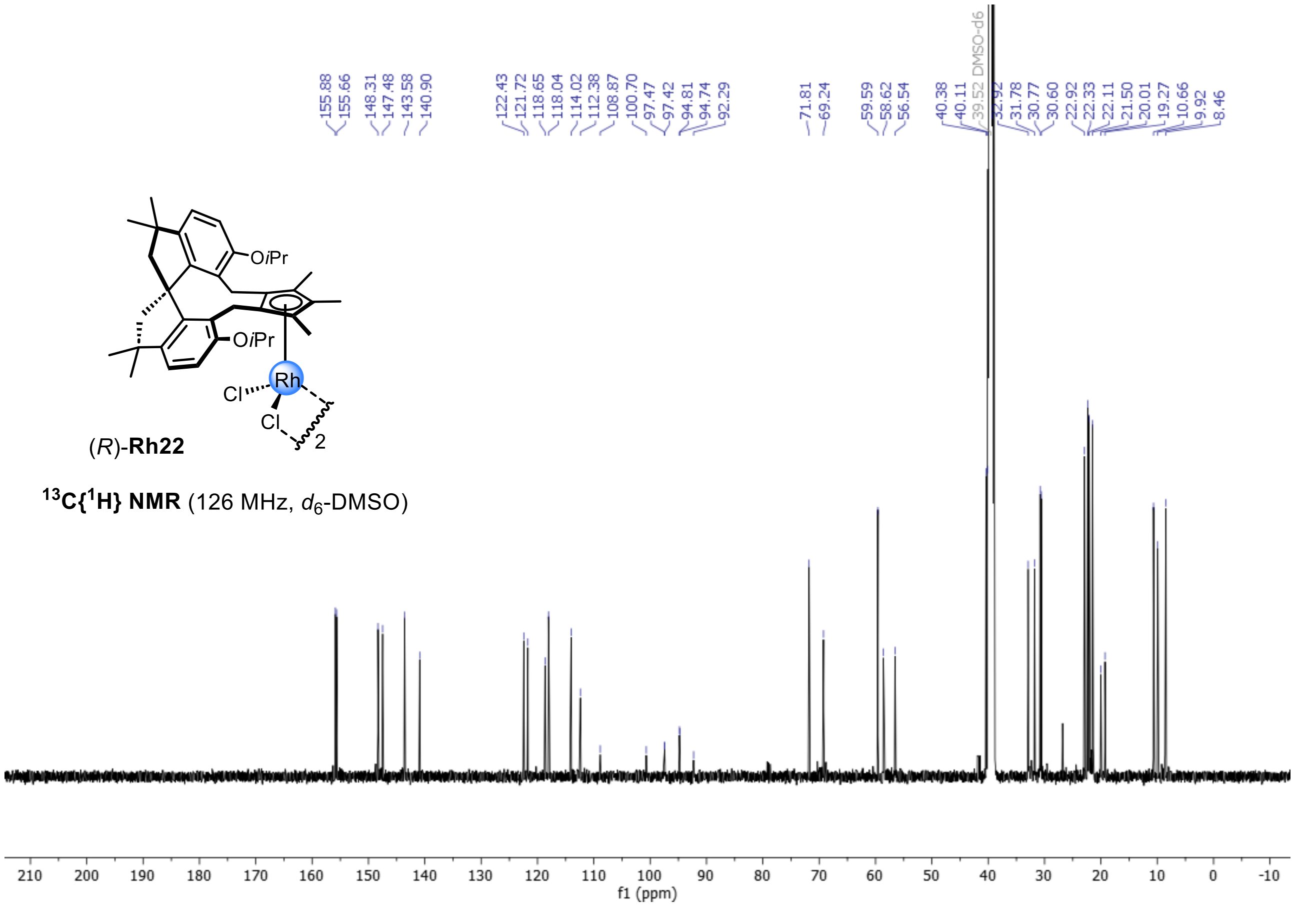

(*R*)-**Rh22**

^{13}C{^{1}H} NMR (126 MHz, d_6-DMSO)

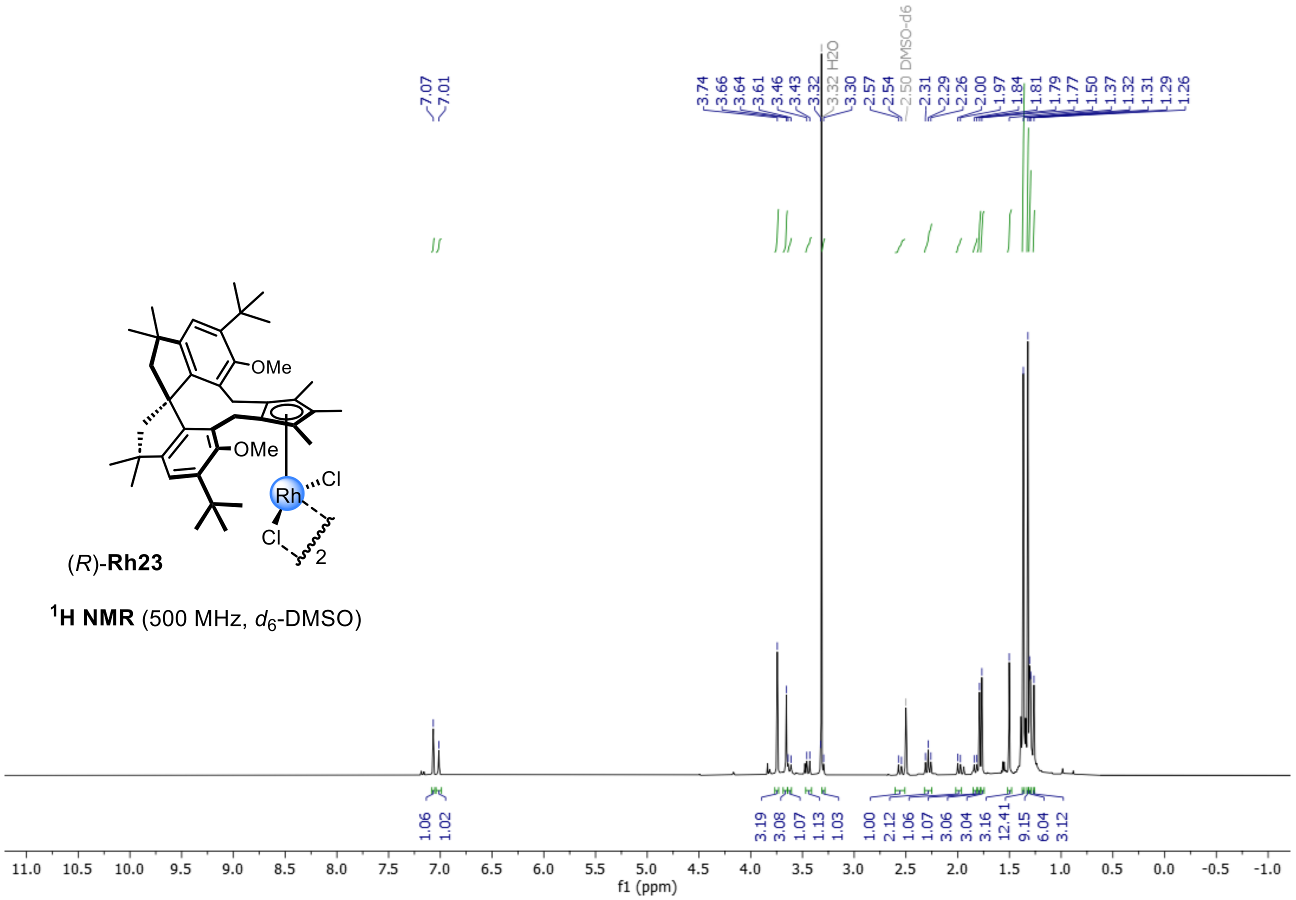

(*R*)-**Rh23**

^{1}H NMR (500 MHz, d_6-DMSO)

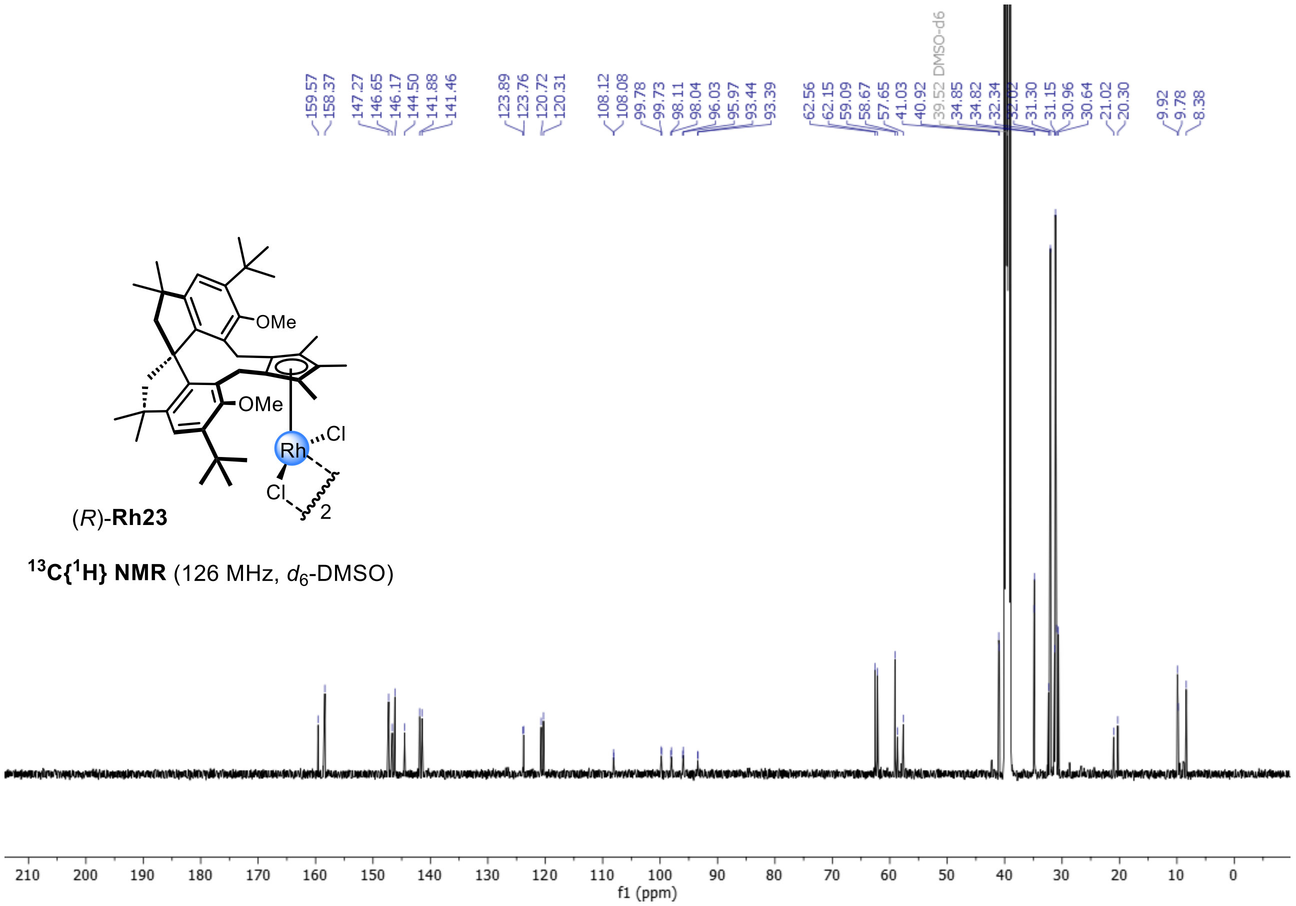

(*R*)-**Rh23**

^{13}C{^{1}H} NMR (126 MHz, d_6-DMSO)

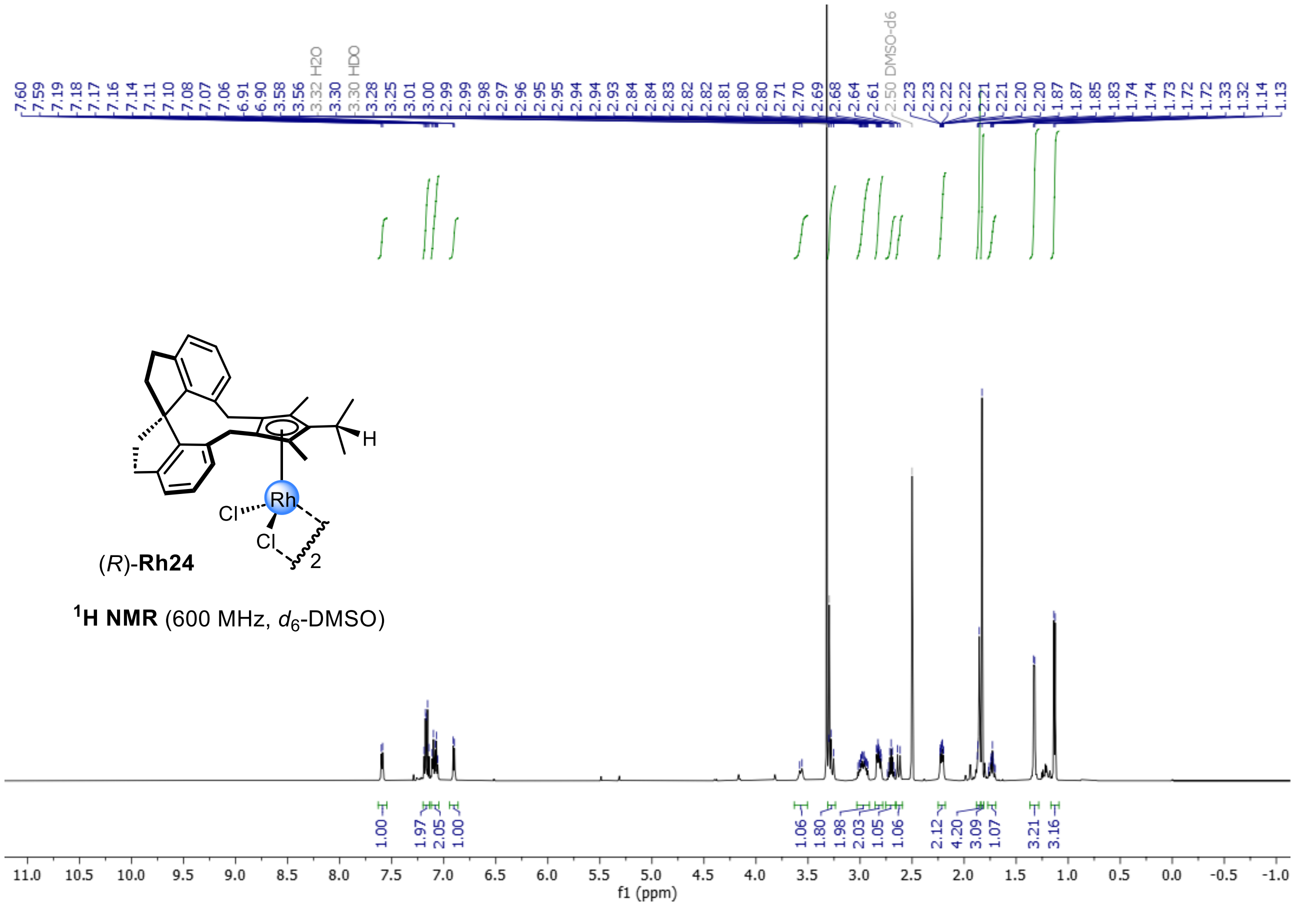

(*R*)-**Rh24**

^{1}H NMR (600 MHz, d_6-DMSO)

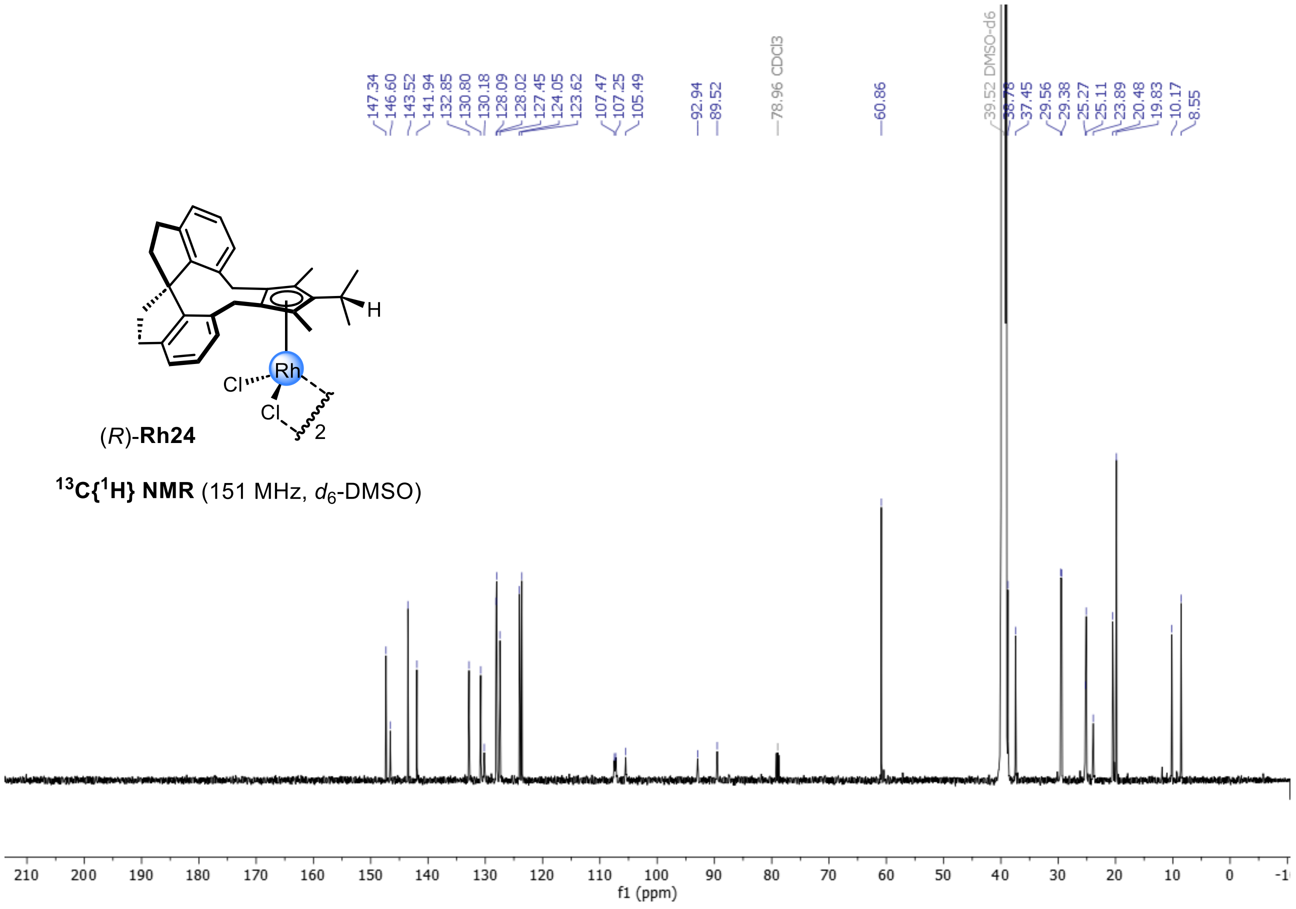

(*R*)-**Rh24**

^{13}C{^{1}H} NMR (151 MHz, d_6-DMSO)

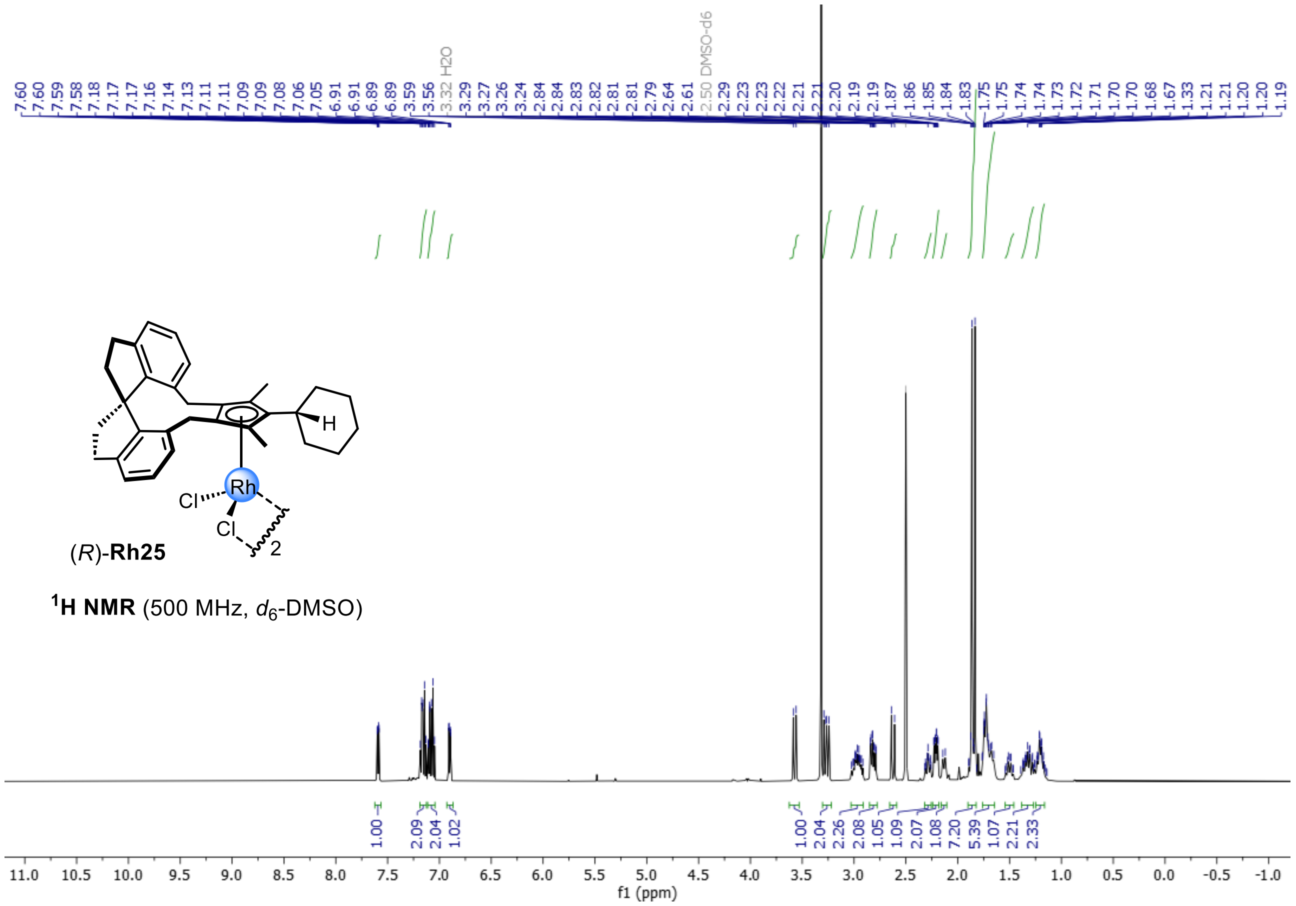
(R)-Rh25
Cl
Rh
H
2
¹H NMR (500 MHz, d6-DMSO)
3.32 H2O
2.50 DMSO-d6
f1 (ppm)

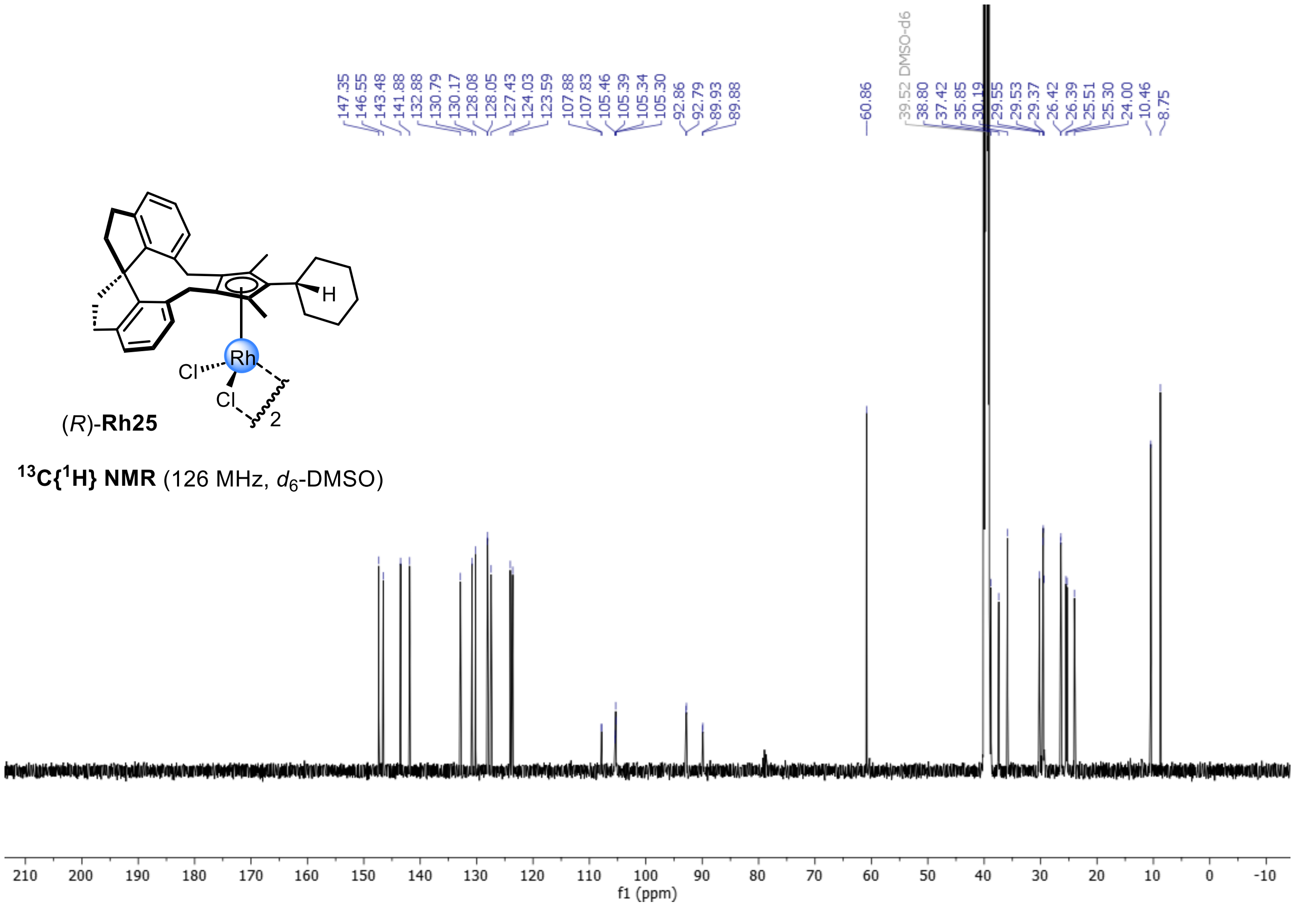
(R)-Rh25
Cl
Rh
H
2
¹³C{¹H} NMR (126 MHz, d6-DMSO)
39.52 DMSO-d6
f1 (ppm)

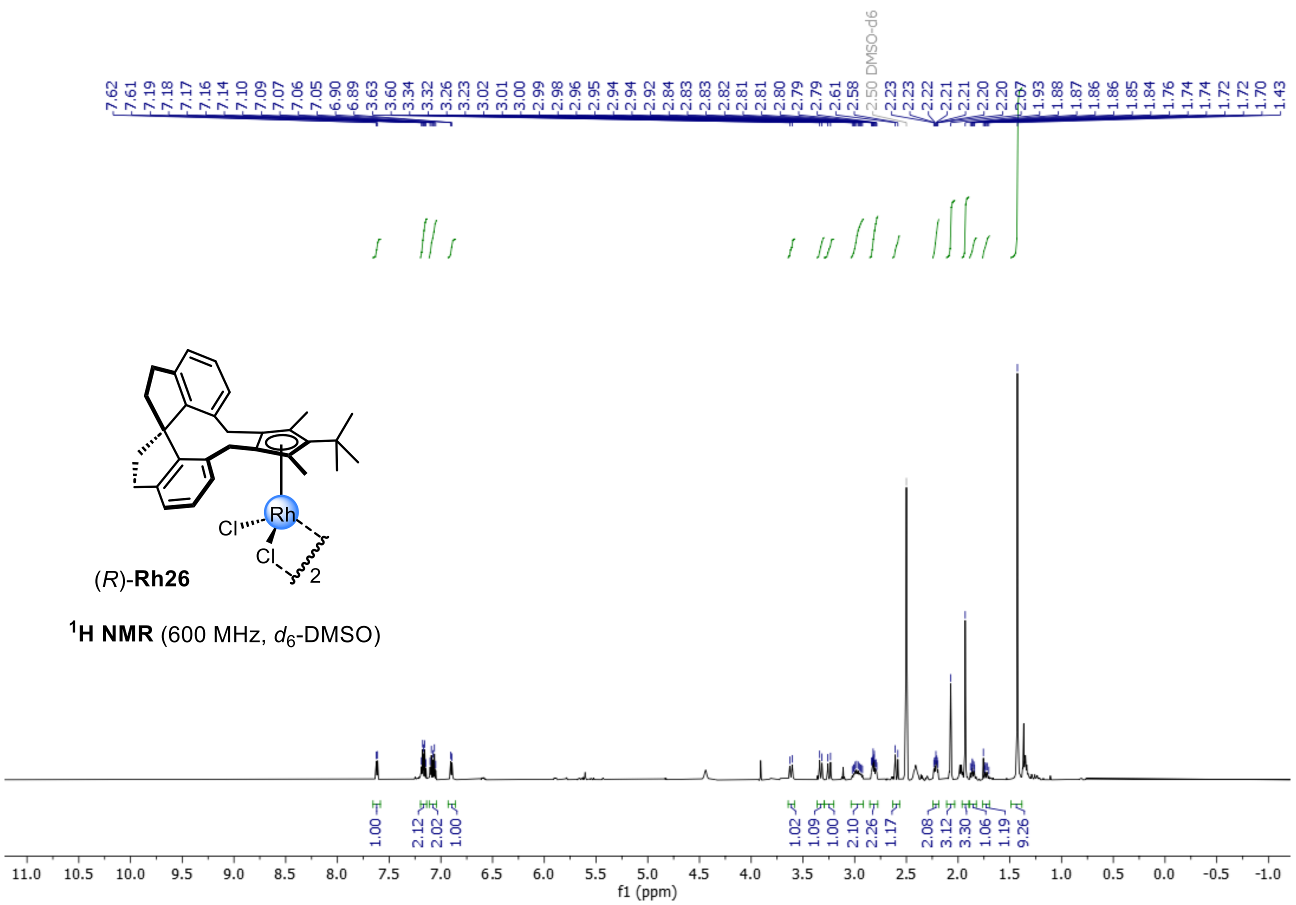

(*R*)-**Rh26**

^{1}H NMR (600 MHz, d_6-DMSO)

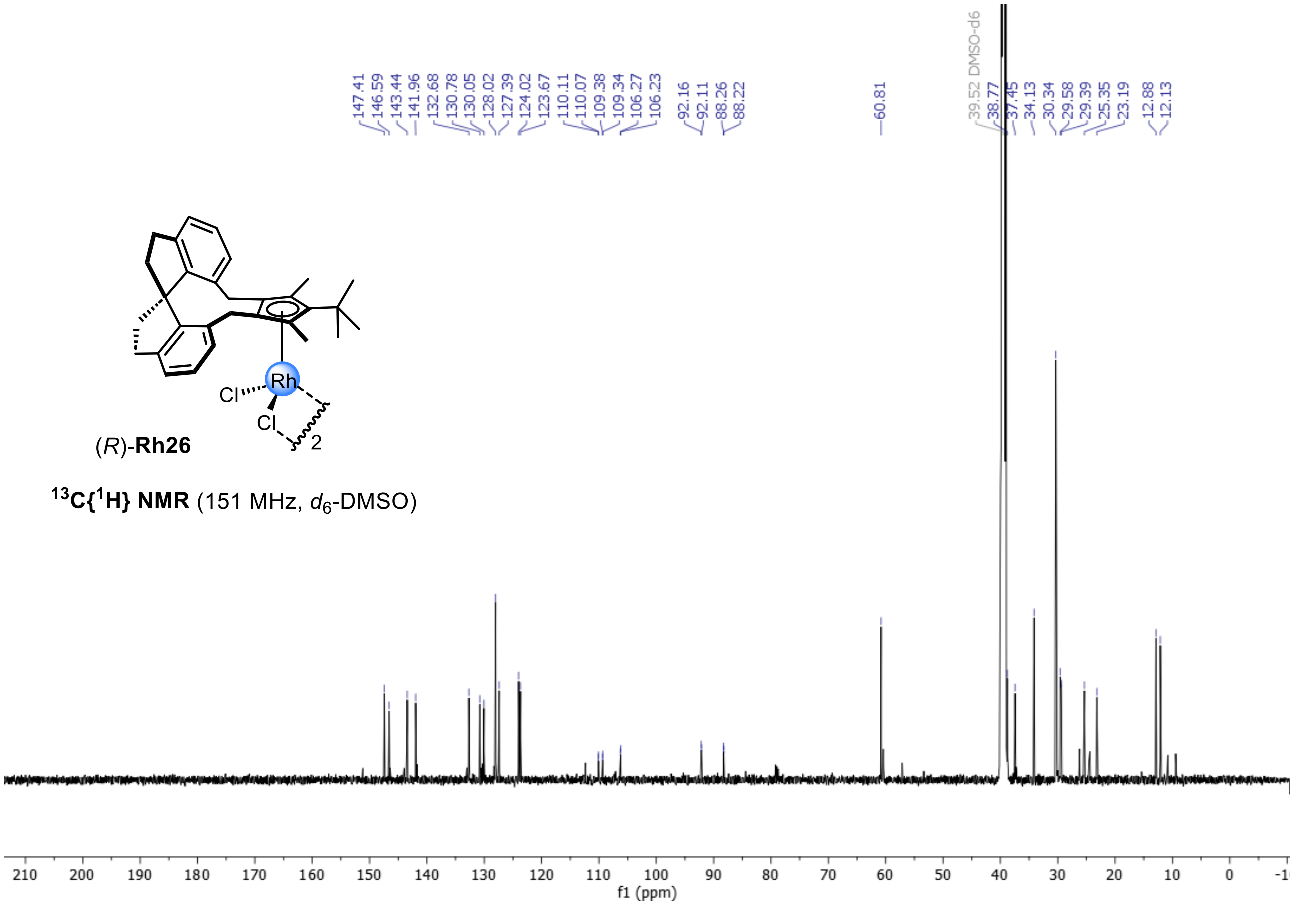

(*R*)-**Rh26**

^{13}C{^{1}H} NMR (151 MHz, d_6-DMSO)

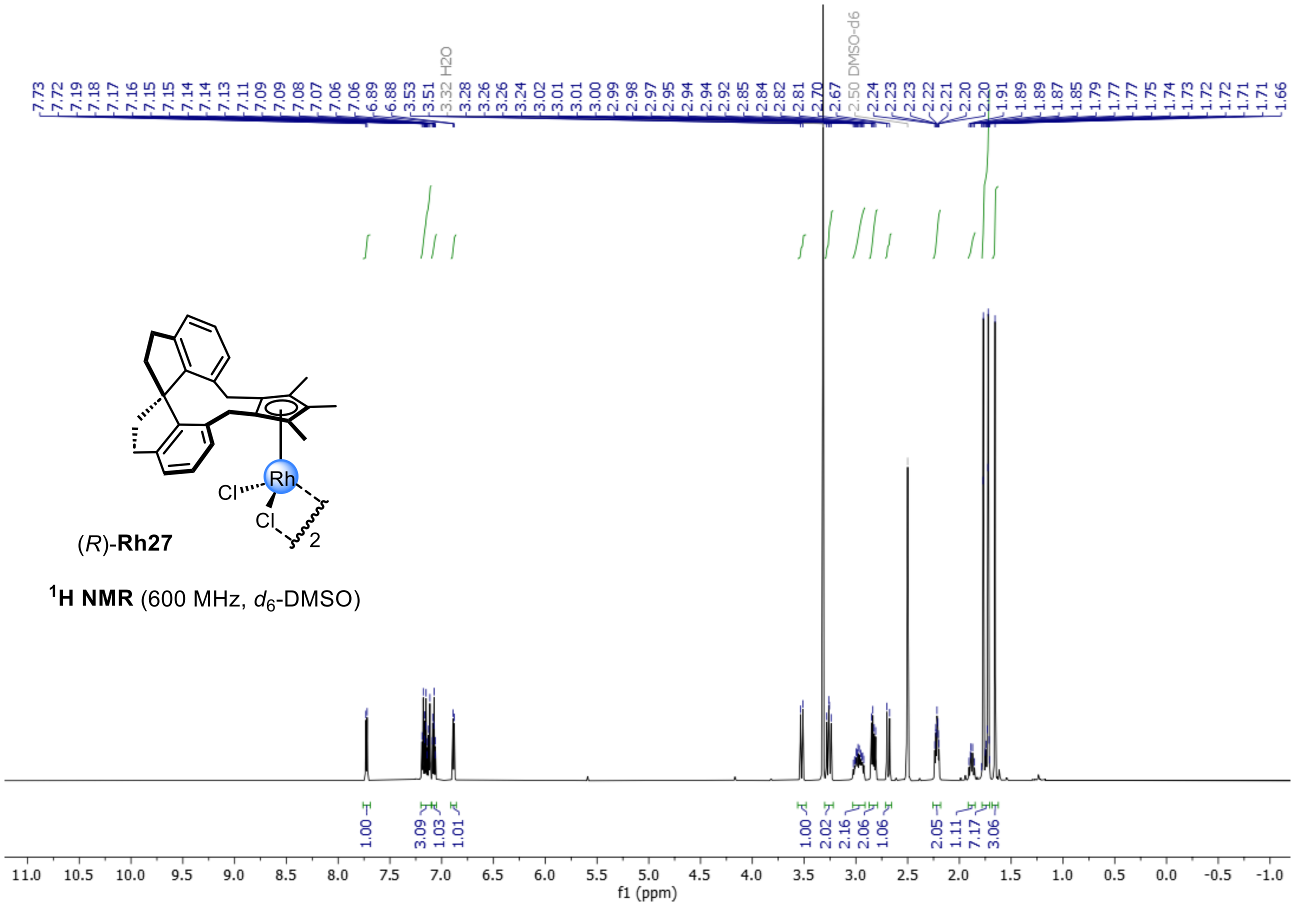
(R)-Rh27
1H NMR (600 MHz, d6-DMSO)
Rh
Cl
Cl
2
7.73 7.72 7.19 7.18 7.17 7.16 7.15 7.14 7.14 7.13 7.11 7.09 7.09 7.08 7.07 7.06 7.06 6.89 6.88 3.53 3.51 3.32 H2O 3.28 3.26 3.26 3.24 3.02 3.01 3.01 3.00 2.99 2.98 2.97 2.95 2.94 2.94 2.92 2.85 2.84 2.82 2.81 2.70 2.67 2.50 DMSO-d6 2.24 2.23 2.23 2.22 2.21 2.20 2.20 1.91 1.89 1.89 1.87 1.85 1.79 1.77 1.77 1.75 1.74 1.73 1.72 1.72 1.71 1.71 1.66
1.00 3.09 1.03 1.01 1.00 2.02 2.16 2.06 1.06 2.05 1.11 7.17 3.06
f1 (ppm)

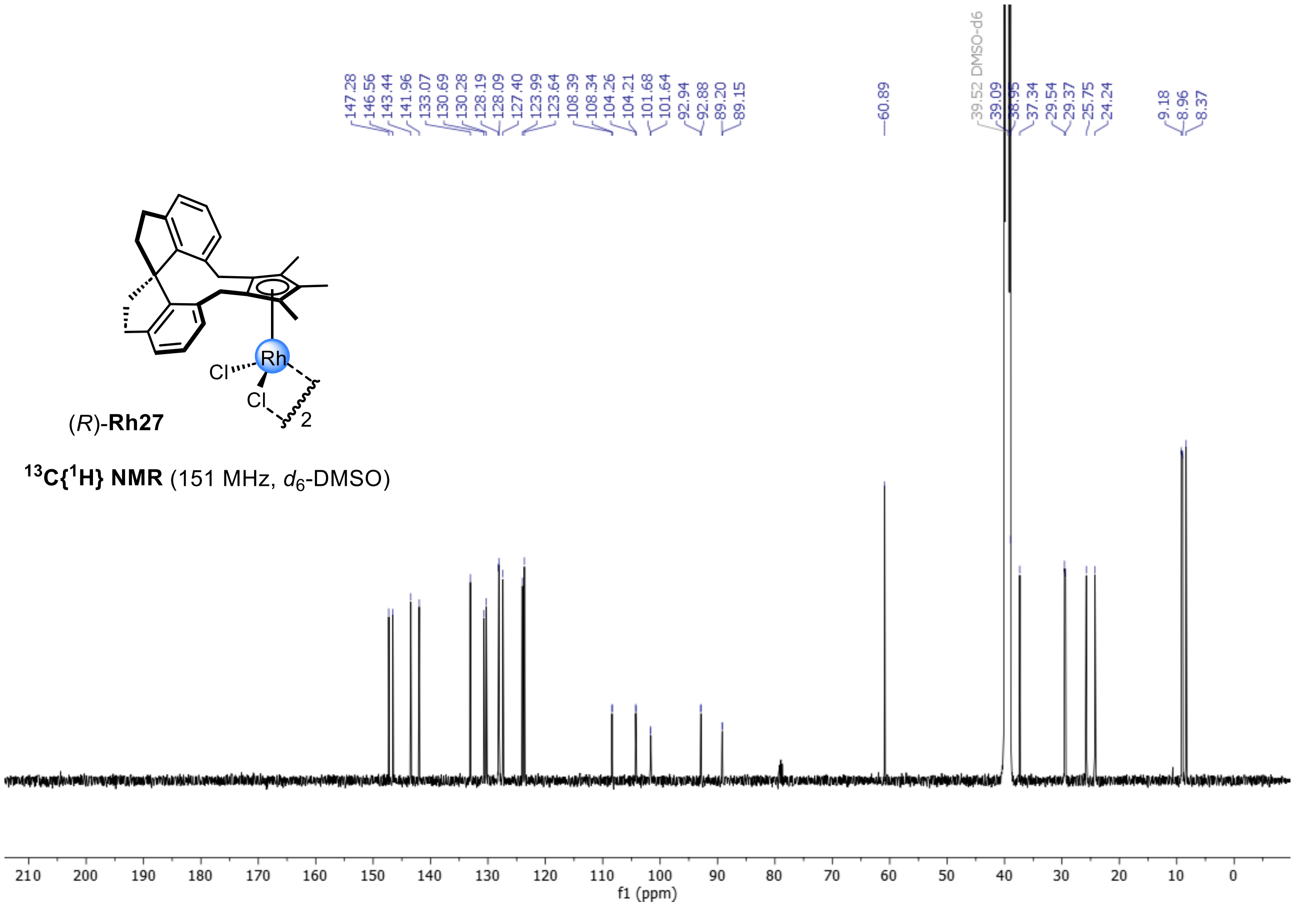
(R)-Rh27
13C{1H} NMR (151 MHz, d6-DMSO)
Rh
Cl
Cl
2
147.28 146.56 143.44 141.96 133.07 130.69 130.28 128.19 128.09 127.40 123.99 123.64 108.39 108.34 104.26 104.21 101.68 101.64 92.94 92.88 89.20 89.15 60.89 39.52 DMSO-d6 39.09 38.95 37.34 29.54 29.37 25.75 24.24 9.18 8.96 8.37
f1 (ppm)

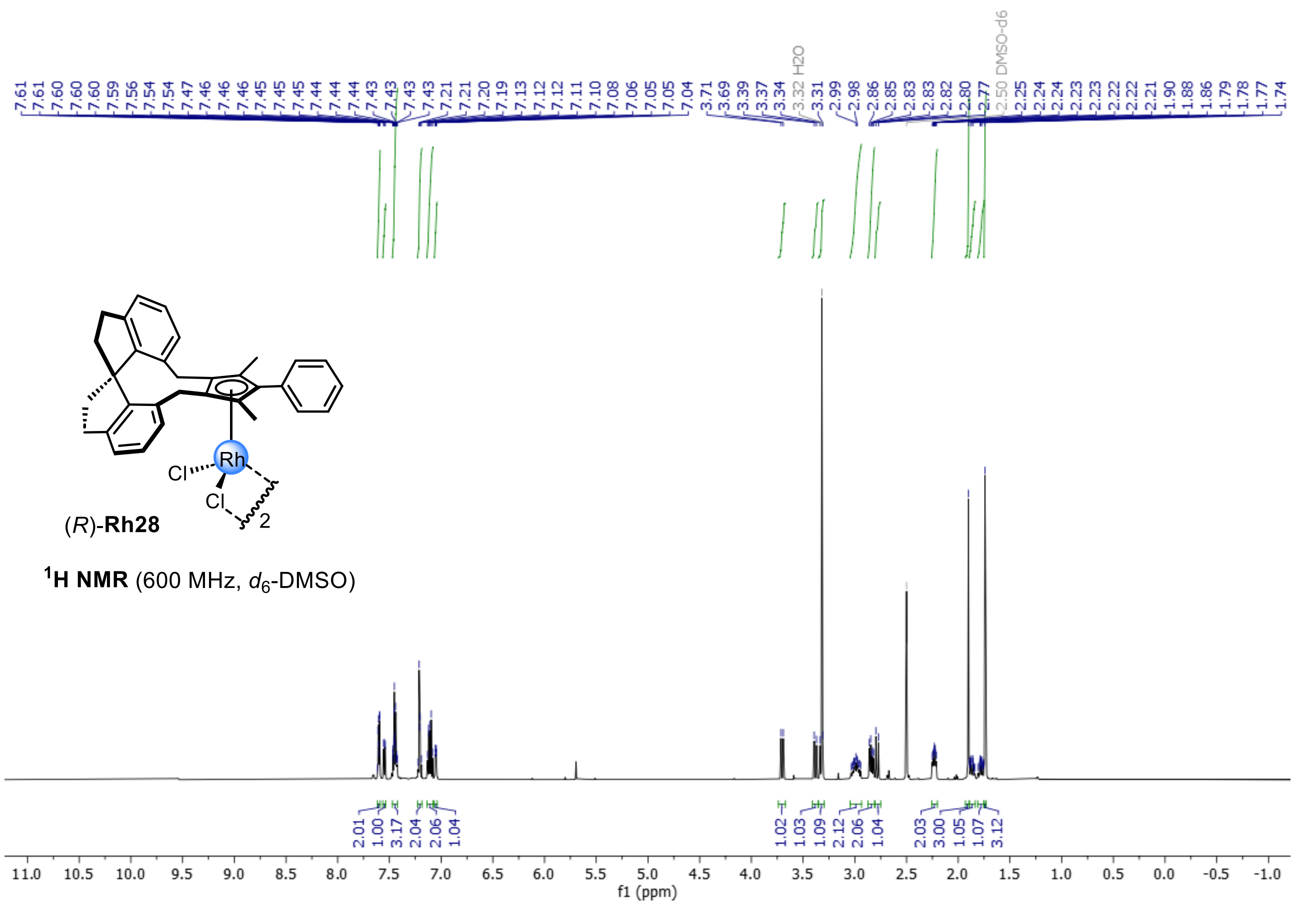
(R)-Rh28
^{1}H NMR (600 MHz, d_6-DMSO)
f1 (ppm)

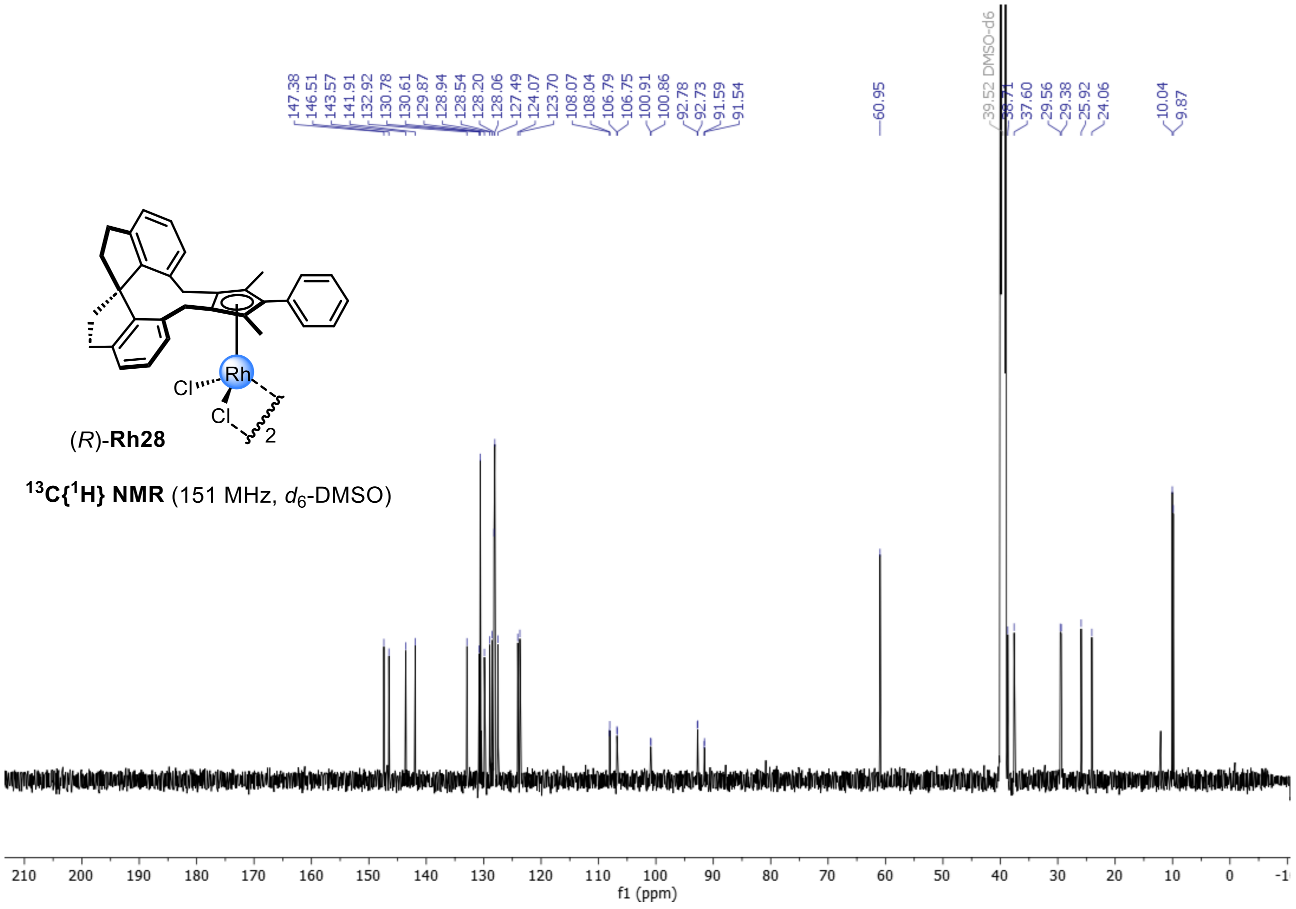
(R)-Rh28
^{13}C{^{1}H} NMR (151 MHz, d_6-DMSO)
f1 (ppm)

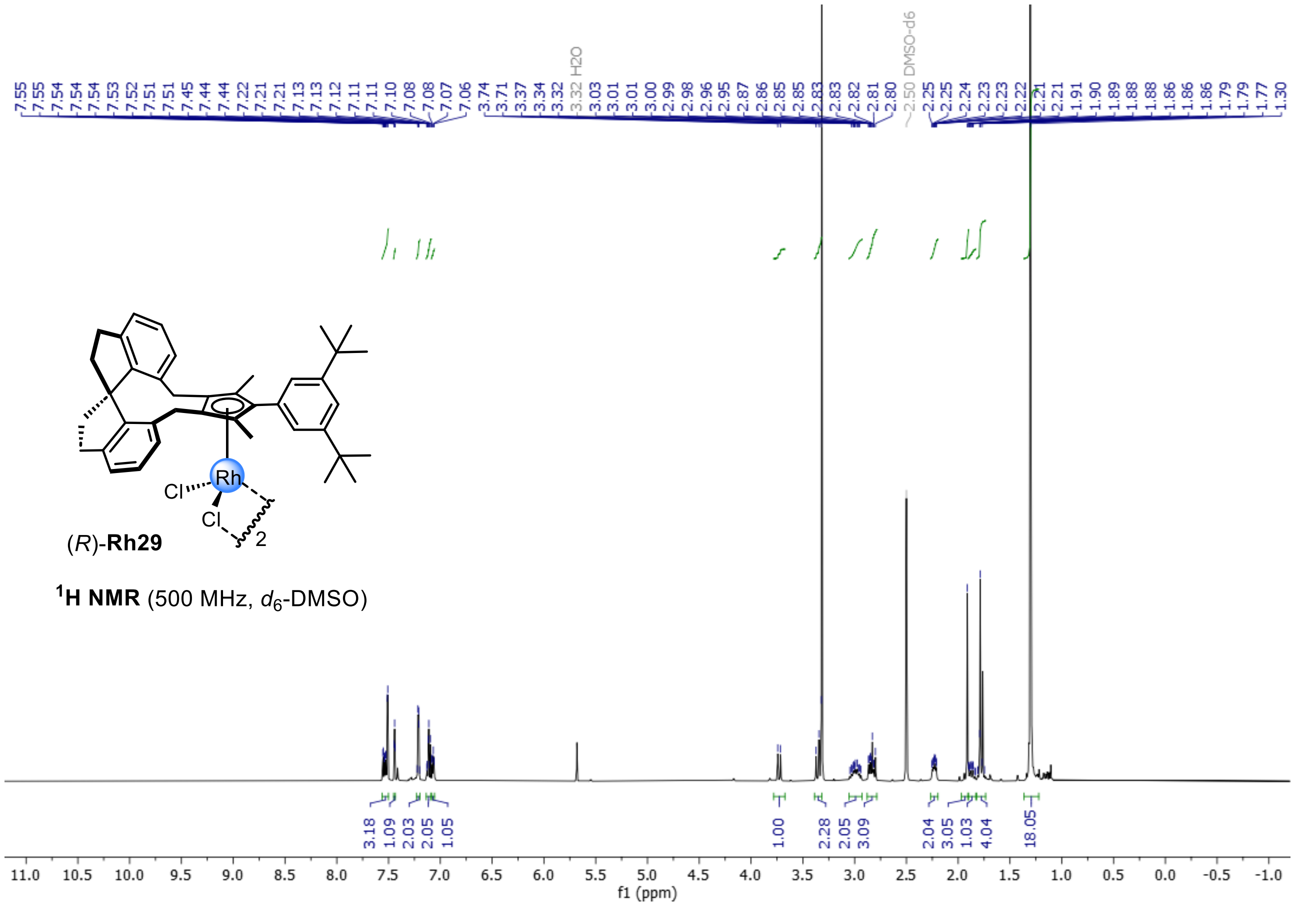

(*R*)-**Rh29**

^{1}H NMR (500 MHz, d_6-DMSO)

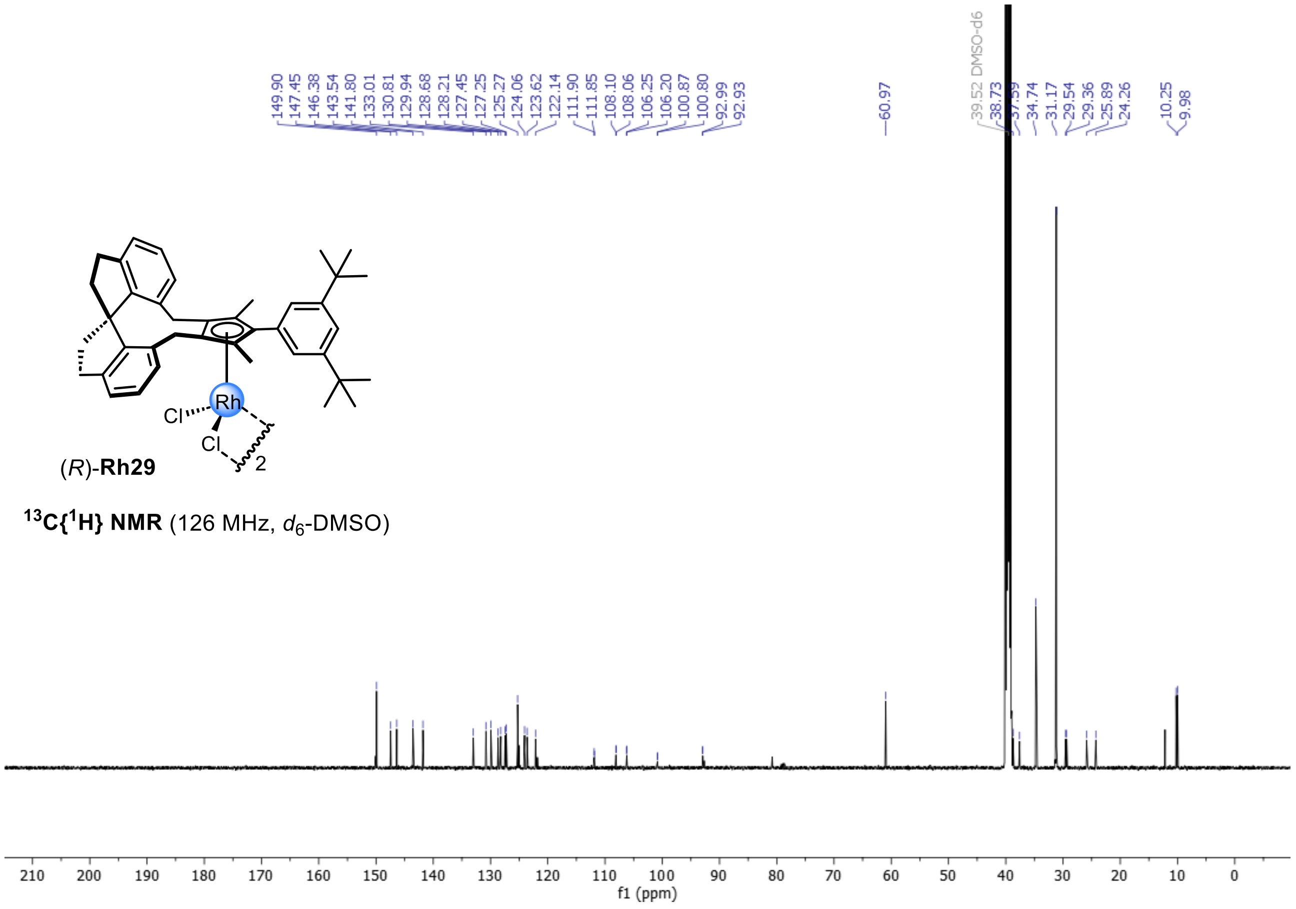

(*R*)-**Rh29**

^{13}C{^{1}H} NMR (126 MHz, d_6-DMSO)

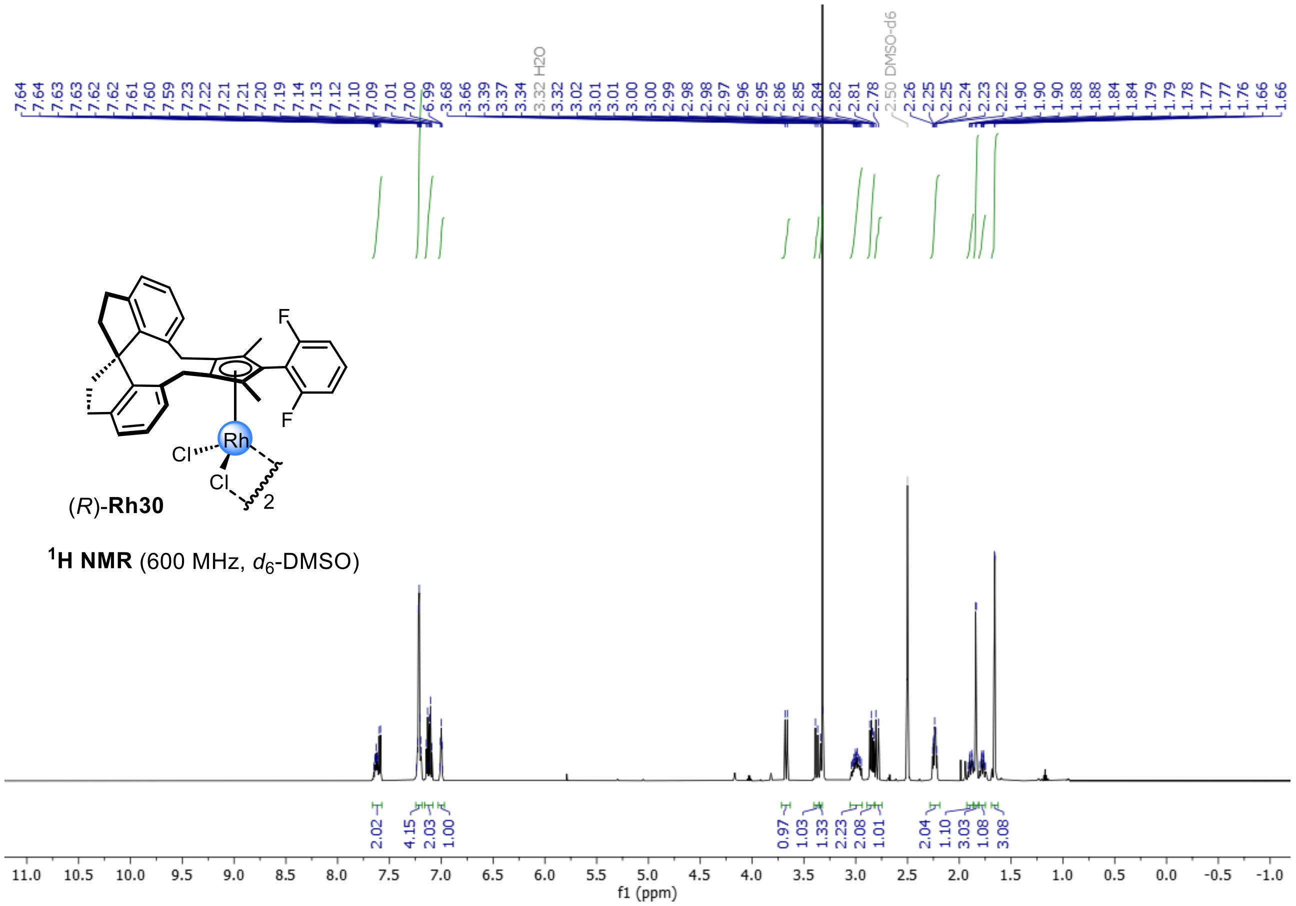

(*R*)-**Rh30**

^{1}H NMR (600 MHz, d_6-DMSO)

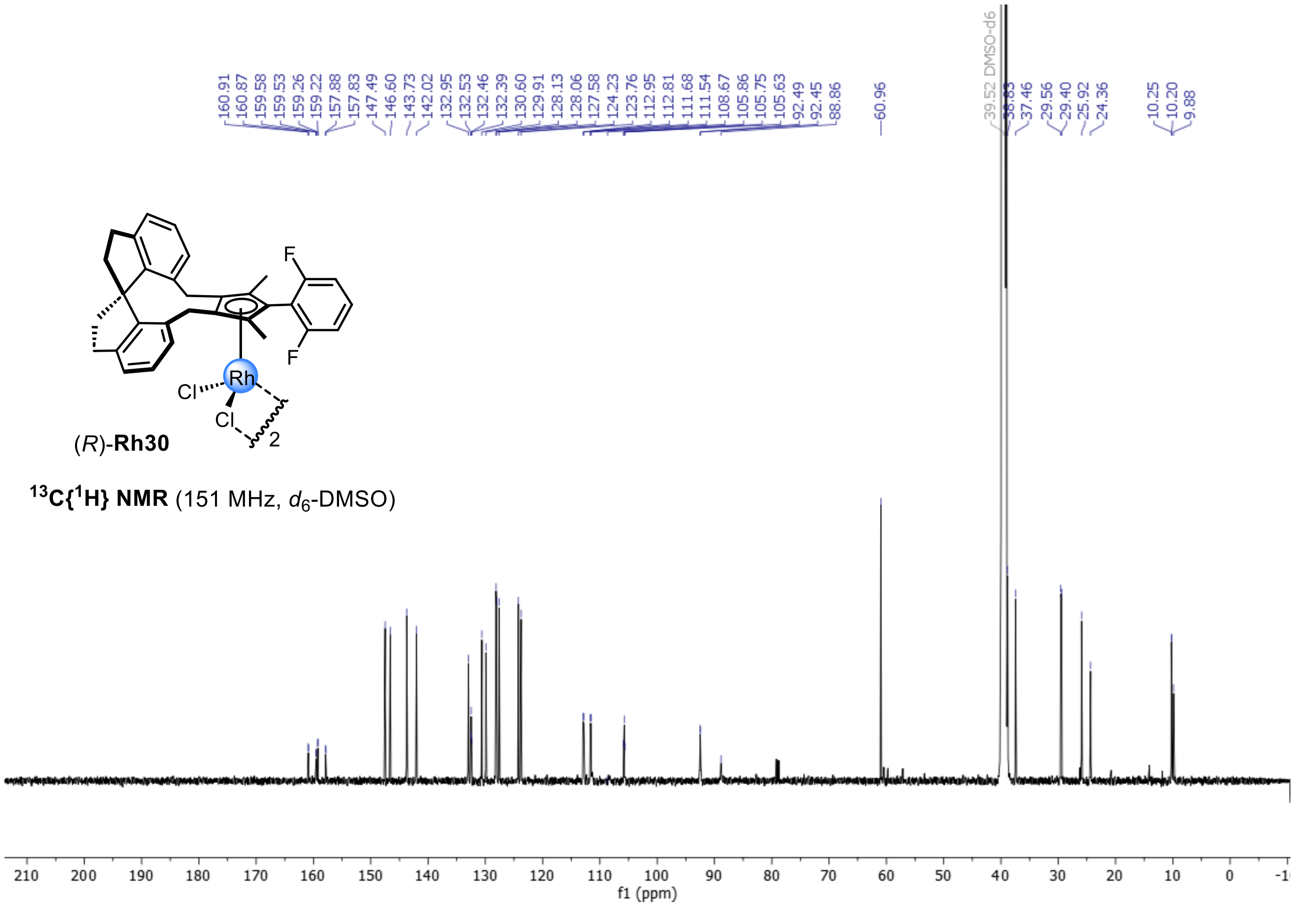

(*R*)-**Rh30**

^{13}C{^{1}H} NMR (151 MHz, d_6-DMSO)

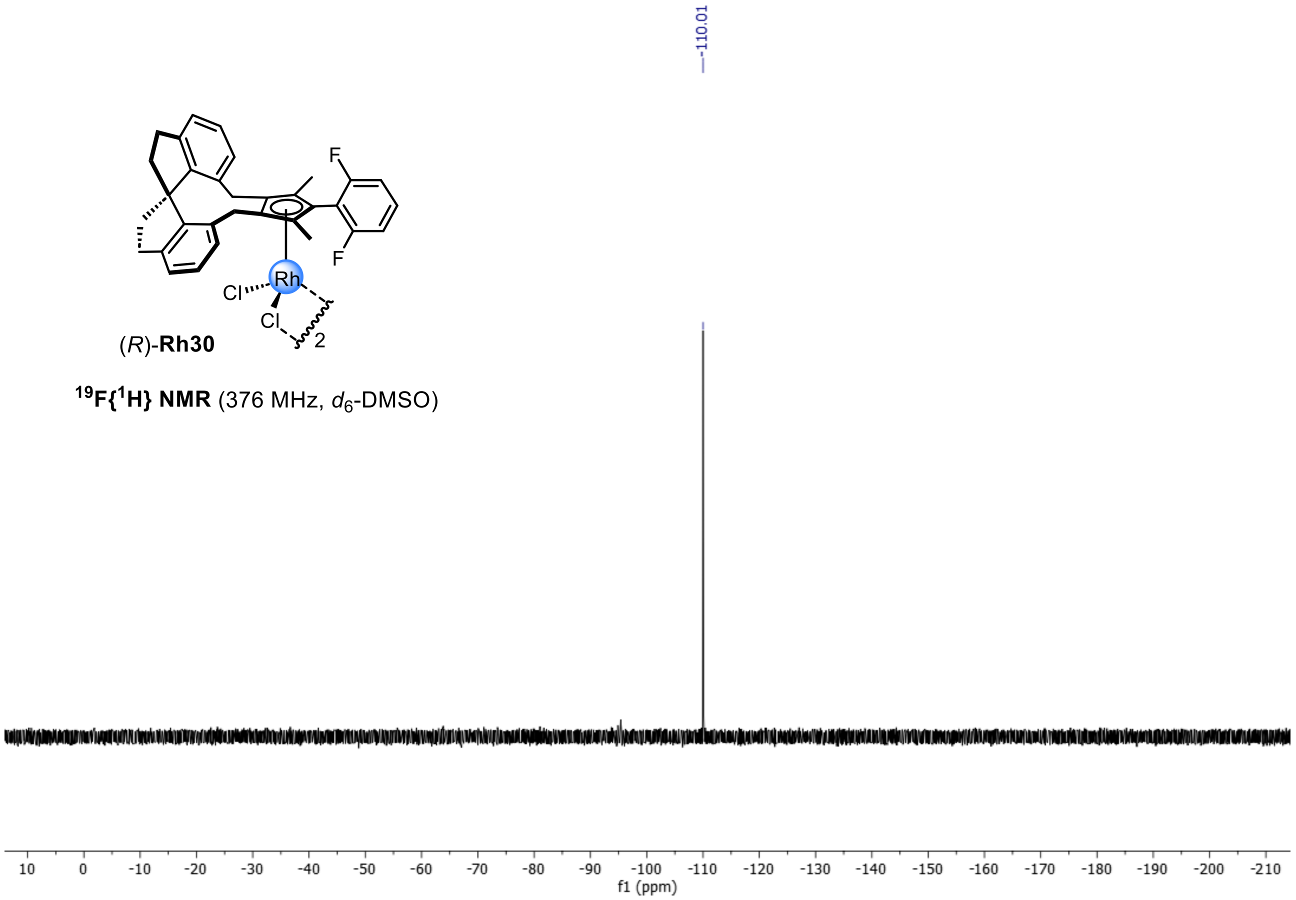
-110.01
F
F
Rh
Cl
Cl
2
(R)-Rh30
19F{1H} NMR (376 MHz, d6-DMSO)
10 0 -10 -20 -30 -40 -50 -60 -70 -80 -90 -100 -110 -120 -130 -140 -150 -160 -170 -180 -190 -200 -210
f1 (ppm)

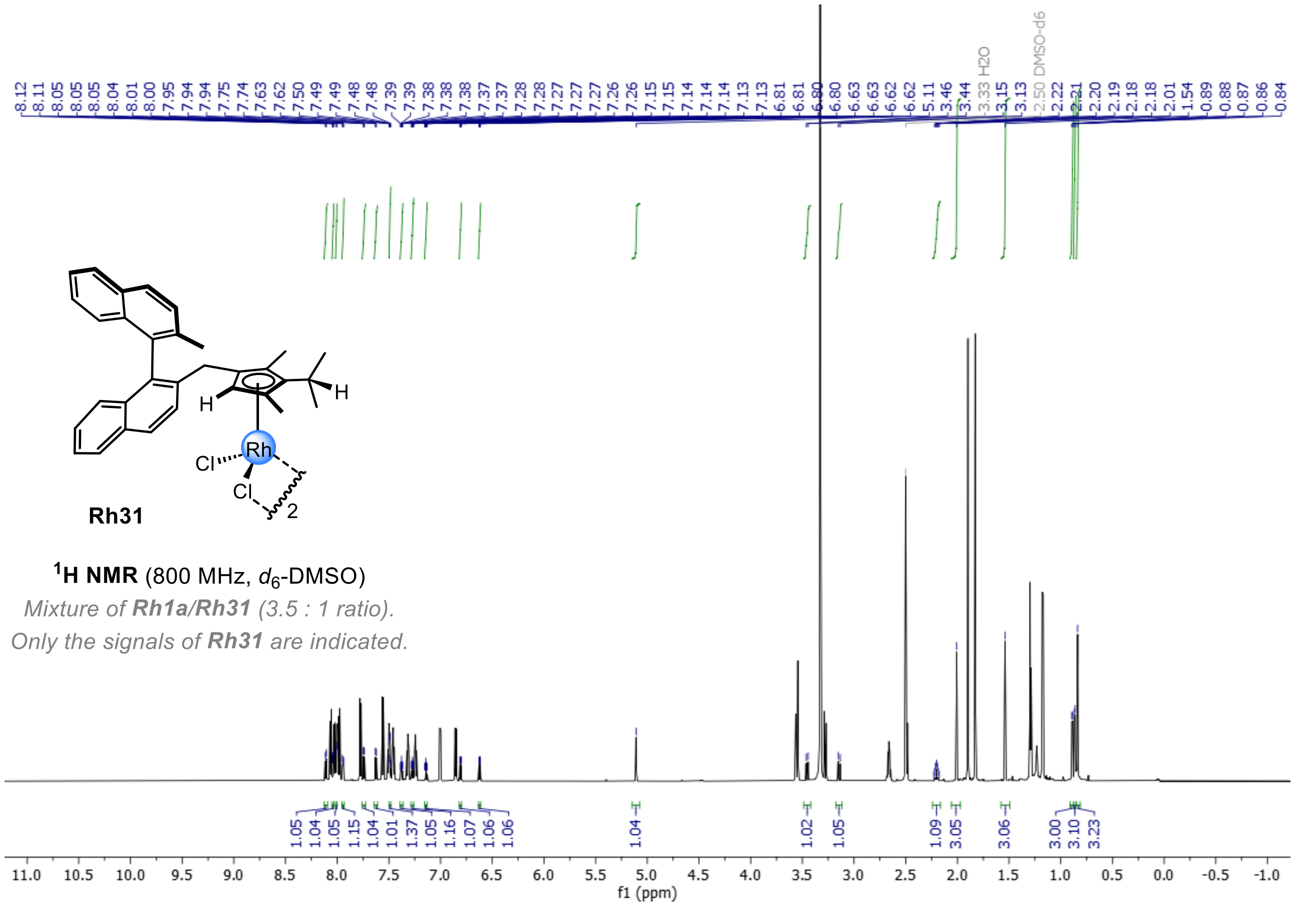

^{1}H NMR (800 MHz, d_6-DMSO)

*Mixture of **Rh1a/Rh31** (3.5 : 1 ratio).*

*Only the signals of **Rh31** are indicated.*

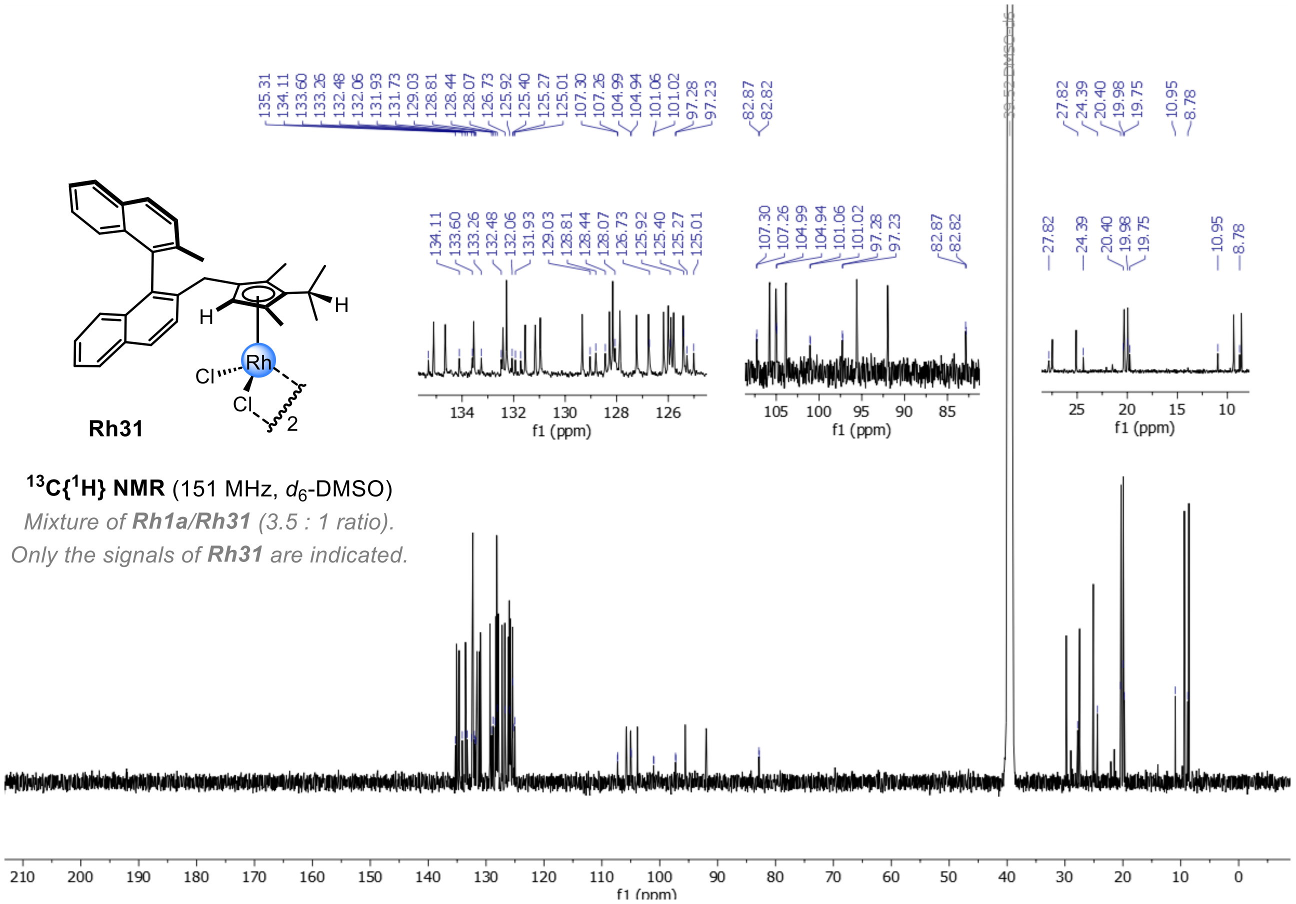

^{13}C{^{1}H} NMR (151 MHz, d_6-DMSO)

*Mixture of **Rh1a/Rh31** (3.5 : 1 ratio).*

*Only the signals of **Rh31** are indicated.*

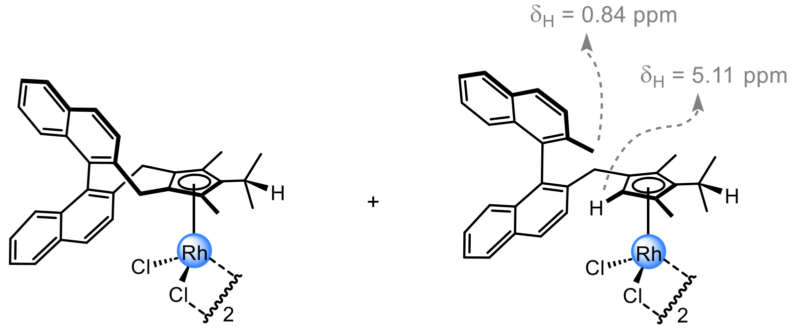

Rh1a (3.5 : 1 ratio) **Rh31**

HSQC (600 MHz, d_6-DMSO)

*Key interactions of the minor complex **Rh31** are indicated.*

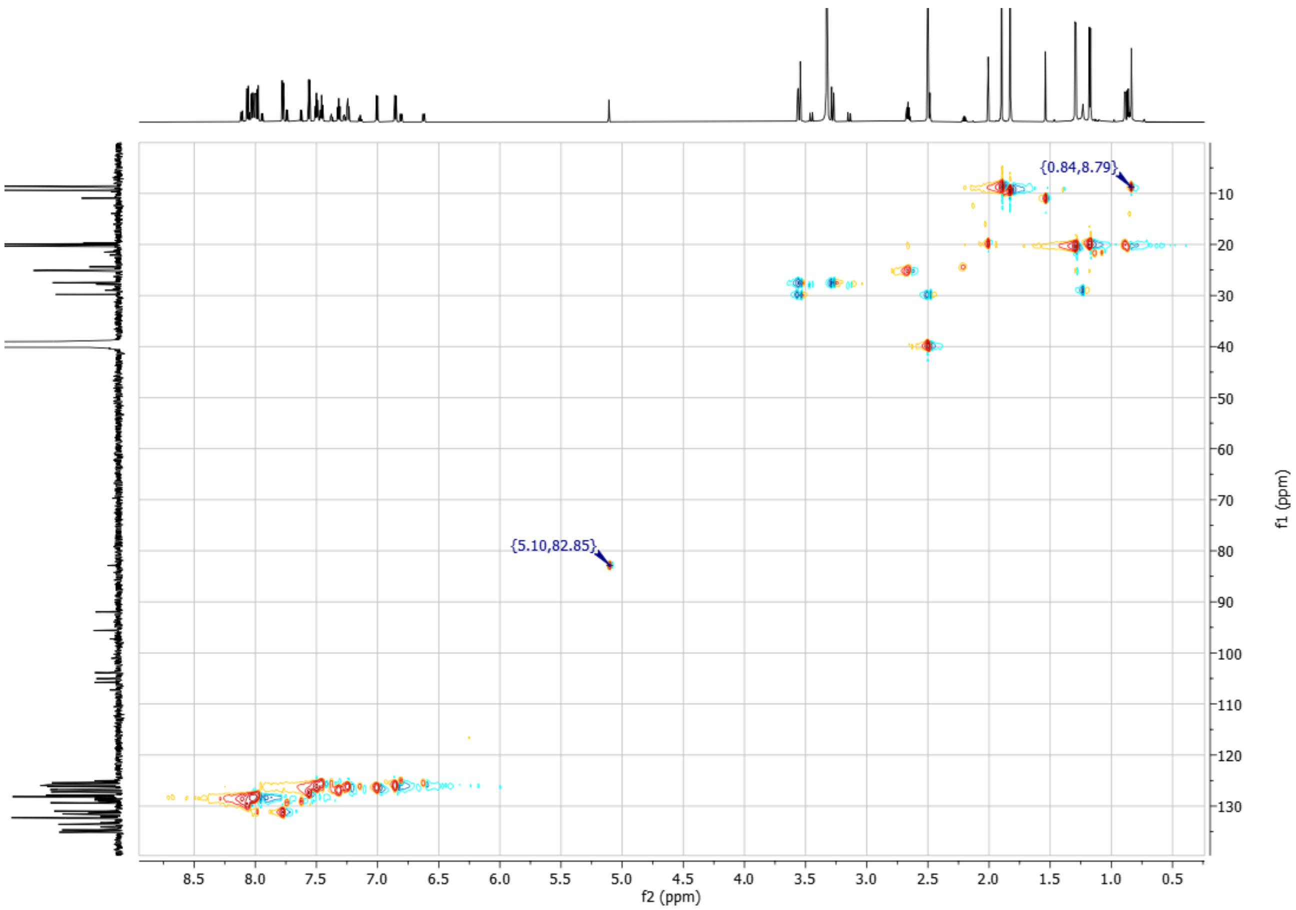

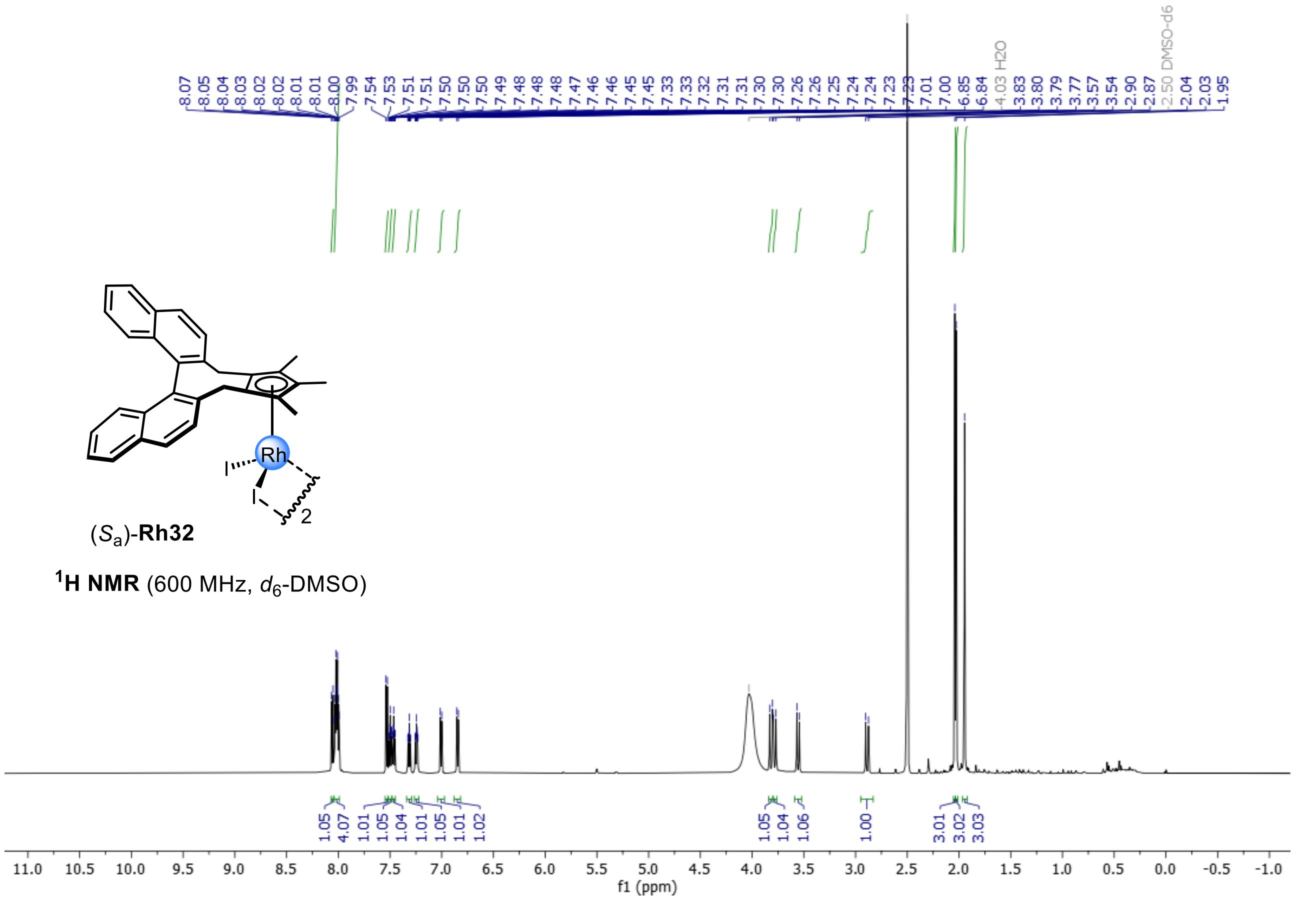

8.07 8.05 8.04 8.03 8.02 8.02 8.01 8.00 7.99 7.54 7.53 7.51 7.51 7.50 7.50 7.50 7.49 7.48 7.48 7.47 7.46 7.45 7.45 7.33 7.33 7.32 7.31 7.30 7.26 7.26 7.25 7.24 7.24 7.23 7.23 7.01 7.00 6.85 6.84
4.03 H2O
3.83 3.80 3.79 3.77 3.57 3.54 2.90 2.87
2.50 DMSO-d6
2.04 2.03 1.95
Rh
I
2
(Sa)-Rh32
1H NMR (600 MHz, d6-DMSO)
1.05 4.07 1.01 1.05 1.04 1.01 1.05 1.01 1.02 1.05 1.04 1.06 1.00 3.01 3.02 3.03
11.0 10.5 10.0 9.5 9.0 8.5 8.0 7.5 7.0 6.5 6.0 5.5 5.0 4.5 4.0 3.5 3.0 2.5 2.0 1.5 1.0 0.5 0.0 -0.5 -1.0
f1 (ppm)

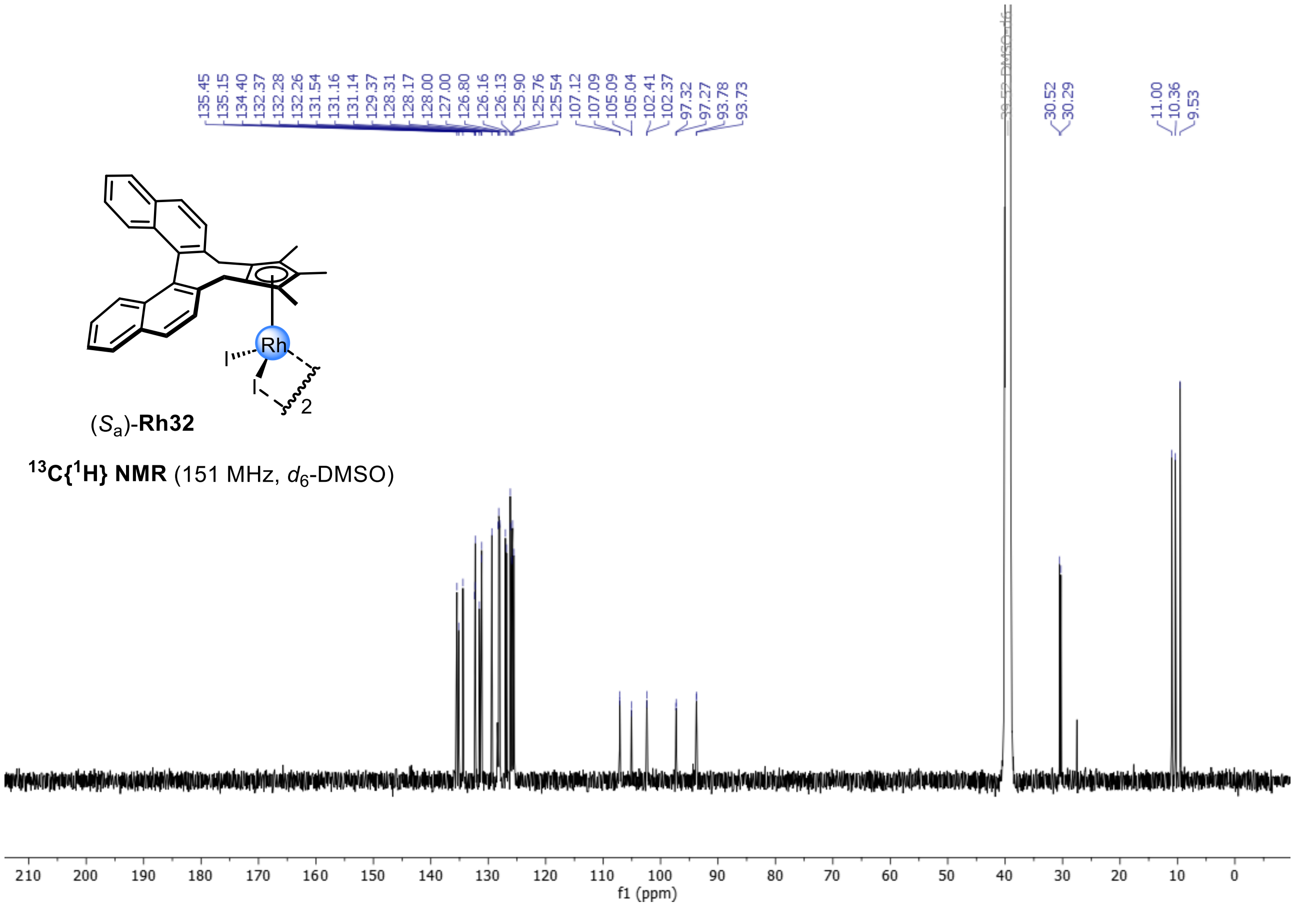

135.45 135.15 134.40 132.37 132.28 132.26 131.54 131.16 131.14 129.37 128.31 128.17 128.00 127.00 126.80 126.16 126.13 125.90 125.76 125.54 107.12 107.09 105.09 105.04 102.41 102.37 97.32 97.27 93.78 93.73
39.52 DMSO-d6
30.52 30.29
11.00 10.36 9.53
Rh
I
2
(Sa)-Rh32
13C{1H} NMR (151 MHz, d6-DMSO)
210 200 190 180 170 160 150 140 130 120 110 100 90 80 70 60 50 40 30 20 10 0
f1 (ppm)

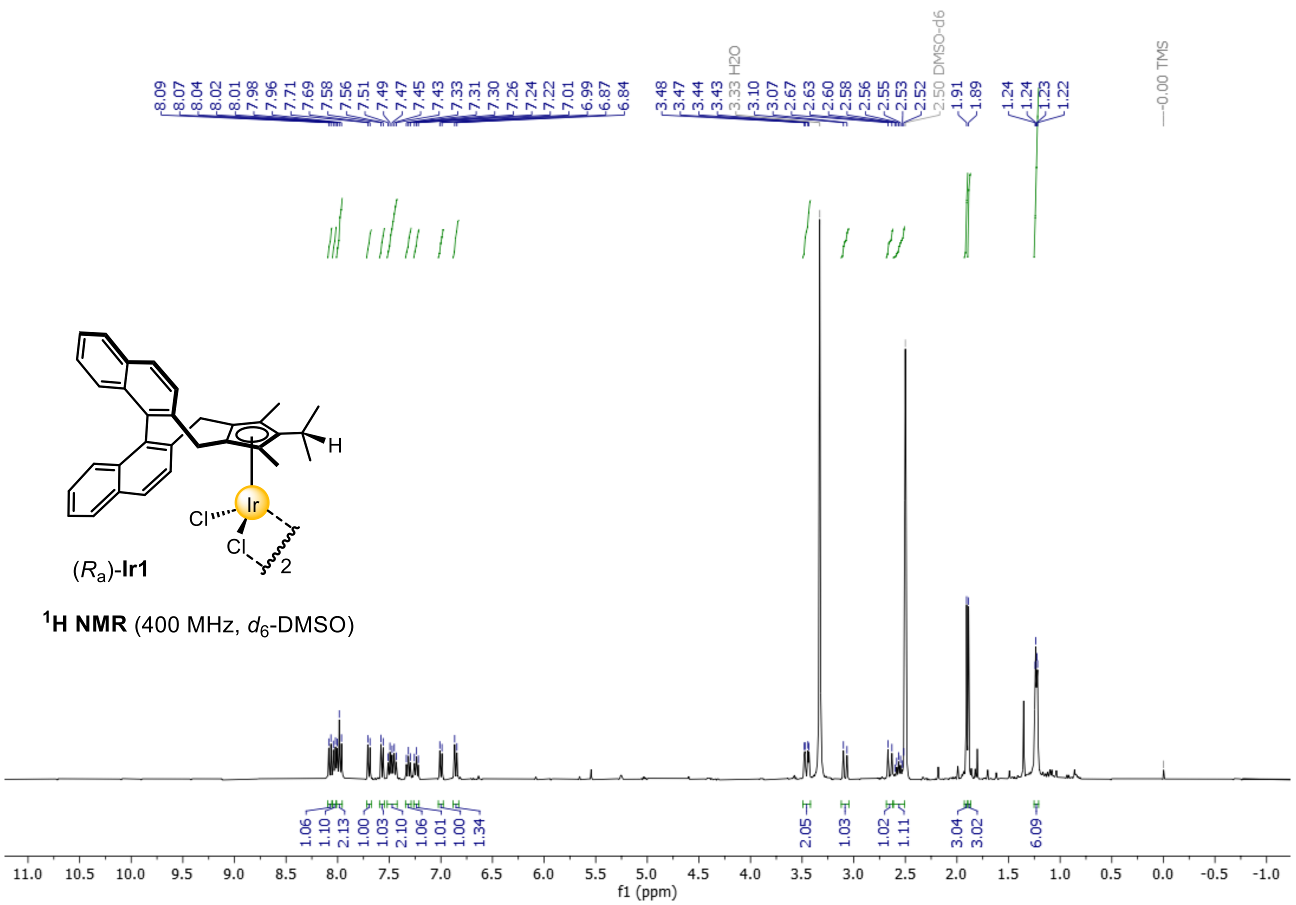
8.09 8.07 8.04 8.02 8.01 7.98 7.96 7.71 7.69 7.58 7.56 7.51 7.49 7.47 7.45 7.43 7.33 7.31 7.30 7.26 7.24 7.22 7.01 6.99 6.87 6.84
3.48 3.47 3.44 3.43 3.33 H2O 3.10 3.07 2.67 2.63 2.60 2.58 2.56 2.55 2.53 2.52 2.50 DMSO-d6 1.91 1.89 1.24 1.24 1.23 1.22
0.00 TMS
Cl
Ir
Cl
2
H
(Ra)-Ir1
1H NMR (400 MHz, d6-DMSO)
1.06 1.10 2.13 1.00 1.03 2.10 1.06 1.01 1.00 1.34 2.05 1.03 1.02 1.11 3.04 3.02 6.09
11.0 10.5 10.0 9.5 9.0 8.5 8.0 7.5 7.0 6.5 6.0 5.5 5.0 4.5 4.0 3.5 3.0 2.5 2.0 1.5 1.0 0.5 0.0 -0.5 -1.0
f1 (ppm)

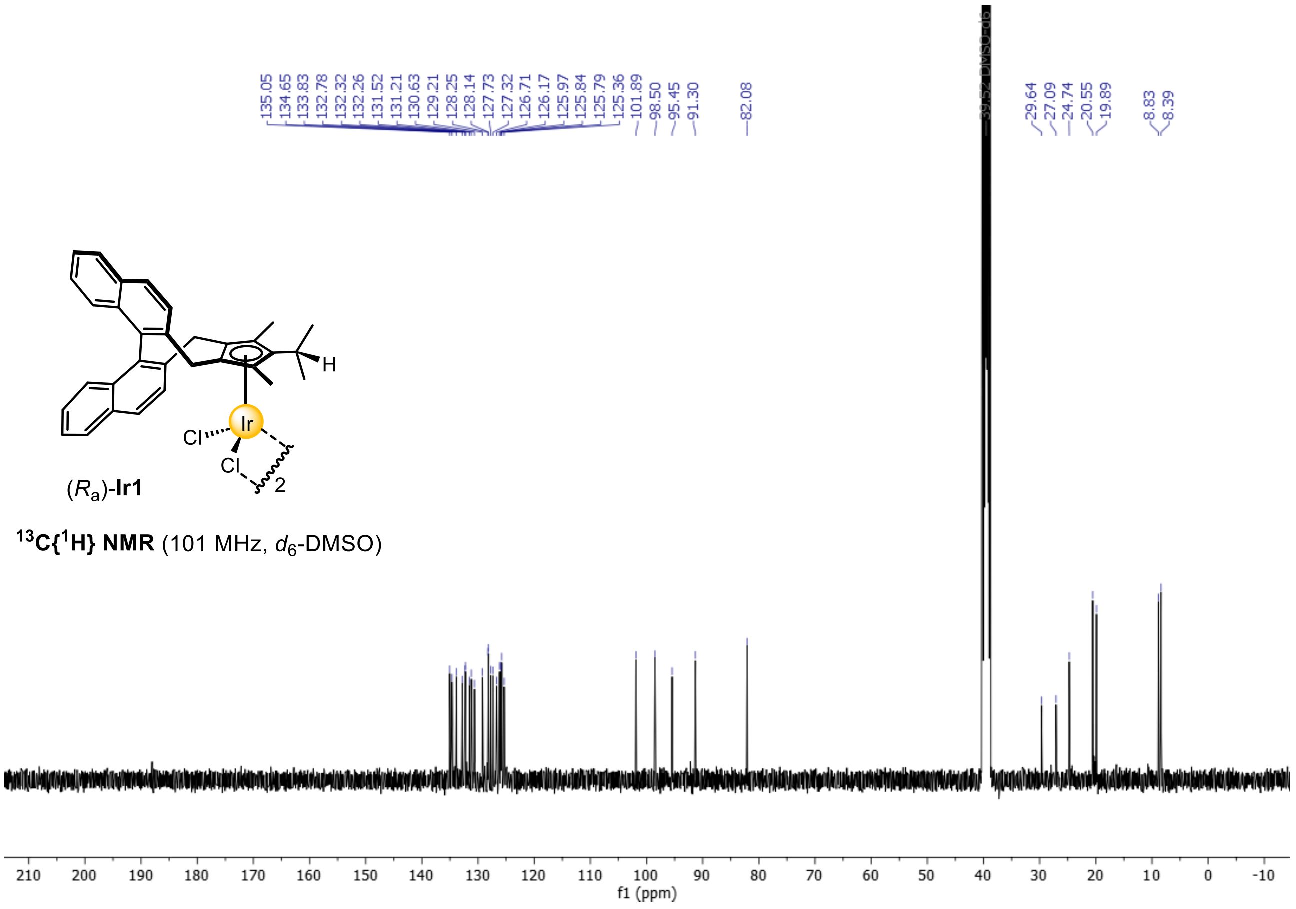
135.05 134.65 133.83 132.78 132.32 132.26 131.52 131.21 130.63 129.21 128.25 128.14 127.73 127.32 126.71 126.17 125.97 125.84 125.79 125.36 101.89 98.50 95.45 91.30
82.08
39.52 DMSO-d6
29.64 27.09 24.74 20.55 19.89 8.83 8.39
Cl
Ir
Cl
2
H
(Ra)-Ir1
13C{1H} NMR (101 MHz, d6-DMSO)
210 200 190 180 170 160 150 140 130 120 110 100 90 80 70 60 50 40 30 20 10 0 -10
f1 (ppm)

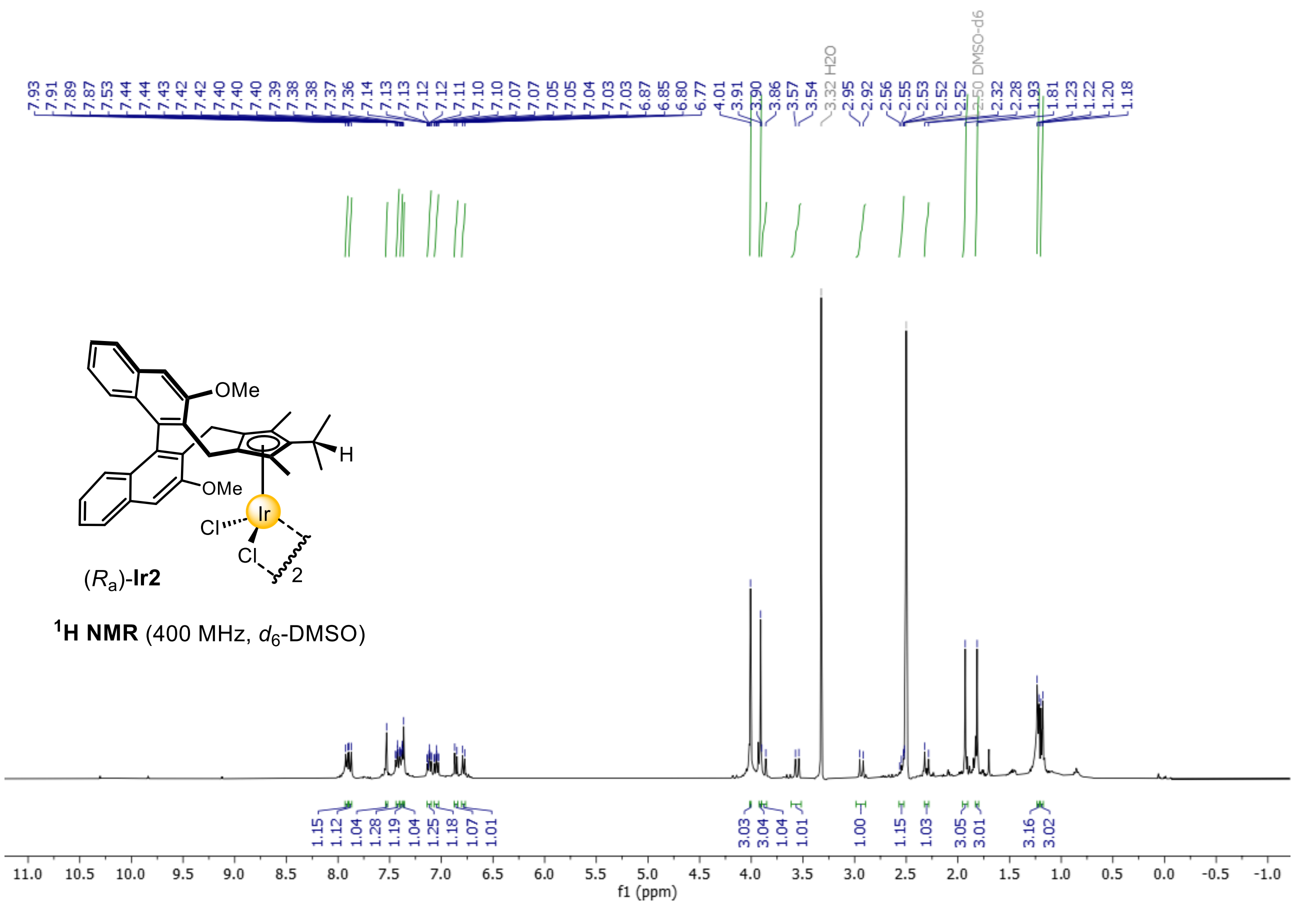
7.93 7.91 7.89 7.87 7.53 7.44 7.44 7.43 7.42 7.42 7.40 7.40 7.40 7.39 7.38 7.38 7.37 7.36 7.14 7.13 7.13 7.12 7.12 7.11 7.10 7.10 7.07 7.07 7.05 7.05 7.04 7.03 7.03 6.87 6.85 6.80 6.77 4.01 3.91 3.90 3.86 3.57 3.54 3.32 H2O 2.95 2.92 2.56 2.55 2.53 2.52 2.52 2.50 DMSO-d6 2.32 2.28 1.93 1.81 1.23 1.22 1.20 1.18
OMe
OMe
H
Cl
Ir
Cl
2
(Ra)-Ir2
1H NMR (400 MHz, d6-DMSO)
1.15 1.12 1.04 1.28 1.19 1.04 1.25 1.18 1.07 1.01 3.03 3.04 1.04 1.01 1.00 1.15 1.03 3.05 3.01 3.16 3.02
11.0 10.5 10.0 9.5 9.0 8.5 8.0 7.5 7.0 6.5 6.0 5.5 5.0 4.5 4.0 3.5 3.0 2.5 2.0 1.5 1.0 0.5 0.0 -0.5 -1.0
f1 (ppm)

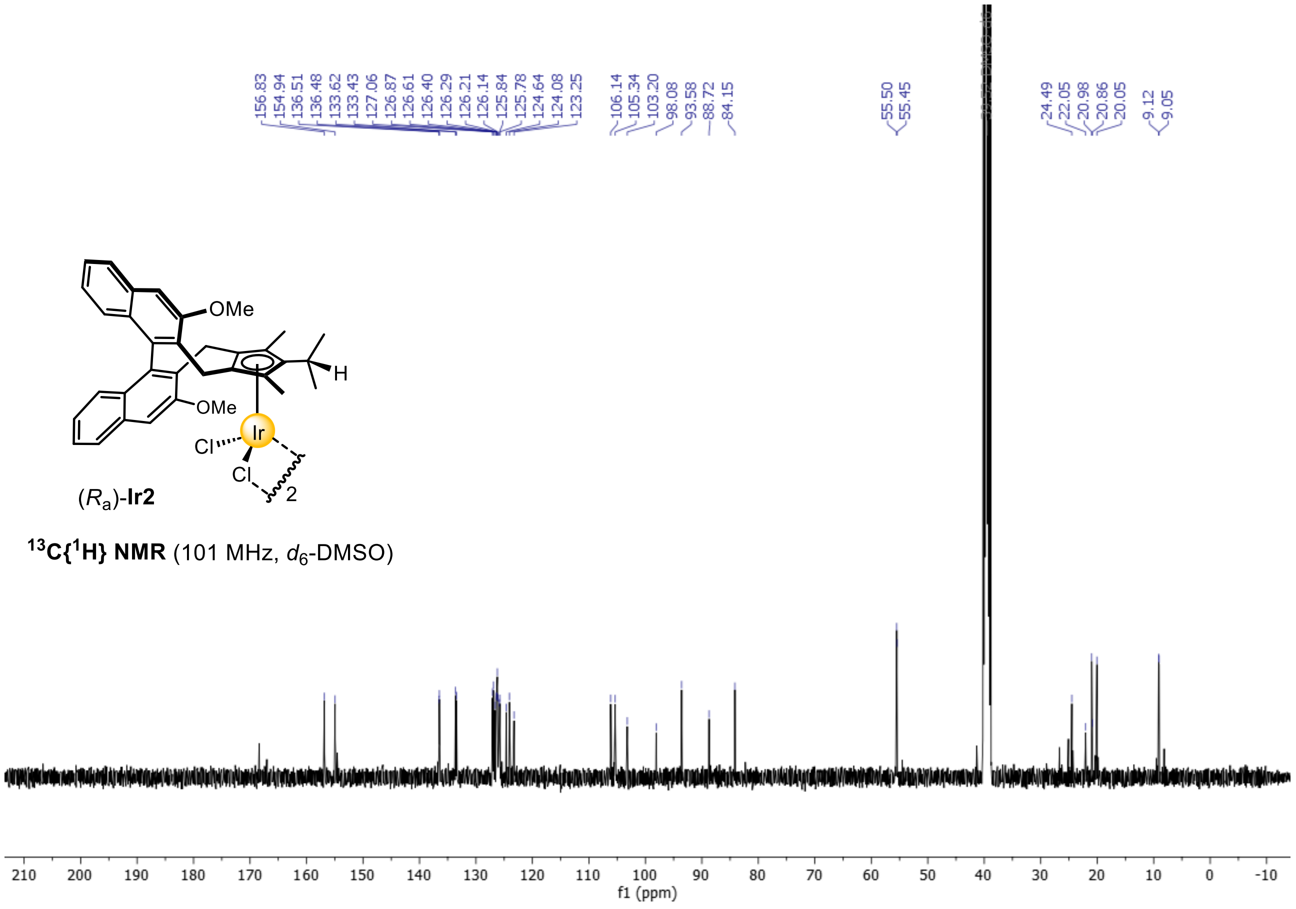
156.83 154.94 136.51 136.48 133.62 133.43 127.06 126.87 126.61 126.40 126.29 126.21 126.14 125.84 125.78 124.64 124.08 123.25 106.14 105.34 103.20 98.08 93.58 88.72 84.15 55.50 55.45 24.49 22.05 20.98 20.86 20.05 9.12 9.05
OMe
OMe
H
Cl
Ir
Cl
2
(Ra)-Ir2
13C{1H} NMR (101 MHz, d6-DMSO)
210 200 190 180 170 160 150 140 130 120 110 100 90 80 70 60 50 40 30 20 10 0 -10
f1 (ppm)

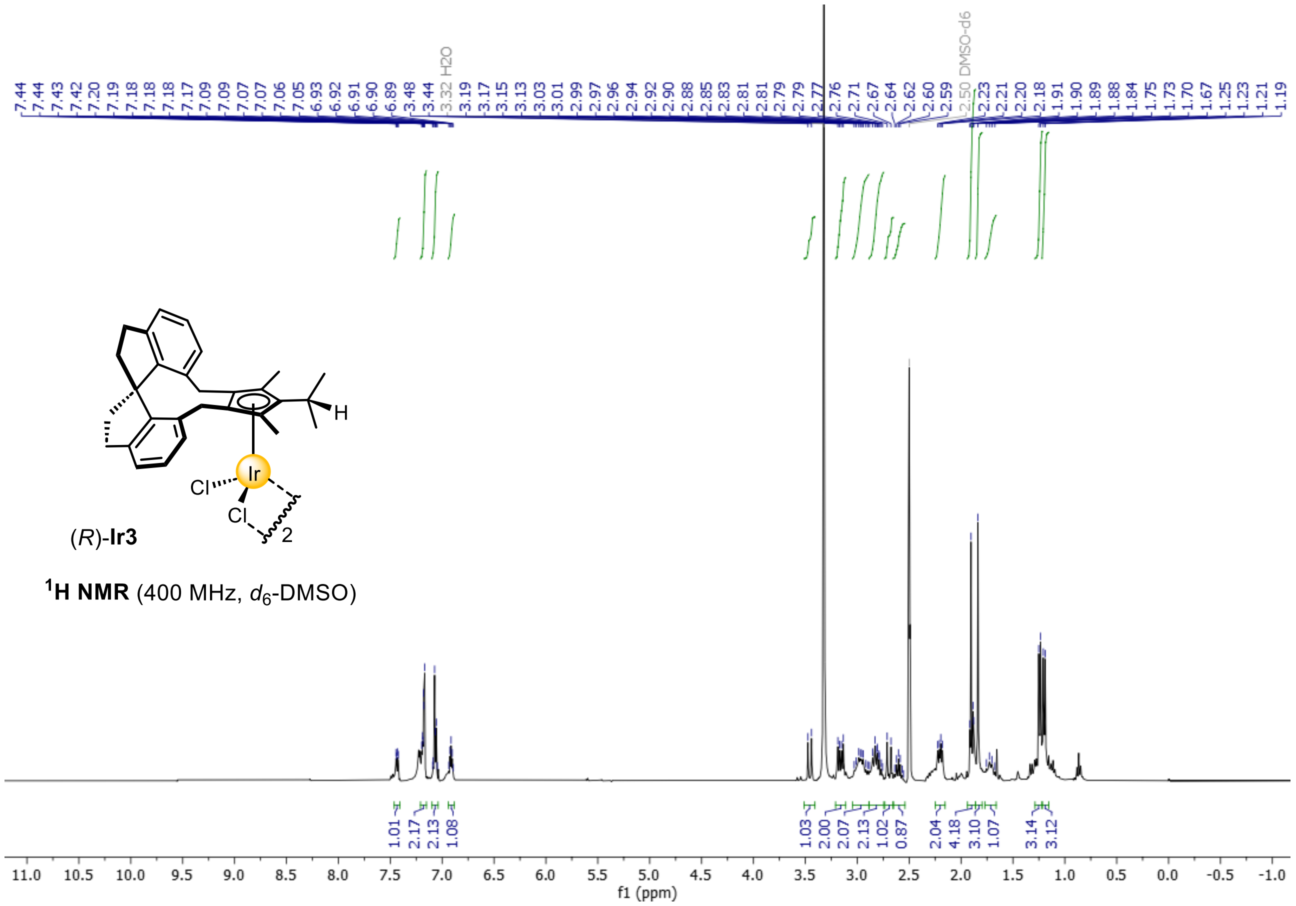
7.44
7.44
7.43
7.42
7.20
7.19
7.18
7.18
7.18
7.17
7.09
7.09
7.07
7.07
7.06
7.05
6.93
6.92
6.91
6.90
6.89
3.48
3.44
3.32 H2O
3.19
3.17
3.15
3.13
3.03
3.01
2.99
2.97
2.96
2.94
2.92
2.90
2.88
2.85
2.83
2.81
2.81
2.79
2.79
2.77
2.76
2.71
2.67
2.64
2.62
2.60
2.59
2.50 DMSO-d6
2.23
2.21
2.20
2.18
1.91
1.90
1.89
1.88
1.84
1.75
1.73
1.70
1.67
1.25
1.23
1.21
1.19
H
Cl
Ir
Cl
2
(R)-Ir3
1H NMR (400 MHz, d6-DMSO)
1.01
2.17
2.13
1.08
1.03
2.00
2.07
2.13
1.02
0.87
2.04
4.18
3.10
1.07
3.14
3.12
11.0 10.5 10.0 9.5 9.0 8.5 8.0 7.5 7.0 6.5 6.0 5.5 5.0 4.5 4.0 3.5 3.0 2.5 2.0 1.5 1.0 0.5 0.0 -0.5 -1.0
f1 (ppm)

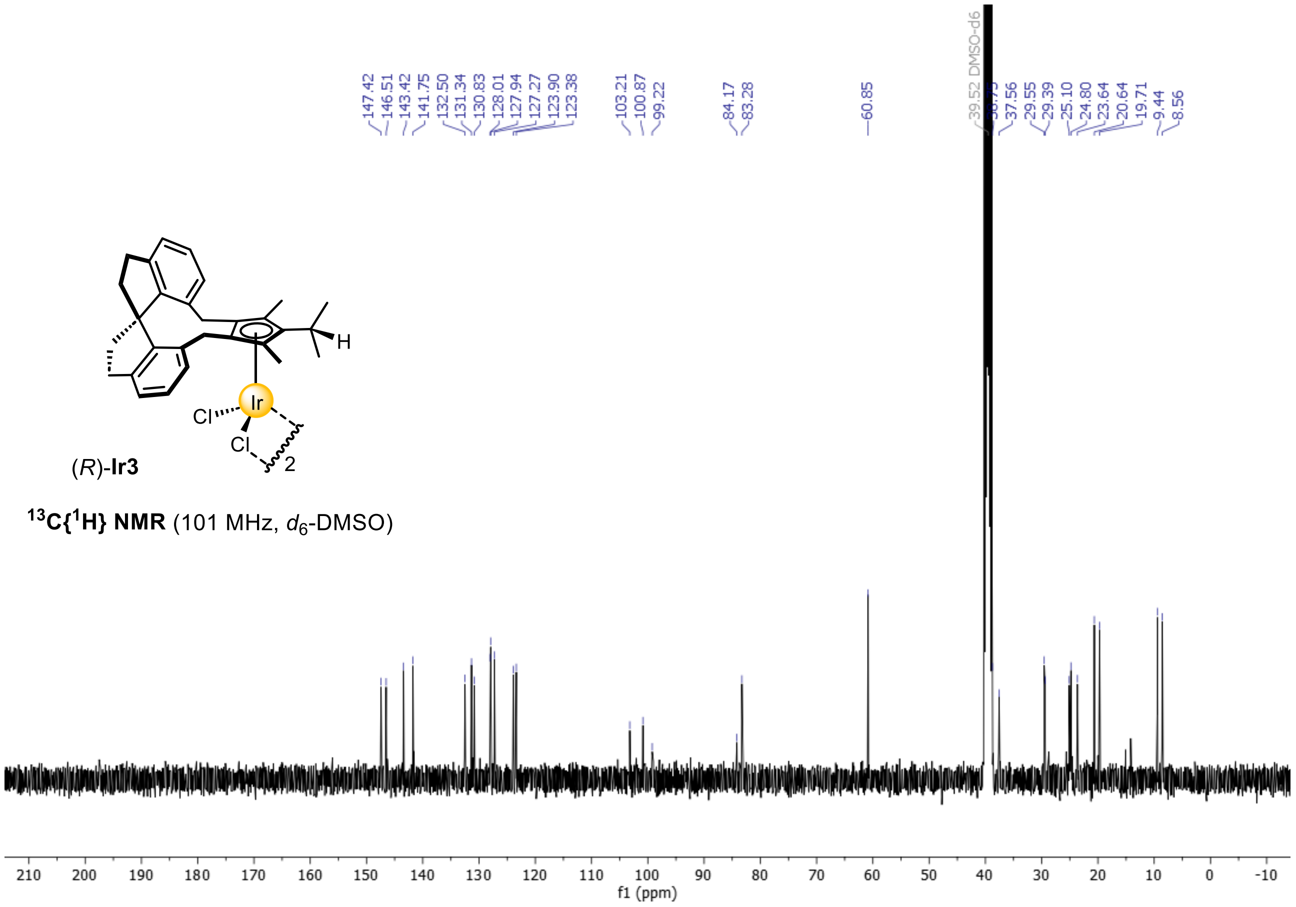
147.42
146.51
143.42
141.75
132.50
131.34
130.83
128.01
127.94
127.27
123.90
123.38
103.21
100.87
99.22
84.17
83.28
60.85
39.52 DMSO-d6
37.56
29.55
29.39
25.10
24.80
23.64
20.64
19.71
9.44
8.56
H
Cl
Ir
Cl
2
(R)-Ir3
13C{1H} NMR (101 MHz, d6-DMSO)
210 200 190 180 170 160 150 140 130 120 110 100 90 80 70 60 50 40 30 20 10 0 -10
f1 (ppm)

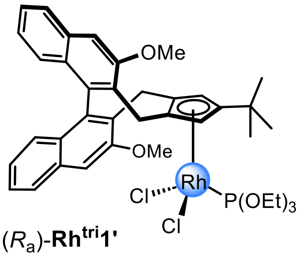

(R_a)-**Rh^{tri}1'**

^{31}P{^{1}H} NMR (162 MHz, CD_2Cl_2)

[in situ]

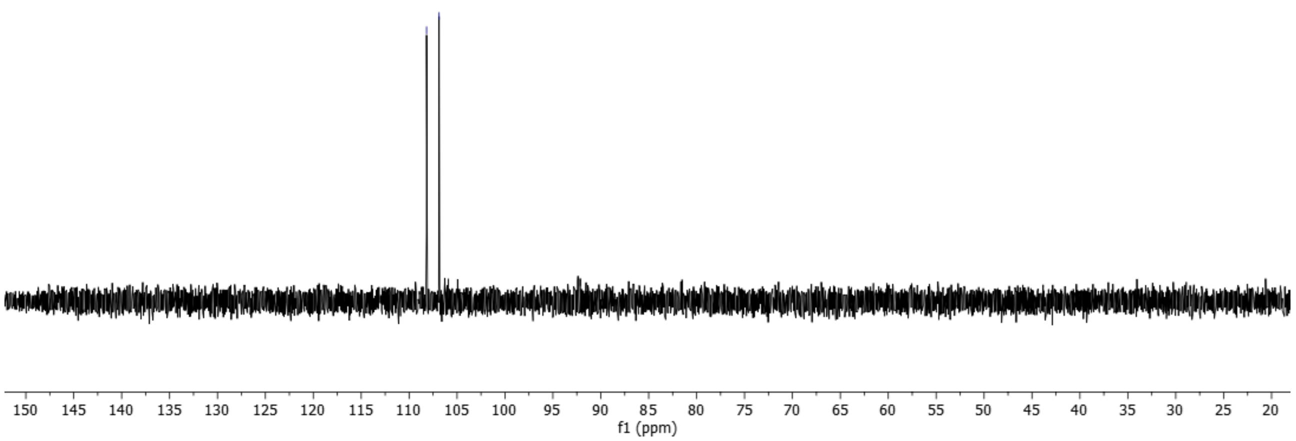

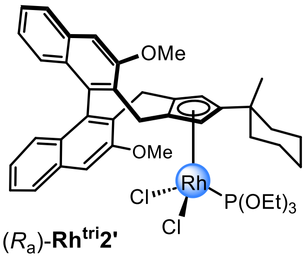

(R_a)-**Rh^{tri}2'**

^{31}P{^{1}H} NMR (162 MHz, CD_2Cl_2)

[in situ]

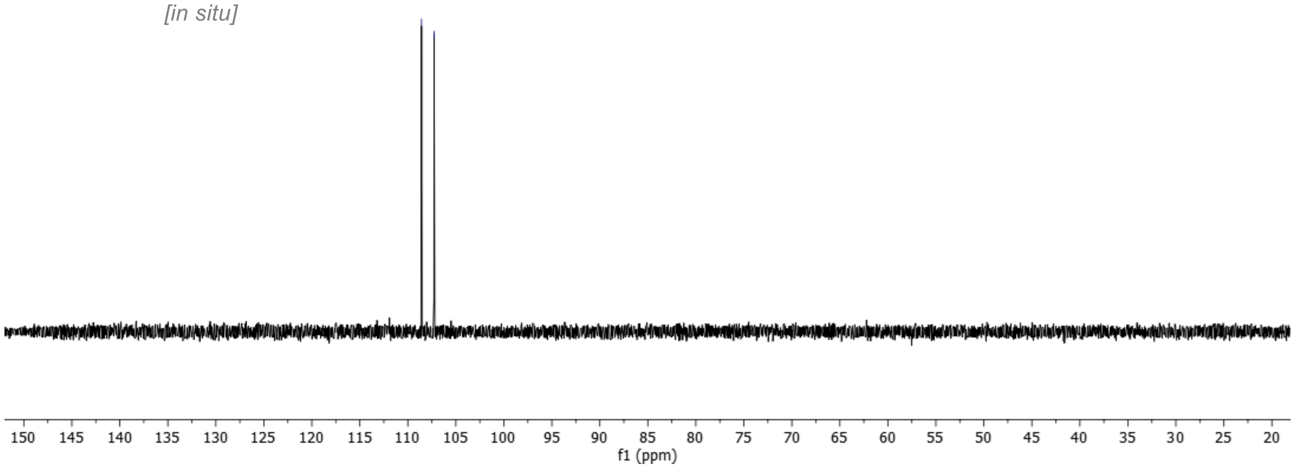

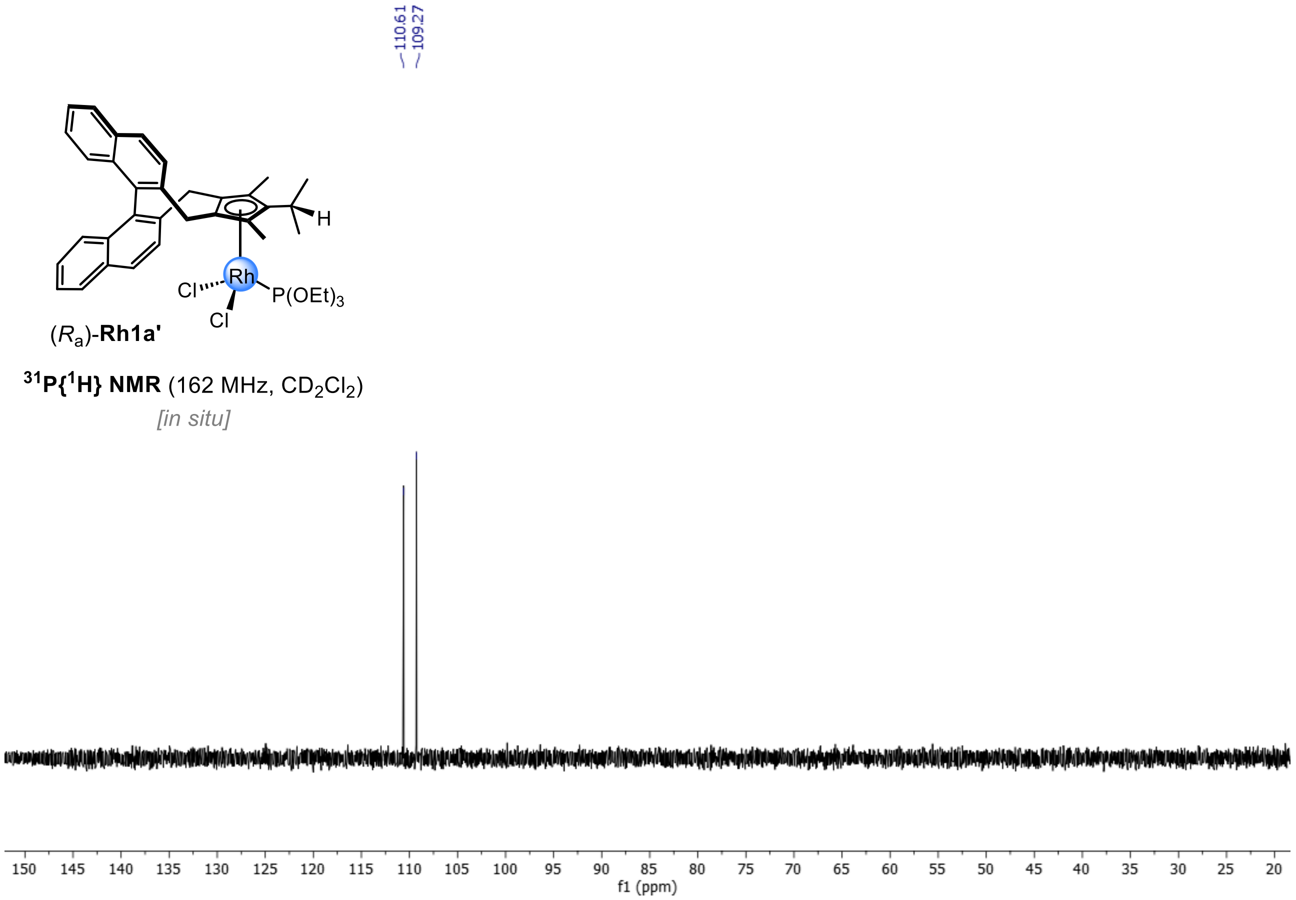
110.61
109.27
Cl
Rh
P(OEt)3
Cl
H
(Ra)-Rh1a'
31P{1H} NMR (162 MHz, CD2Cl2)
[in situ]
150 145 140 135 130 125 120 115 110 105 100 95 90 85 80 75 70 65 60 55 50 45 40 35 30 25 20
f1 (ppm)

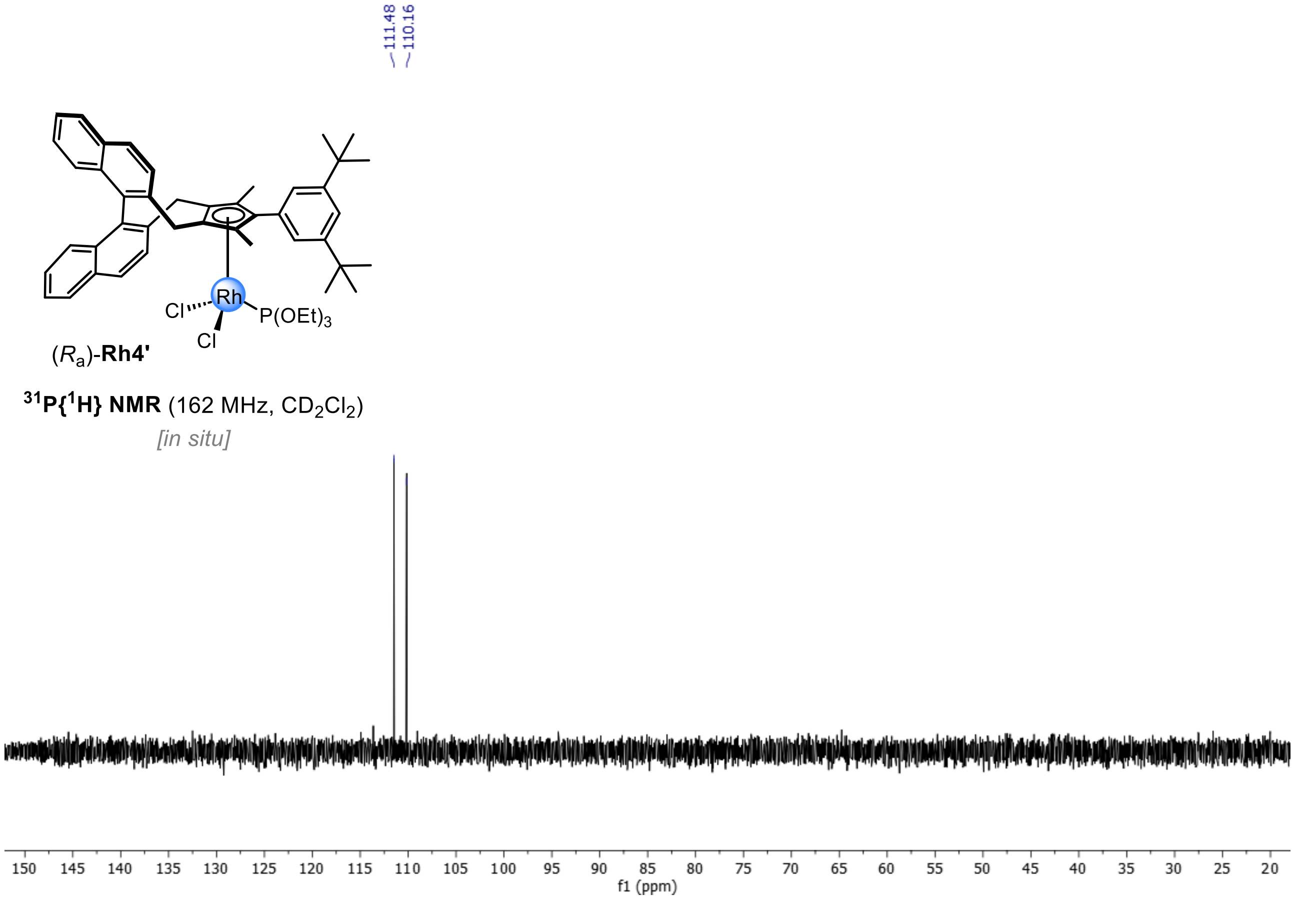
111.48
110.16
Cl
Rh
P(OEt)3
Cl
(Ra)-Rh4'
31P{1H} NMR (162 MHz, CD2Cl2)
[in situ]
150 145 140 135 130 125 120 115 110 105 100 95 90 85 80 75 70 65 60 55 50 45 40 35 30 25 20
f1 (ppm)

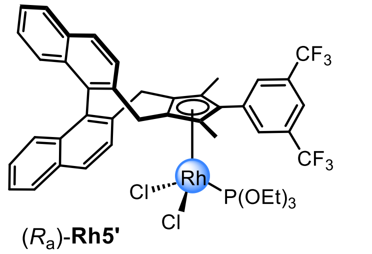

(R_a)-**Rh5'**

$^{31}P\{^1H\}$ NMR (162 MHz, CD_2Cl_2)

[in situ]

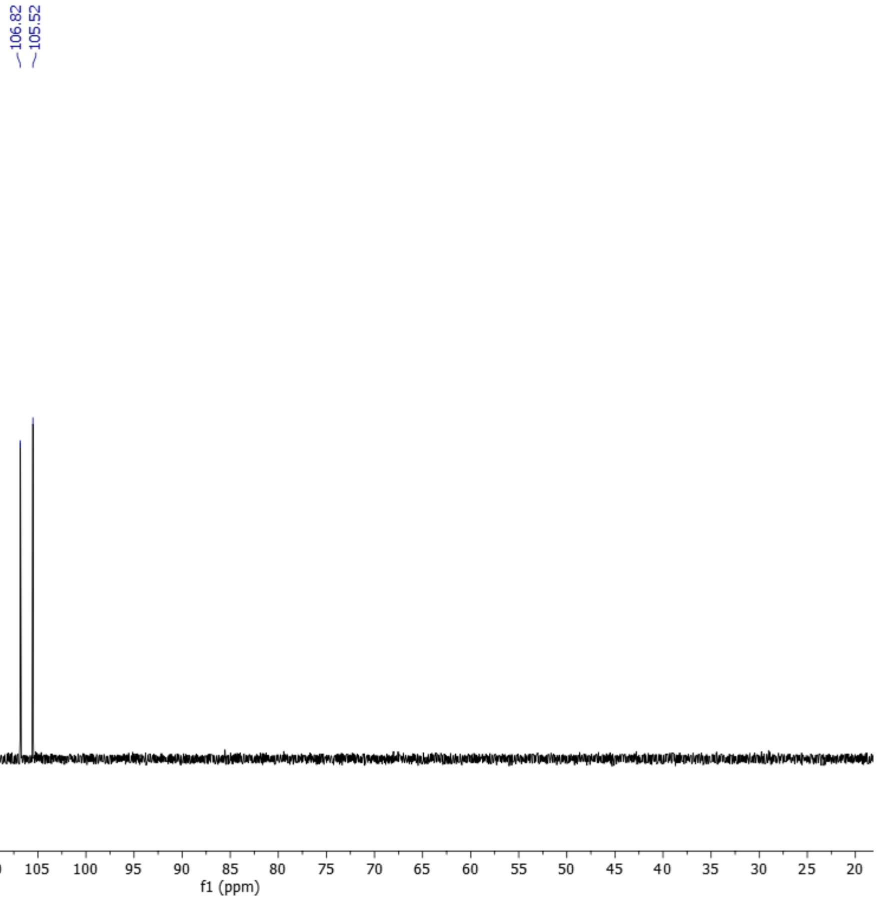

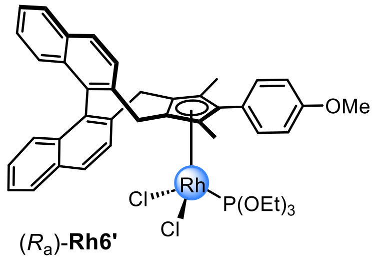

(R_a)-**Rh6'**

$^{31}P\{^1H\}$ NMR (162 MHz, CD_2Cl_2)

[in situ]

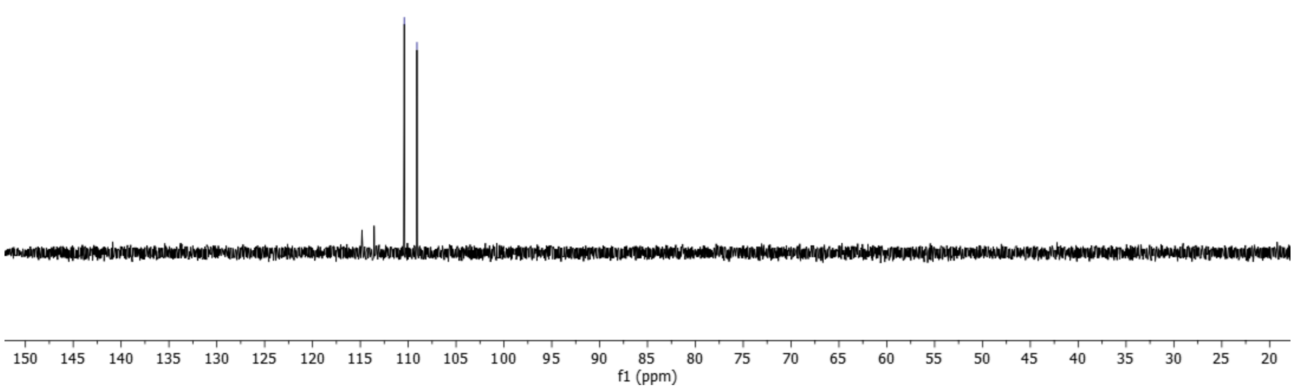

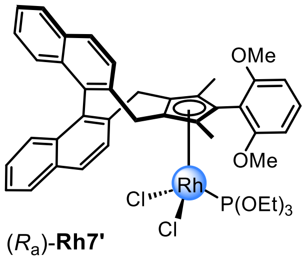

(R_a)-**Rh7'**

^{31}P{^{1}H} NMR (162 MHz, CD_2Cl_2)

[in situ]

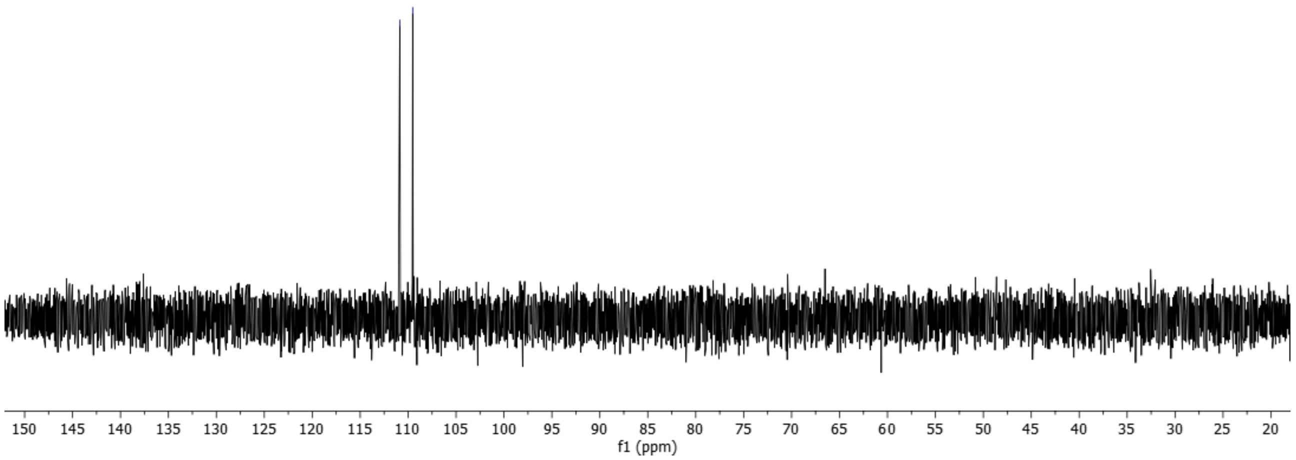

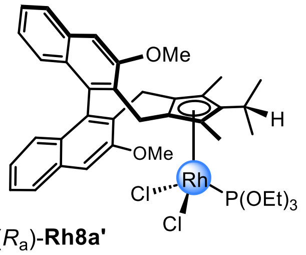

(R_a)-**Rh8a'**

^{31}P{^{1}H} NMR (162 MHz, CD_2Cl_2)

[in situ]

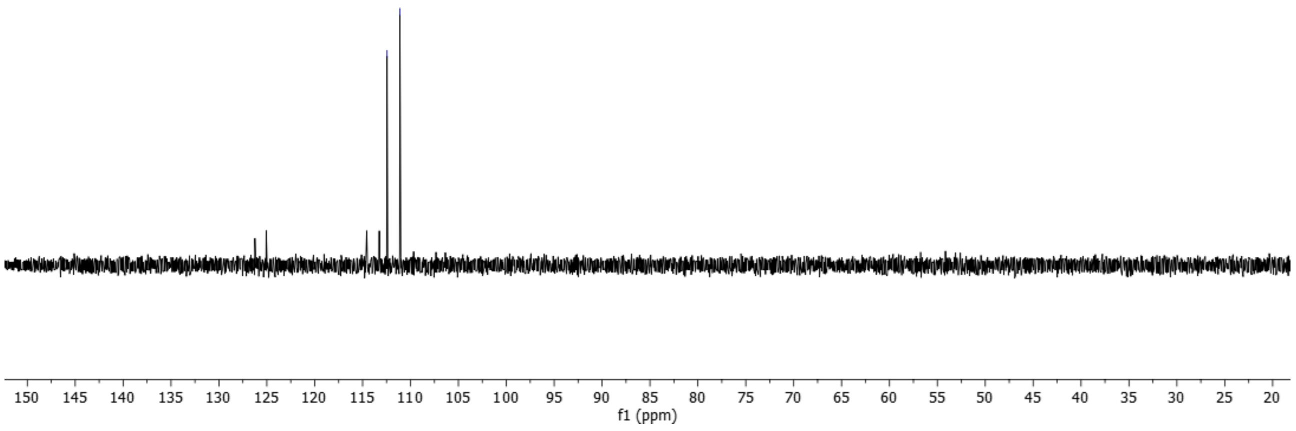

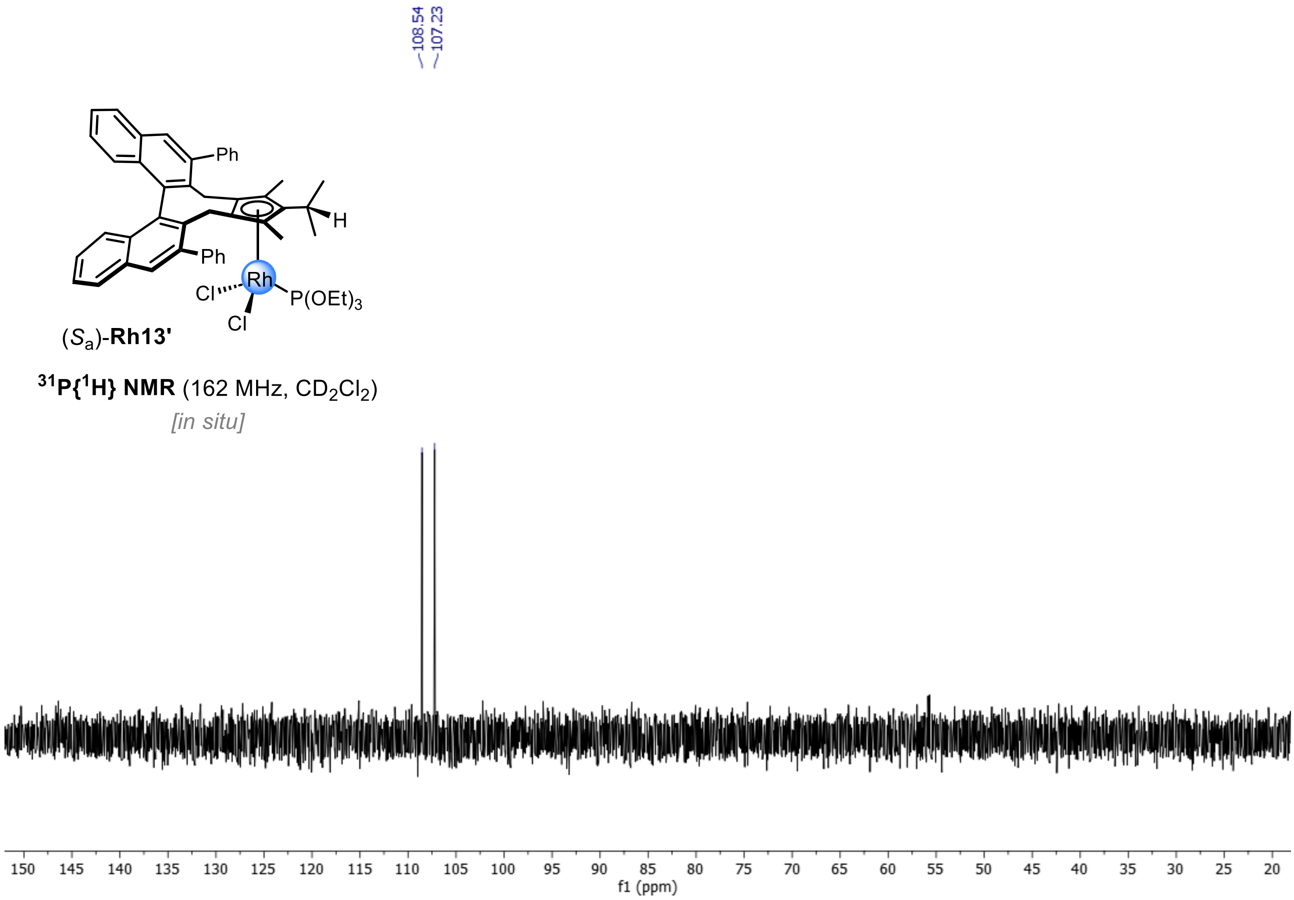
108.54
107.23
Ph
H
Ph
Cl
Rh
P(OEt)3
Cl
(Sa)-Rh13'
31P{1H} NMR (162 MHz, CD2Cl2)
[in situ]
150 145 140 135 130 125 120 115 110 105 100 95 90 85 80 75 70 65 60 55 50 45 40 35 30 25 20
f1 (ppm)

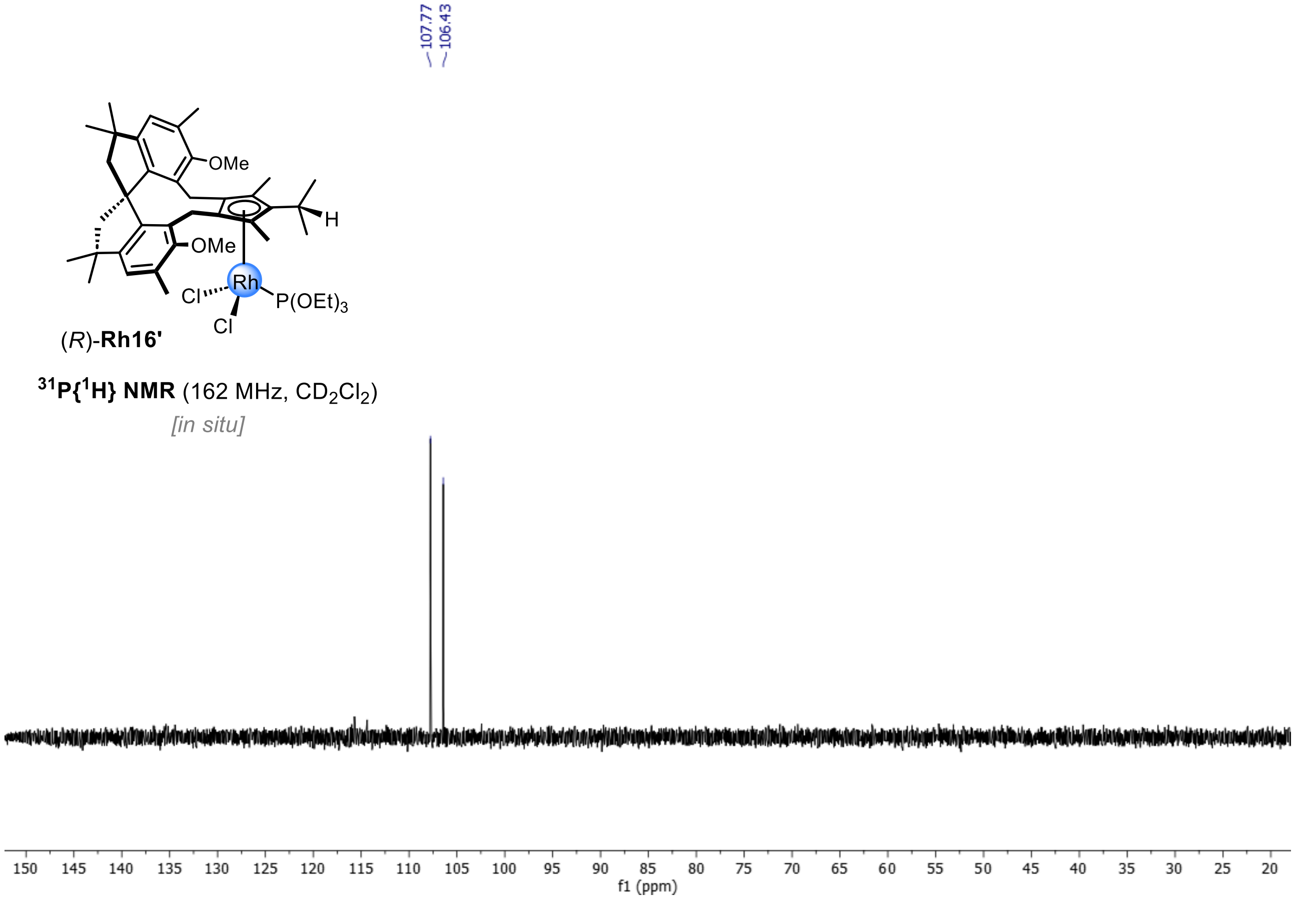
107.77
106.43
OMe
H
OMe
Cl
Rh
P(OEt)3
Cl
(R)-Rh16'
31P{1H} NMR (162 MHz, CD2Cl2)
[in situ]
150 145 140 135 130 125 120 115 110 105 100 95 90 85 80 75 70 65 60 55 50 45 40 35 30 25 20
f1 (ppm)

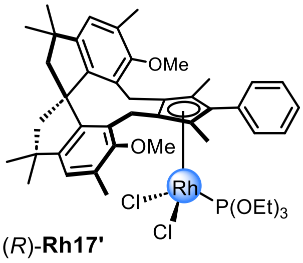

(*R*)-**Rh17'**

^{31}P{^{1}H} NMR (162 MHz, CD_2Cl_2)

[in situ]

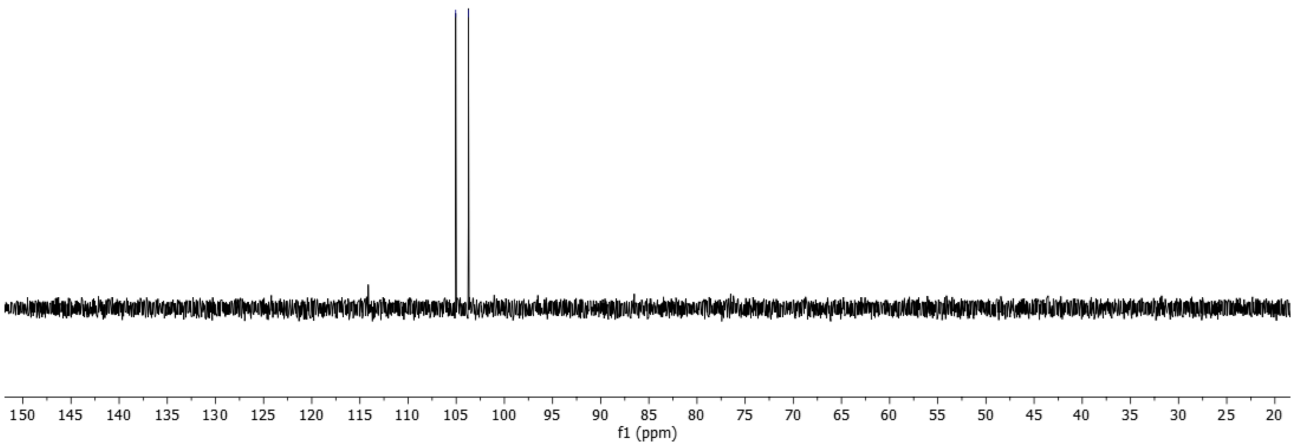

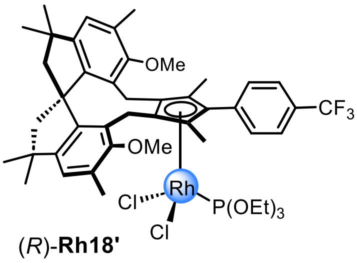

(*R*)-**Rh18'**

^{31}P{^{1}H} NMR (162 MHz, CD_2Cl_2)

[in situ]

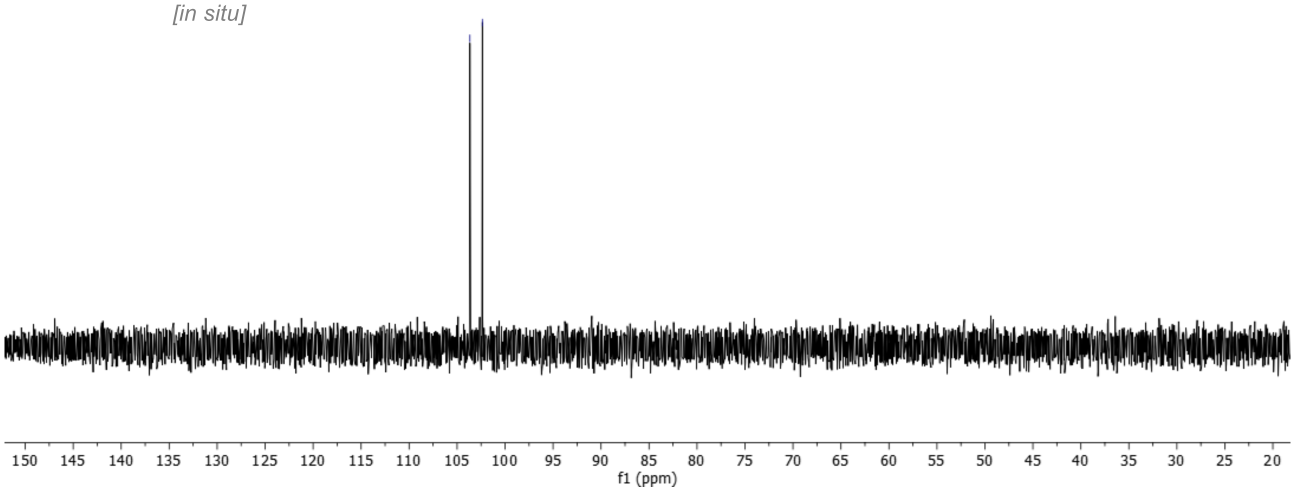

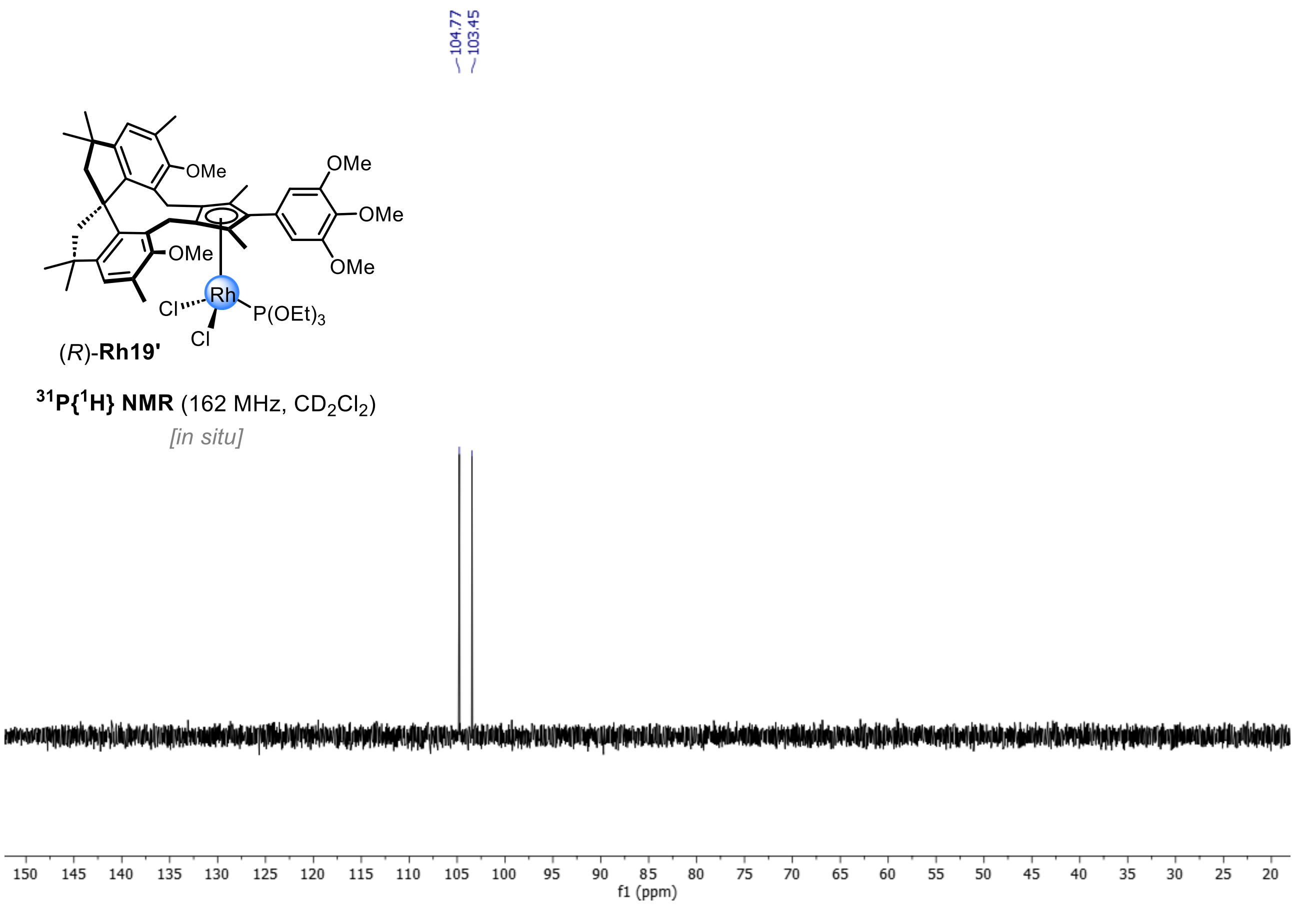

(*R*)-**Rh19'**

^{31}P{^{1}H} NMR (162 MHz, CD_2Cl_2)

[in situ]

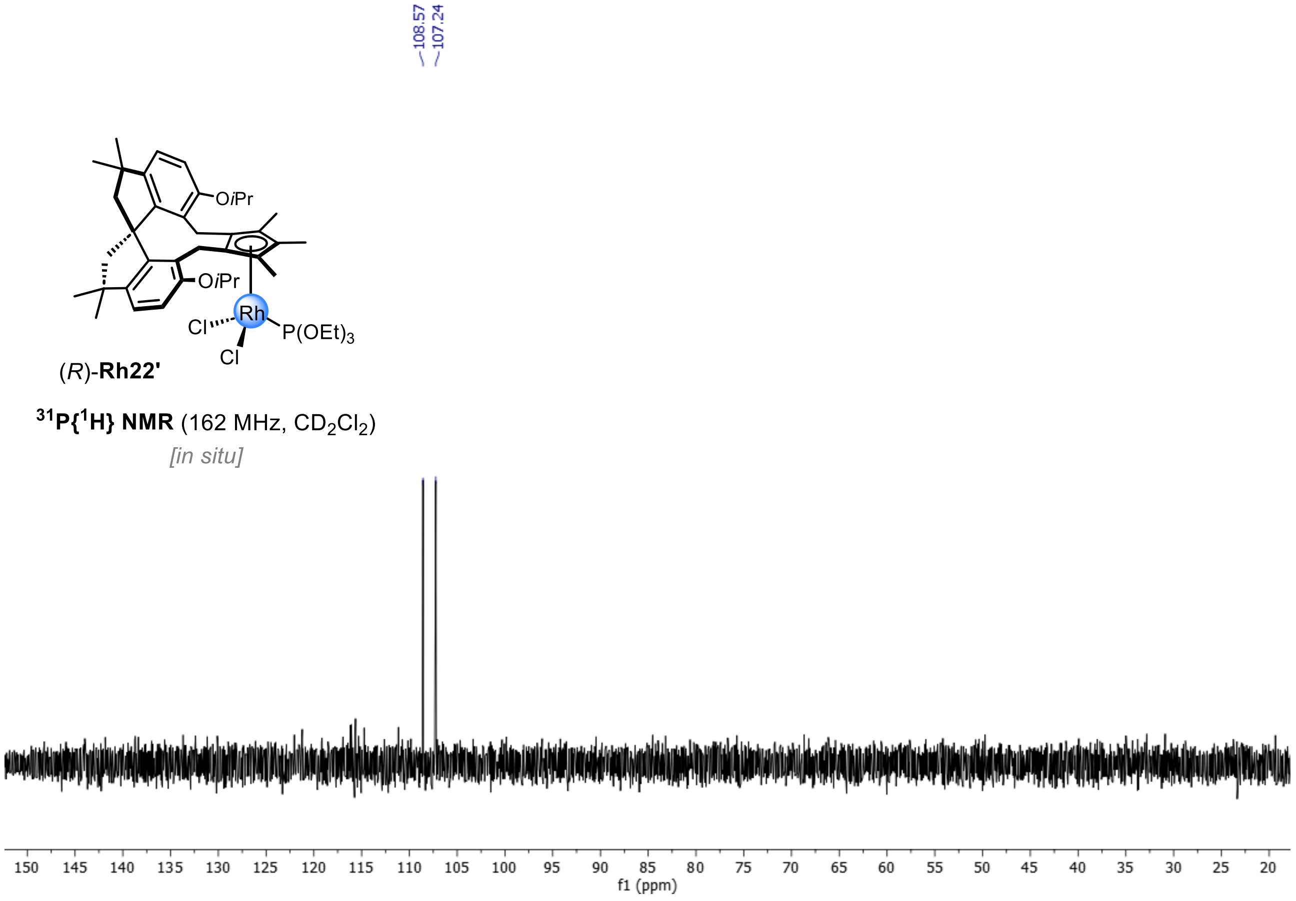

(*R*)-**Rh22'**

^{31}P{^{1}H} NMR (162 MHz, CD_2Cl_2)

[in situ]

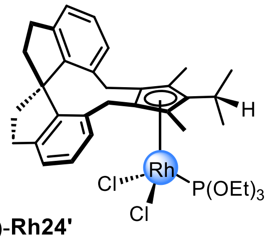

(*R*)-**Rh24'**

^{31}P{^{1}H} NMR (162 MHz, CD_2Cl_2)

[in situ]

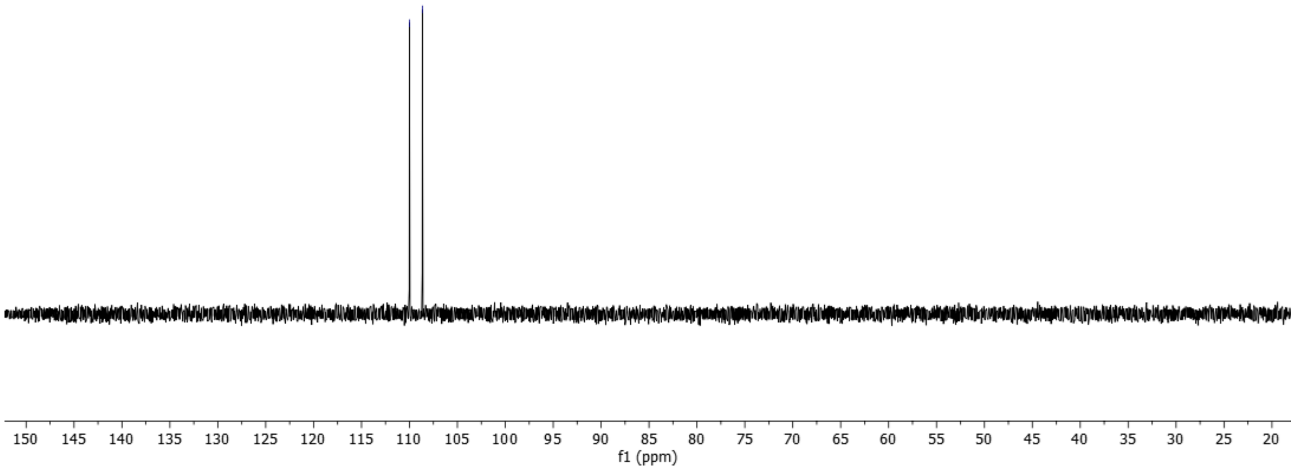

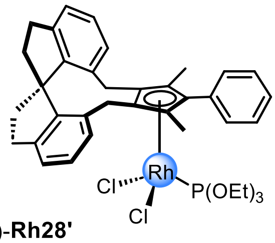

(*R*)-**Rh28'**

^{31}P{^{1}H} NMR (162 MHz, CD_2Cl_2)

[in situ]

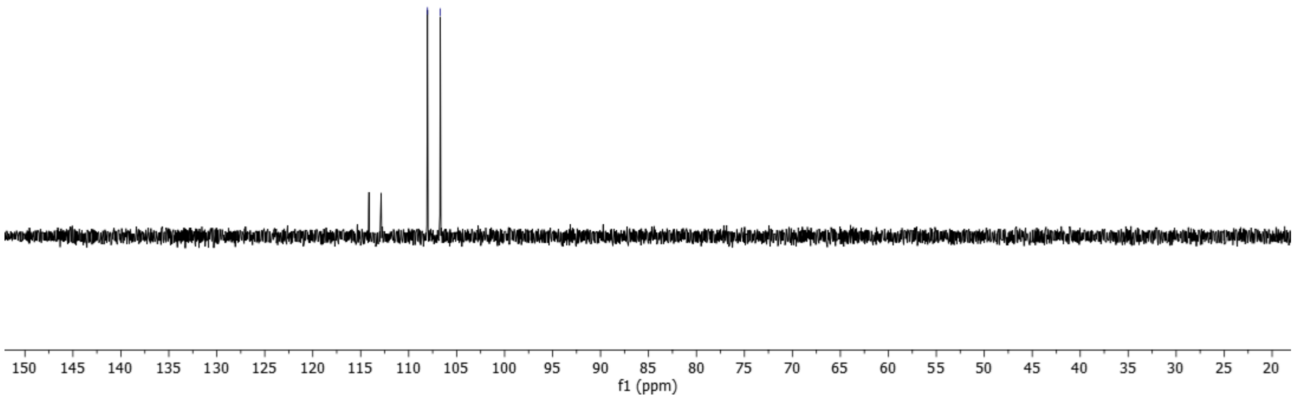

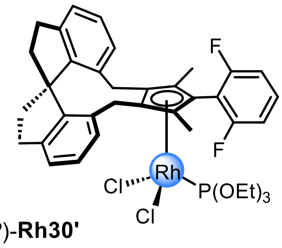

(*R*)-**Rh30'**

^{31}P{^{1}H} NMR (162 MHz, CD_2Cl_2)

[in situ]

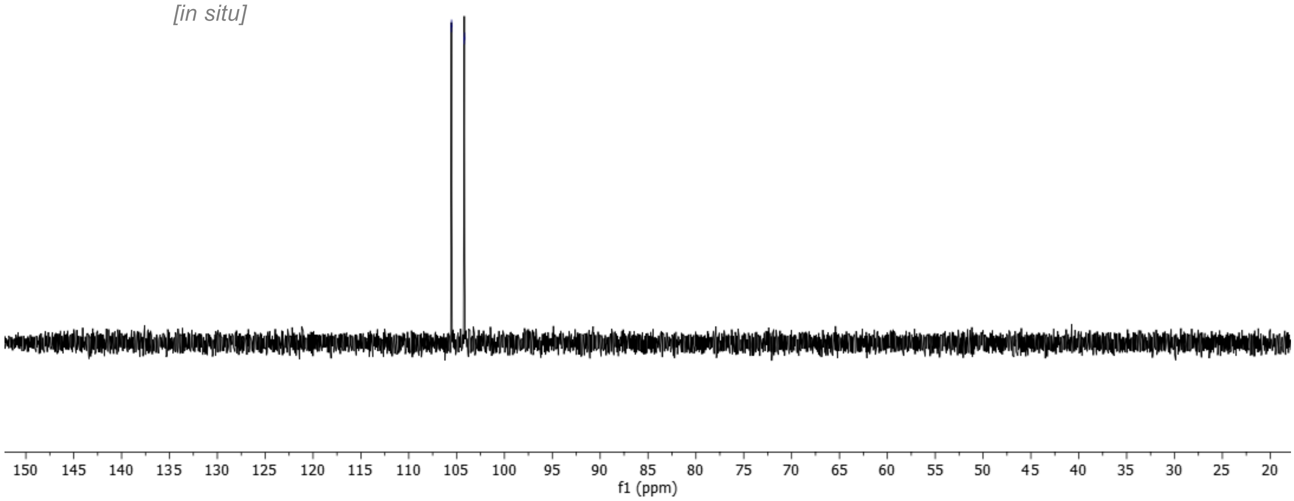

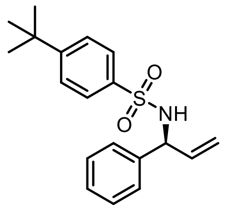

(*S*)-**3j**

^{1}H NMR (400 MHz, $CDCl_3$)

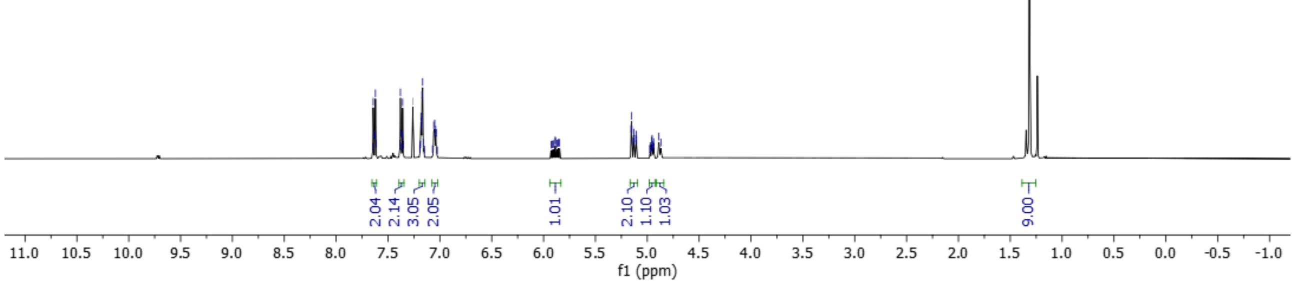

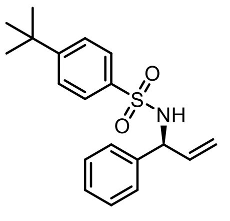

(*S*)-**3j**

^{13}C{^{1}H} NMR (101 MHz, $CDCl_3$)

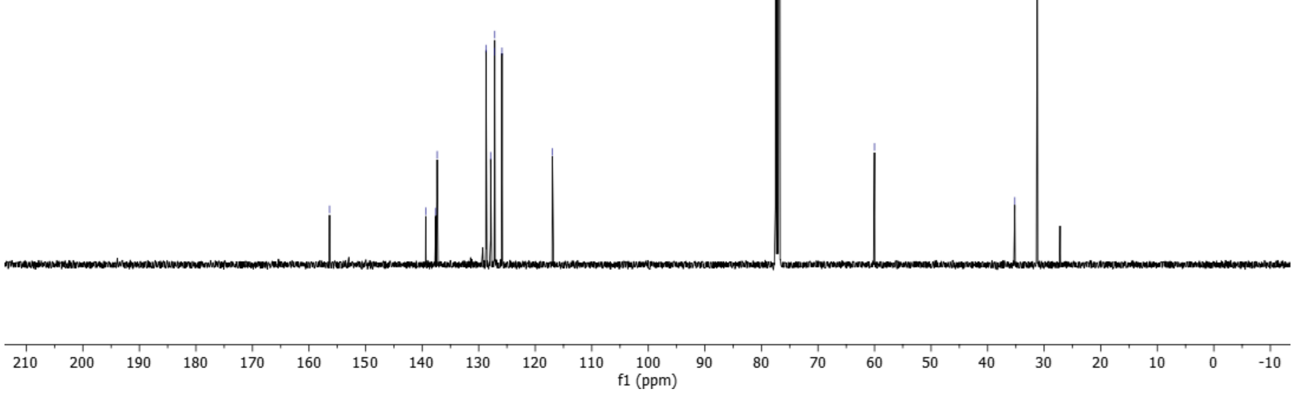

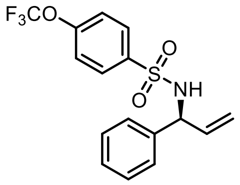

(*S*)-**3m**

^{1}H NMR (400 MHz, $CDCl_3$)

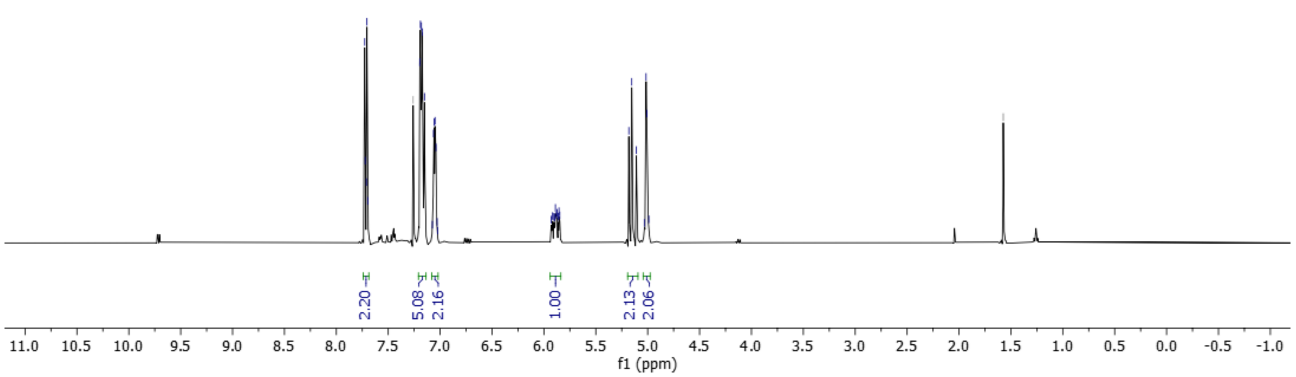

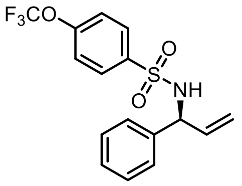

(*S*)-**3m**

^{13}C{^{1}H} NMR (101 MHz, $CDCl_3$)

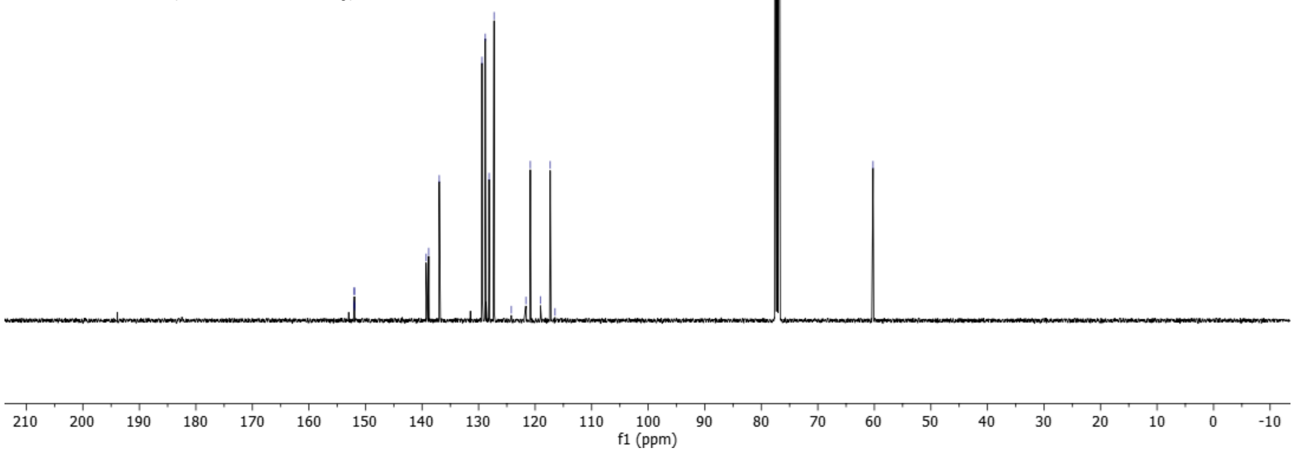

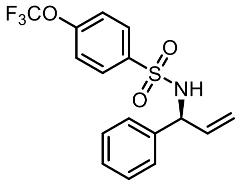

(*S*)-**3m**

^{19}F{^{1}H} NMR (376 MHz, $CDCl_3$)

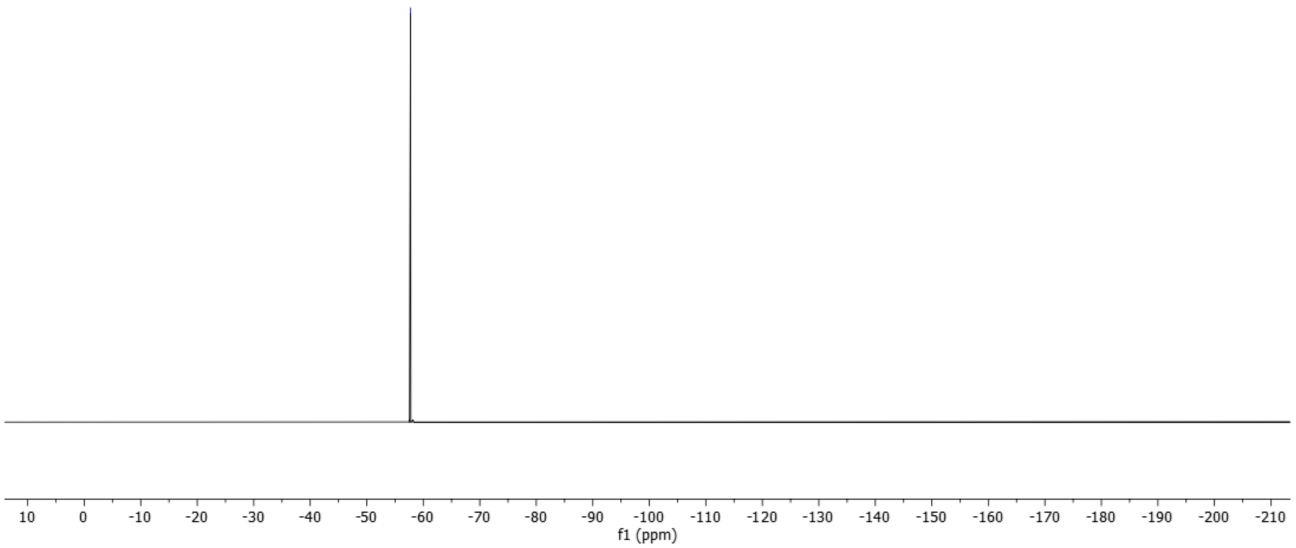

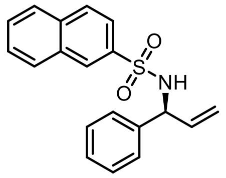

(*S*)-**3n**

^{1}H NMR (400 MHz, $CDCl_3$)

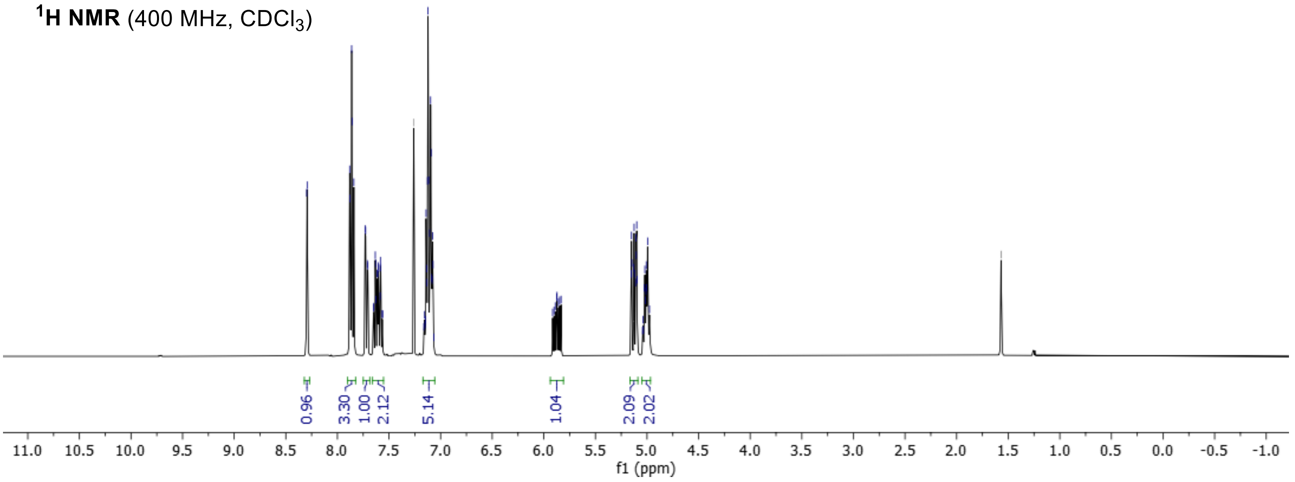

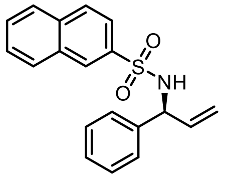

(*S*)-**3n**

^{13}C{^{1}H} NMR (101 MHz, $CDCl_3$)

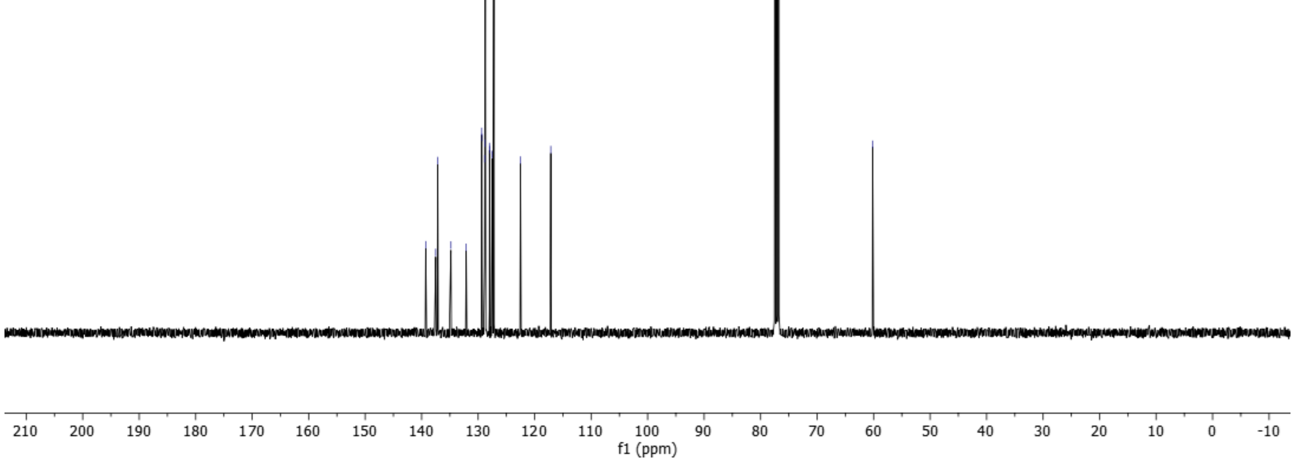

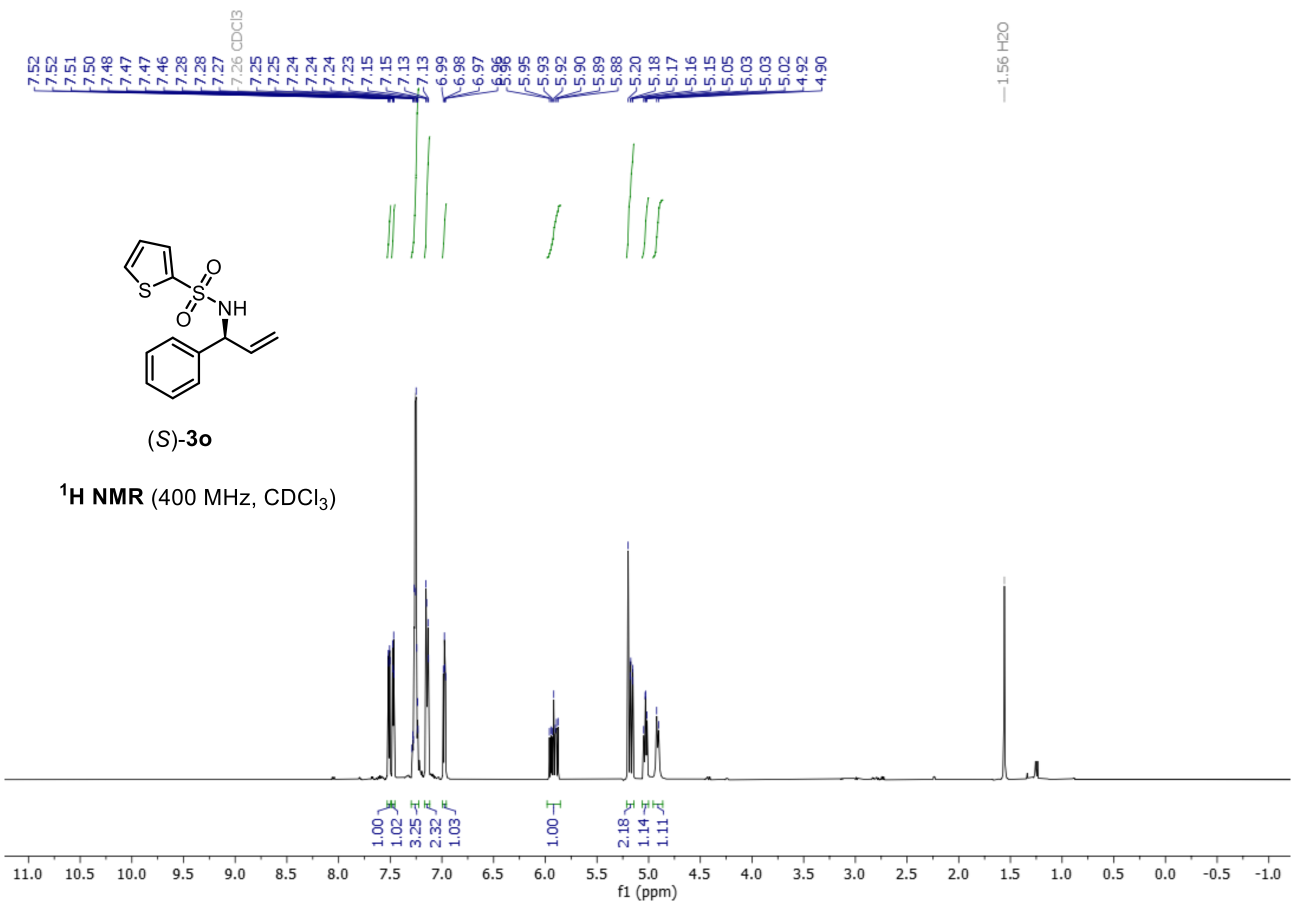

(*S*)-**3o**

^{1}H NMR (400 MHz, $CDCl_3$)

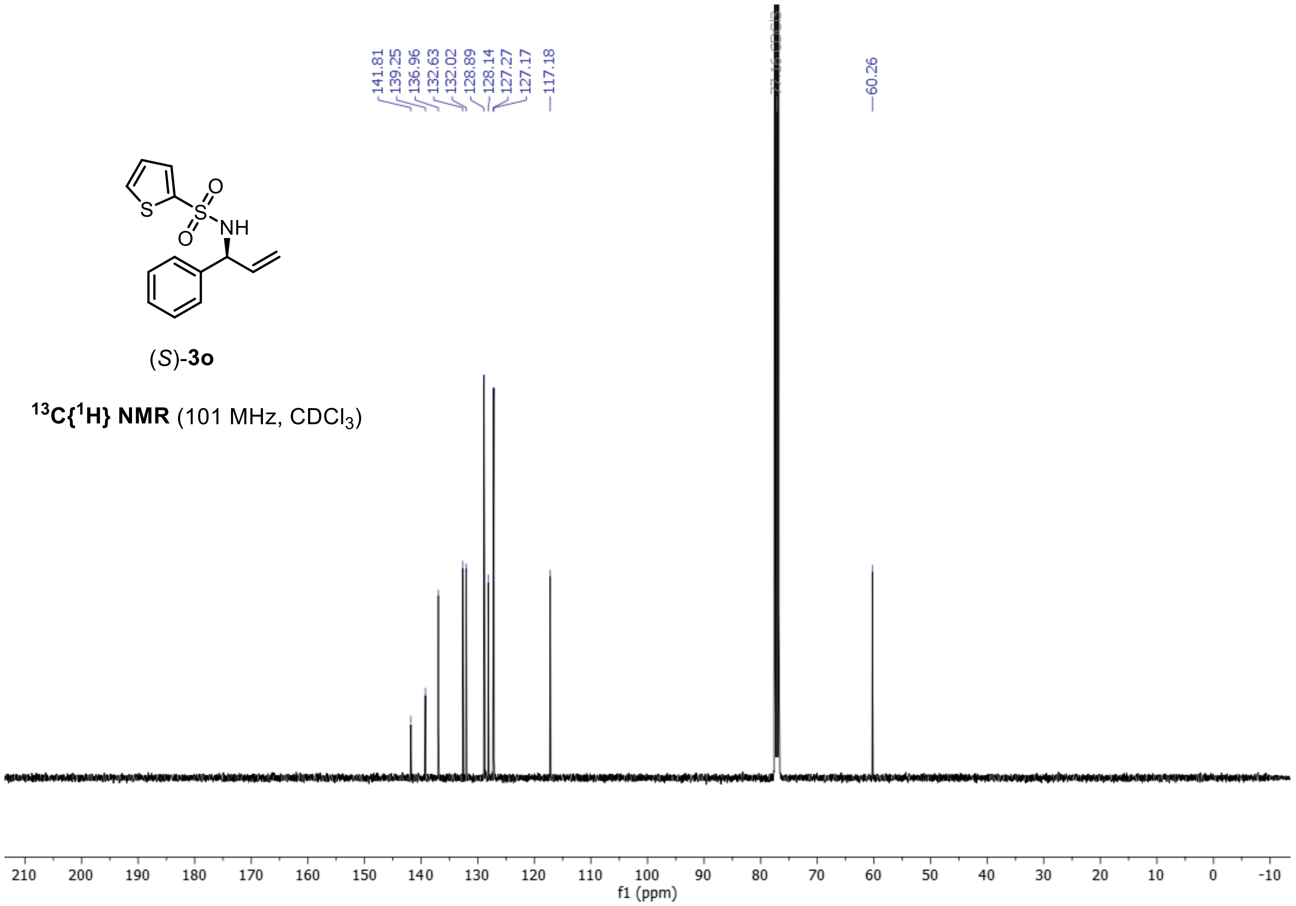

(*S*)-**3o**

^{13}C{^{1}H} NMR (101 MHz, $CDCl_3$)

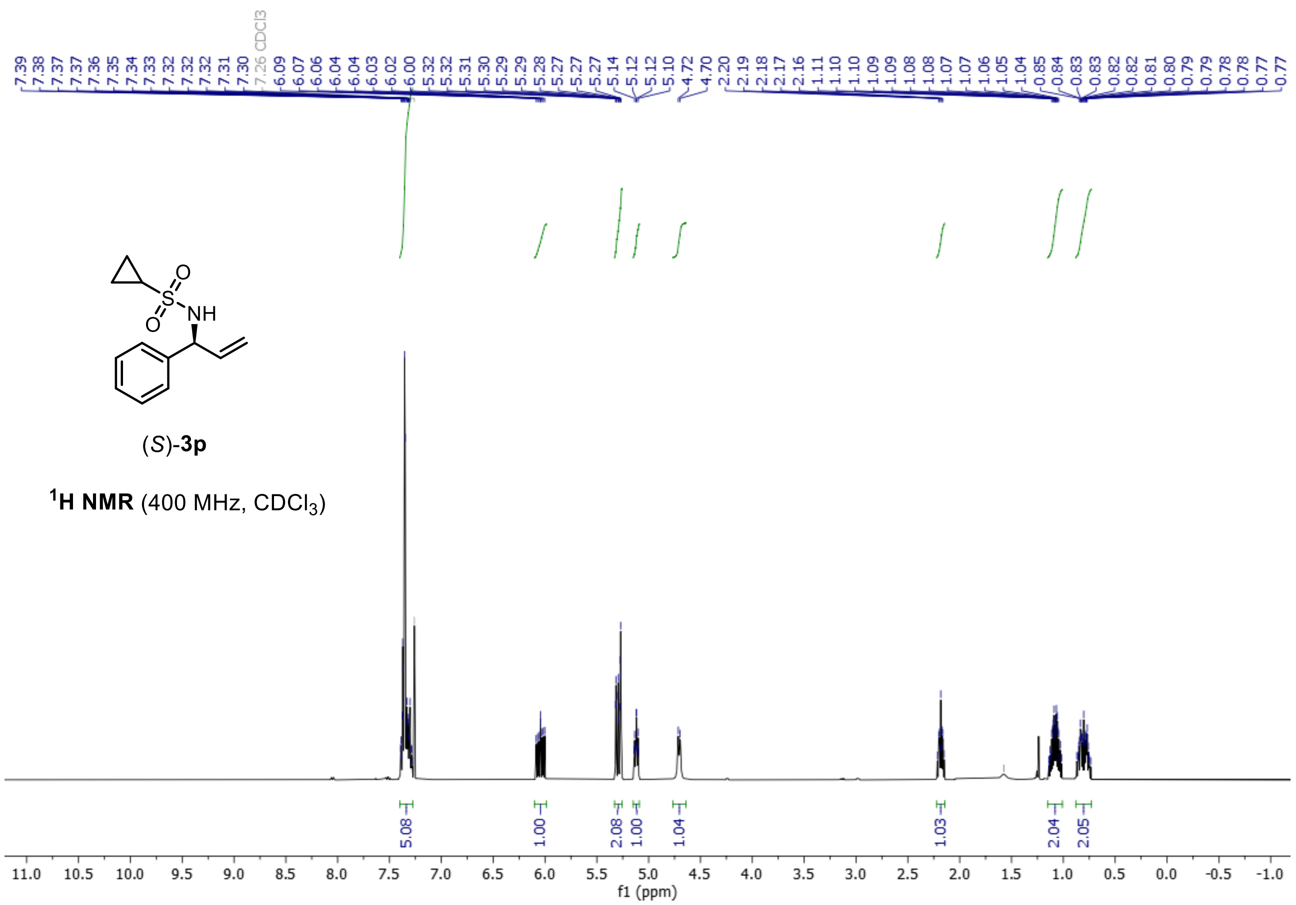
(S)-3p
1H NMR (400 MHz, CDCl3)
f1 (ppm)

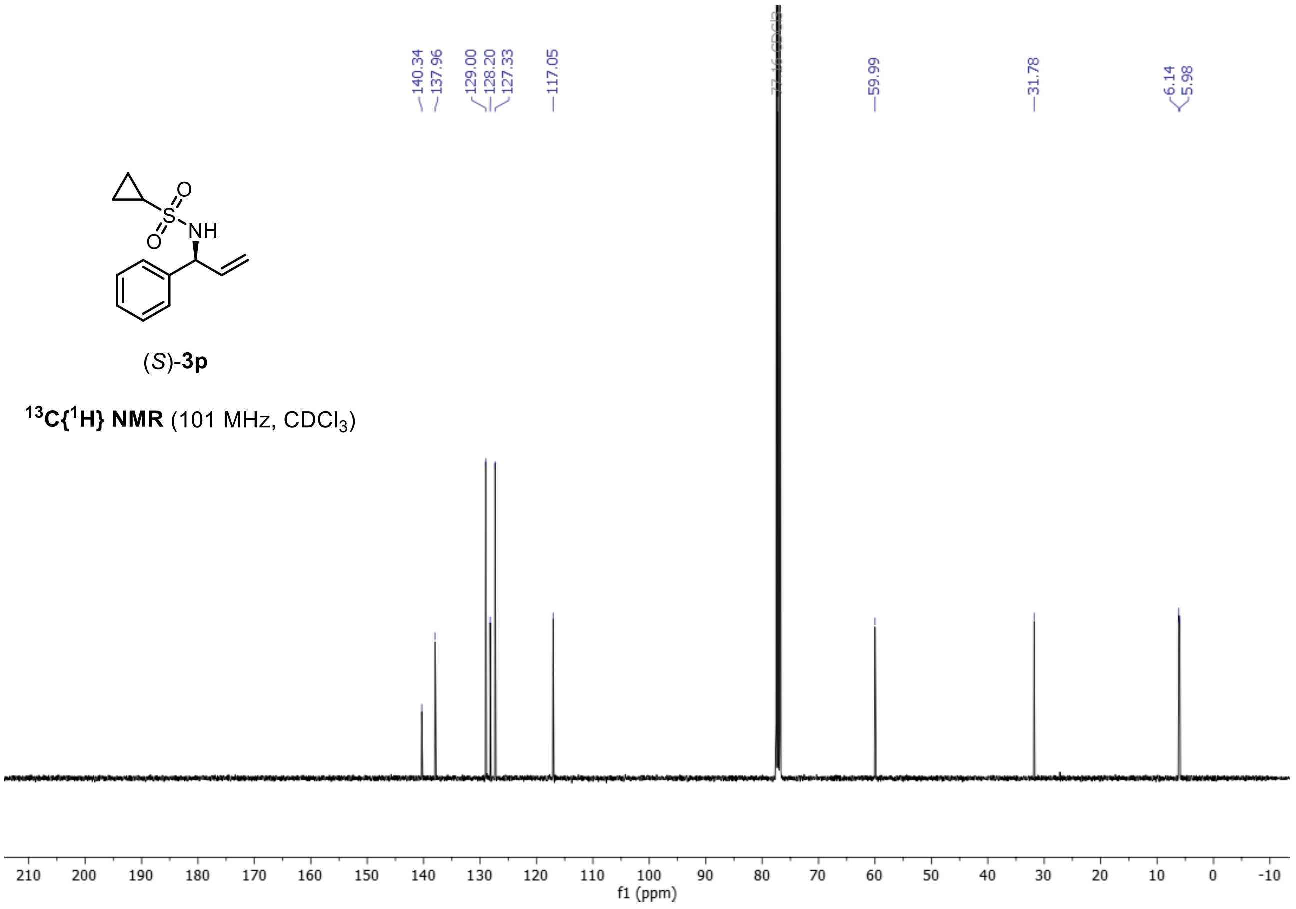
(S)-3p
13C{1H} NMR (101 MHz, CDCl3)
140.34
137.96
129.00
128.20
127.33
117.05
59.99
31.78
6.14
5.98
f1 (ppm)
